# Supplementary material for: Synthesis of N-acyl sulfenamides via copper catalysis and their use as S-sulfenylating reagents of thiols
Source: Nat Commun. 2022 Oct 28;13:6445. doi: 10.1038/s41467-022-34223-7 (PMC9616856; doi:10.1038/s41467-022-34223-7)
Supplement: Supplementary file 1 — Supplementary Information [file 41467_2022_34223_MOESM1_ESM.pdf]

Supplementary Information for

Synthesis of N-acyl sulfenamides via copper catalysis and their use as S-sulfenylating reagents of thiols

Ziqian Bai[1], Shiyang Zhu[1], Yiyao Hu[1], Peng Yang[1], Xin Chu[1], Gang He[1], Hao Wang[1]*, and Gong Chen[1, 2, 3]*

[1]State Key Laboratory and Institute of Elemento-Organic Chemistry, College of Chemistry, Nankai University, Tianjin 300071, China

[2]Frontiers Science Center for New Organic Matter, Nankai University, Tianjin 300192, China

[3]Haihe Laboratory of Sustainable Chemical Transformations, Tianjin 300192, China

Email: hao@nankai.edu.cn, gongchen@nankai.edu.cn

CONTENTS

Supplementary Methods S2

1.1 General information S2
1.2 General procedures for the preparation of dioxazolone substrates S3
1.3 General procedures for the preparation of mercapto substrates S13
1.4 General procedures for the S–N coupling S22
1.5 General procedures for the S–S coupling S49

Supplementary Discussion S64

2.1 Optimization of amidation of thiols with dioxazolones S64
2.2 One-pot synthesis of **47** and **51** S67
2.3 Comparisons of oxidative coupling of thiols with our developed S–S coupling methods S68

Supplementary Notes S69

3.1 X-ray crystallographic data of compound **7** S69
3.2 HPLC spectra of substrates **5a** and **46** S81
3.3 LC-MS of selected substrates S85
3.4 NMR spectra S96

Supplementary References S202

Supplementary Methods

1.1 General information

Reagents: All commercial materials were used as received unless otherwise noted. DCM was dried by distillation over CaH_2. THF was dried by distillation over sodium/benzophenone. TLC was performed on silica gel Huanghai HSGF254 plates and visualization of the developed chromatogram was performed by fluorescence quenching (λ_{max} = 254 nm). Flash chromatography was performed using Silica gel (200-300 mesh) purchased from Qingdao Haiyang Chemical Co. China. IPrCuCl (>95%) and Ag_2CO_3 (>98%) were purchased from Shanghai Bidepharm. CuOAc (>97%) was purchased from Strem Chemicals. $FeCl_2$ (>99.5%) was purchased from Macklin (Shanghai). $[Cp^*IrCl_2]_2$ was synthesized following a reported procedure using $IrCl_3.xH_2O$ (>99%, Shanghai Bokachem). Other metal catalysts were purchased from Sinocompound (Jiangsu). *i*PrSH was purchased from J&K Scientific (Beijing). Hexane and ethyl acetate were purchased from Adamas-beta (Shanghai). DCE was purchased from Energy Chemical. Deuterated solvents for NMR were purchased from Innochem Science & Technology co. (Beijing) and J&K Scientific (Beijing). Acetohydroxamic acid for the preparation of compound **2** was purchased from Aladdin Chemical. Most mercapto substrates and carboxylic acids were commercially available (Shanghai Bidepharm, Adamas-beta, Energy Chemical, J&K Scientific, Tianjin Heowns, Acros, Shanghai Leyan, Alfa, Aldrich, Meryer, TCI, 3AChem and others).

Instruments: NMR spectra were recorded on Bruker AVANCE AV 400 instruments and all NMR experiments were reported in units, parts per million (ppm), using residual solvent peaks ($CDCl_3$ (δ = 7.26 ppm) or TMS (δ = 0.00 ppm) or DMSO-d_6 (δ = 2.50 ppm) or CD_3OD (δ = 3.31 ppm) or CD_3CN (δ = 1.94 ppm) for ^{1}H NMR and $CDCl_3$ (δ = 77.16 ppm) or DMSO-d_6 (δ = 39.50 ppm) or CD_3OD (δ = 49.00 ppm) or CD_3CN (δ = 1.32 and 118.31 ppm) for ^{13}C NMR as internal reference. Multiplicities are recorded as: s = singlet, d = doublet, t = triplet, dd = doublet of doublets, m = multiplet. High resolution ESI mass experiments were operated on a Varian 7.0T FTMS instrument. UPLC-MS analyses were performed with a Dionex UltiMate 3000 connected to a Thermo scientific MSQ PLUS mass spectrometer using Thermo Scientific Acclaim^{TM} 120 C18 (5 μm, 4.6 × 250 mm) or Thermo

Scientific Hypersil GOLD C18 (1.9 μm, 2.1 × 100 mm) UPLC analytical column. Linear gradients using A: H_2O (0.1% HCOOH) and B: MeCN (0.1% HCOOH) were run over varying periods. Semi preparative HPLC was carried out on a Waters 1525 Binary HPLC Pump using a Waters Corp XBridge BEH C18 OBD Prep Column (5 μm, 10 × 150 mm) preparative column and a Waters 2489 UV/Visible Detector. Linear gradients using A: H_2O (0.1% HCOOH) and B: MeCN (0.1% HCOOH) were run over varying periods. The ee values were determined on Thermo Scientific Dionex UltiMate 3000 Standard Systems using Chiral column with hexane and 2-propanol as eluent, Wavelength = 220 or 254 nm.

NOTE: All of the sulfenamides were obtained as a mixture of two rotamers, so the NMR spectra show two sets of signals for each compound, including ^{1}H, ^{13}C and ^{19}F spectrum.

1.2 General procedures for the preparation of dioxazolone substrates

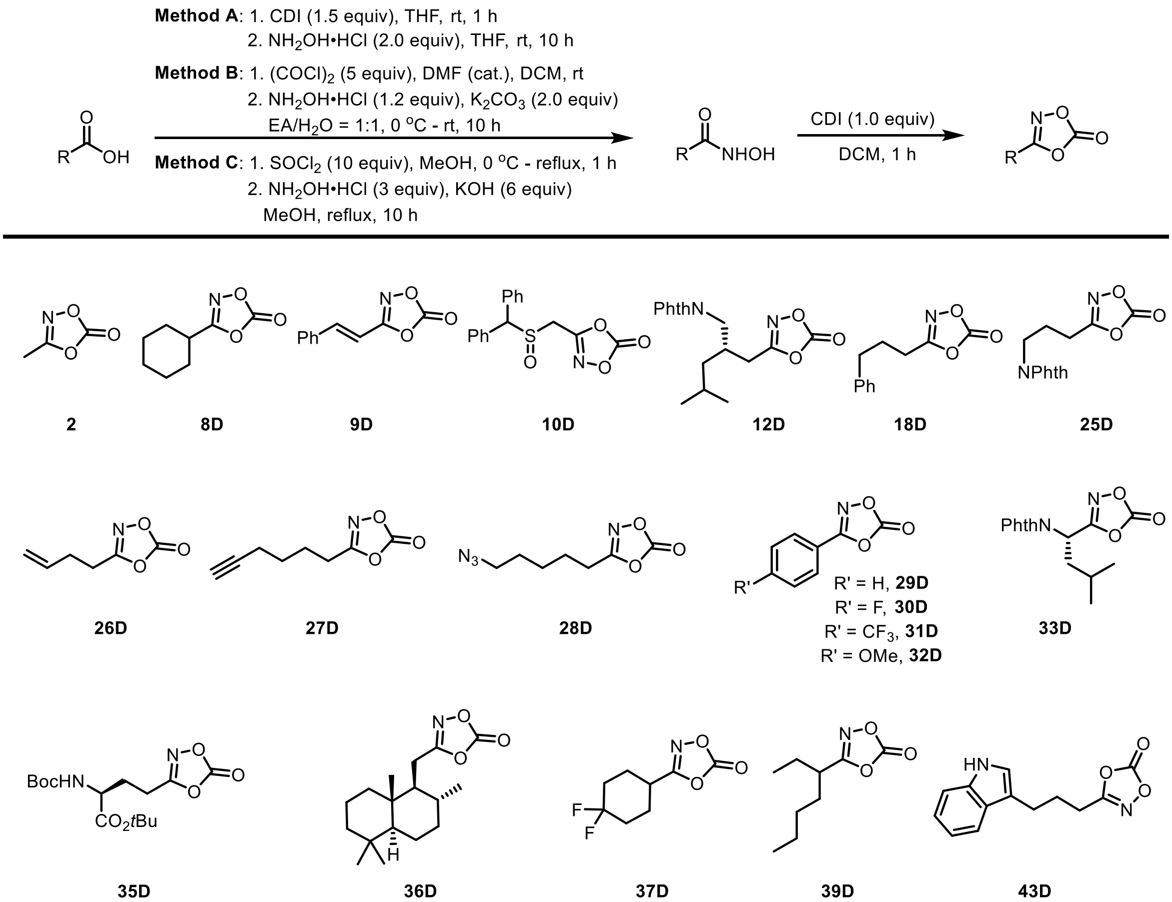

Supplementary Figure 1. The preparation of dioxazolone substrates

Step 1. Synthesis of hydroxamic acids.

Method A: 1,1'-Carbonyldiimidazole (CDI, 1.5 equiv.) was added to a mixture of carboxylic acid (1.0

equiv.) in dry tetrahydrofuran (THF, 1.0 M) at room temperature. The reaction mixture was stirred for 1-2 hours. Afterward, powdered hydroxylamine hydrochloride (2.0 equiv.) was added. The resulting mixture was stirred for 10 h. The reaction mixture was diluted with 5% aq. $KHSO_4$ and extracted with ethyl acetate. The combined organic layer was washed with water and brine, dried over anhydrous Na_2SO_4, and concentrated *in vacuo*. The resulting residue was purified by recrystallization from DCM/MeOH or silica gel flash chromatography (DCM/methanol = 30:1 ~ 10:1) to obtain the hydroxamic acid.

Method B: To a mixture of carboxylic acid (1.0 equiv.) in anhydrous DCM (0.2 M), DMF (2 drops) and $(COCl_2)_2$ (10.0 equiv.) were added dropwise at 0 ^oC. After the addition was completed, the mixture was heated to reflux for 1-2 h. After being cooled to room temperature, the solution was concentrated under reduced pressure to remove DCM and excess $(COCl_2)_2$. And the residue was dried under high vacuum for 1 h, the crude product was used for next step without further purification.

To a stirred solution of hydroxylamine hydrochloride (1.2 equiv.), and K_2CO_3 (2.0 equiv.) in EtOAc/H_2O (1:1, 0.5 M, v/v) was added the EtOAc solution of acyl chloride (1.2 equiv.) dropwise at 0 °C. The reaction mixture was stirred for 30 min. Afterward, the reaction mixture was warmed to room temperature under vigorously magnetically stirring for 10 h. The reaction mixture was then extracted with EtOAc. The combined organic phase was dried over anhydrous Na_2SO_4, filtered and concentrated *in vacuo*. The resulting residue was purified by recrystallization from DCM/MeOH or silica gel flash chromatography (DCM/methanol = 30:1 ~ 10:1) to obtain the hydroxamic acid.

Method C: To an ester, hydroxylamine hydrochloride (3.0 equiv) and KOH (6.0 equiv) were added to MeOH (0.2 M) under Air. The resultant mixture was stirred under reflux for 10 h. The reaction was then cooled to room temperature and acidified with 1 N HCl to pH 4. After removing the methanol from the solution under reduced pressure, the resultant liquid was extracted with EtOAc. The combined organic layer was dried with Na_2SO_4 and concentrated under reduced pressure. The residue was then purified by recrystallization from DCM/MeOH or silica gel flash chromatography (DCM/methanol = 30:1 ~ 10:1) to obtain the hydroxamic acid.

Step 2. Synthesis of dioxazolone substrates.

To a stirred solution of hydroxamic acid (1.0 equiv.) in freshly distilled dichloromethane, 1,1′-carbonyldiimidazole (1.0 equiv.) was added in one portion at room temperature. After being stirred for 1-2 hours, the reaction mixture was quenched with 1 N HCl, and extracted with EtOAc. The combined

organic phase was dried over anhydrous Na_2SO_4, filtered and concentrated *in vacuo*. The resulting residue was purified quickly by short silica pad (PE/EA = 10:1 ~ 5:1) or distillation under vacuum to give the desired dioxazolones.

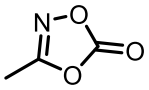

2

Compound **2** is a known compound, and was synthesized in 78% yield (2 steps, 100 mmol, 8.3 g) following the general procedure (**Method A**, Purified by distillation at reduced pressure). The spectra data are consistent with reference 75 in the main text.

Colorless oil. (R_f = 0.8, hexane/ethyl acetate = 5:1, v/v).

^{1}H NMR (400 MHz, $CDCl_3$) δ 2.33 (s, 3H).

^{13}C NMR (101 MHz, $CDCl_3$) δ 164.05, 154.24, 10.43.

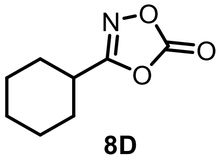

8D

Compound **8D** is a known compound, and was synthesized in 63% yield (2 steps, 10 mmol, 1.06 g) following the general procedure (**Method A**).The spectra data are consistent with reference 75 in the main text.

Colorless oil (R_f = 0.8, PE:EA = 5:1, v/v)

^{1}H NMR (400 MHz, $CDCl_3$) δ 2.68 (tt, J = 11.2, 3.8 Hz, 1H), 2.01 (d, J = 13.1 Hz, 2H), 1.91–1.78 (m, 2H), 1.78–1.67 (m, 1H), 1.60 – 1.45 (m, 2H), 1.46 – 1.22 (m, 3H).

^{13}C NMR (101 MHz, $CDCl_3$) δ 169.32, 154.36, 34.67, 28.17, 25.25, 24.86.

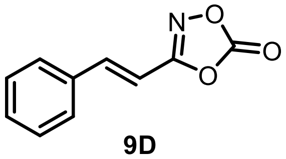

9D

Compound **9D** is a known compound, and was synthesized in 37% yield (2 steps, 20 mmol scale, 1.4 g) following the **Method A**. The spectra data are consistent with reference 75 in the main text.

White solid (R_f = 0.6, PE:EA = 5:1, v/v)

^{1}H NMR (400 MHz, $CDCl_3$) δ 7.58 – 7.52 (m, 2H), 7.50 – 7.41 (m, 4H), 6.65 (d, J = 16.4 Hz, 1H).

^{13}C NMR (101 MHz, CDCl$_3$) δ 163.48, 153.60, 143.21, 133.52, 131.35, 129.33, 128.17, 105.93.

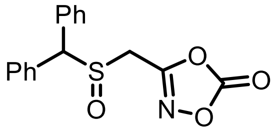

10D

Compound **10D** is an unknown compound, and was synthesized in 90% yield (2 steps, 10 mmol scale, 2.84 g) following the **Method A**.

White solid (R_f = 0.5, PE:EA = 2:1, v/v)

^{1}H NMR (400 MHz, CDCl$_3$) δ 7.55 – 7.34 (m, 10H), 5.16 (s, 1H), 3.87 (d, J = 14.3 Hz, 1H), 3.64 (d, J = 14.3 Hz, 1H).

^{13}C NMR (101 MHz, CDCl$_3$) δ 159.72, 153.15, 133.88, 133.29, 129.89, 129.51, 129.38, 129.20, 128.82, 73.27, 44.65.

HRMS (ESI) *m/z* Calcd for $C_{16}H_{13}NNaO_4S^+$ [M+Na]$^+$: 338.0457, found: 338.0460.

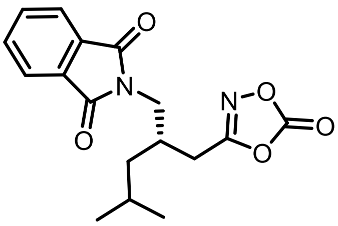

12D

Compound **12D** is a known compound, and was synthesized in 61% yield (2 steps, 30 mmol scale, 6 g) following the **Method B**. The spectra data are consistent with reference 74 in the main text.

Colorless oil (R_f = 0.5, PE:EA = 5:1, v/v)

^{1}H NMR (400 MHz, CDCl$_3$) δ 7.82 – 7.73 (m, 2H), 7.73 – 7.62 (m, 2H), 3.75 – 3.52 (m, 2H), 2.71 – 2.47 (m, 2H), 2.44 – 2.27 (m, 1H), 1.80 – 1.65 (m, 1H), 1.36 – 1.12 (m, 2H), 0.88 (dd, J = 15.8, 6.5 Hz, 6H).

^{13}C NMR (101 MHz, CDCl$_3$) δ 168.49, 165.56, 153.87, 134.26, 131.70, 123.39, 41.21, 41.18, 32.63, 28.20, 25.21, 22.63, 22.19.

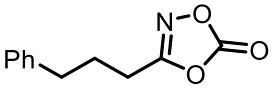

18D

Compound **18D** is a known compound, and was synthesized in 85% yield (2 steps, 10 mmol scale, 1.74 g) following the **Method A**. The spectra data are consistent with reference 68 in the main text.

Colorless oil (R_f = 0.7, PE:EA = 5:1, v/v)

^{1}H NMR (400 MHz, $CDCl_3$) δ 7.31 (t, *J* = 7.4 Hz, 2H), 7.26 – 7.20 (m, 1H), 7.20 – 7.15 (m, 2H), 2.74 (t, *J* = 7.4 Hz, 2H), 2.62 (t, *J* = 7.5 Hz, 2H), 2.07 (p, *J* = 7.4 Hz, 2H).

^{13}C NMR (101 MHz, $CDCl_3$) δ 166.57, 154.22, 139.92, 128.82, 128.59, 126.69, 34.69, 26.01, 24.16.

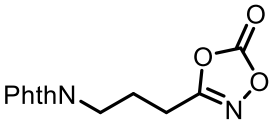

25D

Compound **25D** is a known compound, and was synthesized in 70% yield (2 steps, 20 mmol scale, 3.8 g) following the **Method B**. The spectra data are consistent with reference 74 in the main text.

White solid (R_f = 0.23, PE:EA = 5:1, v/v)

^{1}H NMR (400 MHz, $CDCl_3$) δ 7.90 – 7.81 (m, 2H), 7.80 – 7.71 (m, 2H), 3.84 (t, *J* = 6.6, 2H), 2.75 (t, *J* = 7.6, 2H), 2.27 – 2.00 (m, 2H).

^{13}C NMR (101 MHz, $CDCl_3$) δ 168.27, 165.84, 153.98, 134.28, 131.78, 123.42, 36.54, 23.47, 22.55.

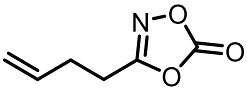

26D

Compound **26D** is a known compound, and was synthesized in 76% yield (2 steps, 10 mmol scale, 1.1 g) following the **Method A**. The spectra data are consistent with those reported in literature. [1]

Colorless oil (R_f = 0.73, PE:EA = 5:1, v/v)

^{1}H NMR (400 MHz, $CDCl_3$) δ 5.87 – 5.71 (m, 1H), 5.20 – 5.07 (m, 2H), 2.73 (t, *J* = 7.4 Hz, 2H), 2.53 – 2.41 (m, 2H).

^{13}C NMR (101 MHz, $CDCl_3$) δ 166.08, 154.11, 134.25, 117.53, 28.27, 24.29.

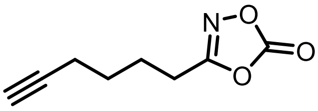

27D

Compound **27D** is a known compound, and was synthesized in 54% yield (2 steps, 10 mmol scale, 910

mg) following the **Method B**. The spectra data are consistent with reference 74 in the main text.

Colorless oil (R_f = 0.7, PE:EA = 5:1, v/v)

^{1}H NMR (400 MHz, $CDCl_3$) δ 2.66 (t, *J* = 7.5 Hz, 2H), 2.25 (td, *J* = 6.9, 2.7 Hz, 2H), 1.97 (t, *J* = 2.7 Hz, 1H), 1.90 – 1.80 (m, 2H), 1.69 – 1.56 (m, 2H).

^{13}C NMR (101 MHz, $CDCl_3$) δ 166.48, 154.19, 83.09, 69.36, 27.24, 24.37, 23.44, 17.88.

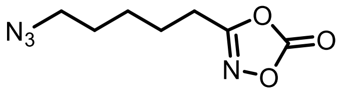

28D

Compound **28D** is an unknown compound, and was synthesized in 41% yield (2 steps, 6.75 mmol scale, 555 mg) following the **Method A**.

Colorless oil. (R_f = 0.5, hexane/ethyl acetate = 5:1, v/v)

^{1}H NMR (400 MHz, $CDCl_3$) δ 3.30 (t, *J* = 6.7 Hz, 2H), 2.65 (t, *J* = 7.5 Hz, 2H), 1.76 (p, *J* = 7.5 Hz, 2H), 1.68 – 1.58 (m, 2H), 1.55 – 1.43 (m, 2H).

^{13}C NMR (101 MHz, $CDCl_3$) δ 166.45, 154.19, 51.10, 28.39, 26.00, 24.76, 24.15.

HRMS (ESI) *m/z* Calcd for $C_7H_{11}N_4O_3^+$ $[M+H]^+$: 199.0826, found: 199.0825.

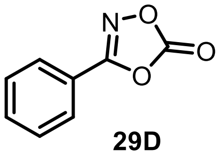

29D

Compound **29D** is a known compound, and was synthesized in 80% yield (2 steps, 20 mmol, 2.6 g) following the **Method B**. The spectra data are consistent with reference 75 in the main text.

White solid. (R_f = 0.8, hexane/ethyl acetate = 5:1, v/v)

^{1}H NMR (400 MHz, $CDCl_3$) δ 7.85 (d, *J* = 7.2 Hz, 2H), 7.65 (t, *J* = 7.7 Hz, 1H), 7.55 (t, *J* = 7.7 Hz, 2H).

^{13}C NMR (101 MHz, $CDCl_3$) δ 163.67, 153.98, 133.93, 129.53, 126.75, 120.24.

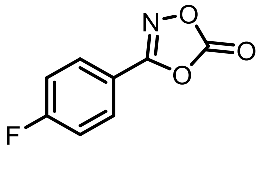

30D

Compound **30D** is a known compound, and was synthesized in 73% yield (2 steps, 20 mmol, 2.6 g) following the **Method B**. The spectra data are consistent with those reported in literature.[2]

White solid. (R_f = 0.83, hexane/ethyl acetate = 5:1, v/v)

^{1}H NMR (400 MHz, $CDCl_3$) δ 7.95 – 7.83 (m, 2H), 7.30 – 7.20 (m, 2H).

^{13}C NMR (101 MHz, $CDCl_3$) δ 167.29, 164.74, 162.90, 153.76, 129.36, 129.27, 117.21, 116.99, 116.53, 116.50.

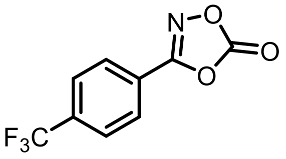

31D

Compound **31D** is a known compound, and was synthesized in 76% yield (2 steps, 20 mmol, 3.5 g) following the **Method B**. The spectra data are consistent with those reported in literature.[2]

White solid. (R_f = 0.83, hexane/ethyl acetate = 5:1, v/v)

^{1}H NMR (400 MHz, $CDCl_3$) δ 8.01 (d, *J* = 8.2 Hz, 2H), 7.83 (d, *J* = 8.2 Hz, 2H).

^{13}C NMR (101 MHz, $CDCl_3$) δ 162.66, 153.45, 135.99, 135.66, 135.33, 135.00, 127.27, 126.69, 126.65, 126.61, 126.57, 124.65, 123.67, 123.65, 121.93, 119.22.

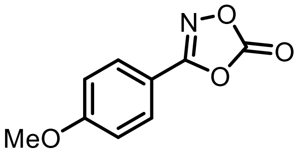

32D

Compound **32D** is a known compound, and was synthesized in 44% yield (2 steps, 20 mmol, 1.7 g) following the **Method B**. The spectra data are consistent with those reported in literature.[2]

White solid. (R_f = 0.63, hexane/ethyl acetate = 5:1, v/v)

^{1}H NMR (400 MHz, $CDCl_3$) δ 7.78 (d, *J* = 8.9 Hz, 2H), 7.02 (d, *J* = 8.9 Hz, 2H).

^{13}C NMR (101 MHz, $CDCl_3$) δ 163.99, 163.55, 154.19, 128.70, 115.01, 112.23, 55.75.

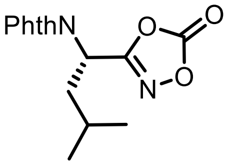

33D

Compound **33D** is an unknown compound, and was synthesized in 35% yield (2 steps, 20 mmol scale, 2.1 g) following the **Method A**.

White solid (R_f = 0.5, PE:EA = 5:1, v/v)

^{1}H NMR (400 MHz, $CDCl_3$) δ 7.91 – 7.86 (m, 2H), 7.81 – 7.77 (m, 2H), 5.43 (dd, *J* = 10.7, 4.7 Hz, 1H), 2.49 – 2.31 (m, 1H), 2.02 – 1.91 (m, 1H), 1.68 – 1.49 (m, 1H), 1.01 – 0.93 (m, 6H).

^{13}C NMR (101 MHz, $CDCl_3$) δ 166.92, 164.49, 153.52, 134.91, 131.34, 124.10, 43.79, 36.45, 24.62, 22.93, 21.29.

HRMS (ESI) *m/z* Calcd for $C_{15}H_{15}N_2O_5^+$ $[M+H]^+$: 303.0975, found: 303.0975.

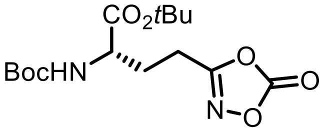

35D

Compound **35D** is an unknown compound, and was synthesized in 63% yield (2 steps, 10 mmol scale, 2.2 g) following the **Method A**.

White solid (R_f = 0.8, PE:EA = 5:1, v/v)

^{1}H NMR (400 MHz, $CDCl_3$) δ 5.22 (d, *J* = 7.8 Hz, 1H), 4.35 – 4.09 (m, 1H), 2.80 – 2.55 (m, 2H), 2.33 – 2.12 (m, 1H), 2.04 – 1.85 (m, 1H), 1.41 (d, *J* = 16.0 Hz, 18H).

^{13}C NMR (101 MHz, $CDCl_3$) δ 170.42, 166.05, 155.43, 154.01, 82.96, 80.24, 52.99, 28.29, 28.22, 27.98, 27.92, 27.82, 21.38.

HRMS (ESI) *m/z* Calcd for $C_{14}H_{24}N_2NaO_5^+$ $[M-CO_2+Na]^+$: 323.1577, found: 323.1582.

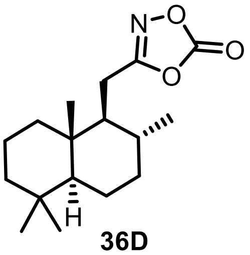

36D

Compound **36D** is a known compound, and was synthesized in 61% yield following the reported literature.[3]

Colorless oil. (R_f = 0.81, hexane/ethyl acetate = 5:1, v/v)

^{1}H NMR (400 MHz, $CDCl_3$) δ 2.70 (dd, *J* = 17.0, 3.3 Hz, 1H), 2.34 (dd, *J* = 17.0, 6.1 Hz, 1H), 1.82 – 1.75 (m, 1H), 1.68 – 1.46 (m, 5H), 1.43 – 1.36 (m, 1H), 1.32 – 1.20 (m, 2H), 1.20 – 1.11 (m, 1H), 1.11 – 0.97 (m, 2H), 0.96 – 0.91 (m, 1H), 0.90 – 0.85 (m, 6H), 0.84 – 0.79 (m, 6H).

^{13}C NMR (101 MHz, $CDCl_3$) δ 168.42, 154.42, 54.90, 53.65, 41.83, 39.09, 38.24, 36.51, 34.12, 33.52, 33.48, 33.39, 24.25, 21.82, 21.67, 20.79, 18.68, 14.09.

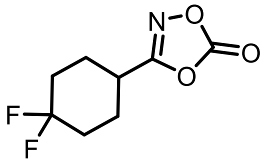

37D

Compound **37D** is a known compound, and was synthesized in 62% yield (2 steps, 20 mmol, 2.54 g) following the **Method A**. The spectra data are consistent with those reported in literature.[2]

White solid. (R_f = 0.54, hexane/ethyl acetate = 5:1, v/v)

^{1}H NMR (400 MHz, $CDCl_3$) δ 2.86 – 2.73 (m, 1H), 2.25 – 2.06 (m, 4H), 1.98 – 1.80 (m, 4H).

^{13}C NMR (101 MHz, $CDCl_3$) δ 167.76, 153.95, 124.17, 121.77, 119.36, 32.57, 32.47, 32.22, 31.97, 24.56, 24.53, 24.48, 24.45.

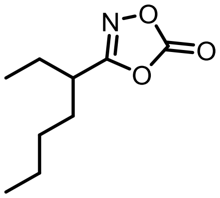

39D

Compound **39D** is an unknown compound, and was synthesized in 76% yield (2 steps, 20 mmol, 2.8 g) following the **Method A**.

Colorless oil. (R_f = 0.9, hexane/ethyl acetate = 5:1, v/v)

^{1}H NMR (400 MHz, $CDCl_3$) δ 2.65 – 2.55 (m, 1H), 1.75 – 1.59 (m, 4H), 1.35 – 1.23 (m, 4H), 0.94 (t, *J* = 7.5 Hz, 3H), 0.89 (t, *J* = 7.0 Hz, 3H).

^{13}C NMR (101 MHz, $CDCl_3$) δ 168.93, 154.45, 38.59, 30.58, 29.10, 24.49, 22.40, 13.86, 11.41.

HRMS (ESI) *m/z* Calcd for $C_9H_{16}NO_3^+$ $[M+H]^+$: 186.1125, found: 186.1126.

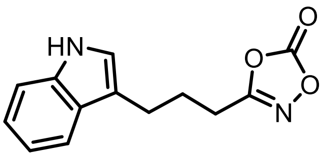

43D

Compound **43D** is a known compound, and was synthesized in 12% yield (2 steps, 20 mmol scale, 586 mg) following the **Method C**. The spectra data are consistent with reference 74 in the main text.

White solid. (R_f = 0.8, hexane/ethyl acetate = 5:1, v/v)

^{1}H NMR (400 MHz, $CDCl_3$) δ 7.97 (brs, 1H), 7.56 (d, *J* = 7.9 Hz, 1H), 7.35 (d, *J* = 8.1 Hz, 1H), 7.25 – 7.17 (m, 1H), 7.17 – 7.09 (m, 1H), 6.98 (s, 1H), 2.88 (t, *J* = 7.1 Hz, 2H), 2.69 – 2.54 (m, 2H), 2.12 (p, *J* = 7.3 Hz, 2H).

^{13}C NMR (101 MHz, $CDCl_3$) δ 166.70, 154.22, 136.42, 127.07, 122.27, 121.89, 119.52, 118.63, 114.02, 111.31, 24.74, 24.14, 24.07.

1.3 General procedures for the preparation of mercapto substrates

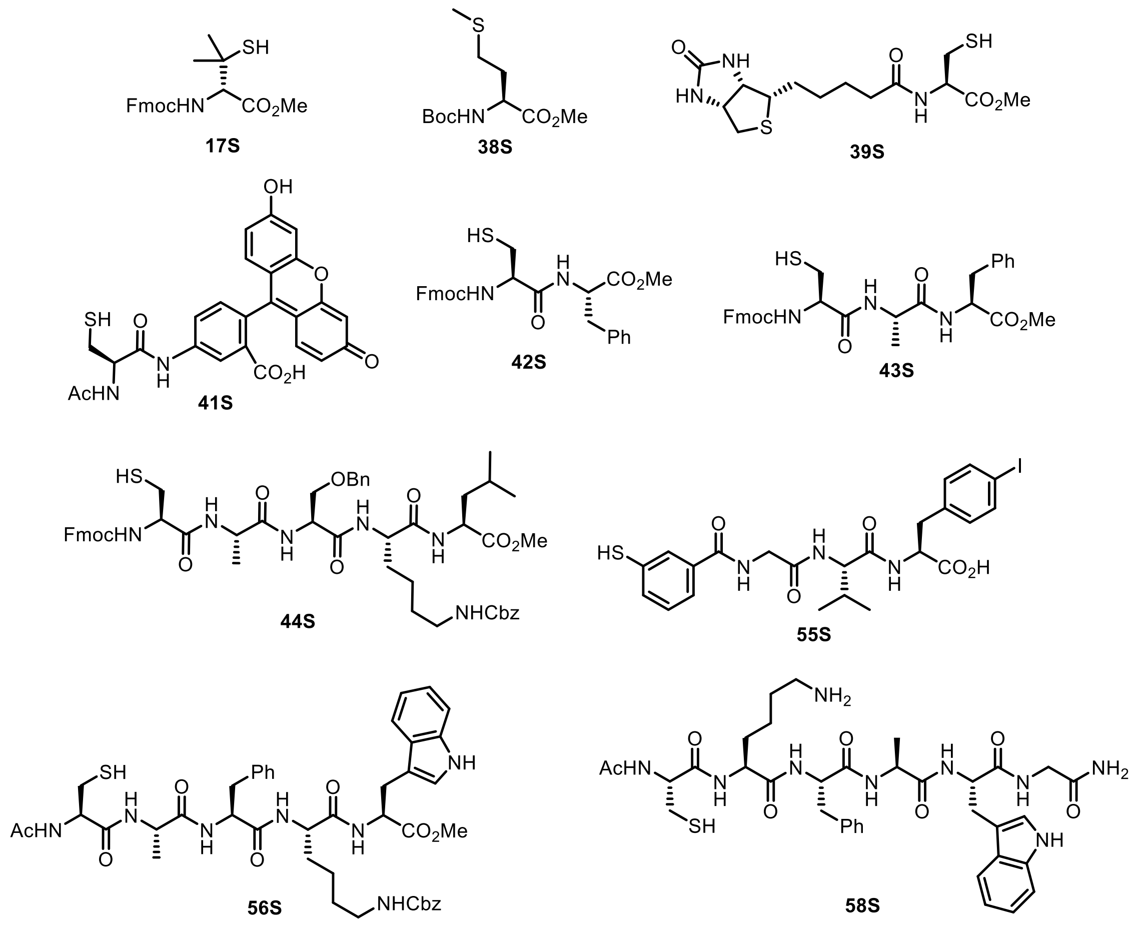

Supplementary Figure 2. Summary of mercapto substrates

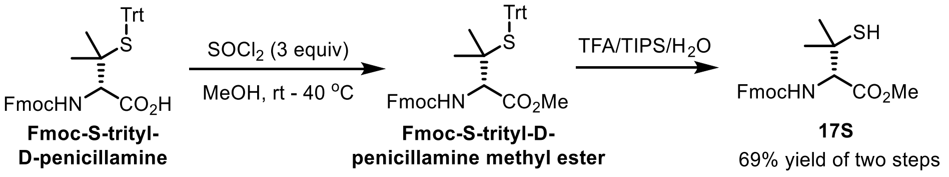

Supplementary Figure 3. The preparation of substrate **17S**

The substrate **17S** was prepared by the following procedure: To the solution of **Fmoc-S-trityl-D-penicillamine** (1 mmol, 614 mg) in MeOH (30 mL) was added dropwise $SOCl_2$ (3 mmol) at room temperature (rt). After the addition was completed, the mixture was heated to 40 °C for 2 h. After being cooled to room temperature, the solution was concentrated under reduced pressure to remove solvent and excess $SOCl_2$. The residue was dried under high vacuum for 1 h and gave the crude product **Fmoc-S-trityl-D-penicillamine methyl ester**. The methyl ester was add to TFA/TIPS/H_2O = 95:2.5:2.5 (v/v/v, 20 mL) and stirred for 2 h at rt. The solution was concentrated under reduced pressure. The

residue was washed with saturated $NaHCO_3$, and dried with Na_2SO_4. The crude product was purified by silica gel flash chromatography using petroleum ethers/ethyl acetate (20:1 to 5:1) to obtain the substrate **17S** in 69% yield of two steps.

Colorless oil (R_f = 0.35, PE:EA = 5:1, v/v)

^{1}H NMR (400 MHz, $CDCl_3$) δ 7.82 – 7.73 (m, 2H), 7.66 – 7.56 (m, 2H), 7.45 – 7.37 (m, 2H), 7.36 – 7.29 (m, 2H), 5.76 (d, J = 9.8 Hz, 1H), 4.52 – 4.34 (m, 3H), 4.24 (t, J = 7.0 Hz, 1H), 3.77 (s, 3H), 1.98 (s, 1H), 1.50 (s, 3H), 1.39 (s, 3H).

^{13}C NMR (101 MHz, $CDCl_3$) δ 171.09, 156.18, 143.92, 143.81, 141.45, 127.88, 127.22, 125.19, 120.14, 67.38, 62.73, 52.36, 47.29, 46.63, 30.96, 29.50.

HRMS (ESI) *m/z* Calcd for $C_{21}H_{23}NNaO_4S^+$ $[M+Na]^+$: 408.1240, found: 408.1239.

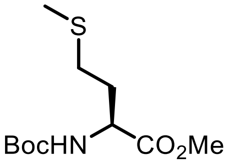

38S

Compound **38S** is a known compound, and was synthesized in 90% yield (10 mmol, 2.37 g) following the reported literature.[4]

Colorless oil (R_f = 0.33, PE:EA = 5:1, v/v)

^{1}H NMR (400 MHz, $CDCl_3$) δ 5.13 (d, J = 8.4 Hz, 1H), 4.40 (q, J = 7.3 Hz, 1H), 3.74 (s, 3H), 2.58 – 2.45 (m, 2H), 2.18 – 2.03 (m, 4H), 1.98 – 1.83 (m, 1H), 1.43 (s, 9H).

^{13}C NMR (101 MHz, $CDCl_3$) δ 172.93, 155.44, 80.16, 52.86, 52.54, 32.33, 30.09, 28.42, 15.61.

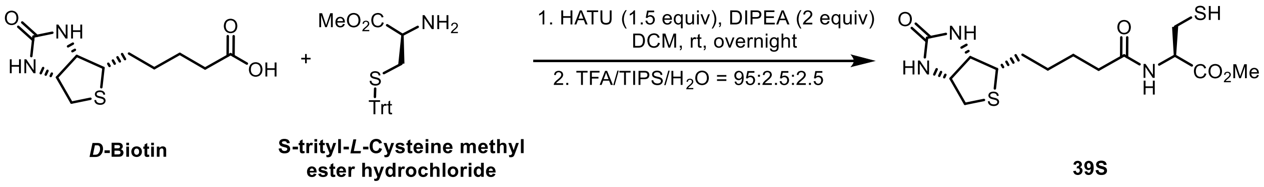

Supplementary Figure 4. The preparation of substrate **39S**

The substrate **39S** was prepared by the following procedure: To a solution of **D-Biotin** (10 mmol, 2.4 g) in DCM (100 mL) was added DIPEA (20 mmol), HATU (15 mmol) and S-trityl-L-Cysteine methyl ester hydrochloride (10 mmol) sequentially and was stirred overnight at rt. The reaction was quenched with HCl (1N, 50 mL) and extracted with DCM three times. The combined organic phase was dried

over anhydrous Na_2SO_4, filtered and concentrated *in vacuo*. The resulting residue was purified by silica gel flash chromatography to obtain the substrate **Trt-39S**. **Trt-39S** was dissolved in TFA/TIPS/H_2O = 95:2.5:2.5 (v/v/v, 20 mL) and stirred for 2 h at rt. The solution was concentrated under reduced pressure. The residue was washed with saturated $NaHCO_3$, and dried with Na_2SO_4. The crude product was purified by silica gel flash chromatography using DCM/MeOH (100:1 to 15:1) to give the substrate **39S** in 18% yield (660 mg) of two steps.

White solid (R_f = 0.43, DCM:MeOH = 10:1, v/v)

^{1}H NMR (400 MHz, $CDCl_3$) δ 7.17 (d, *J* = 7.8 Hz, 1H), 6.55 (brs, 1H), 5.64 (brs, 1H), 4.90 – 4.83 (m, 1H), 4.55 – 4.47 (m, 1H), 4.36 – 4.28 (m, 1H), 3.78 (s, 3H), 3.19 – 3.10 (m, 1H), 2.98 (dd, *J* = 8.9, 4.7 Hz, 2H), 2.94 – 2.87 (m, 1H), 2.74 (d, *J* = 12.8 Hz, 1H), 2.35 – 2.26 (m, 2H), 1.78 – 1.64 (m, 4H), 1.56 – 1.41 (m, 3H).

^{13}C NMR (101 MHz, $CDCl_3$) δ 173.59, 171.59, 164.64, 62.00, 60.40, 56.10, 53.94, 52.85, 40.65, 35.78, 28.53, 28.10, 26.84, 25.71.

HRMS (ESI) *m/z* Calcd for $C_{14}H_{23}N_3NaO_4S_2^+$ $[M+Na]^+$: 384.1022, found: 384.1022.

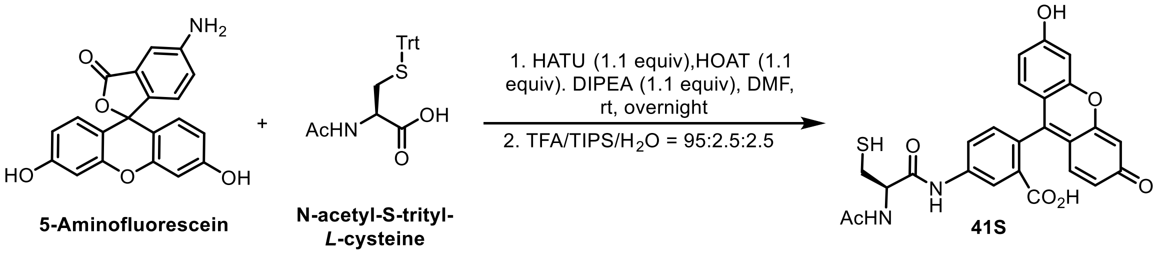

Supplementary Figure 5. The preparation of substrate **41S**

The substrate **41S** was prepared by the following procedure: To a solution of HATU (1.7 mmol, 640 mg), HOAT (1.7 mmol, 229 mg), DIPEA (1.7 mmol, 0.3 mL) and **N-acetyl-S-trityl-L-cysteine** (1.8 mmol, 745 mg) in DMF (10 mL) was added **5-aminofluorescein** (1.53 mmol, 532 mg) and stirred overnight at room temperature. DMF was removed *in vacuo* and the crude product was purified by silica gel flash chromatography to give the substrate **Trt-41S**. **Trt-41S** was dissolved in TFA/TIPS/H_2O = 95:2.5:2.5 (v/v/v, 15 mL) and stirred for 2 h at rt. The solution was concentrated under reduced pressure. The residue was dispersed in Et_2O (30 mL) and filtrated. The filter was collected and washed with Et_2O (5 mL) three times and gave the substrate **41S** in 93% yield (701 mg) of two steps.

Yellow solid (R_f = 0.3, DCM:MeOH = 15:1, v/v)

^{1}H NMR (400 MHz, Methanol-d_4) δ 8.41 (d, J = 2.0 Hz, 1H), 7.92 (dd, J = 8.4, 2.0 Hz, 1H), 7.20 (d, J = 8.3 Hz, 1H), 6.85 – 6.69 (m, 4H), 6.64 (dd, J = 8.8, 2.4 Hz, 2H), 4.65 (t, J = 6.6 Hz, 1H), 3.09 – 2.80 (m, 2H), 2.06 (s, 3H).

^{13}C NMR (101 MHz, Methanol-d_4) δ 173.57, 171.26, 170.59, 155.23, 141.49, 130.84, 129.70, 127.77, 126.79, 117.65, 114.85, 112.57, 103.48, 58.06, 26.71, 22.44, 18.19.

HRMS (ESI) *m/z* Calcd for $C_{25}H_{21}N_2O_7S^+$ $[M+H]^+$: 493.1064, found: 493.1064.

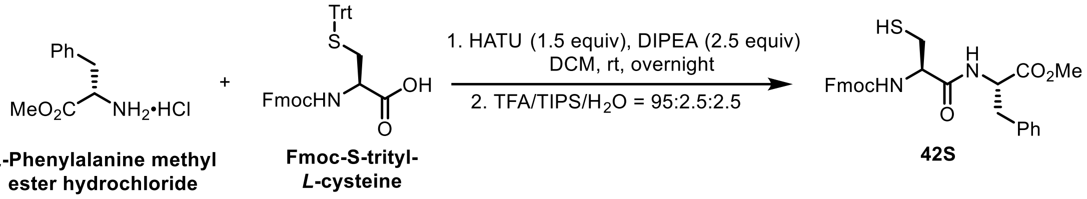

Supplementary Figure 6. The preparation of substrate **42S**

The synthesis method of compound **42S** was similar to substrate **40S** and the DIPEA (2.5 equiv) was used (5 mmol scale, 85% yield, 2.14 g).

White solid (R_f = 0.27, PE:EA = 2:1, v/v)

^{1}H NMR (400 MHz, DMSO-d_6) δ 8.53 (d, J = 8.1 Hz, 1H), 7.89 (d, J = 7.5 Hz, 2H), 7.75 (d, J = 7.5 Hz, 2H), 7.53 (d, J = 8.5 Hz, 1H), 7.42 (t, J = 7.5, 2H), 7.33 (t, J = 7.5, 2H), 7.29 – 7.15 (m, 5H), 4.62 – 4.50 (m, 1H), 4.36 – 4.11 (m, 4H), 3.63 (s, 3H), 3.08 (dd, J = 13.7, 5.1 Hz, 1H), 2.90 (dd, J = 13.7, 9.6 Hz, 1H), 2.63 – 2.54 (m, 1H), 2.50 – 2.43 (m, 1H), 2.05 (t, J = 8.5 Hz, 1H).

^{13}C NMR (101 MHz, DMSO-d_6) δ 171.78, 169.99, 155.95, 143.84, 143.80, 140.74, 137.01, 129.20, 128.22, 127.68, 127.10, 126.60, 125.38, 120.13, 65.84, 57.00, 53.39, 51.99, 46.63, 36.87, 26.40.

HRMS (ESI) *m/z* Calcd for $C_{28}H_{28}N_2NaO_5S^+$ $[M+Na]^+$: 527.1611, found: 527.1615.

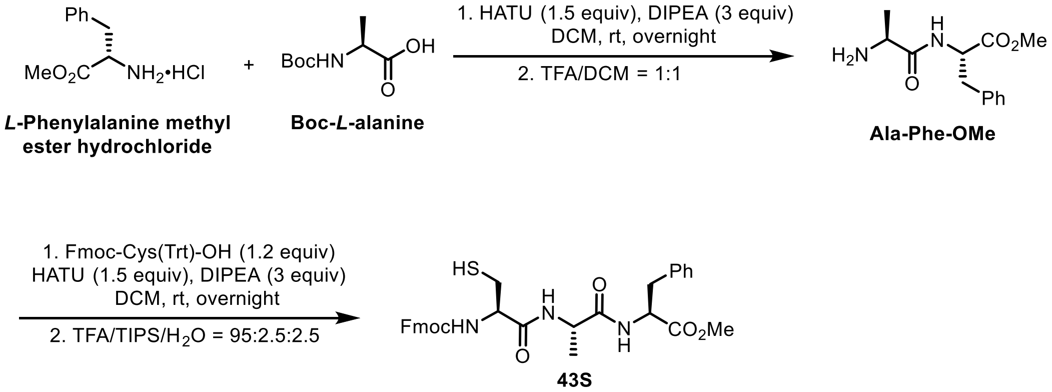

Supplementary Figure 7. The preparation of substrate **43S**

The substrate **43S** was prepared by the following procedure: To a solution of HATU (15 mmol, 5.7 g), **L-Phenylalanine methyl ester hydrochloride** (10 mmol, 2.16 g) and DIPEA (30 mmol, 3.87 g) in DCM (100 mL) was added **Boc-L-alanine** (10 mmol, 1.89 g) and stirred overnight at room temperature. 1 N HCl (100 mL) was added to the mixture and extracted with DCM. The combined organic phase was dried over anhydrous Na_2SO_4, filtered and concentrated *in vacuo*. The crude product was dissolved in TFA/DCM = 1:1 (20 mL) and stirred for 1 h. The reaction mixture was concentrated *in vacuo* and neutralized with saturated $NaHCO_3$. The aqueous phase was extracted with DCM. The combined organic phase was dried over anhydrous Na_2SO_4, filtered and concentrated *in vacuo* and gave the **Ala-Phe-OMe** without further purification.

To a solution of **Ala-Phe-OMe** (10 mmol) in DCM (100 mL) was added DIPEA (30 mmol), HATU (15 mmol) and Fmoc-Cys(Trt)-OH (10 mmol) sequently and was stirred overnight at rt. The reaction was quenched with HCl (1N, 50 mL) and extracted with DCM three times. The combined organic phase was dried over anhydrous Na_2SO_4, filtered and concentrated *in vacuo*. The resulting residue was purified by silica gel flash chromatography to obtain the substrate **Trt-43S**. **Trt-43S** was dissolved in TFA/TIPS/H_2O = 95:2.5:2.5 (v/v/v, 40 mL) and stirred for 2 h at rt. The solution was concentrated under reduced pressure. The residue was washed with saturated $NaHCO_3$, and dried with Na_2SO_4. The crude product was purified by silica gel flash chromatography using DCM/MeOH (100:1 to 30:1) to give the substrate **43S** in 52% overall yield (3 g).

White solid (R_f = 0.42, DCM:MeOH = 20:1, v/v)

^{1}H NMR (400 MHz, DMSO-d_6) δ 8.35 – 8.25 (m, 1H), 8.11 (d, *J* = 7.4 Hz, 1H), 7.89 (d, *J* = 7.5 Hz, 2H), 7.73 (dd, *J* = 7.5, 4.7 Hz, 2H), 7.57 (d, *J* = 8.2 Hz, 1H), 7.42 (t, *J* = 7.4 Hz, 2H), 7.36 – 7.16 (m, 7H), 4.50 – 4.41 (m, 1H), 4.36 – 4.18 (m, 4H), 4.16 – 4.08 (m, 1H), 3.57 (s, 3H), 3.06 – 2.88 (m, 2H), 2.81 – 2.70 (m, 1H), 2.70 – 2.56 (m, 1H), 2.30 (t, *J* = 8.4 Hz, 1H), 1.19 (d, *J* = 7.1 Hz, 3H).

^{13}C NMR (101 MHz, DMSO-d_6) δ 172.19, 171.70, 169.61, 155.94, 143.80, 143.74, 140.69, 136.95, 129.04, 128.22, 127.62, 127.06, 126.54, 125.29, 120.09, 65.72, 57.03, 53.57, 51.79, 48.00, 46.60, 36.55, 26.32, 18.07.

HRMS (ESI) *m/z* Calcd for $C_{31}H_{33}N_3NaO_6S^+$ $[M+Na]^+$: 598.1982, found: 598.1986.

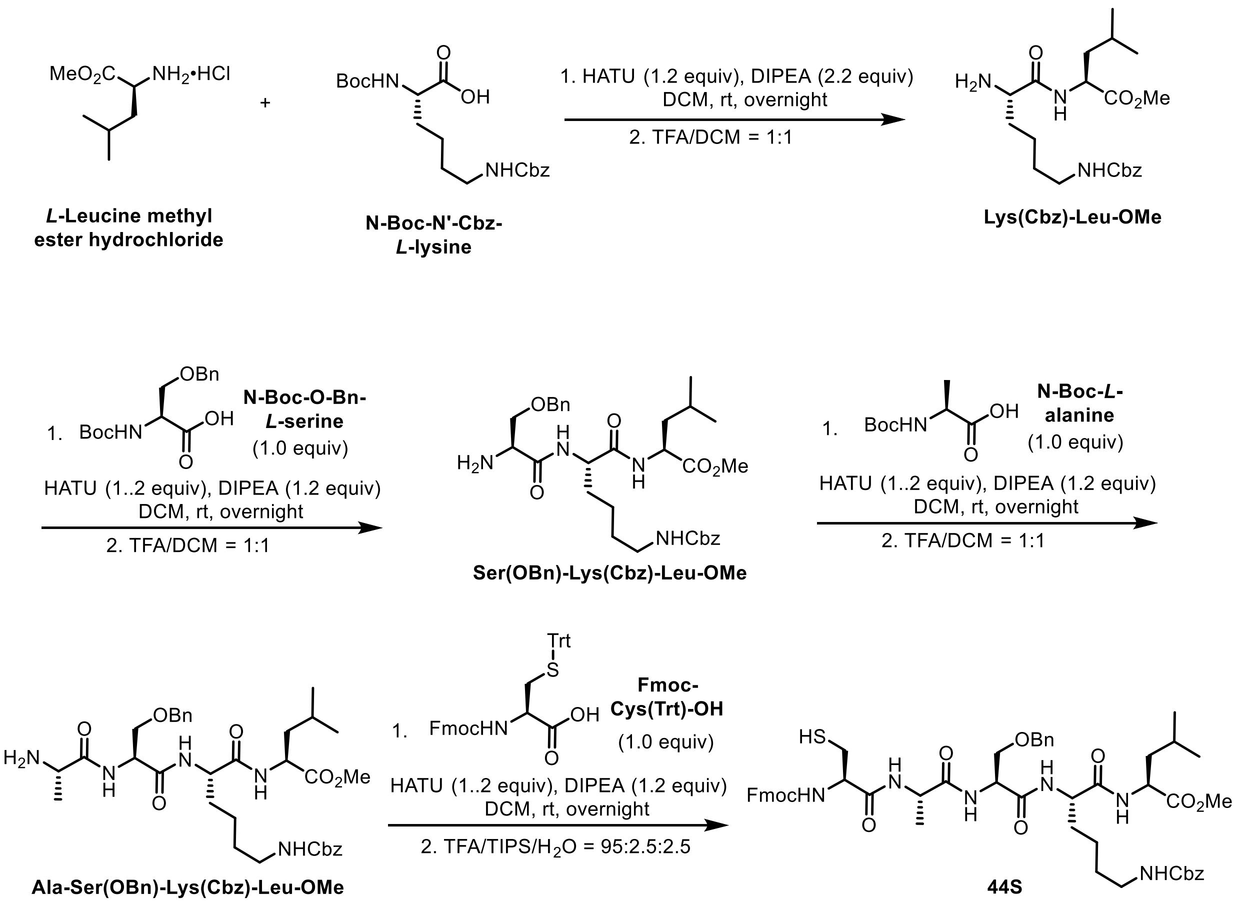

Supplementary Figure 8. The preparation of substrate **44S**

The synthesis methods of **44S** were similar with **43S**: Repeating the coupling and deprotection steps, peptide chain can be elongated with Leu, Lys, Ser, Ala and Cys successively and give the peptide **44S** (5 mmol scale, 736 mg, 15% overall yield).

White solid (R_f = 0.2, DCM:MeOH = 20:1, v/v)

^{1}H NMR (400 MHz, DMSO-d_6) δ 8.26 – 8.16 (m, 2H), 8.13 – 8.05 (m, 1H), 7.94 – 7.85 (m, 3H), 7.73 (t, *J* = 6.2 Hz, 2H), 7.58 (d, *J* = 8.2 Hz, 1H), 7.41 (t, *J* = 7.5 Hz, 2H), 7.37 – 7.23 (m, 12H), 7.20 (t, *J* = 5.6 Hz, 1H), 5.00 (s, 2H), 4.57 – 4.49 (m, 1H), 4.47 (s, 2H), 4.38 – 4.11 (m, 7H), 3.63 – 3.54 (m, 5H), 2.95 (q, *J* = 6.9 Hz, 2H), 2.85 – 2.74 (m, 1H), 2.72 – 2.60 (m, 1H), 2.32 (t, *J* = 8.5 Hz, 1H), 1.69 – 1.34 (m, 7H), 1.31 – 1.19 (m, 5H), 0.85 (d, *J* = 6.4 Hz, 3H), 0.79 (d, *J* = 6.4 Hz, 3H).

^{13}C NMR (101 MHz, DMSO-d_6) δ 172.68, 172.16, 171.35, 169.78, 168.93, 156.02, 155.92, 143.79, 143.73, 140.68, 138.05, 137.23, 128.30, 128.13, 127.70, 127.62, 127.41, 127.34, 127.05, 125.27, 120.08, 72.09, 69.77, 65.73, 65.09, 57.06, 52.70, 52.11, 51.75, 50.18, 48.29, 46.60, 31.96, 29.15, 26.26, 24.12, 22.66, 22.26, 21.19, 18.03.

HRMS (ESI) *m/z* Calcd for $C_{52}H_{64}N_6NaO_{11}S^+$ $[M+Na]^+$: 1003.4246, found: 1003.4252.

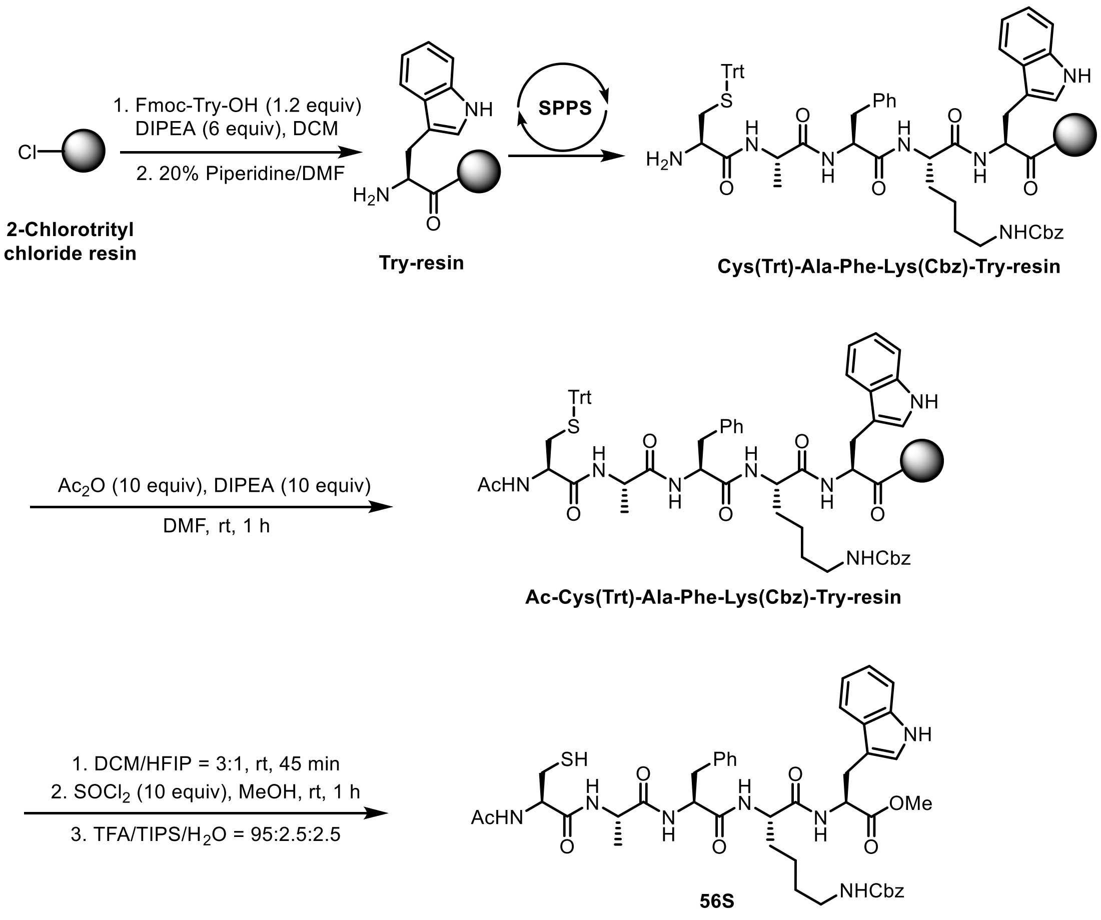

Supplementary Figure 9. The preparation of substrate **56S**

Peptide **56S** obtained in this work were synthesized following the reported **SPPS procedures**[5]:

2-Chlorotrityl chloride resin (1 g, 0.99 mmol, 1.0 equiv) was swelled in 1% DIPEA/DCM for 10 min before added into a 25 mL peptide synthesis tube. After sucking the solvent under vacuum, a solution of the Fmoc-Try-OH (511.8 mg, 1.2 mmol, 1.2 equiv) and DIPEA (1.1 mL, 6.0 mmol, 6.0 equiv) in DCM (20 mL) was added. The tube was capped and shaken for 1 h at room temperature. The tube was then drained, rinsed with CH_2Cl_2 (15 mL). The resin was treated with 20% piperidine/DMF (15 mL) for 10 minutes followed by thorough washing with DMF (10 mL) and DCM (10 mL), which was performed twice. A solution of **N-Fmoc-N'-Cbz-*L*-lysine** (1.5 g, 3.0 mmol, 3.0 equiv) and Oxyma (0.43 g, 1.8 mmol, 3 equiv) in NMP (15 mL) followed by DIC (0.51 mL, 3.3 mmol, 3.3 equiv) were added to the resin and the mixture was shaken for 1 h at room temperature. The resin was then drained and rinsed with DMF (2 x 10 mL) and DCM (2 x 10 mL). Repeating the coupling and deprotection steps, peptide chain can be elongated with Phe, Ala and Cys successively and give **Cys(Trt)-Ala-Phe-Lys(Cbz)-Try-resin**. To a solution of **Cys(Trt)-Ala-Phe-Lys(Cbz)-Try-resin** in DMF (20 mL) was added Ac_2O (0.94 mL, 10 mmol, 10 equiv) and DIPEA (2.74 mL, 10 mmol, 10 equiv) in peptide

synthesis tube, and the mixture was shaken for 1 h at room temperature. The tube was then drained, rinsed with CH_2Cl_2 (15 mL) to give **Ac-Cys(Trt)-Ala-Phe-Lys(Cbz)-Try-resin**. After the completion of peptide elongation, the resin was treated with a solution of DCM/HFIP (20 mL, 3/1, v/v) twice for 1 h each time. The combined solvent was concentrated *in vacuo* to give the peptide with a free carboxylic acid group which was treated with the $SOCl_2$ (0.73 mL, 10 mmol, 10 equiv) in MeOH (25 mL) at 0 ^oC. The solution was gradually warmed to room temperature and stirred for 1 hours. After removing the solvent under vacuum, and the mixture was added the cocktails of TFA/ H_2O/TIPS (95:2.5:2.5) for 1 hour. Then the solvents were evaporated at 30 ^oC and the crude peptide was precipitated by the addition of cold diethyl ether. The crude product was obtained after filtration. The crude product was further purified by silica gel flash chromatography using DCM/MeOH (100:1 to 15:1) to gave **56S** (350 mg, 41% yield).

White solid (R_f = 0.3, DCM:MeOH = 10:1, v/v)

^{1}H NMR (400 MHz, DMSO-d_6) δ 10.87 (s, 1H), 8.35 (d, J = 7.2 Hz, 1H), 8.14 (d, J = 7.2 Hz, 1H), 8.08 (d, J = 7.9 Hz, 1H), 7.93 (d, J = 8.0 Hz, 1H), 7.87 (d, J = 8.2 Hz, 1H), 7.48 (d, J = 7.8 Hz, 1H), 7.39 – 7.27 (m, 6H), 7.24 (t, J = 5.4 Hz, 1H), 7.21 – 7.10 (m, 6H), 7.06 (t, J = 7.5 Hz, 1H), 6.98 (t, J = 7.4 Hz, 1H), 5.00 (s, 2H), 4.55 – 4.44 (m, 2H), 4.39 – 4.24 (m, 2H), 4.20 (p, J = 7.0 Hz, 1H), 3.53 (s, 3H), 3.19 – 3.03 (m, 2H), 3.02 – 2.91 (m, 3H), 2.82 – 2.65 (m, 2H), 2.64 – 2.54 (m, 1H), 2.29 (t, J = 8.5 Hz, 1H), 1.86 (s, 3H), 1.68 – 1.56 (m, 1H), 1.56 – 1.44 (m, 1H), 1.38 (q, J = 7.3 Hz, 2H), 1.29 – 1.19 (m, 2H), 1.14 (d, J = 7.1 Hz, 3H).

^{13}C NMR (101 MHz, DMSO-d_6) δ 172.09, 171.85, 171.50, 170.46, 169.61, 169.46, 156.04, 137.56, 137.23, 136.05, 129.18, 128.33, 127.94, 127.73, 127.02, 126.15, 123.68, 120.95, 118.41, 117.93, 111.41, 109.17, 65.11, 54.89, 53.55, 53.10, 52.14, 51.74, 48.38, 37.23, 32.01, 29.23, 26.97, 26.20, 22.48, 17.91.

HRMS (ESI) *m/z* Calcd for $C_{43}H_{53}N_7NaO_9S^+$ $[M+Na]^+$: 866.3518, found: 866.3517.

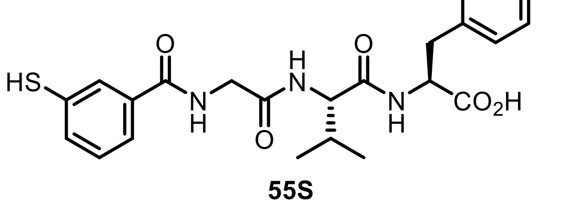

55S

The synthetic methods of peptide **55S** were similar to peptide **56S** (SPPS procedure, 1 mmol scale, 83% yield, 484 mg) without silica gel flash chromatography.

White solid (R_f = 0.1, DCM:MeOH = 2:1, v/v)

^{1}H NMR (400 MHz, Methanol-d_4) δ 7.78 (s, 1H), 7.64 – 7.54 (m, 2H), 7.45 (d, *J* = 7.7 Hz, 1H), 7.32 (t, *J* = 7.8 Hz, 1H), 7.00 (d, *J* = 7.8 Hz, 2H), 4.65 (dd, *J* = 8.7, 5.1 Hz, 1H), 4.23 (d, *J* = 7.0 Hz, 1H), 4.04 (s, 2H), 3.15 (dd, *J* = 13.9, 5.3 Hz, 1H), 2.94 (dd, *J* = 14.0, 8.7 Hz, 1H), 2.11 – 1.96 (m, 1H), 0.89 (dd, *J* = 18.4, 6.8 Hz, 6H).

^{13}C NMR (101 MHz, DMSO-d_6) δ 172.64, 171.10, 168.87, 165.88, 137.45, 137.06, 136.41, 135.31, 131.70, 130.06, 129.71, 126.67, 126.49, 92.36, 57.47, 53.31, 42.88, 36.26, 30.86, 19.28, 17.95.

HRMS (ESI) *m/z* Calcd for $C_{23}H_{26}IN_3NaO_5S^+$ $[M+Na]^+$: 606.0530, found: 606.0532.

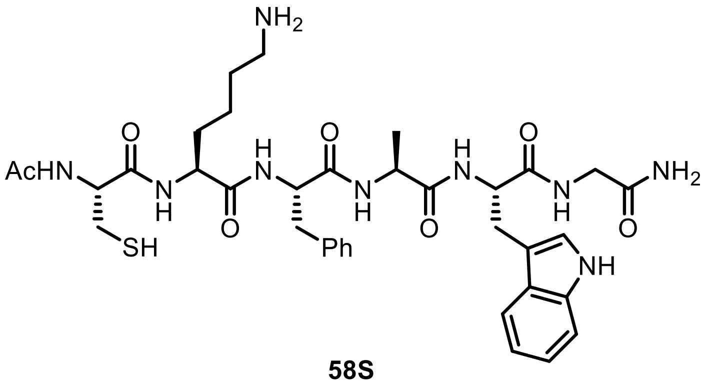

58S

Solid-phase peptide synthesis: Peptide **58S** were assembled on an Automated microwave peptide synthesizer (Liberty BlueTM, CEM) using Rink Amide MBHA resin (0.5 mmol, 1.0 equiv). Fmoc-protected AAs (5.0 equiv), DIC (5.0 equiv) and Oxyma (5.0 equiv) were used in each coupling cycle. Deprotection of Fmoc was conducted by treatment with 20% piperidine/DMF for each cycle. Acetylation: To a solution of resin-peptide in DMF (20 mL) was added Ac_2O (0.47 mL, 5 mmol, 10 equiv) and DIPEA (1.37 mL, 5 mmol, 10 equiv) in peptide synthesis tube, and the mixture was shaken for 1 h at room temperature.

Cleavage of linear peptides from resin: The dry Rink Amide MBHA was treated with a cocktail of TFA/TIPS/H_2O (95/2.5/2.5, v/v/v, 20 mL) for 2 hours. Then the solvents were evaporated at 30 ^oC and the crude peptide was precipitated by the addition of cold diethyl ether. The product was obtained after filtration to give **58S** in 55% yield (207 mg).

White solid

^{1}**H NMR** (400 MHz, Methanol-*d*$_4$) δ 8.52 (s, 1H), 7.56 (d, *J* = 7.7 Hz, 1H), 7.30 – 7.15 (m, 6H), 7.13 (s, 1H), 7.09 – 6.98 (m, 2H), 4.60 – 4.48 (m, 2H), 4.31 (t, *J* = 6.6 Hz, 1H), 4.27 – 4.18 (m, 2H), 3.84 (d, *J* = 17.1 Hz, 1H), 3.67 (d, *J* = 17.0 Hz, 1H), 3.36 – 3.31 (m, 1H), 3.26 – 3.18 (m, 1H), 3.02 (dd, *J* = 14.1, 5.0 Hz, 1H), 2.88 – 2.79 (m, 3H), 2.79 – 2.70 (m, 2H), 1.99 (s, 3H), 1.75 – 1.63 (m, 1H), 1.63 – 1.50 (m, 3H), 1.37 – 1.27 (m, 5H).

^{13}**C NMR** (101 MHz, Methanol-*d*$_4$) δ 174.87, 174.33, 174.27, 173.81, 173.70, 173.60, 172.87, 138.26, 138.04, 130.41, 129.51, 128.72, 127.85, 124.76, 122.58, 119.97, 119.30, 112.49, 110.74, 104.64, 56.22, 55.92, 54.79, 54.36, 50.95, 43.35, 41.07, 40.47, 38.52, 33.06, 32.15, 30.73, 30.45, 28.27, 27.94, 23.49, 22.59, 17.77.

HRMS (ESI) *m/z* Calcd for $C_{36}H_{48}N_9O_7S^-$ $[M-H]^-$: 750.3403, found: 750.3400.

1.4 General procedures for the S–N coupling

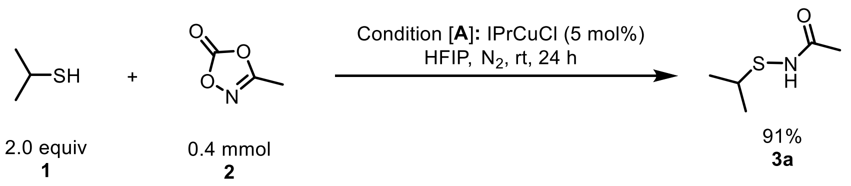

Supplementary Figure 10. General procedures for the the reaction of **1** with **2**

Condition [A]:

General procedure for the synthesis of **3a**: To a solution of isopropanethiol **1** (60.8 mg, 0.8 mmol, 2 equiv) and IPrCuCl (9.8 mg, 0.02 mmol, 5 mol%) in HFIP (1 mL, 0.4 M), 3-methyldioxazolone **2** (40.4 mg, 0.4 mmol, 1 equiv) was added. The reaction mixture was stirred under N_2 atomosphere for 24 hours at rt. The reaction mixture was concentrated *in vacuo*. The resulting residue was purified by silica gel flash chromatography using petroleum ether/ethyl acetate (10:1 to 2:1) eluent to give the desired product **3a** (48.4 mg, 91% yield) as brown oil.

NOTE: All sulfenamides were obtained as a mixture of rotamers, so the NMR spectra show two sets of signals for each compound, including ^{1}H, ^{13}C and ^{19}F spectrum. Sulfenamides are visualized at 220 or 254 nm of UV and can be stained on I_2/silica gel.

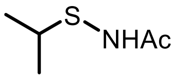

3a

Compound **3a** is a known compound[6], and the synthetic procedure can be scaled up to 15 mmol of **3a** in 80% yield (1.44 g) under **Condition [A]**.

Brown oil (R_f= 0.37, PE:EA = 2:1, v/v)

^{1}H NMR (400 MHz, $CDCl_3$) δ 6.65 and 6.34 (2×brs, 1H), 3.23 (hept, *J* = 6.7 Hz, 0.6H), 3.08 (hept, *J* = 6.6 Hz, 0.4H), 2.22 and 2.10 (2×s, 3H), 1.25 – 1.14 (m, 6H).

^{13}C NMR (101 MHz, $CDCl_3$) δ 178.85, 172.86, 42.01, 40.41, 23.41, 20.56, 20.28, 19.40.

HRMS (ESI) *m/z* Calcd for $C_5H_{11}NNaOS^+$ $[M+Na]^+$: 156.0454, found: 156.0454.

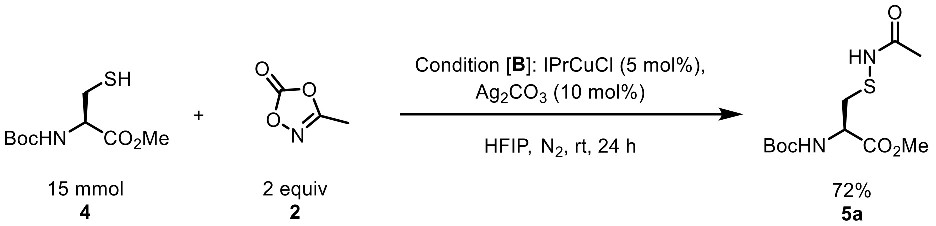

Supplementary Figure 11. General procedures for the the reaction of **4** with **2**

Condition [B]:

To a solution of Boc-protected cysteine methyl ester **4** (3.53 g, 15 mmol, 1.0 equiv), IPrCuCl (366 mg, 0.75 mmol, 5 mol%), and Ag_2CO_3 (414 mg, 1.5 mmol, 10 mol%) in HFIP (37.5 mL, 0.4 M), 3-methyldioxazolone **2** (3.03 g, 30 mmol, 2.0 equiv) was added. The reaction mixture was stirred under N_2 atomosphere for 24 hours at rt. The reaction mixture was then concentrated under reduced pressure, redissolved in DCM (50 mL), and washed with H_2O (50 mL). The organic layer was dried with anhydrous Na_2SO_4 and concentrated *in vacuo*. The resulting residue was purified by silica gel flash chromatography using petroleum ether/ethyl acetate (10:1 to 2:1) eluent to give the desired product **3a** (3.15 g, 72% yield) as white solid.

NOTE: <u>All sulfenamides were obtained as a mixture of rotamers,</u> so the NMR spectra show two sets of signals for each compound, including ^{1}H, ^{13}C and ^{19}F spectrum. <u>Sulfenamides</u> are visualized at 220 or 254 nm of UV and can be stained on I_2/silica gel.

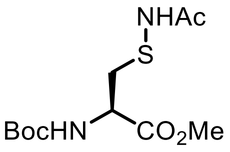

5a

Compound **5a** is an unknown compound.

White solid (R_f = 0.2, PE:EA = 2:1, v/v)

^{1}H NMR (400 MHz, $CDCl_3$) δ 7.02 and 6.58 (2×brs, 1H), 5.63 and 5.45 [2×(d, *J* = 9.1 Hz), 1H], 4.60 – 4.36 (m, 1H), 3.73 (s, 3H), 3.32 – 3.11 (m, 1H), 2.81 – 2.57 (m, 1H), 2.24 and 2.08 (2×s, 3H), 1.44 (s, 9H).

^{13}C NMR (101 MHz, $CDCl_3$) δ 172.72, 171.69, 156.19, 80.56, 52.82, 51.35, 41.71, 28.37, 23.51.

HRMS (ESI) *m/z* Calcd for $C_{11}H_{20}N_2NaO_5S^+$ $[M+Na]^+$: 315.0985, found: 315.0988.

HPLC: The ee value of **5a** (>99.9% ee) was determined by HPLC analysis on a CHIRALPAK® AY-H column (hexanes: isopropanol = 80:20, 1.0 mL/min, T = 30 °C, 220 nm), TR = 6.36 min (minor), 9.52 min (major).

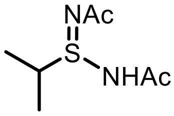

3b

Preparation of **3b**: Isopropanethiol (0.8 mmol, 60.8 mg) was added to a solution of $FeCl_2$-$4H_2O$ (5 mol%, 4 mg) in DCE (0.1 mL) at 50 °C under N_2 atmosphere. 3-Methyldioxazolone **2** (0.4 mmol, 40.4 mg) was added to the mixture and stirred for 24 h. The solvent was removed under reduced pressure, the crude product was purified by silica gel flash chromatography using DCM:MeOH (40:1 to 20:1, v/v) eluent to give the desired product **3b** (50%, 19 mg).

Brown oil (R_f = 0.3, DCM:MeOH = 20:1, v/v)

^{1}H NMR (400 MHz, $CDCl_3$) δ 3.91 (p, *J* = 6.9 Hz, 1H), 2.11 (s, 6H), 1.33 (d, *J* = 6.9 Hz, 6H).

^{13}C NMR (101 MHz, $CDCl_3$) δ 177.76, 49.81, 24.22, 16.80.

HRMS (ESI) *m/z* Calcd for $C_7H_{14}N_2NaO_2S^+$ $[M+Na]^+$: 213.0668, found: 213.0666.

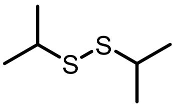

3c

Compound **3c** is a known compound. The spectra data are consistent with those reported in literature. [7]

Colorless oil (R_f = 0.7, 100% PE)

^{1}H NMR (400 MHz, $CDCl_3$) δ 2.96 (hept, *J* = 6.7 Hz, 1H), 1.29 (d, *J* = 6.8 Hz, 6H).

^{13}**C NMR** (101 MHz, $CDCl_3$) δ 41.56, 22.73.

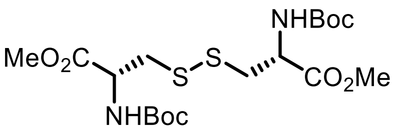

5c

Compound **5c** is a known compound. The spectra data are consistent with those reported in literature. 8

White solid (R_f = 0.15, PE:EA = 5:1, v/v)

^{1}**H NMR** (400 MHz, $CDCl_3$) δ 5.39 (d, *J* = 8.1 Hz, 2H), 4.67 – 4.42 (m, 1H), 3.74 (s, 6H), 3.14 (d, *J* = 5.4 Hz, 4H), 1.43 (s, 18H).

^{13}**C NMR** (101 MHz, $CDCl_3$) δ 171.28, 155.15, 80.39, 52.88, 52.74, 41.37, 28.40.

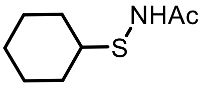

6

Compound **6** is an unknown compound, and was synthesized in 95% yield (65.9 mg, 0.4 mmol scale) under **Conditions [A]**.

Colorless oil (R_f = 0.3, PE:EA = 2:1, v/v)

^{1}**H NMR** (400 MHz, $CDCl_3$) δ 6.82 and 6.39 (2×brs, 1H), 3.00 – 2.70 (2×m, 1H), 2.20 and 2.08 (2×s, 3H), 2.00 – 1.87 (m, 2H), 1.86 – 1.71 (m, 2H), 1.71 – 1.58 (m, 1H), 1.40 – 1.11 (m, 5H).

^{13}**C NMR** (101 MHz, $CDCl_3$) δ 178.52, 172.82, 50.56, 48.81, 30.88, 30.70, 25.76, 25.70, 25.60, 23.47, 19.44.

HRMS (ESI) *m/z* Calcd for $C_8H_{15}NNaOS^+$ $[M+Na]^+$: 196.0767, found: 196.0768.

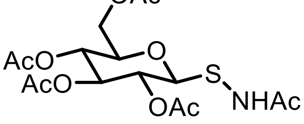

7

Compound **7** is an unknown compound, and was synthesized in 92% yield (77.5 mg, 0.2 mmol scale) under **Conditions [B]**, and was synthesized in 66% yield (55.6 mg, 0.2 mmol scale) under **Conditions [A]**.

White solid (R_f = 0.25, PE:EA = 1:1, v/v)

^{1}H NMR (400 MHz, $CDCl_3$) δ 6.64 and 6.32 (2×brs, 1H), 5.34 – 5.18 (m, 1H), 5.09 – 4.91 (m, 2H), 4.34 – 4.08 (m, 3H), 3.80 – 3.65 (m, 1H), 2.24 – 1.88 (m, 15H)

^{13}C NMR (101 MHz, $CDCl_3$) δ 178.01, 170.70, 170.41, 170.11, 169.41, 86.65, 76.00, 73.55, 73.05, 68.03, 67.50, 62.01, 61.67, 29.75, 20.67, 20.63, 19.82.

HRMS (ESI) *m/z* Calcd for $C_{16}H_{24}NO_{10}S^+$ $[M+H]^+$: 422.1115, found: 422.1112.

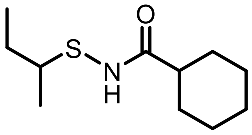

8

Compound **8** is an unknown compound, and was synthesized in 56% yield (48.2 mg, 0.4 mmol scale) under **Conditions [A]**.

Colorless oil (R_f = 0.8, PE:EA = 2:1, v/v)

^{1}H NMR (400 MHz, $CDCl_3$) δ 6.49 and 6.02 (2×brs, 1H), 3.21 – 3.05 (m, 0.12H), 2.95 (h, *J* = 6.8 Hz, 0.88H), 2.84 – 2.71 (m, 0.12H), 2.26 – 2.13 (m, 0.88H), 1.90 – 1.70 (m, 4H), 1.67 – 1.32 (m, 5H), 1.30 – 1.10 (m, 6H), 0.95 (t, *J* = 7.4 Hz, 3H).

^{13}C NMR (101 MHz, $CDCl_3$) δ 178.54, 47.02, 45.98, 29.90, 27.34, 25.69, 17.90, 11.31.

HRMS (ESI) *m/z* Calcd for $C_{11}H_{22}NOS^+$ $[M+H]^+$: 216.1417, found: 216.1415.

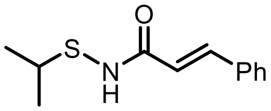

9

Compound **9** is an unknown compound, and was synthesized in 71% yield (31.4 mg, 0.2 mmol scale) under **Conditions [A]**.

Colorless oil (R_f = 0.75, PE:EA = 2:1, v/v)

^{1}H NMR (400 MHz, CD_3CN) δ 7.66 – 7.49 (m, 4H), 7.46 – 7.37 (m, 3H), 6.64 (d, *J* = 15.8 Hz, 1H), 3.21 (p, *J* = 6.7 Hz, 1H), 1.19 (d, *J* = 6.7 Hz, 6H).

^{13}C NMR (101 MHz, CD_3CN) δ 169.01, 142.23, 135.81, 130.96, 129.93, 128.88, 121.52, 41.12, 20.95.

HRMS (ESI) *m/z* Calcd for $C_{12}H_{16}NOS^+$ $[M+H]^+$: 222.0947, found: 222.0948.

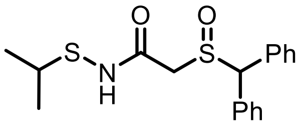

10

Compound **10** is an unknown compound, and was synthesized in 95% yield (66 mg, 0.2 mmol scale) under **Conditions [A]**.

Colorless oil (R_f = 0.5, PE:EA = 1:1, v/v)

^{1}H NMR (400 MHz, $CDCl_3$) δ 7.96 (s, 1H), 7.56 – 7.29 (m, 10H), 5.21 (2×s, 1H), 3.99 – 3.73 (m, 0.2H), 3.55 (d, *J* = 13.7 Hz, 0.9H), 3.33 – 3.16 (m, 1.8H), 2.97 (m, 0.1H), 1.24 – 1.18 (m, 5.4H), 1.13 – 1.07 (m, 0.6H).

^{13}C NMR (101 MHz, $CDCl_3$) δ 167.12, 134.59, 133.83, 129.59, 129.47, 128.96, 128.91, 128.85, 128.73, 71.40, 53.04, 40.86, 20.82, 20.74.

HRMS (ESI) *m/z* Calcd for $C_{18}H_{21}NNaO_2S_2^+$ $[M+Na]^+$: 370.0906, found: 370.0906.

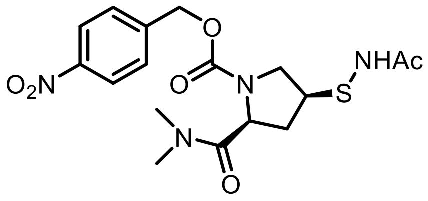

11

Compound **11** is an unknown compound, and was synthesized in 77% yield (63.2 mg, 0.2 mmol scale) under **Conditions [B]**, and was synthesized in 82% yield (67.3 mg, 0.2 mmol scale) under **Conditions [A]**.

White solid (R_f = 0.35, DCM:MeOH = 20:1, v/v)

^{1}H NMR (400 MHz, $CDCl_3$) δ 8.13 (dd, *J* = 8.7, 3.2 Hz, 2H), 7.94 and 7.69 (2×brs, 1H), 7.46 (d, *J* = 8.4 Hz, 1H), 7.38 (d, *J* = 8.4 Hz, 1H), 5.30 – 4.94 (m, 2H), 4.74 – 4.54 (m, 1H), 3.93 (m, 1H), 3.57 – 3.32 (m, 2H), 3.08 – 2.82 (4×s, 6H), 2.70 – 2.53 (m, 1H), 2.03 (s, 3H), 1.97 – 1.82 (m, 1H).

^{13}C NMR (101 MHz, $CDCl_3$) δ 173.53, 173.42, 171.28, 171.24, 153.93, 153.48, 147.54, 144.01, 143.90, 128.12, 127.82, 123.77, 123.64, 65.73, 56.47, 56.37, 51.69, 50.92, 46.74, 45.85, 37.09, 36.87, 36.20, 36.12, 35.20, 34.67, 23.29.

HRMS (ESI) *m/z* Calcd for $C_{17}H_{23}N_4O_6S^+$ $[M+H]^+$: 411.1333, found: 411.1332.

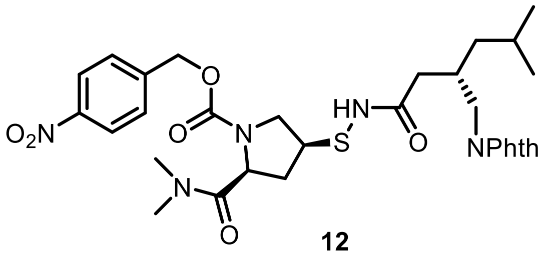

12

Compound **12** is an unknown compound, and was synthesized in 84% yield (107.5 mg, 0.2 mmol scale) under **Conditions [B]**, and was synthesized in 67% yield (85.7 mg, 0.2 mmol scale) under **Conditions [A]**.

White solid (R_f = 0.6, DCM:MeOH = 20:1, v/v)

^{1}H NMR (400 MHz, $CDCl_3$) δ 8.18 – 8.08 (m, 2H), 7.82 – 7.74 (m, 2H), 7.72 – 7.64 (td, *J* = 5.1, 2.4 Hz, 2.5H), 7.56 (s, 0.5H), 7.50 – 7.35 (m, 2H), 5.32 – 4.92 (m, 2H), 4.71 – 4.58 (m, 1H), 4.08 – 3.94 (m, 1H), 3.69 – 3.48 (m, 3H), 3.44 – 3.33 (m, 1H), 3.07 – 2.83 (m, 6H), 2.68 – 2.52 (m, 1H), 2.43 – 2.32 (m, 1H), 2.30 – 2.22 (m, 2H), 1.95 – 1.82 (m, 1H), 1.81 – 1.68 (m, 1H), 1.22 – 1.08 (m, 2H), 0.93 – 0.81 (m, 6H).

^{13}C NMR (101 MHz, $CDCl_3$) δ 174.41, 171.12, 170.98, 169.11, 153.93, 153.37, 147.54, 144.16, 144.02, 134.25, 134.20, 131.91, 131.87, 128.03, 127.88, 123.76, 123.65, 123.42, 123.39, 123.36, 65.70, 56.45, 56.36, 51.83, 51.26, 47.13, 46.24, 41.77, 41.35, 39.63, 36.99, 36.80, 36.09, 34.96, 34.19, 33.77, 33.68, 25.23, 22.67, 22.62.

HRMS (ESI) *m/z* Calcd for $C_{31}H_{37}N_5NaO_8S^+$ $[M+Na]^+$: 662.2255, found: 662.2257.

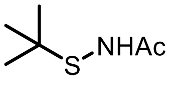

13

Compound **13** is a known compound, and was synthesized in 95% yield (55.9 mg, 0.4 mmol scale) under **Conditions [A]**. The spectra data are consistent with those reported in literature.[9]

Colorless oil (R_f = 0.4, PE:EA = 2:1, v/v)

^{1}H NMR (400 MHz, $CDCl_3$) δ 6.92 and 6.56 (2×brs, 1H), 2.18 and 2.10 (2×s, 3H), 1.23 (s, 9H).

^{13}C NMR (101 MHz, $CDCl_3$) δ 178.64, 172.89, 48.64, 48.60, 28.60, 28.03, 23.51, 19.33.

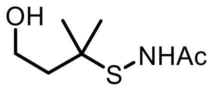

14

Compound **14** is an unknown compound, and was synthesized in 95% yield (67.4 mg, 0.4 mmol scale) under **Conditions [A]**.

Colorless oil (R_f = 0.2, DCM:MeOH = 20:1, v/v)

^{1}H NMR (400 MHz, $CDCl_3$) δ 7.46 and 7.08 (2×brs, 1H), 3.86 – 3.71 (m, 1H), 3.48 and 3.06 (2×brs, 1H), 2.19 and 2.10 (2×s, 3H), 1.84 – 1.67 (m, 2H), 1.27 – 1.14 (m, 6H).

^{13}C NMR (101 MHz, $CDCl_3$) δ 179.05, 174.03, 59.22, 58.83, 50.47, 50.30, 42.71, 42.44, 26.62, 26.04, 23.59, 19.72.

HRMS (ESI) *m/z* Calcd for $C_7H_{16}NO_2S^+$ $[M+H]^+$: 178.0896, found: 178.0895.

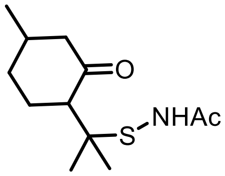

15

Compound **15** is an unknown compound, and was synthesized in 62% yield (60.4 mg, 0.4 mmol scale) under **Conditions [A]**.

White solid (R_f = 0.27, PE:EA = 2:1, v/v)

^{1}H NMR (400 MHz, $CDCl_3$) δ 6.81 and 6.40 (2×brs, 1H), 2.61 – 2.41 (m, 2H), 2.36 – 2.23 (m, 1H), 2.19 and 2.11 (2×s, 2H), 2.05 (t, J = 12.2 Hz, 1H), 1.96 – 1.80 (m, 2H), 1.61 – 1.48 (m, 1H), 1.41 – 1.32 (m, 4H), 1.28 – 1.21 (m, 3H), 1.04 – 0.95 (m, 3H).

^{13}C NMR (101 MHz, $CDCl_3$) δ 217.30, 212.10, 178.41, 173.00, 58.61, 57.43, 52.43, 52.26, 52.07, 36.89, 36.62, 34.51, 29.89, 29.48, 25.34, 23.61, 22.71, 22.33.

HRMS (ESI) *m/z* Calcd for $C_{12}H_{22}NO_2S^+$ $[M+H]^+$: 244.1366, found: 244.1364.

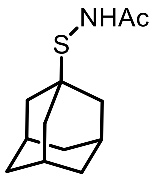

16

Compound **16** is an unknown compound, and was synthesized in 96% yield (85.7 mg, 0.4 mmol scale) under **Conditions [A]**.

White solid (R_f = 0.42, PE:EA = 2:1, v/v)

^{1}H NMR (400 MHz, $CDCl_3$) δ 6.79 and 6.43 (2×brs, 1H), 2.18 and 2.10 (2×s, 3H), 2.08 – 1.96 (m, 3H), 1.82 – 1.73 (m, 6H), 1.72 – 1.55 (m, 6H).

^{13}C NMR (101 MHz, $CDCl_3$) δ 178.82, 173.11, 50.59, 41.16, 40.72, 36.21, 36.13, 29.37, 29.28, 23.54, 19.42.

HRMS (ESI) *m/z* Calcd for $C_{12}H_{20}NOS^+$ $[M+H]^+$: 226.1260, found: 226.1258.

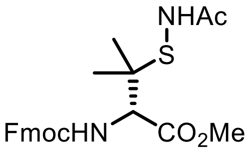

17

Compound **17** is an unknown compound, and was synthesized in 78% yield (69 mg, 0.2 mmol scale) under **Conditions [A]**.

White solid (R_f = 0.54, PE:EA = 2:1, v/v)

^{1}H NMR (400 MHz, $CDCl_3$) δ 7.76 (d, *J* = 7.5 Hz, 2H), 7.65 – 7.55 (m, 2H), 7.40 (t, *J* = 7.5 Hz, 2H), 7.35 – 7.28 (m, 2H), 7.18 and 6.83 (2×brs, 1H), 5.91 and 5.68 (2×d, 1H), 4.55 – 4.45 (m, 1H), 4.45 – 4.31 (m, 2H), 4.28 – 4.16 (m, 1H), 3.77 (s, 3H), 2.22 and 2.13 (2×s, 3H), 1.36 and 1.34 (2×s, 3H), 1.17 (s, 3H).

^{13}C NMR (101 MHz, $CDCl_3$) δ 172.69, 170.95, 156.99, 143.68, 141.38, 127.87, 127.19, 127.16, 125.20, 125.12, 120.11, 67.57, 59.09, 54.34, 52.52, 47.16, 25.29, 23.64, 19.68.

HRMS (ESI) *m/z* Calcd for $C_{23}H_{27}N_2O_5S^+$ $[M+H]^+$: 443.1635, found: 443.1642.

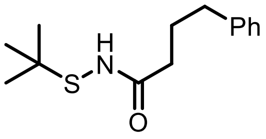

18

Compound **18** is an unknown compound, and was synthesized in 70% yield (70.4 mg, 0.4 mmol scale) under **Conditions [A]**.

Colorless oil (R_f = 0.7, PE:EA = 5:1, v/v)

^{1}H NMR (400 MHz, $CDCl_3$) δ 7.32 – 7.23 (m, 2H), 7.22 – 7.11 (m, 3H), 6.62 and 6.37 (2×brs, 1H), 2.66 (t, J = 7.6 Hz, 2H), 2.62 – 2.54 (m, 0.6H), 2.32 (t, J = 7.4 Hz, 1.4H), 2.07 – 1.88 (m, 2H), 1.26 and 1.22 (2×s, 9H).

^{13}C NMR (101 MHz, $CDCl_3$) δ 180.44, 175.36, 141.36, 128.54, 128.47, 126.07, 48.63, 36.13, 35.23, 30.30, 28.66, 27.99, 27.39, 26.58.

HRMS (ESI) *m/z* Calcd for $C_{14}H_{21}NNaOS^+$ $[M+Na]^+$: 274.1236, found: 274.1242.

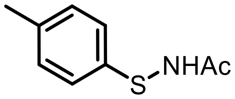

19

Compound **19** is a known compound, and was synthesized in 61% yield (44.2 mg, 0.4 mmol scale) under **Conditions [B]**. The spectra data are consistent with reference 43 in the main text.

White solid (R_f = 0.2, PE:EA = 5:1, v/v)

^{1}H NMR (400 MHz, $CDCl_3$) δ 7.38 and 6.80 (2×brs, 1H), 7.24 – 7.00 (m, 4H), 2.32 and 2.29 (2×s, 3H), 2.20 and 2.11 (2×s, 3H).

^{13}C NMR (101 MHz, $CDCl_3$) δ 178.27, 172.41, 141.93, 137.41, 137.23, 135.35, 135.13, 130.15, 129.80, 127.02, 124.60, 23.39, 21.13, 21.08, 19.48.

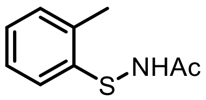

20

Compound **20** is an unknown compound, and was synthesized in 80% yield (58 mg, 0.4 mmol scale) under **Conditions [B]**.

White solid (R_f = 0.38, PE:EA = 2:1, v/v)

¹H NMR (400 MHz, $CDCl_3$) δ 7.25 – 7.03 (m, 4H), 6.99 and 6.56 (2×brs, 1H), 2.29 and 2.28 (2×s, 3H), 2.19 (s, 3H).

¹³C NMR (101 MHz, $CDCl_3$) δ 178.45, 172.47, 137.59, 137.11, 133.48, 132.73, 130.63, 130.36, 126.97, 126.63, 126.34, 126.20, 123.64, 122.29, 23.40, 19.40, 19.01, 18.73.

HRMS (ESI) *m/z* Calcd for $C_9H_{12}NOS^+$ $[M+H]^+$: 182.0634, found: 182.0635.

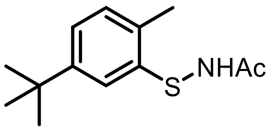

21

Compound **21** is an unknown compound, and was synthesized in 58% yield (55.1 mg, 0.4 mmol scale) under **Conditions [B]**.

White solid (R_f = 0.6, PE:EA = 2:1, v/v)

¹H NMR (400 MHz, $CDCl_3$) δ 7.24 – 7.04 (m, 3H), 7.02 and 6.61 (2×brs, 1H), 2.29 and 2.26 (2×s, 3H), 2.20 and 2.18 (2×s, 3H), 1.29 and 1.28 (2×s, 9H).

¹³C NMR (101 MHz, $CDCl_3$) δ 178.28, 172.08, 150.22, 149.62, 136.80, 136.29, 131.65, 130.51, 130.21, 123.99, 123.80, 122.23, 120.19, 34.73, 34.61, 31.36, 29.80, 23.33, 19.34, 18.69, 18.28.

HRMS (ESI) *m/z* Calcd for $C_{13}H_{20}NOS^+$ $[M+H]^+$: 238.1260, found: 238.1259.

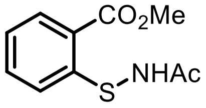

22

Compound **22** is a known compound, and was synthesized in 40% yield (36 mg, 0.4 mmol scale) under **Conditions [B]**. The spectra data are consistent with reference 43 in the main text.

Colorless oil (R_f = 0.5, PE:EA = 1:1, v/v)

¹H NMR (400 MHz, $CDCl_3$) δ 8.03 (d, *J* = 7.8 Hz, 0.5H), 7.94 (d, *J* = 7.8 Hz, 0.5H), 7.53 (t, *J* = 7.8 Hz, 0.5H), 7.48 – 7.31 (m, 1.5H), 7.25 – 7.16 (m, 1H), 7.12 (t, *J* = 7.5 Hz, 0.5H), 6.69 (brs, 0.5H), 3.92 and 3.87 (2×s, 3H), 2.19 and 2.11 (2×s, 3H).

^{13}**C NMR** (101 MHz, $CDCl_3$) δ 178.34, 172.66, 167.18, 167.06, 145.79, 144.88, 141.58, 141.54, 133.64, 133.07, 131.40, 131.15, 125.08, 124.54, 124.14, 123.87, 122.01, 121.64, 52.60, 52.42, 23.48, 19.40.

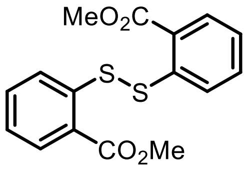

22’

Compound **22’** is a known compound, and was synthesized in 51% yield (68.1 mg, 0.4 mmol scale) under **Conditions [B]**. The spectra data are consistent with those reported in literature.[10]

White solid (R_f = 0.25, PE:EA = 20:1, v/v)

^{1}**H NMR** (400 MHz, $CDCl_3$) δ 8.05 (d, *J* = 9.3 Hz, 2H), 7.75 (d, *J* = 8.2 Hz, 2H), 7.45 – 7.36 (m, 2H), 7.22 (t, *J* = 7.5 Hz, 2H), 3.98 (s, 6H).

^{13}**C NMR** (101 MHz, $CDCl_3$) δ 167.02, 140.48, 133.19, 131.58, 127.42, 125.95, 125.60, 52.50.

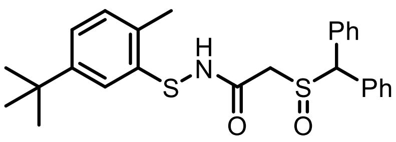

23

Compound **23** is an unknown compound, and was synthesized in 95% yield (85.7 mg, 0.2 mmol scale) under **Conditions [B]**.

Colorless oil (R_f = 0.6, PE:EA = 2:1, v/v)

^{1}**H NMR** (400 MHz, $CDCl_3$) δ 8.39 (s, 1H), 7.49 (d, *J* = 2.0 Hz, 1H), 7.42 – 7.32 (m, 10H), 7.23 – 7.18 (m, 1H), 7.14 and 7.12 (2×s, 1H), 5.11 (s, 1H), 3.60 (d, *J* = 14.0 Hz, 1H), 3.26 (d, *J* = 14.0 Hz, 1H), 2.39 (s, 3H), 1.27 (s, 9H).

^{13}**C NMR** (101 MHz, $CDCl_3$) δ 166.56, 149.84, 135.53, 134.50, 133.85, 133.63, 130.32, 129.52, 129.49, 128.96, 128.91, 128.74, 125.63, 125.10, 71.31, 71.24, 52.43, 34.65, 31.43, 31.37, 19.26.

HRMS (ESI) *m/z* Calcd for $C_{26}H_{29}NNaO_2S_2^+$ $[M+Na]^+$: 474.1532, found: 474.1538.

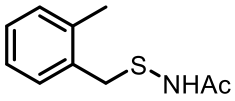

24

Compound **24** is an unknown compound, and was synthesized in 65% yield (50.8 mg, 0.4 mmol scale) under **Conditions [B]**.

White solid (R_f = 0.33, PE:EA = 2:1, v/v)

^{1}H NMR (400 MHz, $CDCl_3$) δ 7.23 – 6.98 (m, 4H), 6.13 and 6.03 (2×brs, 1H), 3.96 and 3.89 (2×s, 2H), 2.40 (s, 3H), 2.03 and 1.77 (2×s, 3H).

^{13}C NMR (101 MHz, $CDCl_3$) δ 178.16, 172.22, 137.16, 133.69, 130.85, 130.66, 130.47, 128.20, 127.87, 126.41, 125.95, 43.41, 40.31, 23.46, 19.26.

HRMS (ESI) *m/z* Calcd for $C_{10}H_{14}NOS^+$ $[M+H]^+$: 196.0791, found: 196.0790.

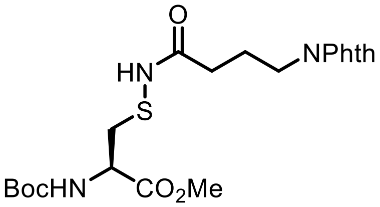

25

Compound **25** is an unknown compound, and was synthesized in 79% yield (73.5 mg, 0.2 mmol scale) under **Conditions [B]**.

Colorless oil (R_f = 0.4, PE:EA = 1:1, v/v)

^{1}H NMR (400 MHz, $CDCl_3$) δ 7.85 – 7.78 (m, 2H), 7.73 – 7.66 (m, 2H), 7.17 and 6.60 (2×brs, 1H), 5.72 (d, *J* = 9.1 Hz, 0.87H), 5.49 (s, 0.13H), 4.60 – 4.29 (m, 1H), 3.79 – 3.63 (m, 5H), 3.30 – 3.08 (m, 1H), 2.81 – 2.61 (m, 1H), 2.32 and 2.20 (2×t, *J* = 7.3 Hz and 7.0 Hz, 2H), 1.96 – 2.07 (m, 2H), 1.41 and 1.22 (2×s, 9H).

^{13}C NMR (101 MHz, $CDCl_3$) δ 174.67, 171.70, 168.65, 156.10, 134.18, 132.02, 123.42, 80.43, 52.80, 51.71, 41.83, 37.20, 33.84, 28.39, 28.31, 24.78.

HRMS (ESI) *m/z* Calcd for $C_{21}H_{28}N_3O_7S^+$ $[M+H]^+$: 466.1642, found: 466.1641.

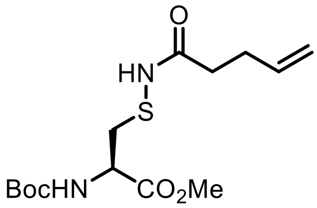

26

Compound **26** is an unknown compound, and was synthesized in 53% yield (35.2 mg, 0.2 mmol scale) under **Conditions [B]**.

Colorless oil (R_f = 0.7, PE:EA = 1:1, v/v)

^{1}H NMR (400 MHz, $CDCl_3$) δ 6.99 (s, 1H), 5.88 – 5.74 (m, 1H), 5.60 (d, *J* = 9.1 Hz, 1H), 5.13 – 4.89 (m, 2H), 4.53 – 4.36 (m, 1H), 3.73 (s, 3H), 3.36 – 3.19 (m, 1H), 2.71 – 2.53 (m, 1H), 2.39 (s, 3H), 1.44 (s, 9H).

^{13}C NMR (101 MHz, $CDCl_3$) δ 174.94, 171.69, 156.23, 136.58, 116.06, 80.55, 52.84, 51.32, 41.90, 36.05, 29.55, 28.38.

HRMS (ESI) *m/z* Calcd for $C_{14}H_{25}N_2O_5S^+$ $[M+H]^+$: 333.1479, found: 333.1477.

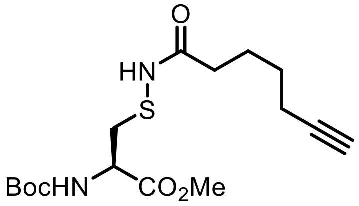

27

Compound **27** is an unknown compound, and was synthesized in 67% yield (48 mg, 0.2 mmol scale) under **Conditions [B]**.

Colorless oil (R_f = 0.7, PE:EA = 1:1, v/v)

^{1}H NMR (400 MHz, $CDCl_3$) δ 7.03 and 6.57 (2×brs, 1H), 5.64 (d, *J* = 9.2 Hz, 0.9H), 5.45 (s, 0.1H), 4.60 – 4.28 (m, 1H), 3.72 (s, 4H), 3.24 (dd, *J* = 14.6, 3.9 Hz, 1H), 2.63 (dd, *J* = 14.4, 8.7 Hz, 1H), 2.30 (t, *J* = 7.5 Hz, 2H), 2.18 (td, *J* = 7.0, 2.6 Hz, 2H), 1.92 (t, *J* = 2.7 Hz, 1H), 1.75 (p, *J* = 7.5 Hz, 2H), 1.60 – 1.48 (m, 2H), 1.43 (s, 9H).

^{13}C NMR (101 MHz, $CDCl_3$) δ 175.28, 171.63, 156.18, 83.95, 80.48, 68.80, 52.80, 51.31, 41.81, 36.16, 28.35, 27.84, 24.64, 18.20.

HRMS (ESI) *m/z* Calcd for $C_{16}H_{27}N_2O_5S^+$ $[M+H]^+$: 359.1635, found: 359.1631.

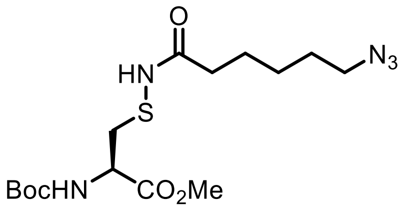

28

Compound **28** is an unknown compound, and was synthesized in 46% yield (36 mg, 0.2 mmol scale) under **Conditions [B]**.

Colorless oil (R_f = 0.3, PE:EA = 2:1, v/v)

^{1}H NMR (400 MHz, $CDCl_3$) δ 6.99 and 6.57 (2×brs, 1H), 5.70 – 5.32 (m, 1H), 4.58 – 4.31 (m, 1H), 3.74 (s, 3H), 3.52 (t, J = 6.6 Hz, 0.2H), 3.33 – 3.20 (m, 2.8H), 2.68 – 2.48 (m, 1H), 2.30 (t, J = 7.5 Hz, 1.8H), 2.21 – 2.13 (s, 0.2H), 1.73 – 1.63 (m, 2H), 1.63 – 1.54 (m, 2H), 1.48 – 1.34 (m, 11H).

^{13}C NMR (101 MHz, $CDCl_3$) δ 175.32, 171.64, 156.26, 80.62, 52.86, 51.30, 41.86, 36.58, 28.66, 28.38, 26.36, 25.09.

HRMS (ESI) *m/z* Calcd for $C_{15}H_{27}N_5NaO_5S^+$ $[M+Na]^+$: 412.1625, found: 412.1630.

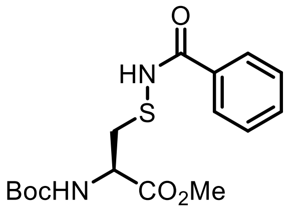

29

Compound **29** is an unknown compound, and was synthesized in 76% yield (53.9 mg, 0.2 mmol scale) under **Conditions [B]**.

White solid (R_f = 0.5, PE:EA = 1:1, v/v)

^{1}H NMR (400 MHz, $CDCl_3$) δ 7.87 (d, *J* = 8 Hz, 2H), 7.79 (brs, 1H), 7.58 – 7.50 (m, 1H), 7.48 – 7.41 (m, 2H), 5.64 (d, *J* = 9.1 Hz, 0.9H), 5.40 (d, 8.1 Hz, 0.1H), 4.64 – 4.40 (m, 1H), 3.76 and 3.66 (2×s, 3H), 3.41 (dd, *J* = 14.4, 4.0 Hz, 1H), 3.46 – 3.10 (2×m, 1H), 2.69 (dd, *J* = 14.4, 8.9 Hz, 1H), 1.46 and 1.44 (2×s, 9H).

^{13}C NMR (101 MHz, $CDCl_3$) δ 171.72, 169.33, 156.36, 133.26, 132.41, 128.82, 127.70, 80.65, 52.82, 51.34, 41.81, 28.37.

HRMS (ESI) *m/z* Calcd for $C_{16}H_{23}N_2O_5S^+$ $[M+H]^+$: 355.1322, found: 355.1321.

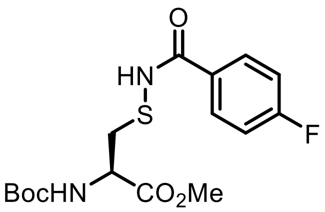

30

Compound **30** is an unknown compound, and was synthesized in 72% yield (53.6 mg, 0.2 mmol scale) under **Conditions [B]**.

White solid (R_f = 0.55, PE:EA = 1:1, v/v)

^{1}H NMR (400 MHz, $CDCl_3$) δ 7.98 – 7.82 (m, 3H), 7.12 (t, *J* = 8.6 Hz, 2H), 5.66 (d, *J* = 9.1 Hz, 1H), 4.49 (td, *J* = 9.2, 3.9 Hz, 1H), 3.66 (s, 3H), 3.41 (dd, *J* = 14.4, 4.0 Hz, 1H), 2.64 (dd, *J* = 14.4, 9.2 Hz, 1H), 1.45 (s, 9H).

^{13}C NMR (101 MHz, $CDCl_3$) δ 171.63, 168.29, 166.54, 164.03, 156.46, 130.28, 130.18, 129.46, 116.00, 115.78, 80.74, 52.84, 51.30, 41.75, 28.36.

^{19}F NMR (376 MHz, $CDCl_3$) δ -106.65.

HRMS (ESI) *m/z* Calcd for $C_{16}H_{22}FN_2O_5S^+$ $[M+H]^+$: 373.1228, found: 373.1225.

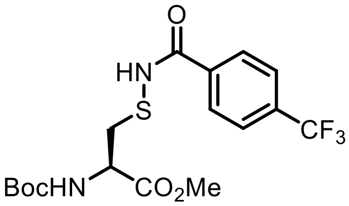

31

Compound **31** is an unknown compound, and was synthesized in 55% yield (46.5 mg, 0.2 mmol scale) under **Conditions [B]**.

White solid (R_f = 0.62, PE:EA = 1:1, v/v)

^{1}H NMR (400 MHz, $CDCl_3$) δ 8.16 (brs, 1H), 8.01 (d, *J* = 8.0 Hz, 2H), 7.71 (d, *J* = 8.1 Hz, 2H), 5.65 (d, *J* = 9.1 Hz, 0.89H), 5.41 (d, *J* = 8.1 Hz, 0.11H), 4.63 – 4.39 (2×m, 1H), 3.74 and 3.68 (2×s, 3H), 3.52 – 3.08 (2×m, 1H), 2.61 (dd, *J* = 14.4, 9.5 Hz, 1H), 1.44 and 1.43 (2×s, 9H).

^{13}C NMR (101 MHz, $CDCl_3$) δ 171.53, 168.15, 156.61, 136.56, 134.12, 133.80, 128.24, 125.89, 125.86, 125.03, 122.32, 80.94, 52.92, 51.24, 41.81, 28.37.

^{19}F NMR (376 MHz, $CDCl_3$) δ -63.07.

HRMS (ESI) *m/z* Calcd for $C_{17}H_{22}F_3N_2O_5S^+$ $[M+H]^+$: 423.1196, found: 423.1192.

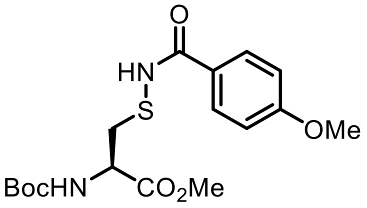

32

Compound **32** is an unknown compound, and was synthesized in 44% yield (33.8 mg, 0.2 mmol scale) under **Conditions [B]**.

White solid (R_f = 0.4, PE:EA = 1:1, v/v)

^{1}H NMR (400 MHz, $CDCl_3$) δ 7.84 (d, *J* = 8.4 Hz, 2H), 7.73 (brs, 1H), 6.92 (d, *J* = 8.3 Hz, 2H), 5.71 (d, *J* = 9.2 Hz, 1H), 4.50 (td, *J* = 8.9, 4.0 Hz, 1H), 3.84 (s, 3H), 3.64 (s, 3H), 3.36 (dd, *J* = 14.4, 4.0 Hz, 1H), 2.71 (dd, *J* = 14.4, 8.7 Hz, 1H), 1.45 (s, 9H).

^{13}C NMR (101 MHz, $CDCl_3$) δ 171.82, 168.77, 162.94, 156.36, 129.70, 125.53, 114.01, 80.61, 55.54, 52.79, 51.32, 41.81, 28.39.

HRMS (ESI) *m/z* Calcd for $C_{17}H_{25}N_2O_6S^+$ $[M+H]^+$: 385.1428, found: 385.1425.

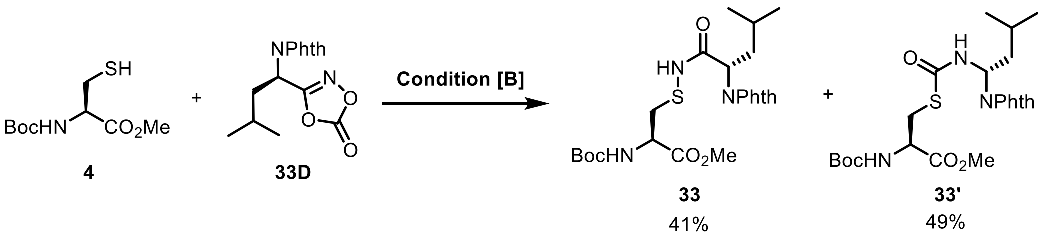

Supplementary Figure 12. Formation of thiourea **33'** using dioxazolone derived from α-amino acid as nitrene precursor.

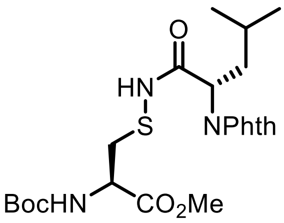

33

Compound **33** is an unknown compound, and was synthesized in 41% yield (40.5 mg, 0.2 mmol scale) under **Conditions [B]**.

Colorless oil (R_f = 0.4, PE:EA = 2:1, v/v)

^{1}H NMR (400 MHz, $CDCl_3$) δ 7.93 (brs, 1H), 7.87 – 7.81 (m, 2H), 7.74 – 7.68 (m, 2H), 5.42 (d, J = 9.2 Hz, 1H), 4.96 (dd, J = 11.7, 4.4 Hz, 1H), 4.31 (td, J = 9.8, 3.8 Hz, 1H), 3.73 (s, 3H), 3.39 (dd, J = 14.1, 3.8 Hz, 1H), 2.42 – 2.23 (m, 2H), 2.05 – 1.92 (m, 1H), 1.47 – 1.33 (m, 1H), 1.16 (s, 9H), 0.91 (dd, J = 6.6, 4.2 Hz, 6H).

^{13}C NMR (101 MHz, $CDCl_3$) δ 171.96, 171.55, 167.96, 156.44, 134.27, 131.96, 123.67, 80.45, 53.20, 52.88, 50.73, 41.73, 37.07, 28.07, 25.28, 23.24, 21.18.

HRMS (ESI) *m/z* Calcd for $C_{23}H_{31}N_3NaO_7S^+$ $[M+Na]^+$: 516.1775, found: 516.1780.

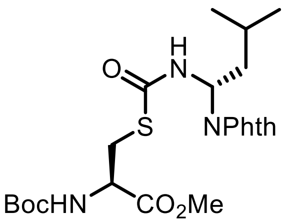

33'

Compound **33’** is an unknown compound, and was synthesized in 49% yield (48.4 mg, 0.2 mmol scale) under **Conditions [B]**.

Colorless oil (R_f = 0.6, PE:EA = 2:1, v/v)

^{1}H NMR (400 MHz, $CDCl_3$) δ 7.88 – 7.80 (m, 2H), 7.76 – 7.68 (m, 2H), 6.64 (d, J = 9.4 Hz, 1H), 6.22 (d, J = 8.8 Hz, 1H), 5.38 (d, J = 8.1 Hz, 0.85H), 5.14 (s, 0.15H), 4.64 – 4.55 (m, 0.15H), 4.51 – 4.38 (m, 0.85H), 3.75 (s, 0.5H), 3.69 (s, 2.5H), 3.31 (d, J = 5.8 Hz, 1.7H), 3.15 (d, J = 5.3 Hz, 0.3H), 1.90 (t, J = 7.4 Hz, 2H), 1.63 – 1.49 (m, 1H), 1.43 (s, 1.5H), 1.38 (s, 7.5H), 0.95 (dd, J = 11.6, 6.6 Hz, 6H).

^{13}C NMR (101 MHz, $CDCl_3$) δ 171.13, 167.53, 155.33, 134.44, 131.73, 123.79, 123.68, 80.12, 57.05, 54.01, 52.70, 52.64, 42.09, 32.10, 28.41, 28.31, 25.00, 22.36.

HRMS (ESI) *m/z* Calcd for $C_{23}H_{31}N_3NaO_7S^+$ $[M+Na]^+$: 516.1775, found: 516.1780.

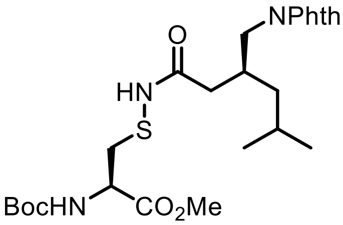

34

Compound **34** is an unknown compound, and was synthesized in 75% yield (78.2 mg, 0.2 mmol scale) under **Conditions [B]**.

White solid (R_f = 0.7, PE:EA = 1:1, v/v)

^{1}H NMR (400 MHz, $CDCl_3$) δ 7.85 – 7.77 (m, 2H), 7.73 – 7.65 (m, 2H), 5.69 (d, *J* = 8.6 Hz, 0.91H), 5.43 (s, 0.09 H), 4.58 – 4.24 (m, 1H), 3.74 – 3.64 (m, 4H), 3.63 – 3.53 (m, 1H), 3.27 – 3.08 (m, 1H), 2.74 (dd, *J* = 14.4, 8.2 Hz, 1H), 2.49 – 2.36 (m, 1H), 2.32 – 2.15 (m, 2H), 1.81 – 1.66 (m, 1H), 1.40 (s, 9H), 1.25 – 1.09 (m, 2H), 0.92 (d, *J* = 6.5 Hz, 3H), 0.87 (d, *J* = 6.5 Hz, 3H).

^{13}C NMR (101 MHz, $CDCl_3$) δ 174.29, 171.68, 169.00, 156.01, 134.16, 131.98, 123.41, 80.32, 52.69, 51.76, 41.70, 41.56, 39.88, 33.65, 28.34, 25.30, 22.81, 22.53.

HRMS (ESI) *m/z* Calcd for $C_{25}H_{36}N_3O_7S^+$ $[M+H]^+$: 522.2268, found: 522.2268.

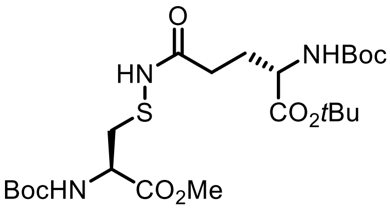

35

Compound **35** is an unknown compound, and was synthesized in 61% yield (65.4 mg, 0.2 mmol scale) under **Conditions [B]**.

White solid (R_f = 0.3, PE:EA = 2:1, v/v)

^{1}H NMR (400 MHz, $CDCl_3$) δ 7.30 and 6.64 (brs×2, 1H), 5.75 (d, *J* = 8.8 Hz, 0.9H), 5.47 (s, 0.1H), 5.24 (d, *J* = 8.1 Hz, 0.9H), 5.08 (s, 0.1H), 4.46 (s, 1H), 4.12 (s, 1H), 3.71 (s, 3H), 3.20 (dd, *J* = 14.6, 4.3 Hz, 1H), 2.85 – 2.64 (m, 1H), 2.42 – 2.26 (m, 2H), 2.21 – 2.07 (m, 1H), 1.95 – 1.79 (m, 1H), 1.46 – 1.37 (m, 27H).

^{13}C NMR (101 MHz, $CDCl_3$) δ 174.75, 171.50, 171.21, 155.92, 82.34, 80.28, 79.98, 53.36, 52.62, 51.52, 41.58, 32.71, 29.08, 28.34, 28.24, 28.00, 27.91.

HRMS (ESI) *m/z* Calcd for $C_{23}H_{41}N_3NaO_9S^+$ $[M+Na]^+$: 558.2456, found: 558.2460.

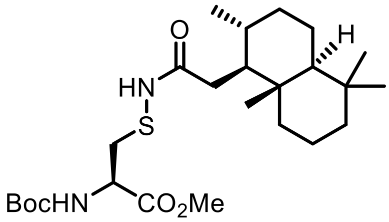

36

Compound **36** is an unknown compound, and was synthesized in 62% yield (60.1 mg, 0.2 mmol scale) under **Conditions [B]**.

Colorless oil (R_f = 0.25, PE:EA = 5:1, v/v)

^{1}H NMR (400 MHz, $CDCl_3$) δ 6.97 (brs, 1H), 5.71 – 5.35 (m, 1H), 4.59 – 4.35 (m, 1H), 3.74 (s, 3H), 3.32 (d, *J* = 14.4 Hz, 1H), 2.60 – 2.45 (m, 1H), 2.45 – 2.32 (m, 1H), 2.02 – 1.86 (m, 2H), 1.78 – 1.66 (m, 1H), 1.63 – 1.54 (m, 2H), 1.54 – 1.28 (m, 14H), 1.24 – 0.91 (m, 5H), 0.89 – 0.73 (m, 12H).

^{13}C NMR (101 MHz, $CDCl_3$) δ 176.60, 171.77, 156.33, 80.51, 54.75, 53.65, 52.85, 51.28, 51.11, 41.99, 38.96, 37.76, 36.55, 34.25, 33.54, 33.48, 33.37, 28.45, 28.33, 21.85, 21.81, 20.76, 20.72, 18.80, 14.63, 14.60.

HRMS (ESI) *m/z* Calcd for $C_{25}H_{44}N_2NaO_5S^+$ $[M+Na]^+$: 507.2863, found: 507.2865.

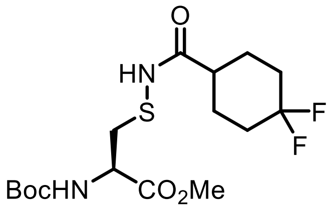

37

Compound **37** is an unknown compound, and was synthesized in 63% yield (49.9 mg, 0.2 mmol scale) under **Conditions [B]**.

Colorless oil (R_f = 0.45, PE:EA = 1:1, v/v)

^{1}H NMR (400 MHz, $CDCl_3$) δ 7.14 (brs, 1H), 5.56 (d, *J* = 9.1 Hz, 1H), 4.49 – 4.33 (m, 1H), 3.74 (s, 3H), 3.31 (d, *J* = 12 Hz, 1H), 2.62 – 2.44 (m, 1H), 2.40 – 2.24 (m, 1H), 2.24 – 2.07 (m, 2H), 2.01 – 1.66 (m, 6H), 1.45 (s, 9H).

^{13}**C NMR** (101 MHz, $CDCl_3$) δ 176.65, 171.51, 156.35, 124.97, 122.57, 120.17, 80.70, 52.90, 51.26, 42.97, 41.94, 33.04, 32.99, 32.80, 32.75, 32.55, 32.50, 28.39, 26.04, 25.95, 25.84, 25.75.

^{19}**F NMR** (376 MHz, $CDCl_3$) δ -92.89, -93.52, -100.28, -100.91.

HRMS (ESI) *m/z* Calcd for $C_{16}H_{27}F_2N_2O_5S^+$ $[M+H]^+$: 397.1603, found: 397.1601.

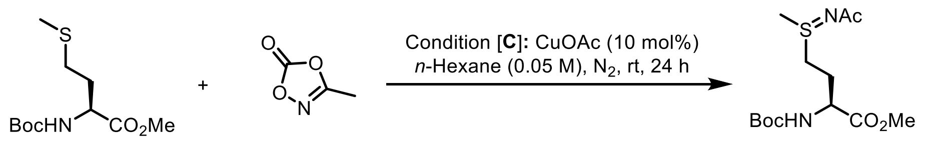

38S
2.0 equiv

2
0.2 mmol

38
75%, dr = 1:1

Supplementary Figure 13. General procedures for the the reaction of **39S** with **2**

Conditions [C]:

To a solution of Boc-protected methionine methyl ester **38S** (105.2 mg, 0.4 mmol, 2 equiv) and CuOAc (2.5 mg, 0.02 mmol, 10 mol%) in *n*-hexane (4 mL, 0.05 M), 3-methyldioxazolone **2** (20.2 mg, 0.2 mmol, 1 equiv) was added. The reaction mixture was stirred under N_2 atomosphere for 24 hours at rt. The reaction mixture was concentrated *in vacuo*. The resulting residue was purified by silica gel flash chromatography using DCM/MeOH (100:1 to 20:1, v/v) eluent to give the desired product **38** (48 mg, 75% yield, dr = 1:1, mixture of diastereoisomer) as a colorless oil.

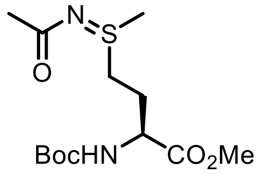

38

Compound **38** is an unknown compound.

Colorless oil (R_f = 0.2, DCM:MeOH = 20:1, v/v)

^{1}**H NMR** (400 MHz, $CDCl_3$) δ 5.85 (d, *J* = 7.9 Hz, 0.5H), 5.66 (d, *J* = 7.7 Hz, 0.5H), 4.40 – 4.22 (m, 1H), 3.70 (d, *J* = 1.8 Hz, 3H), 3.02 – 2.86 (m, 2H), 2.58 and 2.57 (2×s, 3H), 2.34 – 2.19 (m, 1H), 2.12 – 2.00 (m, 1H), 1.98 (d, *J* = 2.3 Hz, 3H), 1.37 (d, *J* = 1.5 Hz, 9H).

^{13}**C NMR** (101 MHz, $CDCl_3$) δ 182.46, 171.81, 171.78, 155.64, 80.34, 80.31, 52.80, 52.75, 52.65, 52.22, 43.23, 42.95, 30.03, 28.30, 28.27, 26.84, 26.10, 24.26.

HRMS (ESI) *m/z* Calcd for $C_{13}H_{24}N_2NaO_5S^+$ $[M+Na]^+$: 343.1298, found: 343.1302.

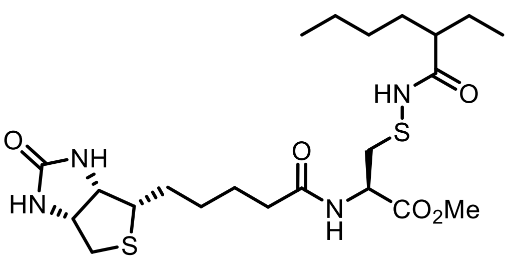

39

Compound **39** is an unknown compound, and was synthesized in 53% yield (53.3 mg, 0.2 mmol scale) under **Conditions [B]**.

White solid (R_f = 0.2, DCM:MeOH = 15:1, v/v)

^{1}H NMR (400 MHz, $CDCl_3$) δ 7.72 (dd, *J* = 8.8, 6.3 Hz, 1H), 7.42 (d, *J* = 3.3 Hz, 1H), 6.86 (brs, 1H), 6.00 (brs, 1H), 4.77 (td, *J* = 9.1, 3.7 Hz, 1H), 4.51 (dd, *J* = 7.9, 4.7 Hz, 1H), 4.31 (dd, *J* = 8.0, 4.6 Hz, 1H), 3.71 (s, 3H), 3.31 (dd, *J* = 14.5, 3.7 Hz, 1H), 3.19 – 3.08 (m, 1H), 2.89 (dd, *J* = 12.8, 4.8 Hz, 1H), 2.78 – 2.61 (m, 2H), 2.33 (t, *J* = 7.5 Hz, 2H), 2.20 – 2.07 (m, 1H), 1.82 – 1.64 (m, 4H), 1.65 – 1.54 (m, 2H), 1.53 – 1.38 (m, 4H), 1.31 – 1.17 (m, 4H), 0.91 – 0.80 (m, 6H).

^{13}C NMR (101 MHz, $CDCl_3$) δ 179.14, 174.62, 172.09, 164.66, 61.89, 60.45, 56.07, 52.84, 52.82, 49.84, 41.33, 40.64, 35.91, 32.57, 32.47, 29.82, 29.79, 28.45, 28.14, 26.16, 25.76, 22.82, 14.06, 14.02, 12.10, 12.03.

HRMS (ESI) *m/z* Calcd for $C_{22}H_{39}N_4O_5S_2^+$ $[M+H]^+$: 503.2356, found: 503.2355.

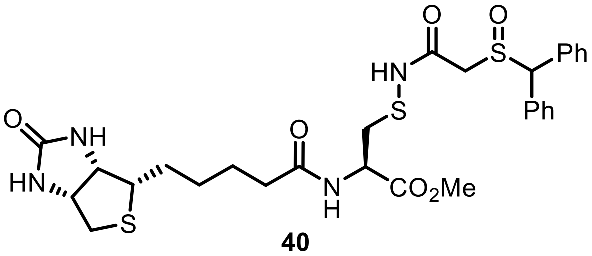

40

Compound **40** is an unknown compound, and was synthesized in 65% yield (82.3 mg, 0.2 mmol scale) under **Conditions [B]**.

White solid (R_f = 0.6, DCM:MeOH = 10:1, v/v)

^{1}H NMR (400 MHz, $CDCl_3$) δ 9.25 (brs, 0.6H), 9.16 (brs, 0.4H), 7.95 (d, *J* = 7.7 Hz, 0.6H), 7.83 (d, *J* = 7.7 Hz, 0.4H), 7.56 – 7.43 (m, 4H), 7.41 – 7.29 (m, 6H), 6.49 (s, 0.4H), 6.42 (s, 0.6H), 5.75 (s, 0.4H), 5.61 (s, 0.6H), 5.51 (s, 0.6H), 5.41 (s, 0.4H), 4.96 – 4.81 (m, 1H), 4.54 – 4.41 (m, 1H), 4.36 – 4.23 (m, 1H), 3.79 – 3.66 (m, 3.6H), 3.54 – 3.25 (m, 2.4H), 3.13 – 3.02 (m, 2H), 2.91 – 2.80 (m, 1H),

2.74 – 2.61 (m, 1H), 2.37 – 2.21 (m, 1H), 2.15 – 1.92 (m, 1H), 1.86 – 1.69 (m, 2H), 1.53 – 1.30 (m, 4H).

^{13}C NMR (101 MHz, $CDCl_3$) δ 174.42, 174.32, 171.79, 171.70, 167.77, 167.67, 164.63, 164.55, 134.83, 134.73, 134.63, 129.69, 129.61, 129.51, 129.47, 129.24, 128.94, 128.88, 128.81, 128.74, 128.54, 72.21, 71.05, 61.48, 61.44, 60.55, 60.52, 55.92, 55.86, 54.30, 52.93, 52.73, 52.70, 52.40, 52.31, 40.64, 40.56, 39.75, 34.03, 33.80, 27.54, 27.40, 27.24, 27.10, 25.34, 25.28.

HRMS (ESI) *m/z* Calcd for $C_{29}H_{36}N_4NaO_6S_3^+$ $[M+Na]^+$: 655.1689, found: 655.1689.

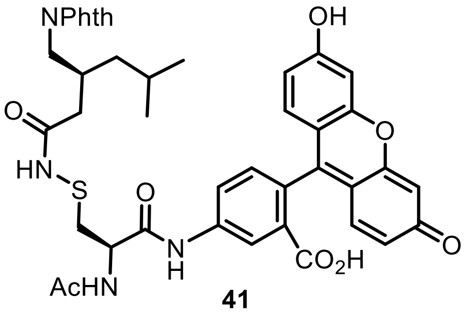

41

Compound **41** is an unknown compound, and was synthesized in 82% yield (62.9 mg, 0.1 mmol scale) under **Conditions [B]**.

Yellow solid (R_f = 0.4, DCM:MeOH = 10:1, v/v)

^{1}H NMR (400 MHz, Methanol-*d*$_4$) δ 8.40 – 8.32 (m, 1H), 7.87 – 7.65 (m, 5H), 7.18 – 7.09 (m, 1H), 6.68 – 6.60 (m, 4H), 6.57 – 6.48 (m, 2H), 4.62 (t, *J* = 6.9 Hz, 0.5H), 4.55 – 4.46 (m, 0.5H), 3.71 – 3.50 (m, 2H), 3.16 – 3.07 (m, 1H), 2.98 – 2.88 (m, 0.5H), 2.80 – 2.71 (m, 0.5H), 2.64 – 2.46 (m, 1H), 2.37 – 2.26 (m, 2H), 2.01 (d, *J* = 2.3 Hz, 3H), 1.79 – 1.68 (m, 1H), 0.96 – 0.84 (m, 6H).

^{13}C NMR (101 MHz, Methanol-*d*$_4$) δ 179.07, 178.72, 173.35, 173.24, 171.42, 170.94, 170.84, 170.24, 170.10, 154.44, 141.21, 141.17, 135.32, 133.25, 133.20, 130.32, 127.88, 126.01, 124.18, 124.16, 114.11, 111.75, 103.58, 54.37, 43.24, 43.15, 42.93, 42.84, 42.47, 40.71, 34.36, 34.13, 30.72, 26.47, 26.44, 23.15, 22.96, 22.54.

HRMS (ESI) *m/z* Calcd for $C_{41}H_{39}N_4O_{10}S^+$ $[M+H]^+$: 779.2381, found: 779.2376.

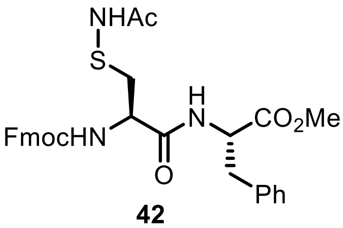

42

Compound **42** is an unknown compound, and was synthesized in 81% yield (91 mg, 0.2 mmol scale) under **Conditions [B]**.

White solid (R_f = 0.58, DCM:MeOH = 20:1, v/v)

^{1}H NMR (400 MHz, DMSO-d_6) δ 8.96 and 8.85 (2×s, 1H), 8.52 (d, J = 8.1 Hz, 1H), 7.89 (d, J = 7.6 Hz, 2H), 7.75 (d, J = 7.5 Hz, 2H), 7.62 (d, J = 8.2 Hz, 1H), 7.42 (t, J = 7.5 Hz, 2H), 7.33 (t, J = 7.4 Hz, 2H), 7.28 – 7.14 (m, 5H), 4.63 – 4.43 (m, 1H), 4.34 – 4.13 (m, 4H), 3.62 and 3.44 (2×s, 3H), 3.09 – 2.99 (m, 1H), 2.96 – 2.85 (m, 1H), 2.80 – 2.70 (m, 1H), 2.70 – 2.57 (m, 1H), 2.07 and 1.96 (2×s, 3H).

^{13}C NMR (101 MHz, DMSO-d_6) δ 172.15, 171.74, 170.33, 155.80, 143.81, 143.79, 140.71, 136.95, 129.19, 128.22, 127.66, 127.08, 126.55, 125.36, 120.10, 65.86, 53.56, 53.41, 51.94, 46.57, 40.71, 36.86, 22.92.

HRMS (ESI) *m/z* Calcd for $C_{30}H_{32}N_3O_6S^+$ [M+H]$^+$: 562.2006, found: 562.2003.

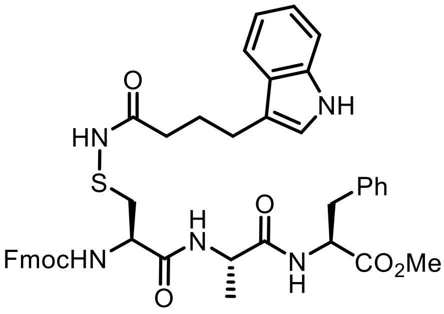

43

Compound **43** is an unknown compound, and was synthesized in 59% yield (91.6 mg, 0.2 mmol scale) under **Conditions [B]**.

White solid (R_f = 0.58, DCM:MeOH = 20:1, v/v)

^{1}H NMR (400 MHz, Methanol-d_4) δ 7.75 (dt, J = 7.7, 0.9 Hz, 2H), 7.61 (d, J = 7.5 Hz, 2H), 7.51 (d, J = 7.9 Hz, 1H), 7.35 (t, J = 7.5 Hz, 2H), 7.31 – 7.23 (m, 3H), 7.21 – 7.08 (m, 5H), 7.08 – 6.98 (m, 2H), 6.94 (t, J = 7.4 Hz, 1H), 4.67 (dd, J = 7.8, 5.9 Hz, 1H), 4.40 – 4.27 (m, 3H), 4.23 (dd, J = 8.6, 5.6 Hz, 1H), 4.15 (t, J = 7.0 Hz, 1H), 3.63 (s, 3H), 3.11 – 2.91 (m, 3H), 2.89 – 2.72 (m, 3H), 2.36 (t, J = 7.4 Hz, 2H), 2.04 (p, J = 7.3 Hz, 2H), 1.33 (d, J = 7.2 Hz, 3H).

^{13}C NMR (101 MHz, $CDCl_3$) δ 178.38, 172.08, 171.78, 169.06, 155.60, 141.42, 136.54, 135.99, 129.63, 128.55, 127.92, 127.51, 127.24, 126.97, 125.25, 122.18, 121.98, 120.17, 119.43, 118.92, 115.07, 111.36, 67.32, 52.95, 52.45, 50.26, 47.17, 42.89, 38.32, 36.18, 25.87, 24.57, 17.73, 14.26.

HRMS (ESI) *m/z* Calcd for $C_{43}H_{46}N_5O_7S^+$ $[M+H]^+$: 776.3112, found: 776.3106.

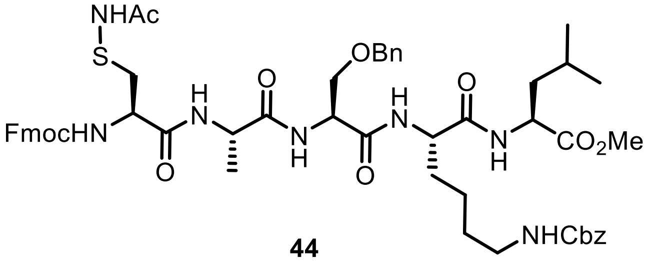

44

Compound **44** is an unknown compound, and was synthesized in 75% yield (98.1 mg, 0.1 mmol scale) under **Conditions [B]**.

White solid (R_f = 0.69, DCM:MeOH = 20:1, v/v)

^{1}H NMR (400 MHz, DMSO-d_6) δ 8.92 (brs, 1H), 8.31 (d, *J* = 6.9 Hz, 1H), 8.21 (d, *J* = 7.6 Hz, 2H), 7.93 (d, *J* = 8.0 Hz, 1H), 7.89 (d, *J* = 7.6 Hz, 2H), 7.78 – 7.60 (m, 3H), 7.41 (t, *J* = 7.5 Hz, 2H), 7.37 – 7.18 (m, 13H), 5.04 and 5.00 (2×s, 2H), 4.58 – 4.51 (m, 1H), 4.46 (s, 2H), 4.39 – 4.13 (m, 7H), 3.65 – 3.52 (m, 5H), 3.00 – 2.87 (m, 3H), 2.81 – 2.75 (m, 1H), 2.07 and 1.92 (2×s, 3H), 1.70 – 1.46 (m, 5H), 1.42 – 1.33 (m, 2H), 1.30 – 1.19 (m, 5H), 0.85 (d, *J* = 6.4 Hz, 3H), 0.78 (d, *J* = 6.3 Hz, 3H).

^{13}C NMR (101 MHz, DMSO-d_6) δ 172.73, 172.51, 172.34, 171.40, 169.93, 168.94, 156.05, 155.80, 143.80, 143.77, 140.71, 138.07, 137.25, 128.34, 128.17, 127.74, 127.44, 127.38, 127.10, 125.41, 125.30, 120.19, 120.06, 72.11, 69.79, 65.85, 65.12, 53.51, 53.43, 52.73, 52.23, 52.11, 51.86, 51.76, 50.25, 50.14, 48.56, 48.42, 46.59, 31.97, 29.19, 24.14, 22.91, 22.73, 22.68, 22.33, 21.22, 21.18, 18.12.

HRMS (ESI) *m/z* Calcd for $C_{54}H_{66}N_7O_{12}S^-$ $[M-H]^-$: 1036.4496, found: 1036.4489.

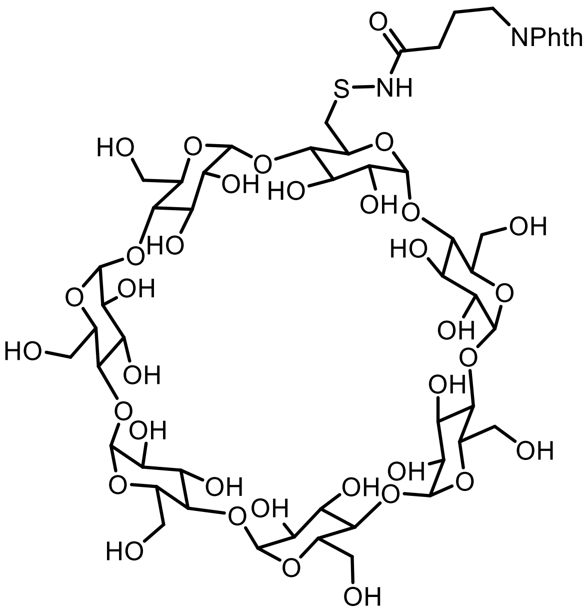

45

Compound **45** is an unknown compound, and was synthesized in 57% yield (78.7 mg, 0.1 mmol scale) under **Conditions [B]** purified by semi preparative HPLC.

White solid

^{1}**H NMR** (400 MHz, DMSO-d_6) δ 8.70 (s, 1H), 7.88 – 7.78 (m, 4H), 5.89 – 5.68 (m, 14H), 4.90 – 4.37 (m, 15H), 3.84 – 3.57 (m, 18H), 3.50 – 3.21 (s, 14H), 3.05 (d, *J* = 13.9 Hz, 1H), 2.93 – 2.85 (m, 1H), 2.15 (t, *J* = 7.4 Hz, 2H), 1.84 – 1.74 (m, 2H).

^{13}**C NMR** (101 MHz, DMSO-d_6) δ 174.16, 167.92, 134.35, 131.63, 123.01, 101.93, 81.50, 73.01, 72.43, 72.03, 59.91, 48.61, 37.00, 32.86, 23.89.

HPLC: t_R = 2.05 min, 5% to 95% of B for 10 min, then 95% B 10-15 min, λ = 254 nm

HRMS (ESI) *m/z* Calcd for $C_{54}H_{79}N_2O_{37}S^-$ $[M-H]^-$: 1379.4088, found: 1379.4091.

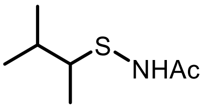

X-1

Compound **X-1** is an unknown compound, and was synthesized in 50% yield (322.6 mg, 4 mmol scale) under **Conditions [C]**.

Colorless oil (R_f = 0.6, PE:EA = 1:1, v/v)

^{1}**H NMR** (400 MHz, $CDCl_3$) δ 6.20 and 6.04 (2×brs, 1H), 3.12 – 3.00 (m, 0.6H), 2.94 – 2.81 (m, 0.4H), 2.23 and 2.09 (2×s, 3H), 1.91 – 1.78 (m, 1H), 1.12 – 1.08 (m, 3H), 1.03 – 0.92 (m, 6H).

^{13}C NMR (101 MHz, $CDCl_3$) δ 177.01, 171.29, 142.69, 140.40, 134.81, 130.09, 129.73, 128.95, 128.48, 23.34, 21.87, 21.47, 20.10.

HRMS (ESI) *m/z* Calcd for $C_7H_{16}NOS^+$ $[M+H]^+$: 162.0947, found: 162.0948.

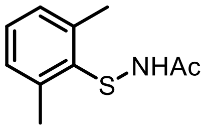

X-2

Compound **X-2** is an unknown compound, and was synthesized in 13% yield (39 mg, 1.5 mmol scale) under **Conditions [B]**.

White solid (R_f = 0.42, PE:EA = 2:1, v/v)

^{1}H NMR (400 MHz, $CDCl_3$) δ 7.21 – 7.13 (m, 1H), 7.13 – 7.04 (m, 2H), 6.64 and 6.40 (2×brs, 1H), 2.65 and 2.56 (2×s, 6H), 2.19 and 2.00 (2×s, 3H).

^{13}C NMR (101 MHz, $CDCl_3$) δ 177.01, 171.29, 142.69, 140.40, 134.81, 130.09, 129.73, 128.95, 128.48, 23.34, 21.87, 21.47, 20.10.

HRMS (ESI) *m/z* Calcd for $C_{10}H_{14}NOS^+$ $[M+H]^+$: 196.0791, found: 196.0791.

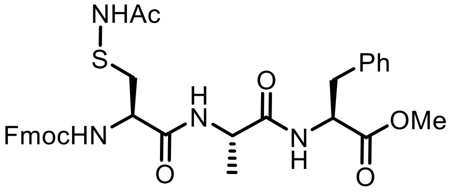

X-3

Compound **X-3** is an unknown compound, and was synthesized in 70% yield (88.6 mg, 0.2 mmol scale) under **Conditions [B]**.

White solid (R_f = 0.44, DCM:MeOH = 20:1, v/v)

^{1}H NMR (400 MHz, $CDCl_3$) δ 8.84 (d, *J* = 7.3 Hz, 1H), 7.75 (d, *J* = 7.5 Hz, 2H), 7.61 – 7.52 (m, 2H), 7.39 (t, *J* = 7.5 Hz, 2H), 7.30 (d, *J* = 7.5 Hz, 2H), 7.23 – 7.11 (m, 5H), 7.10 – 7.01 (m, 2H), 6.18 (d, *J* = 7.2 Hz, 1H), 4.99 – 4.81 (m, 1H), 4.48 (p, *J* = 7.3 Hz, 1H), 4.38 – 4.25 (m, 2H), 4.23 – 4.10 (m, 2H), 3.68 (s, 3H), 3.23 (d, *J* = 14.6, 1H), 3.13 – 3.01 (m, 2H), 2.53 (dd, *J* = 14.5, 10.7 Hz, 1H), 2.14 (s, 3H), 1.48 (d, *J* = 7.2 Hz, 3H).

^{13}C NMR (101 MHz, $CDCl_3$) δ 175.46, 172.00, 171.84, 169.05, 155.64, 143.82, 143.66, 141.37, 135.91, 129.58, 128.51, 127.90, 127.20, 126.94, 125.20, 125.16, 120.14, 67.32, 52.93, 52.43, 50.23, 47.10, 42.74, 38.24, 23.38, 17.77.

HRMS (ESI) *m/z* Calcd for $C_{33}H_{36}N_4NaO_7S^+$ $[M+Na]^+$: 655.2197, found: 655.2203.

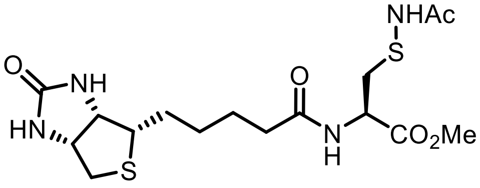

X-4

Compound **X-4** is an unknown compound, and was synthesized in 50% yield (41.9 mg, 0.2 mmol scale) under **Conditions [B]**.

White solid (R_f = 0.15, DCM:MeOH = 15:1, v/v)

^{1}H NMR (400 MHz, $CDCl_3$) δ 7.90 and 7.83 (2×brs, 1H), 7.76 and 7.65 (2×d, J = 7.9 Hz and 8.4 Hz, 1H), 7.09 and 6.78 (2×brs, 1H), 6.23 and 5.98 (2×brs, 1H), 5.03 – 4.73 (2×m, 1H), 4.60 – 4.47 (m, 1H), 4.40 – 4.26 (m, 1H), 3.72 (s, 3H), 3.28 – 2.99 (m, 3H), 2.96 – 2.84 (m, 1H), 2.80 – 2.67 (m, 1H), 2.37 – 2.27 (m, 3H), 2.23 and 2.07 (2×s, 3H), 1.83 – 1.61 (m, 4H), 1.55 – 1.37 (m, 2H).

^{13}C NMR (101 MHz, $CDCl_3$) δ 174.27, 173.57, 172.12, 61.84, 60.54, 56.00, 52.92, 50.16, 40.65, 40.55, 35.64, 28.13, 28.05, 25.67, 23.41.

HRMS (ESI) *m/z* Calcd for $C_{16}H_{27}N_4O_5S_2^+$ $[M+H]^+$: 419.1417, found: 419.1413.

1.5 General procedures for the S–S coupling

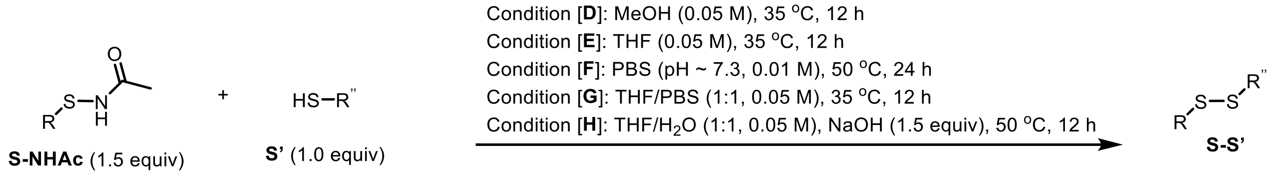

Supplementary Figure 14. General procedures for the S–S coupling

Condition [**D**]: General procedures for the synthesis of **46**. Sulfenamide **5a** (87.6 mg, 0.3 mmol, 1.5 equiv) and *p*-toluenethiol (24.8 mg, 0.2 mmol, 1.0 equiv) were dissolved in MeOH (4.0 mL). The

reaction mixture was stirred at 35°C under air for 12 hours. The reaction mixture was concentrated *in vacuo*. The resulting residue was purified by silica gel flash chromatography using hexanes/EtOAc eluent (5/1 to 1.5:1) to give the desired product **46** as a white solid (63.5 mg, 89% yield).

Condition [E] is same as condition **[D]** except for using THF as solvent.

Condition [F] is same as condition **[D]** except for using PBS (pH 7.3, 0.01 M) as solvent and react at 50°C under air for 24 h. (Note: the substrates **50**, **54**, **55**, **57** and **58** were purified by semi-preparative HPLC.)

Condition [G] is same as condition **[D]** except for using THF/PBS = 1:1 (pH 7.3, 0.05 M) as solvent.

Condition [H]: General procedures for the synthesis of **64**. To the solution of sulfenamide **X-1** (48.4 mg, 0.3 mmol, 1.5 equiv) and 1-adamantanethiol (33.7 mg, 0.2 mmol, 1.0 equiv) in THF (2.0 mL), aqueous solution of NaOH (12.0 mg, 0.3 mmol, 1.5 equiv in 2.0 mL of water) were added. The reaction mixture was stirred at 50°C under air for 12 hours. The reaction mixture was concentrated *in vacuo*. The resulting residue was purified by silica gel flash chromatography using hexanes/EtOAc eluent (20/1 to 5:1) to give the desired product **64** as a colorless oil (51.3 mg, 95% yield).

Condition [H'] is same as condition **[H]** except a 1:2 ratio of sulfenamide (**S-NHAc**) and thiol (**S')**.

(**NOTE**: Disulfides compounds are visualized at 220 or 254 nm of UV)

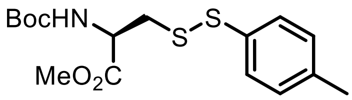

46

Compound **46** is an unknown compound, and was synthesized in 89% yield (63.5 mg, 0.2 mmol scale) using **5a** as sulfenamide reagent under **Condition [D]**.

White solid (R_f = 0.5, PE:EA = 5:1, v/v)

^{1}H NMR (400 MHz, $CDCl_3$) δ 7.42 (d, *J* = 8.2 Hz, 2H), 7.15 (d, *J* = 7.9 Hz, 2H), 5.30 (d, *J* = 8.1 Hz, 1H), 4.71 – 4.52 (m, 1H), 3.74 (s, 3H), 3.26 – 3.07 (m, 2H), 2.34 (s, 3H), 1.44 (s, 9H).

^{13}C NMR (101 MHz, $CDCl_3$) δ 171.29, 138.01, 130.07, 129.54, 52.74, 40.84, 28.42, 21.23.

HRMS (ESI) *m/z* Calcd for $C_{16}H_{22}NO_4S_2^-$ [M-H]$^-$: 356.0996, found: 356.0999.

HPLC: The ee value of **46** (>99.9% ee) was determined by HPLC analysis on a CHIRALPAK® AY-H column (hexanes: isopropanol = 80:20, 1.0 mL/min, T = 30 °C, 254 nm), TR = 5.45 min (minor), 11.50 min (major).

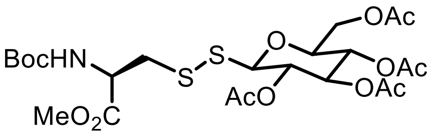

47

Compound **47** is an unknown compound, and was synthesized in 86% yield (102.8 mg, 0.2 mmol scale) using **5a** as sulfenamide reagent under **Condition [D]**.

White solid (R_f = 0.4, PE:EA = 2:1, v/v)

^{1}H NMR (400 MHz, $CDCl_3$) δ 5.33 (d, *J* = 8.6 Hz, 1H), 5.28 – 5.18 (m, 2H), 5.15 – 5.05 (m, 1H), 4.70 – 4.59 (m, 1H), 4.59 – 4.50 (m, 1H), 4.28 – 4.19 (m, 1H), 4.17 – 4.08 (m, 1H), 3.81 – 3.75 (m, 1H), 3.73 (s, 3H), 3.28 (dd, *J* = 13.8, 4.7 Hz, 1H), 3.02 (dd, *J* = 13.9, 7.8 Hz, 1H), 2.05 (s, 3H), 2.02 – 1.94 (m, 9H), 1.42 (s, 9H).

^{13}C NMR (101 MHz, $CDCl_3$) δ 171.45, 170.66, 170.22, 169.44, 169.20, 155.13, 87.92, 80.28, 76.20, 73.87, 68.97, 67.92, 62.02, 52.98, 52.66, 42.66, 28.36, 20.70, 20.66, 20.64.

HRMS (ESI) *m/z* Calcd for $C_{23}H_{36}NO_{13}S_2^+$ $[M+H]^+$: 598.1623, found: 598.1620.

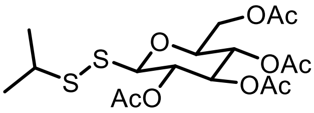

48

Compound **48** is an unknown compound, and was synthesized in 92% yield (80.7 mg, 0.2 mmol scale) using **3a** as sulfenamide reagent under **Condition [G]**.

White solid (R_f = 0.74, DCM:MeOH = 20:1, v/v)

^{1}H NMR (400 MHz, Methanol-d_4) δ 4.50 – 4.35 (m, 1H), 4.05 – 3.82 (m, 2H), 3.73 (s, 3H), 3.67 – 3.56 (m, 1H), 3.25 – 3.14 (m, 1H), 3.05 – 2.84 (m, 1H).

^{13}C NMR (101 MHz, Methanol-d_4) δ 174.83, 174.75, 173.17, 173.13, 173.03, 80.85, 54.28, 52.94, 52.92, 42.42, 41.10, 40.74, 28.68, 17.22, 17.14.

HRMS (ESI) *m/z* Calcd for $C_{17}H_{27}O_9S_2^+$ $[M+H]^+$: 439.1091, found: 439.1086.

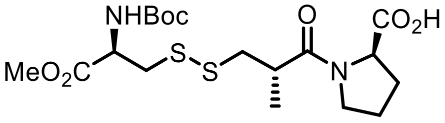

49

Compound **49** is an unknown compound, and was synthesized in 78% yield (70.2 mg, 0.2 mmol scale) using **5a** as sulfenamide reagent under **Condition [F]**.

White solid (R_f = 0.2, DCM:MeOH = 15:1, v/v)

^{1}H NMR (400 MHz, Methanol-d_4) δ 4.49 – 3.36 (m, 2H), 3.78 – 3.66 (m, 5H), 3.20 – 3.07 (m, 2H), 3.03 – 2.87 (m, 2H), 2.76 (dd, J = 13.3, 5.2 Hz, 0H), 2.31 – 2.20 (m, 1H), 2.08 – 1.97 (m, 2H), 1.44 (s, 9H), 1.20 (dd, J = 6.9, 2.4 Hz, 3H).

^{13}C NMR (101 MHz, Methanol-d_4) δ 175.80, 173.14, 173.10, 157.80, 80.83, 60.29, 54.35, 52.94, 47.71, 42.84, 41.10, 39.25, 30.25, 28.70, 25.66, 23.41, 17.12.

HPLC: t_R = 8..47 min, 5% to 95% of B for 10 min, then 95% B 10-15 min, λ = 254 nm

HRMS (ESI) *m/z* Calcd for $C_{18}H_{31}N_2O_7S_2^+$ [M+H]$^+$: 451.1567, found: 451.1563.

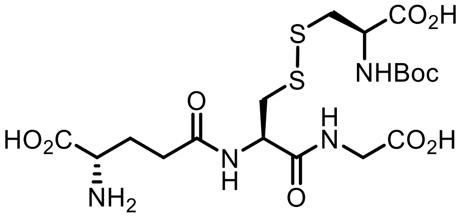

50

Compound **50** is an unknown compound, and was synthesized in 66% yield (69.5 mg, 0.2 mmol scale) using **5a** as sulfenamide reagent under **Condition [F]**.

White solid

^{1}H NMR (400 MHz, DMSO-d_6) δ 8.60 (s, 2H), 7.39 (d, J = 8.1 Hz, 1H), 4.60 – 4.39 (m, 1H), 4.34 – 4.19 (m, 1H), 3.74 – 3.54 (m, 5H), 3.35 – 3.25 (m, 1H), 3.20 – 3.10 (m, 1H), 3.10 – 3.00 (m, 1H), 2.95 – 2.77 (m, 2H), 2.41 – 2.25 (m, 2H), 2.03 – 1.89 (m, 1H), 1.88 – 1.73 (m, 1H), 1.37 (s, 9H).

^{13}C NMR (101 MHz, DMSO-d_6) δ 172.05, 171.59, 171.52, 170.36, 155.41, 78.64, 53.09, 52.77, 52.23, 52.06, 41.83, 31.38, 28.22, 28.00, 26.77.

HPLC: t_R = 5.30 min, 5% to 95% of B for 10 min, then 95% B 10-15 min, λ = 254 nm

HRMS (ESI) *m/z* Calcd for $C_{19}H_{33}N_4O_{10}S_2^+$ [M+H]$^+$: 541.1633, found: 541.1631.

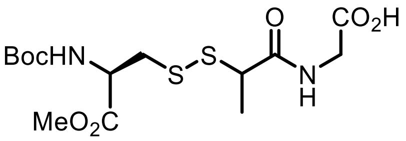

51

Compound **51** is an unknown compound, and was synthesized in 69% yield (54.6 mg, 0.2 mmol scale) using **5a** as sulfenamide reagent under **Condition [G]**.

White solid (R_f = 0.15, DCM:MeOH = 5:1, v/v)

^{1}H NMR (400 MHz, Methanol-d_4) δ 4.49 – 3.38 (m, 1H), 4.04 – 3.83 (m, 2H), 3.73 (s, 3H), 3.68 – 3.56 (m, 1H), 3.25 – 3.14 (m, 1H), 3.05 – 2.83 (m, 1H), 1.49 – 1.40 (m, 12H).

^{13}C NMR (101 MHz, Methanol-d_4) δ 174.83, 174.75, 173.17, 173.13, 173.03, 80.85, 54.28, 52.94, 52.92, 42.42, 41.10, 40.74, 28.68, 17.22, 17.14.

HPLC: t_R = 5.54 min, 5% to 95% of B for 10 min, then 95% B 10-15 min, λ = 254 nm

HRMS (ESI) *m/z* Calcd for $C_{14}H_{23}N_2O_7S_2^-$ [M-H]$^-$: 395.0952, found: 395.0955.

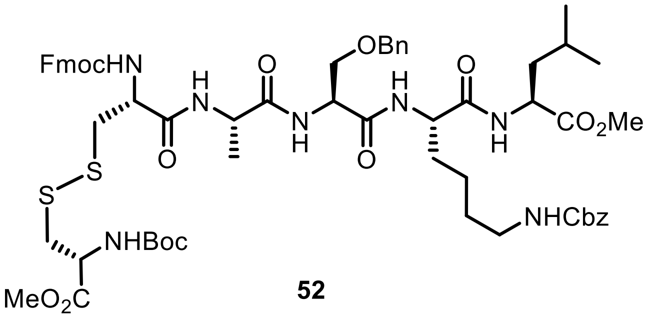

52

Compound **52** is an unknown compound, and was synthesized in 87% yield (105.6 mg, 0.1 mmol scale) using **5a** as sulfenamide reagent under **Condition [G]**.

White solid (R_f = 0.74, DCM:MeOH = 20:1, v/v)

^{1}H NMR (400 MHz, DMSO-d_6) δ 8.26 – 8.11 (m, 1H), 7.94 – 7.84 (m, 3H), 7.79 – 7.66 (m, 3H), 7.45 – 7.18 (m, 16H), 4.99 (s, 2H), 4.57 – 4.49 (m, 1H), 4.47 (s, 2H), 4.40 – 4.16 (m, 8H), 3.65 – 3.54 (m, 8H), 3.16 – 3.04 (m, 2H), 2.99 – 2.80 (m, 4H), 1.71 – 1.45 (m, 5H), 1.40 – 1.30 (m, 11H), 1.29 – 1.20 (m, 5H), 0.85 (d, *J* = 6.5 Hz, 3H), 0.79 (d, *J* = 6.4 Hz, 3H).

^{13}C NMR (101 MHz, DMSO-d_6) δ 172.69, 172.04, 171.48, 171.37, 169.74, 168.93, 156.02, 155.95, 155.28, 143.73, 140.67, 138.05, 137.23, 129.17, 128.31, 128.13, 127.71, 127.62, 127.41, 127.35, 127.07, 125.29, 121.36, 120.08, 78.48, 72.10, 69.75, 65.09, 52.63, 52.05, 51.76, 50.18, 48.22, 46.58, 31.98, 29.16, 28.07, 24.12, 22.67, 22.25, 21.19, 18.17.

HRMS (ESI) *m/z* Calcd for $C_{61}H_{78}N_7O_{15}S_2^-$ [M-H]$^-$: 1212.5003, found: 1212.4990.

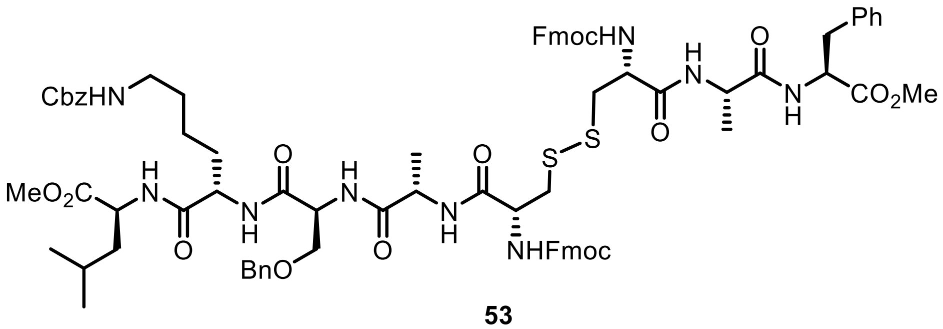

53

Compound **53** is an unknown compound, and was synthesized in 56% yield (87 mg, 0.1 mmol scale) using **X-3** as sulfenamide reagent under **Condition [G]**.

White solid (R_f = 0.74, DCM:MeOH = 20:1, v/v)

^{1}H NMR (400 MHz, DMSO-d_6) δ 8.33 (d, *J* = 7.5 Hz, 1H), 8.22 (t, *J* = 8.4 Hz, 2H), 8.13 (d, *J* = 7.4 Hz, 1H), 8.06 (d, *J* = 7.6 Hz, 1H), 7.93 – 7.83 (m, 5H), 7.74 (t, *J* = 7.3 Hz, 2H), 7.71 – 7.64 (t, *J* = 5.3 Hz, 4H), 7.42 – 7.15 (m, 24H), 4.99 (s, 2H), 4.53 (q, *J* = 6.2 Hz, 1H), 4.48 – 4.17 (m, 15H), 3.60 – 3.50 (m, 8H), 3.17 – 3.06 (m, 2H), 3.04 – 2.82 (m, 6H), 1.69 – 1.45 (m, 5H), 1.42 – 1.33 (m, 2H), 1.29 – 1.14 (m, 8H), 0.84 (d, *J* = 6.5 Hz, 3H), 0.77 (d, *J* = 6.4 Hz, 3H).

^{13}C NMR (101 MHz, DMSO-d_6) δ 172.69, 172.05, 171.69, 171.36, 169.73, 169.62, 168.93, 156.02, 155.98, 143.72, 143.70, 140.66, 138.04, 137.23, 136.92, 129.02, 128.31, 128.22, 128.12, 127.71, 127.60, 127.41, 127.34, 127.06, 126.54, 125.29, 125.25, 120.07, 72.10, 69.77, 65.84, 65.09, 53.79, 53.61, 52.72, 52.09, 51.78, 50.17, 48.18, 47.99, 46.55, 36.55, 32.02, 29.16, 24.12, 22.66, 22.23, 21.18, 18.27, 18.17.

HRMS (ESI) *m/z* Calcd for $C_{83}H_{94}N_9O_{17}S_2^-$ [M-H]$^-$: 1552.6215, found: 1552.6216.

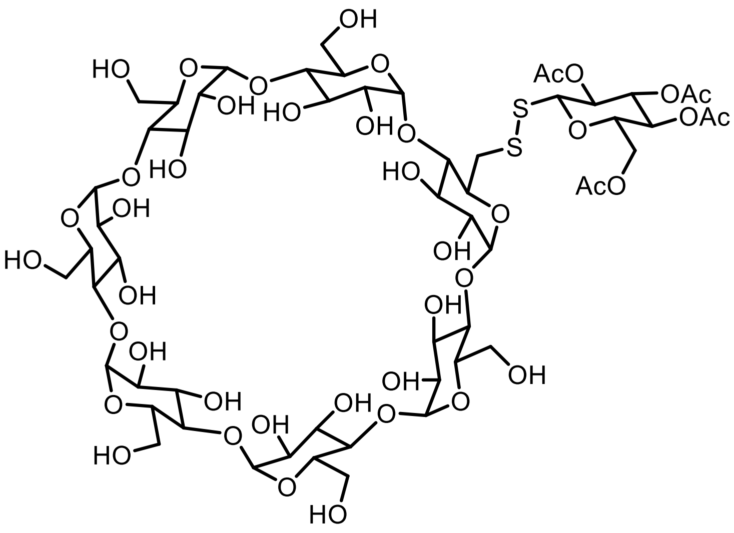

54

Compound **54** is an unknown compound, and was synthesized in 48% yield (72.6 mg, 0.1 mmol scale) using **7** as sulfenamide reagent under **Condition [F]**.

White solid

^{1}H NMR (400 MHz, Methanol-d_4) δ 6.27 – 5.82 (m, 1H), 5.35 – 5.25 (m, 1H), 5.25 – 5.16 (m, 1H), 5.06 – 4.89 (m, 8H), 4.76 (d, J = 10.9 Hz, 1H), 4.40 – 4.27 (m, 1H), 4.05 (d, J = 11.6 Hz, 2H), 3.97 – 3.64 (m, 25H), 3.61 – 3.54 (m, 2H), 3.54 – 3.35 (m, 12H), 3.28 – 3.25 (m, 2H), 2.06 (s, 3H), 2.02 – 1.93 (m, 9H).

^{13}C NMR (101 MHz, Methanol-d_4) δ 172.12, 171.62, 171.10, 104.29, 103.81, 103.57, 103.47, 87.70, 87.04, 83.13, 83.02, 82.74, 81.79, 77.04, 75.23, 74.85, 74.74, 74.62, 74.29, 74.13, 73.58, 73.52, 73.42, 72.81, 70.69, 69.27, 63.06, 61.94, 61.84, 61.43, 45.94, 20.95, 20.72, 20.70, 20.60.

HPLC: t_R = 3.65 min, 5% to 95% of B for 10 min, then 95% B 10-15 min, λ = 254 nm

HRMS (ESI) *m/z* Calcd for $C_{56}H_{88}NaO_{43}S_2^+$ [M+Na]$^+$: 1535.4033, found: 1535.4032.

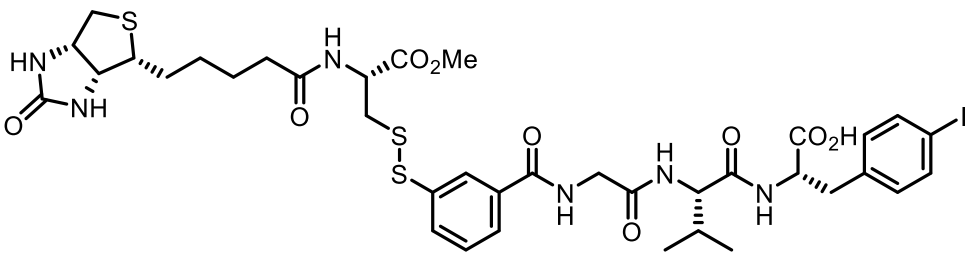

55

Compound **55** is an unknown compound, and was synthesized in 59% yield (55.6 mg, 0.1 mmol scale) using **X-4** as sulfenamide reagent under **Condition [F]**.

White solid (R_f = 0.38, DCM:MeOH = 5:1, v/v)

^{1}H NMR (400 MHz, DMSO-d_6) δ 12.74 (brs, 1H), 8.85 (t, J = 6.0 Hz, 1H), 8.44 (d, J = 7.7 Hz, 1H), 8.28 (d, J = 7.7 Hz, 1H), 8.01 (s, 1H), 7.85 (d, J = 9.0 Hz, 1H), 7.78 (d, J = 7.8 Hz, 1H), 7.71 (d, J = 6.8 Hz, 1H), 7.60 (d, J = 7.9 Hz, 2H), 7.51 (t, J = 7.8 Hz, 1H), 7.04 (d, J = 7.9 Hz, 2H), 6.39 (d, J = 22.6 Hz, 2H), 4.61 – 4.51 (m, 1H), 4.43 – 4.33 (m, 1H), 4.33 – 4.26 (m, 1H), 4.26 – 4.18 (m, 1H), 4.16 – 4.08 (m, 1H), 3.93 (d, J = 6.0 Hz, 2H), 3.61 (s, 3H), 3.18 – 2.94 (m, 4H), 2.91 – 2.76 (m, 2H), 2.57 (d, J = 12.4 Hz, 1H), 2.13 (t, J = 7.3 Hz, 2H), 1.94 (h, J = 6.7 Hz, 1H), 1.66 – 1.56 (m, 1H), 1.56 – 1.41 (m, 3H), 1.37 – 1.26 (m, 2H), 0.83 (d, J = 6.7 Hz, 3H), 0.78 (d, J = 6.8 Hz, 3H).

^{13}C NMR (101 MHz, DMSO-d_6) δ 172.52, 172.42, 170.88, 170.83, 168.56, 165.67, 162.69, 137.42, 136.85, 136.44, 135.12, 131.59, 129.93, 129.46, 126.18, 126.05, 92.31, 79.17, 61.02, 59.17, 57.16, 55.42, 53.26, 52.21, 51.03, 42.50, 36.04, 34.75, 30.76, 28.03, 28.00, 25.10, 19.14, 17.83.

HPLC: t_R = 8.61 min, 5% to 95% of B for 10 min, then 95% B 10-15 min, λ = 254 nm

HRMS (ESI) *m/z* Calcd for $C_{37}H_{47}IN_6NaO_9S_3^+$ $[M+Na]^+$: 965.1504, found: 965.1492.

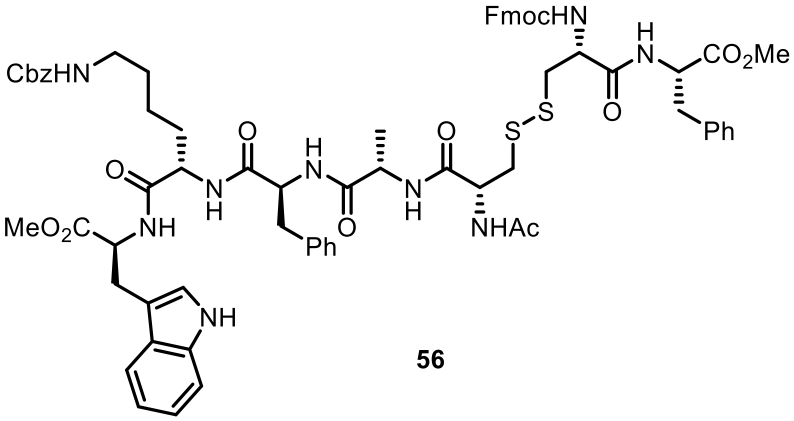

56

Compound **56** is an unknown compound, and was synthesized in 80% yield (106.7 mg, 0.1 mmol scale) using **42** as sulfenamide reagent under **Condition [G]**.

White solid (R_f = 0.5, DCM:MeOH = 15:1, v/v)

^{1}H NMR (400 MHz, DMSO-d_6) δ 10.87 (s, 1H), 8.56 (d, J = 8.0 Hz, 1H), 8.35 (d, J = 7.2 Hz, 1H), 8.27 (d, J = 8.4 Hz, 1H), 8.13 – 8.02 (m, 2H), 7.97 (d, J = 8.1 Hz, 1H), 7.93 (d, J = 7.9 Hz, 1H), 7.88 (d, J = 7.6 Hz, 2H), 7.72 (t, J = 7.7 Hz, 2H), 7.62 (d, J = 8.8 Hz, 1H), 7.49 (d, J = 7.8 Hz, 1H), 7.44 – 7.38 (m, 2H), 7.37 – 7.27 (m, 8H), 7.26 – 7.14 (m, 10H), 7.14 – 7.09 (m, 1H), 7.06 (t, J = 7.6 Hz, 1H), 7.02 – 6.95 (m, 1H), 5.00 (s, 2H), 4.60 – 4.46 (m, 4H), 4.35 – 4.16 (m, 6H), 3.61 (s, 3H), 3.53 (s, 3H),

3.19 – 3.03 (m, 4H), 3.03 – 2.74 (m, 7H), 2.70 – 2.60 (m, 1H), 1.88 (s, 3H), 1.68 – 1.57 (m, 1H), 1.50 (d, J = 8.7 Hz, 1H), 1.39 (p, J = 7.3 Hz, 2H), 1.30 – 1.20 (m, 2H), 1.16 (d, J = 7.0 Hz, 3H).

^{13}C NMR (101 MHz, DMSO-d_6) δ 172.13, 171.76, 171.69, 171.56, 170.54, 170.07, 169.85, 169.56, 156.09, 155.92, 143.82, 143.76, 140.72, 140.70, 137.62, 137.26, 137.02, 136.09, 129.22, 129.20, 128.36, 128.33, 128.22, 127.98, 127.75, 127.06, 126.58, 126.19, 125.38, 123.75, 120.99, 120.20, 120.03, 118.45, 118.03, 111.45, 109.20, 65.89, 65.15, 53.52, 53.04, 52.25, 52.02, 51.87, 51.71, 48.35, 48.22, 46.57, 40.87, 37.24, 36.82, 32.08, 29.26, 27.01, 22.60, 22.42, 18.17, 18.06.

HRMS (ESI) *m/z* Calcd for $C_{71}H_{80}N_9O_{14}S_2^+$ [M+H]$^+$: 1346.5261, found: 1346.5266.

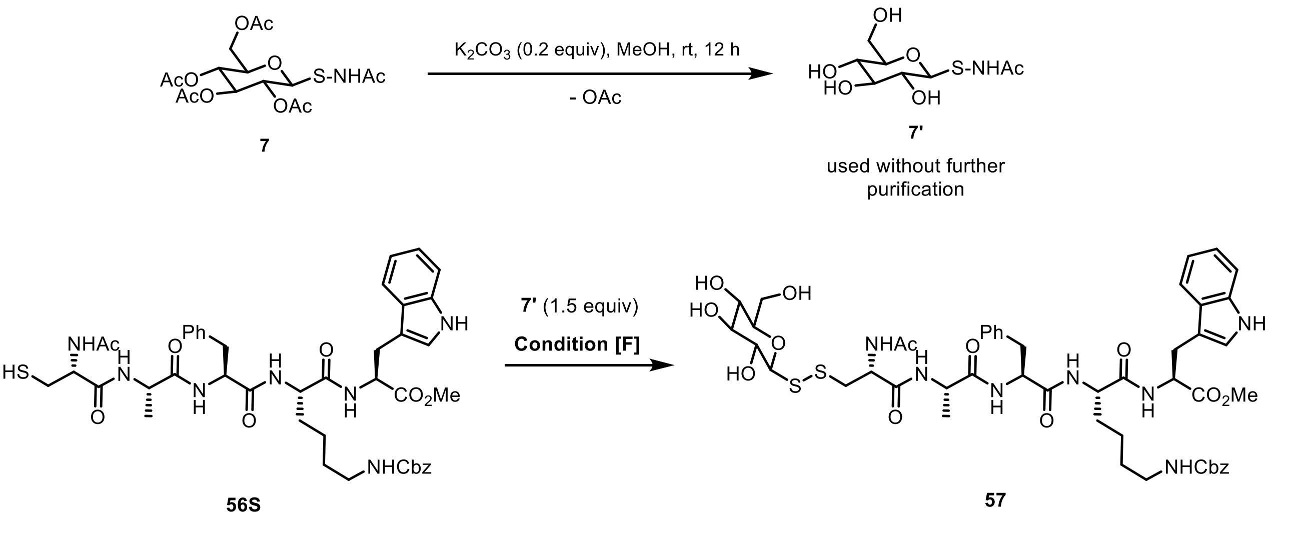

Supplementary Figure 15. The preparation of substrate 57

Preparation of **57**:

7 (0.15 mmol, 63.2 mg) was dissolved in MeOH (1.5 mL) and K_2CO_3 (20 mol%, 4.1 mg) was added to the system. The mixture was stirred for 12 h at room temperature under air. Solvent was removed under reduced pressure and gave the crude product **7'** without further purification.

7' was dissolved in PBS buffer (pH~7.3, 10 mL) and mercapto substrate (0.1 mmol, 84.4 mg) was added to the system. The reaction mixture was stirred for 24 h at 50 °C under air. Water was removed under reduced pressure. The crude product was redissolved in MeCN/H_2O/MeOH, filtrated and purified by Semi preparative HPLC to give the desired product **57** in 33% isolated yield (34.3 mg).

White solid

^{1}H NMR (400 MHz, DMSO-*d*$_6$) δ 10.88 (s, 1H), 8.35 (d, *J* = 7.2 Hz, 1H), 8.21 (d, *J* = 8.1 Hz, 1H), 8.03 – 7.83 (m, 3H), 7.48 (d, *J* = 7.8 Hz, 1H), 7.40 – 7.27 (m, 6H), 7.26 – 7.11 (m, 7H), 7.06 (t, *J* = 7.5 Hz, 1H), 6.99 (t, *J* = 7.4 Hz, 1H), 5.37 – 4.91 (m, 5H), 4.69 (s, 1H), 4.68 – 4.54 (m, 1H), 4.54 – 4.45 (m, 2H), 4.35 – 4.25 (m, 2H), 4.25 – 4.15 (m, 1H), 3.71 (d, *J* = 11.6 Hz, 1H), 3.53 (s, 3H), 3.50 – 3.37 (m, 2H), 3.27 – 3.04 (m, 7H), 3.03 – 2.92 (m, 3H), 2.88 (dd, *J* = 13.2, 9.7 Hz, 1H), 2.78 (dd, *J* = 14.0, 9.2 Hz, 1H), 1.86 (s, 3H), 1.62 (s, 1H), 1.56 – 1.44 (m, 1H), 1.44 – 1.34 (m, 2H), 1.31 – 1.24 (m, 2H), 1.14 (d, *J* = 7.0 Hz, 3H).

^{13}C NMR (101 MHz, DMSO-*d*$_6$) δ 172.14, 171.79, 171.57, 170.53, 169.94, 169.72, 156.10, 137.62, 137.27, 136.10, 129.22, 128.37, 128.02, 127.78, 127.07, 126.23, 123.76, 120.99, 118.45, 117.97, 111.47, 109.21, 90.44, 81.19, 77.94, 71.44, 69.66, 65.16, 61.09, 53.69, 53.15, 52.22, 52.13, 51.78, 48.32, 41.74, 37.26, 32.03, 29.26, 27.00, 22.59, 22.43, 18.06.

HPLC: t_R = 8.33 min, 5% to 95% of B for 10 min, then 95% B 10-15 min, λ = 254 nm

HRMS (ESI) *m/z* Calcd for $C_{49}H_{63}N_7NaO_{14}S_2^+$ $[M+Na]^+$: 1060.3767, found: 1060.3768.

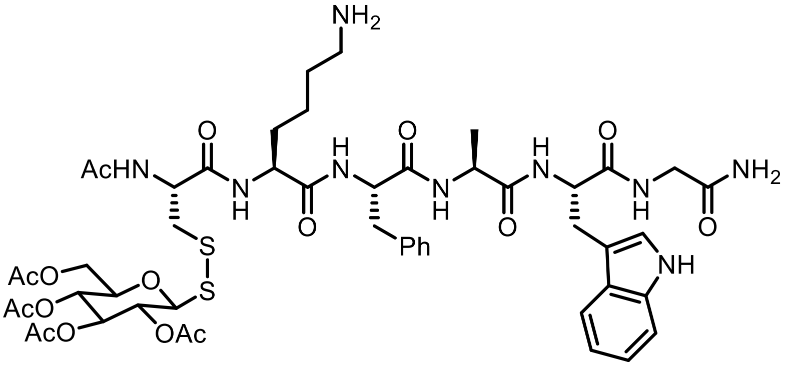

58

Compound **58** is an unknown compound, and was synthesized in 40% yield (44.6 mg, 0.1 mmol scale) using **7** as sulfenamide reagent under **Condition [F]**.

White solid

^{1}H NMR (400 MHz, Methanol-*d*$_4$) δ 8.52 (s, 1H), 7.56 (d, *J* = 7.6 Hz, 1H), 7.30 (d, *J* = 7.9 Hz, 1H), 7.25 – 7.12 (m, 6H), 7.07 (t, *J* = 7.5 Hz, 1H), 7.01 (t, *J* = 7.4 Hz, 1H), 5.35 – 5.18 (m, 2H), 5.04 (t, *J* = 9.5 Hz, 1H), 4.72 (dd, *J* = 9.3, 2.9 Hz, 1H), 4.63 – 4.51 (m, 3H), 4.30 – 4.09 (m, 4H), 3.88 – 3.78 (m, 2H), 3.69 (d, *J* = 16.8 Hz, 1H), 3.37 – 3.31 (m, 1H), 3.26 – 3.17 (m, 2H), 3.10 – 2.98 (m, 2H), 2.88 – 2.75 (m, 3H), 2.05 – 1.89 (m, 15H), 1.72 – 1.62 (m, 1H), 1.60 – 1.49 (m, 3H), 1.33 – 1.27 (m, 5H).

^{13}C NMR (101 MHz, Methanol-d_4) δ 174.93, 174.32, 173.81, 173.73, 173.69, 172.89, 172.39, 171.52, 171.20, 170.95, 138.26, 137.95, 130.33, 130.29, 129.52, 128.67, 127.86, 124.80, 122.55, 119.95, 119.31, 112.48, 110.79, 88.21, 76.96, 75.05, 70.40, 69.34, 63.10, 56.19, 54.92, 54.62, 51.03, 43.38, 42.00, 40.34, 38.26, 31.96, 31.29, 30.69, 28.22, 27.91, 23.36, 22.59, 20.83, 20.64, 20.56, 17.66.

HRMS (ESI) *m/z* Calcd for $C_{50}H_{66}N_9O_{16}S_2^-$ [M-H]$^-$: 1112.4074, found: 1112.4070.

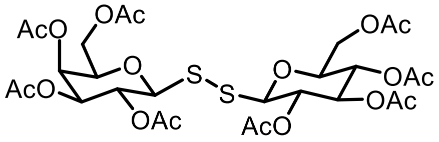

59

Compound **59** is an unknown compound, and was synthesized in 95% yield (69 mg, 0.1 mmol scale) under using **7** as sulfenamide reagent under **Condition [F]**.

White solid (R_f = 0.4, PE:EA = 1:1, v/v)

^{1}H NMR (400 MHz, Methanol-d_4) δ 5.48 – 5.40 (m, 1H), 5.38 – 5.29 (m, 2H), 5.27 – 5.14 (m, 2H), 5.09 – 5.00 (m, 1H), 4.95 (d, *J* = 9.6 Hz, 1H), 4.82 – 4.79 (s, 1H), 4.45 (dd, *J* = 12.6, 5.0 Hz, 1H), 4.25 – 4.11 (m, 4H), 4.00 – 390 (m, 1H), 2.22 – 1.93 (m, 24H).

^{13}C NMR (101 MHz, Methanol-d_4) δ 172.34, 172.04, 171.96, 171.47, 171.37, 171.21, 171.15, 170.82, 90.33, 86.54, 77.32, 75.86, 75.17, 73.11, 70.18, 69.29, 68.93, 68.82, 62.85, 62.61, 20.95, 20.91, 20.69, 20.65, 20.62, 20.55.

HRMS (ESI) *m/z* Calcd for $C_{28}H_{38}NaO_{18}S_2^+$ [M+Na]$^+$: 749.1392, found: 749.1389.

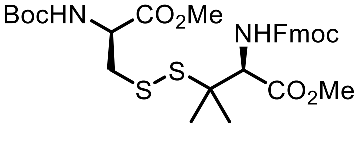

60

Compound **60** is an unknown compound, and was synthesized in 80% yield (99 mg, 0.2 mmol scale) using **5a** as sulfenamide reagent under **Condition [G]**.

White solid (R_f = 0.54, PE:EA = 2:1, v/v)

^{1}H NMR (400 MHz, $CDCl_3$) δ 7.76 (d, *J* = 7.5 Hz, 2H), 7.60 (d, *J* = 7.0 Hz, 2H), 7.40 (t, *J* = 7.5 Hz, 2H), 7.35 – 7.29 (m, 2H), 5.58 (d, *J* = 9.4 Hz, 1H), 5.37 (d, *J* = 8.3 Hz, 1H), 4.65 – 4.54 (m, 1H), 4.54

– 4.34 (m, 3H), 4.23 (t, *J* = 7.0 Hz, 1H), 3.80 – 3.69 (m, 6H), 3.28 – 3.07 (m, 2H), 1.49 – 1.32 (m, 15H).

^{13}C NMR (101 MHz, $CDCl_3$) δ 171.12, 170.73, 156.01, 155.07, 143.89, 143.79, 141.42, 127.85, 127.21, 125.20, 120.11, 80.32, 67.35, 60.52, 53.20, 52.75, 52.70, 52.46, 47.26, 42.66, 28.42, 25.76, 24.50.

HRMS (ESI) *m/z* Calcd for $C_{30}H_{38}N_2NaO_8S_2^+$ $[M+Na]^+$: 641.1962, found: 641.1955.

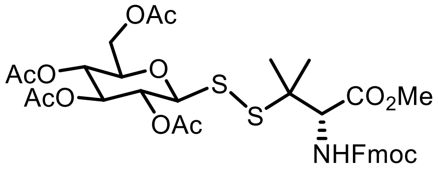

61

Compound **61** is an unknown compound, and was synthesized in 90% yield (134.6 mg, 0.2 mmol scale) using **7** as sulfenamide reagent under **Condition [G]**.

White solid (R_f = 0.25, PE:EA = 2:1, v/v)

^{1}H NMR (400 MHz, $CDCl_3$) δ 7.76 (d, *J* = 7.5 Hz, 2H), 7.61 (d, *J* = 7.5 Hz, 2H), 7.40 (t, *J* = 7.4 Hz, 2H), 7.36 – 7.28 (m, 2H), 5.71 (d, *J* = 9.7 Hz, 1H), 5.28 – 5.07 (m, 3H), 4.58 – 4.43 (m, 3H), 4.39 – 4.29 (m, 1H), 4.28 – 4.15 (m, 2H), 4.11 – 4.01 (m, 1H), 3.76 (s, 3H), 3.72 – 3.64 (m, 1H), 2.11 – 1.95 (m, 12H), 1.43 (s, 3H), 1.34 (s, 3H).

^{13}C NMR (101 MHz, $CDCl_3$) δ 170.68, 170.46, 170.32, 169.43, 169.34, 155.95, 143.94, 143.88, 141.51, 141.47, 127.88, 127.85, 127.23, 125.28, 125.18, 120.13, 88.63, 76.36, 74.07, 69.65, 68.06, 67.17, 62.03, 61.20, 52.45, 52.03, 47.38, 25.71, 25.24, 20.79, 20.74, 20.70.

HRMS (ESI) *m/z* Calcd for $C_{35}H_{41}NNaO_{13}S_2^+$ $[M+Na]^+$: 770.1912, found: 770.1921.

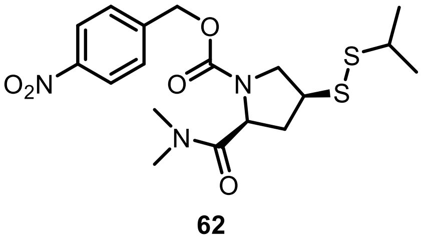

62

Compound **62** is an unknown compound, and was synthesized in 89% yield (76 mg, 0.2 mmol scale) using **3a** as sulfenamide reagent under **Condition [G]**.

White solid (R_f = 0.75, DCM:MeOH = 20:1, v/v)

^{1}H NMR (400 MHz, $CDCl_3$) δ 8.22 – 8.11 (m, 2H), 7.54 – 7.37 (m, 2H), 5.33 – 4.96 (m, 2H), 4.74 – 4.60 (m, 1H), 4.14 – 3.98 (m, 1H), 3.51 – 3.29 (m, 2H), 3.11 – 2.87 (m, 7H), 2.69 – 2.53 (m, 1H), 2.02– 1.89 (m, 1H), 1.27 (dd, *J* = 6.8, 2.7 Hz, 6H).

^{13}C NMR (101 MHz, $CDCl_3$) δ 170.97, 170.79, 153.88, 153.29, 147.60, 144.20, 144.07, 128.09, 127.92, 123.80, 123.66, 65.72, 56.71, 56.57, 53.08, 52.58, 47.78, 47.12, 41.70, 41.67, 36.99, 36.80, 36.58, 36.13, 36.08, 35.74, 22.53, 22.50.

HRMS (ESI) *m/z* Calcd for $C_{18}H_{26}N_3O_5S_2^+$ $[M+H]^+$: 428.1308, found: 428.1306.

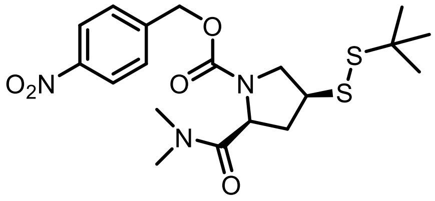

63

Compound **63** is an unknown compound, and was synthesized in 84% yield (74.2 mg, 0.2 mmol scale) using **13** as sulfenamide reagent under **Condition [H']**.

White solid (R_f = 0.75, DCM:MeOH = 20:1, v/v)

^{1}H NMR (400 MHz, $CDCl_3$) δ 8.25 – 8.11 (m, 2H), 7.56 – 7.36 (m, 2H), 5.34 – 4.96 (m, 2H), 4.75 – 4.58 (m, 1H), 4.19 – 4.00 (m, 1H), 3.53 – 3.28 (m, 2H), 3.11 – 2.85 (m, 6H), 2.73 – 2.58 (m, 1H), 2.01 – 1.84 (m, 1H), 1.30 (s, 9H).

^{13}C NMR (101 MHz, $CDCl_3$) δ 170.99, 170.82, 153.89, 153.29, 147.62, 144.23, 144.08, 128.10, 127.94, 123.83, 123.69, 65.74, 65.72, 56.83, 56.74, 53.32, 52.96, 48.93, 48.14, 47.79, 37.15, 37.01, 36.82, 36.16, 36.11, 29.94.

HRMS (ESI) *m/z* Calcd for $C_{19}H_{28}N_3O_5S_2^+$ $[M+H]^+$: 442.1465, found: 442.1461.

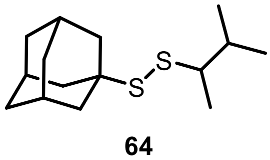

64

Compound **64** is an unknown compound, and was synthesized in 95% yield (51.3 mg, 0.2 mmol scale) using **X-1** as sulfenamide reagent under **Condition [H]**.

Colorless oil (R_f = 0.5, 100% PE)

^{1}H NMR (400 MHz, $CDCl_3$) δ 2.76 – 2.65(m, 1H), 2.10 – 2.03 (m, 3H), 2.03 – 1.93(m, 1H), 1.88 – 1.80 (m, 6H), 1.73 – 1.61 (m, 6H), 1.21 (d, *J* = 7.0 Hz, 3H), 0.98 (d, *J* = 6.9 Hz, 3H), 0.94 (d, *J* = 6.8 Hz, 3H).

^{13}C NMR (101 MHz, $CDCl_3$) δ 54.75, 49.19, 42.78, 36.31, 32.13, 29.99, 20.50, 17.84, 16.13.

HRMS (EI) *m/z* Calcd for $C_{15}H_{26}S_2^+$ $[M]^+$: 270.1470, found: 270.1471.

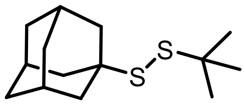

65

Compound **65** is an unknown compound, and was synthesized in 94% yield (48.2 mg, 0.2 mmol scale) using **13** as sulfenamide reagent under **Condition [H']**.

Colorless oil (R_f = 0.8, 100% PE)

^{1}H NMR (400 MHz, $CDCl_3$) δ 2.09 – 2.02 (m, 3H), 1.85 – 1.79 (m, 6H), 1.72 – 1.61 (m, 6H), 1.30 (s, 9H).

^{13}C NMR (101 MHz, $CDCl_3$) δ 47.97, 46.04, 43.37, 36.37, 30.79, 30.29.

HRMS (ESI) *m/z* Calcd for $C_{14}H_{25}S_2^+$ $[M+H]^+$: 257.1392, found: 257.1392.

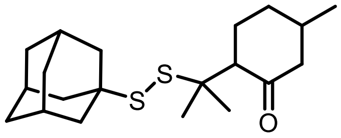

66

Compound **66** is an unknown compound, and was synthesized in 57% yield (40.2 mg, 0.2 mmol scale) using **15** as sulfenamide reagent under **Condition [H]**.

Colorless oil (R_f = 0.5, PE:EA = 20:1, v/v)

^{1}H NMR (400 MHz, $CDCl_3$) δ 2.58 – 2.46 (m, 2H), 2.31 – 2.24 (m, 1H), 2.10 – 2.00 (m, 4H), 1.94 – 1.84 (m, 2H), 1.84 – 1.77 (m, 6H), 1.72 – 1.59 (m, 6H), 1.52 – 1.44 (m, 4H), 1.38 – 1.31 (m, 4H), 1.00 (d, *J* = 6.2 Hz, 3H).

^{13}C NMR (101 MHz, $CDCl_3$) δ 211.18, 57.84, 52.50, 50.93, 48.23, 43.37, 36.99, 36.21, 34.62, 30.19, 29.97, 27.72, 23.47, 22.41.

HRMS (ESI) *m/z* Calcd for $C_{20}H_{32}NaOS_2^+$ $[M+Na]^+$: 375.1787, found: 375.1787.

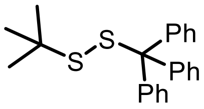

67

Compound **67** is an unknown compound, and was synthesized in 81% yield (59 mg, 0.2 mmol scale) using **13** as sulfenamide reagent under **Condition [H]**. (**Attention:** the polarity of this compound is same with triphenylmethanethiol.)

Colorless oil (R_f = 0.2, 100% PE)

^{1}H NMR (400 MHz, $CDCl_3$) δ 7.47 – 7.43 (m, 6H), 7.30 – 7.25 (m, 6H), 7.24 – 7.20 (m, 3H), 0.89 (s, 9H).

^{13}C NMR (101 MHz, $CDCl_3$) δ 144.19, 130.62, 127.74, 127.03, 71.35, 48.18, 30.17.

HRMS (ESI) *m/z* Calcd for $C_{23}H_{25}S_2^+$ $[M+H]^+$: 365.1392, found: 365.1395.

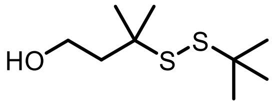

68

Compound **68** is an unknown compound, and was synthesized in 92% yield (38.3 mg, 0.2 mmol scale) using **13** as sulfenamide reagent under **Condition [H']**.

Colorless oil (R_f = 0.42, PE:EA = 5:1, v/v)

^{1}H NMR (400 MHz, $CDCl_3$) δ 3.78 (t, *J* = 7.0 Hz, 2H), 1.84 (t, *J* = 7.0 Hz, 2H), 1.75 (s, 1H), 1.30 (d, *J* = 2.8 Hz, 15H).

^{13}C NMR (101 MHz, $CDCl_3$) δ 59.99, 48.27, 46.55, 44.50, 30.70, 28.76.

HRMS (EI) *m/z* Calcd for $C_9H_{20}OS_2^+$ $[M]^+$: 208.0950, found: 208.0949.

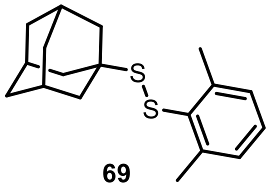

69

Compound **69** is an unknown compound, and was synthesized in 90% yield (54.8 mg, 0.2 mmol scale) using **X-2** as sulfenamide reagent under **Condition [H]**.

Colorless oil (R_f = 0.3, 100% PE)

^{1}H NMR (400 MHz, $CDCl_3$) δ 7.14 – 7.01 (m, 3H), 2.64 (s, 6H), 2.07 – 1.98 (m, 3H), 1.83 – 1.76 (m, 6H), 1.72 – 1.59 (m, 6H).

^{13}C NMR (101 MHz, $CDCl_3$) δ 141.48, 137.39, 128.51, 128.38, 50.26, 42.67, 36.25, 29.96, 22.39.

HRMS (EI) *m/z* Calcd for $C_{18}H_{24}S_2^+$ $[M]^+$: 304.1314, found: 304.1316.

Supplementary Discussion

2.1 Optimization of amidation of thiols with dioxazolones

2.1.1 Amidation of secondary thiol - screening of metal catalysts

Supplementary Table 1. Amidation of secondary thiol - screening of metal catalysts.

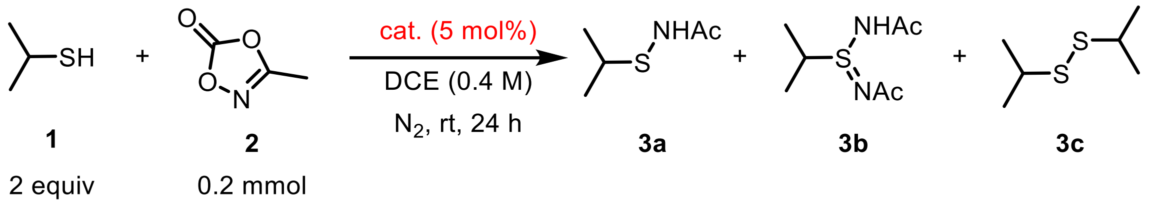

Entry	Conditions	yield of **3a** (%)	yield of **3b** (%)	yield of **3c** (%)
1	$FeCl_2·4H_2O$	21	51	9
2	$FeBr_2$	21	28	19
3	[Ru(*p*-cymene)Cl_2]$_2$	ND	ND	67
4	[RhCp*Cl_2]$_2$	ND	ND	2
5	[IrCp*Cl_2]$_2$	3	ND	ND
6	$Pd(OAc)_2$	ND	ND	ND
7	$RuCl_3$	13	12	30
8	$PtCl_2$	ND	ND	ND
9	[Ir(cod)Cl]$_2$	5	ND	25
10	$CoCl_2$	12	ND	2
11	$NiBr_2·3H_2O$	17	15	2
12	$Rh_2(OAc)_4$	ND	ND	ND
13	$Cu(OAc)_2$	27	ND	4
14	CuOAc	53	ND	8
15	$FeCl_2$·PDI	17	32	5
16	$FeBr_2$·PDI	24	36	5
17	[RuCp*Cl_2]$_2$	16	39	15

18	Fe^{III}(TPP)Cl	5	ND	7

All screening reactions were carried on a 0.2 mmol scale, yields are based on ^{1}H NMR analysis of crude reaction using CH_2Br_2 as internal standard. ND: not detected.

- Fe, Ir, Ru, Co, Ni and Cu complexes can give the desired S-imidation products in varied yields and selectivities.
- **3a** can be formed selectively when using CuOAc as a catalyst.
- **3c** is not a major byproduct in most reaction systems that use secondary thiol as a substrate.
- Di-amination product **3b** can be formed in some cases even when thiol is excess, demonstratingg that **3a** still displays high amidation reactivity.

2.1.2 CuOAc catalyzed amidation of secondary thiol - screening of solvent

Supplementary Table 2. CuOAc catalyzed amidation of secondary thiol - screening of solvent

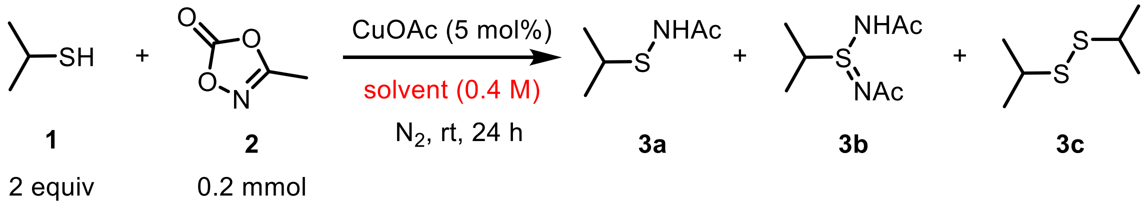

Entry	solvent	yield of **3a** (%)	yield of **3b** (%)	yield of **3c** (%)
1	DCE	53	ND	8
2	$CHCl_3$	22	ND	4
3	MeOH	31	ND	5
4	MeCN	29	ND	9
5	toluene	75	ND	10
6	PhCl	57	ND	4
7	^{n}Hexane	83	ND	11
8	HFIP	11	ND	4

All screening reactions were carried on a 0.2 mmol scale, yields are based on ^{1}H NMR analysis of crude reaction using CH_2Br_2 as internal standard. ND: not detected.

- Non-polar solvents are favorable for the formation of products when using CuOAc as a catalyst.
- Di-amination product **3b** was not detected in all examined solvents.
- *n*-Hexane was the most favorable solvent using CuOAc as catalyst.

2.1.3 IPrCuCl catalyzed amidation of secondary thiol - screening of solvent

Supplementary Table 3. IPrCuCl catalyzed amidation of secondary thiol - screening of solvent

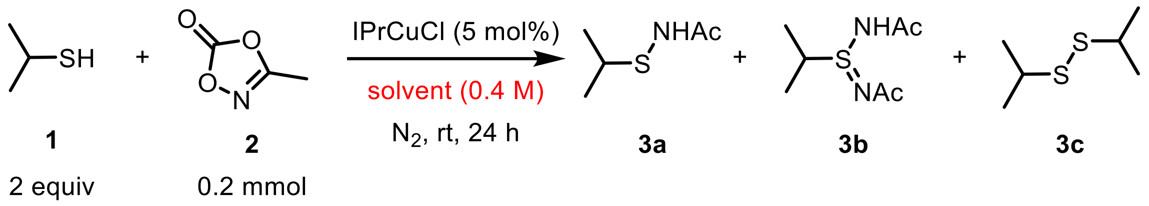

Entry	solvent	yield of **3a** (%)	yield of **3b** (%)	yield of **3c** (%)
1	DCE	57	11	19
2	MeOH	27	ND	7
3	HFIP	95	ND	5
4	toluene	37	18	9
5	THF	55	ND	6
6	MeCN	64	ND	12
7	*n*Hexane	46	29	13
8	DMF	83	ND	ND

All screening reactions were carried on a 0.2 mmol scale, yields are based on ^{1}H NMR analysis of crude reaction using CH_2Br_2 as internal standard. ND: not detected.

- When employing IPrCuCl as a catalyst, non-polar solvent systems are more favorable for the formation of **3b**, while polar solvent systems produce more sulfenamide **3a**.
- Polar solvents are required for dissolving polar substrates.

2.1.4 Amidation of primary thiol - screening of Cu catalysts

Supplementary Table 4. Amidation of primary thiol - screening of Cu catalysts

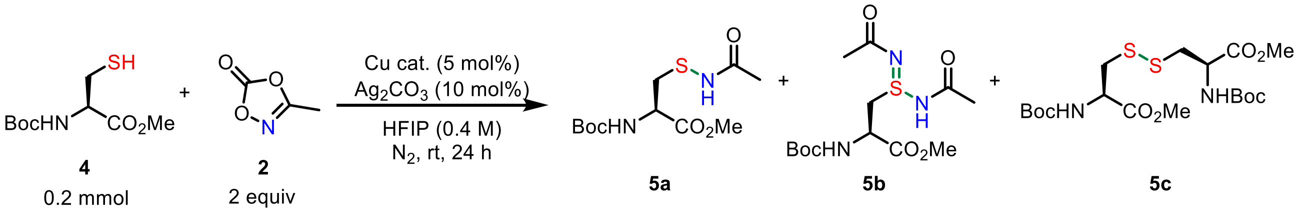

Entry	Cu cat.	yield of **5a** (%)	yield of **5b** (%)	yield of **5c** (%)
1	IPrCuCl	74	ND	22
2	IPrCuBr	60	ND	29
3	IPrCuI	47	ND	32
4	SIPrCuCl	49	ND	29
5	IMesCuCl	65	ND	20
6	SIMesCuCl	62	ND	21
7	ICyCuCl	57	ND	25
8	IAdCuCl	59	ND	24

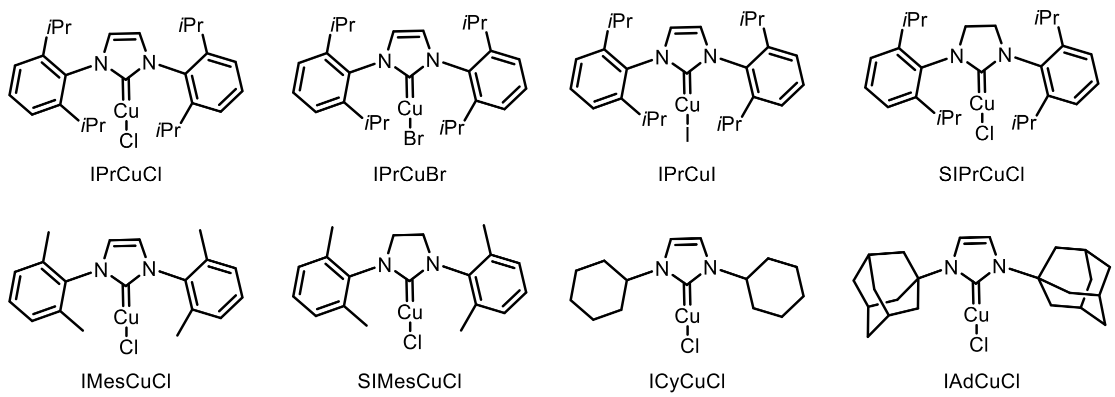

All screening reactions were carried on a 0.2 mmol scale, yields are based on ^{1}H NMR analysis of crude reaction using CH_2Br_2 as internal standard. ND: not detected.

- As the atomic radius of the halogen ion increases, the yield of **5a** decreases and more disulfide **5c** is detected.
- Catalysts featuring more sterically hindered aryl substituents exert beneficial effect on the formation of **5a**.

2.2 One-pot synthesis of 47 and 51

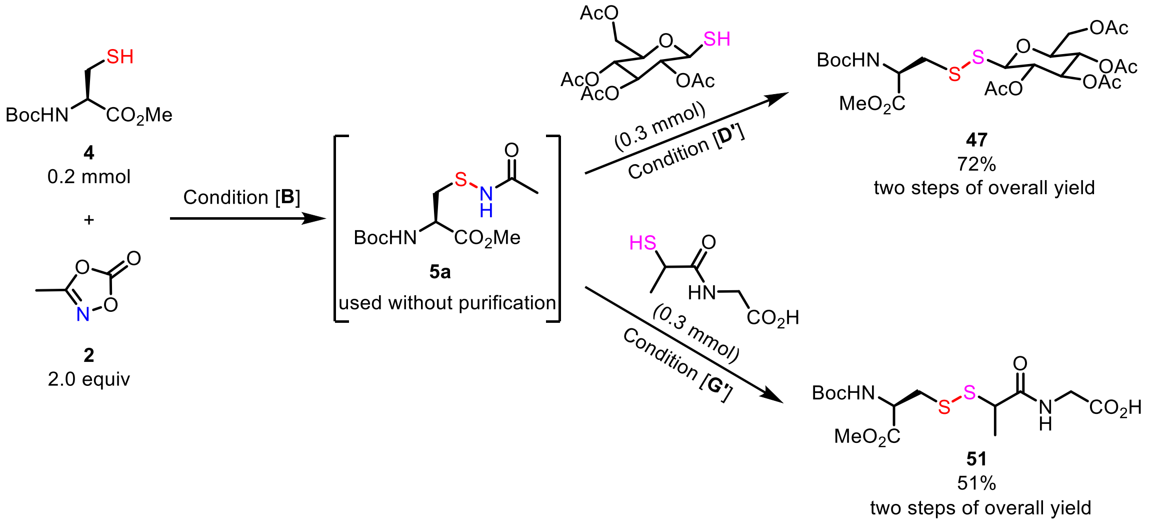

Supplementary Figure 16. One-pot synthesis of **47** and **51.**

One-pot synthesis of **47**: To a solution of Boc-protected cysteine methyl ester **4** (47 mg, 0.2 mmol, 1.0 equiv), IPrCuCl (4.9 mg, 0.01 mmol, 5 mol%) and Ag_2CO_3 (5.5 mg, 0.02 mmol, 10 mol%) in HFIP (0.5 mL, 0.4 M), 3-methyldioxazolone 2 (0.4 mmol, 40.4 mg, 2.0 equiv) was added. The reaction mixture was stirred under N_2 atmosphere for 24 hours at rt. The reaction mixture was then concentrated

under reduced pressure. The residue was dried under high vacuum for 30 min and give crude **5a**. The crude **5a** was dissolved in MeOH (4 mL, 0.05 M), and 1-thio-β-D-glucose tetraacetate (109.3 mg, 0.3 mmol, 1.5 equiv) was added to the mixture. The reaction mixture was stirred under air for 12 hours at 35 °C. The reaction mixture was then concentrated under reduced pressure. The resulting residue was purified by silica gel flash chromatography using hexanes/EtOAc (5/1 to 1.5:1) eluent to give the desired product **47** as a white solid (86.1 mg, 72% overall yield).

The procedure of one-pot synthesis of **51** is similar to **47** except for using PBS/THF = 1:1 as solvent in the S-S coupling step and gave **51** as a white solid (40.4 mg, 51% overall yield).

Note: Condition [**D'**] or [**G'**] are same as condition **[D]** or **[G]** except a 1:1.5 ratio of sulfenamide (**S-NHAc**) and thiol (**S'**).

2.3 Comparisons of oxidative coupling of thiols with our developed S-S coupling methods

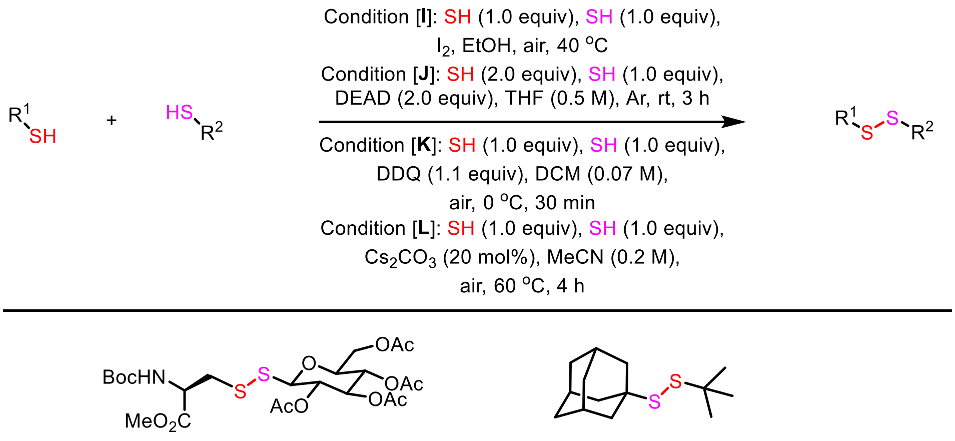

47
trace [**I**], 30% [**J**]
57% [**K**], 22% [**L**]
89% [**D**]

65
7% [**I**][a], trace [**J**][a]
trace [**K**][a], trace [**L**][a]
94% [**H'**]

Supplementary Figure 17. Comparisons of oxidative coupling of thiols with our developed S-S coupling methods. Isolated yield by silica gel column chromatography at 0.2 mmol scale unless otherwise specified. DEAD: Diethyl azodicarboxylate. DDQ: 2,3-Dichloro-5,6-dicyano-1,4-benzoquinone. [a]0.4 mmol scale.

- We compared our S-S coupling strategy with previously reported oxidative couplings procedures, such as using I_2, DEAD, DDQ or Cs_2CO_3/air as oxidants (references 80-83 in the main text.). For the less bulky substrate **47**, DDQ displays better oxidative reactivity of cross coupling than I_2,

DEAD and Cs_2CO_3/air systems and gave **47** in 57% yield. Self-oxidative coupling of thiols can not be avoided. But our S-S coupling method using sulfenamide **5a** as a *S*-sulfenylation reagent (Condition [**D**]) gave **47** in 89% yield. For the bulky substrate **65**, all of oxidative couplings methods were unsuccessful, indicating that sterically hindered substrates are challengging to obtain by conventional oxidative coupling procedures. On the contrary, sulfenamide **13** demonstrated to be an effective S-sulfenylation reagent that exhibited high reactivity and gave **65** in 94% yield (Condition [**H'**]).

Supplementary Notes

3.1 X-ray crystallographic data of compound 7

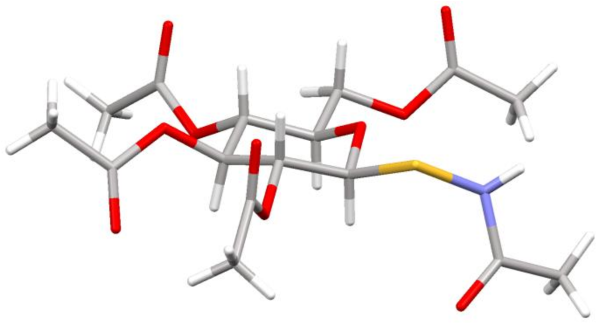

Supplementary Figure 18. X-ray structure of compound **7**

Single crystals for X-ray studies were grown by slow evaporation of a solution of compound **7** in a mixture of $CHCl_3$/n-heptane in NMR tube at room temperature. The X-ray data of **7** is deposited in the Cambridge Crystallographic Data Centre with a number of CCDC: 2151305.

Supplementary table 5. Crystal data and structure refinement for **7**.

Identification code	**7**
Empirical formula	$C_{16}H_{23}NO_{10}S$
Formula weight	421.41
Temperature/K	294.15
Crystal system	monoclinic

Space group	$P2_1$
a/Å	7.4149(5)
b/Å	10.4557(7)
c/Å	13.9701(7)
α /°	90
β /°	98.394(5)
γ /°	90
Volume/Å^3	1071.47(12)
Z	2
ρ_{calc}g/cm^3	1.306
μ /mm^{-1}	1.799
F(000)	444.0
Crystal size/mm^3	0.2 × 0.17 × 0.13
Radiation	CuKα (λ = 1.54184)
2Θ range for data collection/°	10.61 to 159.874
Index ranges	-8 ≤ h ≤ 7, -13 ≤ k ≤ 13, -17 ≤ l ≤ 17
Reflections collected	24757
Independent reflections	4457 [R_{int} = 0.0925, R_{sigma} = 0.0471]
Data/restraints/parameters	4457/7/258
Goodness-of-fit on F^2	1.070
Final R indexes [I>=2σ (I)]	R_1 = 0.0508, wR_2 = 0.1548
Final R indexes [all data]	R_1 = 0.0545, wR_2 = 0.1593
Largest diff. peak/hole / e Å^{-3}	0.20/-0.22
Flack parameter	0.025(14)

Supplementary table 6. Fractional Atomic Coordinates (×10^4) and Equivalent Isotropic Displacement Parameters (Å^2×10^3) for **7**. U_{eq} is defined as 1/3 of of the trace of the orthogonalised U_{IJ} tensor.

**Atom**	***x***	***y***	***z***	**U(eq)**
S1	7655.5(15)	5422.1(18)	9296.8(7)	105.2(5)
O1	5416(9)	7550(9)	9864(5)	157(3)
O2	5715(4)	4484(3)	7749.1(19)	83.2(7)
O3	2163(5)	3436(3)	7245(3)	99.3(9)
O4	2874(10)	1447(5)	7652(5)	155(2)
O5	5070(4)	5053(2)	5151.9(17)	75.7(6)
O6	5852(6)	3204(4)	4537(3)	114.4(12)
O7	8766(4)	5737(3)	5784.5(18)	75.5(6)
O8	8812(6)	7836(3)	5506(3)	107.6(11)
O9	8910(4)	7111(3)	7662(2)	85.6(7)
O10	11659(7)	6309(6)	8090(7)	184(3)
N1	5774(6)	5340(9)	9837(3)	133(2)
C1	3312(11)	6243(15)	10569(6)	170(4)
C2	4910(11)	6454(15)	10082(5)	144(3)
C3	6660(5)	5627(4)	8054(2)	80.3(9)
C4	4685(6)	4634(4)	6809(3)	75.7(8)
C5	6041(5)	4770(3)	6094(2)	71.0(8)
C6	7330(5)	5876(3)	6372(2)	67.5(7)
C7	8146(5)	5873(3)	7430(3)	73.1(8)
C8	3513(9)	3454(5)	6595(4)	105.5(16)
C9	1999(7)	2368(5)	7735(4)	95.0(12)
C10	638(10)	2514(7)	8391(6)	124(2)
C11	5130(5)	4209(4)	4420(3)	78.2(9)

C12	4181(8)	4718(6)	3496(3)	98.1(13)
C13	9328(5)	6780(4)	5357(3)	80.0(9)
C14	10629(7)	6409(7)	4692(4)	104.7(15)
C15	10714(7)	7232(6)	7954(4)	104.2(15)
C16	11250(11)	8563(8)	8124(7)	148(3)

Supplementary table 7. Anisotropic Displacement Parameters ($Å^2 \times 10^3$) for **7**. The Anisotropic displacement factor exponent takes the form: $-2\pi^2[h^2a^{*2}U_{11}+2hka^*b^*U_{12}+...]$.

**Atom**	**U_{11}**	**U_{22}**	**U_{33}**	**U_{23}**	**U_{13}**	**U_{12}**
S1	79.6(6)	170.8(13)	65.0(4)	1.4(6)	10.1(3)	14.1(7)
O1	116(4)	236(8)	123(4)	-46(5)	27(3)	34(4)
O2	91.0(18)	89.8(17)	70.9(14)	10.8(12)	18.7(12)	3.3(13)
O3	110(2)	90.7(18)	104(2)	8.0(15)	37.0(18)	-13.7(16)
O4	180(5)	114(3)	192(5)	53(3)	102(4)	40(3)
O5	87.0(15)	74.0(13)	66.0(12)	-4.7(10)	11.4(10)	0.3(11)
O6	125(3)	102(2)	114(3)	-38.9(19)	6(2)	14(2)
O7	74.7(13)	79.8(14)	74.9(13)	2.4(11)	20.6(10)	4.9(11)
O8	114(3)	84.8(19)	131(3)	23.0(18)	43(2)	0.3(17)
O9	84.6(17)	81.5(16)	86.8(16)	-9.1(13)	-0.4(12)	0.3(12)
O10	87(3)	153(4)	295(9)	-64(5)	-33(4)	14(3)
N1	86(3)	247(7)	68.2(18)	0(3)	18.5(16)	10(4)
C1	115(5)	283(11)	122(5)	-12(6)	46(4)	20(6)
C2	96(4)	256(11)	80(3)	-19(5)	13(2)	15(6)
C3	78(2)	96(2)	67.4(16)	-2.0(17)	10.6(13)	11.0(18)
C4	84(2)	76.3(19)	69.5(18)	-1.9(15)	18.9(15)	-9.5(16)
C5	78(2)	69.6(18)	67.0(17)	1.2(13)	16.3(14)	2.3(14)

C6	68.6(17)	68.3(17)	67.3(16)	2.2(13)	15.8(12)	5.7(12)
C7	74(2)	74.5(19)	70.7(17)	-0.7(14)	9.4(14)	7.2(15)
C8	130(4)	94(3)	103(3)	-12(2)	53(3)	-33(3)
C9	90(3)	100(3)	97(3)	18(2)	21(2)	-6(2)
C10	107(4)	142(5)	133(5)	43(4)	51(3)	12(3)
C11	76(2)	84(2)	77(2)	-13.3(16)	22.2(15)	-15.9(17)
C12	108(3)	117(3)	70(2)	-7(2)	18(2)	-26(3)
C13	68.4(19)	94(3)	77.8(19)	17.2(18)	10.6(14)	-4.3(17)
C14	85(3)	141(4)	94(3)	16(3)	31(2)	-7(3)
C15	87(3)	114(3)	108(3)	-21(3)	3(2)	-8(3)
C16	129(5)	131(5)	171(7)	-19(5)	-16(5)	-39(4)

Supplementary table 8. Bond Lengths for **7**.

Atom	Atom	Length/Å	Atom	Atom	Length/Å
S1	N1	1.682(4)	O9	C7	1.430(5)
S1	C3	1.797(4)	O9	C15	1.346(6)
O1	C2	1.257(13)	O10	C15	1.191(8)
O2	C3	1.419(6)	N1	C2	1.396(14)
O2	C4	1.429(5)	C1	C2	1.466(12)
O3	C8	1.447(6)	C3	C7	1.523(5)
O3	C9	1.325(6)	C4	C5	1.523(5)
O4	C9	1.177(7)	C4	C8	1.513(6)
O5	C5	1.436(4)	C5	C6	1.515(5)
O5	C11	1.356(5)	C6	C7	1.513(5)
O6	C11	1.180(6)	C9	C10	1.466(7)

O7	C6	1.444(4)	C11	C12	1.476(7)
O7	C13	1.339(5)	C13	C14	1.485(6)
O8	C13	1.197(6)	C15	C16	1.458(10)

Supplementary table 9. Bond Angles for **7**.

Atom	Atom	Atom	Angle/°	Atom	Atom	Atom	Angle/°
N1	S1	C3	100.9(2)	O7	C6	C5	105.5(3)
C3	O2	C4	111.0(3)	O7	C6	C7	109.4(3)
C9	O3	C8	117.3(4)	C7	C6	C5	113.4(3)
C11	O5	C5	119.3(3)	O9	C7	C3	108.6(3)
C13	O7	C6	118.4(3)	O9	C7	C6	107.9(3)
C15	O9	C7	119.8(4)	C6	C7	C3	109.8(3)
C2	N1	S1	120.6(7)	O3	C8	C4	108.5(4)
O1	C2	N1	122.5(7)	O3	C9	C10	111.0(5)
O1	C2	C1	122.6(11)	O4	C9	O3	123.1(5)
N1	C2	C1	114.8(12)	O4	C9	C10	125.9(5)
O2	C3	S1	107.7(3)	O5	C11	C12	111.2(4)
O2	C3	C7	109.7(3)	O6	C11	O5	122.6(4)
C7	C3	S1	110.1(3)	O6	C11	C12	126.2(4)
O2	C4	C5	107.3(3)	O7	C13	C14	109.8(4)
O2	C4	C8	107.8(4)	O8	C13	O7	123.0(4)
C8	C4	C5	111.2(3)	O8	C13	C14	127.1(4)
O5	C5	C4	109.2(3)	O9	C15	C16	112.0(6)
O5	C5	C6	106.9(3)	O10	C15	O9	120.5(5)
C6	C5	C4	110.8(3)	O10	C15	C16	127.4(6)

Supplementary table 10. Torsion Angles for **7**.

A	B	C	D	Angle/°	A	B	C	D	Angle/°
S1	N1	C2	O1	4.3(10)	C4	C5	C6	C7	48.1(4)
S1	N1	C2	C1	-178.6(5)	C5	O5	C11	O6	5.2(6)
S1	C3	C7	O9	-69.4(3)	C5	O5	C11	C12	-175.2(3)
S1	C3	C7	C6	172.8(3)	C5	C4	C8	O3	-175.4(4)
O2	C3	C7	O9	172.2(3)	C5	C6	C7	O9	-164.3(3)
O2	C3	C7	C6	54.4(4)	C5	C6	C7	C3	-46.1(4)
O2	C4	C5	O5	-174.2(3)	C6	O7	C13	O8	6.8(6)
O2	C4	C5	C6	-56.6(4)	C6	O7	C13	C14	-172.8(3)
O2	C4	C8	O3	67.2(5)	C7	O9	C15	O10	-5.6(9)
O5	C5	C6	O7	-73.2(3)	C7	O9	C15	C16	178.0(6)
O5	C5	C6	C7	167.1(3)	C8	O3	C9	O4	-1.2(9)
O7	C6	C7	O9	78.2(3)	C8	O3	C9	C10	177.6(6)
O7	C6	C7	C3	-163.5(3)	C8	C4	C5	O5	68.1(5)
N1	S1	C3	O2	-66.6(4)	C8	C4	C5	C6	-174.4(4)
N1	S1	C3	C7	173.7(4)	C9	O3	C8	C4	-129.3(5)
C3	S1	N1	C2	-83.4(5)	C11	O5	C5	C4	-116.7(3)
C3	O2	C4	C5	68.1(4)	C11	O5	C5	C6	123.4(3)
C3	O2	C4	C8	-172.0(3)	C13	O7	C6	C5	135.7(3)
C4	O2	C3	S1	172.3(2)	C13	O7	C6	C7	-102.1(4)
C4	O2	C3	C7	-67.9(4)	C15	O9	C7	C3	123.5(4)
C4	C5	C6	O7	167.8(3)	C15	O9	C7	C6	-117.5(4)

Supplementary table 11. Hydrogen Atom Coordinates ($Å \times 10^4$) and Isotropic Displacement Parameters ($Å^2 \times 10^3$) for **7**.

Atom	*x*	*y*	*z*	U(eq)

H1	5946.03	4830.32	10324.72	160
H1A	3303.08	6861.62	11075.72	255
H1B	3363.05	5397.72	10839.84	255
H1C	2222.45	6330.98	10109.06	255
H3	5805.72	6347	7998.19	96
H4	3915.4	5397.9	6790.65	91
H5	6734.32	3975.34	6072.43	85
H6	6687.01	6685.22	6214.65	81
H7	9094.81	5216.5	7550.9	88
H8A	2917.24	3470.56	5929.54	127
H8B	4263.61	2691.2	6688.08	127
H10A	967.55	3221.68	8818.72	186
H10B	591.44	1746.16	8763.04	186
H10C	-536.37	2670.5	8019.94	186
H12A	2923.66	4465.63	3415.71	147
H12B	4742.57	4385.48	2970.57	147
H12C	4261.23	5634.72	3500.02	147
H14A	11848.07	6419.87	5036.91	157
H14B	10538.84	7002.96	4163.33	157
H14C	10342.43	5564.28	4446.78	157
H16A	11039.55	9025.81	7524.75	221
H16B	12521.03	8603.46	8383.99	221
H16C	10543.59	8936.42	8575.8	221

checkCIF/PLATON report

Structure factors have been supplied for datablock(s) p20211012a

THIS REPORT IS FOR GUIDANCE ONLY. IF USED AS PART OF A REVIEW PROCEDURE FOR PUBLICATION, IT SHOULD NOT REPLACE THE EXPERTISE OF AN EXPERIENCED CRYSTALLOGRAPHIC REFEREE.

No syntax errors found. CIF dictionary Interpreting this report

Datablock: p20211012a

Bond precision:	C-C = 0.0071 A		Wavelength=1.54184
Cell:	a=7.4149(5)	b=10.4557(7)	c=13.9701(7)
	alpha=90	beta=98.394(5)	gamma=90
Temperature:	294 K		

	Calculated	Reported
Volume	1071.47(12)	1071.47(12)
Space group	P 21	P 1 21 1
Hall group	P 2yb	P 2yb
Moiety formula	C16 H23 N O10 S	C16 H23 N O10 S
Sum formula	C16 H23 N O10 S	C16 H23 N O10 S
Mr	421.41	421.41
Dx,g cm-3	1.306	1.306
Z	2	2
Mu (mm-1)	1.799	1.799
F000	444.0	444.0
F000’	446.25	
h,k,lmax	9,13,17	8,13,17
Nref	4690[2476]	4457
Tmin,Tmax	0.733,0.791	0.882,1.000
Tmin’	0.665	

Correction method= # Reported T Limits: Tmin=0.882 Tmax=1.000
AbsCorr = MULTI-SCAN

Data completeness= 1.80/0.95 Theta(max)= 79.937

R(reflections)= 0.0508(3923) wR2(reflections)= 0.1593(4457)

S = 1.070 Npar= 258

The following ALERTS were generated. Each ALERT has the format
test-name_ALERT_alert-type_alert-level.
Click on the hyperlinks for more details of the test.

Alert level C

```
PLAT193_ALERT_1_C Cell and Diffraction Temperatures Differ by ....           1 Degree
PLAT241_ALERT_2_C High   'MainMol' Ueq as Compared to Neighbors of          C8 Check
PLAT242_ALERT_2_C Low    'MainMol' Ueq as Compared to Neighbors of          C9 Check
PLAT242_ALERT_2_C Low    'MainMol' Ueq as Compared to Neighbors of         C11 Check
PLAT242_ALERT_2_C Low    'MainMol' Ueq as Compared to Neighbors of         C13 Check
PLAT242_ALERT_2_C Low    'MainMol' Ueq as Compared to Neighbors of         C15 Check
PLAT260_ALERT_2_C Large Average Ueq of Residue Including        S1       0.107 Check
PLAT340_ALERT_3_C Low Bond Precision on  C-C Bonds ...............      0.0071 Ang.
PLAT911_ALERT_3_C Missing FCF Refl Between Thmin & STh/L=    0.600          35 Report
```

Alert level G

```
PLAT003_ALERT_2_G Number of Uiso or Uij Restrained non-H Atoms ...           1 Report
PLAT007_ALERT_5_G Number of Unrefined Donor-H Atoms ..............           1 Report
PLAT186_ALERT_4_G The CIF-Embedded .res File Contains ISOR Records           1 Report
PLAT199_ALERT_1_G Reported _cell_measurement_temperature ..... (K)         293 Check
PLAT791_ALERT_4_G Model has Chirality at C3          (Sohnke SpGr)           S Verify
PLAT791_ALERT_4_G Model has Chirality at C4          (Sohnke SpGr)           R Verify
PLAT791_ALERT_4_G Model has Chirality at C5          (Sohnke SpGr)           S Verify
PLAT791_ALERT_4_G Model has Chirality at C6          (Sohnke SpGr)           S Verify
PLAT791_ALERT_4_G Model has Chirality at C7          (Sohnke SpGr)           R Verify
PLAT860_ALERT_3_G Number of Least-Squares Restraints ............            7 Note
PLAT910_ALERT_3_G Missing # of FCF Reflection(s) Below Theta(Min).           1 Note
PLAT912_ALERT_4_G Missing # of FCF Reflections Above STh/L=  0.600          33 Note
PLAT978_ALERT_2_G Number C-C Bonds with Positive Residual Density.           0 Info
PLAT992_ALERT_5_G Repd & Actual _reflns_number_gt Values Differ by           9 Check
```

0 **ALERT level A** = Most likely a serious problem - resolve or explain
0 **ALERT level B** = A potentially serious problem, consider carefully
9 **ALERT level C** = Check. Ensure it is not caused by an omission or oversight
14 **ALERT level G** = General information/check it is not something unexpected

2 ALERT type 1 CIF construction/syntax error, inconsistent or missing data
8 ALERT type 2 Indicator that the structure model may be wrong or deficient
4 ALERT type 3 Indicator that the structure quality may be low
7 ALERT type 4 Improvement, methodology, query or suggestion
2 ALERT type 5 Informative message, check

It is advisable to attempt to resolve as many as possible of the alerts in all categories. Often the minor alerts point to easily fixed oversights, errors and omissions in your CIF or refinement strategy, so attention to these fine details can be worthwhile. In order to resolve some of the more serious problems it may be necessary to carry out additional measurements or structure refinements. However, the purpose of your study may justify the reported deviations and the more serious of these should normally be commented upon in the discussion or experimental section of a paper or in the "special_details" fields of the CIF. checkCIF was carefully designed to identify outliers and unusual parameters, but every test has its limitations and alerts that are not important in a particular case may appear. Conversely, the absence of alerts does not guarantee there are no aspects of the results needing attention. It is up to the individual to critically assess their own results and, if necessary, seek expert advice.

Publication of your CIF in IUCr journals

A basic structural check has been run on your CIF. These basic checks will be run on all CIFs submitted for publication in IUCr journals (*Acta Crystallographica*, *Journal of Applied Crystallography*, *Journal of Synchrotron Radiation*); however, if you intend to submit to *Acta Crystallographica Section C* or *E* or *IUCrData*, you should make sure that full publication checks are run on the final version of your CIF prior to submission.

Publication of your CIF in other journals

Please refer to the *Notes for Authors* of the relevant journal for any special instructions relating to CIF submission.

PLATON version of 13/07/2021; check.def file version of 13/07/2021

Datablock p20211012a - ellipsoid plot

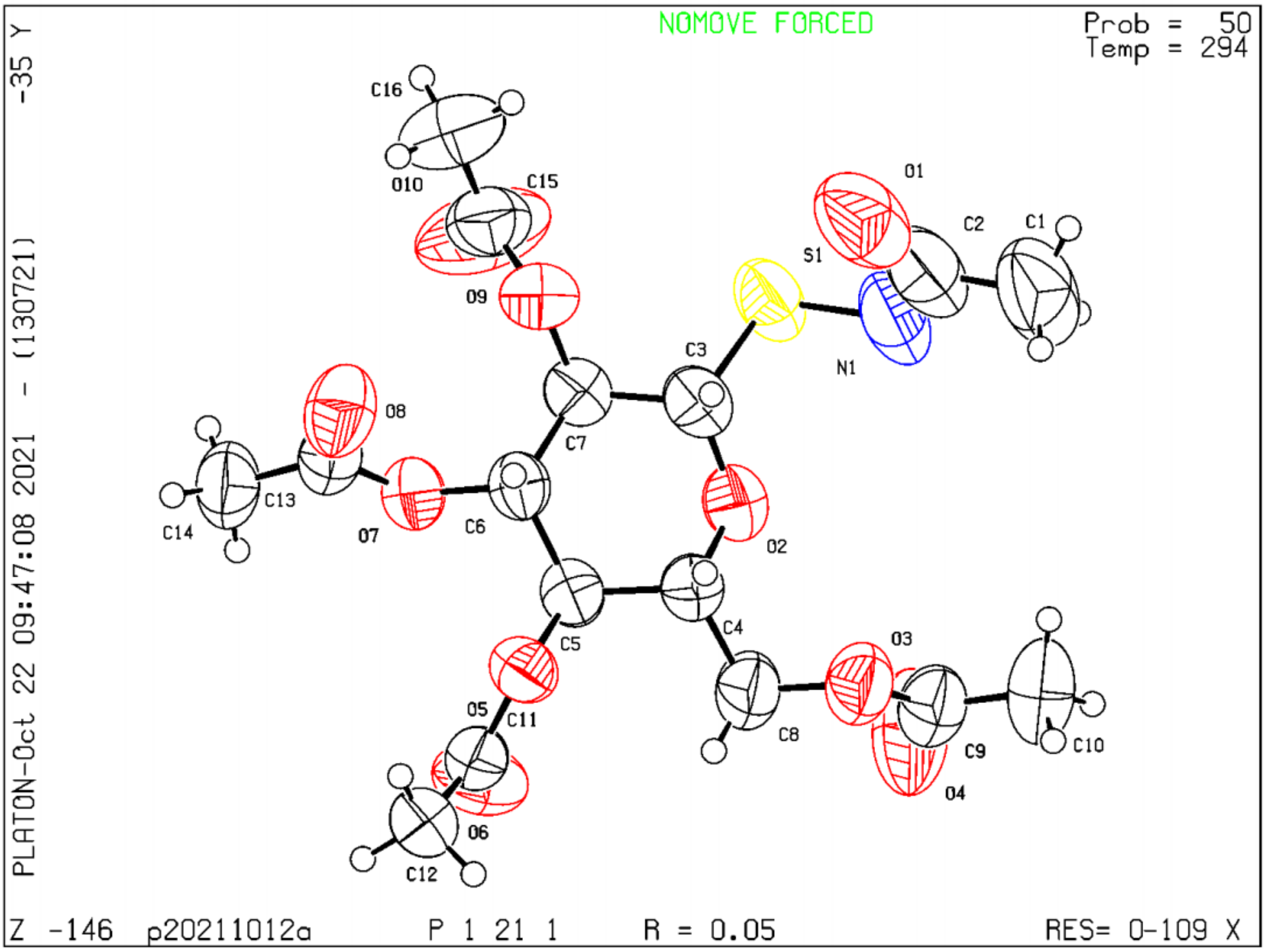

Supplementary Figure 19. The ellipsoids of non-hydrogen atoms are shown at 50% probability levels for compound **7**.

3.2 HPLC spectra of substrates 5a and 46

Peak Integration Report

Sample Name:	**BZQ-Cys-S-N-rac-AY**	*Inj. Vol.:*	**30.00**
Injection Type:	**Unknown**	*Dilution Factor:*	**1.0000**
Instrument Method:	**80-20 20 min 220nm**	*Operator:*	**Chen**
Inj. Date / Time:	**26-Jul-2022 / 12:30**	*Run Time:*	**20.00**

No.	Time min	Peak Name	Peak Type	Area mAU*min	Height mAU	Amount n.a.
			TOTAL:	0.00	0.00	0.00

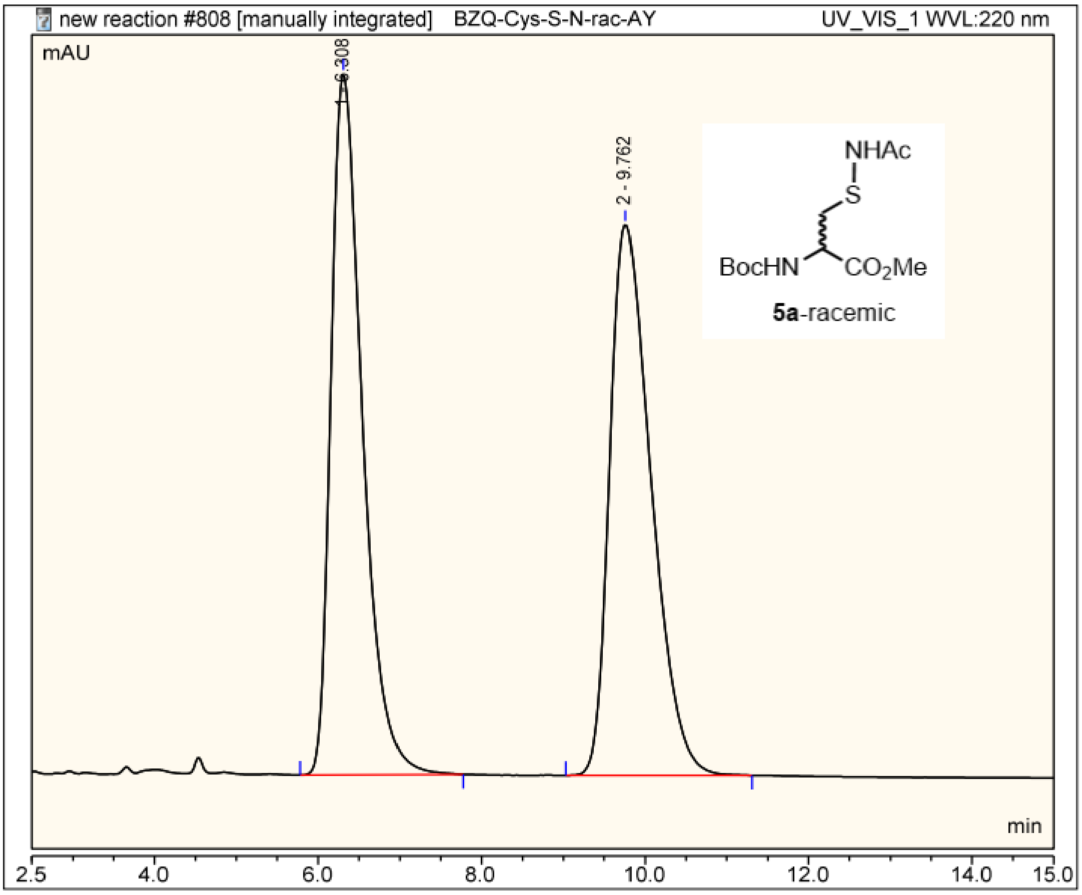

Integration Results

No.	Peak Name	Retention Time min	Area mAU*min	Height mAU	Relative Area %	Relative Height %	ee %
1		6.308	42.430	94.821	49.39	56.01	1.22
2		9.762	43.478	74.474	50.61	43.99	
**Total:**			**85.908**	**169.295**	**100.00**	**100.00**	

Supplementary Figure 20. HPLC spectrum of 5a-racemic. The ee value of **5a**-racemic was determined by HPLC analysis on a CHIRALPAK® AY-H column (hexanes: isopropanol = 80:20, 1.0 mL/min, T = 30 ºC, 220 nm), TR = 6.31 min and 9.76 min.

Sample Name:	**BZQ-Cys-S-N-L**	*Inj. Vol.:*	**15.00**
Injection Type:	**Unknown**	*Dilution Factor:*	**1.0000**
Instrument Method:	**80-20 20 min 220nm**	*Operator:*	**Chen**
Inj. Date / Time:	**26-Jul-2022 / 11:29**	*Run Time:*	**20.00**

No.	Time min	Peak Name	Peak Type	Area mAU*min	Height mAU	Amount n.a.
			**TOTAL:**	0.00	0.00	0.00

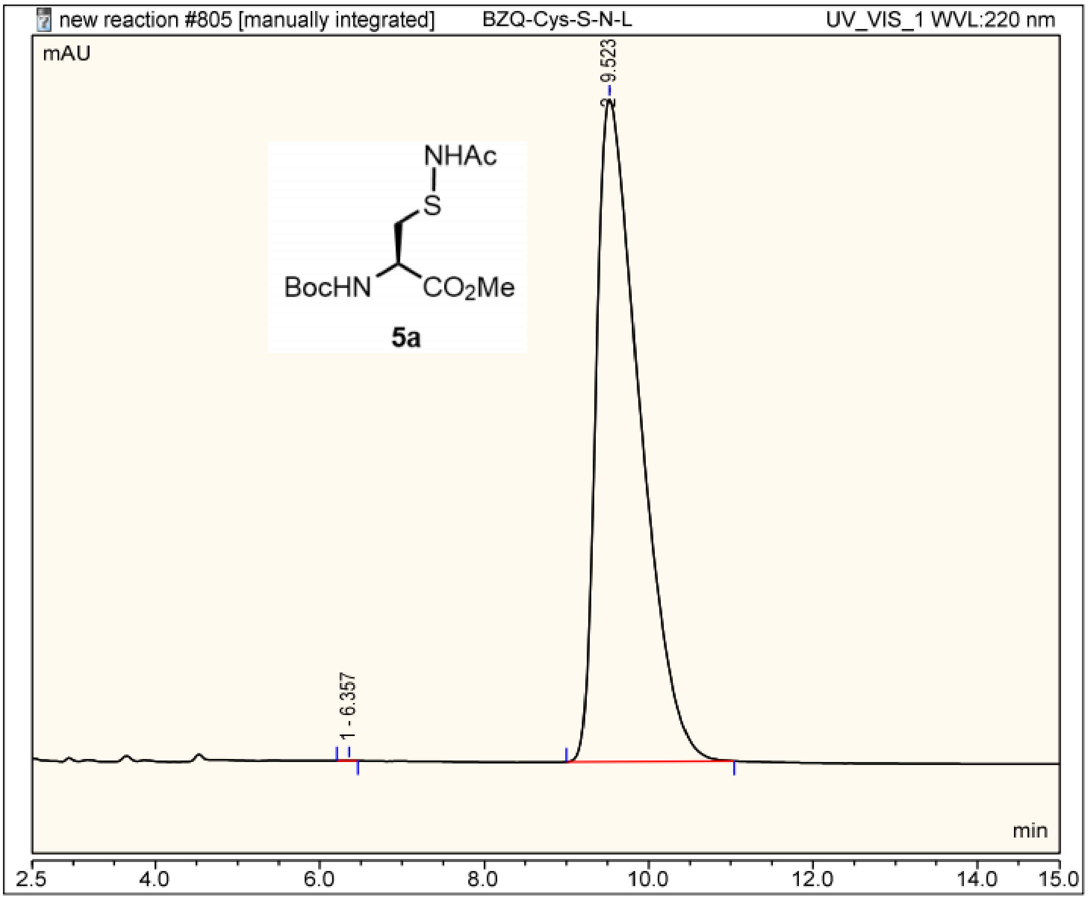

Integration Results							
No.	Peak Nam	Retention Time min	Area mAU*min	Height mAU	Relative Area %	Relative Height %	ee %
1		6.357	0.003	0.026	0.00	0.02	99.99
2		9.523	89.669	145.756	100.00	99.98	
**Total:**			**89.671**	**145.781**	**100.00**	**100.00**	

Supplementary Figure 21. HPLC spectrum of 5a. The ee value of **5a** (>99.9% ee) was determined by HPLC analysis on a CHIRALPAK® AY-H column (hexanes: isopropanol = 80:20, 1.0 mL/min, T = 30 ºC, 220 nm), TR = 6.36 min (minor) and 9.52 min (major)..

Sample Name:	**BZQ-Cys-S-S-rac-AY**	*Inj. Vol.:*	**10.00**
Injection Type:	**Unknown**	*Dilution Factor:*	**1.0000**
Instrument Method:	**80-20 20 min 254nm**	*Operator:*	**Chen**
Inj. Date / Time:	**26-Jul-2022 / 20:25**	*Run Time:*	**20.00**

No.	Time min	Peak Name	Peak Type	Area mAU*min	Height mAU	Amount n.a.
		TOTAL:		0.00	0.00	0.00

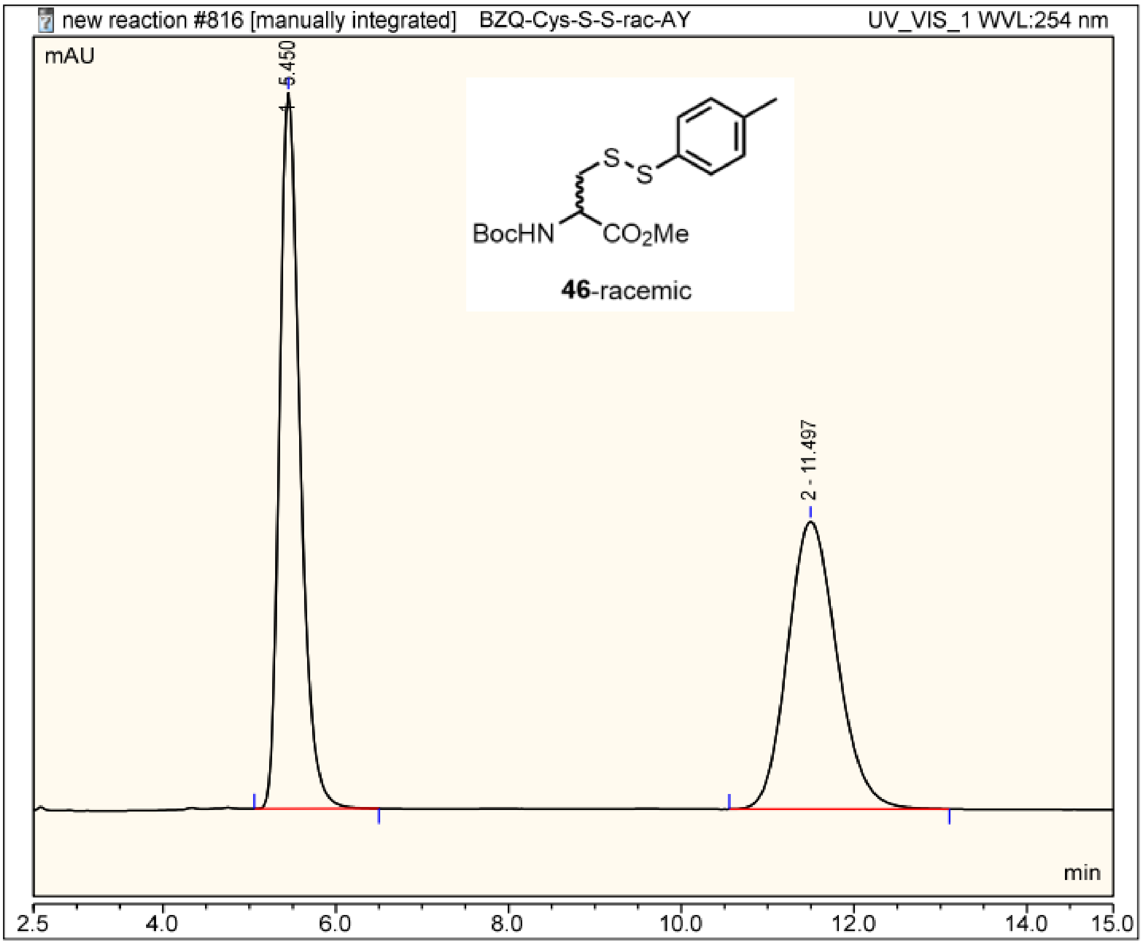

Integration Results							
No.	Peak Nam	Retention Time min	Area mAU*min	Height mAU	Relative Area %	Relative Height %	ee %
1		5.450	46.056	166.479	50.95	71.33	-1.90
2		11.497	44.343	66.929	49.05	28.67	
**Total:**			**90.399**	**233.409**	**100.00**	**100.00**	

Supplementary Figure 22. HPLC spectrum of 46-racemic. The ee value of **46**-racemic was determined by HPLC analysis on a CHIRALPAK® AY-H column (hexanes: isopropanol = 80:20, 1.0 mL/min, T = 30 ºC, 254 nm), TR = 5.45 min, 11.50 min.

Sample Name:	**BZQ-Cys-S-S-L**	*Inj. Vol.:*	**10.00**
Injection Type:	**Unknown**	*Dilution Factor:*	**1.0000**
Instrument Method:	**80-20 20 min 254nm**	*Operator:*	**Chen**
Inj. Date / Time:	**26-Jul-2022 / 15:28**	*Run Time:*	**20.00**

No.	Time min	Peak Name	Peak Type	Area mAU*min	Height mAU	Amount n.a.
		TOTAL:		0.00	0.00	0.00

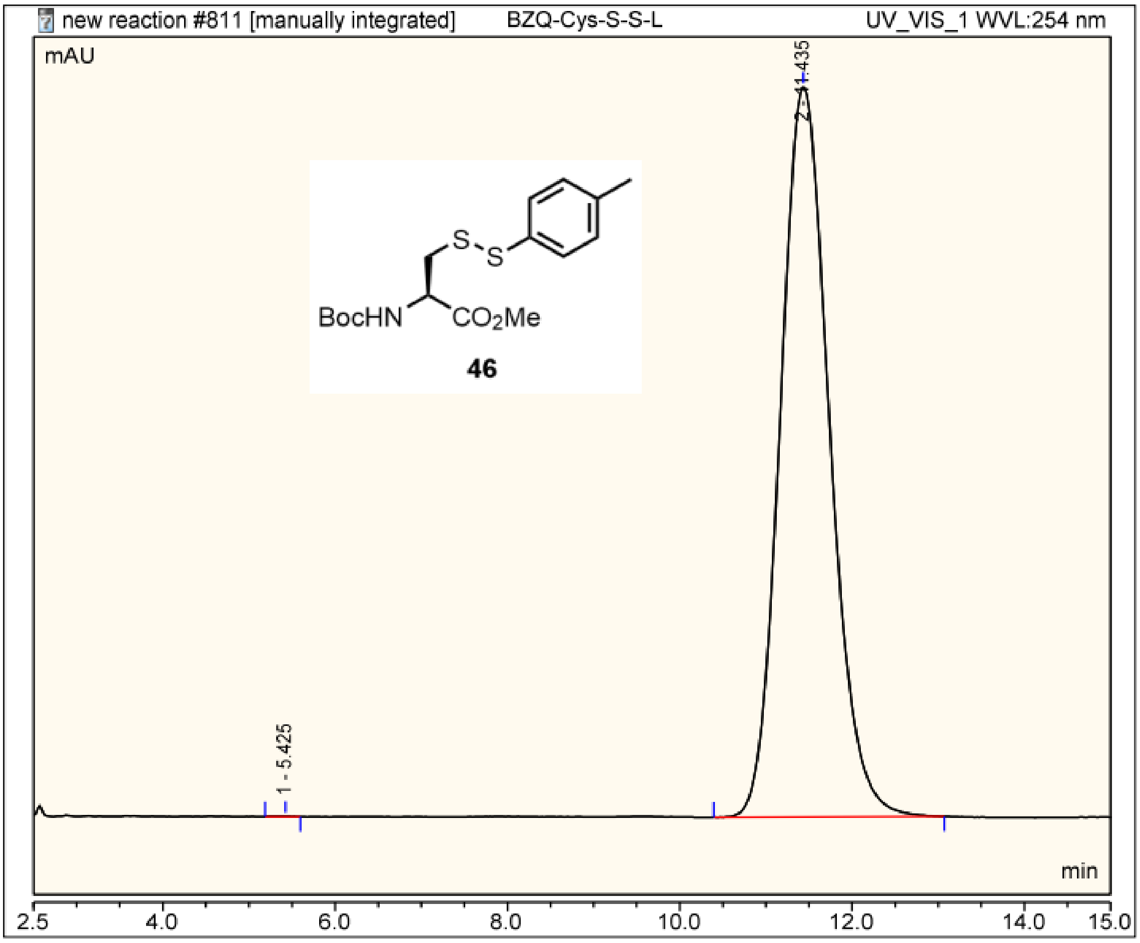

Integration Results

No.	Peak Nam	Retention Time min	Area mAU*min	Height mAU	Relative Area %	Relative Height %	ee %
1		5.425	0.005	0.025	0.01	0.05	99.97
2		11.435	31.016	46.801	99.99	99.95	
**Total:**			**31.021**	**46.827**	**100.00**	**100.00**	

Supplementary Figure 23. HPLC spectrum of 45. The ee value of **45** (>99.9% ee) was determined by HPLC analysis on a CHIRALPAK® AY-H column (hexanes: isopropanol = 80:20, 1.0 mL/min, T = 30 °C, 254 nm), TR = 5.43 min (minor), 11.44 min (major).

3.3 LC-MS of selected substrates

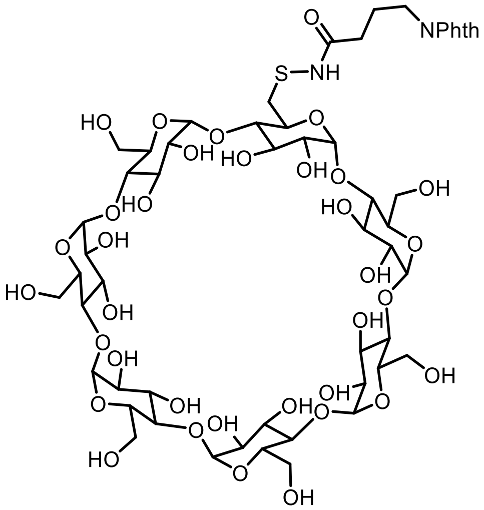

45

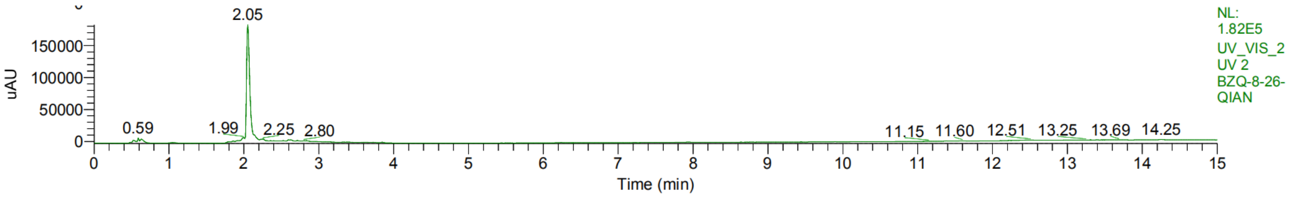

Supplementary Figure 24. UPLC analysis of compound **45** on a Thermo Scientific Hypersil GOLD C18 (1.9 µm, 2.1 × 100 mm) analytical column: t_R = 2.05 min, B/(A+B) = 5% to 95% for 0-12 min, λ = 254 nm. A: H_2O (0.1% HCOOH). B: MeCN (0.1% HCOOH).

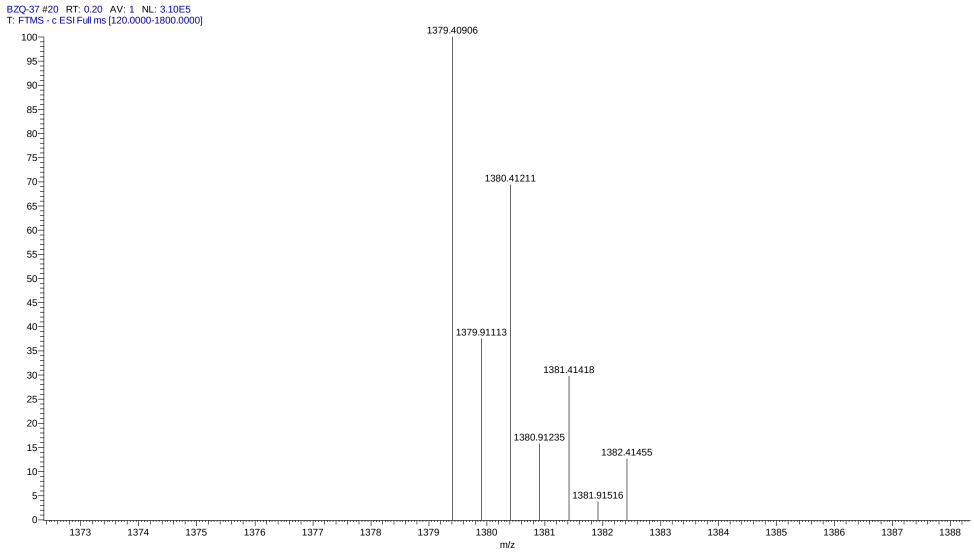

Supplementary Figure 25. HRMS (ESI) of compound **45**: *m/z* Calcd for $C_{54}H_{79}N_2O_{37}S^-$ [M-H]$^-$: 1379.4088, found: 1379.4091.

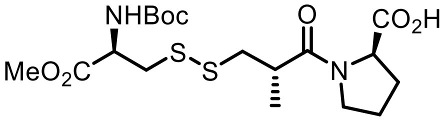

49

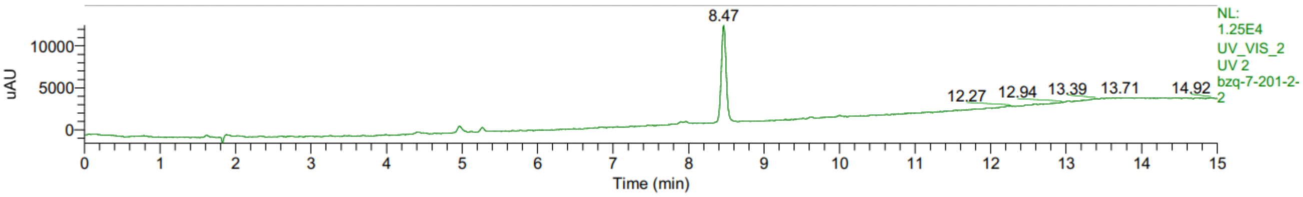

Supplementary Figure 26. UPLC analysis of compound **49** on a Thermo Scientific Acclaim^{TM} 120 C18 (5 µm, 4.6 × 250 mm) analytical column: t_R = 8.47 min, B/(A+B) = 5% to 95% for 0-12 min, λ = 254 nm. A: H_2O (0.1% HCOOH). B: MeCN (0.1% HCOOH). (NOTE: Disulfides compounds are visualized at 220 or 254 nm of UV)

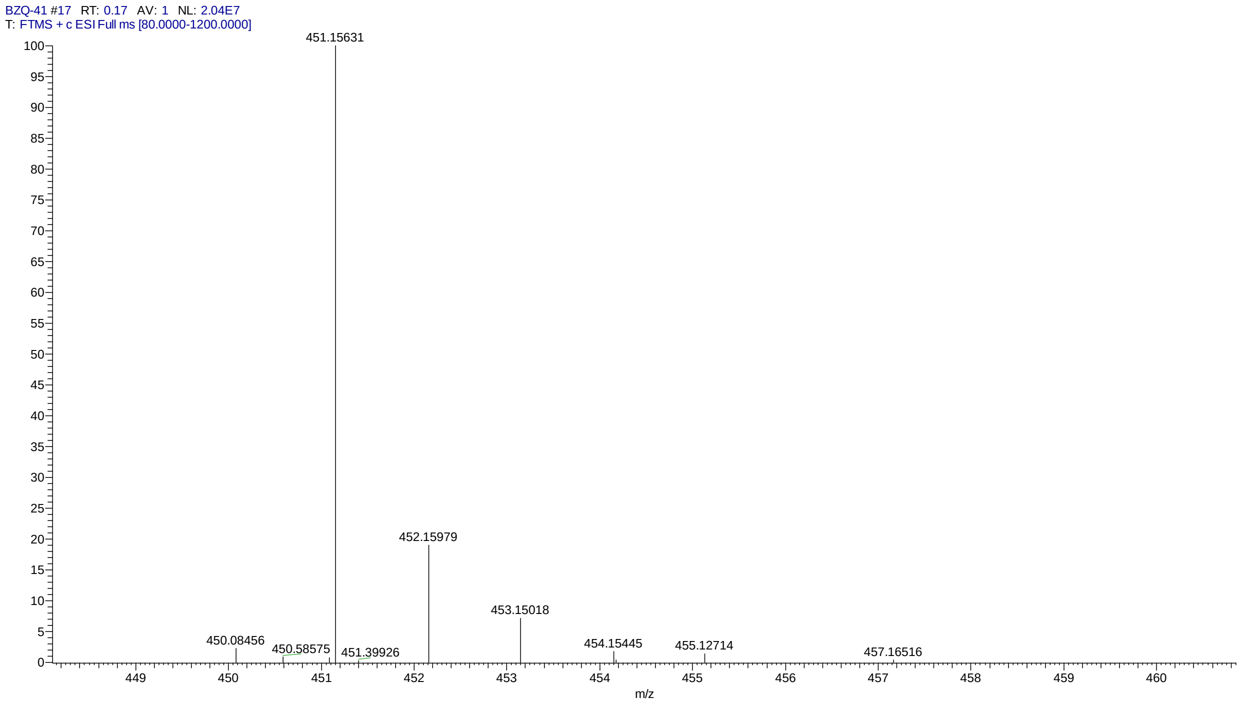

Supplementary Figure 27. HRMS (ESI) of compound **49**: *m/z* Calcd for $C_{18}H_{31}N_2O_7S_2^+$ $[M+H]^+$: 451.1567, found: 451.1563.

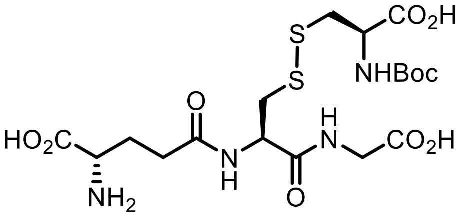

50

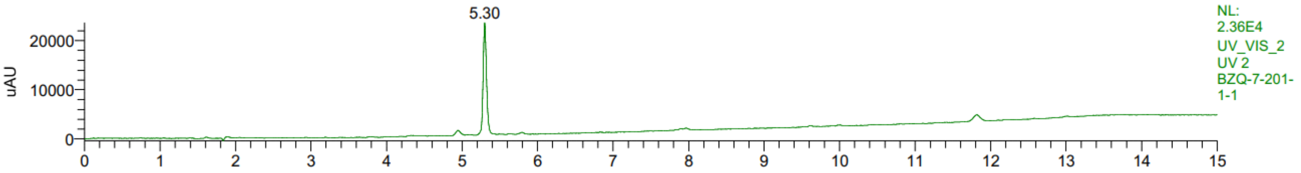

Supplementary Figure 28. UPLC analysis of compound **50** on a Thermo Scientific Acclaim™ 120 C18 (5 μm, 4.6 × 250 mm) analytical column: t_R = 5.30 min, B/(A+B) = 5% to 95% for 0-12 min, λ = 254 nm. A: H_2O (0.1% HCOOH). B: MeCN (0.1% HCOOH).

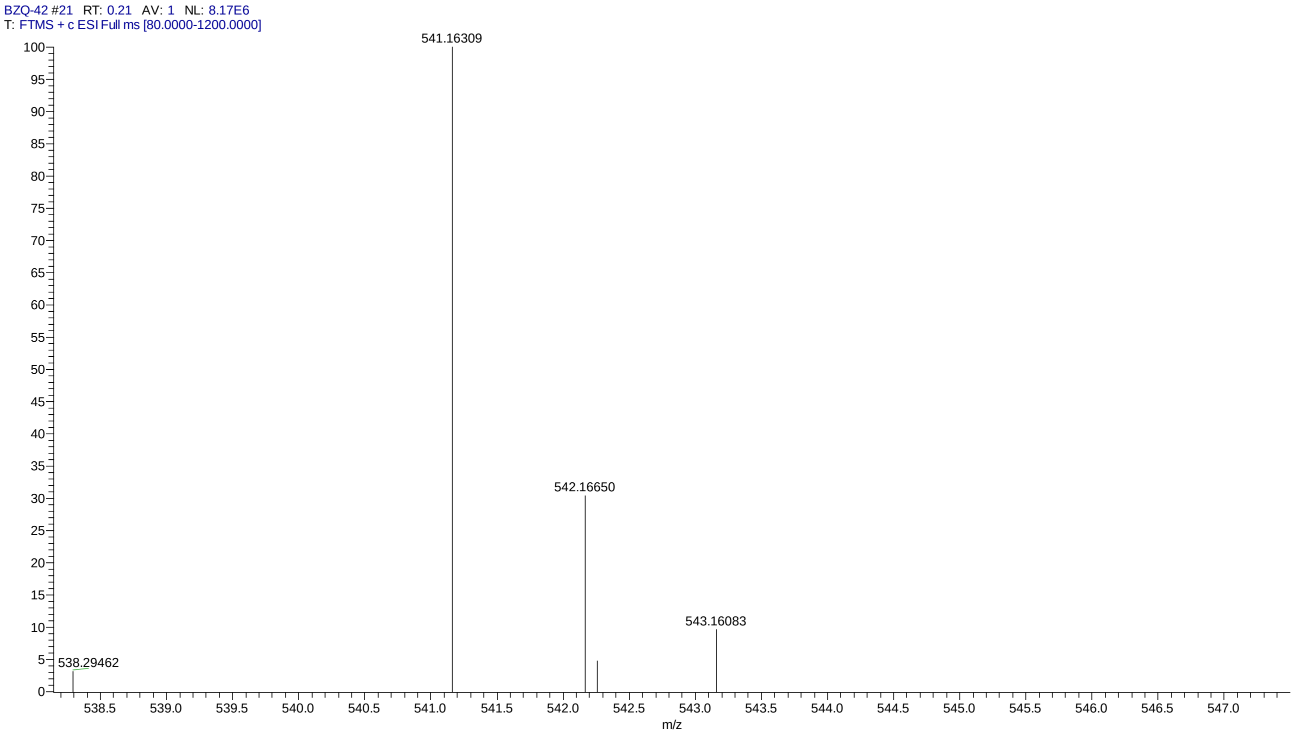

Supplementary Figure 29. HRMS (ESI) of compound **50**: *m/z* Calcd for $C_{19}H_{33}N_4O_{10}S_2^+$ $[M+H]^+$: 541.1633, found: 541.1631.

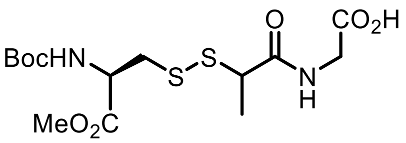

51

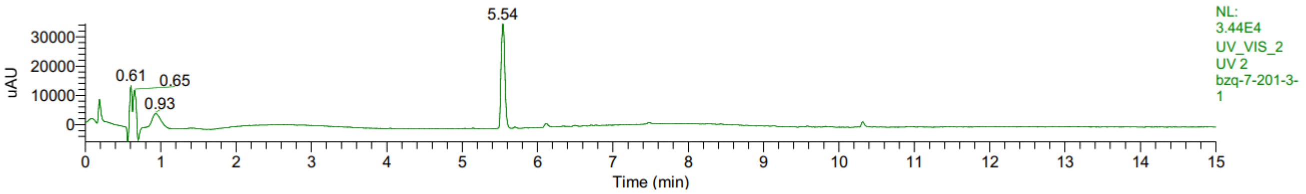

Supplementary Figure 30-1. UPLC analysis of compound **51** on a Thermo Scientific Hypersil GOLD C18 (1.9 μm, 2.1 × 100 mm) analytical column: t_R = 5.54 min, B/(A+B) = 5% to 95% for 0-12 min, λ = 254 nm. A: H_2O (0.1% HCOOH). B: MeCN (0.1% HCOOH).

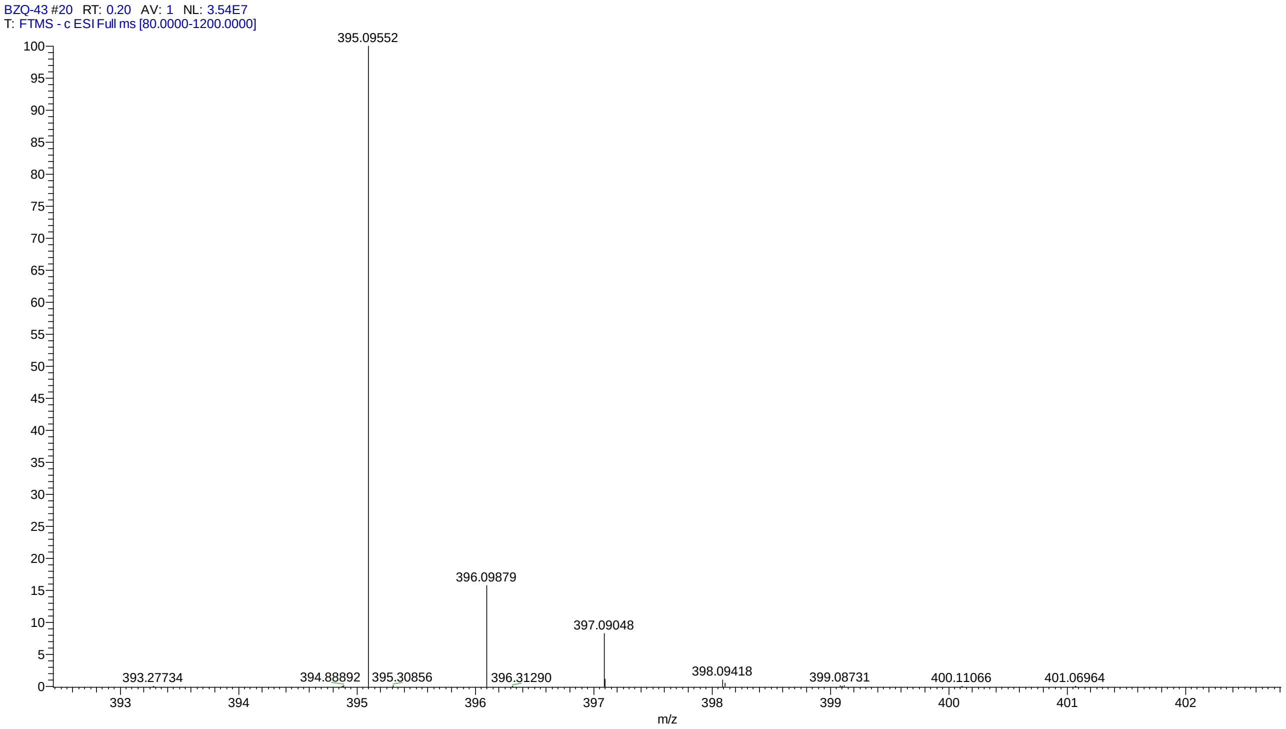

Supplementary Figure 30-2. HRMS (ESI) of compound **51**: *m/z* Calcd for $C_{14}H_{23}N_2O_7S_2^-$ $[M-H]^-$: 395.0952, found: 395.0955.

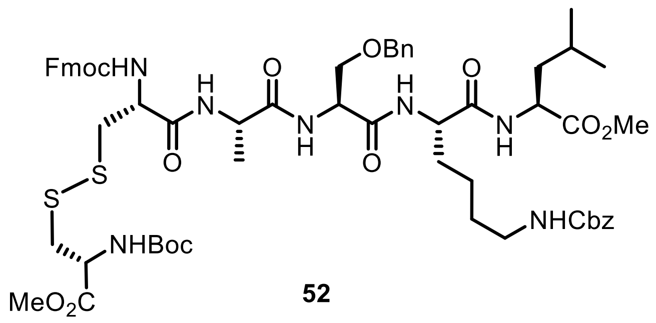

52

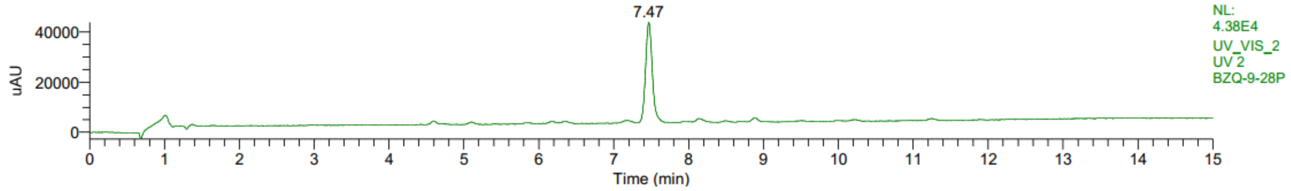

Supplementary Figure 31-1. UPLC analysis of compound **52** on a Thermo Scientific Acclaim™ 120 C18 (5 µm, 4.6 × 250 mm) analytical column: t_R = 7.47 min, B/(A+B) = 70% to 95% for 0-12 min, λ = 254 nm. A: H_2O (0.1% HCOOH). B: MeCN (0.1% HCOOH).

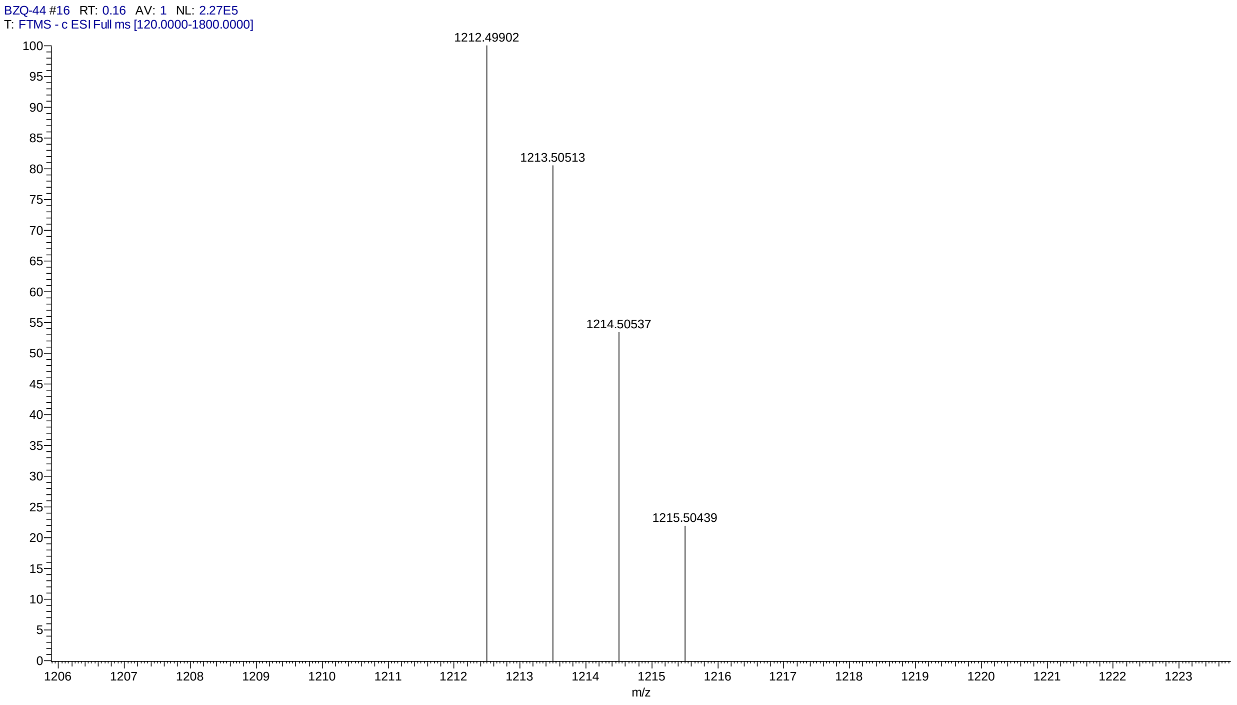

Supplementary Figure 31-2. HRMS (ESI) of compound **52**: *m/z* Calcd for $C_{61}H_{78}N_7O_{15}S_2^-$ $[M-H]^-$: 1212.5003, found: 1212.4990.

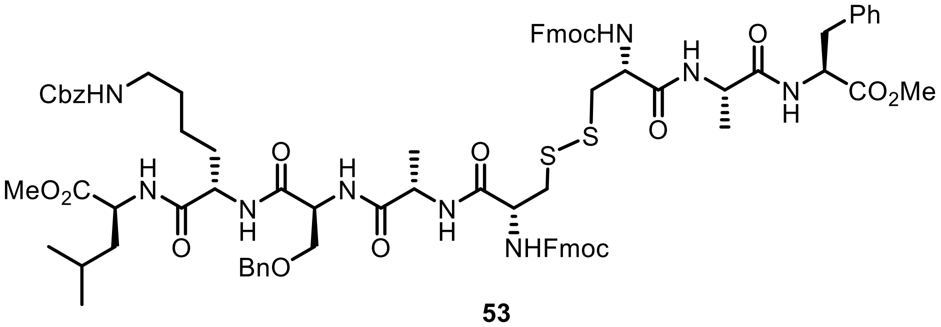

53

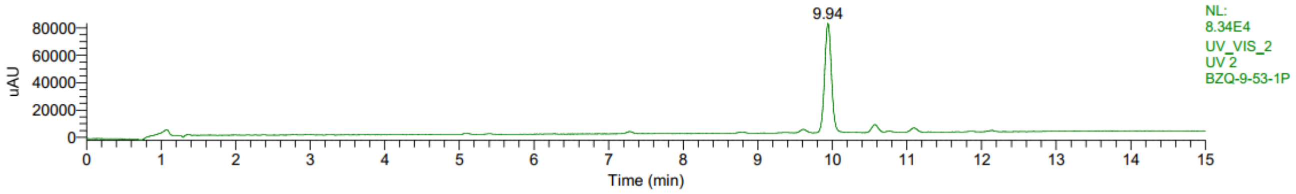

Supplementary Figure 32. UPLC analysis of compound **53** on a Thermo Scientific Acclaim™ 120 C18 (5 µm, 4.6 × 250 mm) analytical column: t_R = 9.94 min, B/(A+B) = 70% to 95% for 0-12 min, λ = 254 nm. A: H_2O (0.1% HCOOH). B: MeCN (0.1% HCOOH).

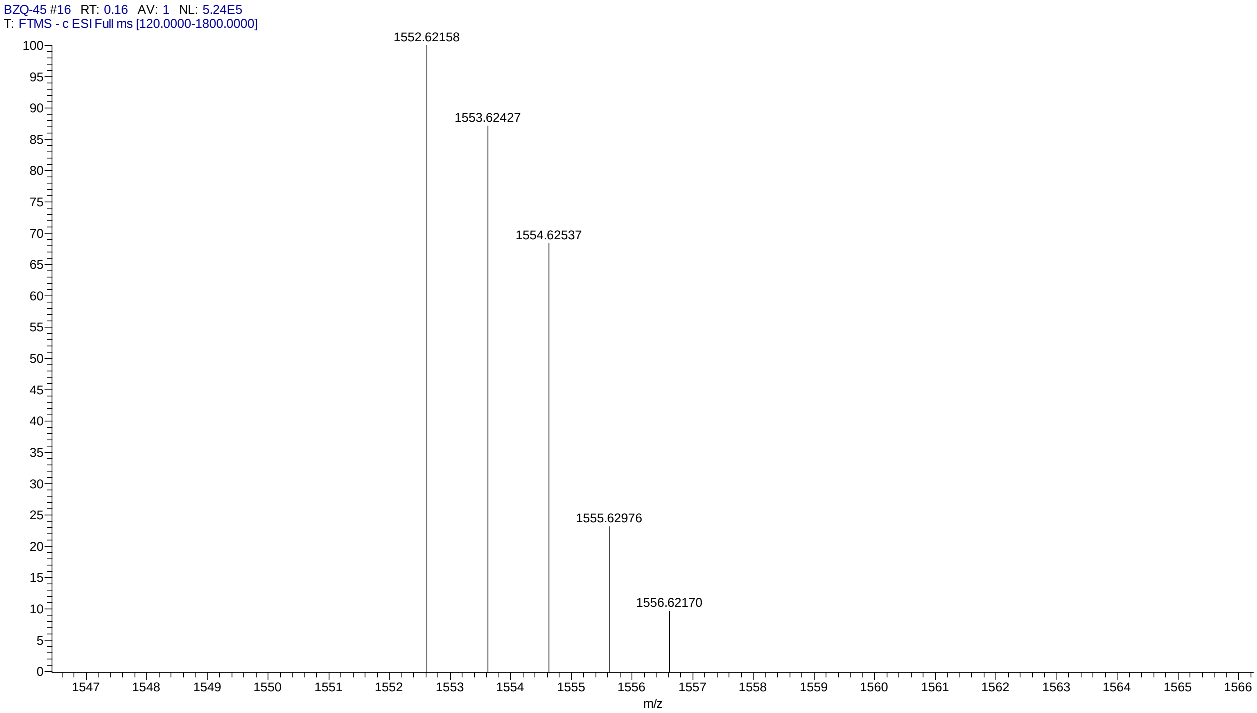

Supplementary Figure 33. HRMS (ESI) of compound **53**: *m/z* Calcd for $C_{83}H_{94}N_9O_{17}S_2^-$ [M-H]$^-$: 1552.6215, found: 1552.6216.

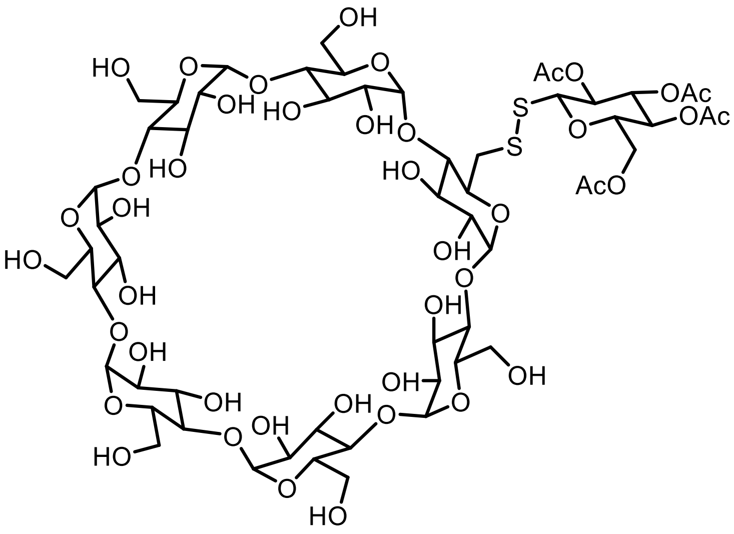

54

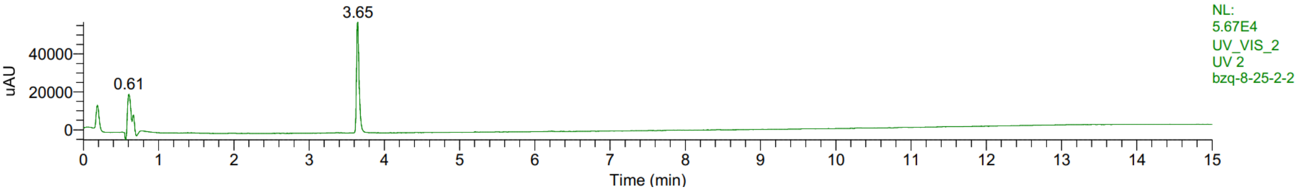

Supplementary Figure 34. UPLC analysis of compound **54** on a Thermo Scientific Hypersil GOLD C18 (1.9 μm, 2.1 × 100 mm) analytical column: t_R = 3.65 min, B/(A+B) = 5% to 95% for 0-12 min, λ = 254 nm. A: H_2O (0.1% HCOOH). B: MeCN (0.1% HCOOH).

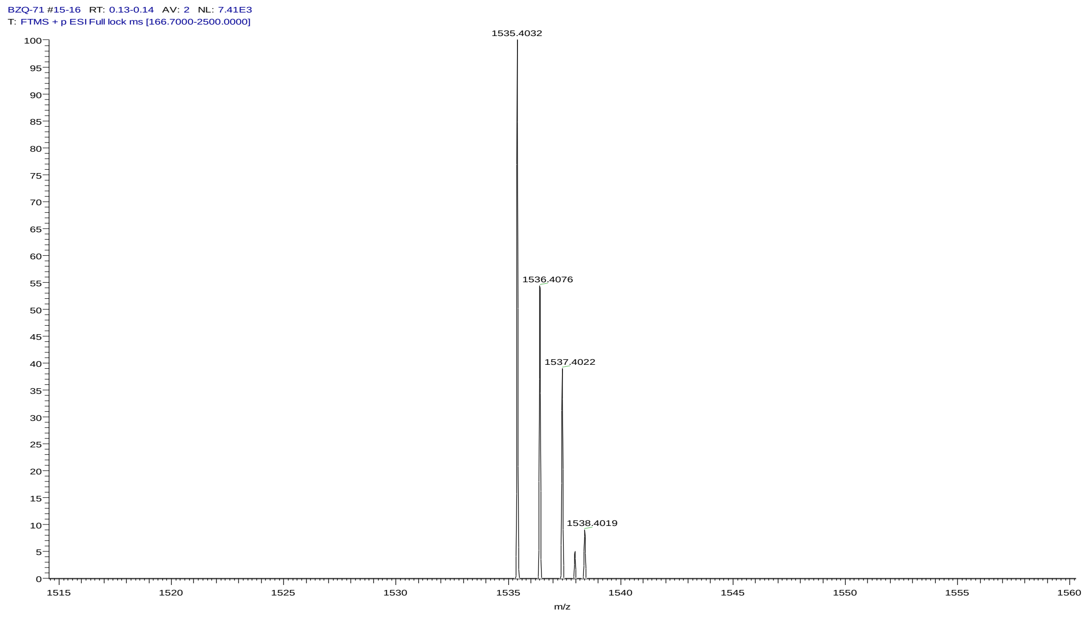

Supplementary Figure 35. HRMS (ESI) of compound **54**: *m/z* Calcd for $C_{56}H_{88}NaO_{43}S_2^+$ $[M+Na]^+$: 1535.4033, found: 1535.4032.

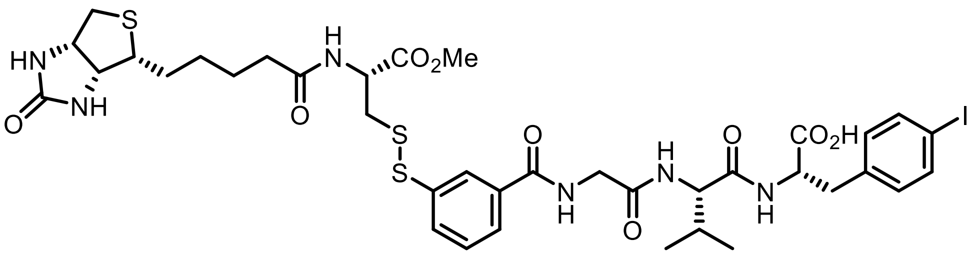

55

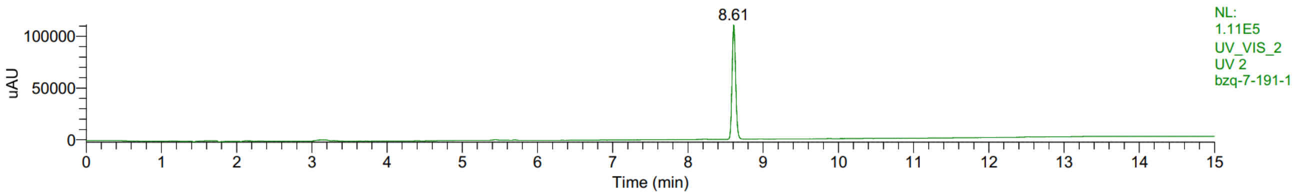

Supplementary Figure 36. UPLC analysis of compound **50** on a Thermo Scientific Acclaim™ 120 C18 (5 µm, 4.6 × 250 mm) analytical column: t_R = 8.61 min, B/(A+B) = 5% to 95% for 0-12 min, λ = 254 nm. A: H_2O (0.1% HCOOH). B: MeCN (0.1% HCOOH).

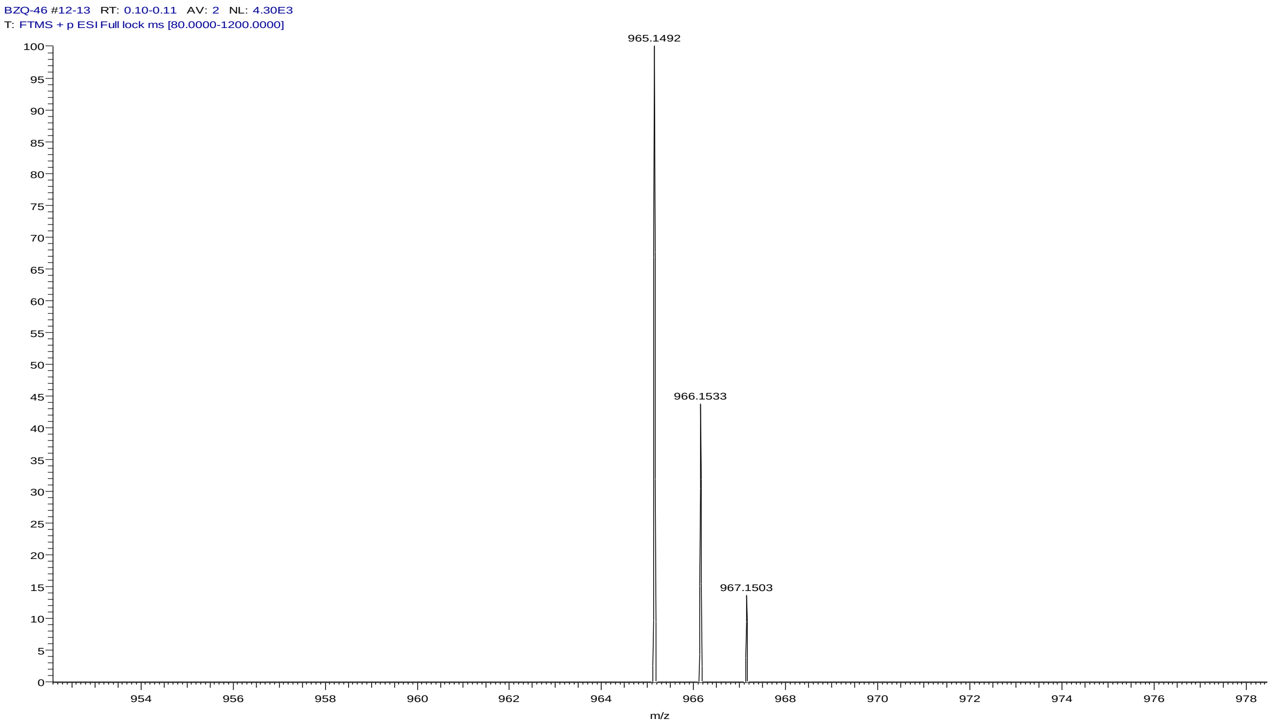

Supplementary Figure 37. HRMS (ESI) of compound **55**: *m/z* Calcd for $C_{37}H_{47}IN_6NaO_9S_3^+$ $[M+Na]^+$: 965.1504, found: 965.1492.

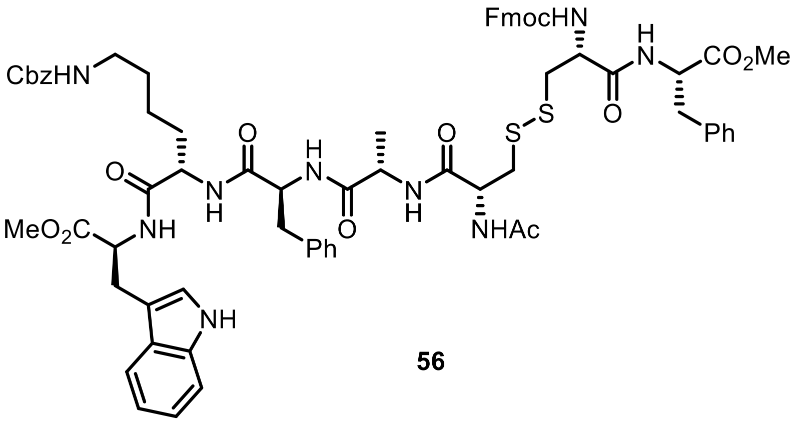

56

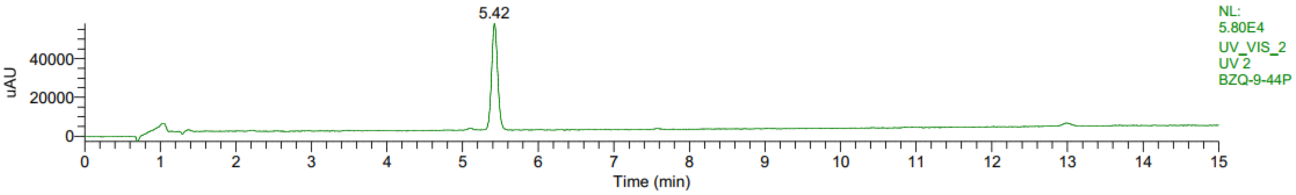

Supplementary Figure 38. UPLC analysis of compound **56** on a Thermo Scientific Acclaim™ 120 C18 (5 µm, 4.6 × 250 mm) analytical column: t_R = 5.42 min, B/(A+B) = 70% to 95% for 0-12 min, λ = 254 nm. A: H_2O (0.1% HCOOH). B: MeCN (0.1% HCOOH).

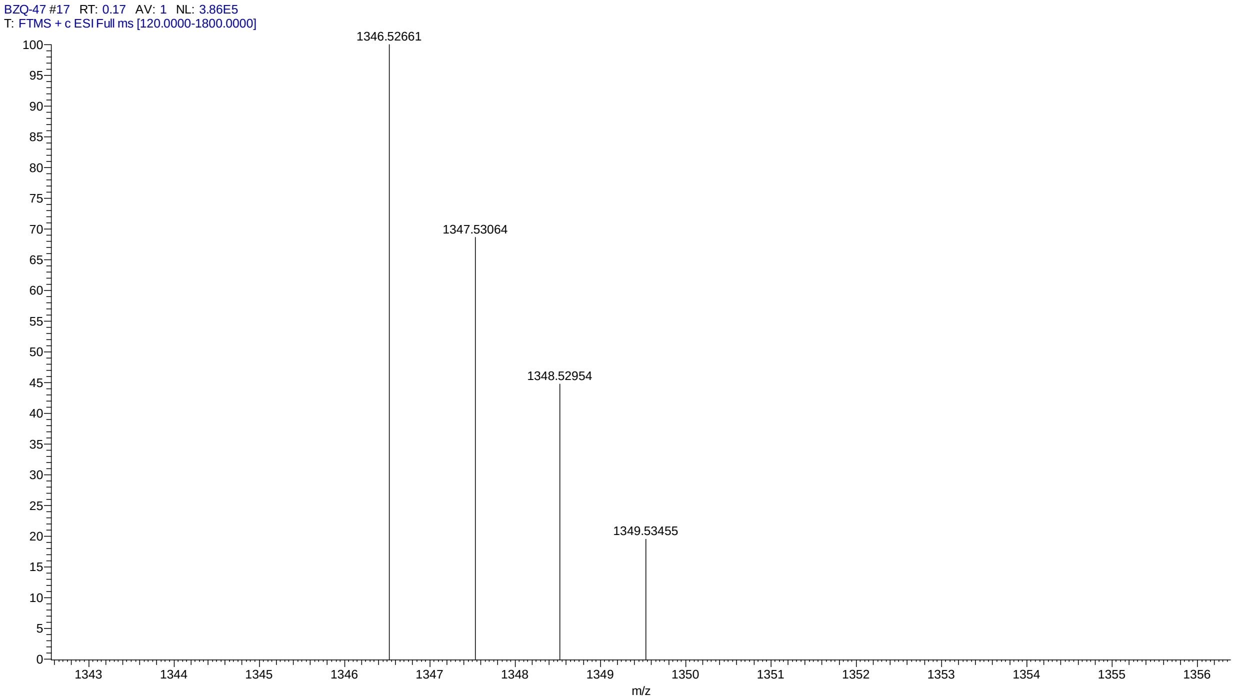

Supplementary Figure 39. HRMS (ESI) of compound **56**: *m/z* Calcd for $C_{71}H_{80}N_9O_{14}S_2^+$ $[M+H]^+$: 1346.5261, found: 1346.5266.

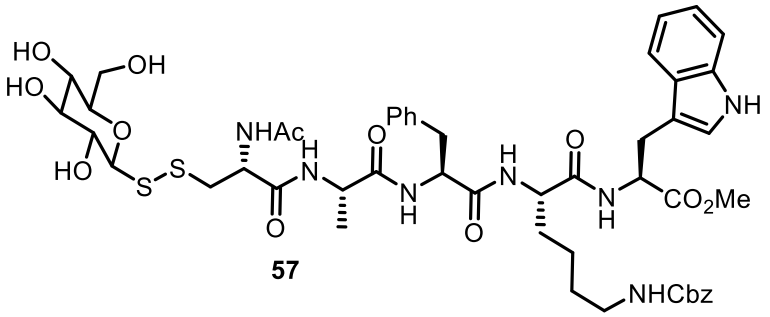

57

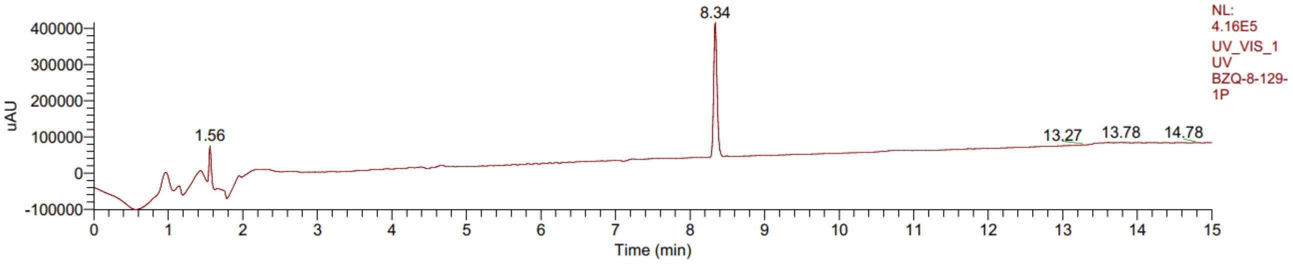

Supplementary Figure 40. UPLC analysis of compound **57** on a Thermo Scientific Acclaim™ 120 C18 (5 µm, 4.6 × 250 mm) analytical column: t_R = 8.34 min, B/(A+B) = 5% to 95% for 0-12 min, λ = 220 nm. A: H_2O (0.1% HCOOH). B: MeCN (0.1% HCOOH).

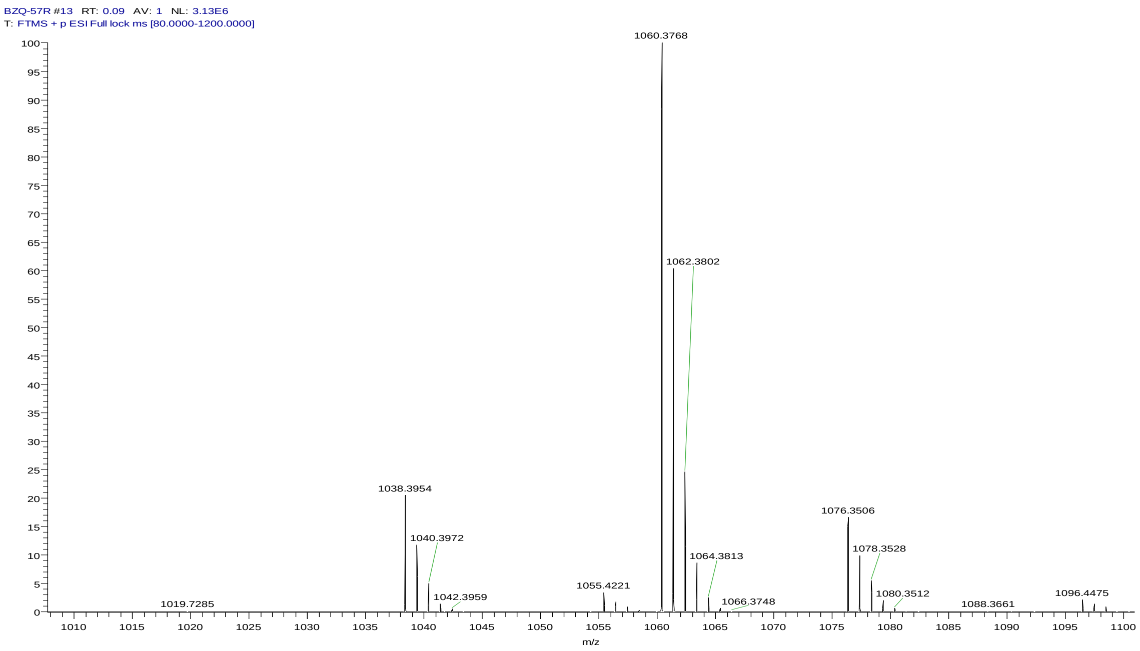

Supplementary Figure 41. HRMS (ESI) of compound **57**: *m/z* Calcd for $C_{49}H_{63}N_7NaO_{14}S_2^+$ $[M+Na]^+$: 1060.3767, found: 1060.3768.

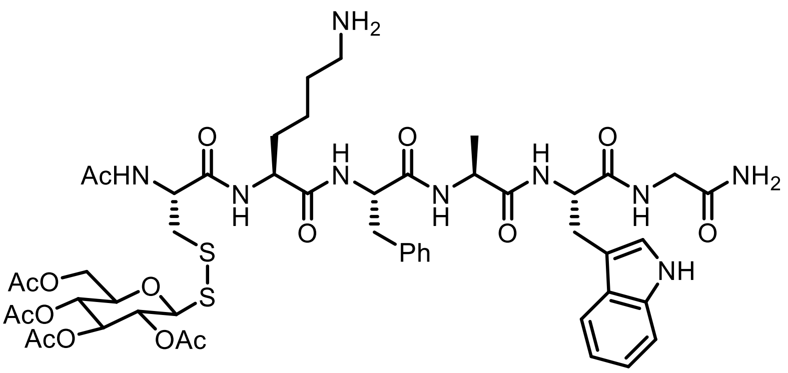

58

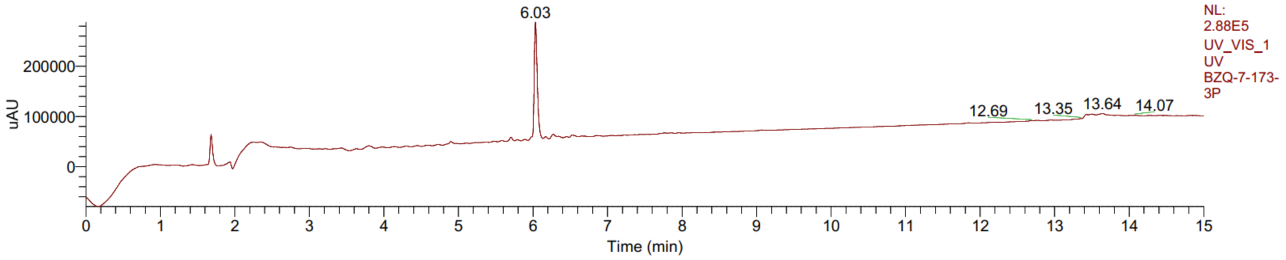

Supplementary Figure 42. UPLC analysis of compound **58** on a Thermo Scientific Acclaim™ 120 C18 (5 μm, 4.6 × 250 mm) analytical column: t_R = 6.03 min, B/(A+B) = 5% to 95% for 0-12 min, λ = 220 nm. A: H_2O (0.1% HCOOH). B: MeCN (0.1% HCOOH).

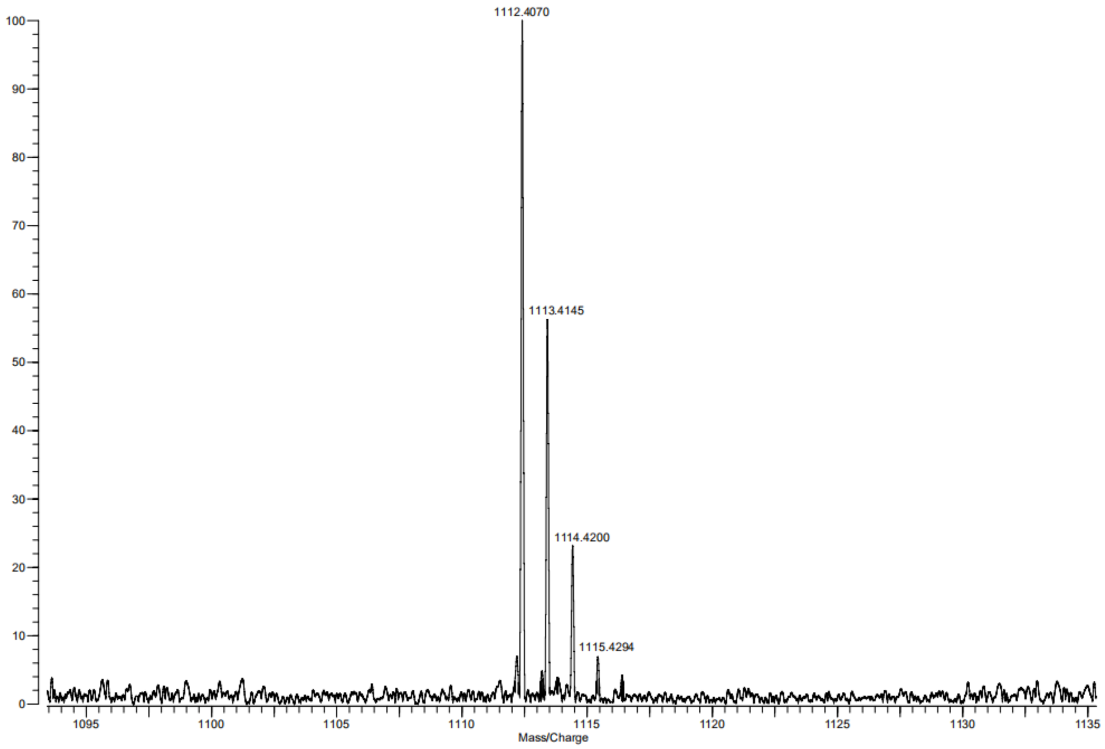

Supplementary Figure 43. HRMS (ESI) of compound **58**: *m/z* Calcd for $C_{50}H_{66}N_9O_{16}S_2^-$ [M-H]$^-$: 1112.4074, found: 1112.4070.

3.4 NMR spectra

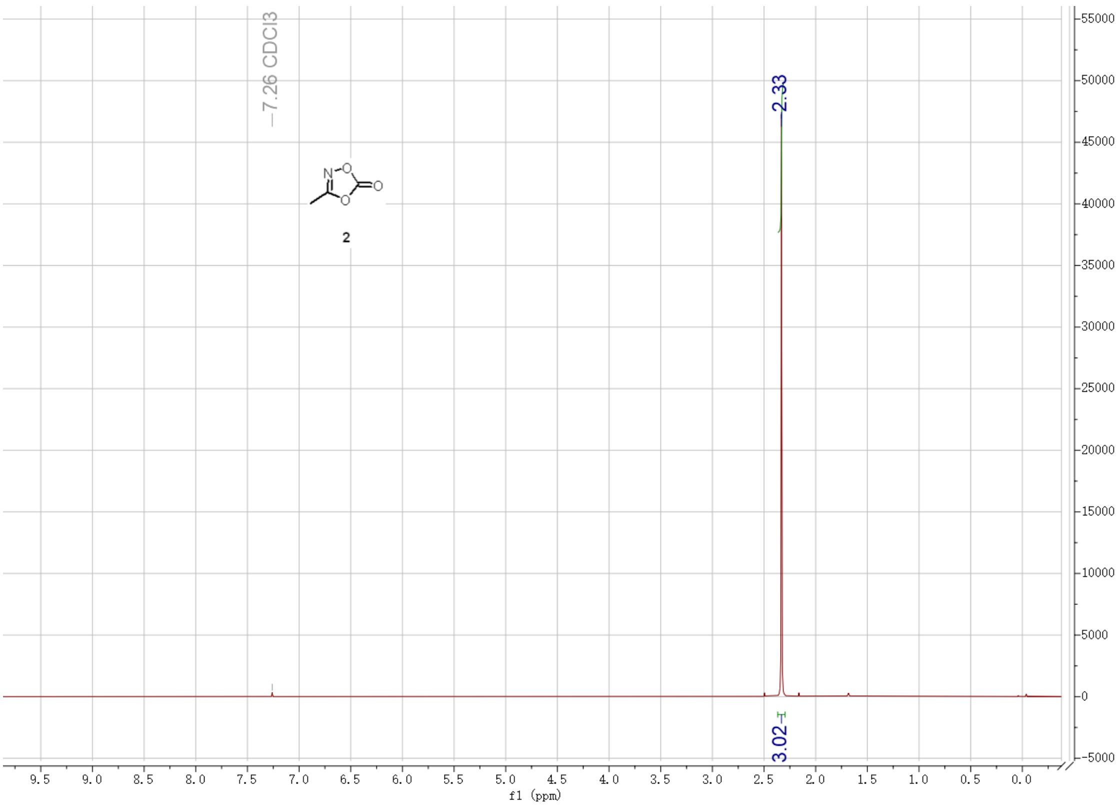

Supplementary Figure 44. ^{1}H NMR (400 MHz, $CDCl_3$, 293 K) spectrum of **2**.

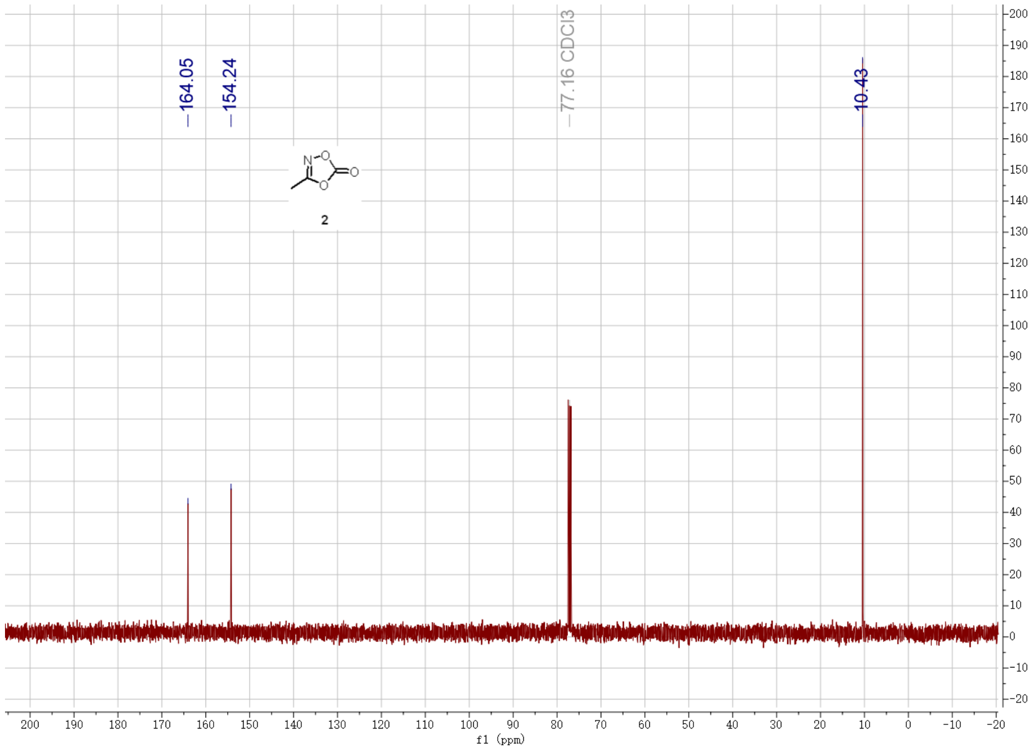

Supplementary Figure 45. ^{13}C NMR (101 MHz, $CDCl_3$, 293 K) spectrum of **2**.

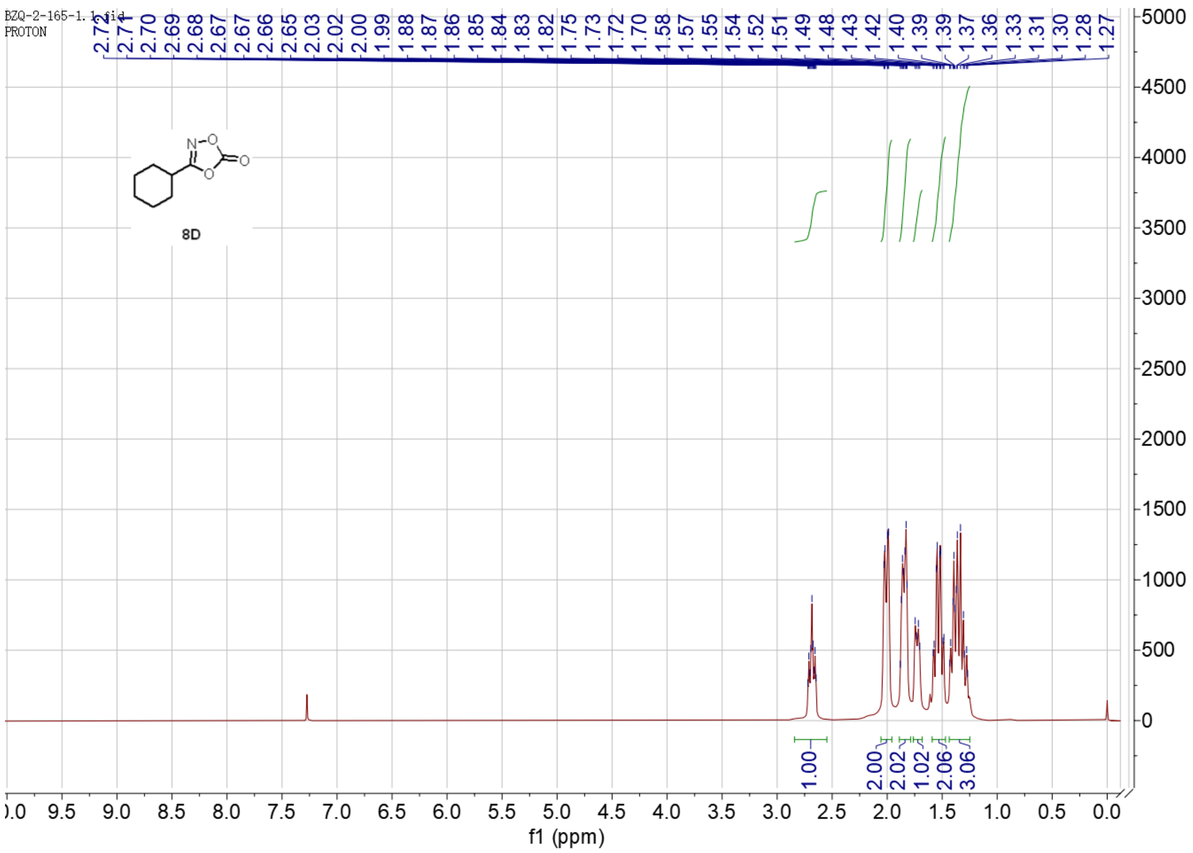

Supplementary Figure 46. ^{1}H NMR (400 MHz, $CDCl_3$, 293 K) spectrum of **8D**.

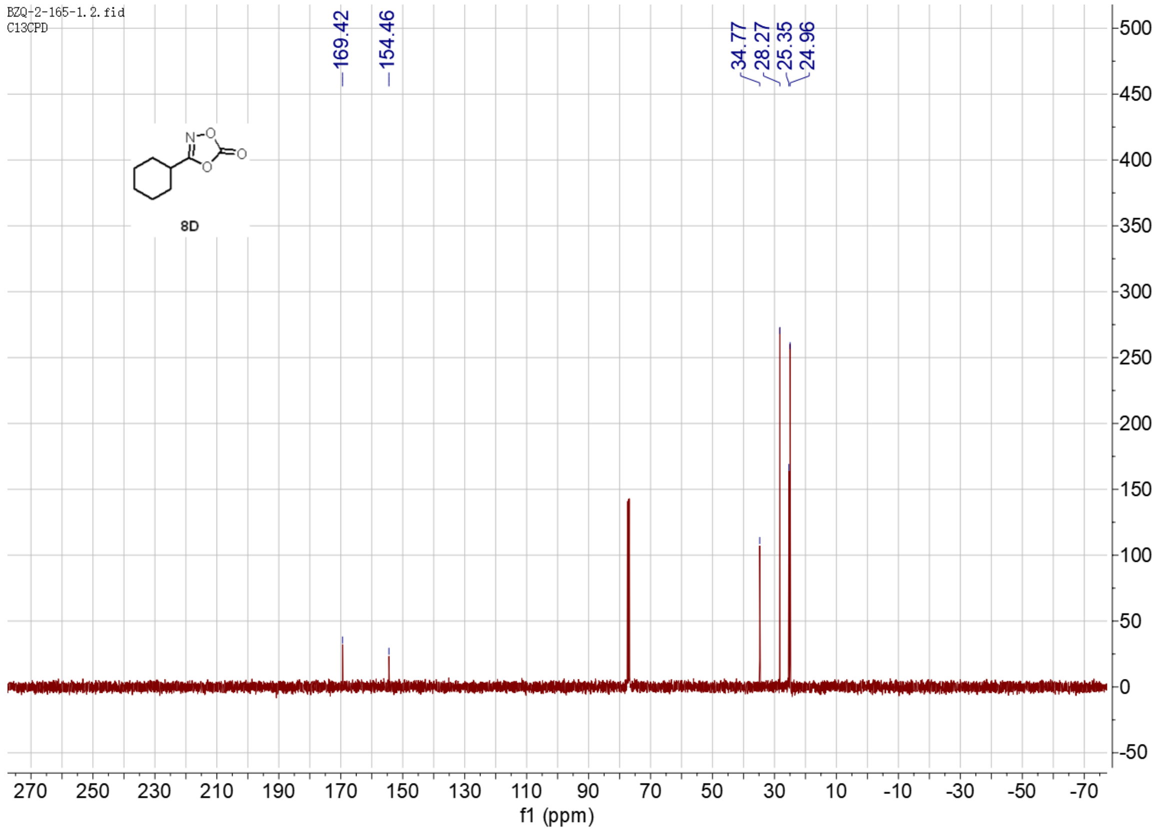

Supplementary Figure 47. ^{13}C NMR (101 MHz, $CDCl_3$, 293 K) spectrum of **8D**.

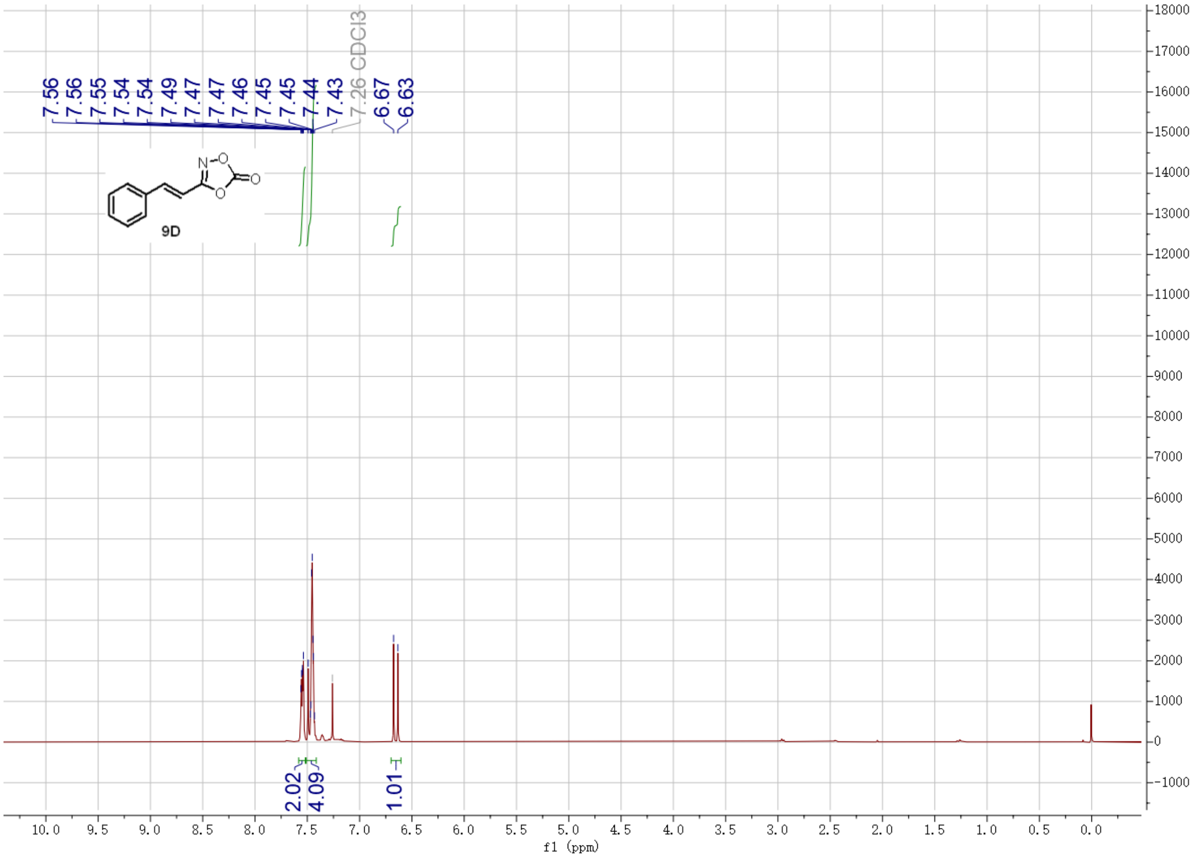

Supplementary Figure 48. ^{1}H NMR (400 MHz, $CDCl_3$, 293 K) spectrum of **9D**.

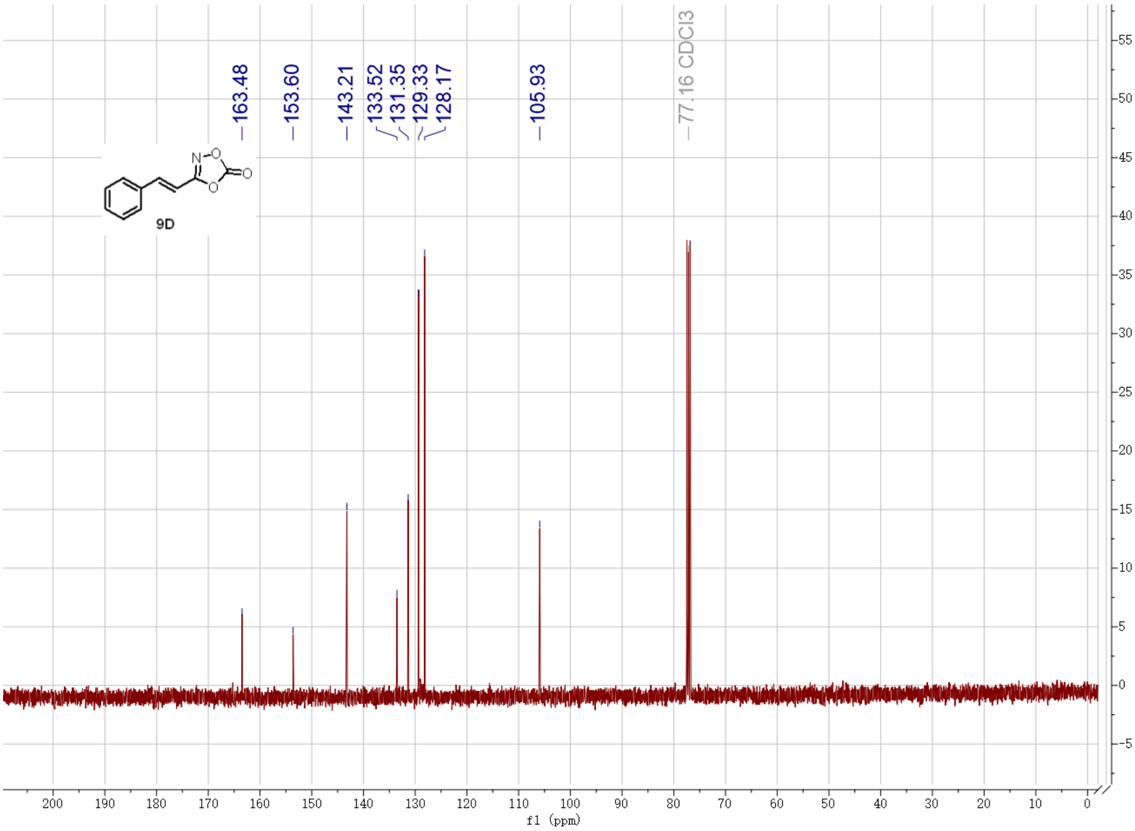

Supplementary Figure 49. ^{13}C NMR (101 MHz, $CDCl_3$, 293 K) spectrum of **9D**.

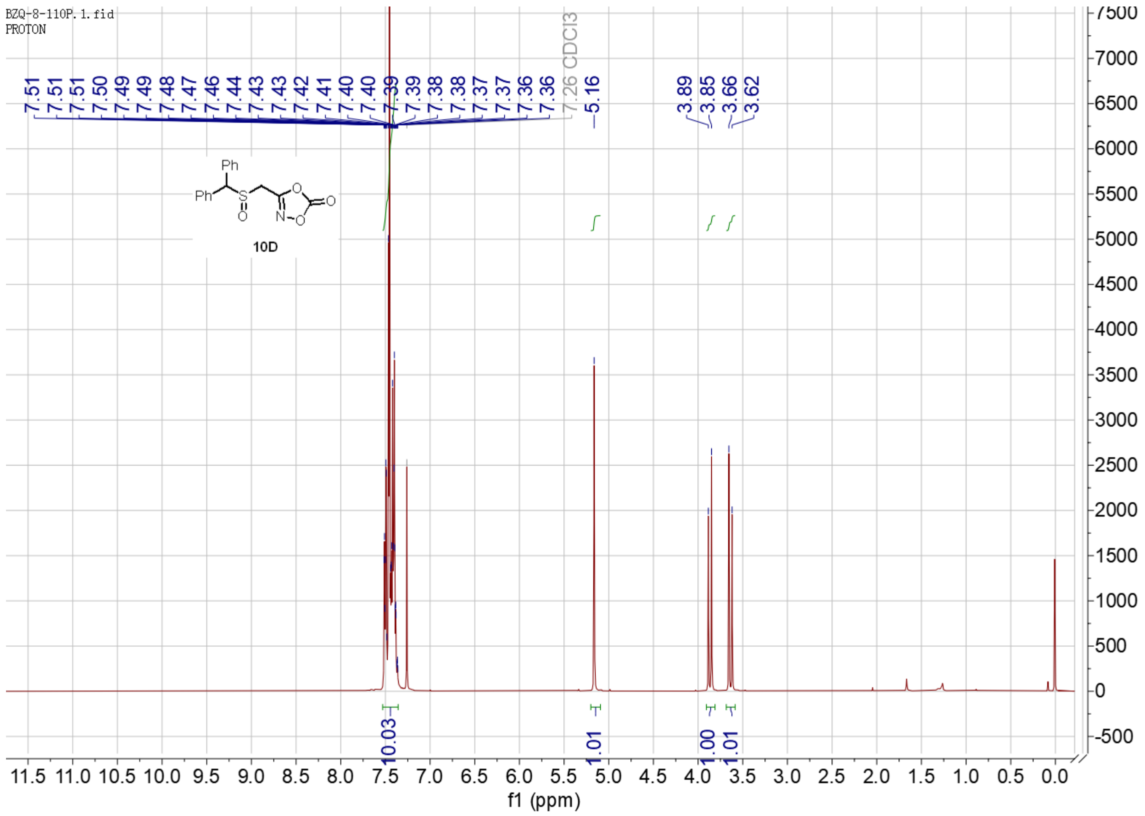

Supplementary Figure 50. ^{1}H NMR (400 MHz, $CDCl_3$, 293 K) spectrum of **10D**.

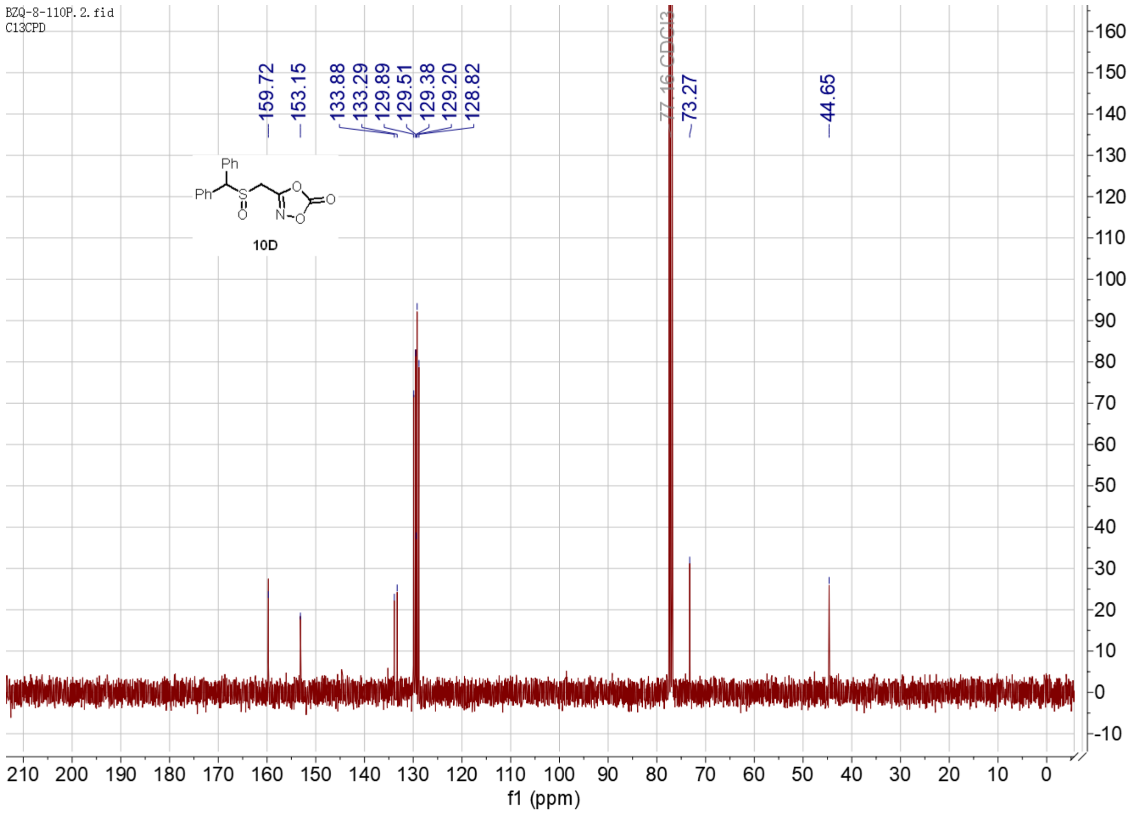

Supplementary Figure 51. ^{13}C NMR (101 MHz, $CDCl_3$, 293 K) spectrum of **10D**.

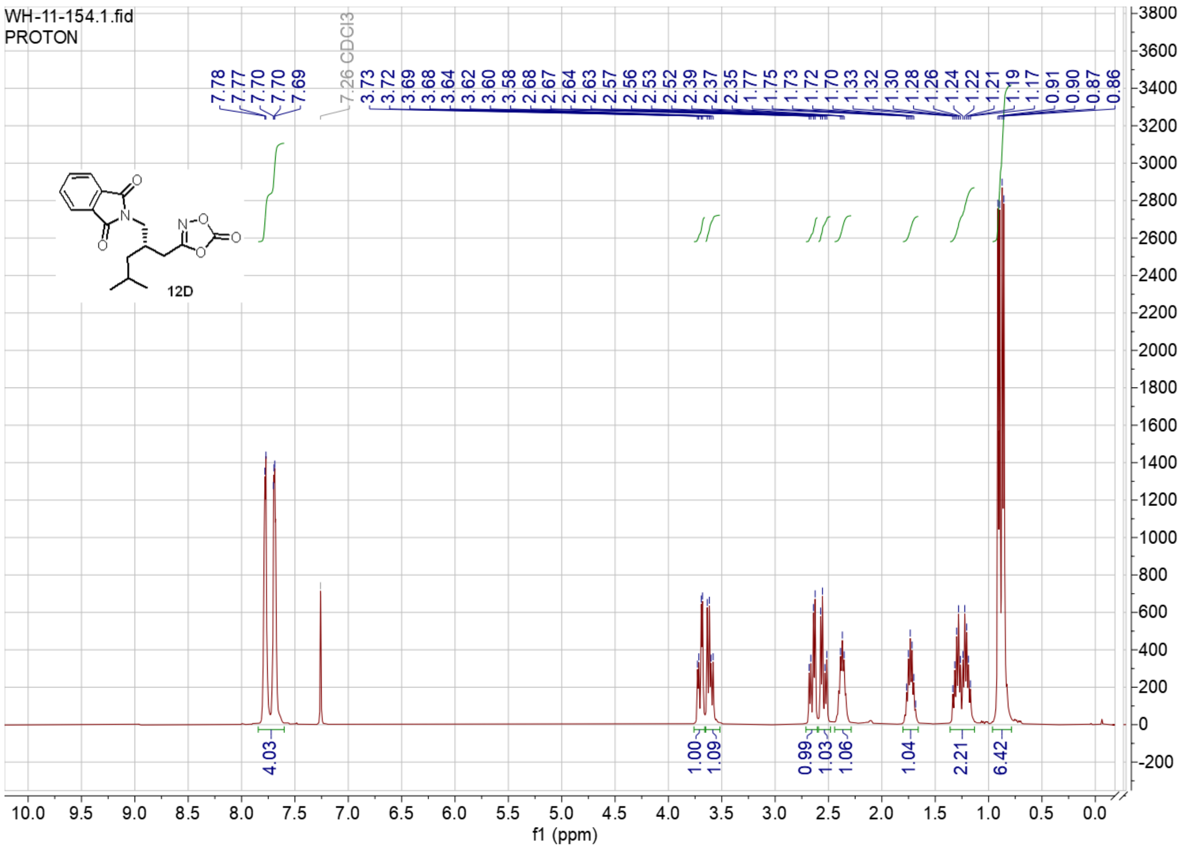

Supplementary Figure 52. ^{1}H NMR (400 MHz, $CDCl_3$, 293 K) spectrum of **12D**.

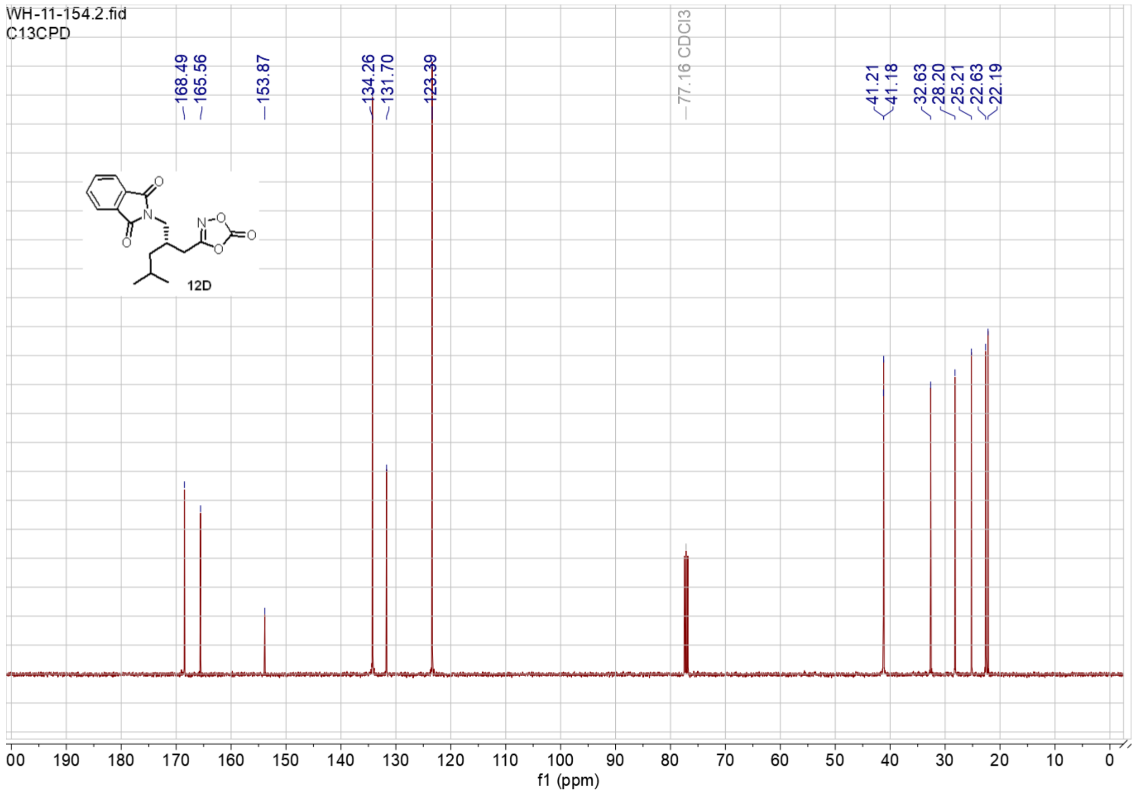

Supplementary Figure 53. ^{13}C NMR (101 MHz, $CDCl_3$, 293 K) spectrum of **12D**.

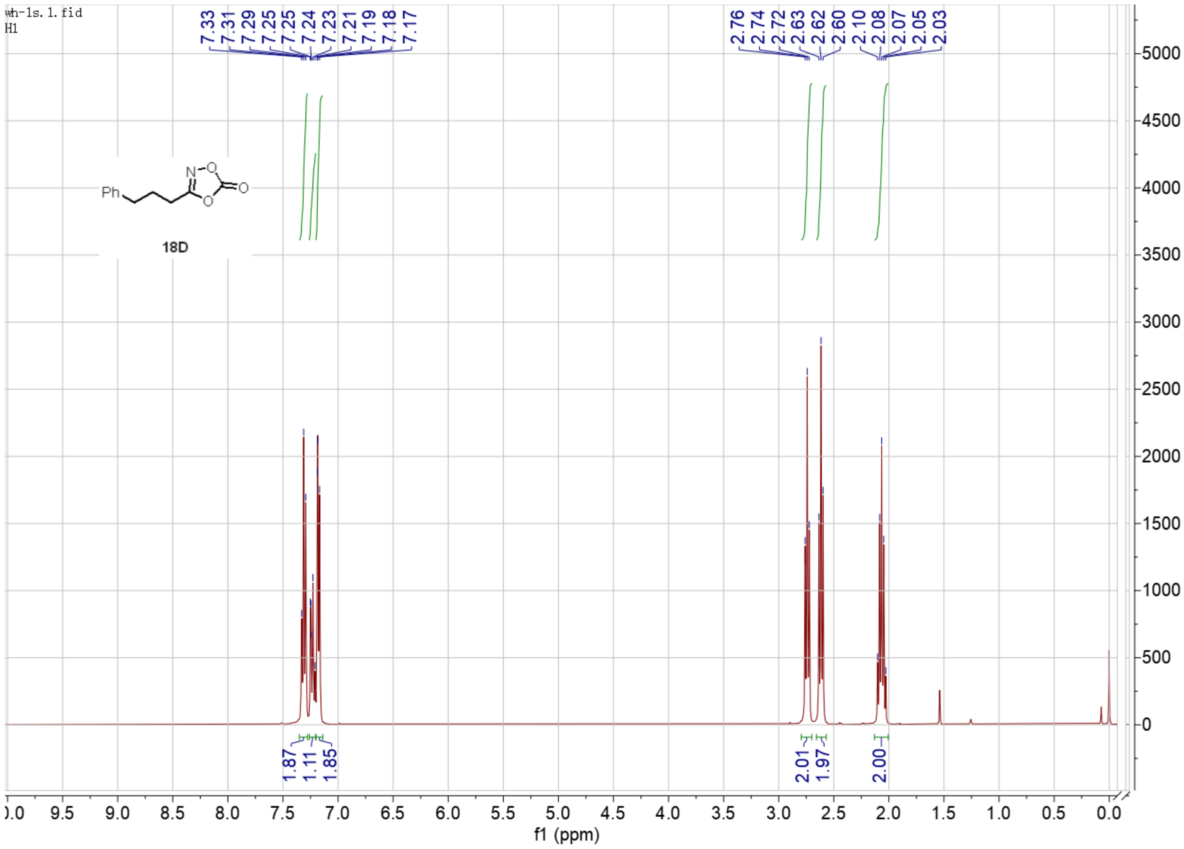

Supplementary Figure 54. ^{1}H NMR (400 MHz, $CDCl_3$, 293 K) spectrum of **18D**.

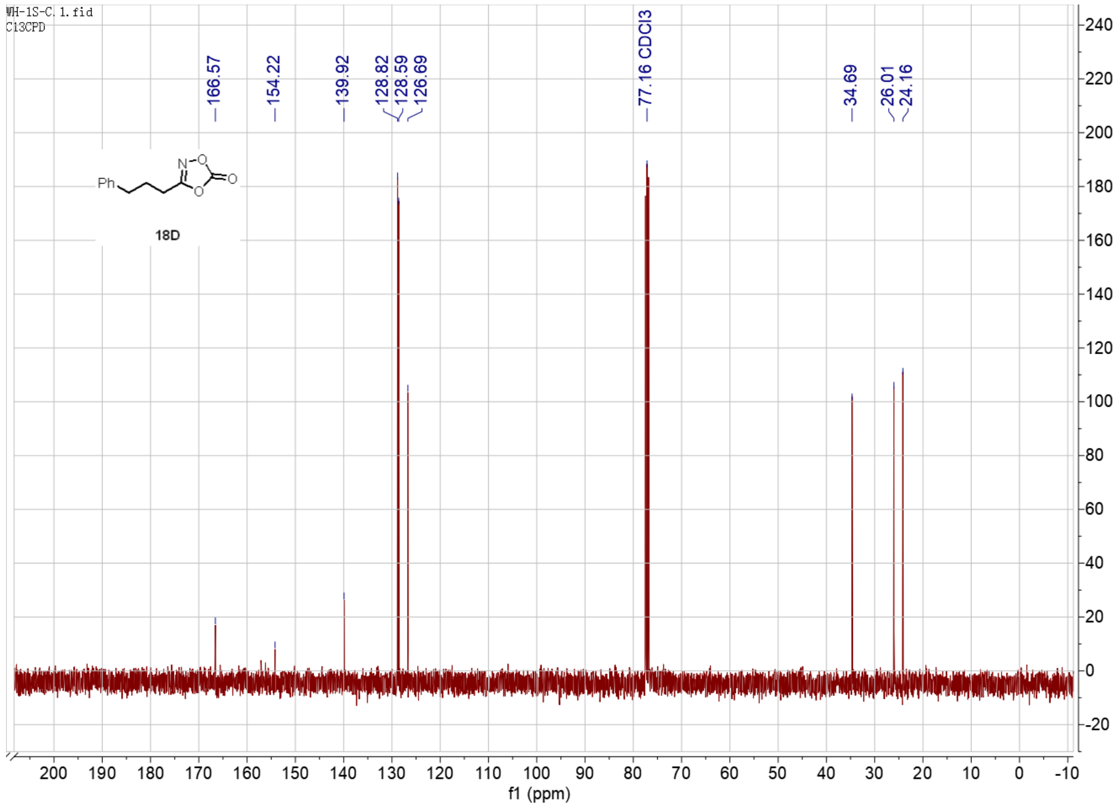

Supplementary Figure 55. ^{13}C NMR (101 MHz, $CDCl_3$, 293 K) spectrum of **18D**.

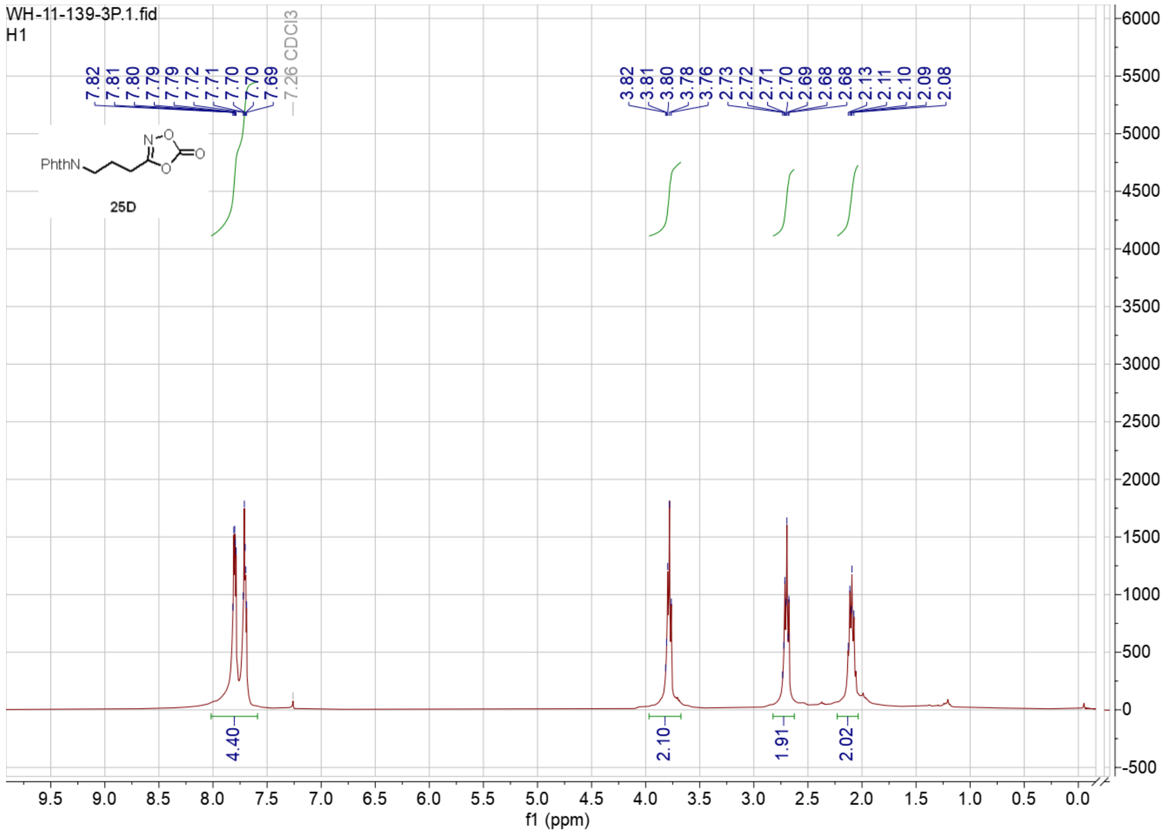

Supplementary Figure 56. ^{1}H NMR (400 MHz, $CDCl_3$, 293 K) spectrum of **25D**.

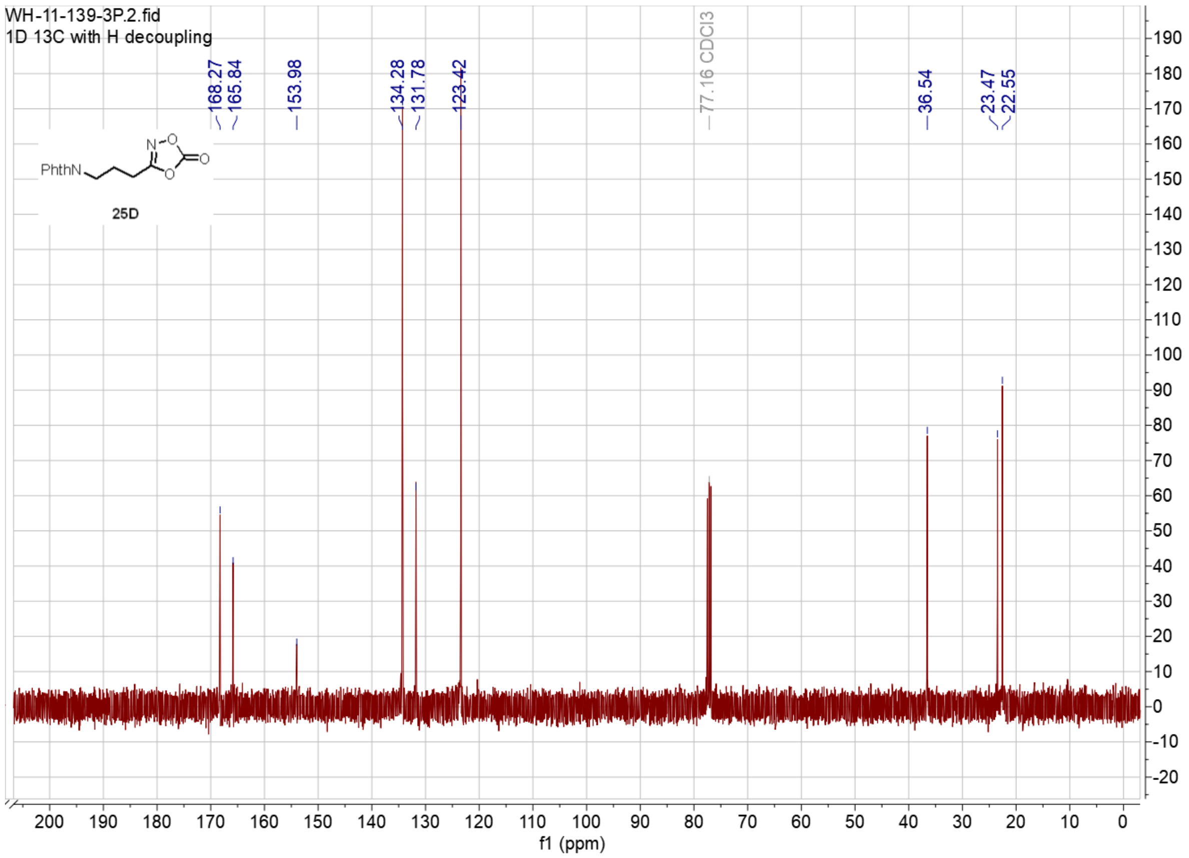

Supplementary Figure 57. ^{13}C NMR (101 MHz, $CDCl_3$, 293 K) spectrum of **25D**.

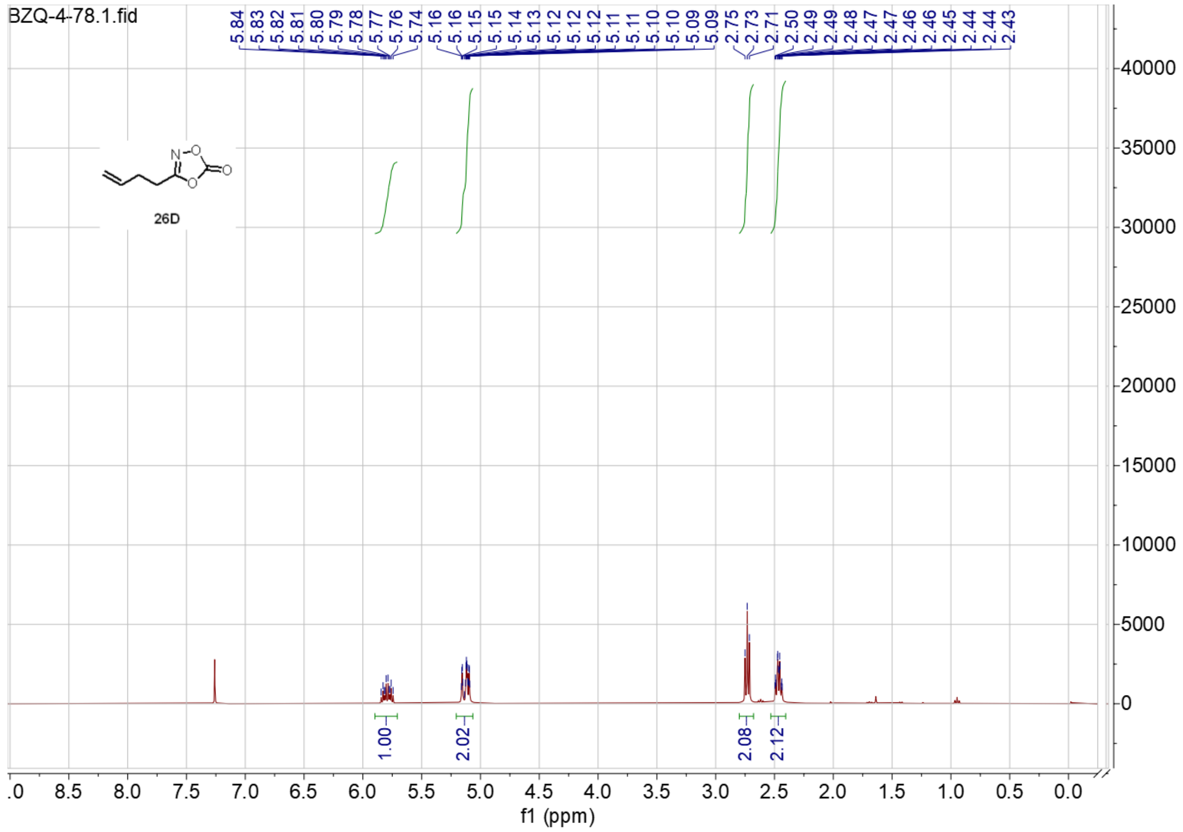

Supplementary Figure 58. ^{1}H NMR (400 MHz, $CDCl_3$, 293 K) spectrum of **26D**.

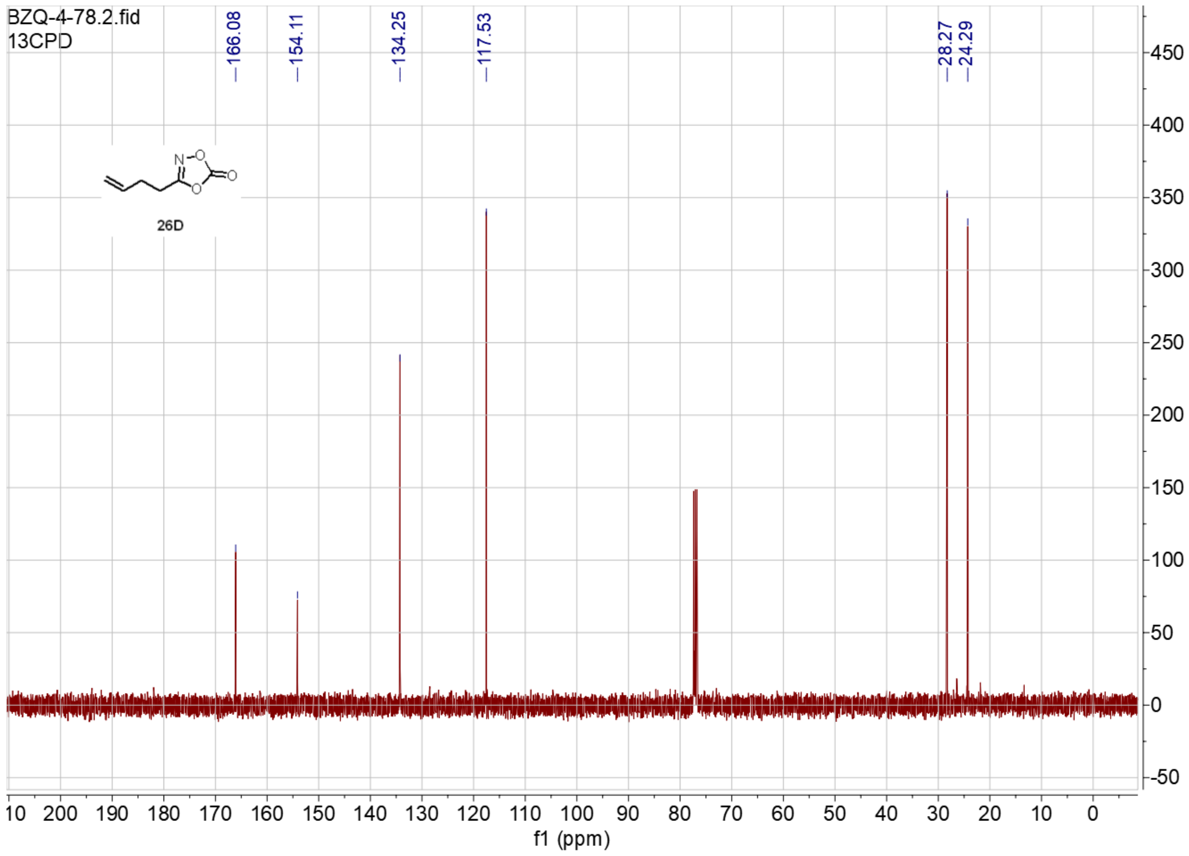

Supplementary Figure 59. ^{13}C NMR (101 MHz, $CDCl_3$, 293 K) spectrum of **26D**.

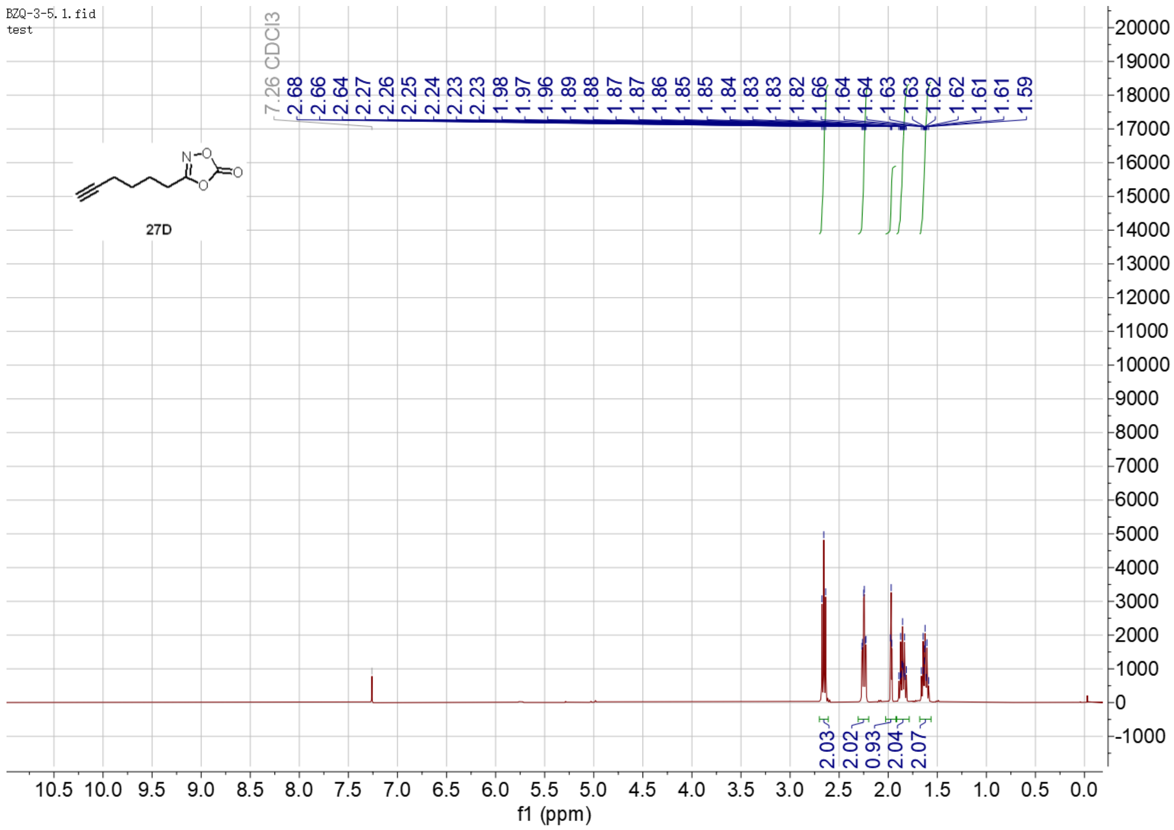

Supplementary Figure 60. ^{1}H NMR (400 MHz, $CDCl_3$, 293 K) spectrum of **27D**.

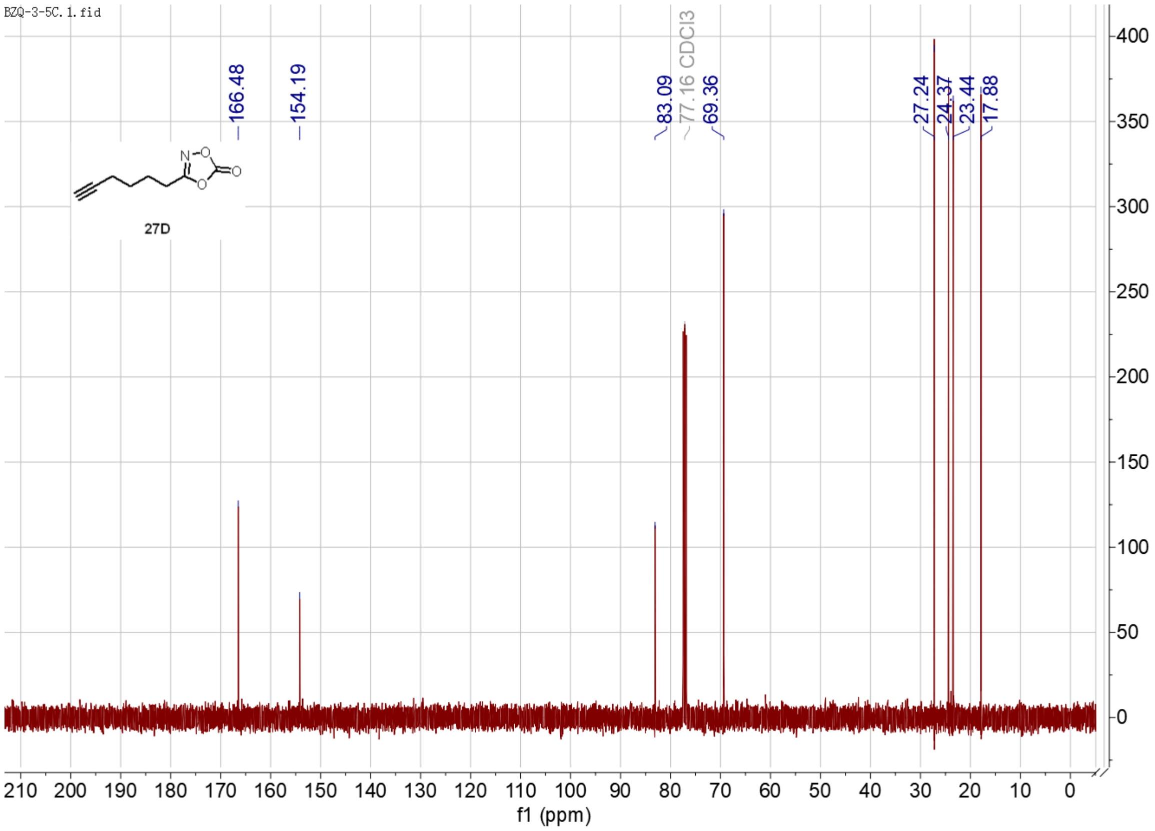

Supplementary Figure 61. ^{13}C NMR (101 MHz, $CDCl_3$, 293 K) spectrum of **27D**.

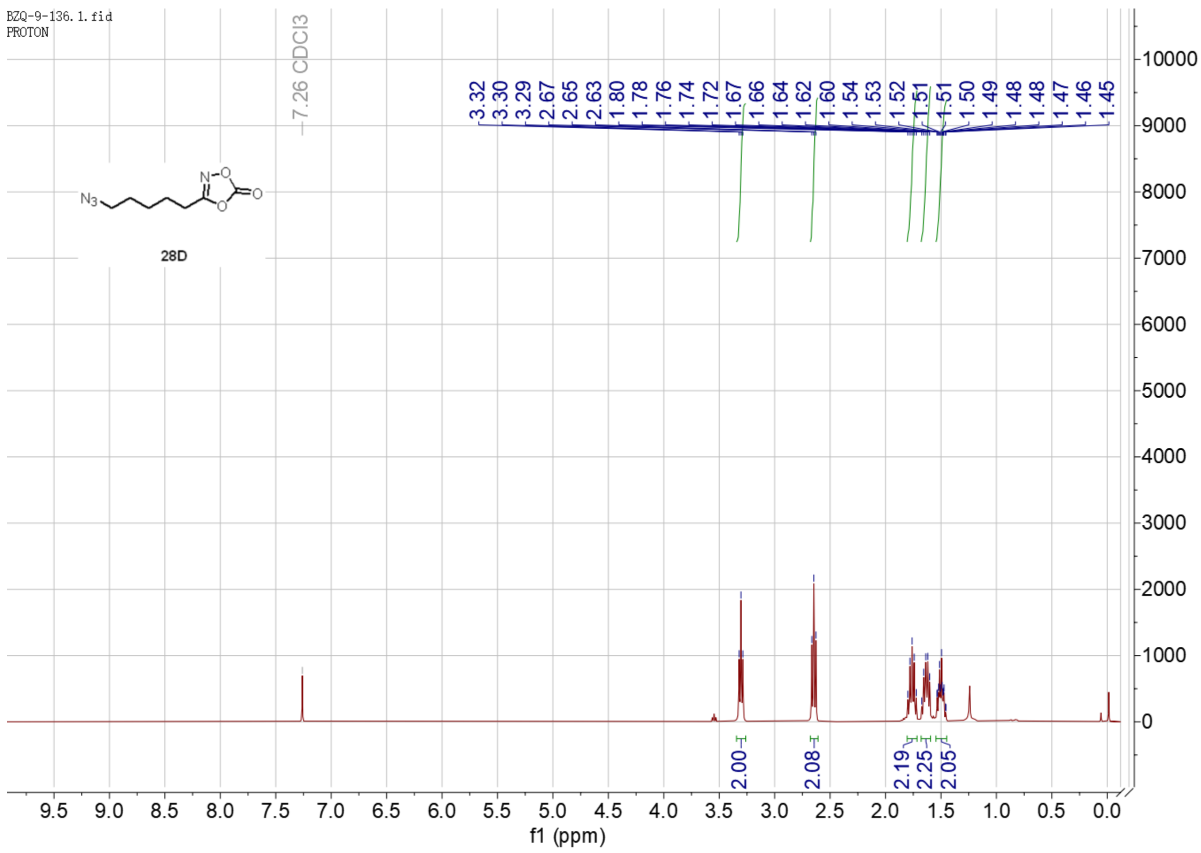

Supplementary Figure 62. ^{1}H NMR (400 MHz, $CDCl_3$, 293 K) spectrum of **28D**.

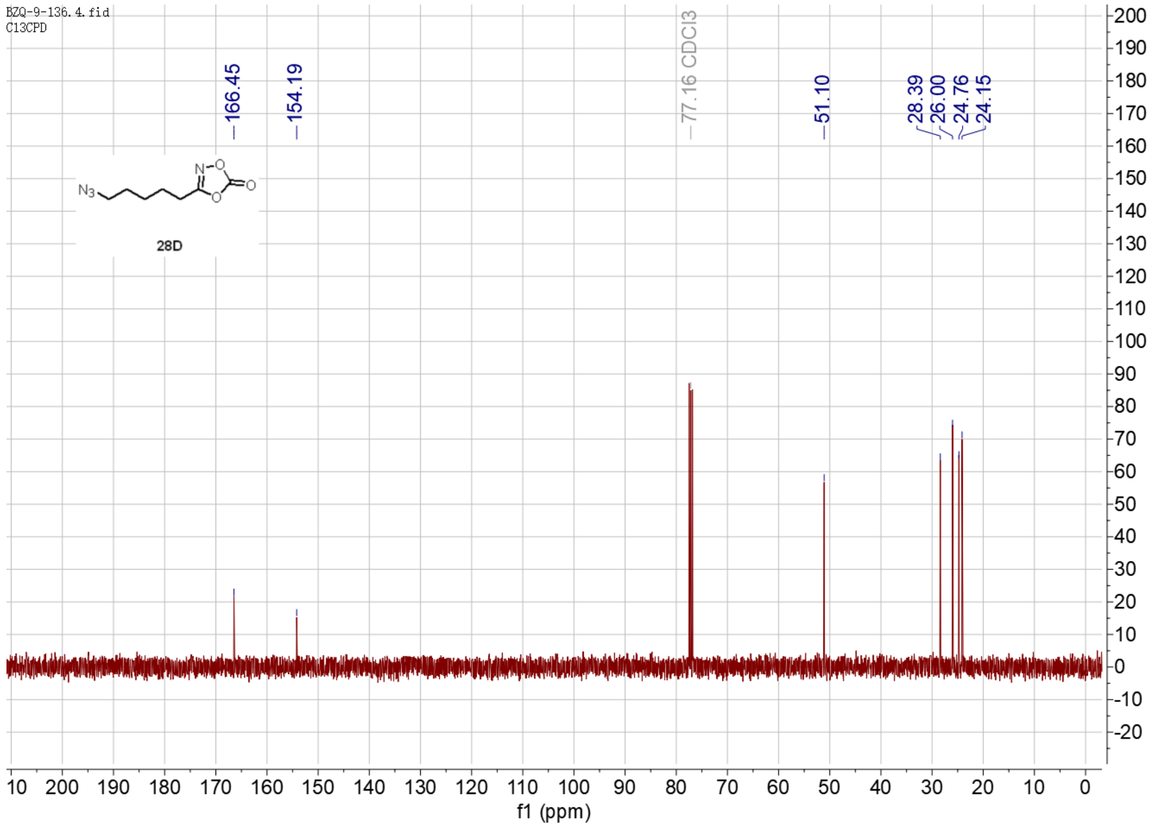

Supplementary Figure 63. ^{13}C NMR (101 MHz, $CDCl_3$, 293 K) spectrum of **28D**.

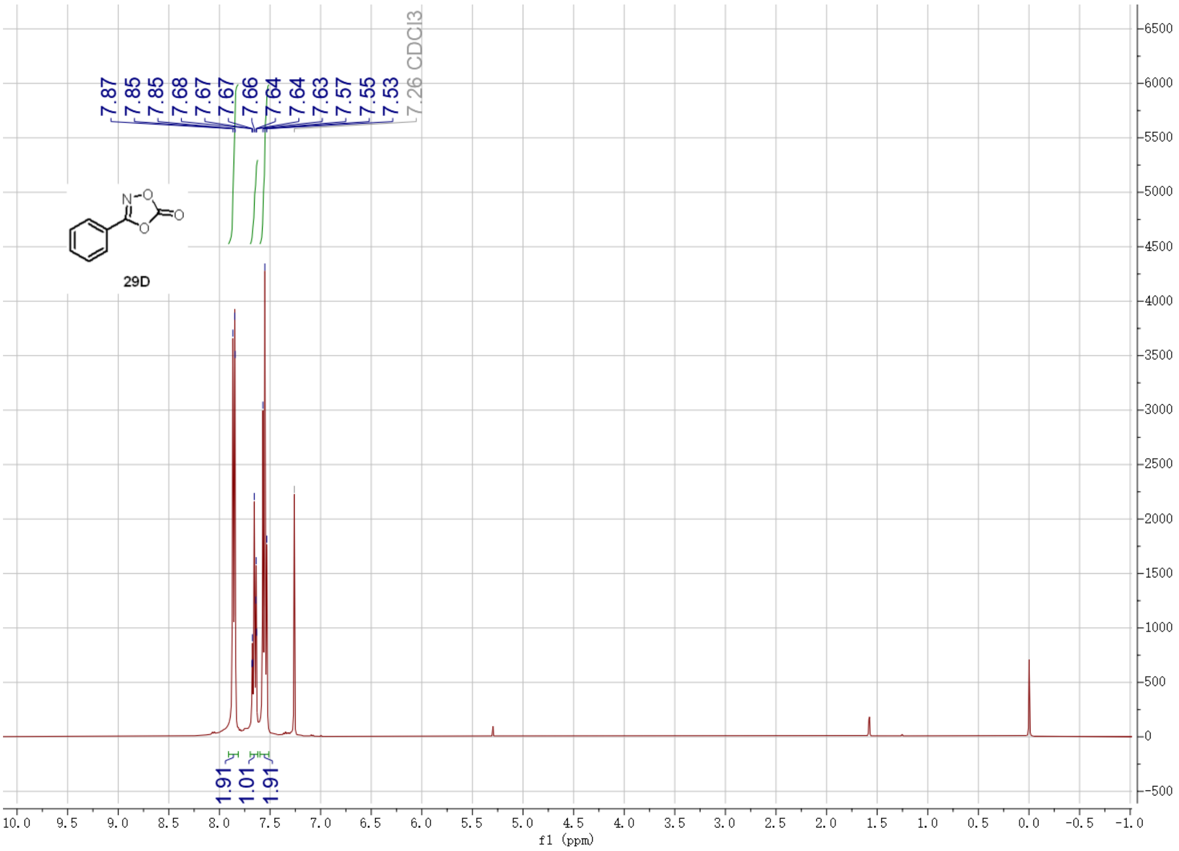

Supplementary Figure 64. ^{1}H NMR (400 MHz, $CDCl_3$, 293 K) spectrum of **29D**.

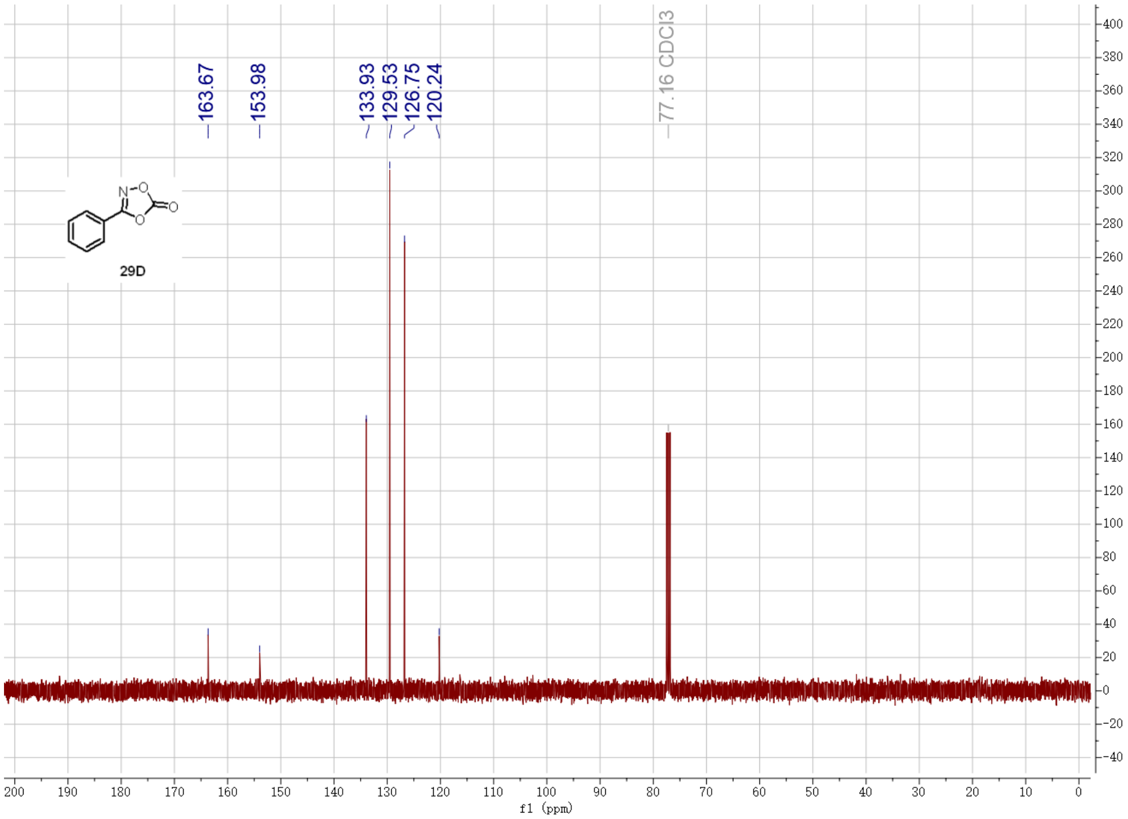

Supplementary Figure 65. ^{13}C NMR (101 MHz, $CDCl_3$, 293 K) spectrum of **29D**.

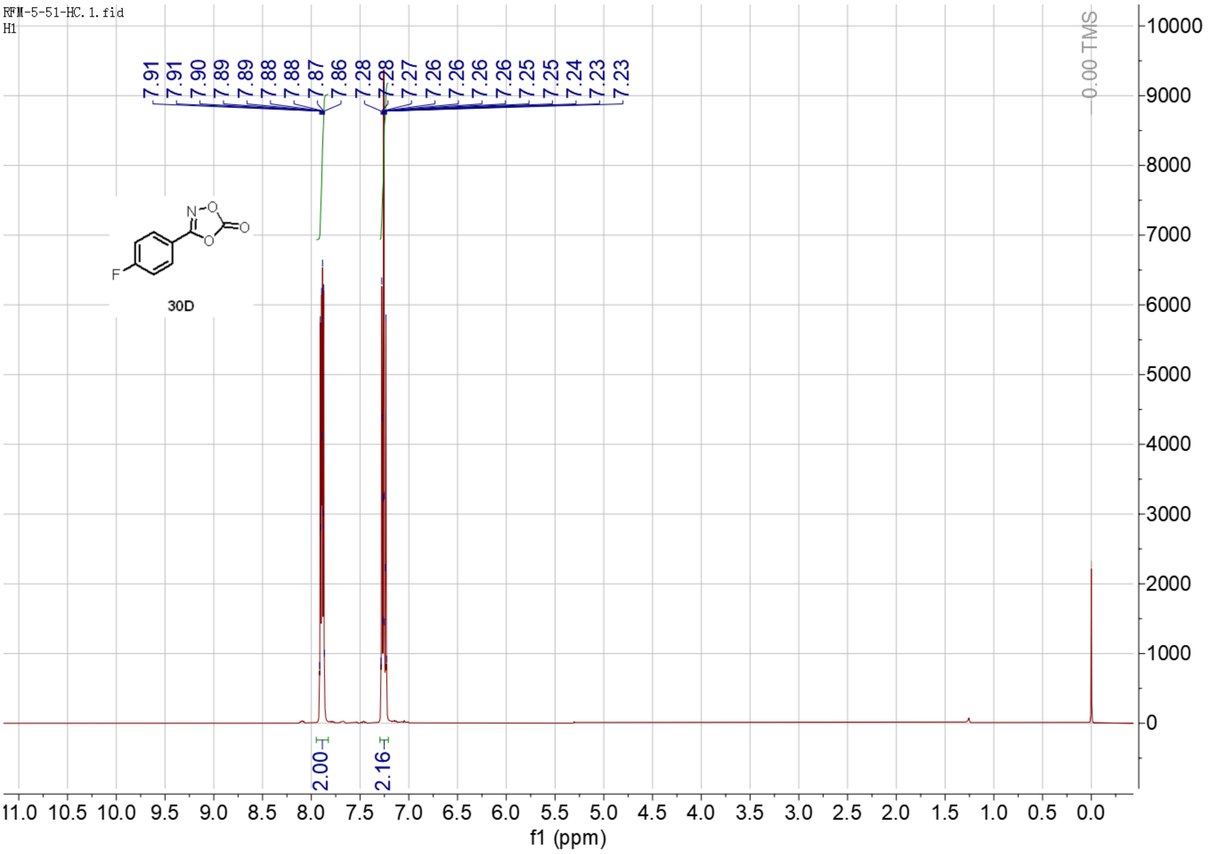

Supplementary Figure 66. ^{1}H NMR (400 MHz, $CDCl_3$, 293 K) spectrum of **30D**.

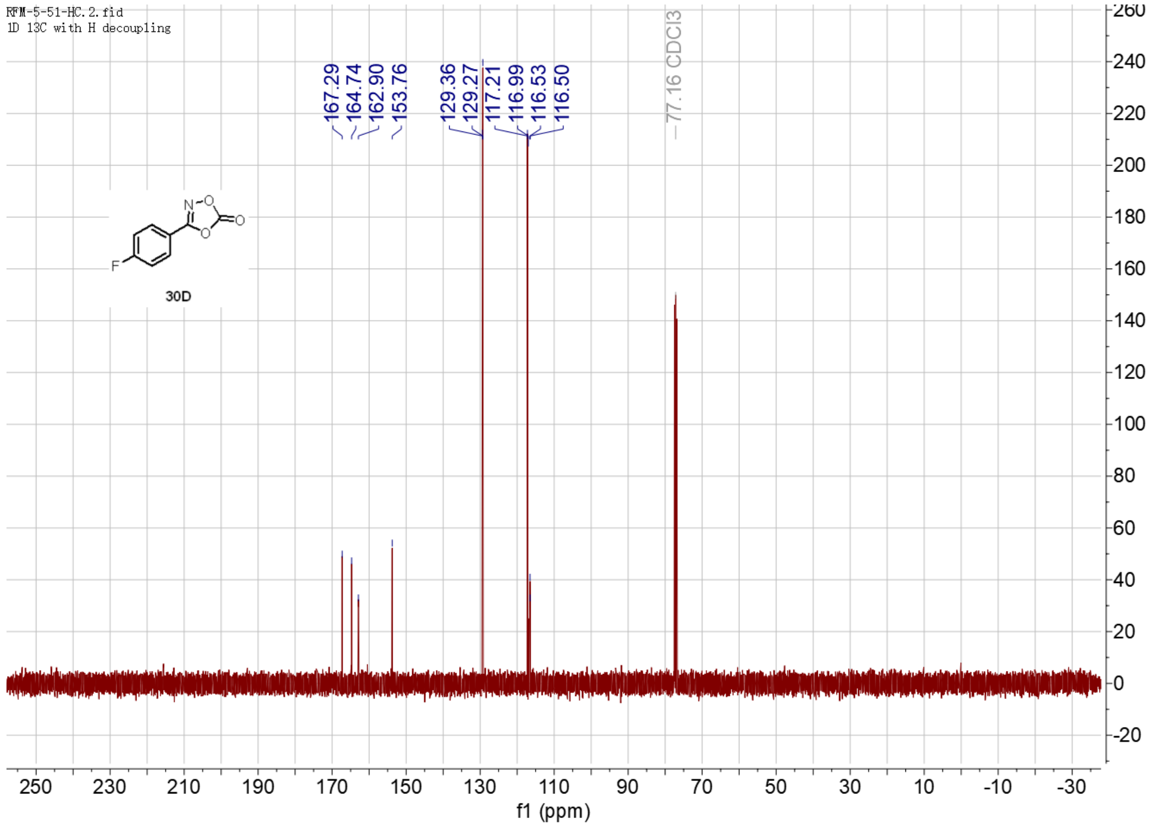

Supplementary Figure 67. ^{13}C NMR (101 MHz, $CDCl_3$, 293 K) spectrum of **30D**.

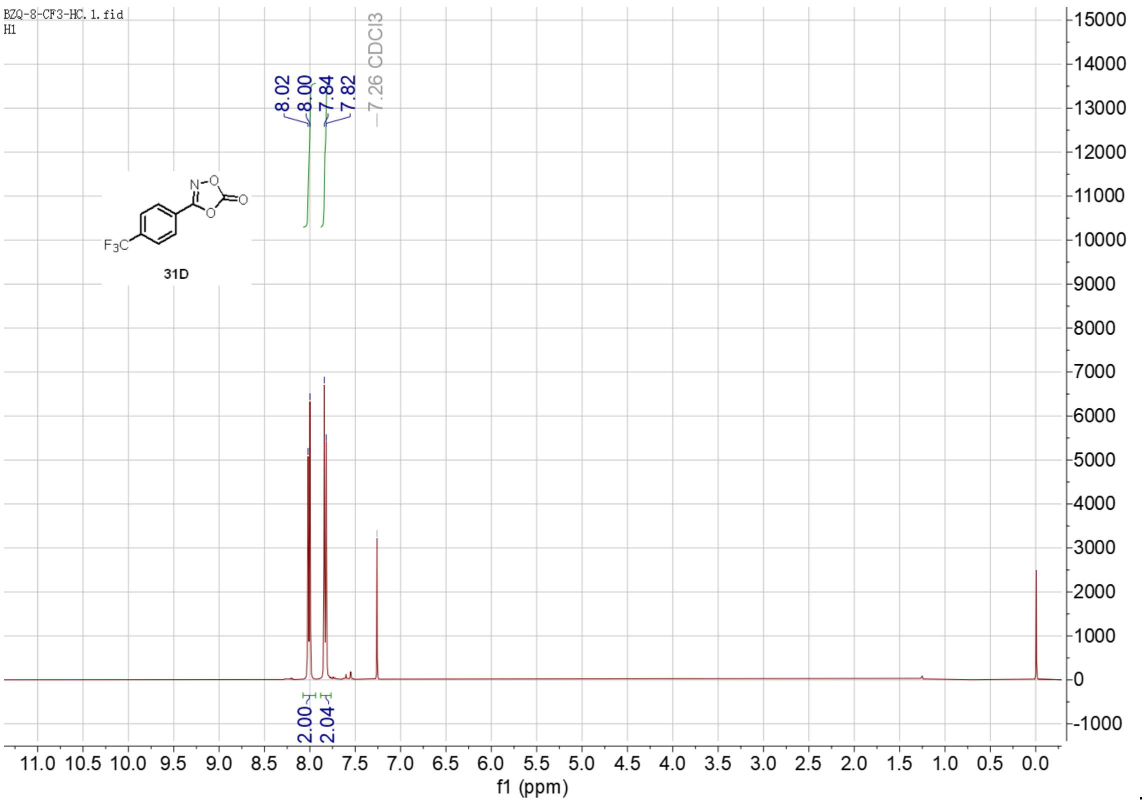

Supplementary Figure 68. ^{1}H NMR (400 MHz, $CDCl_3$, 293 K) spectrum of **31D**.

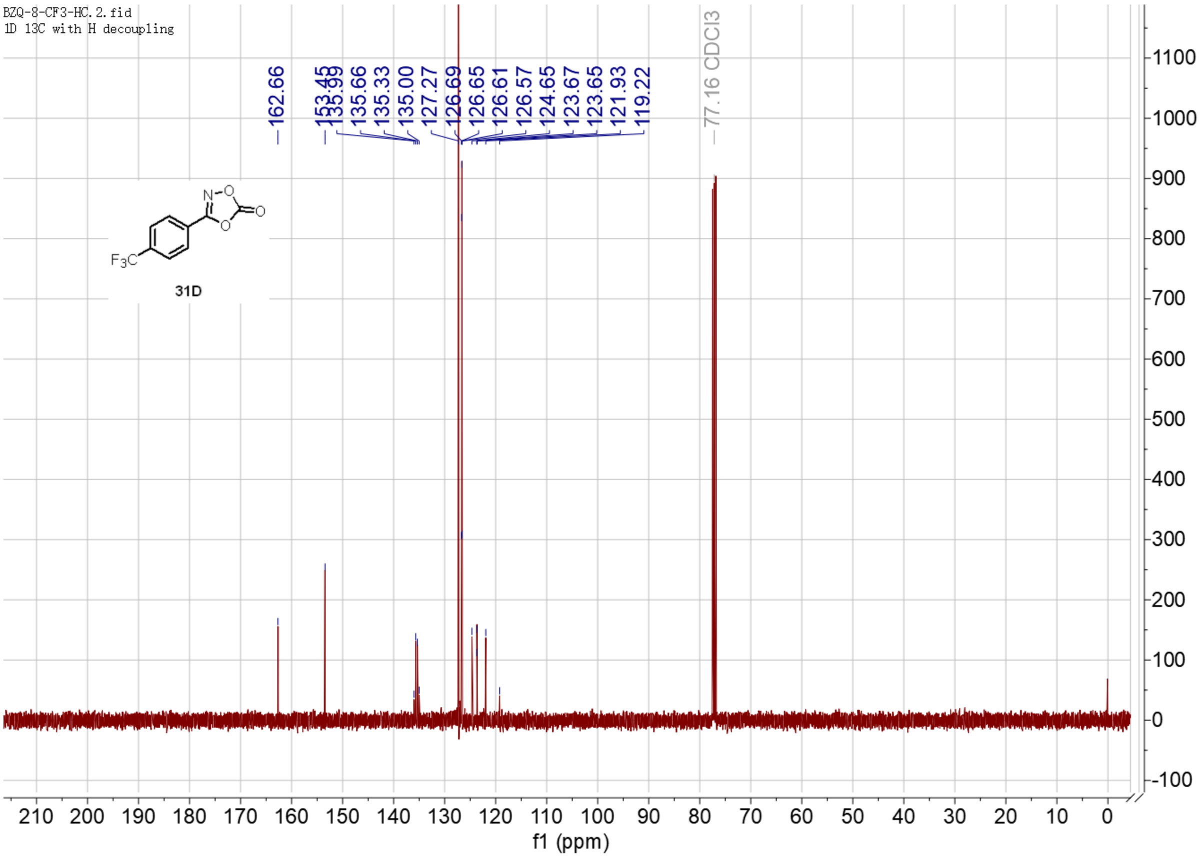

Supplementary Figure 69. ^{13}C NMR (101 MHz, $CDCl_3$, 293 K) spectrum of **31D**.

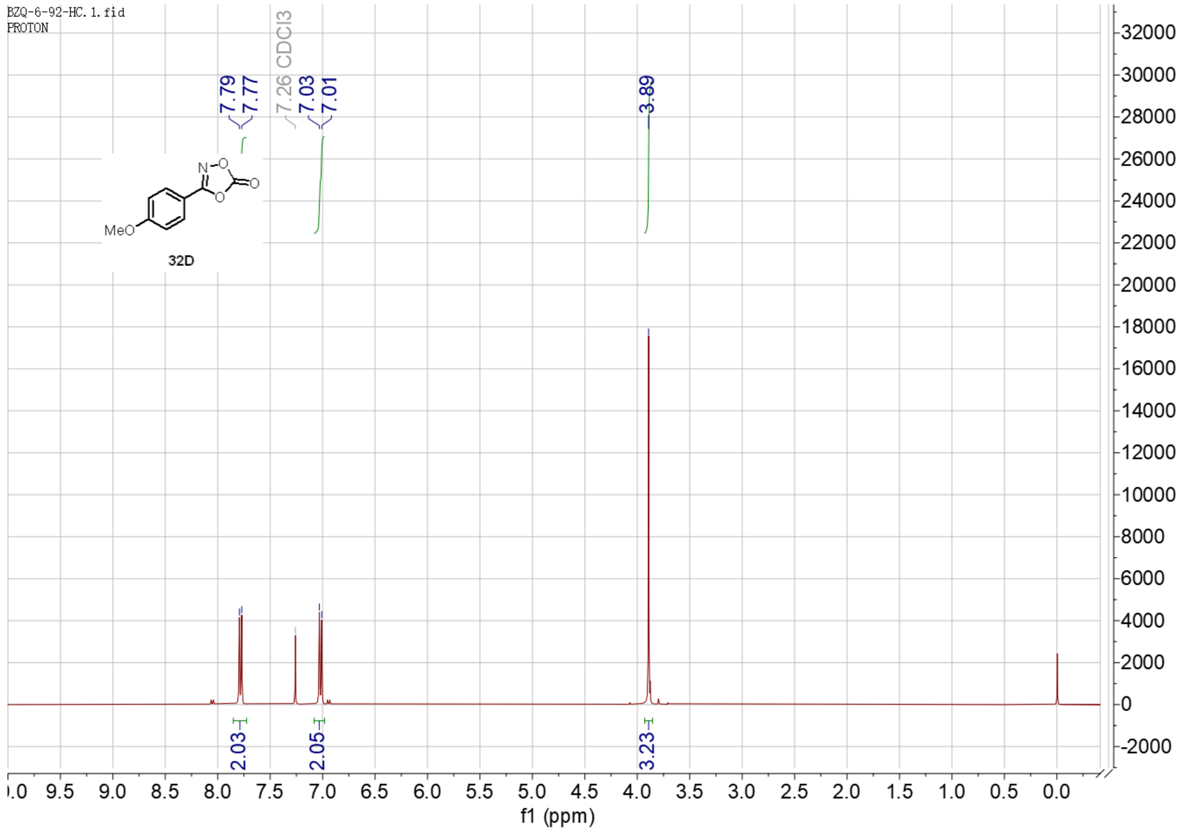

Supplementary Figure 70. ^{1}H NMR (400 MHz, $CDCl_3$, 293 K) spectrum of **32D**.

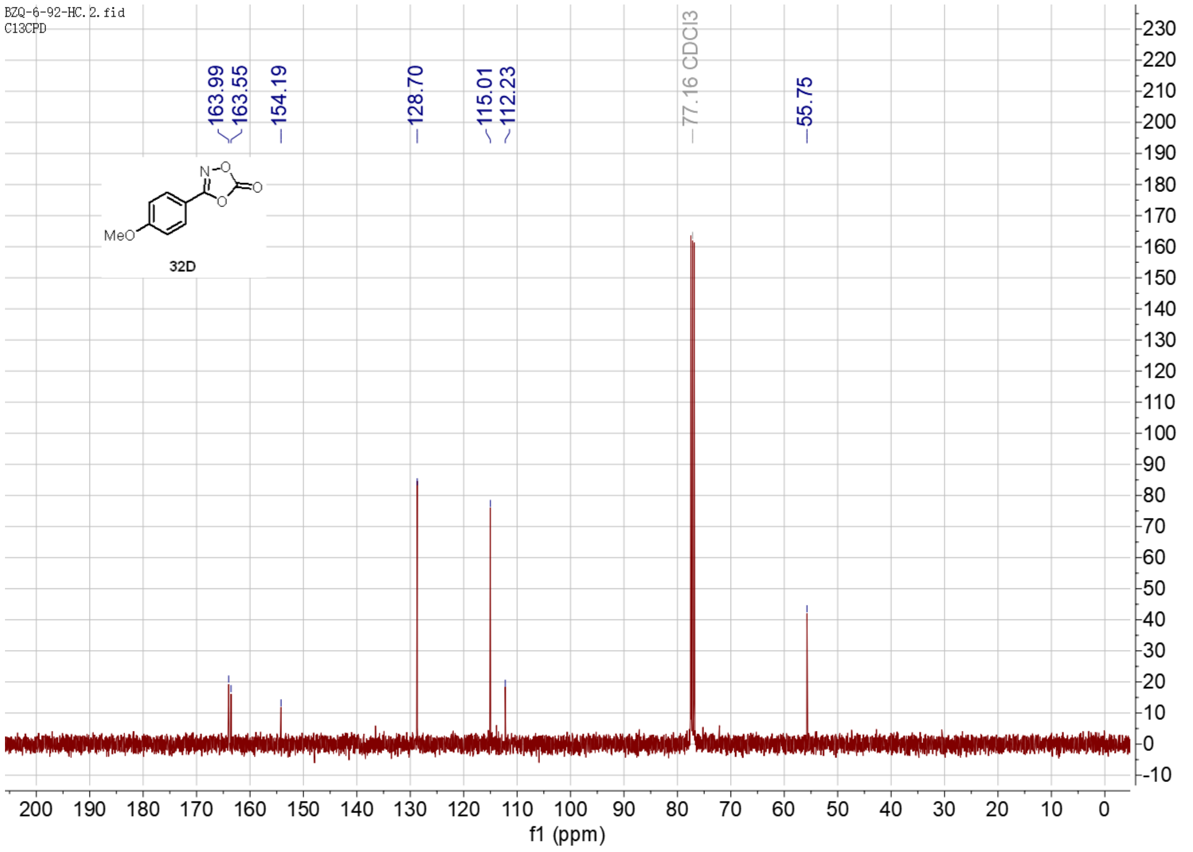

Supplementary Figure 71. ^{13}C NMR (101 MHz, $CDCl_3$, 293 K) spectrum of **32D**.

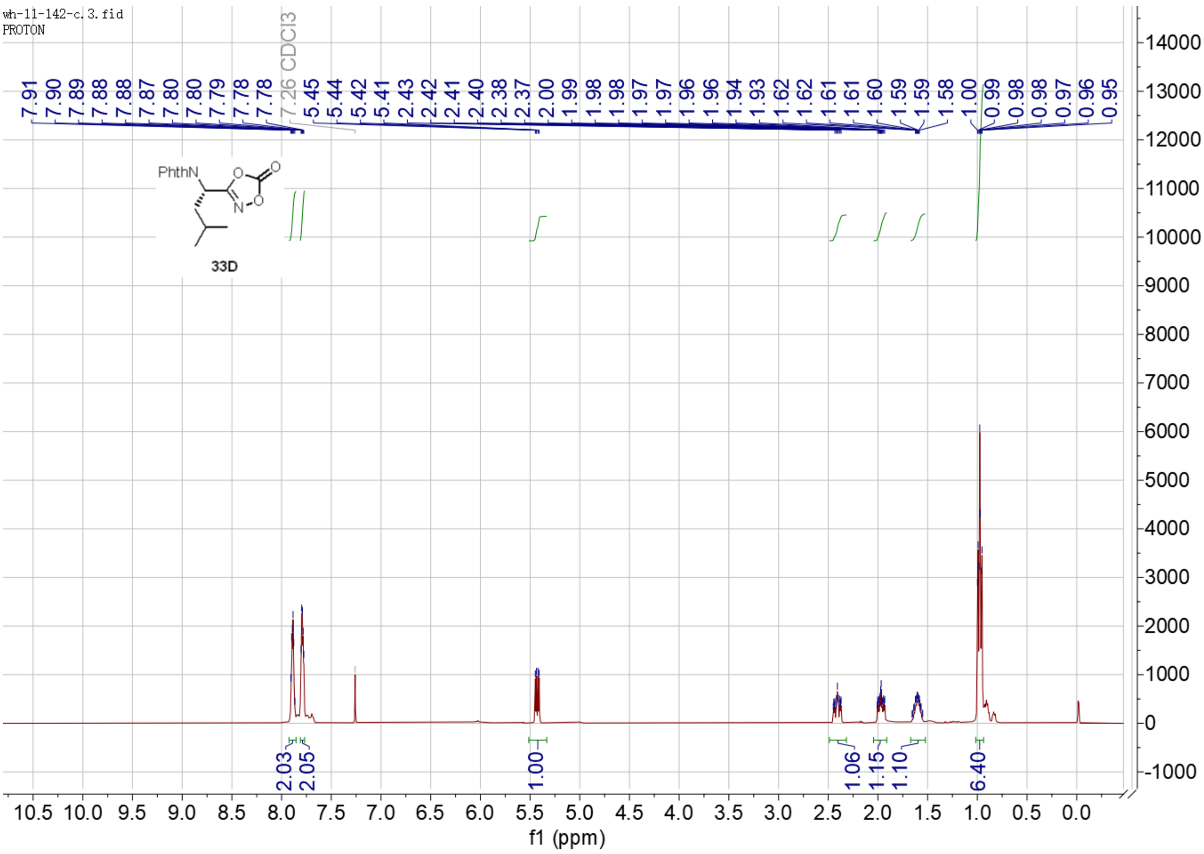

Supplementary Figure 72. ^{1}H NMR (400 MHz, $CDCl_3$, 293 K) spectrum of **33D**.

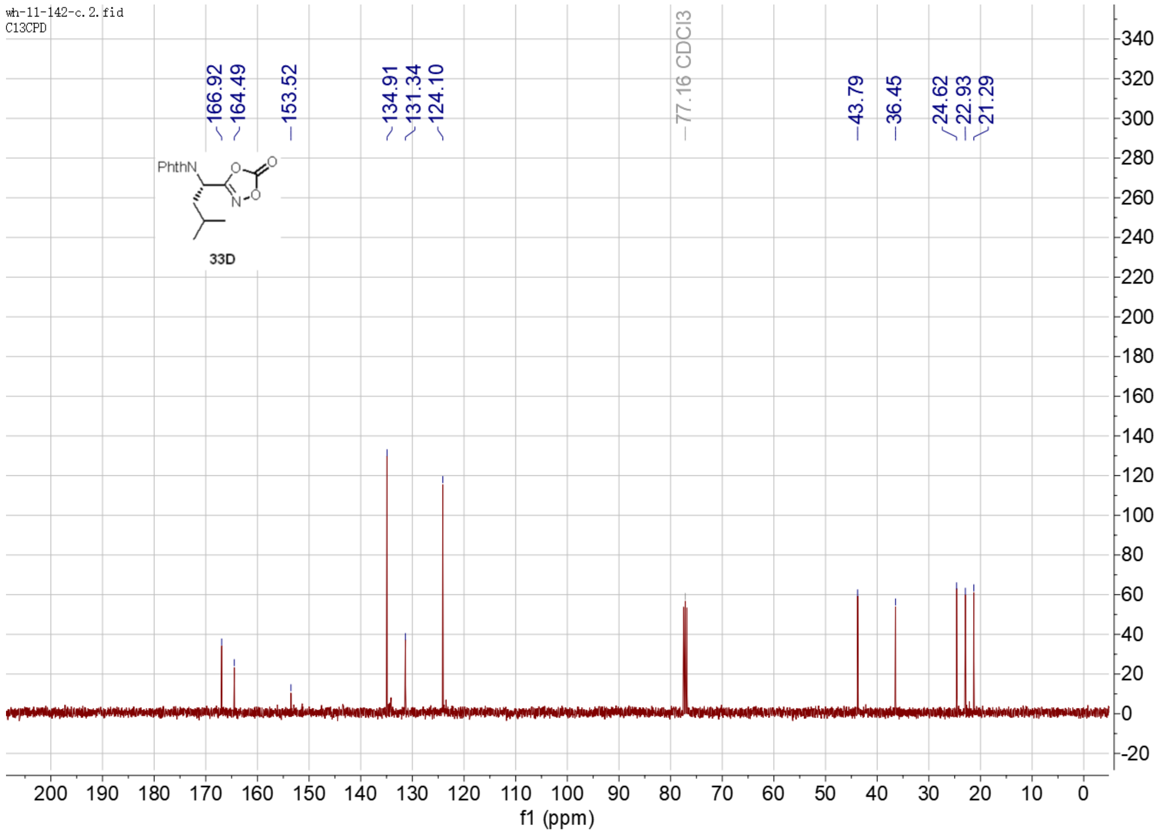

Supplementary Figure 73. ^{13}C NMR (101 MHz, $CDCl_3$, 293 K) spectrum of **33D**.

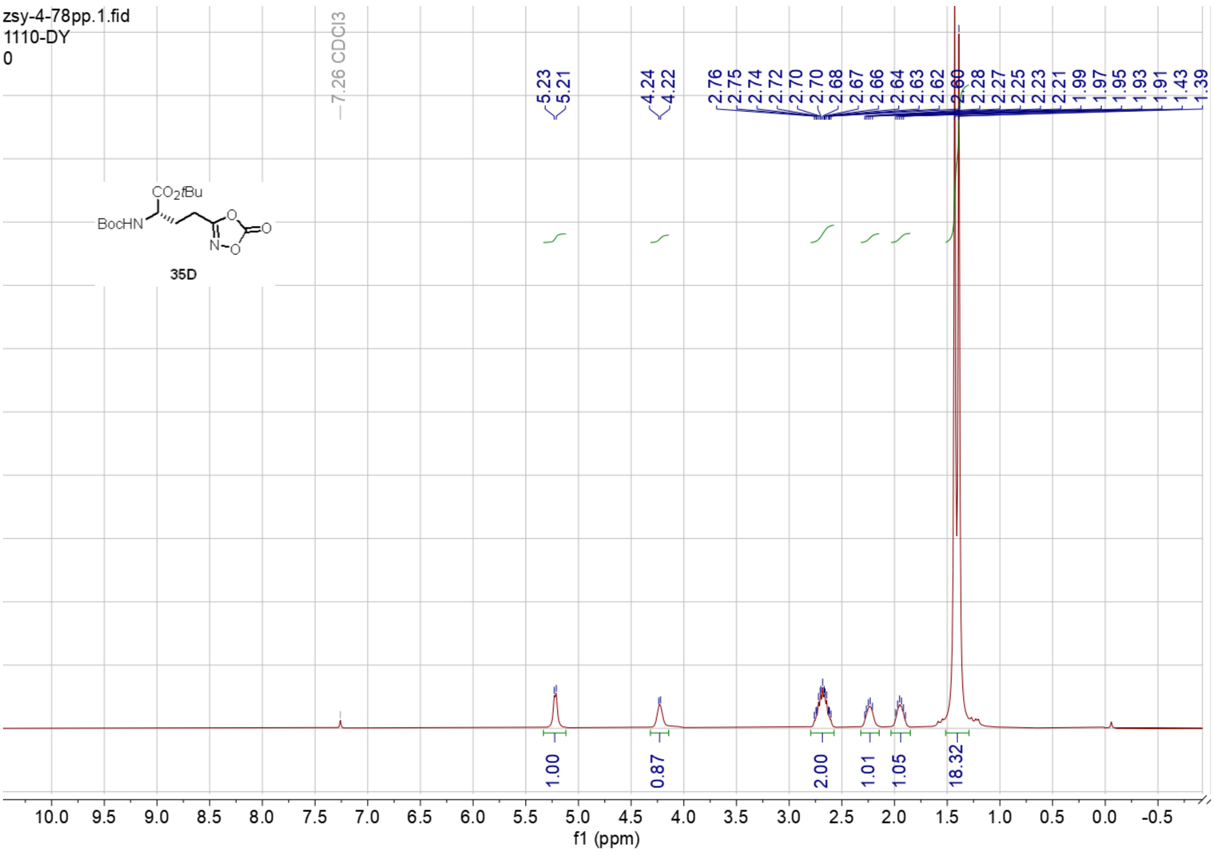

Supplementary Figure 74. ^{1}H NMR (400 MHz, $CDCl_3$, 293 K) spectrum of **35D**.

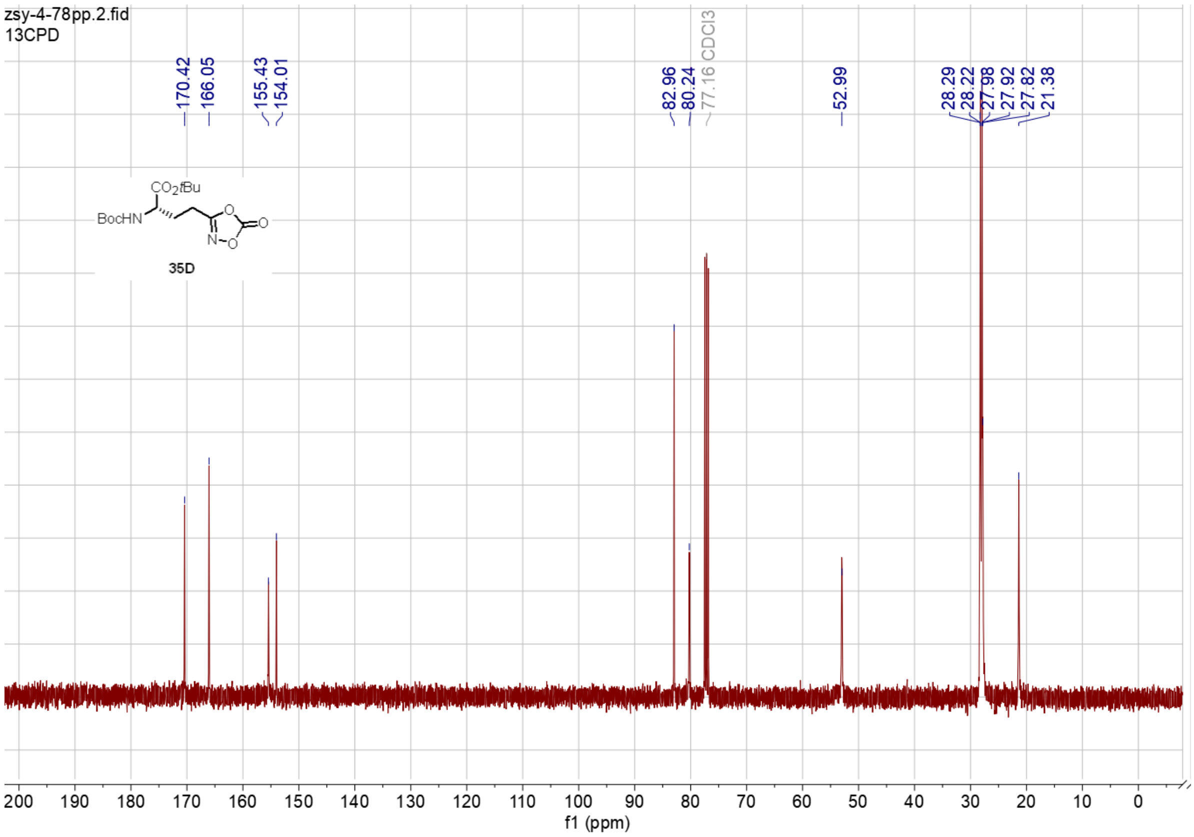

Supplementary Figure 75. ^{13}C NMR (101 MHz, $CDCl_3$, 293 K) spectrum of **35D**.

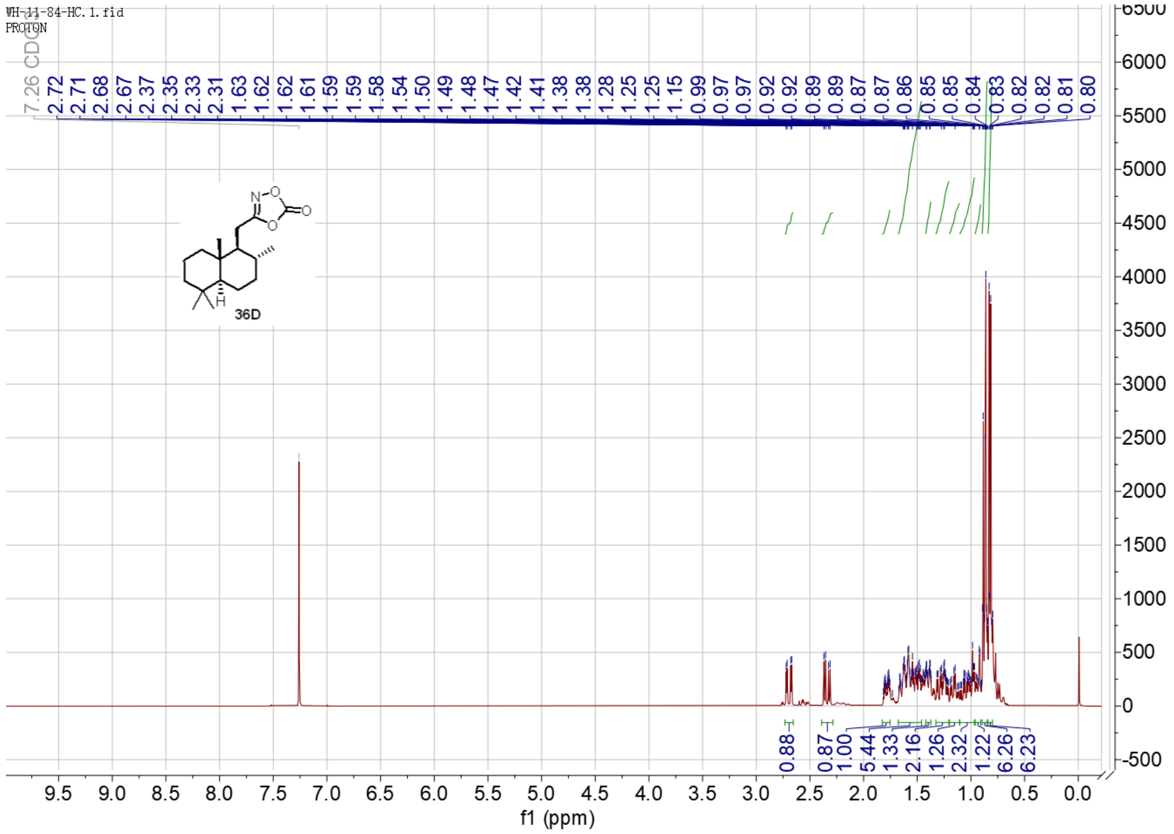

Supplementary Figure 76. ^{1}H NMR (400 MHz, $CDCl_3$, 293 K) spectrum of **36D**.

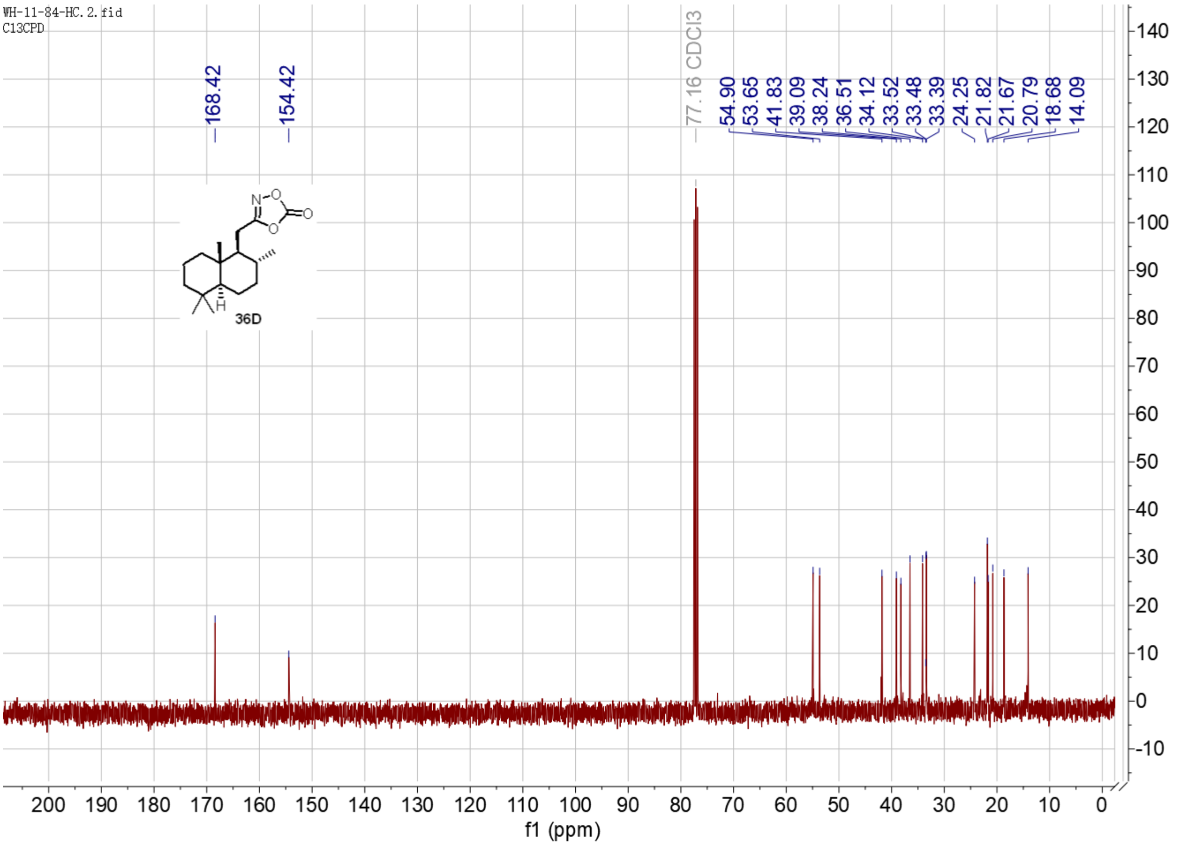

Supplementary Figure 77. ^{13}C NMR (101 MHz, $CDCl_3$, 293 K) spectrum of **36D**.

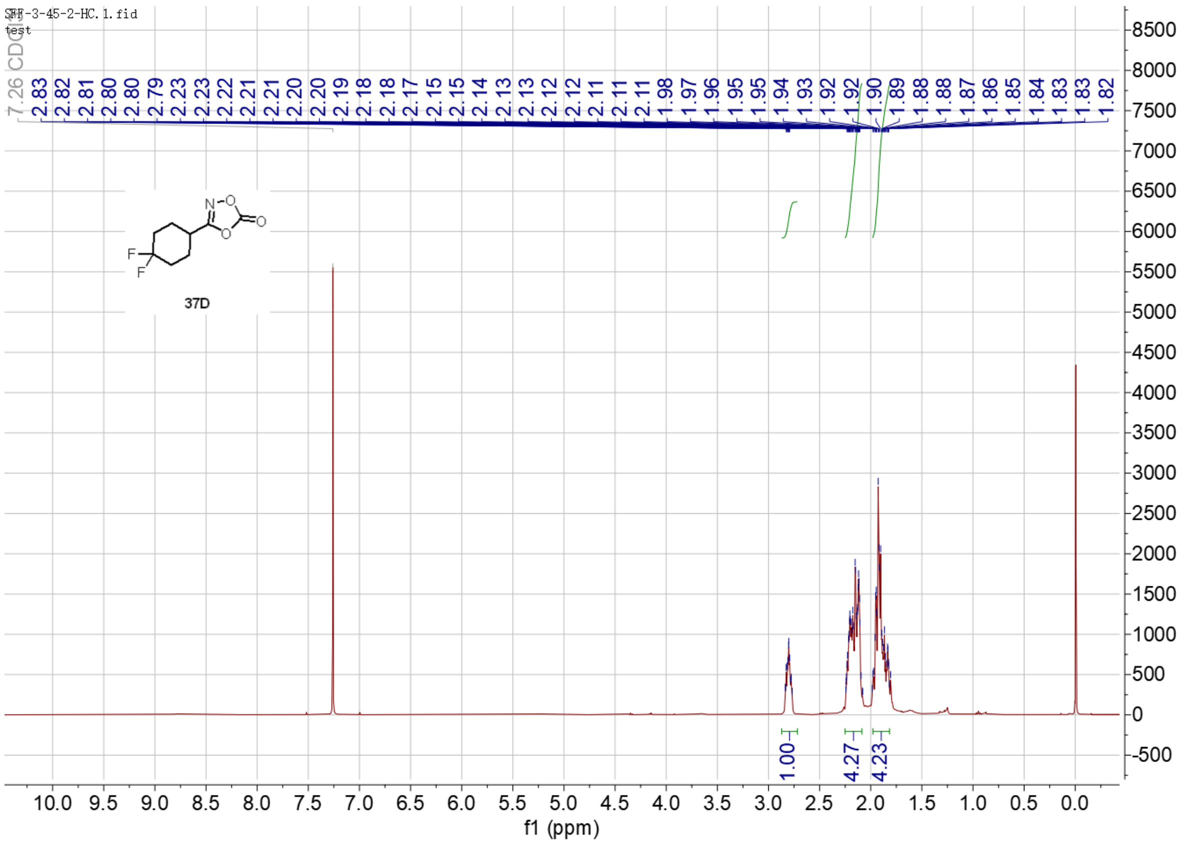

Supplementary Figure 78. ^{1}H NMR (400 MHz, $CDCl_3$, 293 K) spectrum of **37D**.

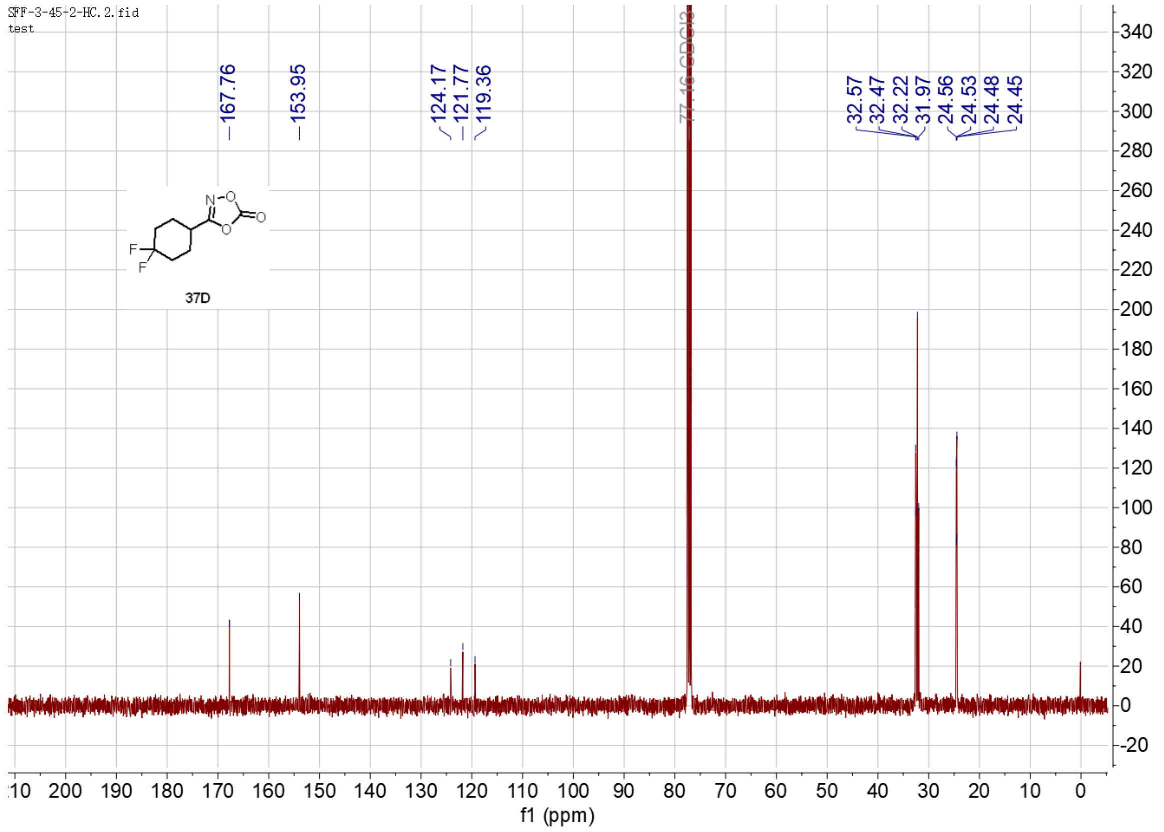

Supplementary Figure 79. ^{13}C NMR (101 MHz, $CDCl_3$, 293 K) spectrum of **37D**.

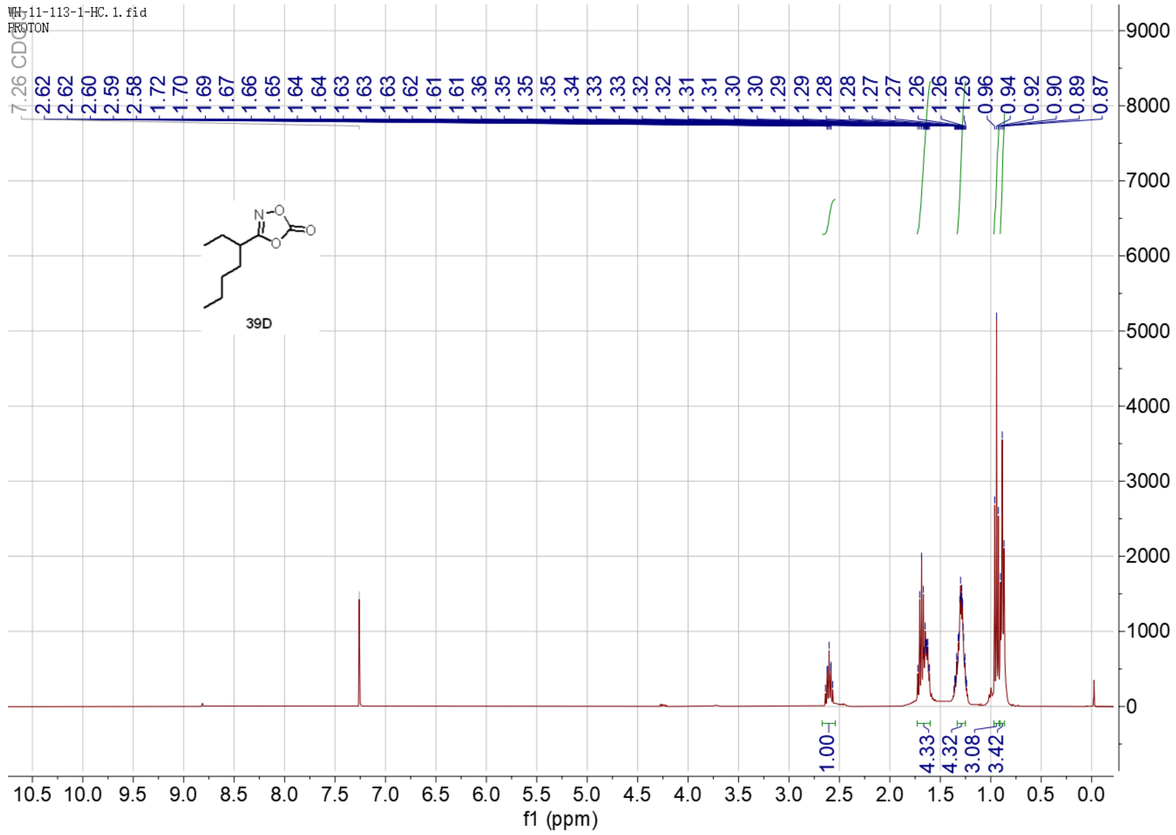

Supplementary Figure 80. ^{1}H NMR (400 MHz, $CDCl_3$, 293 K) spectrum of **39D**.

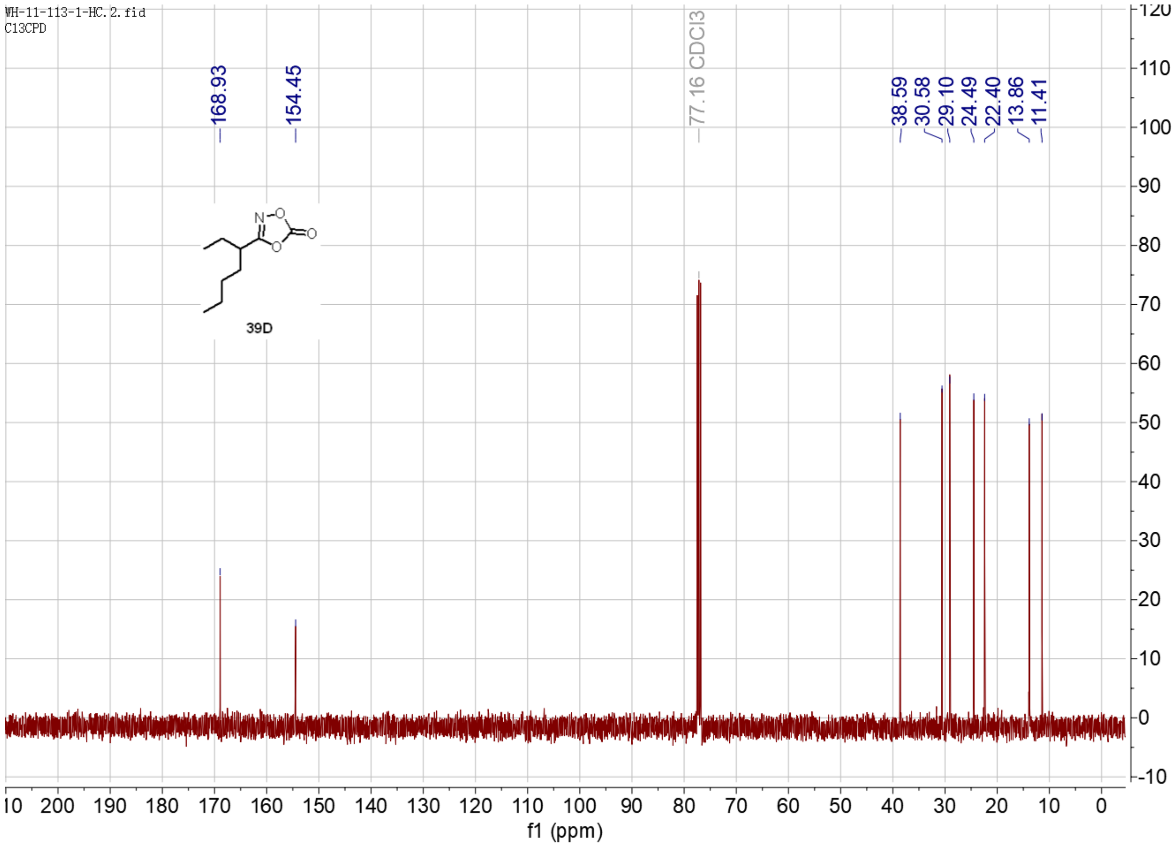

Supplementary Figure 81. ^{13}C NMR (101 MHz, $CDCl_3$, 293 K) spectrum of **39D**.

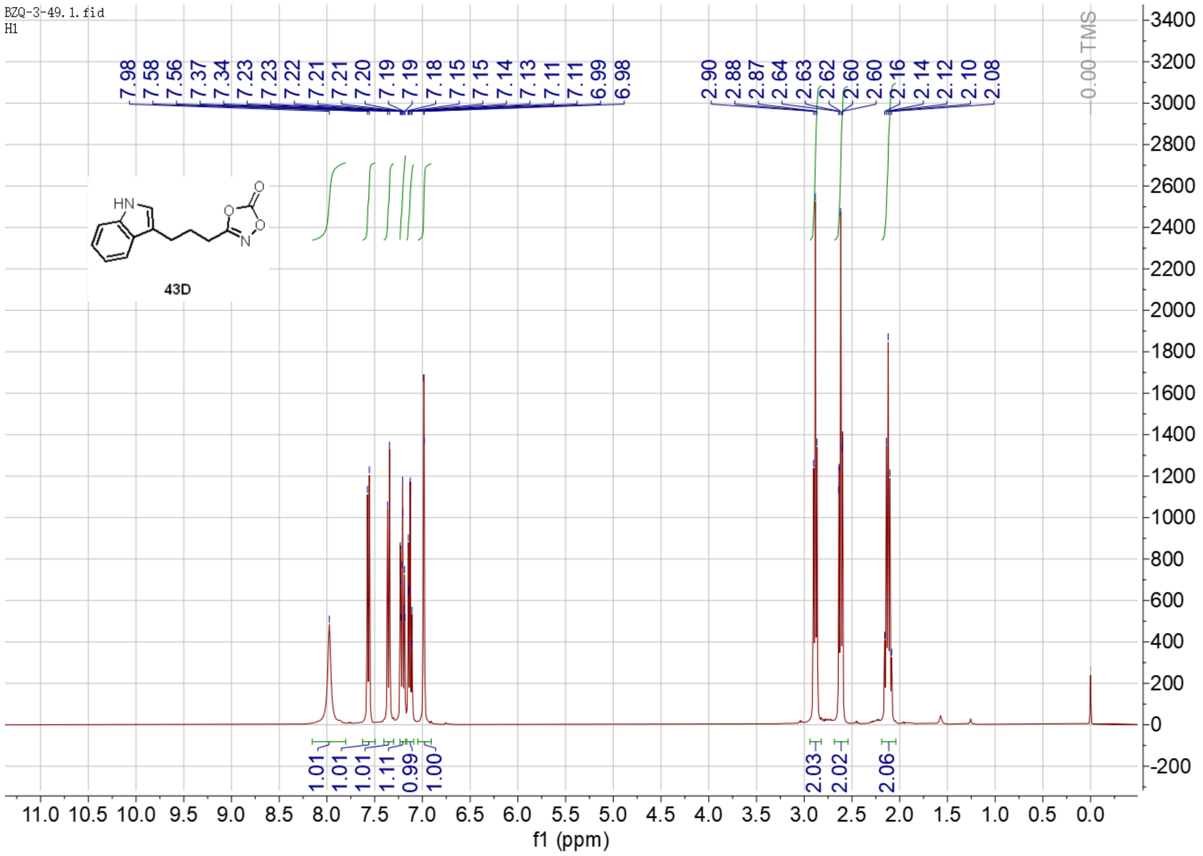

Supplementary Figure 82. ^{1}H NMR (400 MHz, $CDCl_3$, 293 K) spectrum of **43D**.

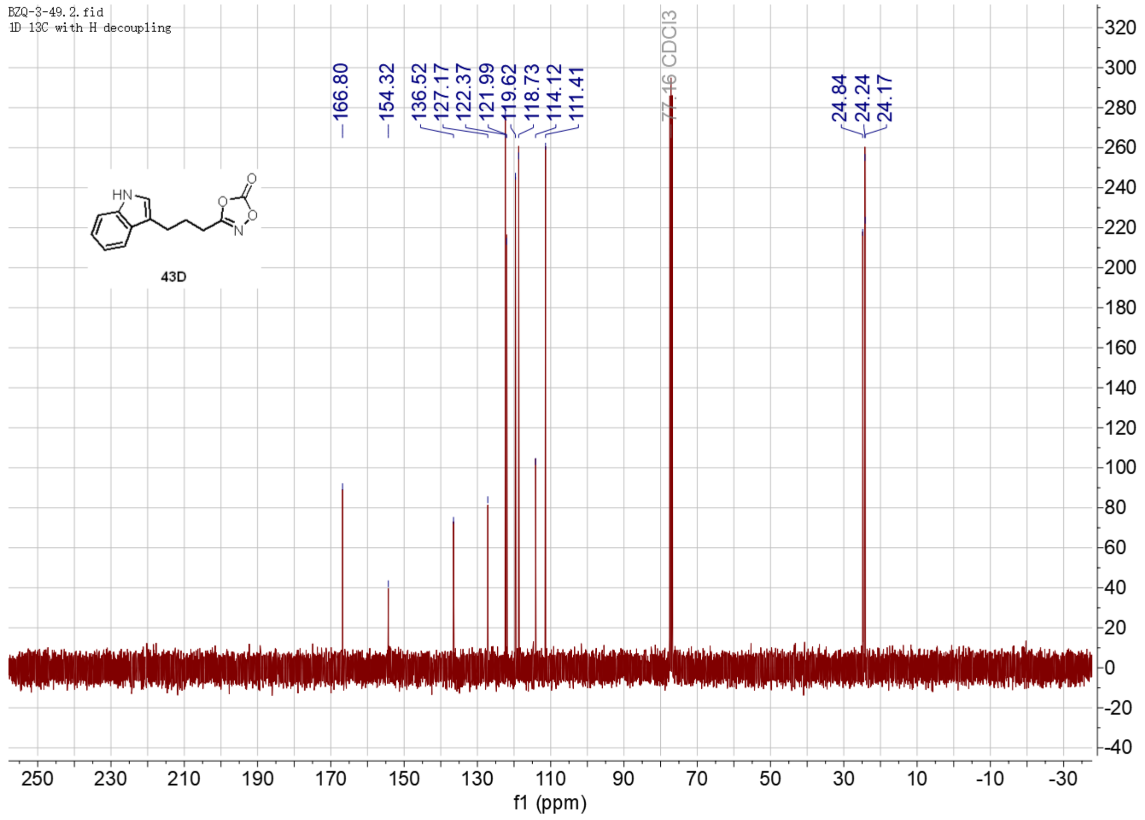

Supplementary Figure 83. ^{13}C NMR (101 MHz, $CDCl_3$, 293 K) spectrum of **43D**.

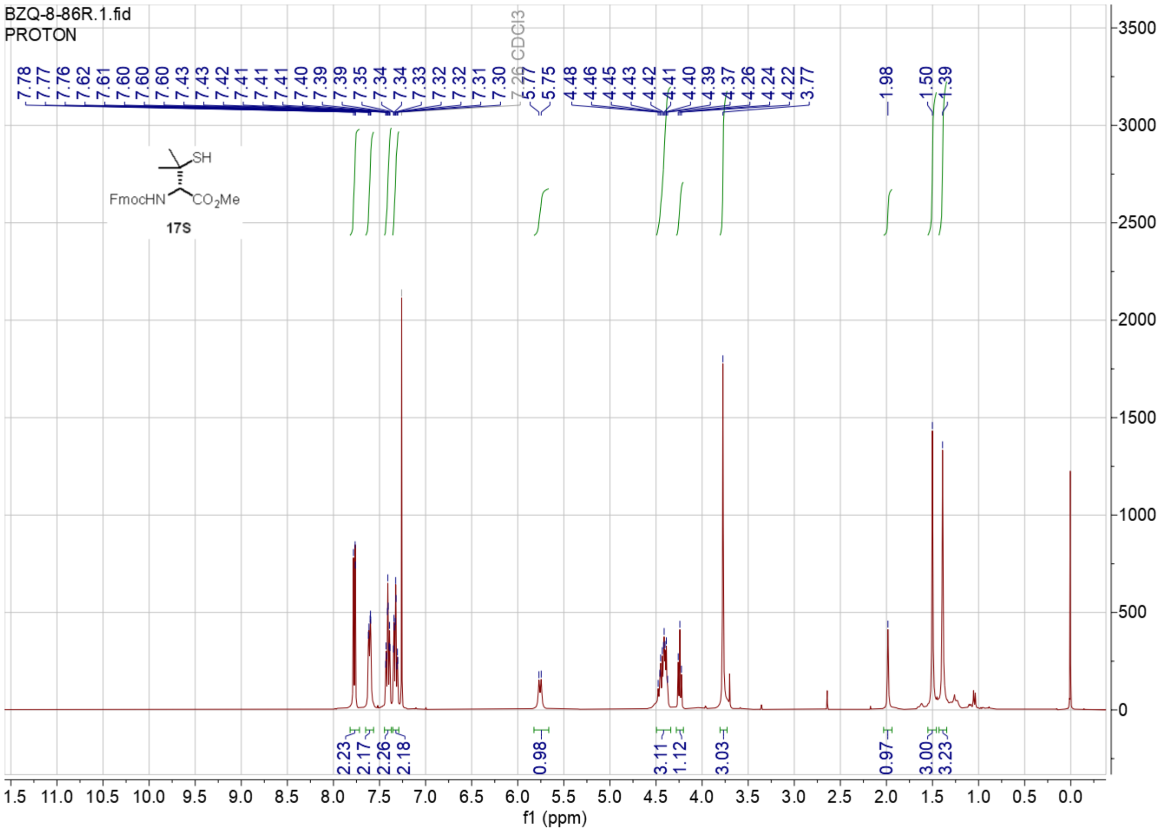

Supplementary Figure 84. ^{1}H NMR (400 MHz, $CDCl_3$, 293 K) spectrum of **17S**.

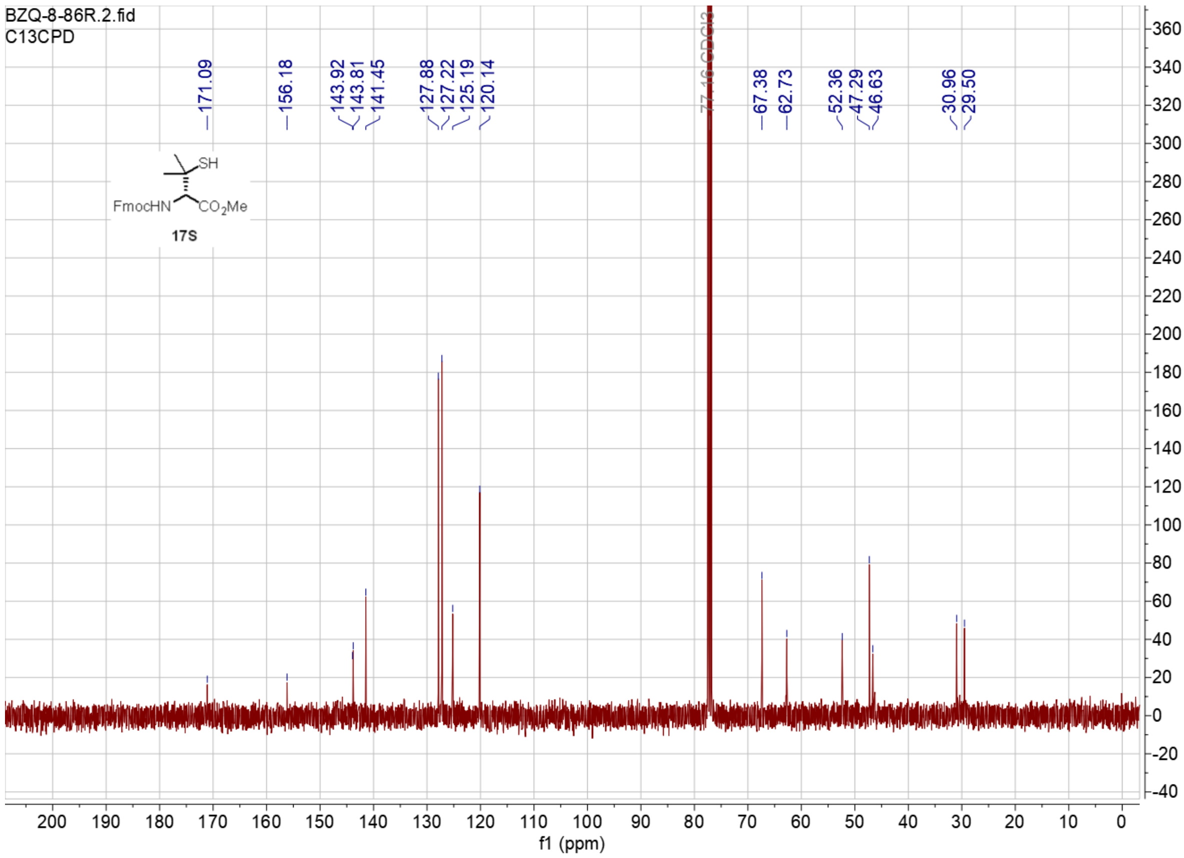

Supplementary Figure 85. ^{13}C NMR (101 MHz, $CDCl_3$, 293 K) spectrum of **17S**.

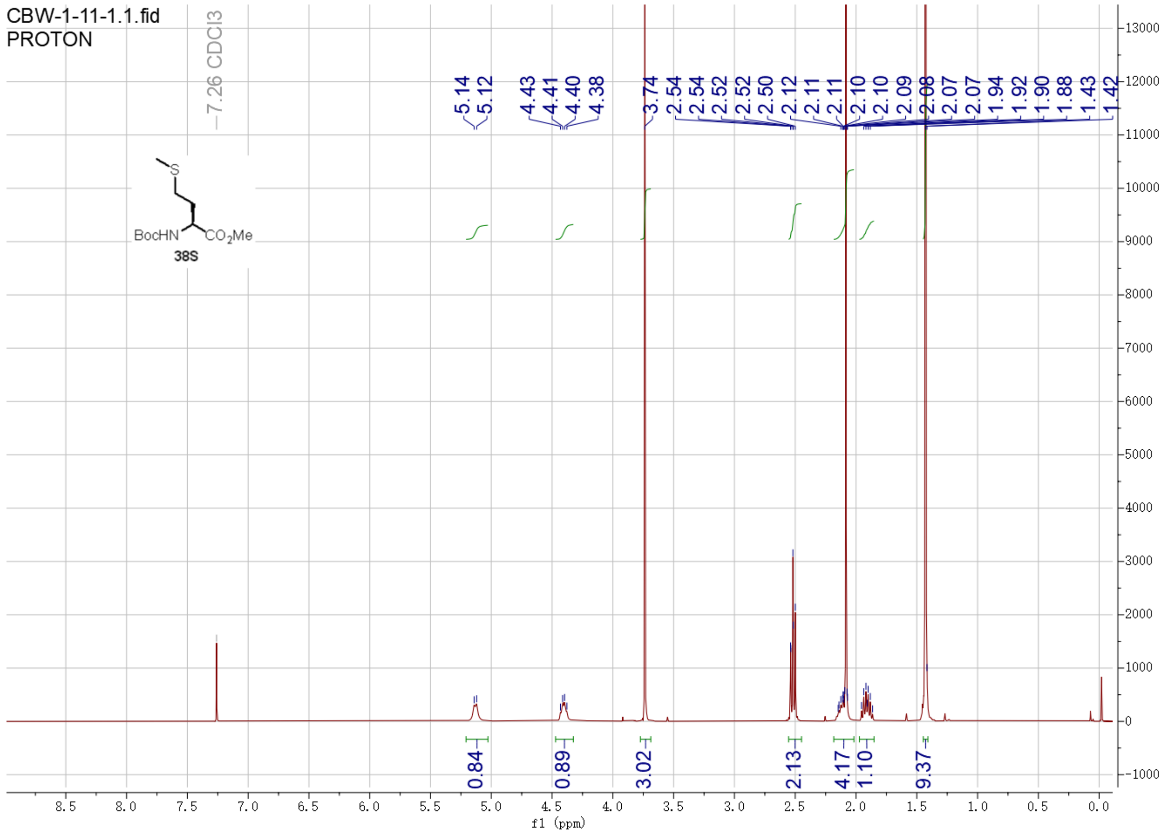

Supplementary Figure 86. ^{1}H NMR (400 MHz, $CDCl_3$, 293 K) spectrum of **38S**.

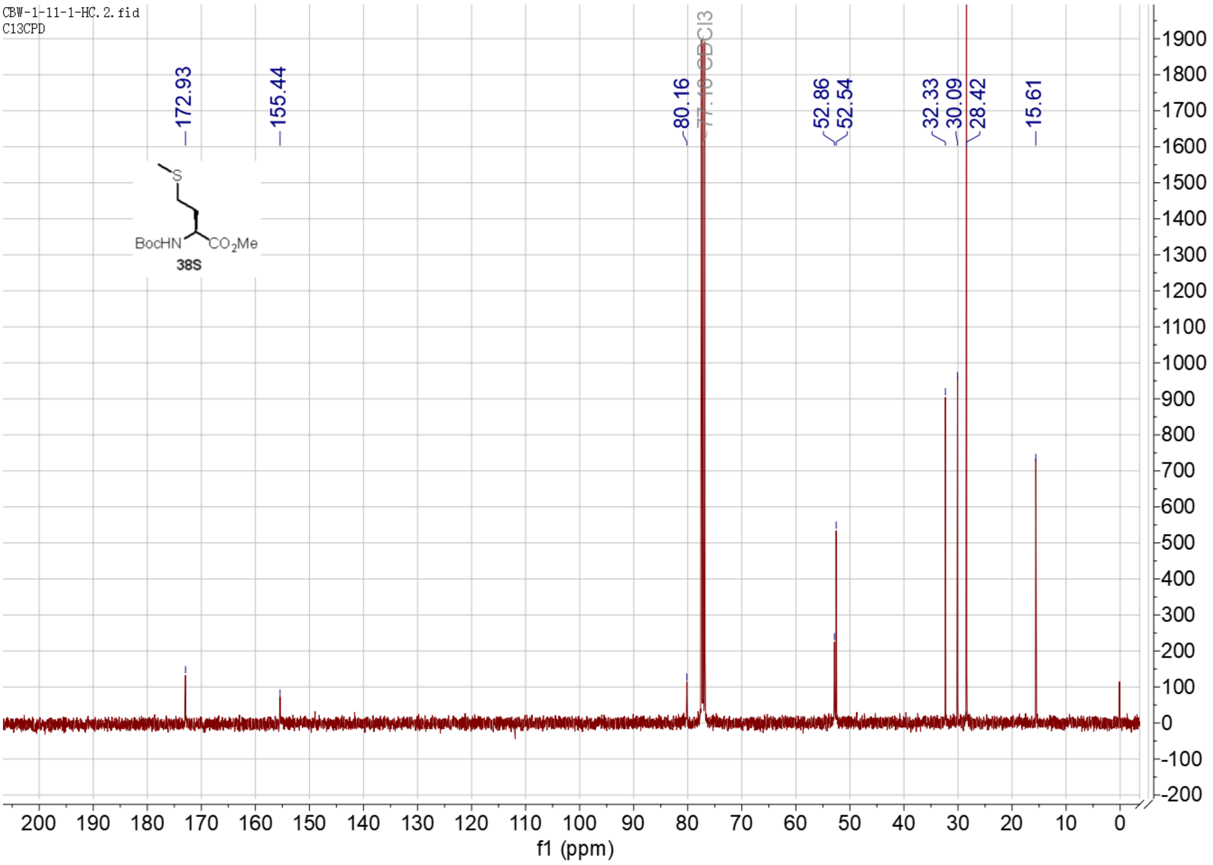

Supplementary Figure 87. ^{13}C NMR (101 MHz, $CDCl_3$, 293 K) spectrum of **38S**.

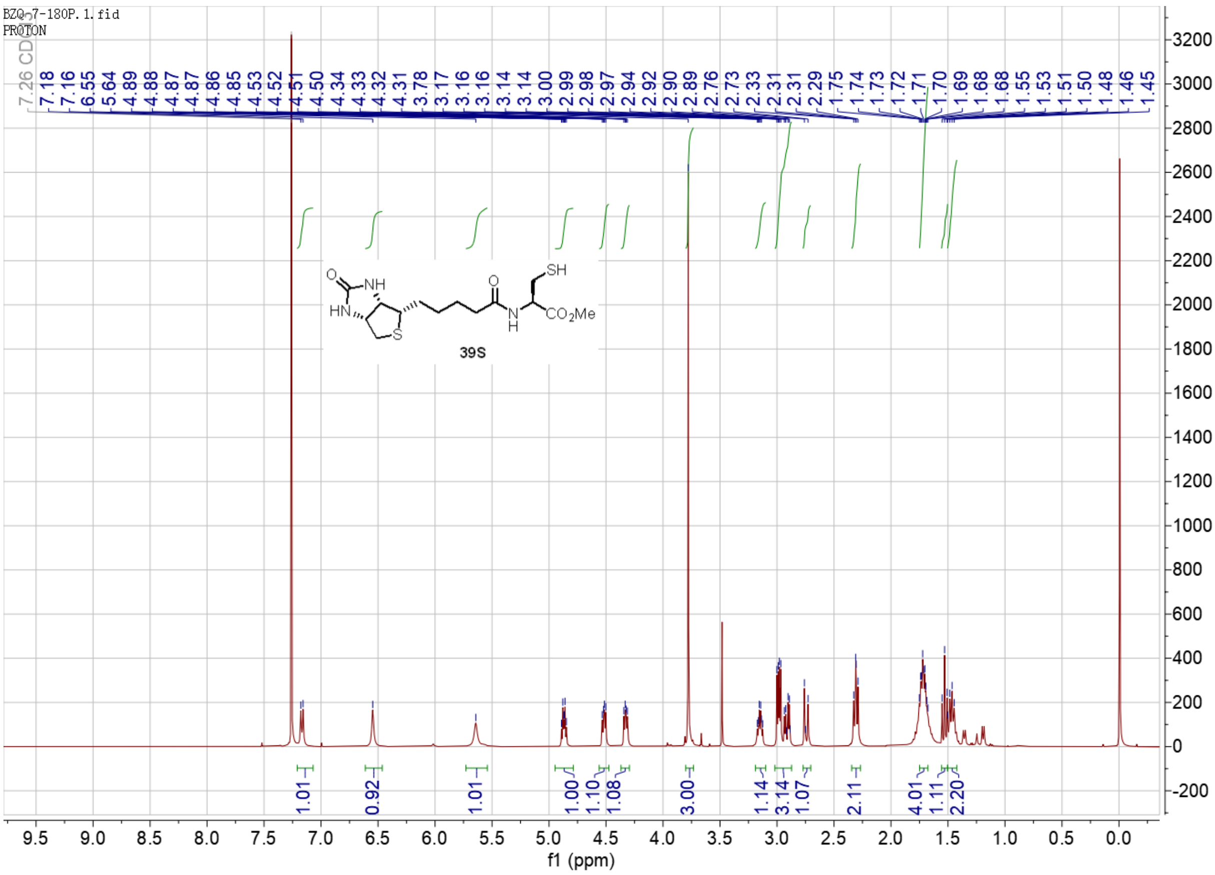

Supplementary Figure 88. ^{1}H NMR (400 MHz, $CDCl_3$, 293 K) spectrum of **39S**.

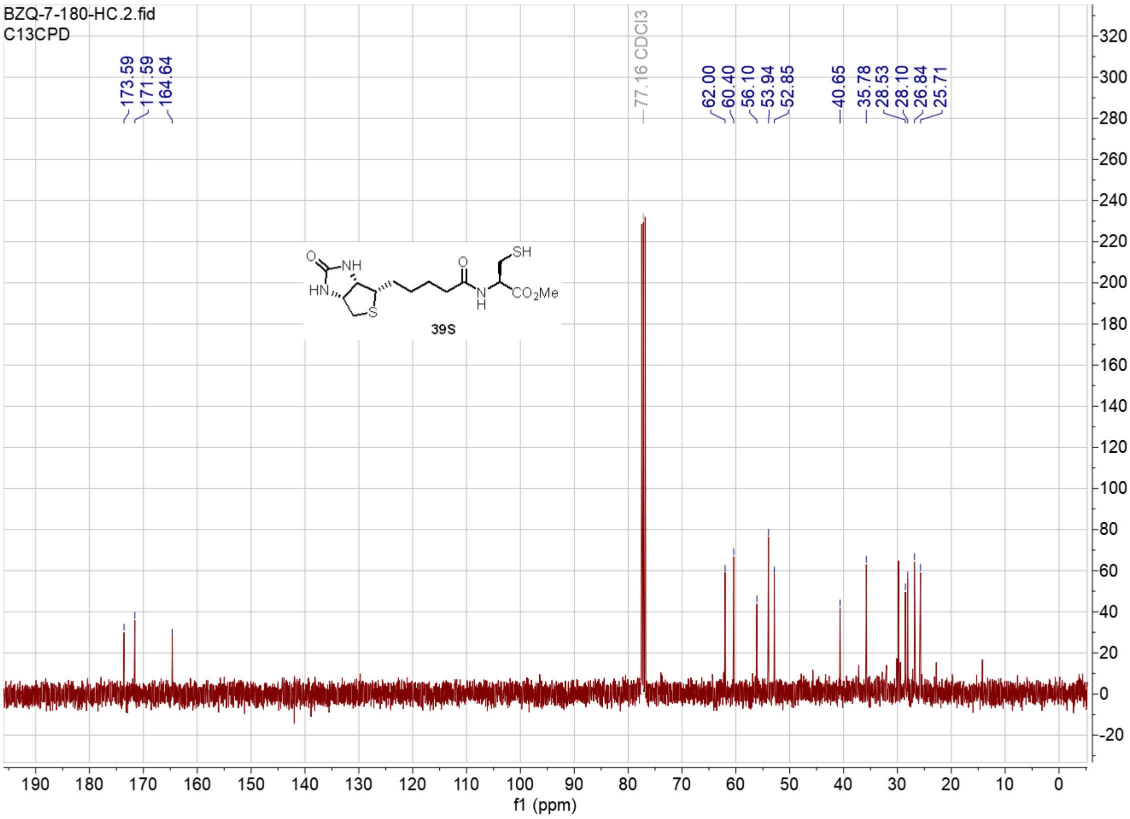

Supplementary Figure 89. ^{13}C NMR (101 MHz, $CDCl_3$, 293 K) spectrum of **39S**.

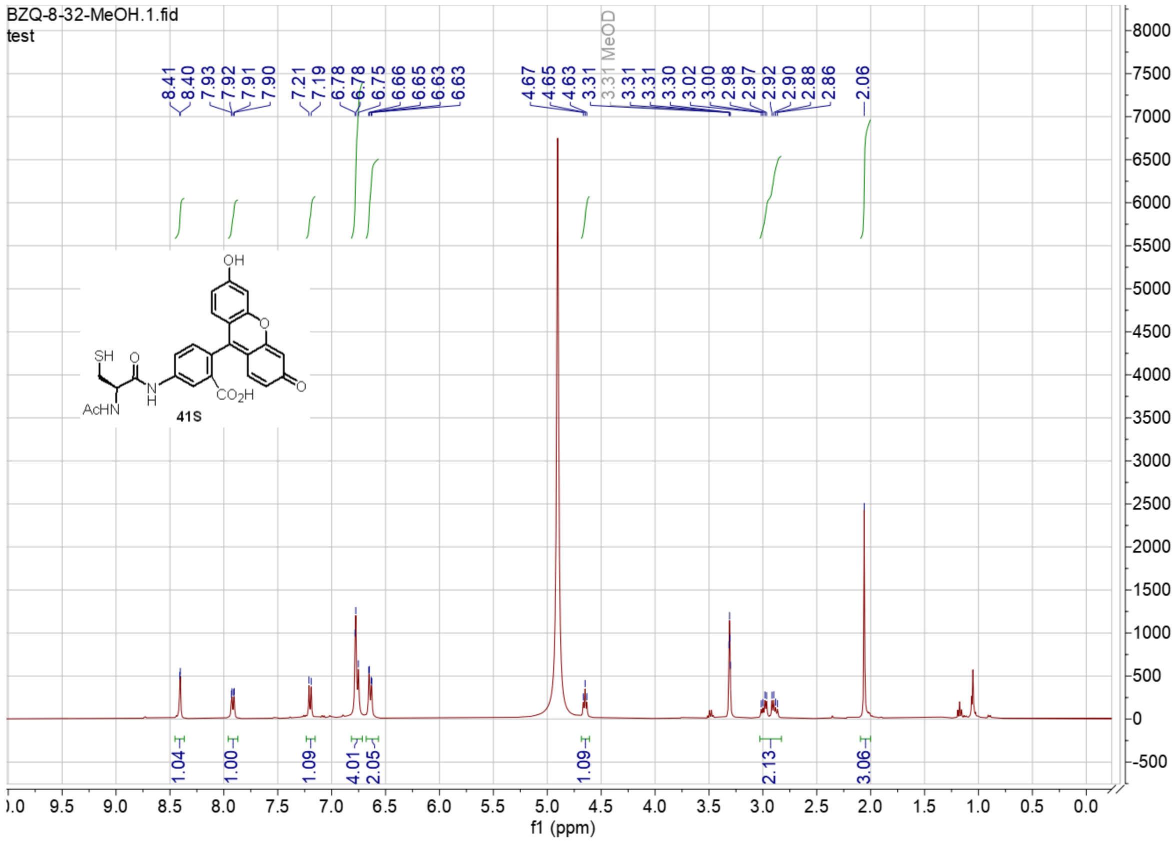

Supplementary Figure 90. ^{1}H NMR (400 MHz, Methanol-d_4, 293 K) spectrum of **41S**.

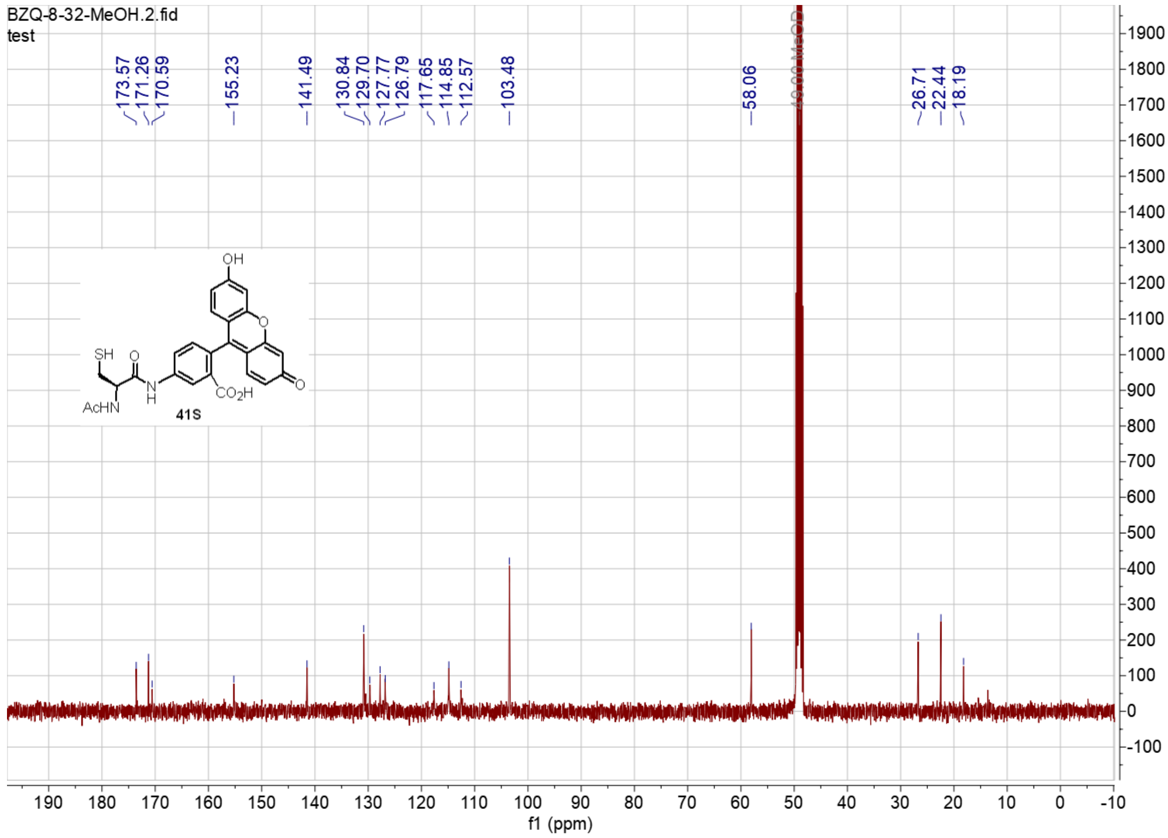

Supplementary Figure 91. ^{13}C NMR (101 MHz, Methanol-d_4, 293 K) spectrum of **41S**.

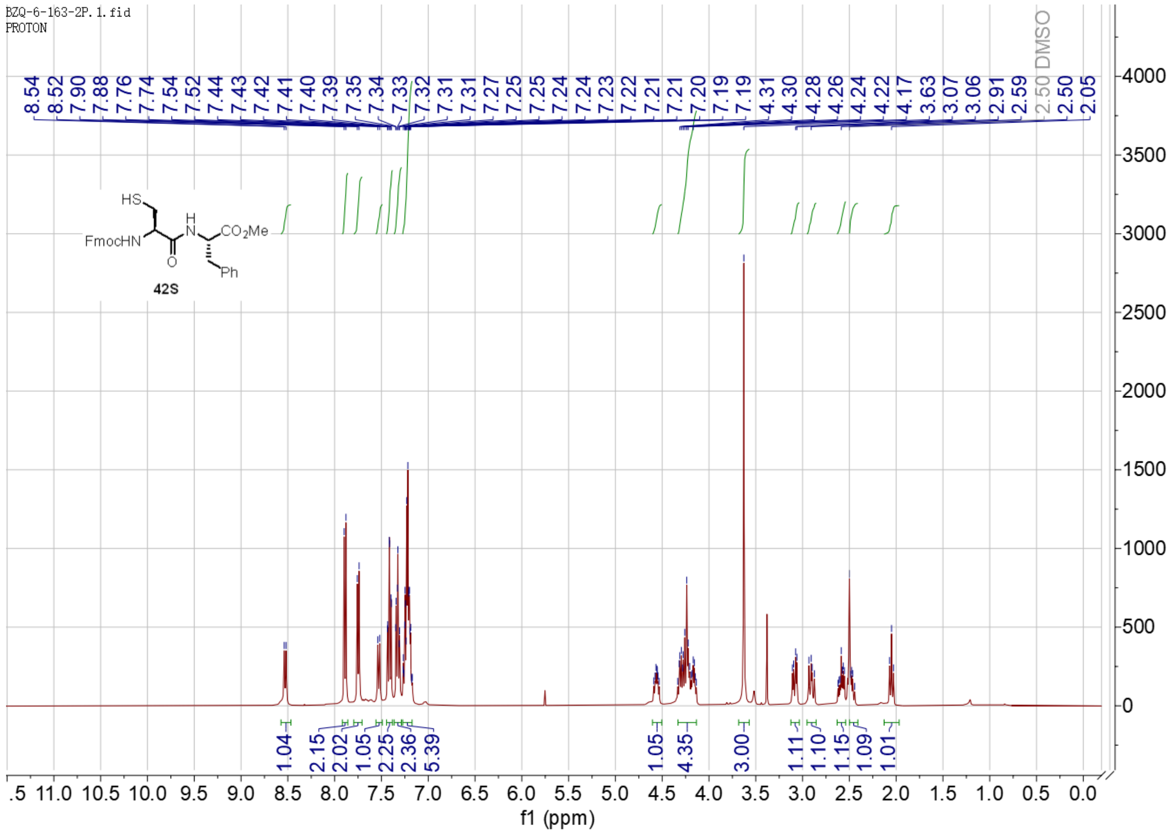

Supplementary Figure 92. ^{1}H NMR (400 MHz, DMSO-d_6, 293 K) spectrum of **42S**.

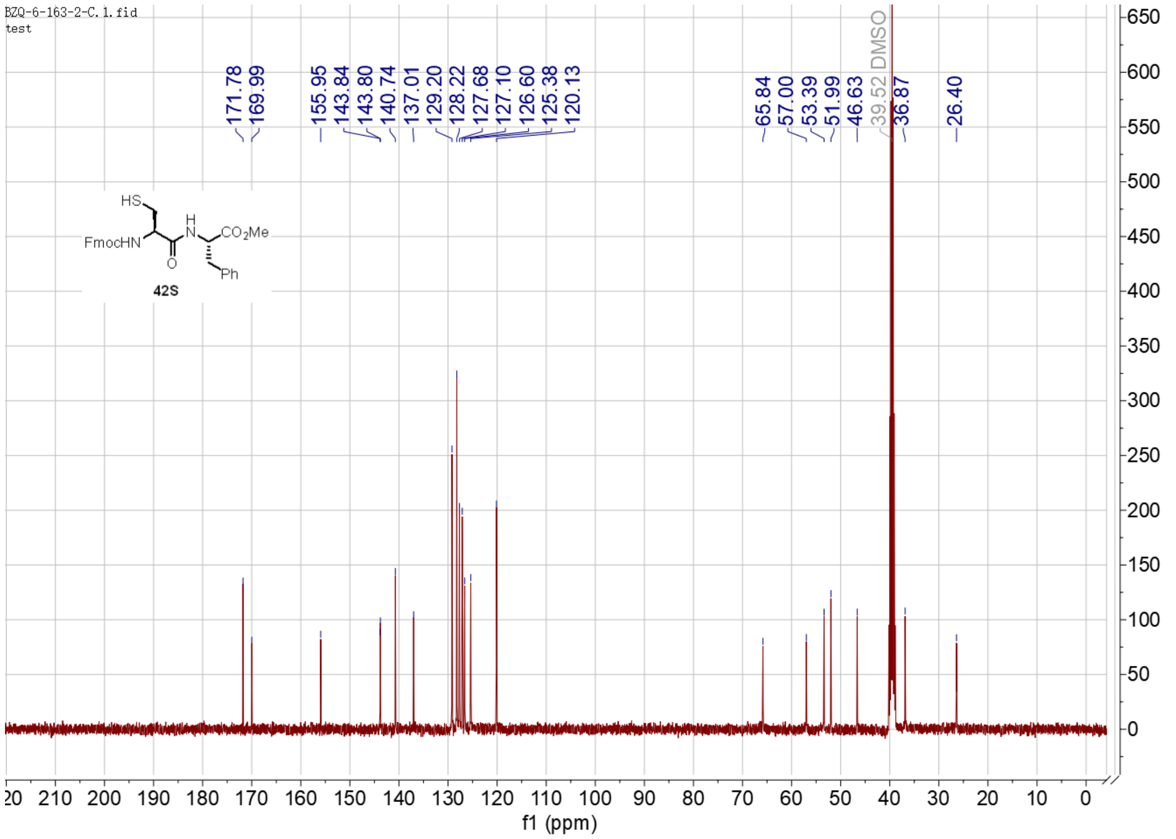

Supplementary Figure 93. ^{13}C NMR (101 MHz, DMSO-d_6, 293 K) spectrum of **42S**.

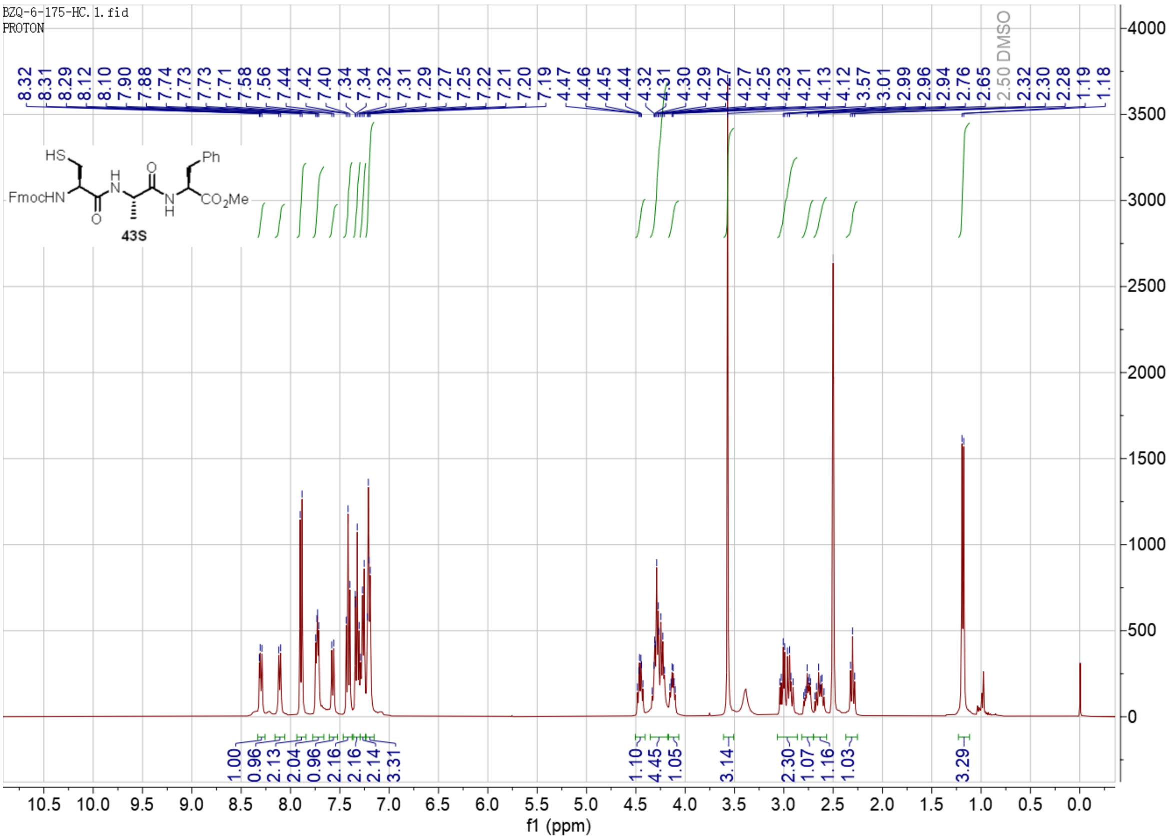

Supplementary Figure 94. ^{1}H NMR (400 MHz, DMSO-d_6, 293 K) spectrum of **43S**.

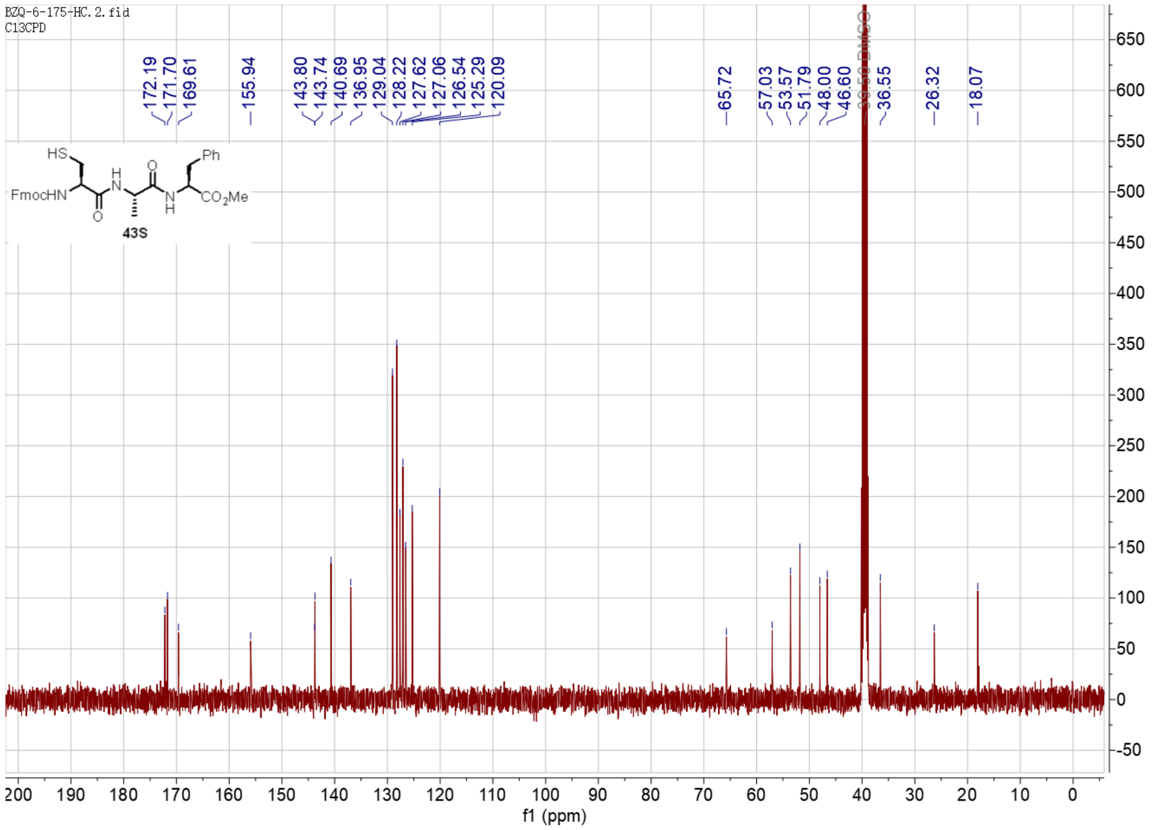

Supplementary Figure 95. ^{13}C NMR (101 MHz, DMSO-d_6, 293 K) spectrum of **43S**.

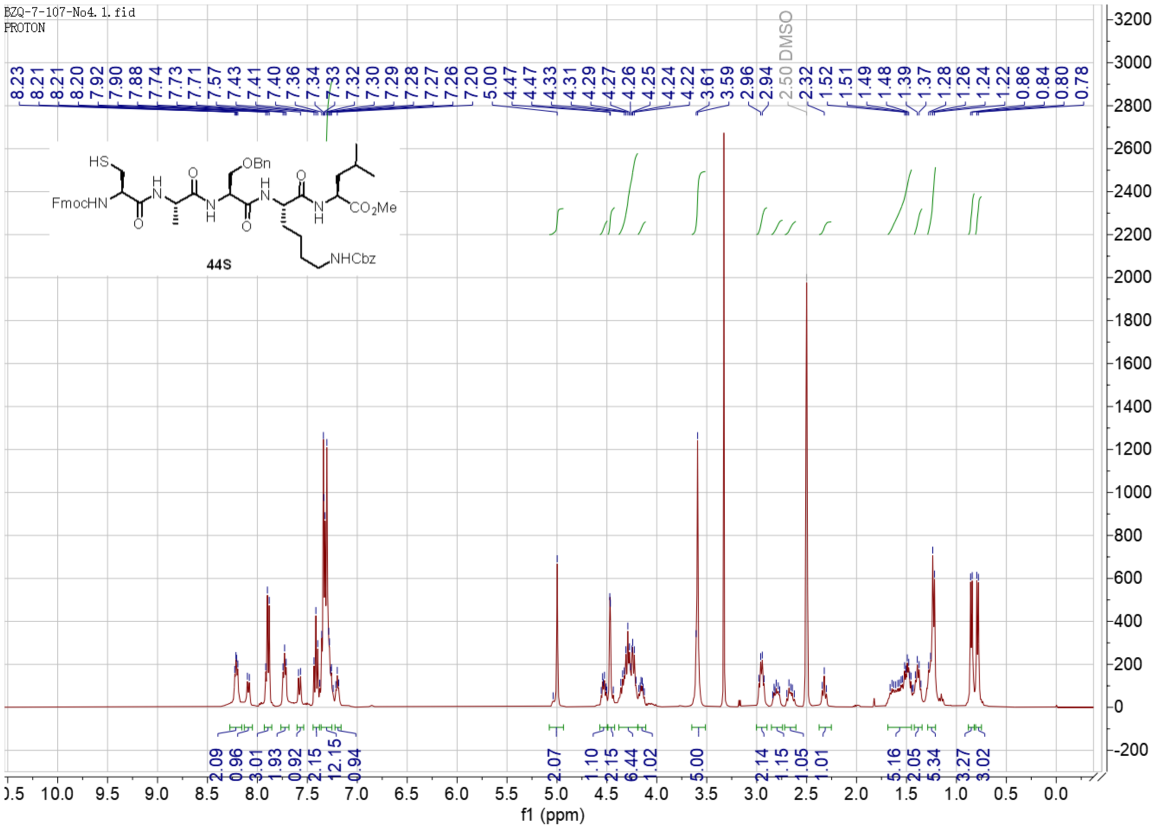

Supplementary Figure 96. ^{1}H NMR (400 MHz, DMSO-d_6, 293 K) spectrum of **44S**.

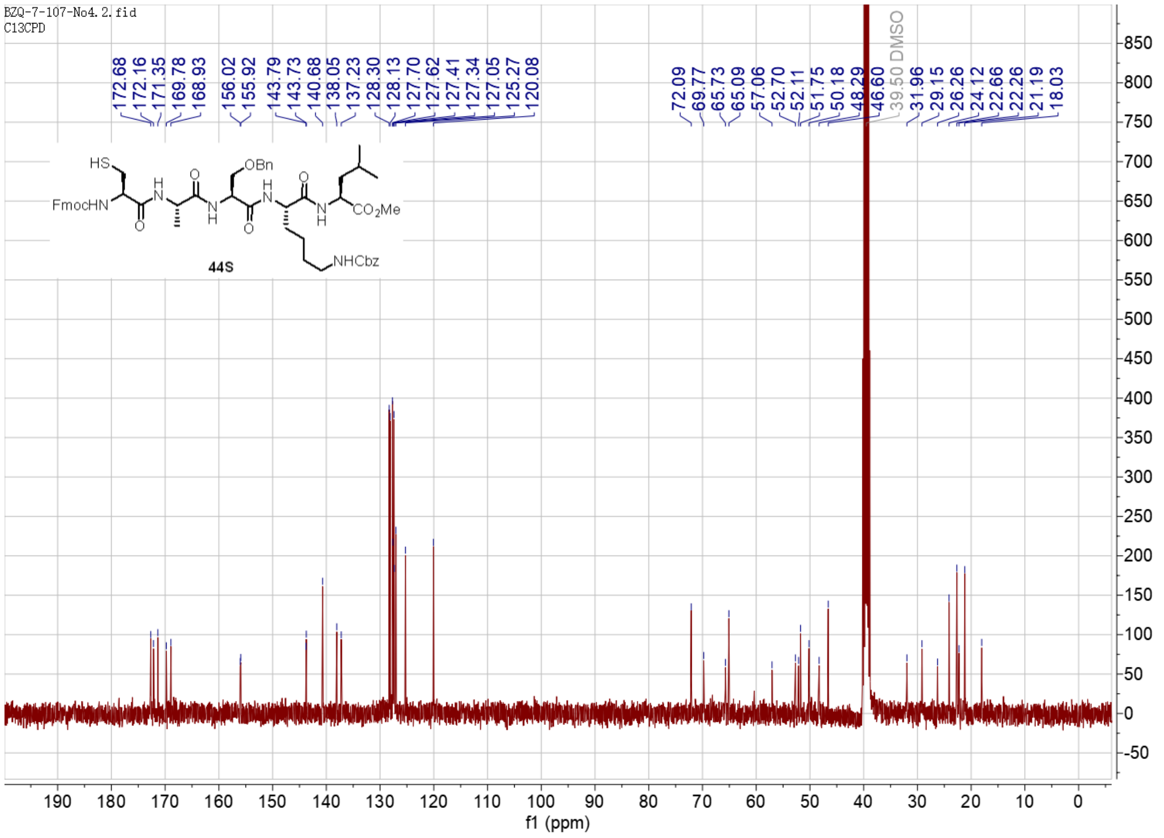

Supplementary Figure 97. ^{13}C NMR (101 MHz, DMSO-d_6, 293 K) spectrum of **44S**.

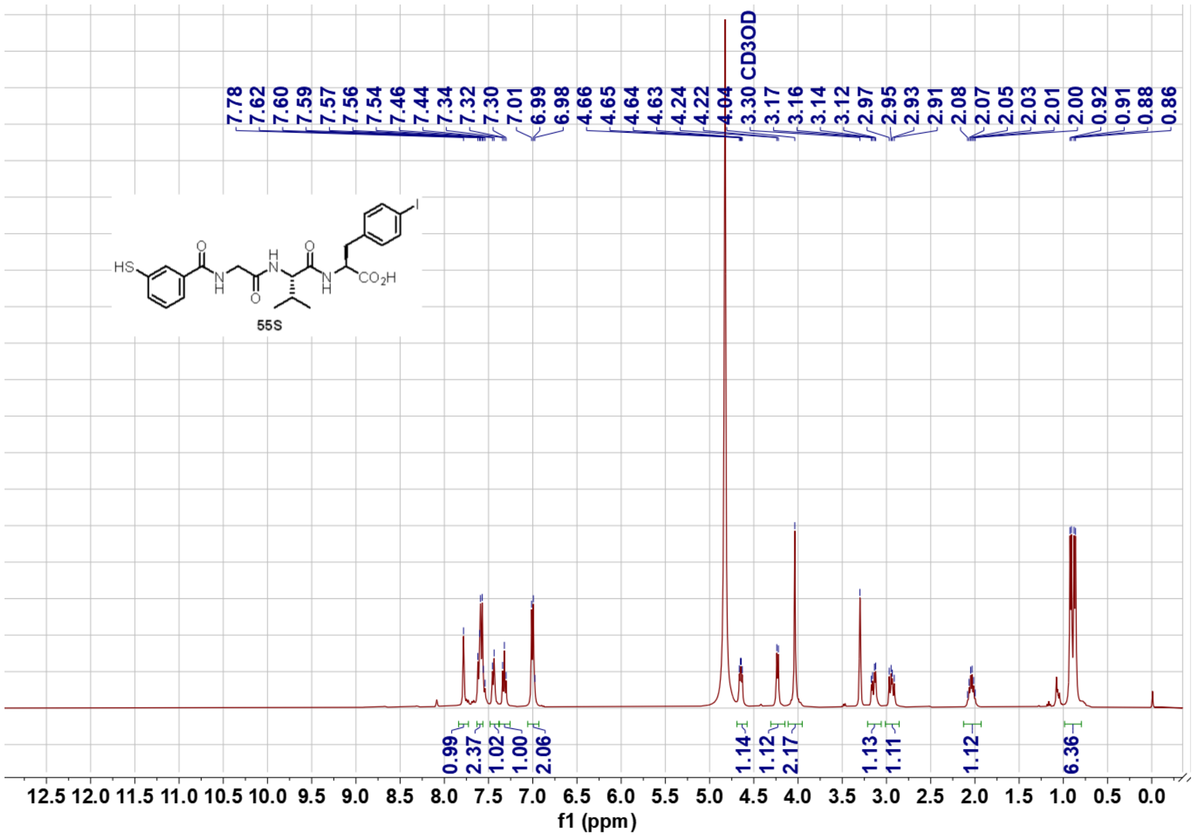

Supplementary Figure 98. ^{1}H NMR (400 MHz, Methanol-d_4, 293 K) spectrum of **55S**.

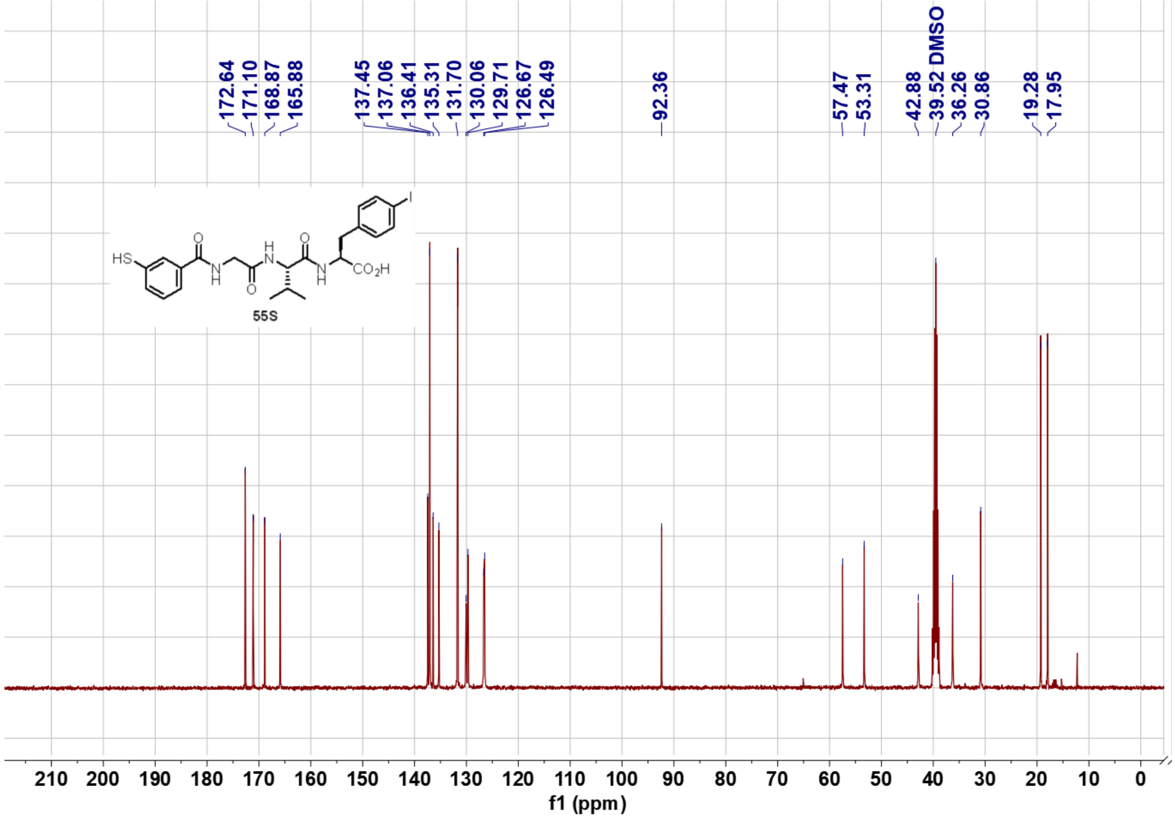

Supplementary Figure 99. ^{13}C NMR (101 MHz, DMSO-d_6, 293 K) spectrum of **55S**.

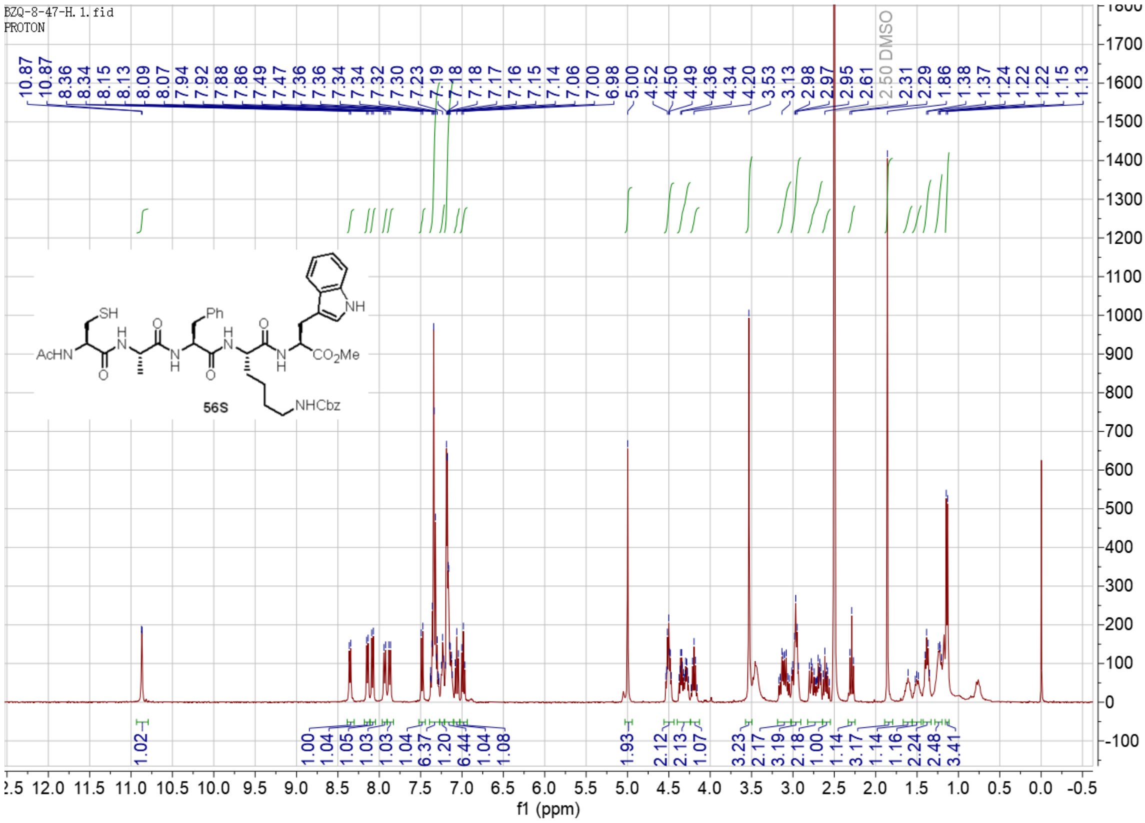

Supplementary Figure 100. ^{1}H NMR (400 MHz, DMSO-d_6, 293 K) spectrum of **56S**.

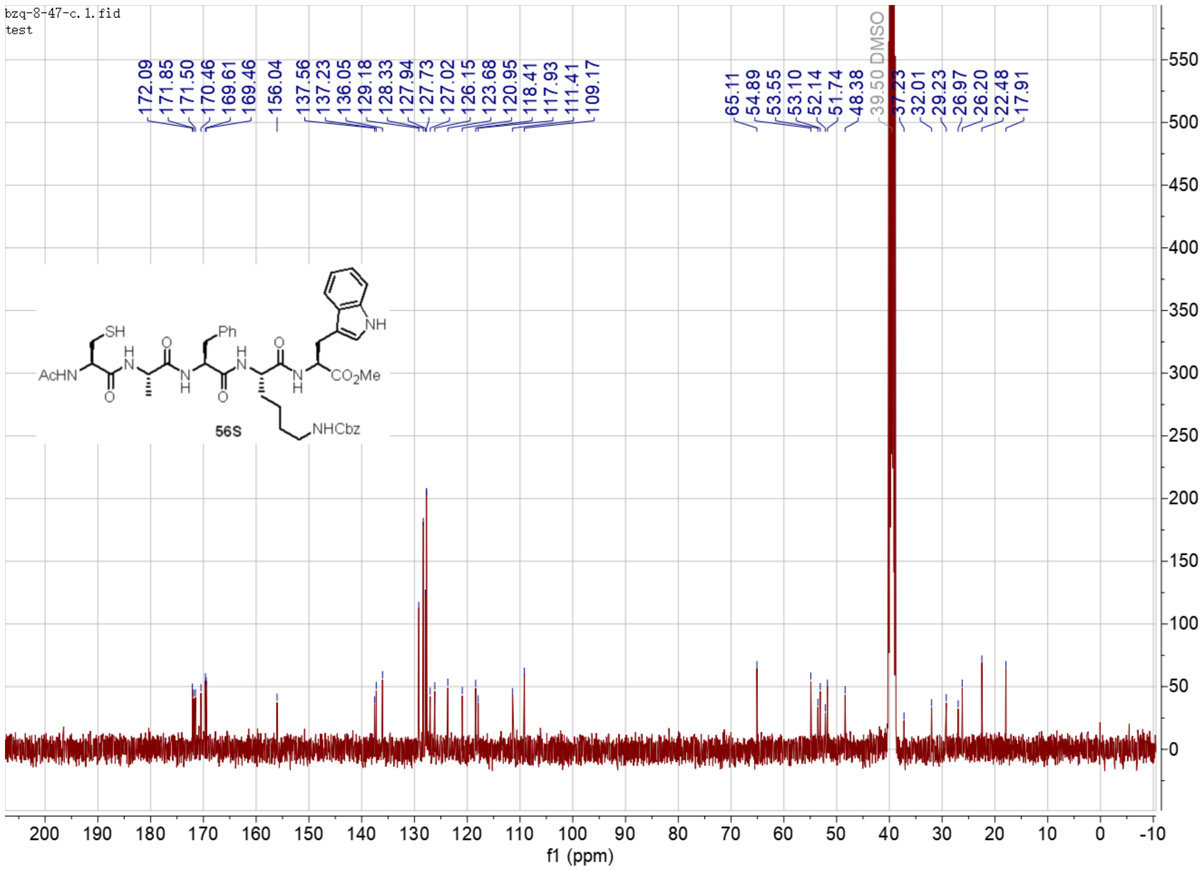

Supplementary Figure 101. ^{13}C NMR (101 MHz, DMSO-d_6, 293 K) spectrum of **56S**.

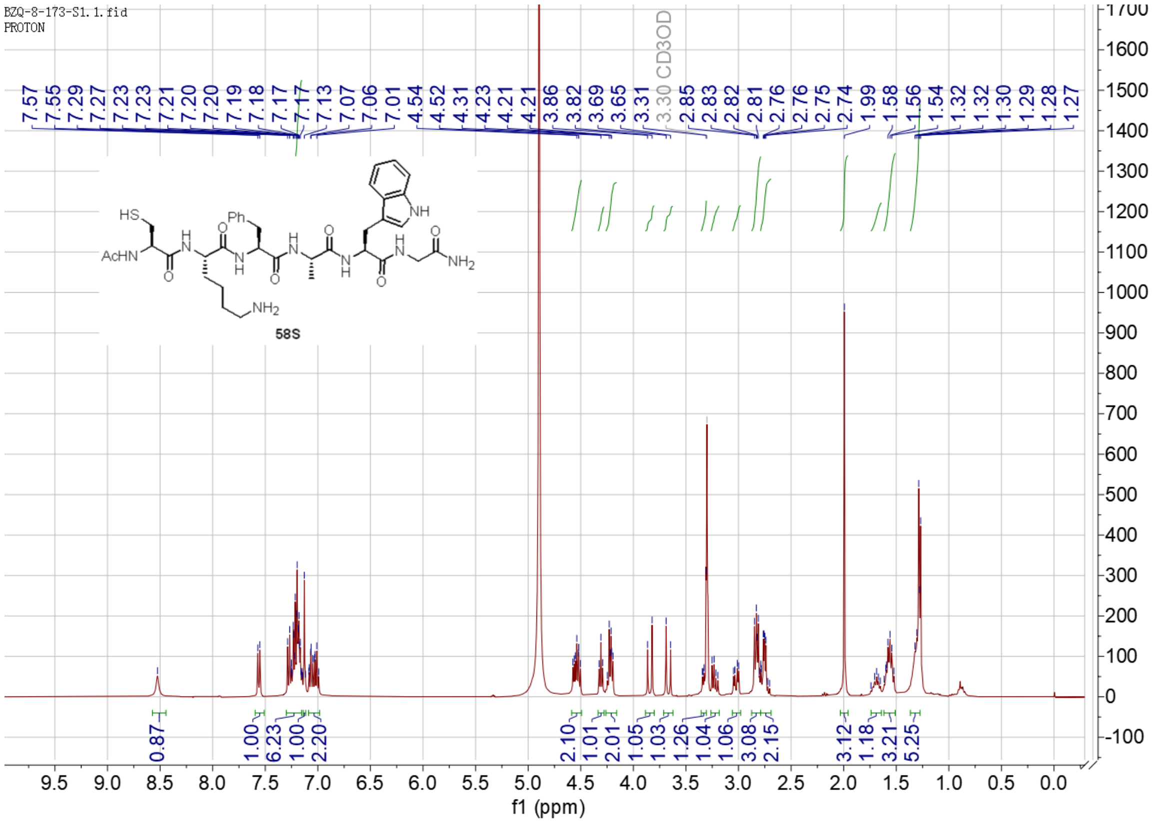

Supplementary Figure 102. ^{1}H NMR (400 MHz, Methanol-d_4, 293 K) spectrum of **58S**.

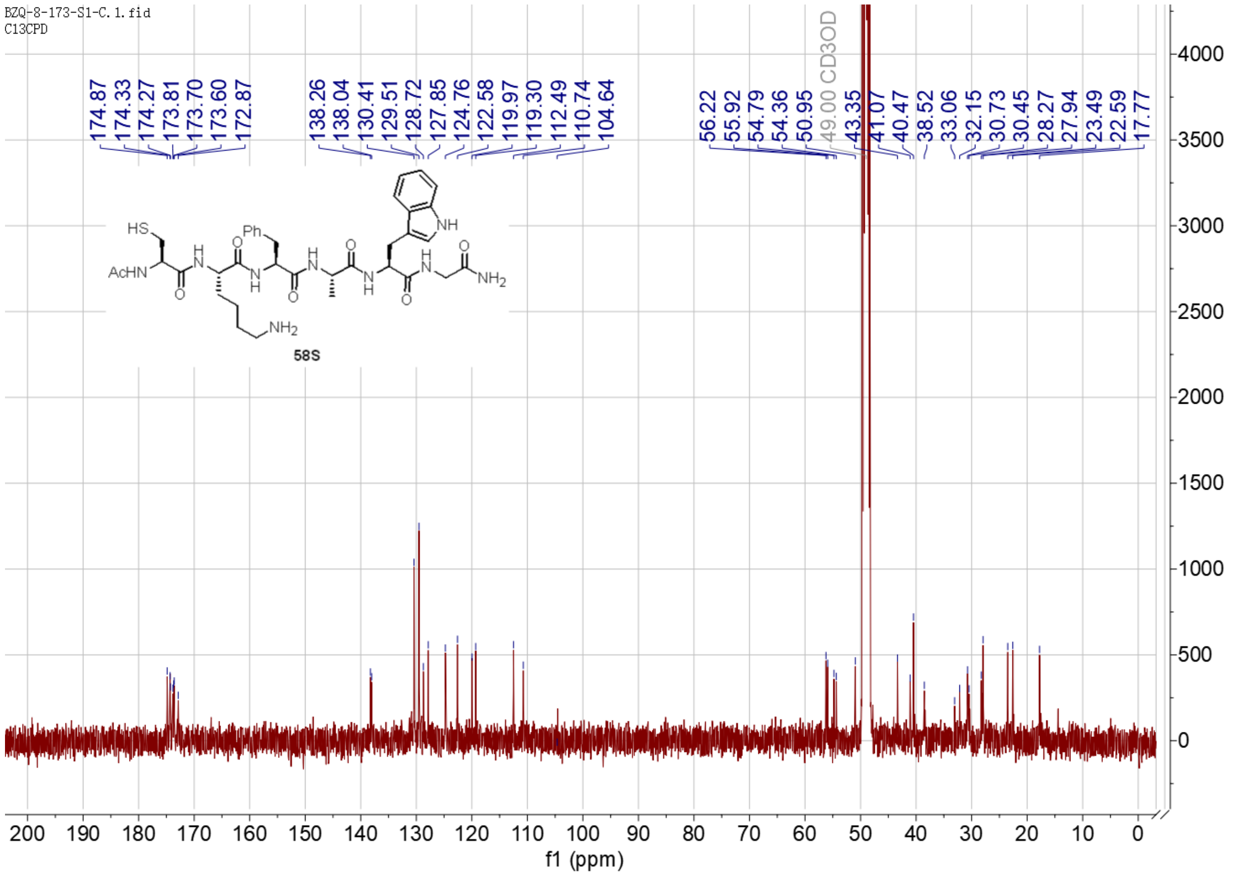

Supplementary Figure 103. ^{13}C NMR (101 MHz, Methanol-d_4, 293 K) spectrum of **58S**.

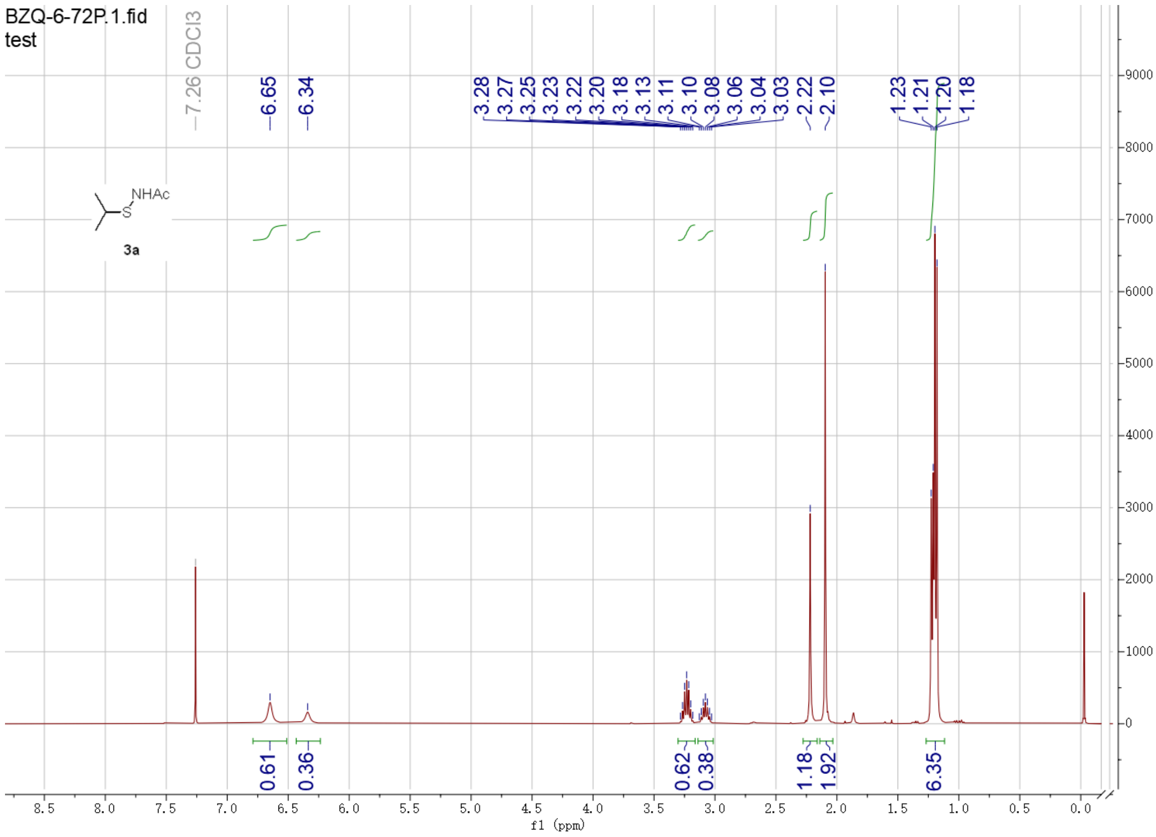

Supplementary Figure 104. ^{1}H NMR (400 MHz, $CDCl_3$, 293 K) spectrum of **3a**.

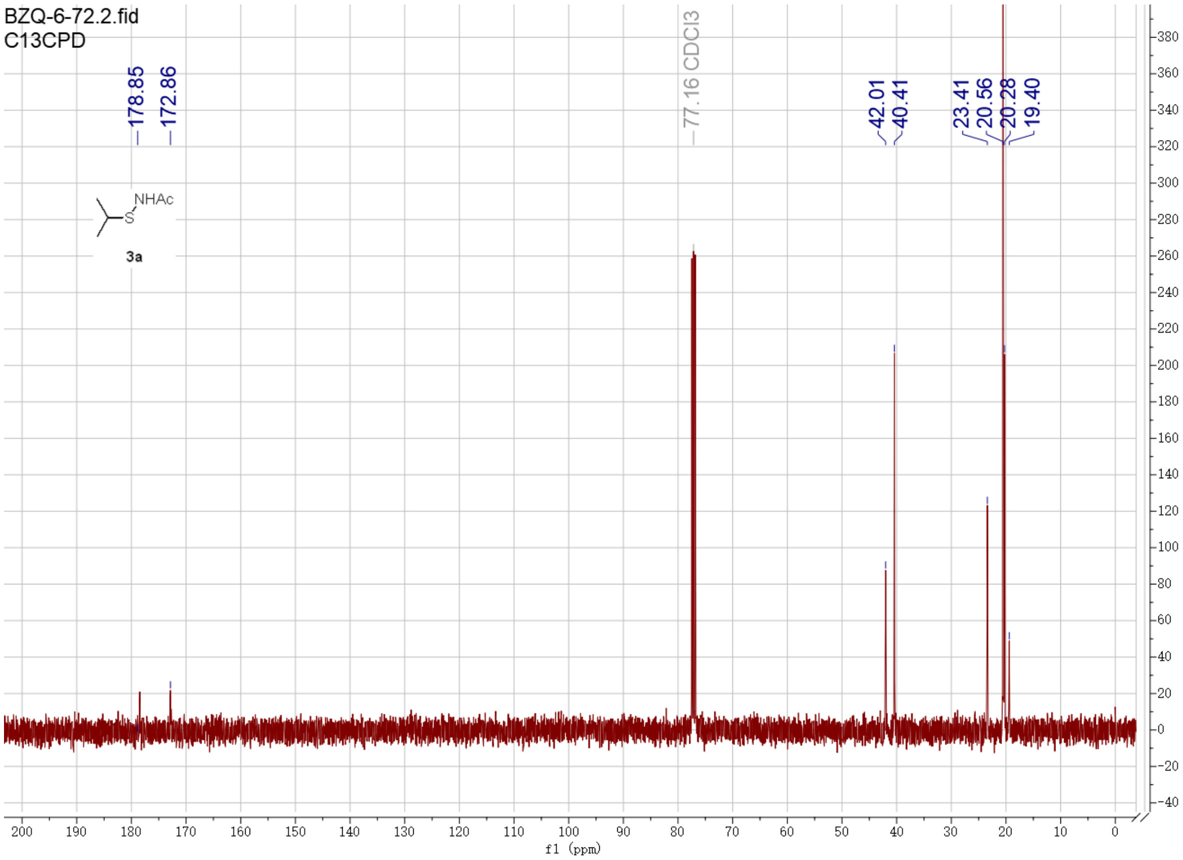

Supplementary Figure 105. ^{13}C NMR (101 MHz, $CDCl_3$, 293 K) spectrum of **3a**.

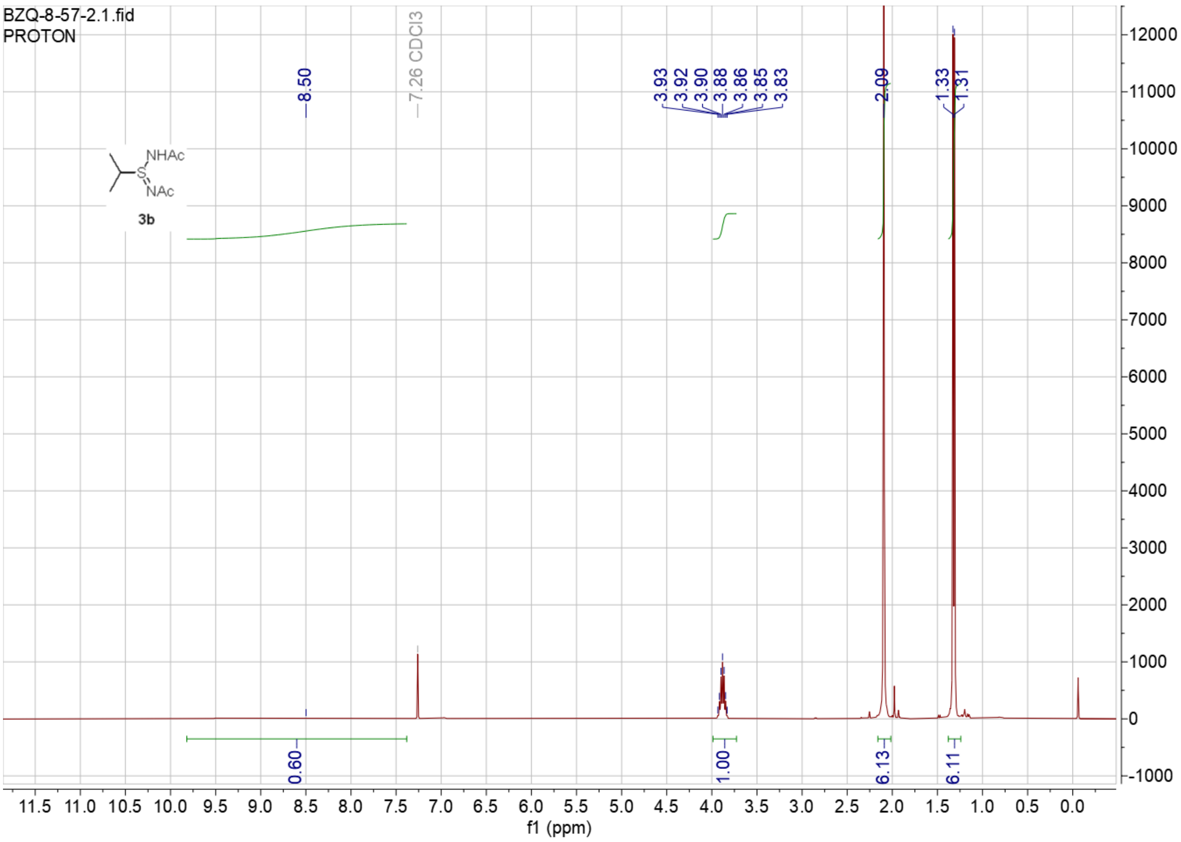

Supplementary Figure 106. ^{1}H NMR (400 MHz, $CDCl_3$, 293 K) spectrum of **3b**.

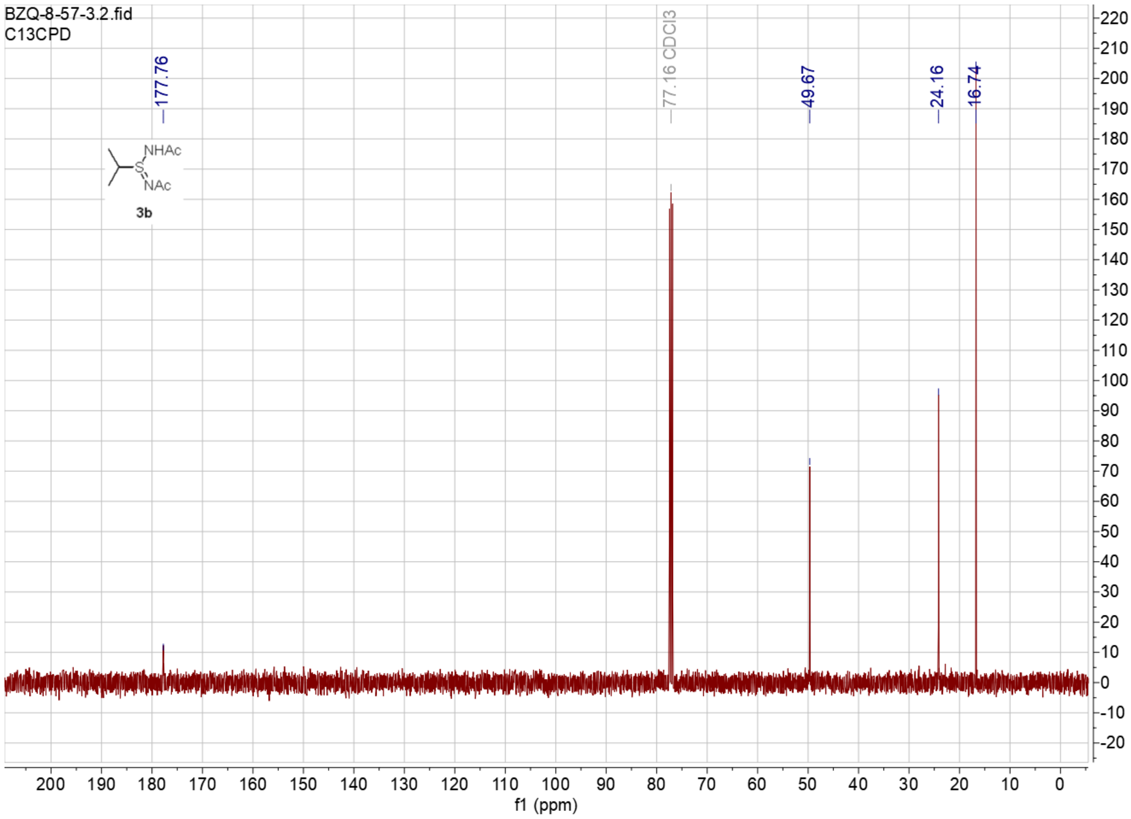

Supplementary Figure 107. ^{13}C NMR (101 MHz, $CDCl_3$, 293 K) spectrum of **3b**.

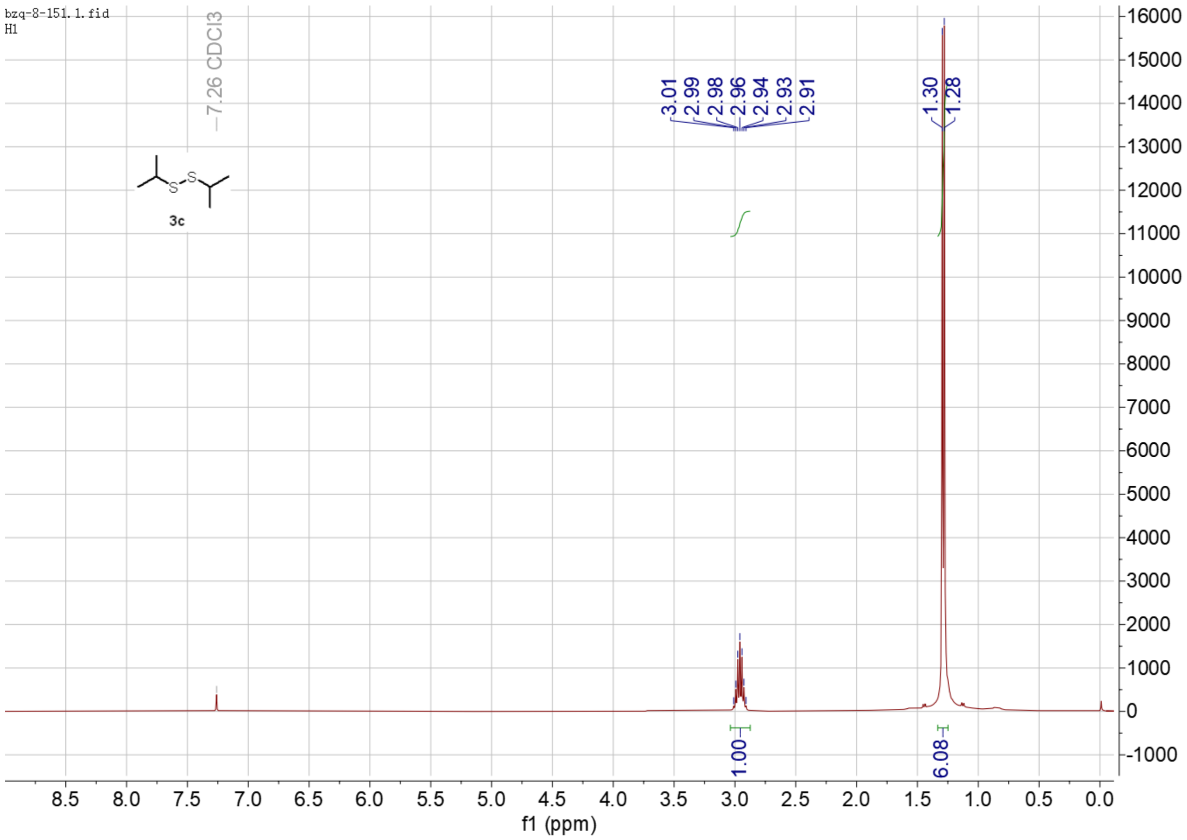

Supplementary Figure 108. ^{1}H NMR (400 MHz, $CDCl_3$, 293 K) spectrum of **3c**.

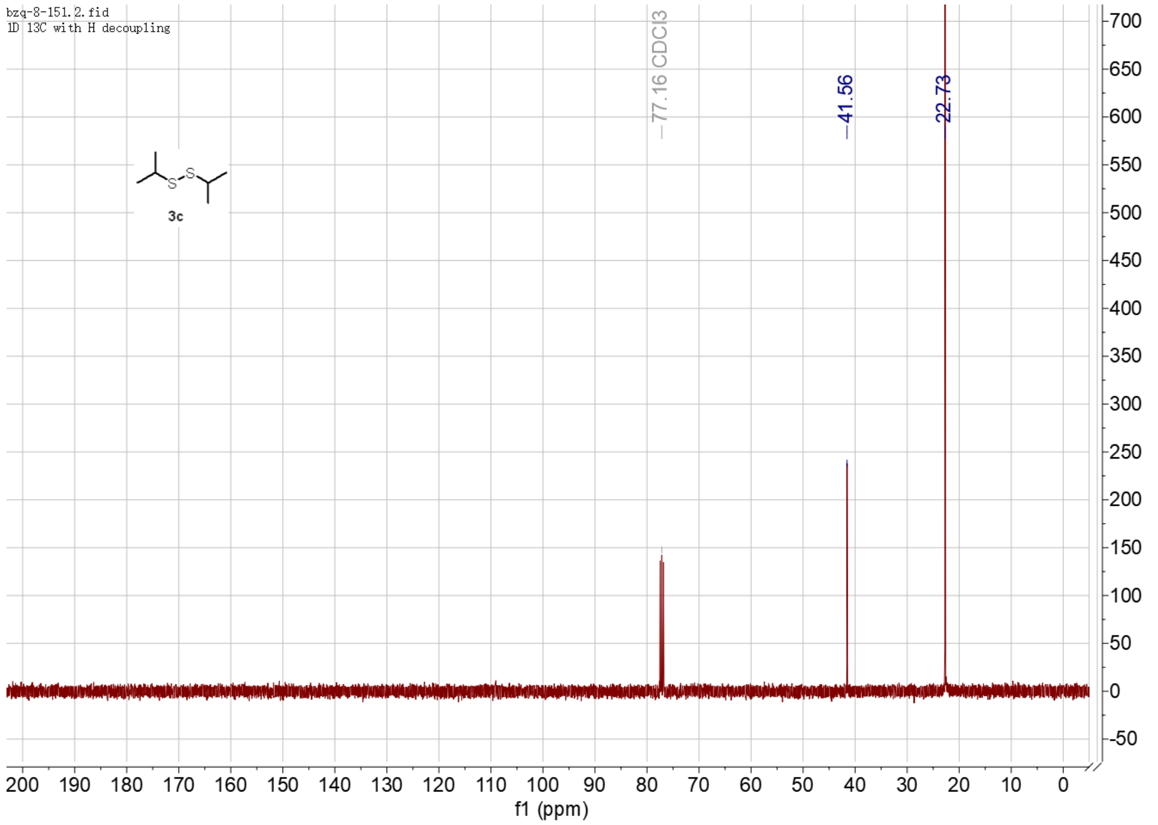

Supplementary Figure 109. ^{13}C NMR (101 MHz, $CDCl_3$, 293 K) spectrum of **3c**.

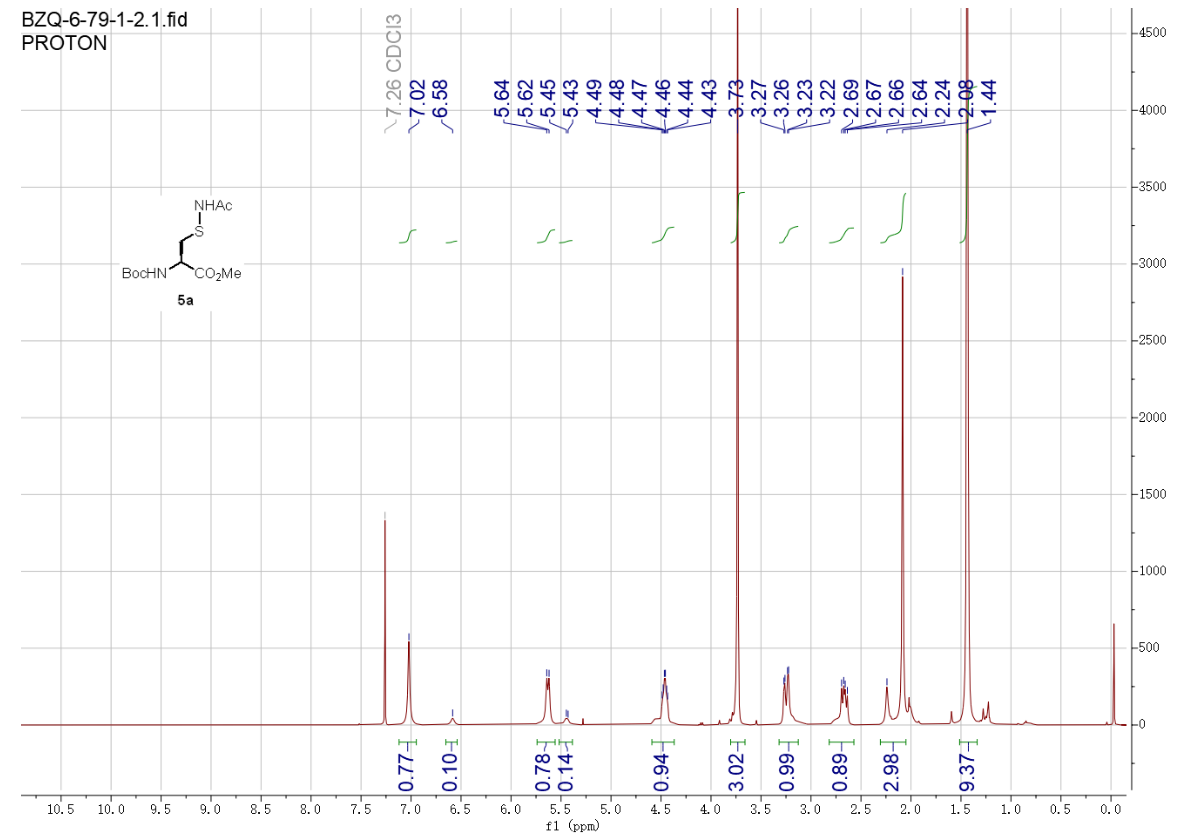

Supplementary Figure 110. ${}^{1}H$ NMR (400 MHz, $CDCl_3$, 293 K) spectrum of **5a**.

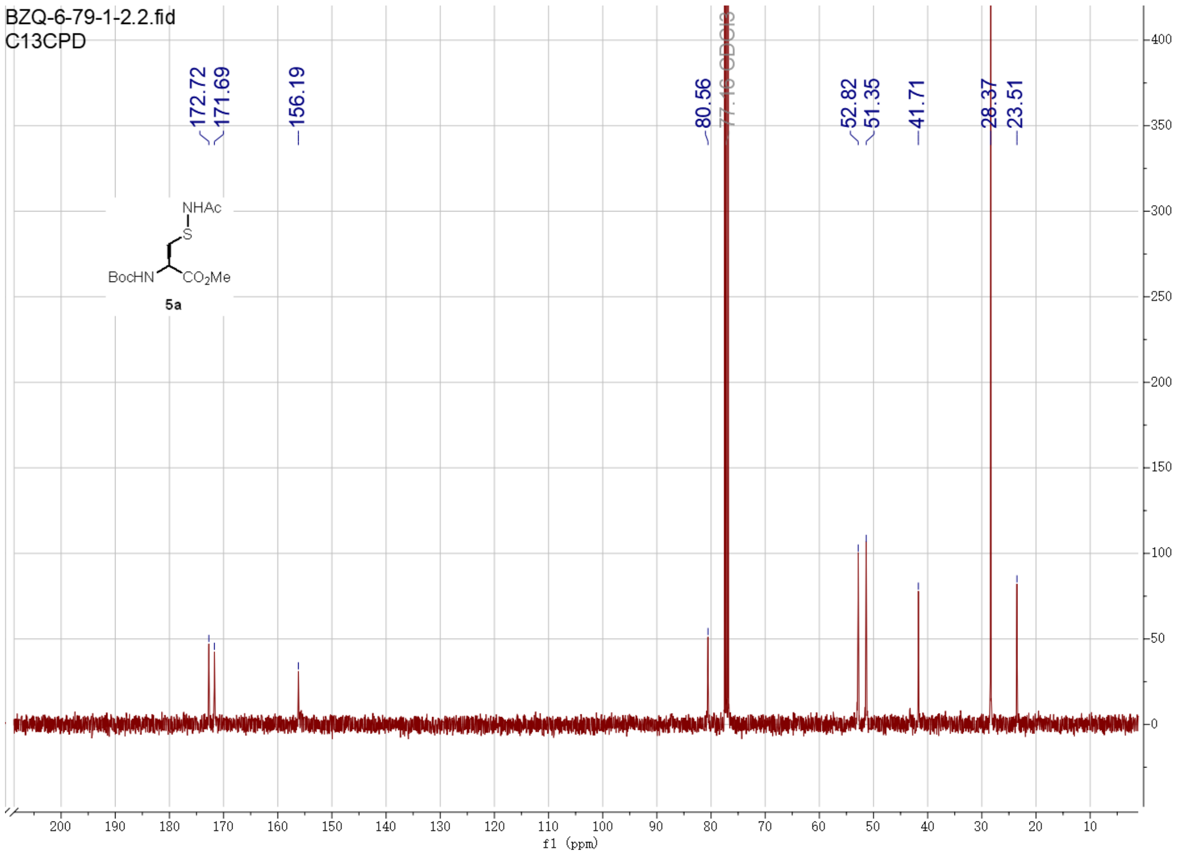

Supplementary Figure 111. ${}^{13}C$ NMR (101 MHz, $CDCl_3$, 293 K) spectrum of **5a**.

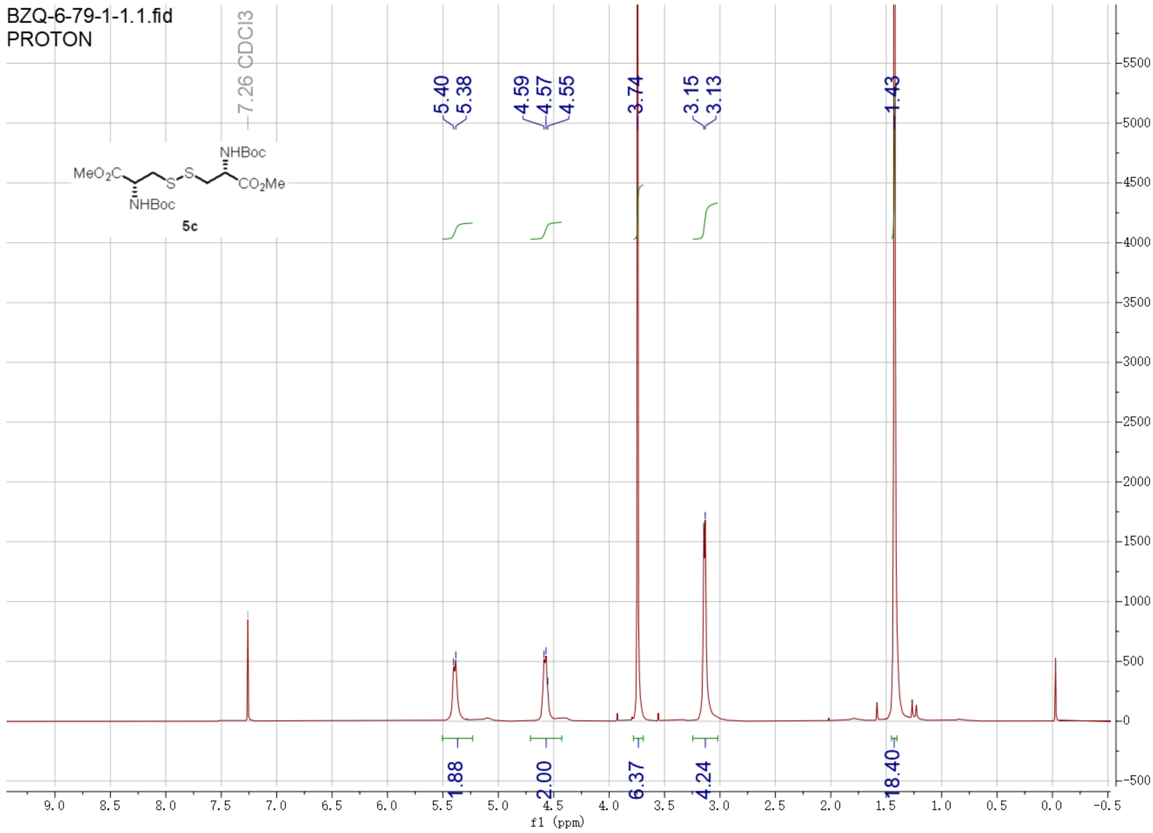

Supplementary Figure 112. ^{1}H NMR (400 MHz, $CDCl_3$, 293 K) spectrum of **5c**.

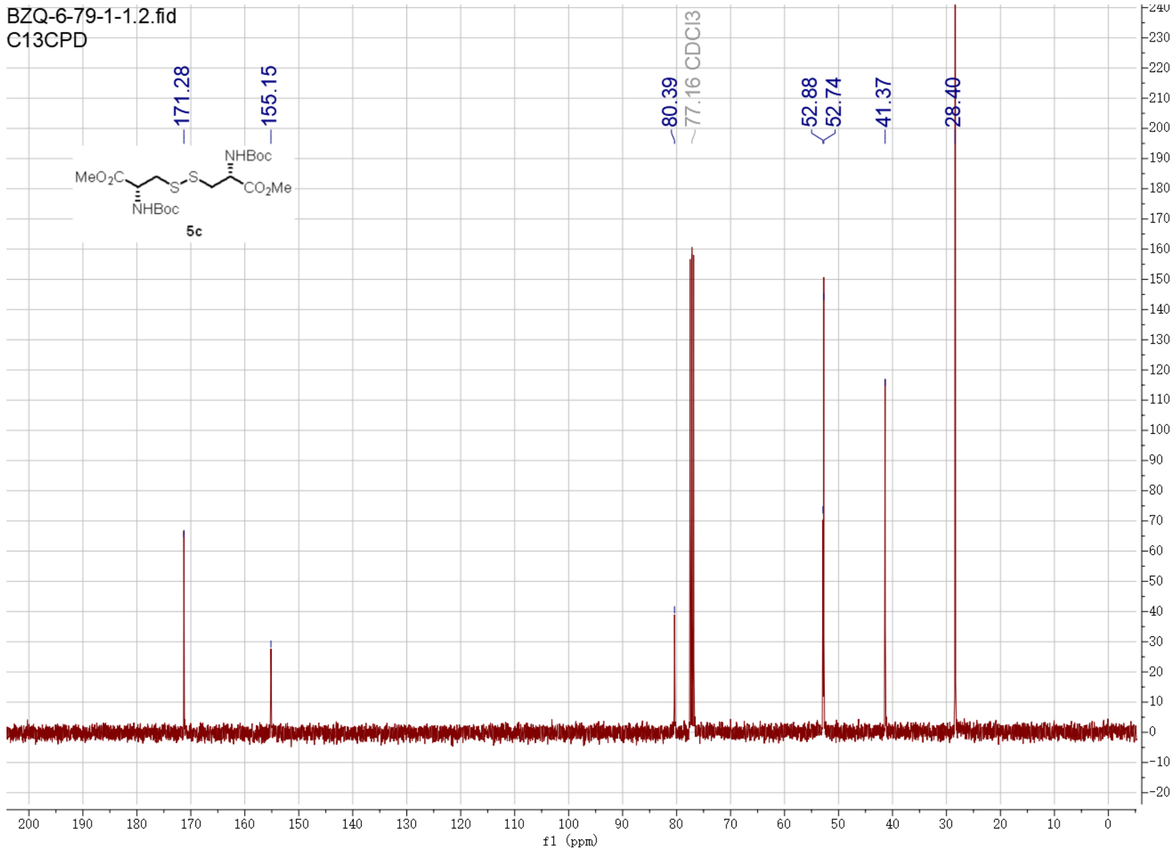

Supplementary Figure 113. ^{13}C NMR (101 MHz, $CDCl_3$, 293 K) spectrum of **5c**.

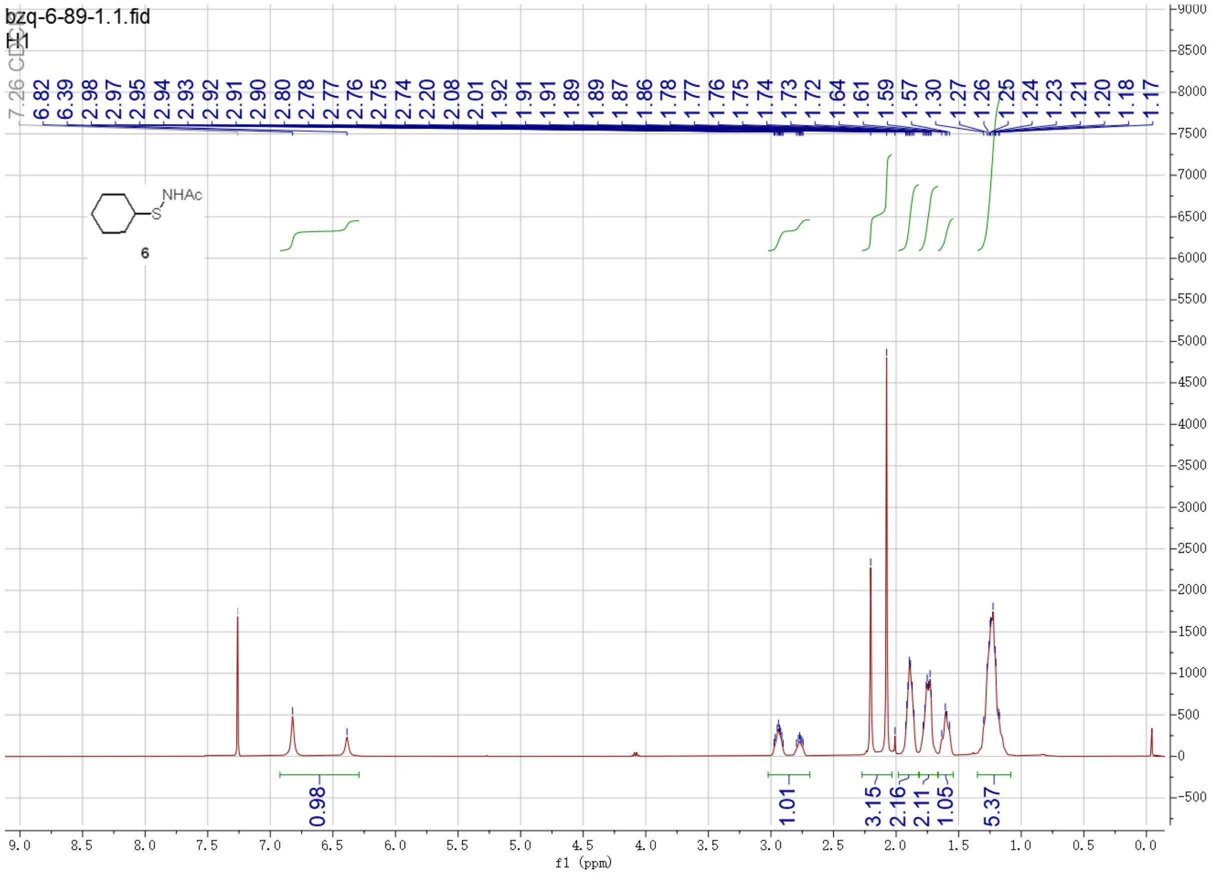

Supplementary Figure 114. ^{1}H NMR (400 MHz, $CDCl_3$, 293 K) spectrum of **6**.

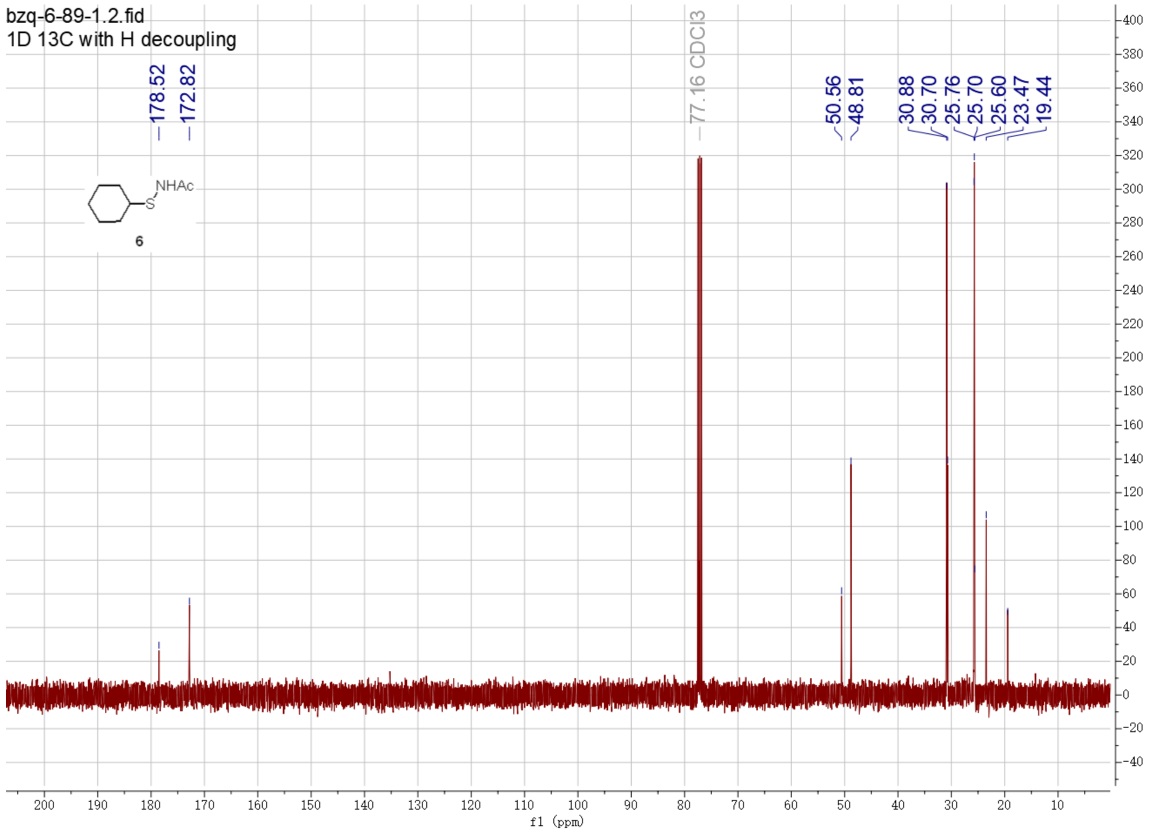

Supplementary Figure 115. ^{13}C NMR (101 MHz, $CDCl_3$, 293 K) spectrum of **6**.

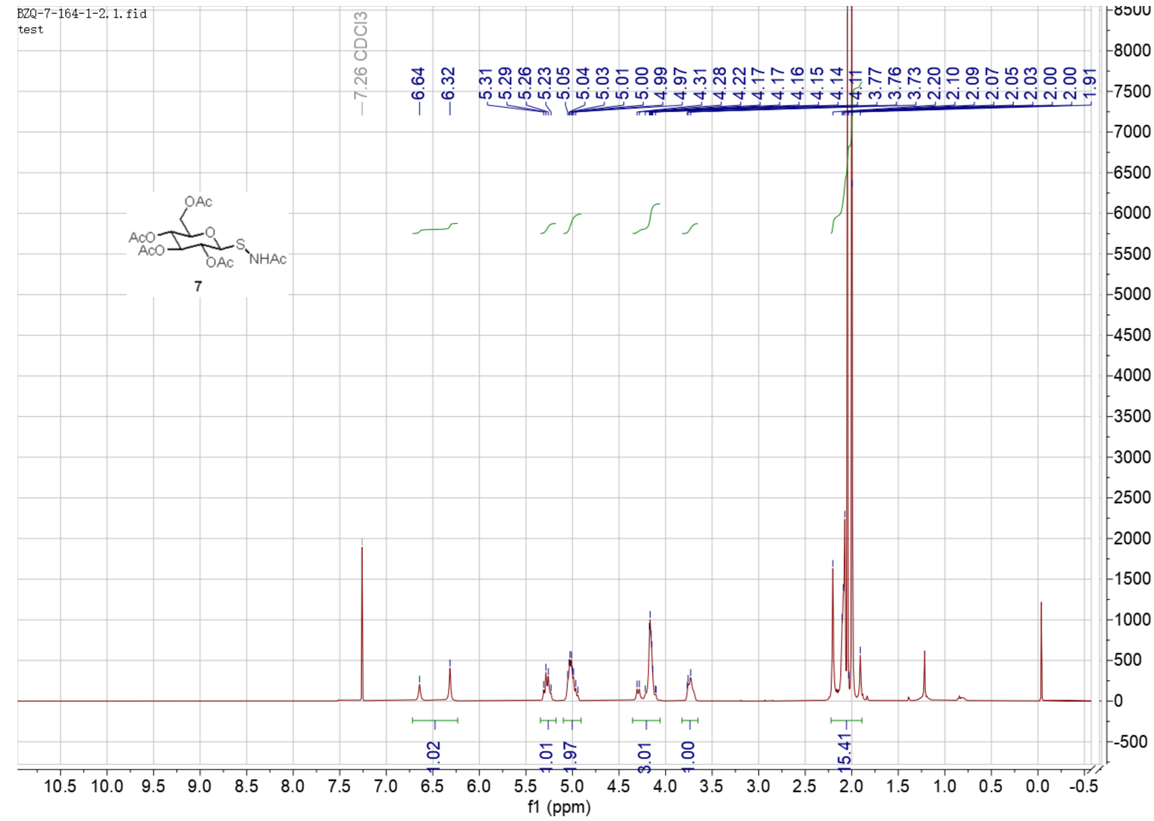

Supplementary Figure 116. ^{1}H NMR (400 MHz, $CDCl_3$, 293 K) spectrum of **7**.

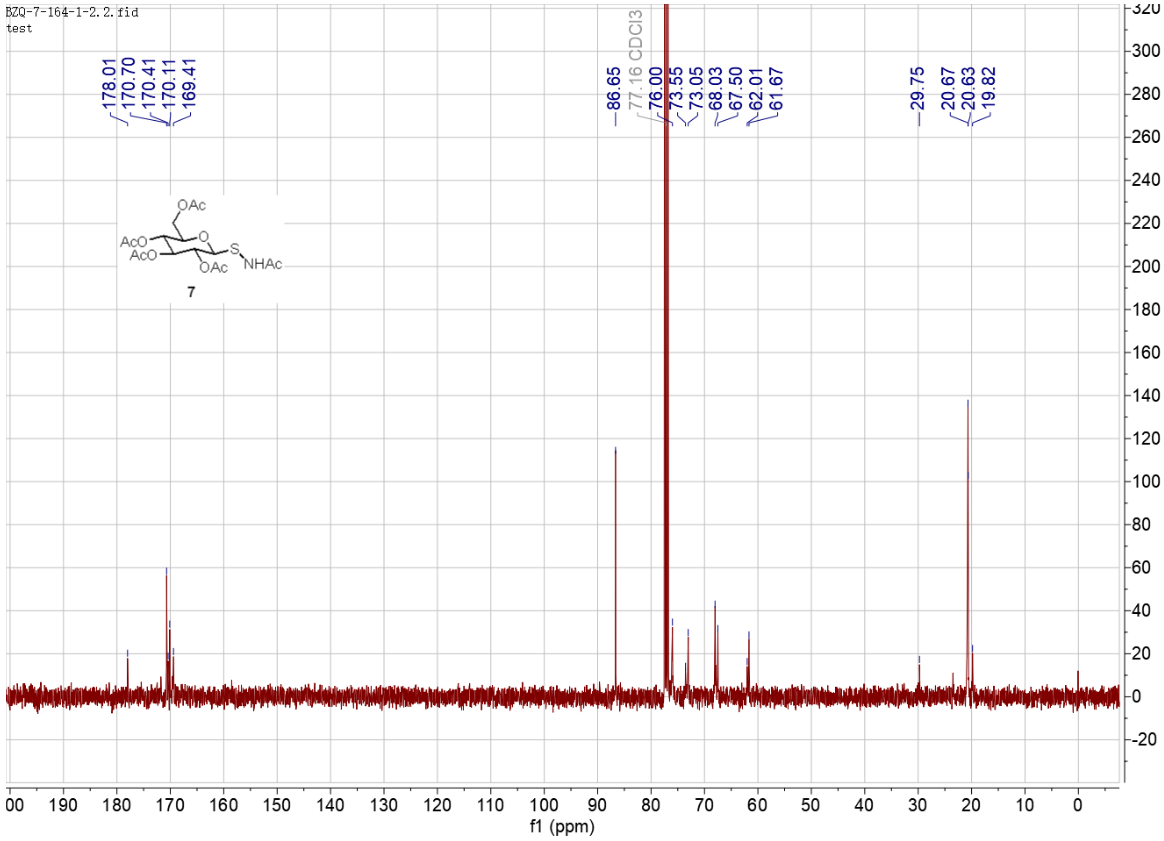

Supplementary Figure 117. ^{13}C NMR (101 MHz, $CDCl_3$, 293 K) spectrum of **7**.

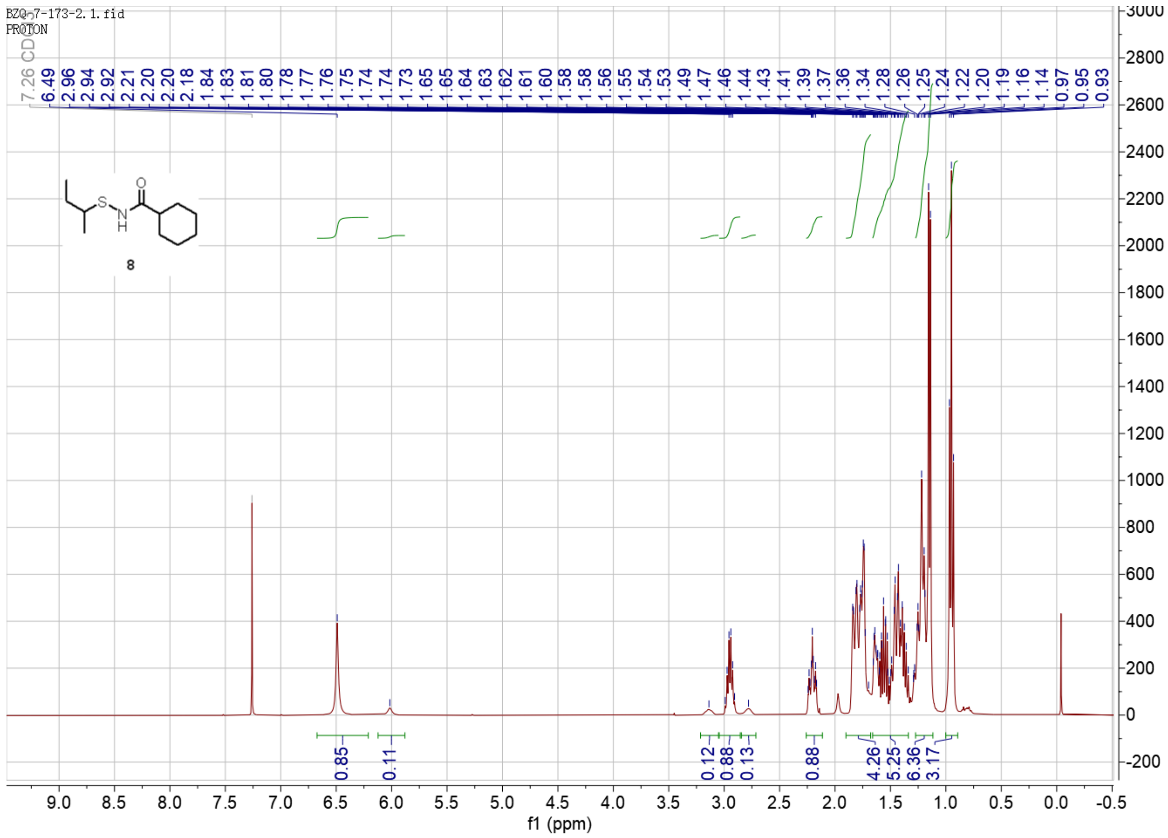

Supplementary Figure 118. ^{1}H NMR (400 MHz, $CDCl_3$, 293 K) spectrum of **8**.

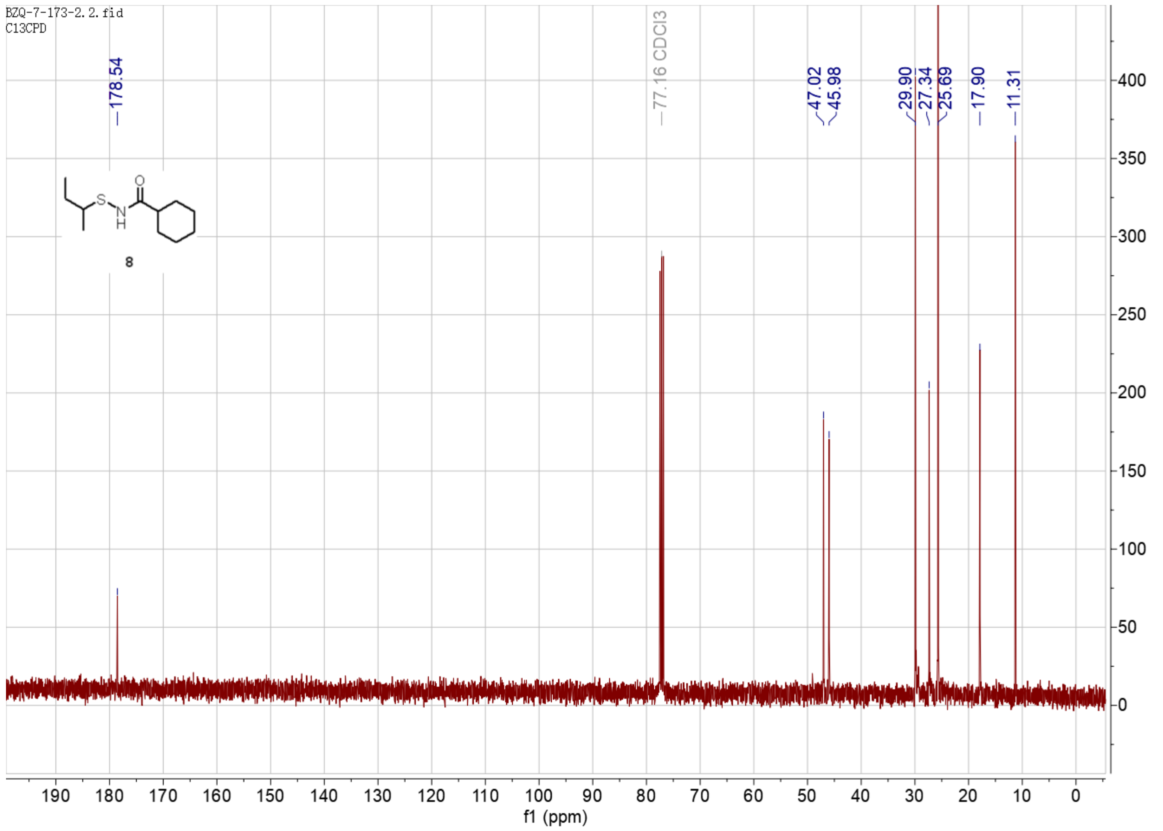

Supplementary Figure 119. ^{13}C NMR (101 MHz, $CDCl_3$, 293 K) spectrum of **8**.

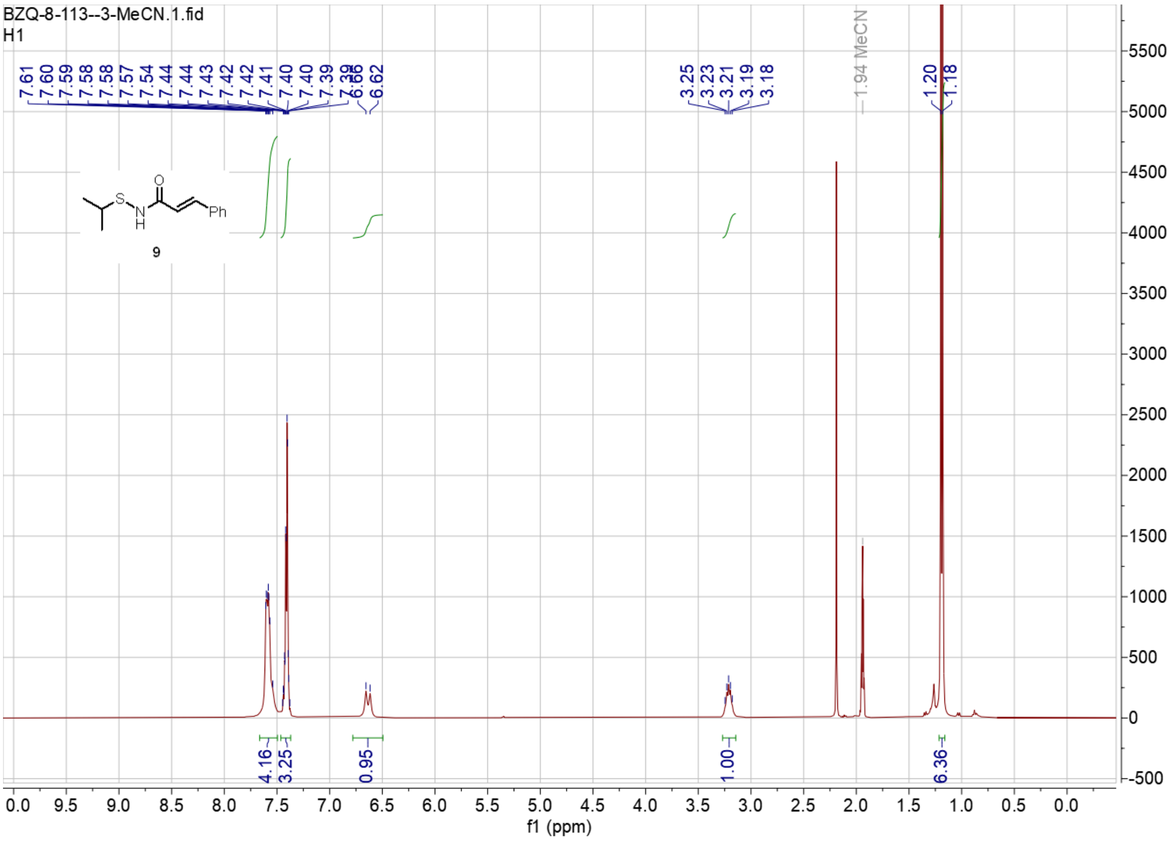

Supplementary Figure 120. ^{1}H NMR (400 MHz, $CDCl_3$, 293 K) spectrum of **9**.

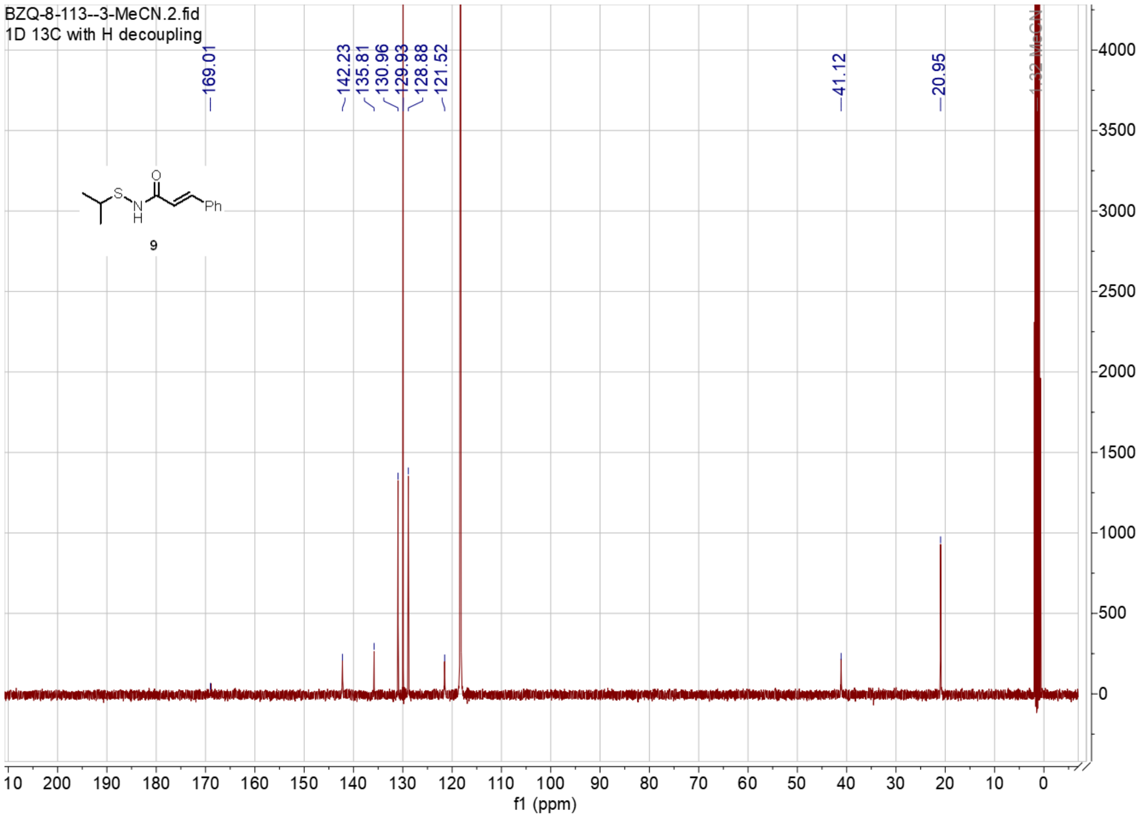

Supplementary Figure 121. ^{13}C NMR (101 MHz, $CDCl_3$, 293 K) spectrum of **9**.

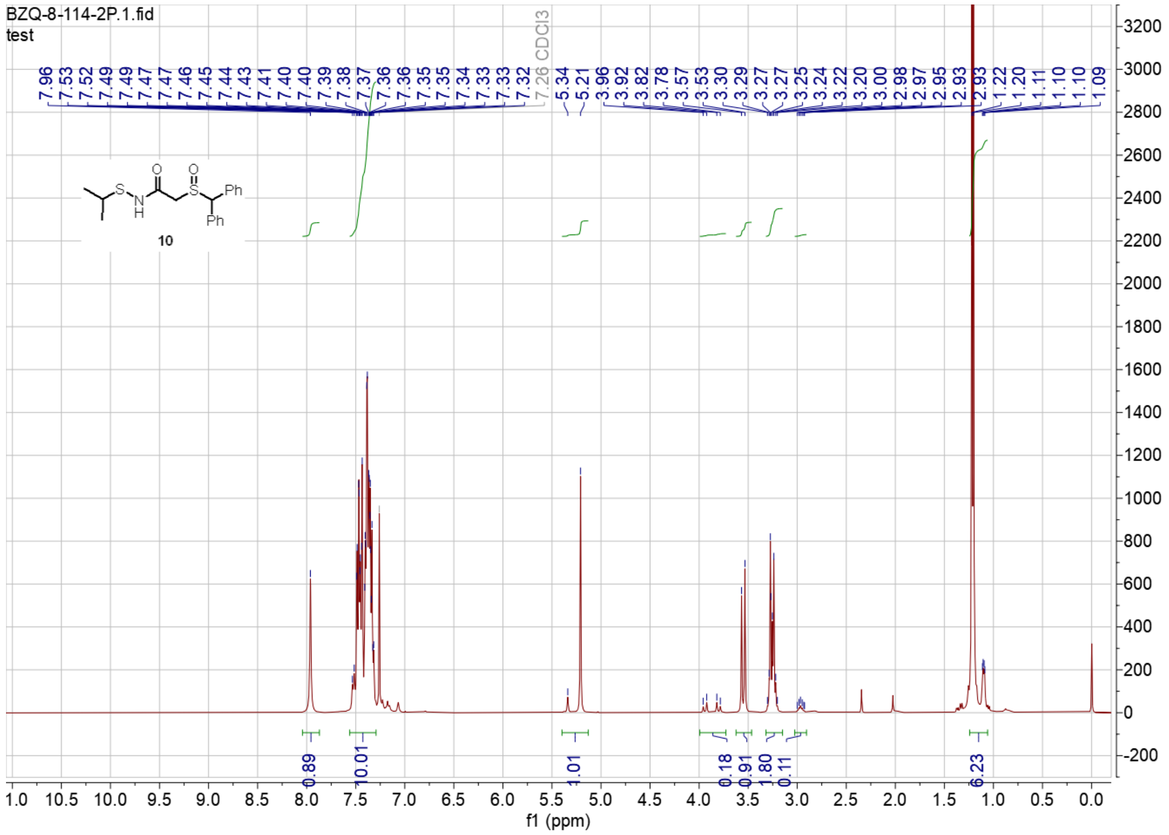

Supplementary Figure 122. ^{1}H NMR (400 MHz, $CDCl_3$, 293 K) spectrum of **10**.

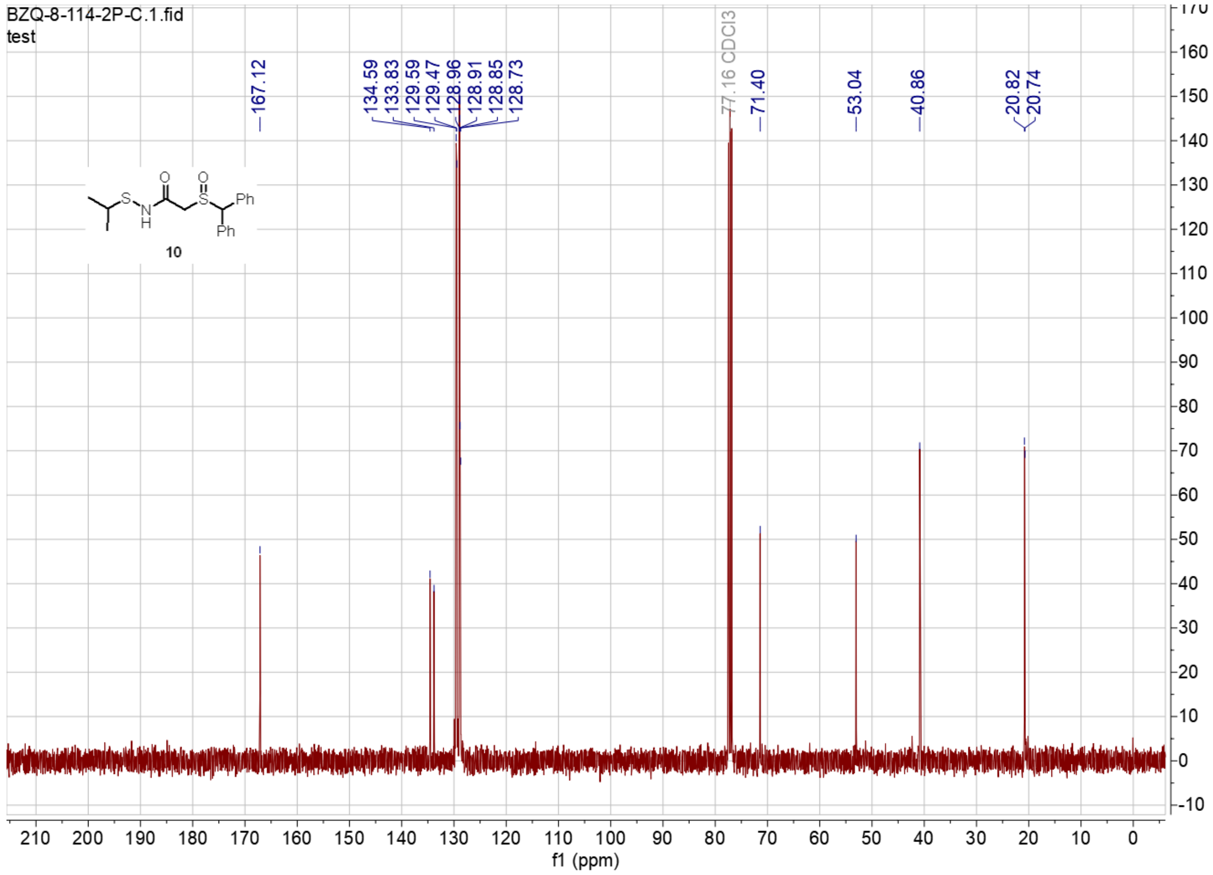

Supplementary Figure 123. ^{13}C NMR (101 MHz, $CDCl_3$, 293 K) spectrum of **10**.

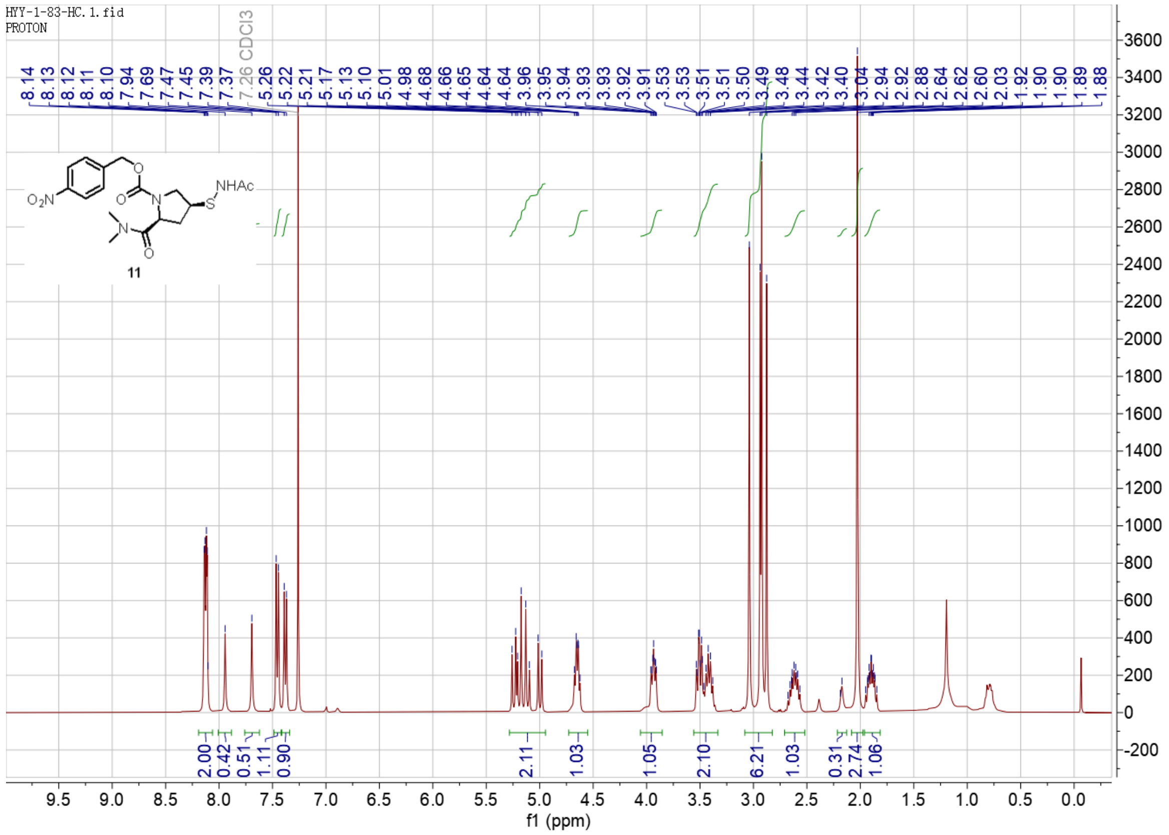

Supplementary Figure 124. ^{1}H NMR (400 MHz, $CDCl_3$, 293 K) spectrum of **11**.

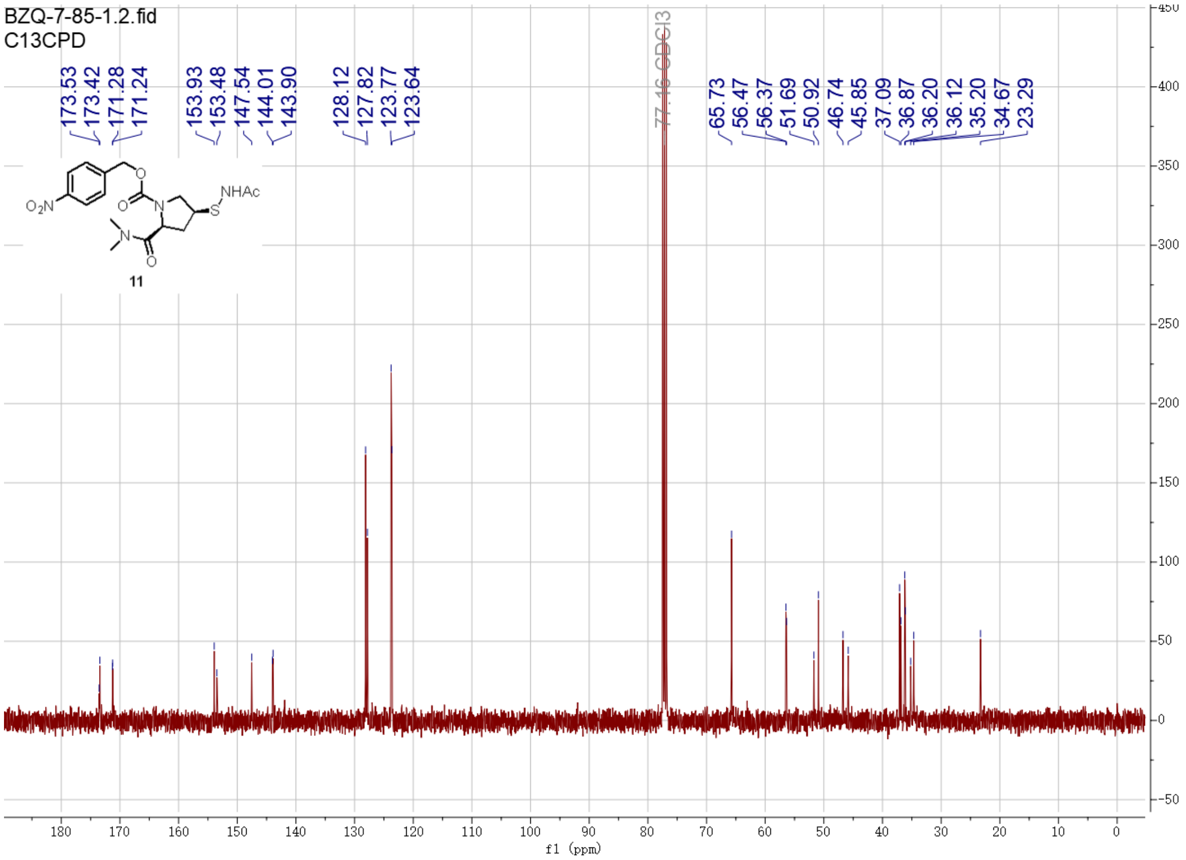

Supplementary Figure 125. ^{13}C NMR (101 MHz, $CDCl_3$, 293 K) spectrum of **11**.

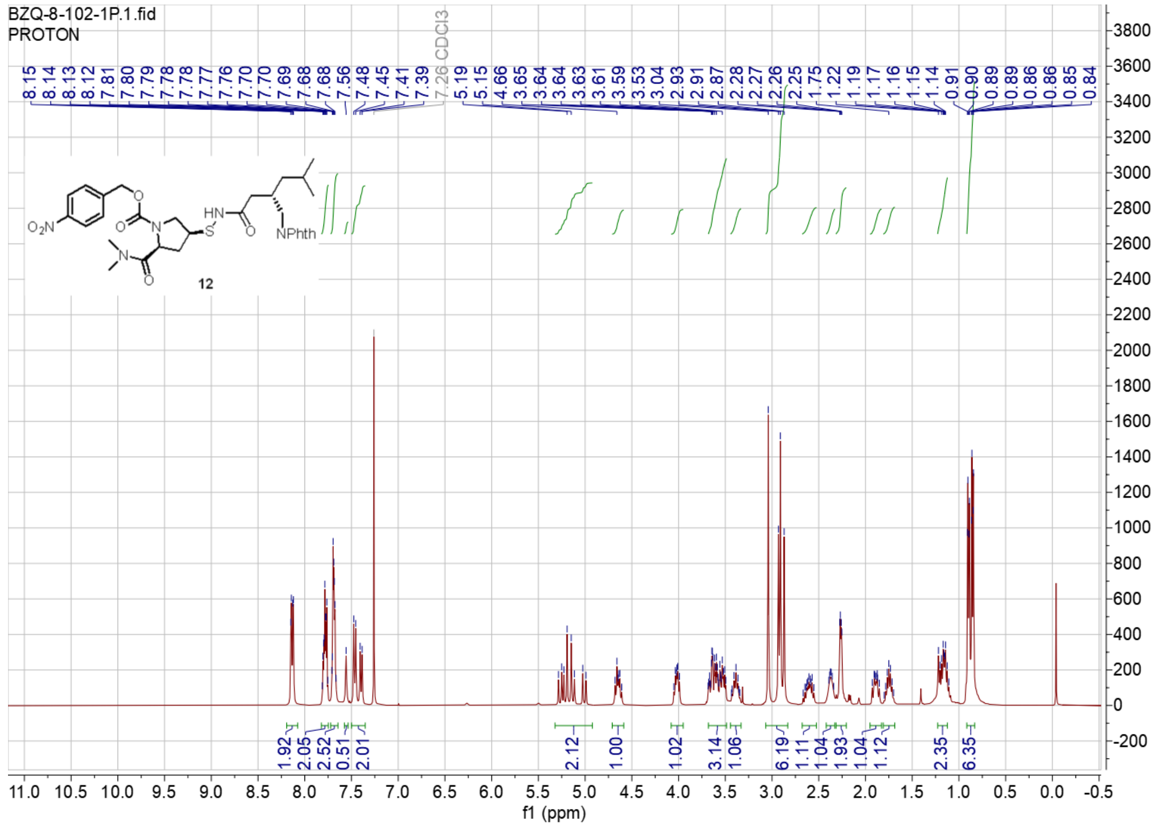

Supplementary Figure 126. ^{1}H NMR (400 MHz, $CDCl_3$, 293 K) spectrum of **12**.

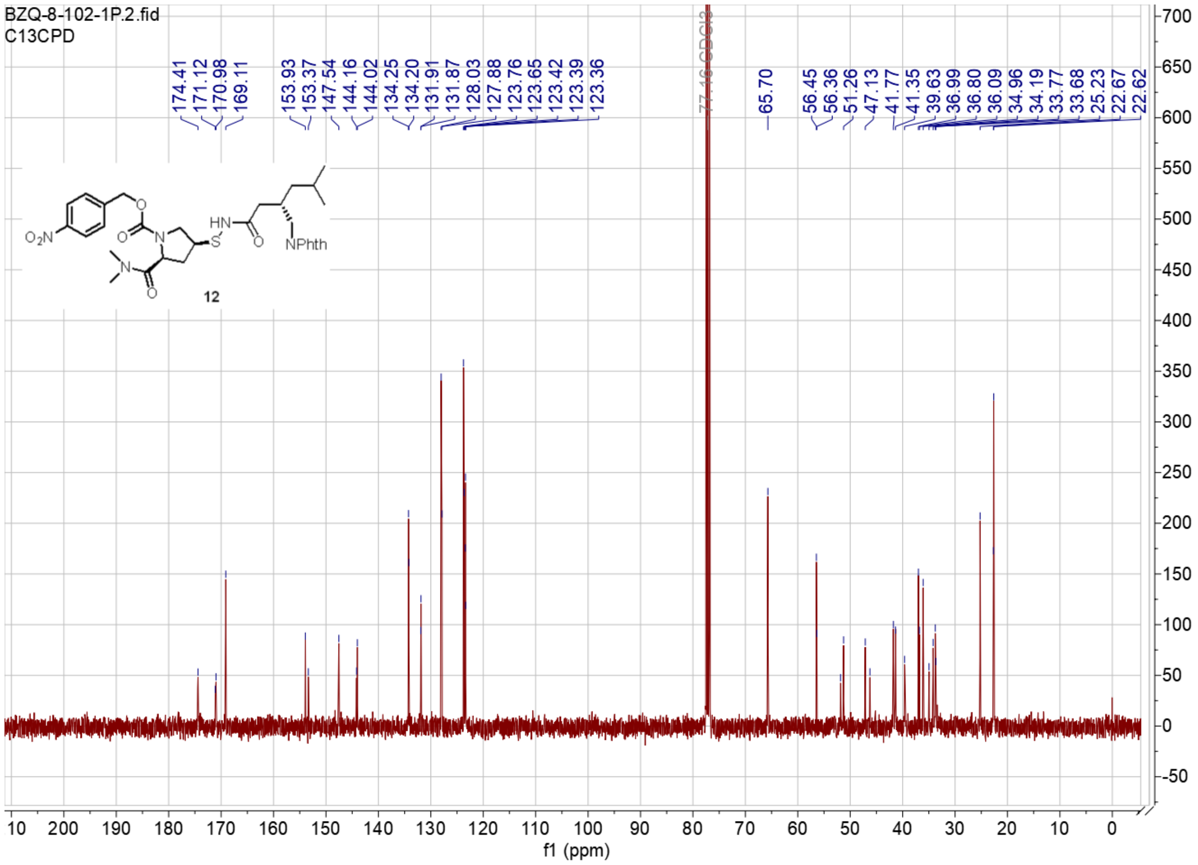

Supplementary Figure 127. ^{13}C NMR (101 MHz, $CDCl_3$, 293 K) spectrum of **12**.

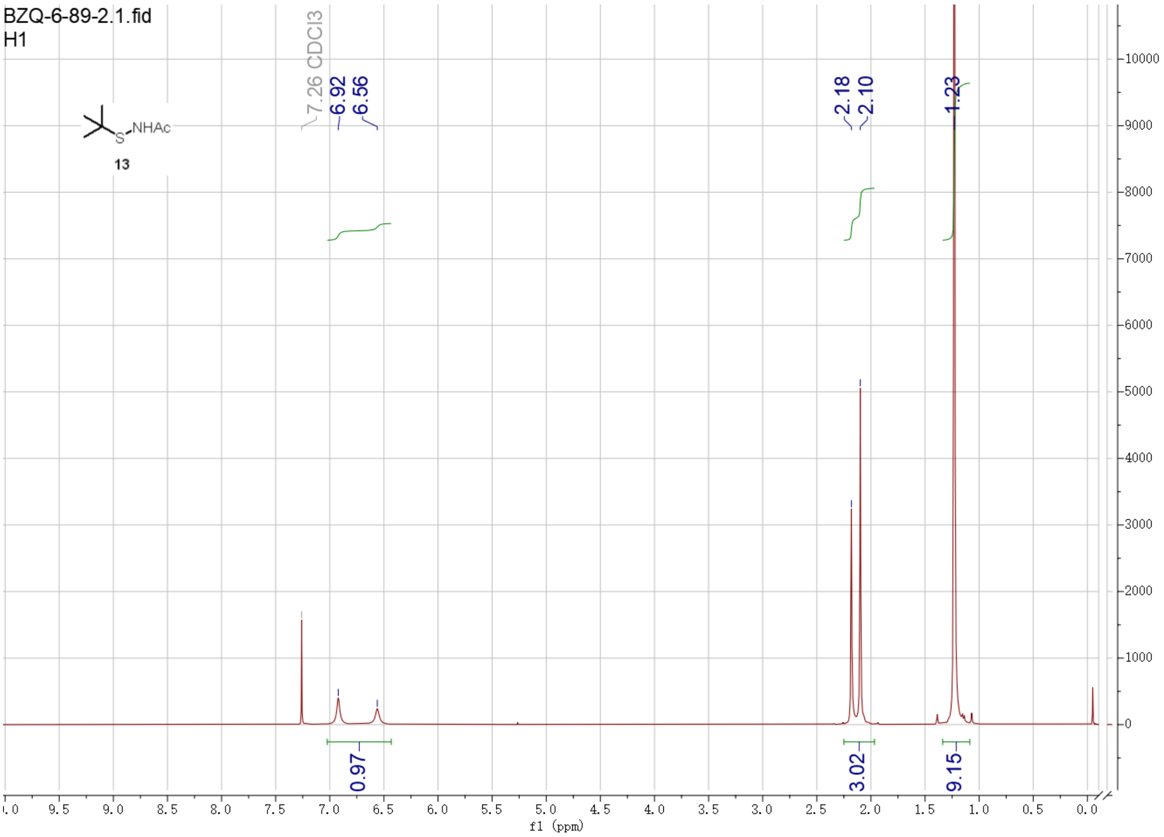

Supplementary Figure 128. ^{1}H NMR (400 MHz, $CDCl_3$, 293 K) spectrum of **13**.

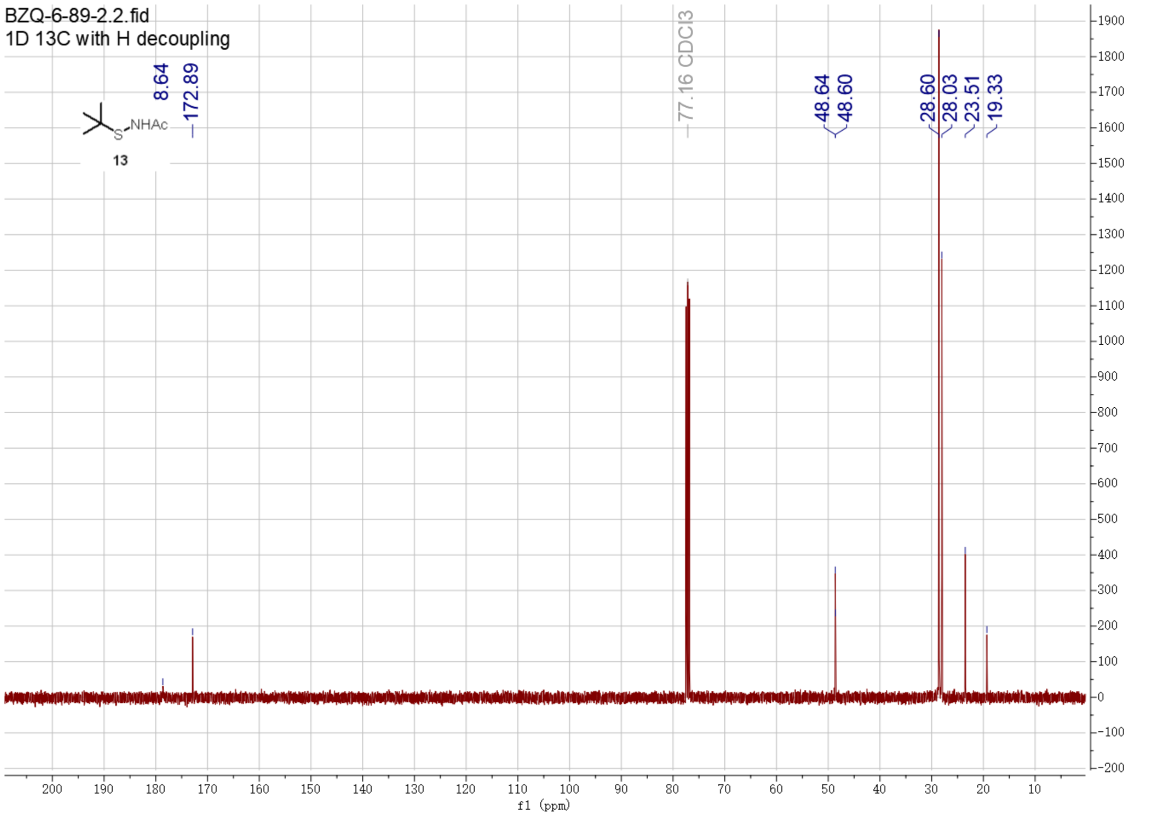

Supplementary Figure 129. ^{13}C NMR (101 MHz, $CDCl_3$, 293 K) spectrum of **13**.

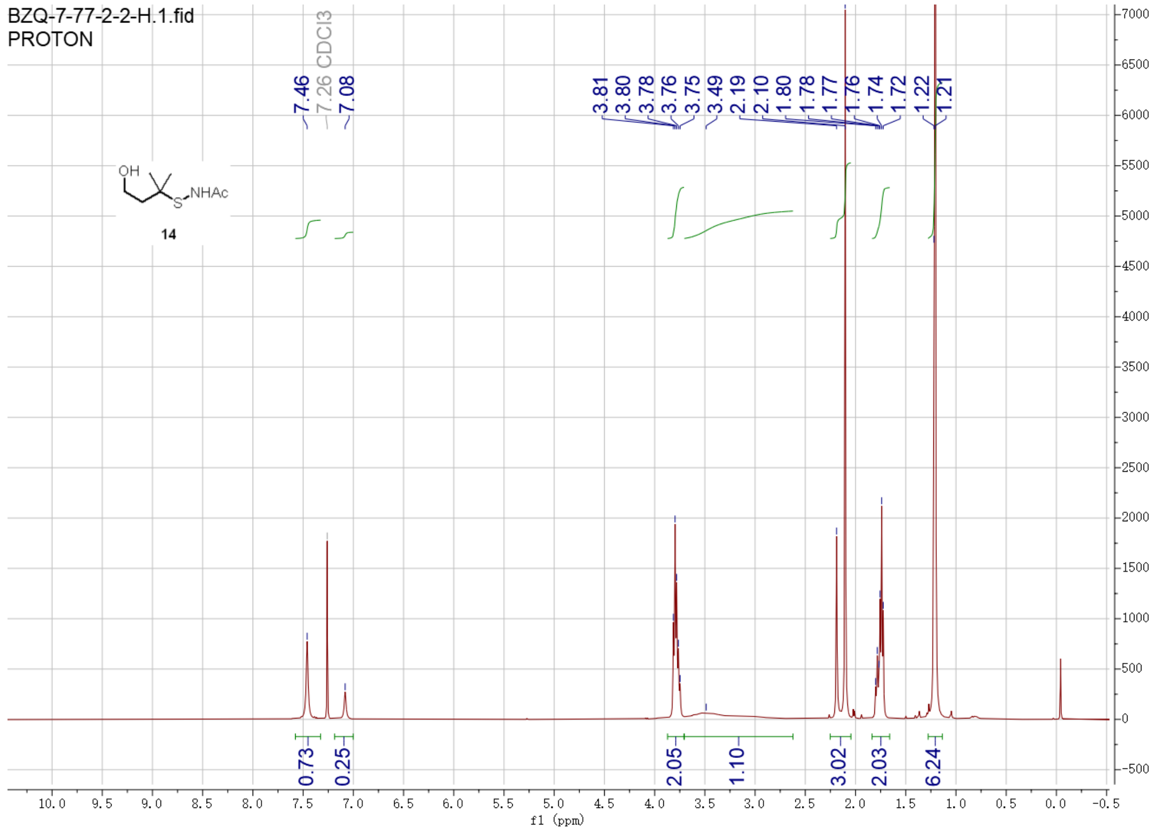

Supplementary Figure 130. ^{1}H NMR (400 MHz, $CDCl_3$, 293 K) spectrum of **14**.

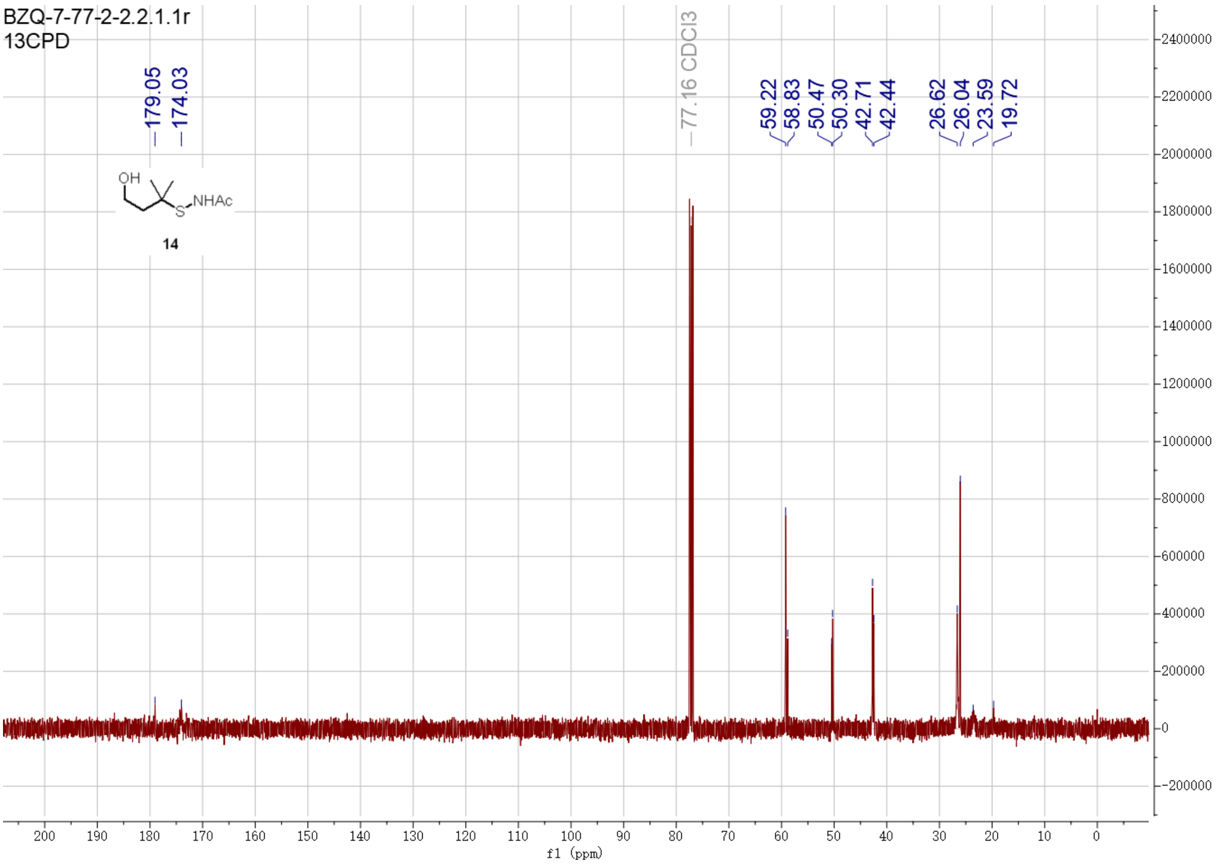

Supplementary Figure 131. ^{13}C NMR (101 MHz, $CDCl_3$, 293 K) spectrum of **14**.

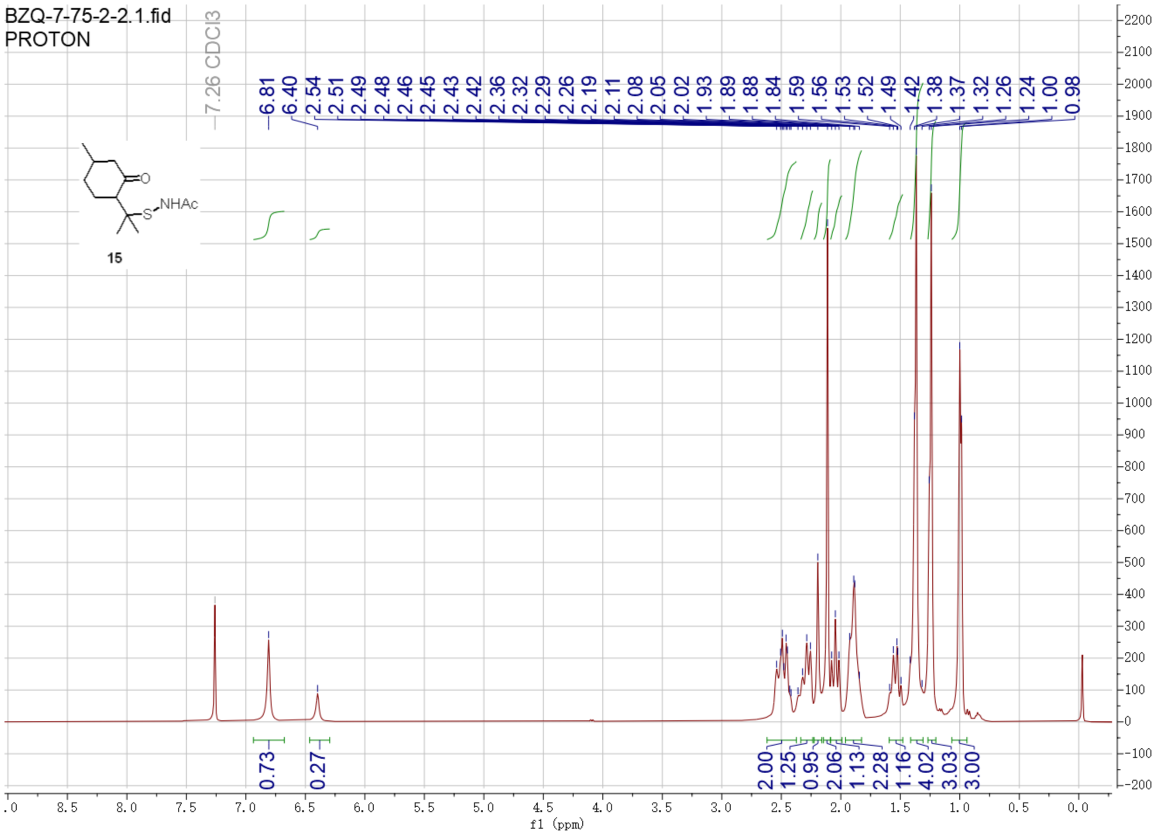

Supplementary Figure 132. ^{1}H NMR (400 MHz, $CDCl_3$, 293 K) spectrum of **15**.

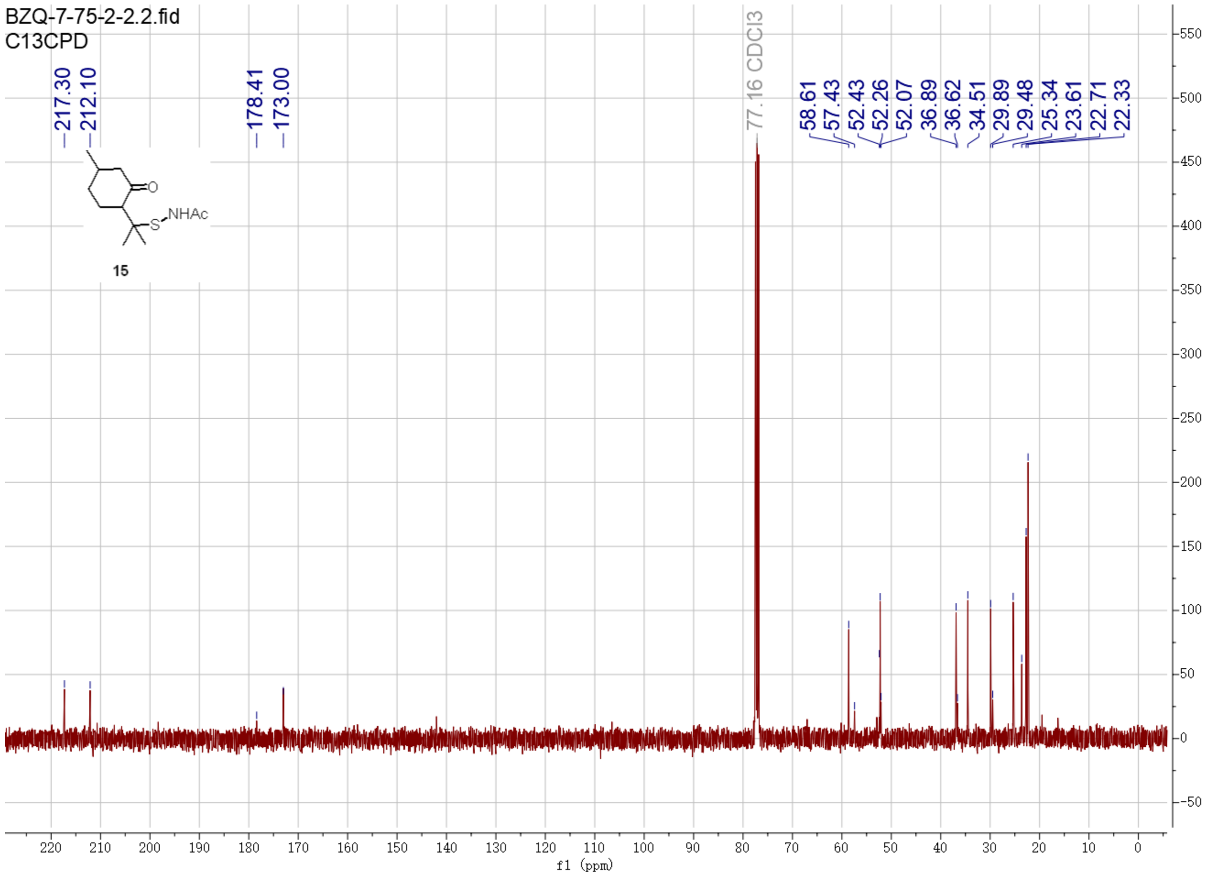

Supplementary Figure 133. ^{13}C NMR (101 MHz, $CDCl_3$, 293 K) spectrum of **15**.

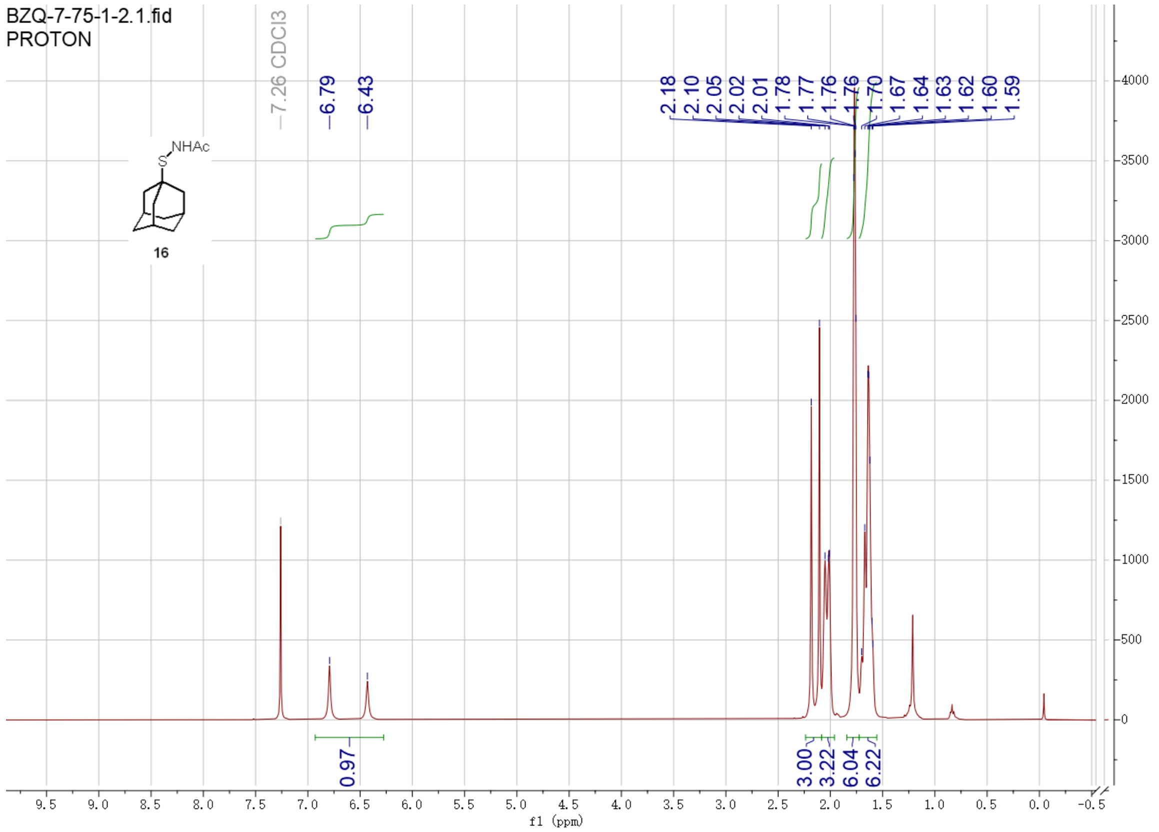

Supplementary Figure 134. ^{1}H NMR (400 MHz, $CDCl_3$, 293 K) spectrum of **16**.

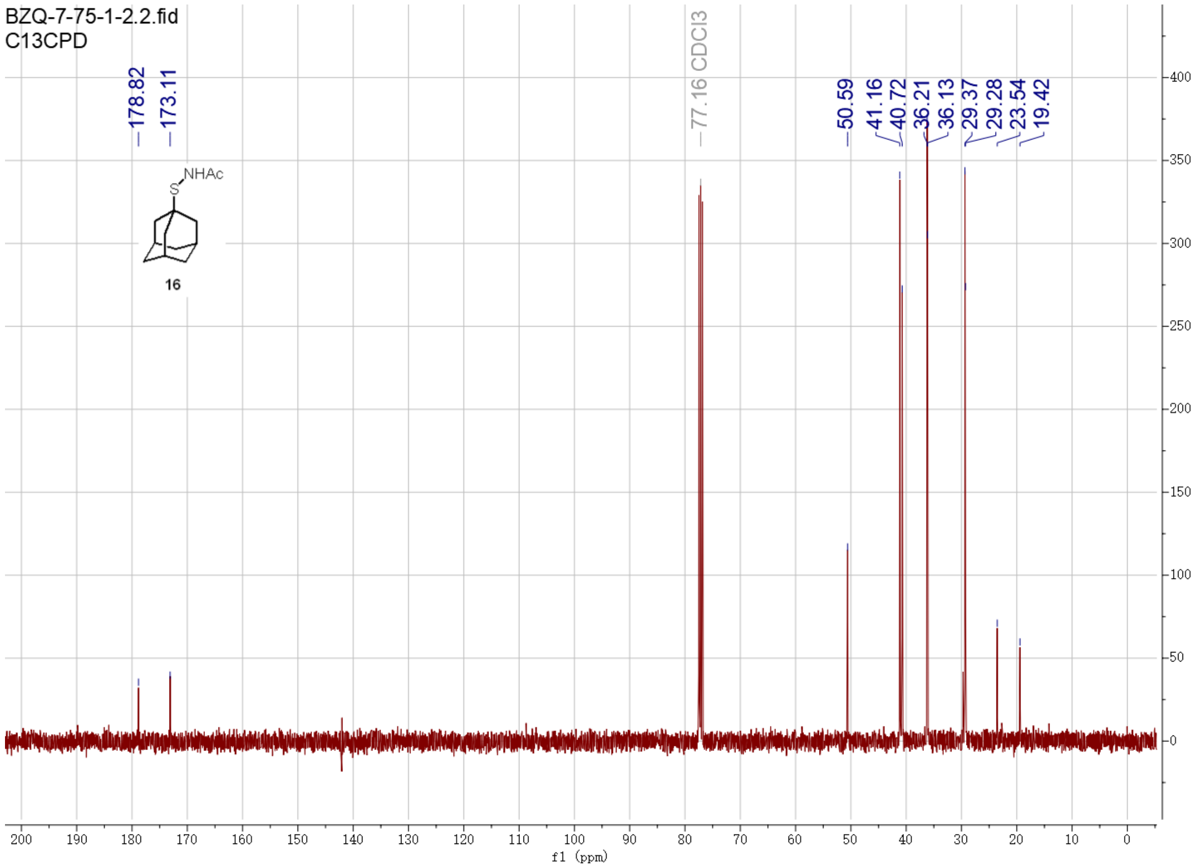

Supplementary Figure 135. ^{13}C NMR (101 MHz, $CDCl_3$, 293 K) spectrum of **16**.

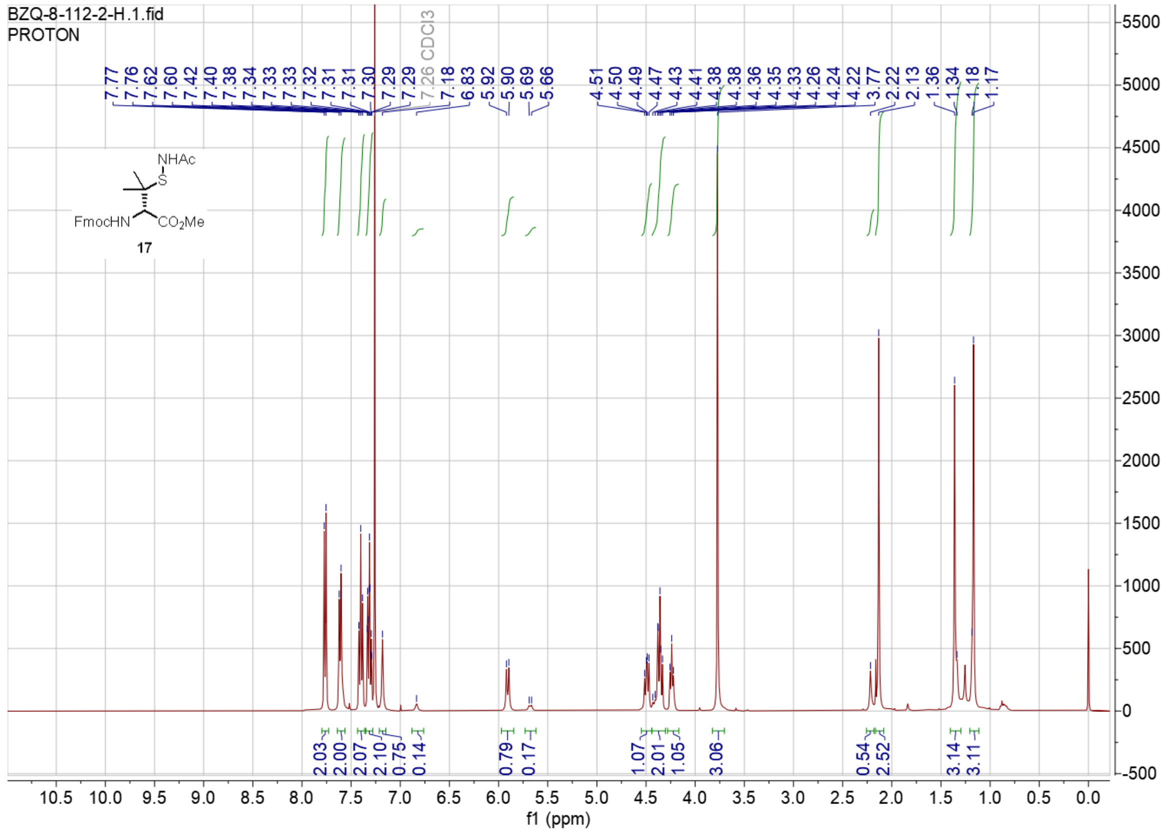

Supplementary Figure 136. ^{1}H NMR (400 MHz, $CDCl_3$, 293 K) spectrum of **17**.

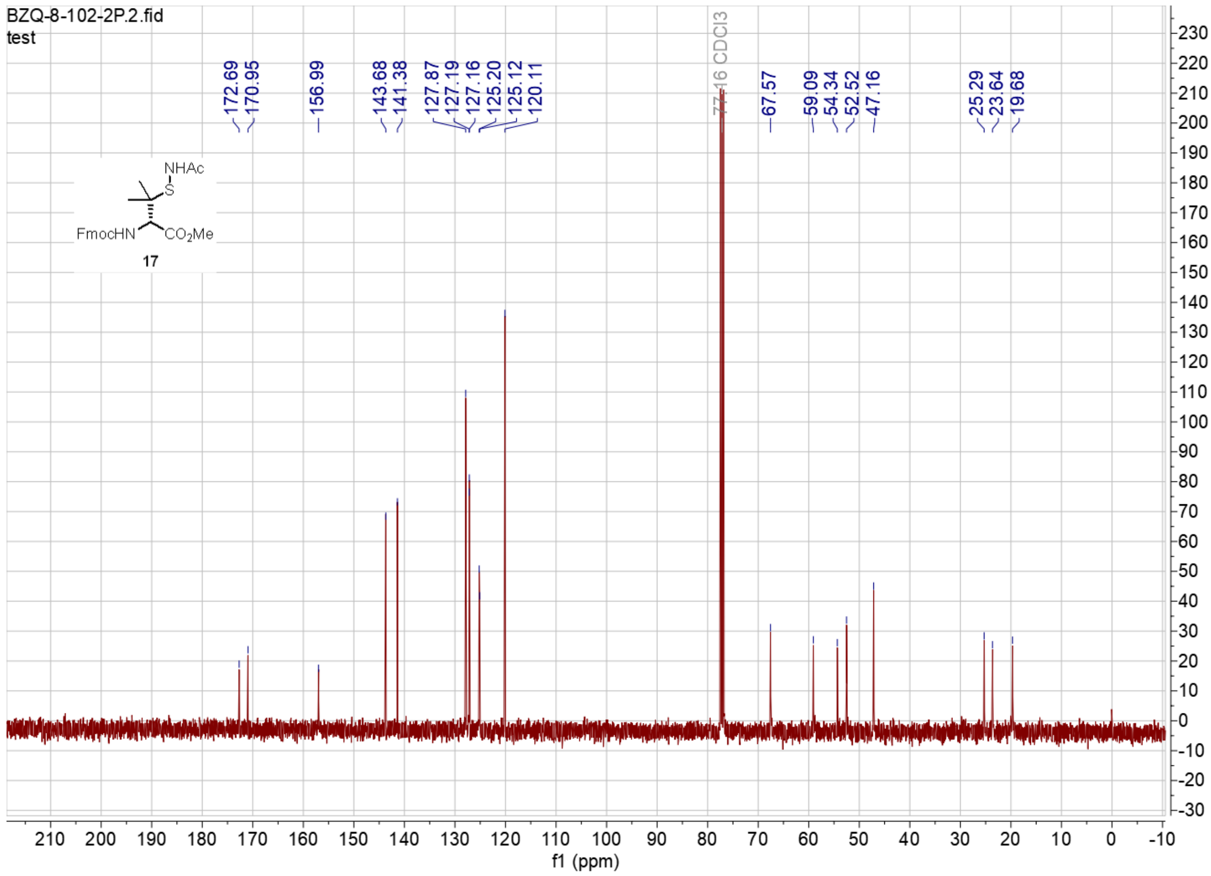

Supplementary Figure 137. ^{13}C NMR (101 MHz, $CDCl_3$, 293 K) spectrum of **17**.

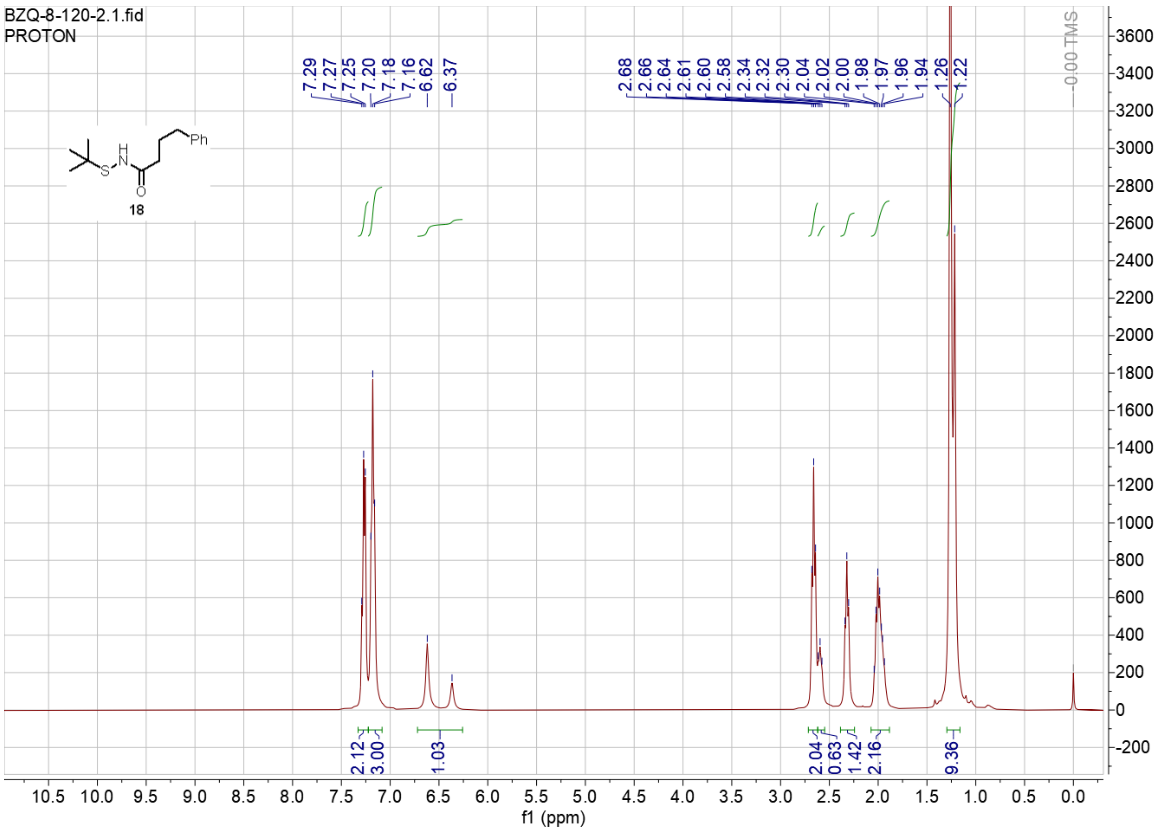

Supplementary Figure 138. ^{1}H NMR (400 MHz, $CDCl_3$, 293 K) spectrum of **18**.

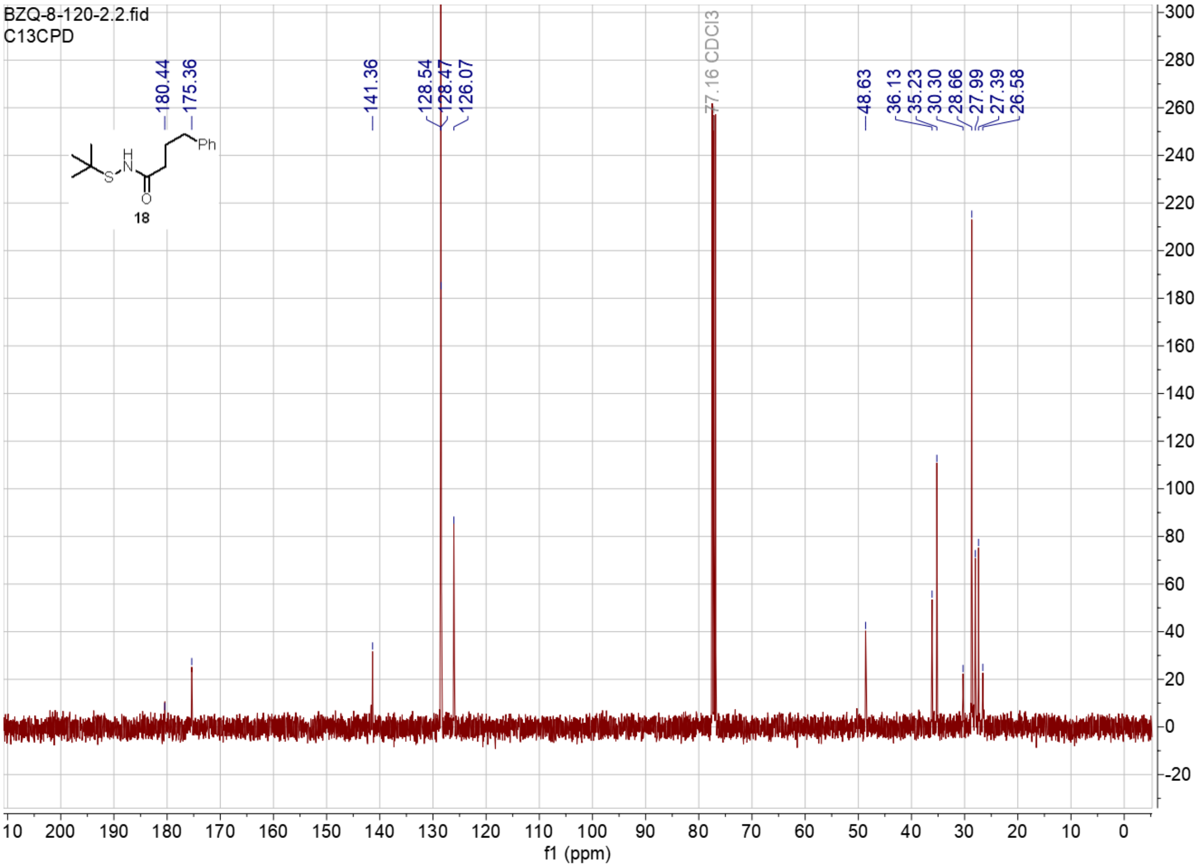

Supplementary Figure 139. ^{13}C NMR (101 MHz, $CDCl_3$, 293 K) spectrum of **18**.

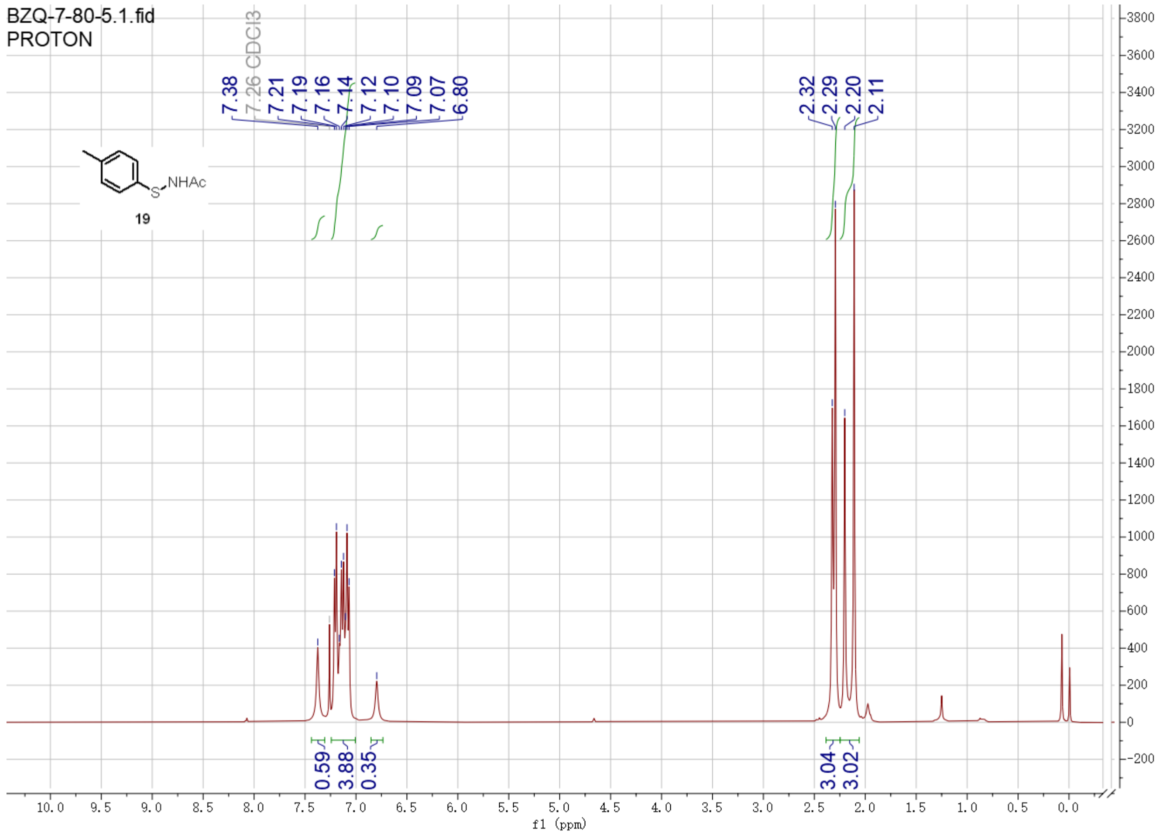

Supplementary Figure 140. ^{1}H NMR (400 MHz, $CDCl_3$, 293 K) spectrum of **19**.

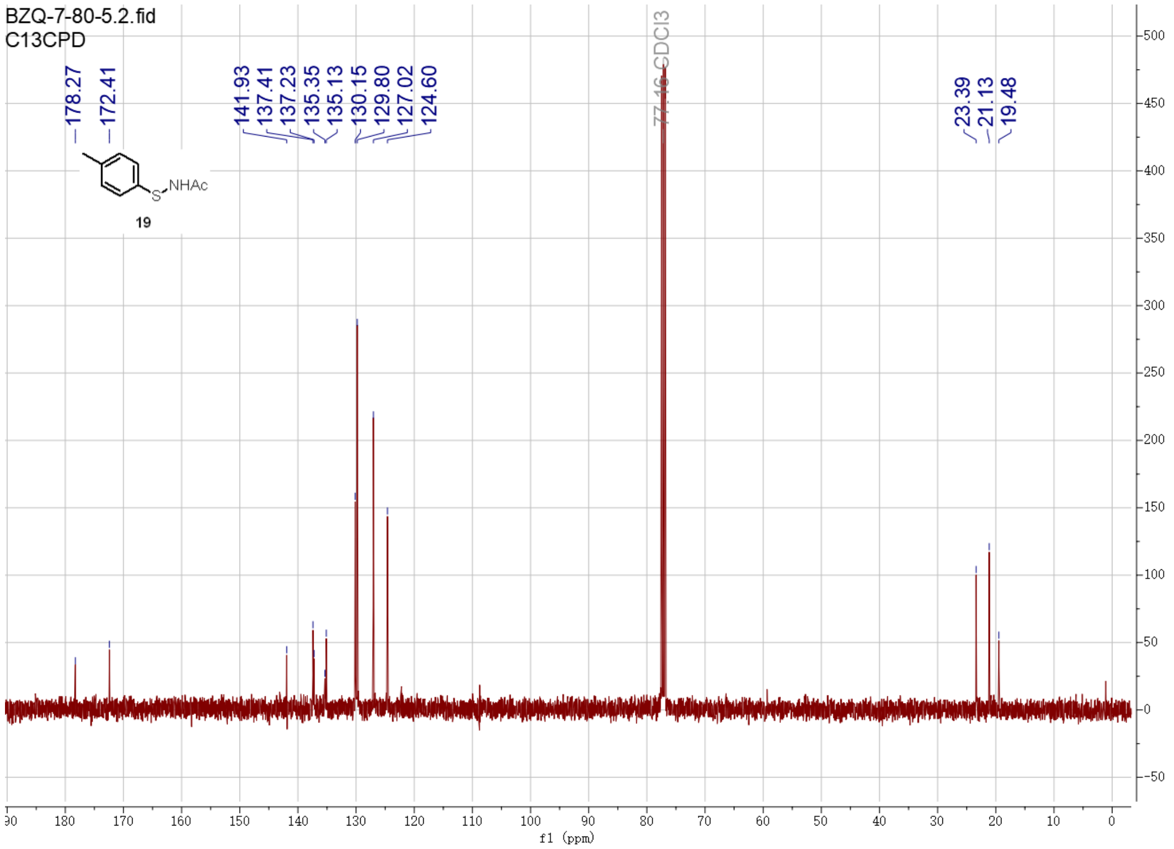

Supplementary Figure 141. ^{13}C NMR (101 MHz, $CDCl_3$, 293 K) spectrum of **19**.

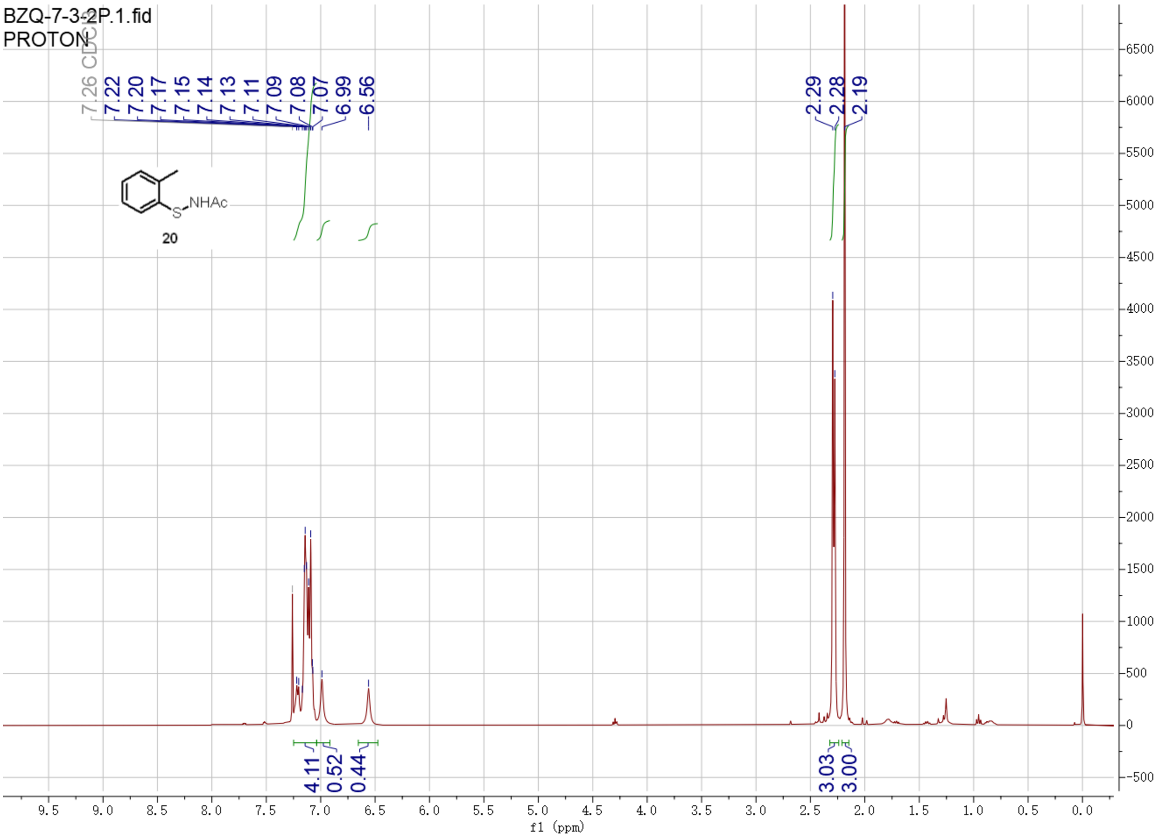

Supplementary Figure 142. ^{1}H NMR (400 MHz, $CDCl_3$, 293 K) spectrum of **20**.

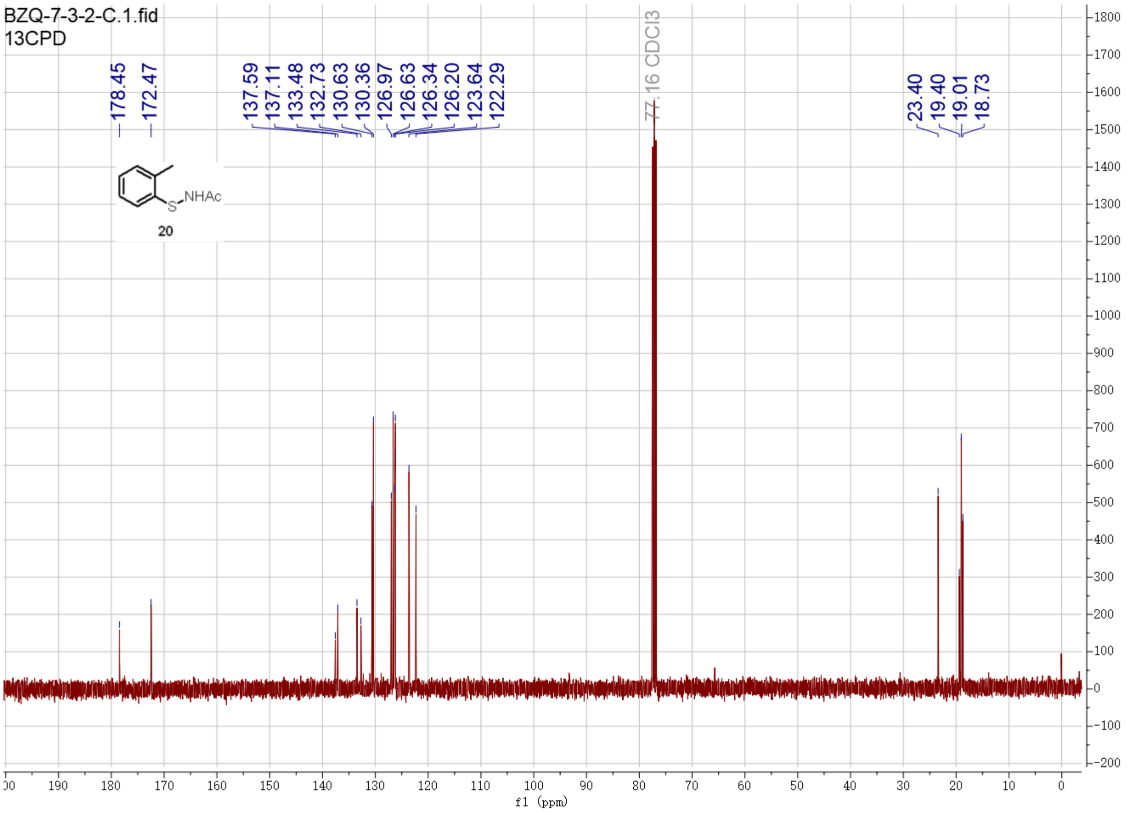

Supplementary Figure 143. ^{13}C NMR (101 MHz, $CDCl_3$, 293 K) spectrum of **20**.

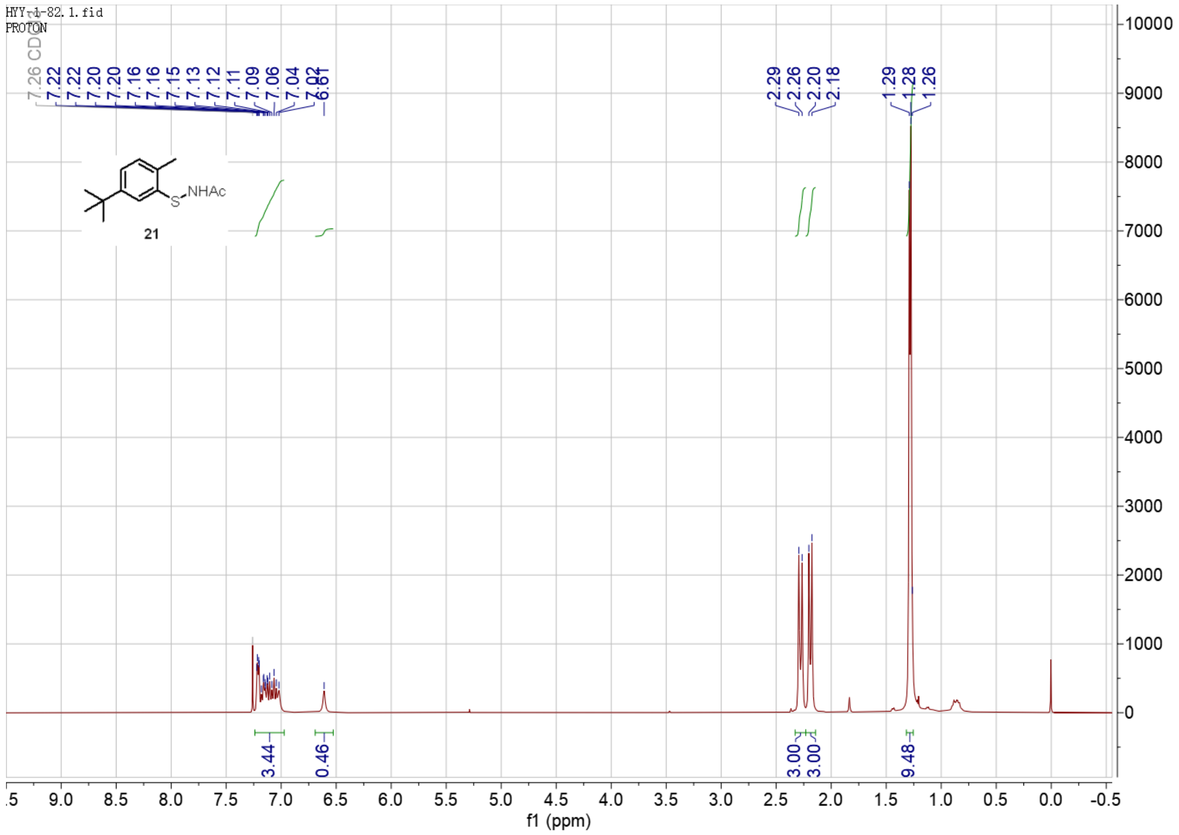

Supplementary Figure 144. ^{1}H NMR (400 MHz, $CDCl_3$, 293 K) spectrum of **21**.

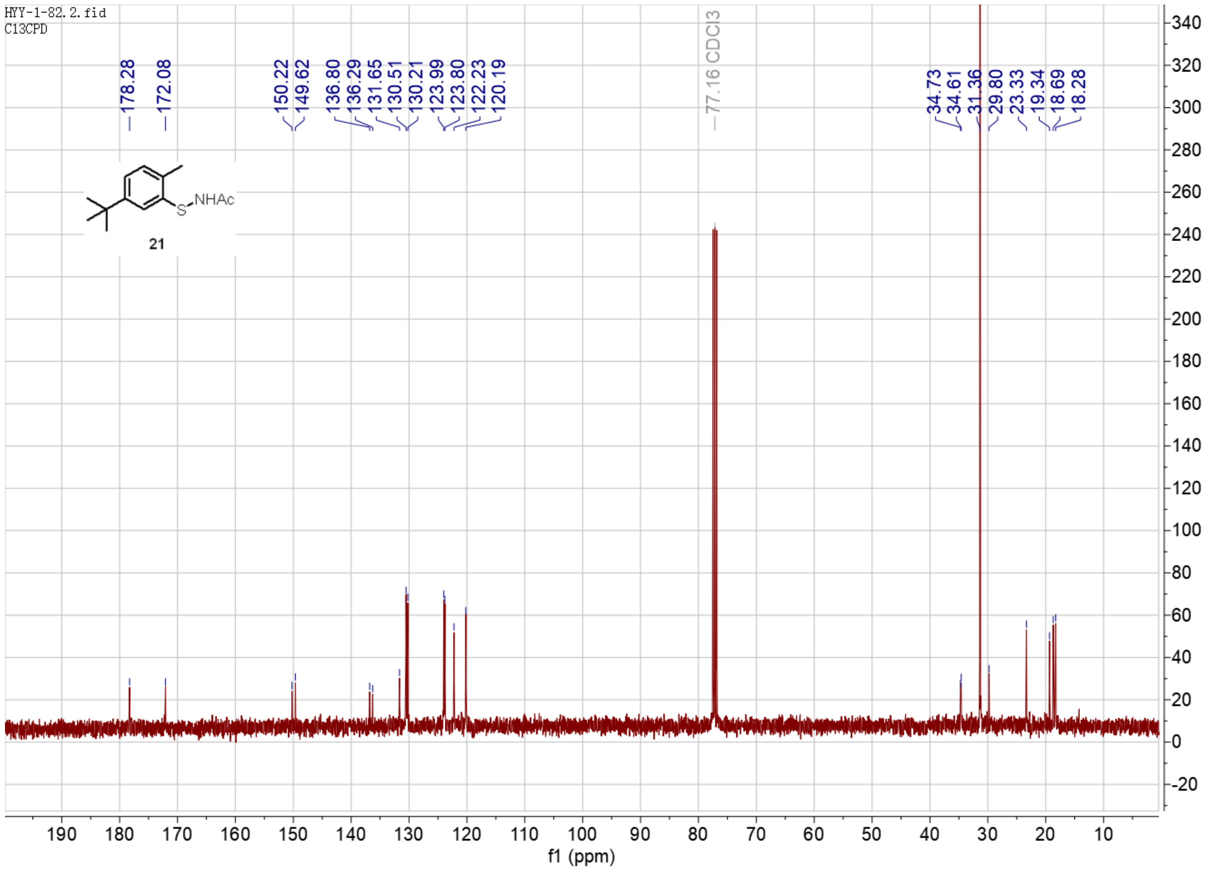

Supplementary Figure 145. ^{13}C NMR (101 MHz, $CDCl_3$, 293 K) spectrum of **21**.

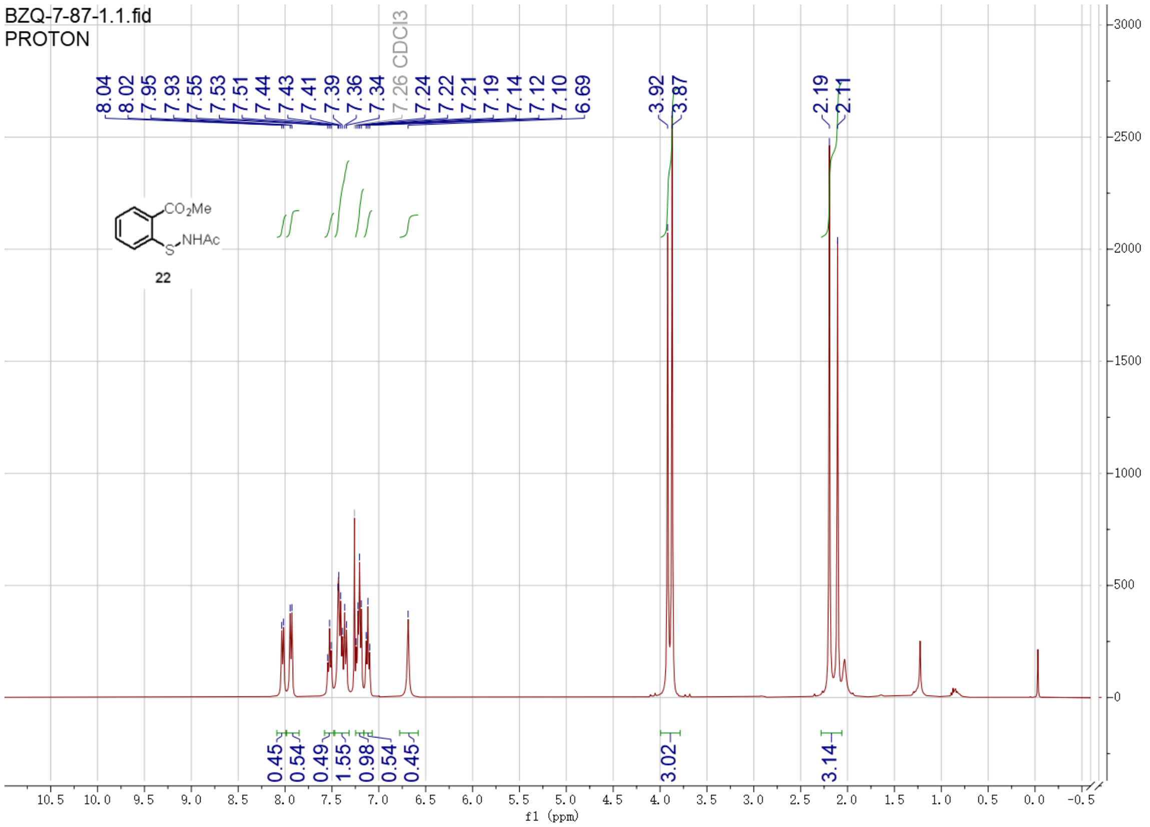

Supplementary Figure 146. ^{1}H NMR (400 MHz, $CDCl_3$, 293 K) spectrum of **22**.

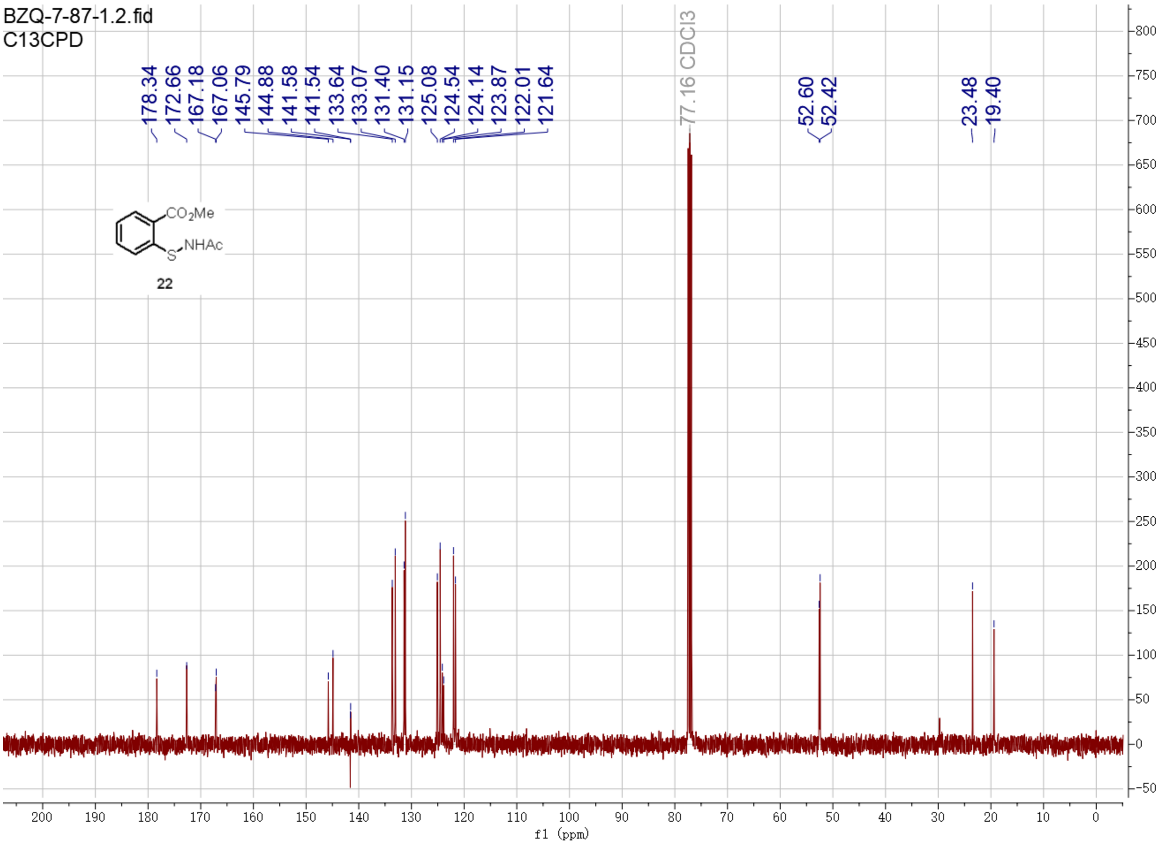

Supplementary Figure 147. ^{13}C NMR (101 MHz, $CDCl_3$, 293 K) spectrum of **22**.

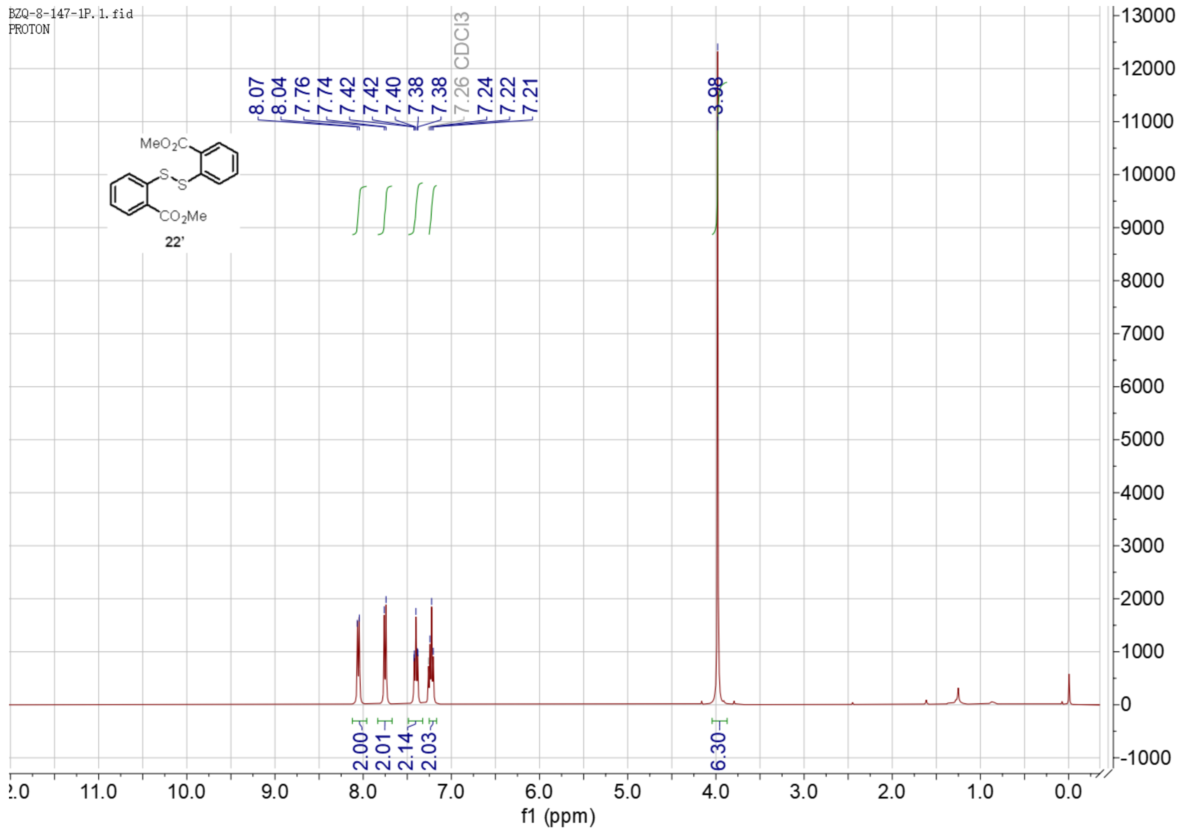

Supplementary Figure 148. ^{1}H NMR (400 MHz, $CDCl_3$, 293 K) spectrum of **22’**.

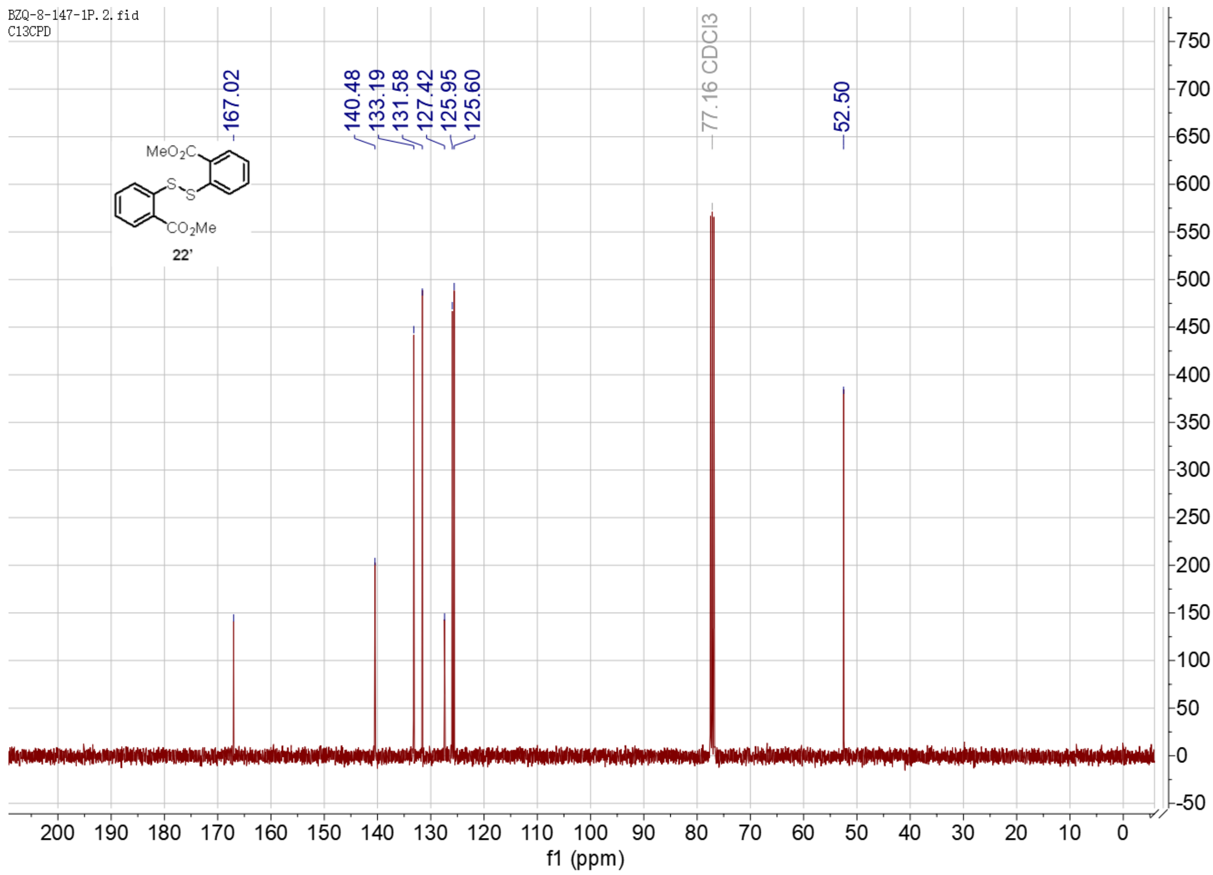

Supplementary Figure 149. ^{13}C NMR (101 MHz, $CDCl_3$, 293 K) spectrum of **22’**.

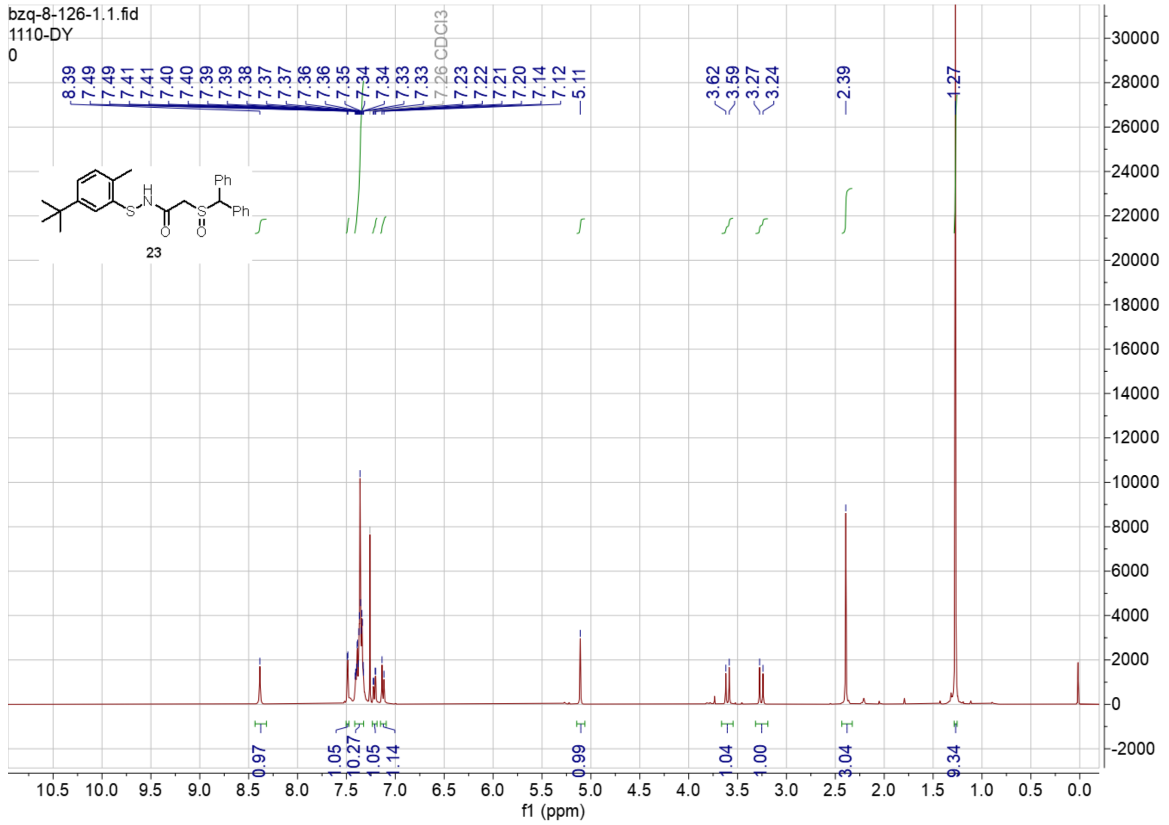

Supplementary Figure 150. ^{1}H NMR (400 MHz, $CDCl_3$, 293 K) spectrum of **23**.

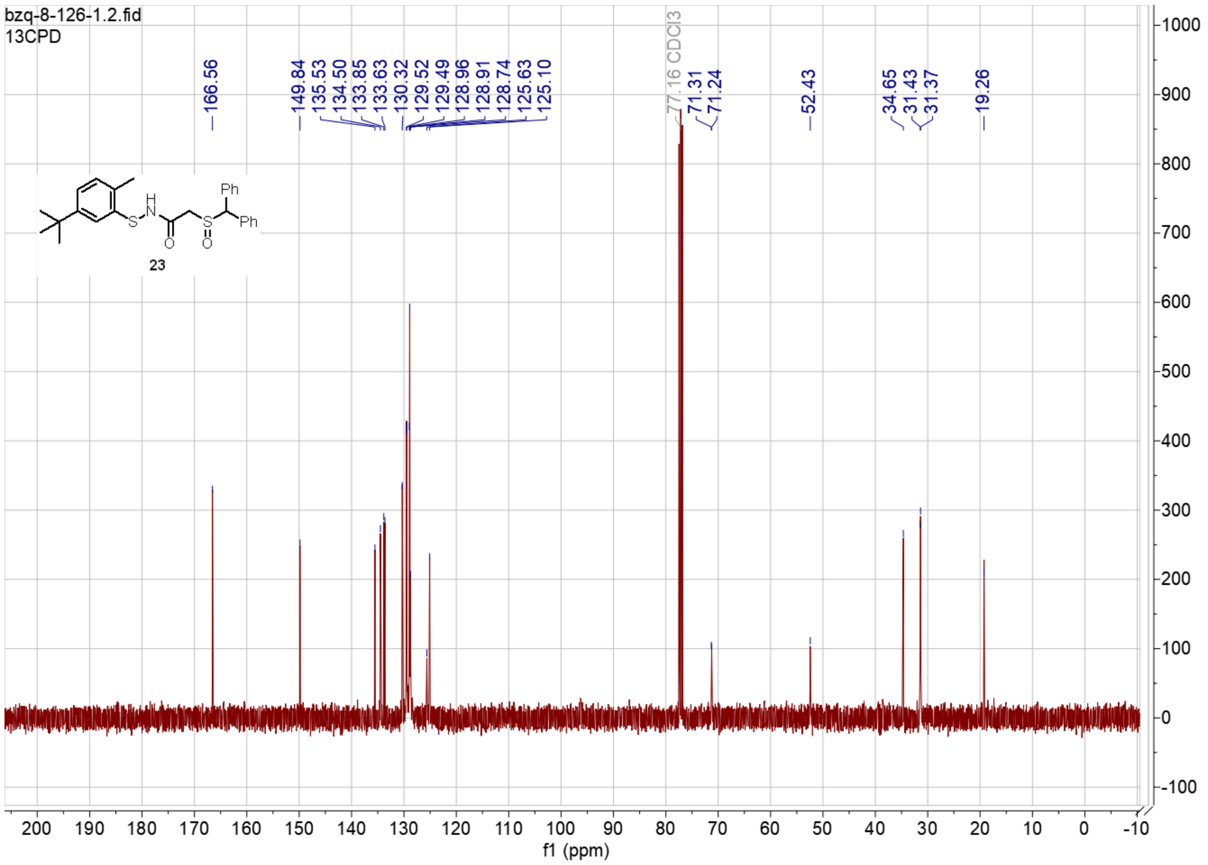

Supplementary Figure 151. ^{13}C NMR (101 MHz, $CDCl_3$, 293 K) spectrum of **23**.

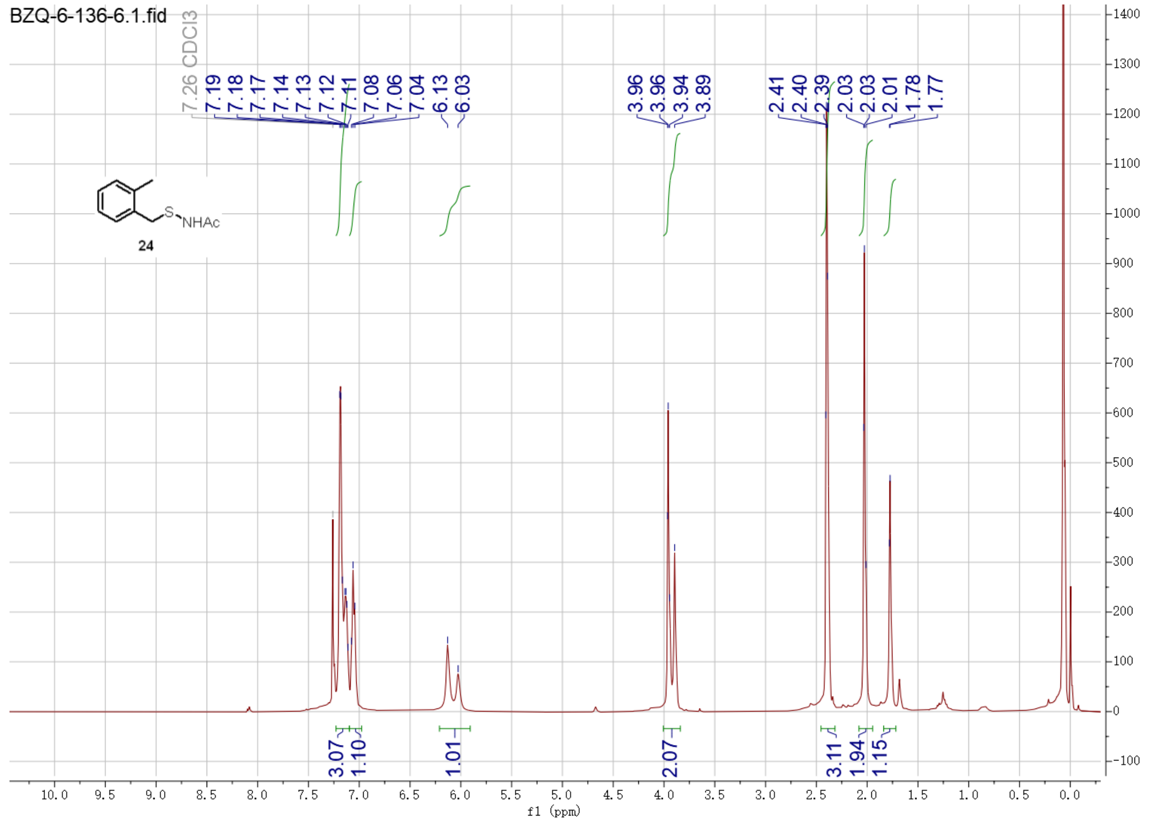

Supplementary Figure 152. ^{1}H NMR (400 MHz, $CDCl_3$, 293 K) spectrum of **24**.

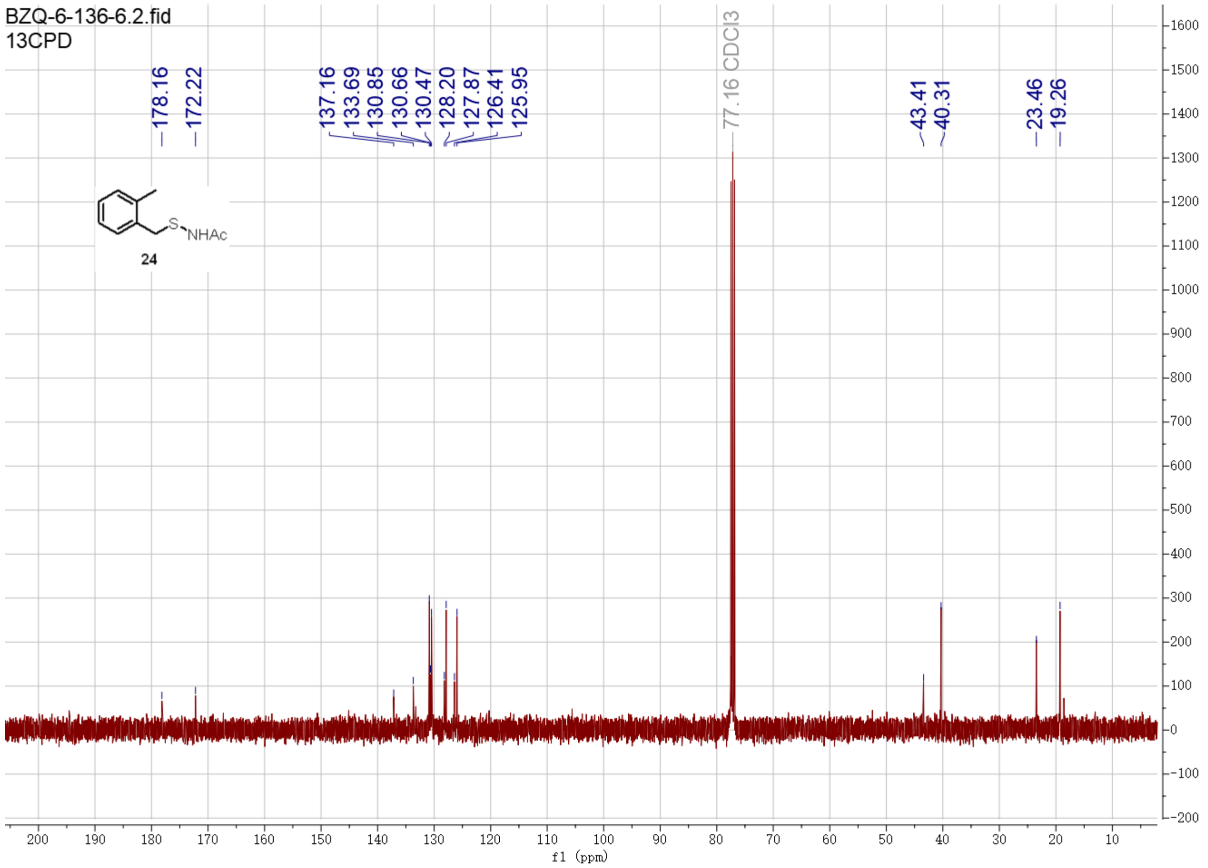

Supplementary Figure 153. ^{13}C NMR (101 MHz, $CDCl_3$, 293 K) spectrum of **24**.

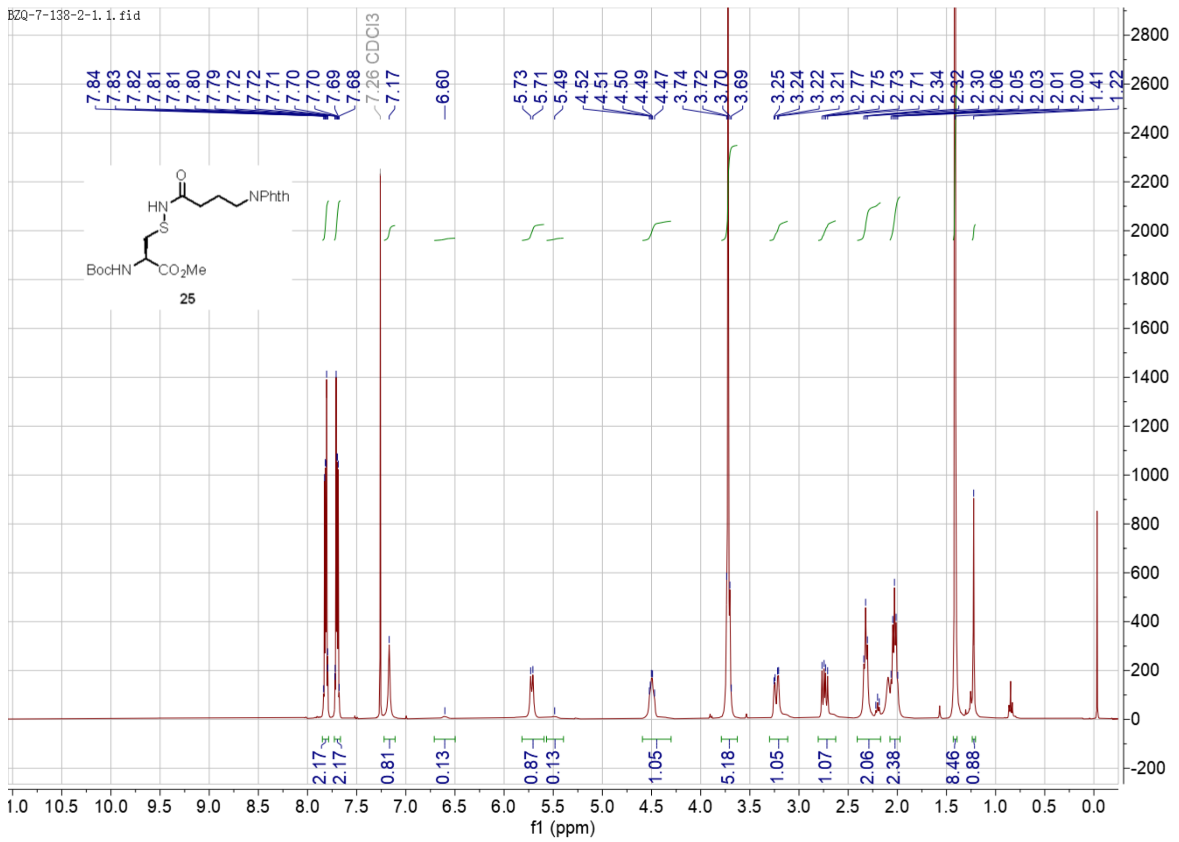

Supplementary Figure 154. ^{1}H NMR (400 MHz, $CDCl_3$, 293 K) spectrum of **25**.

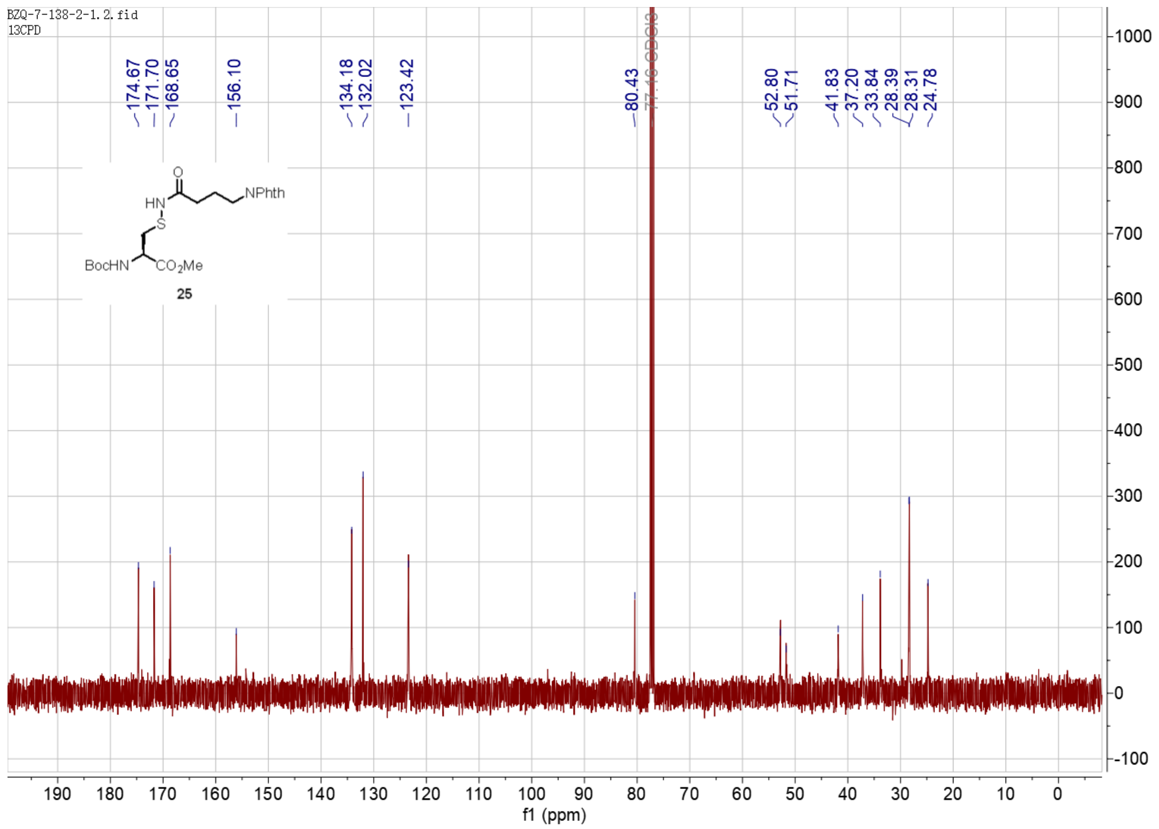

Supplementary Figure 155. ^{13}C NMR (101 MHz, $CDCl_3$, 293 K) spectrum of **25**.

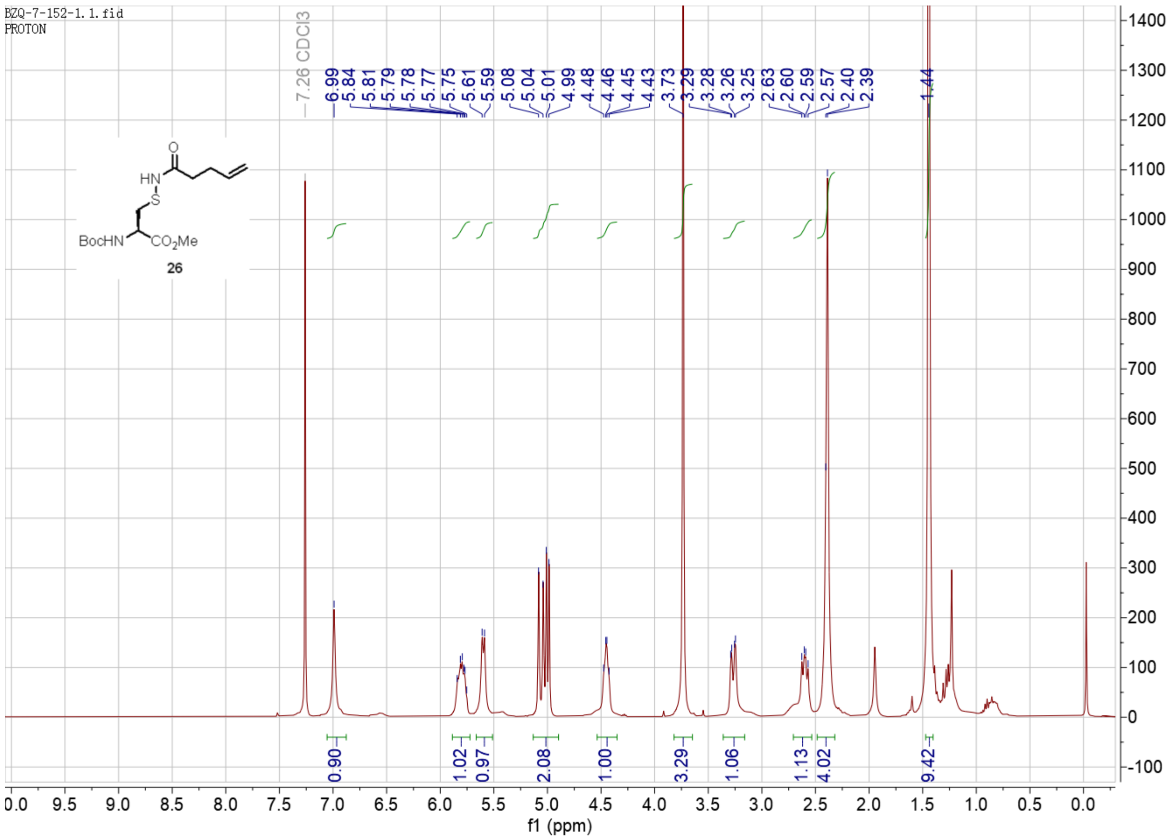

Supplementary Figure 156. ^{1}H NMR (400 MHz, $CDCl_3$, 293 K) spectrum of **26**.

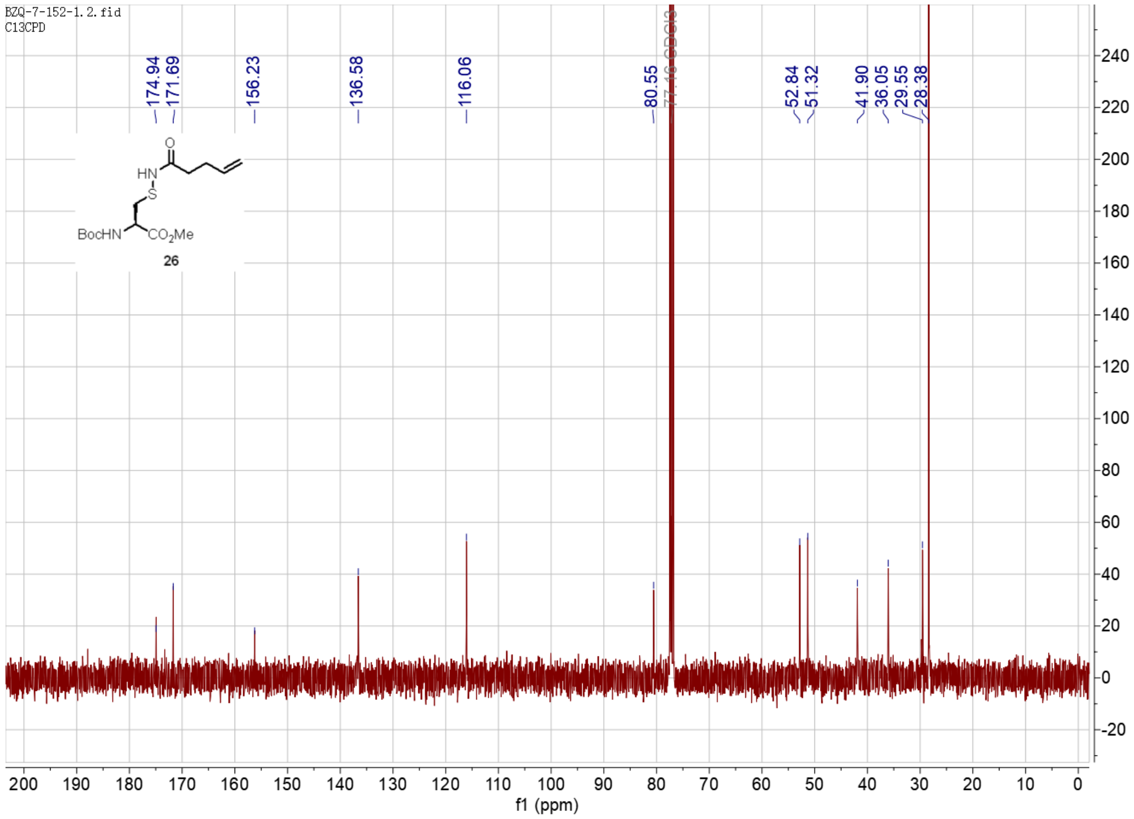

Supplementary Figure 157. ^{13}C NMR (101 MHz, $CDCl_3$, 293 K) spectrum of **26**.

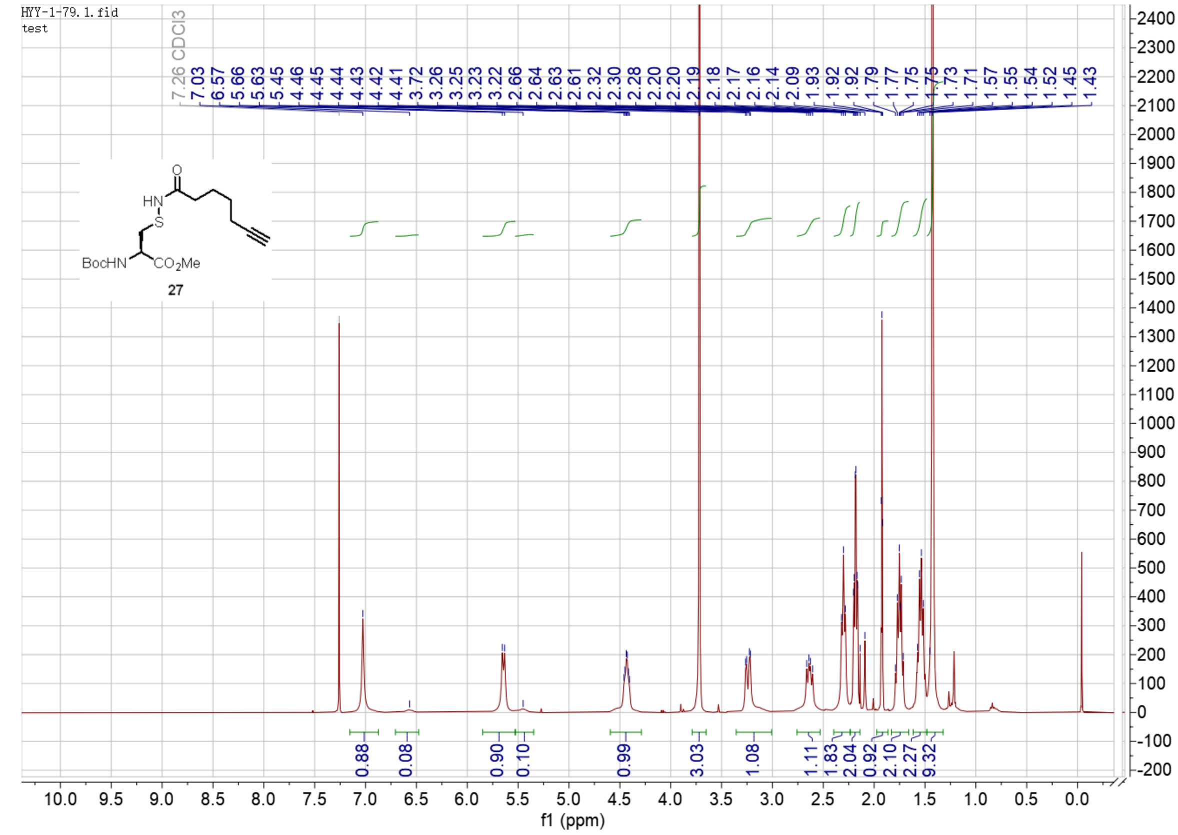

Supplementary Figure 158. ^{1}H NMR (400 MHz, $CDCl_3$, 293 K) spectrum of **27**.

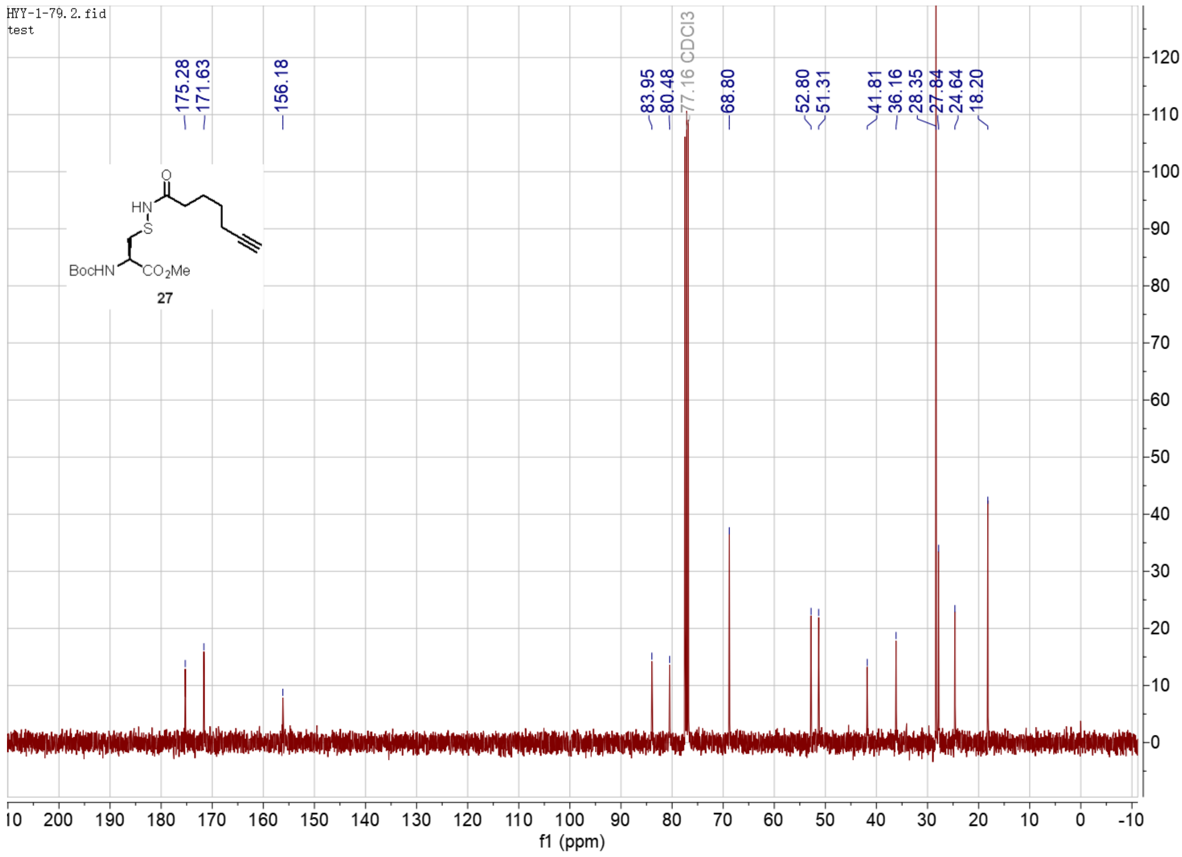

Supplementary Figure 159. ^{13}C NMR (101 MHz, $CDCl_3$, 293 K) spectrum of **27**.

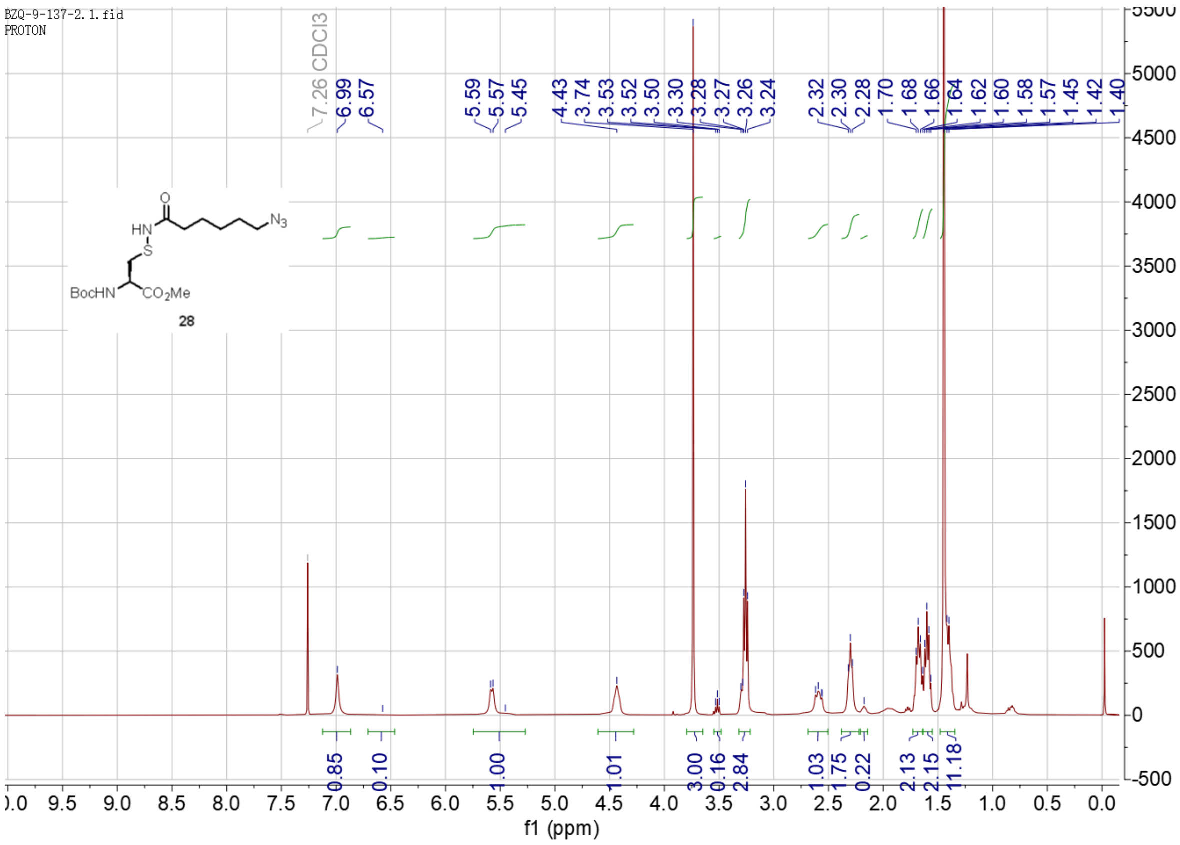

Supplementary Figure 160. ^{1}H NMR (400 MHz, $CDCl_3$, 293 K) spectrum of **28**.

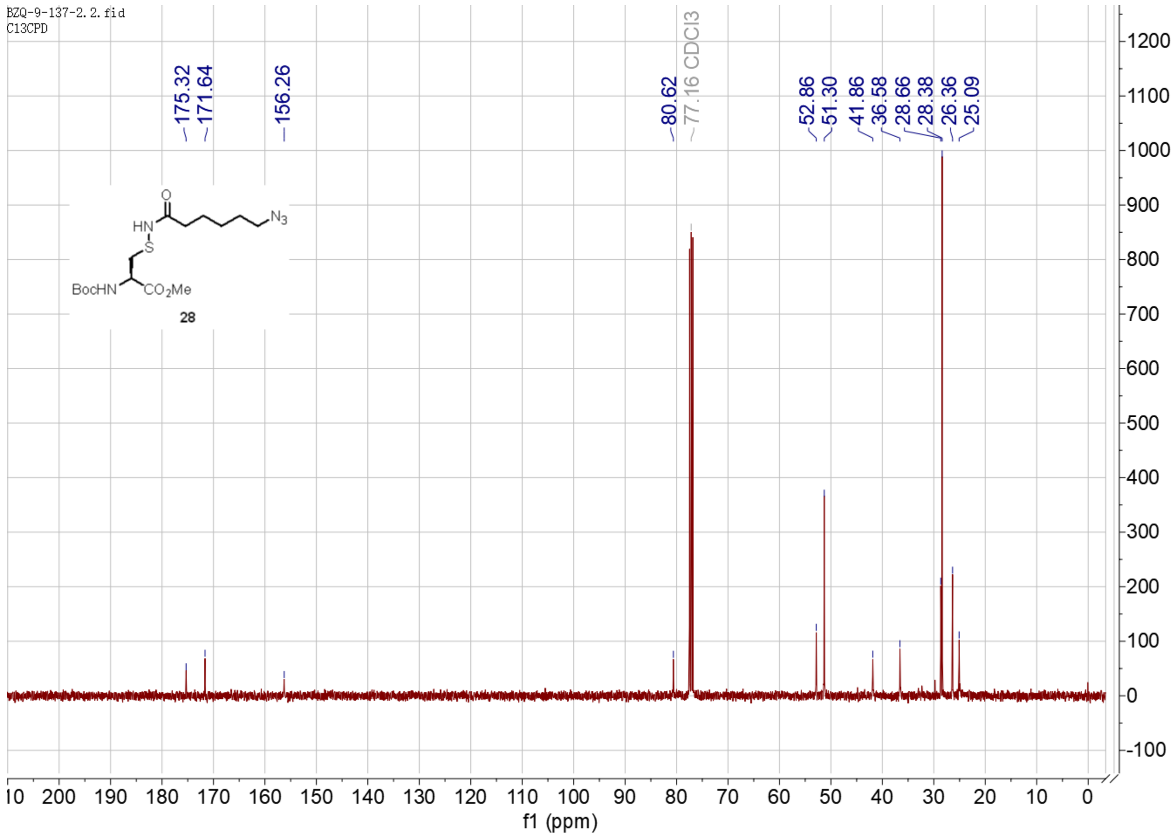

Supplementary Figure 161. ^{13}C NMR (101 MHz, $CDCl_3$, 293 K) spectrum of **28**.

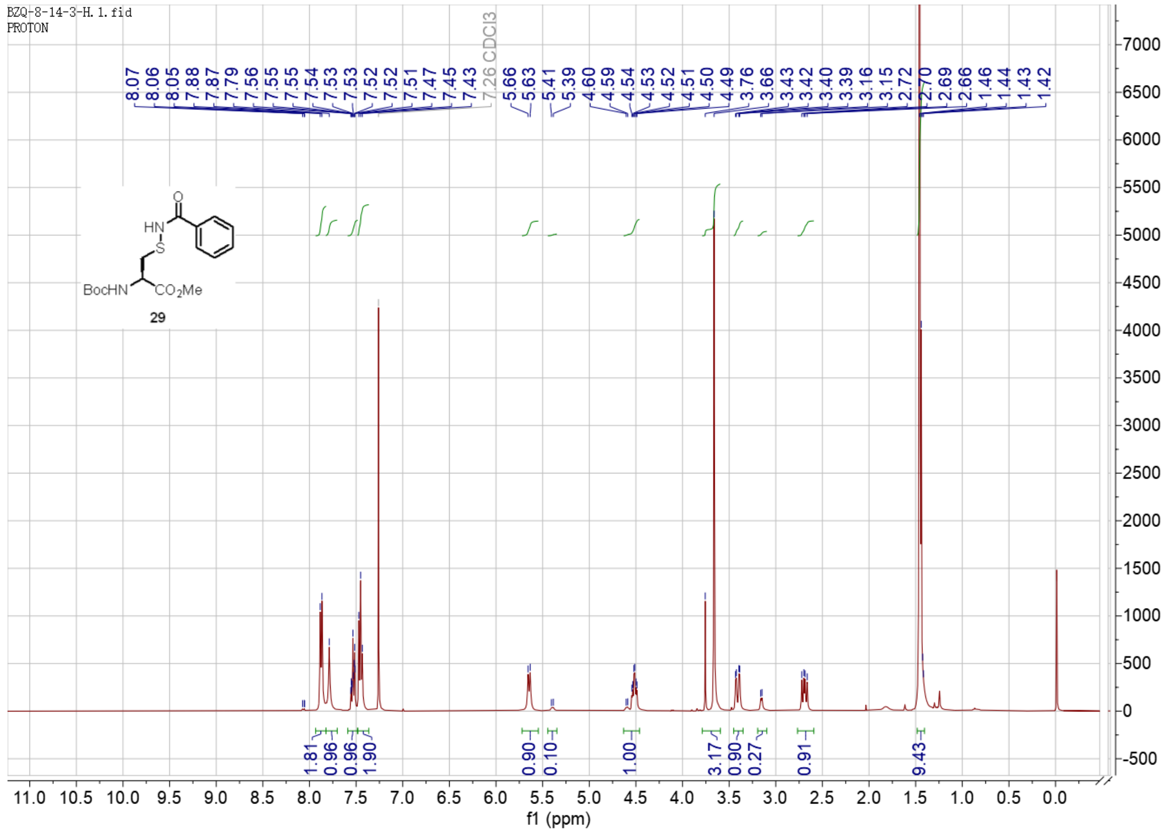

Supplementary Figure 162. ^{1}H NMR (400 MHz, $CDCl_3$, 293 K) spectrum of **29**.

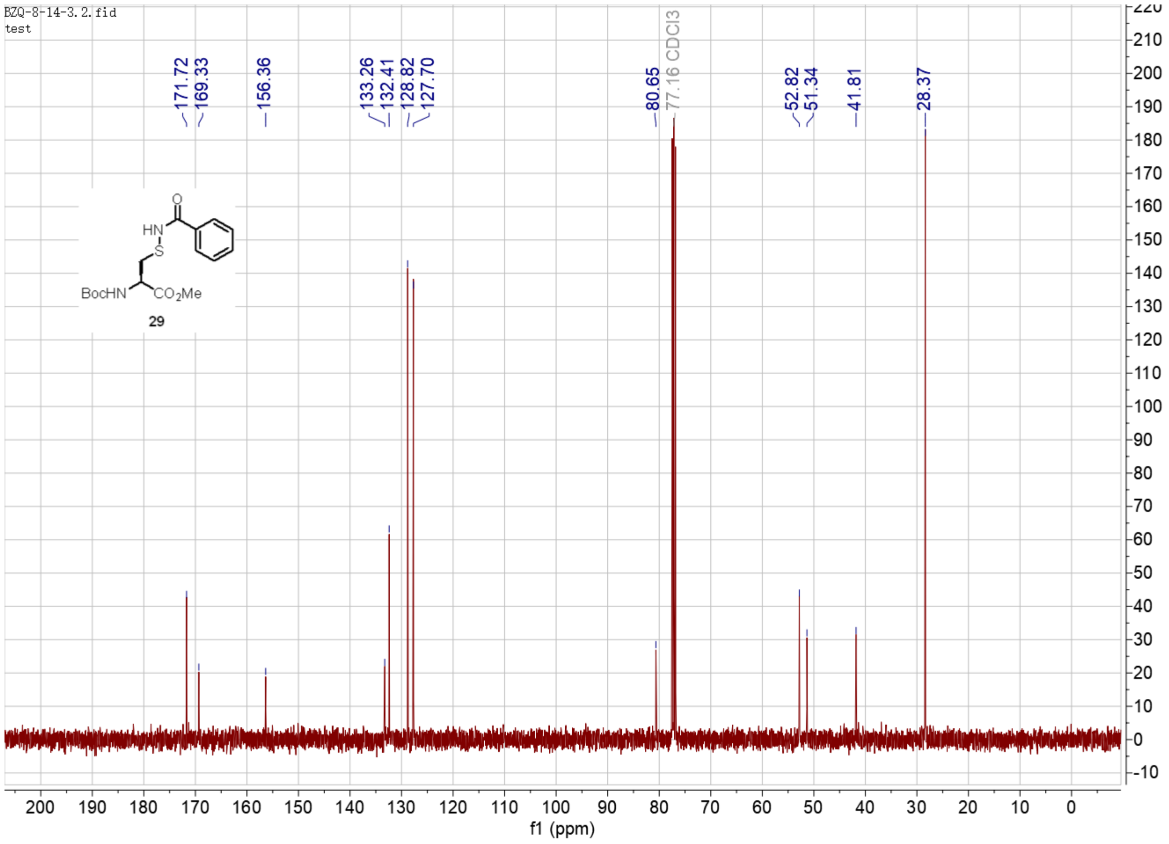

Supplementary Figure 163. ^{13}C NMR (101 MHz, $CDCl_3$, 293 K) spectrum of **29**.

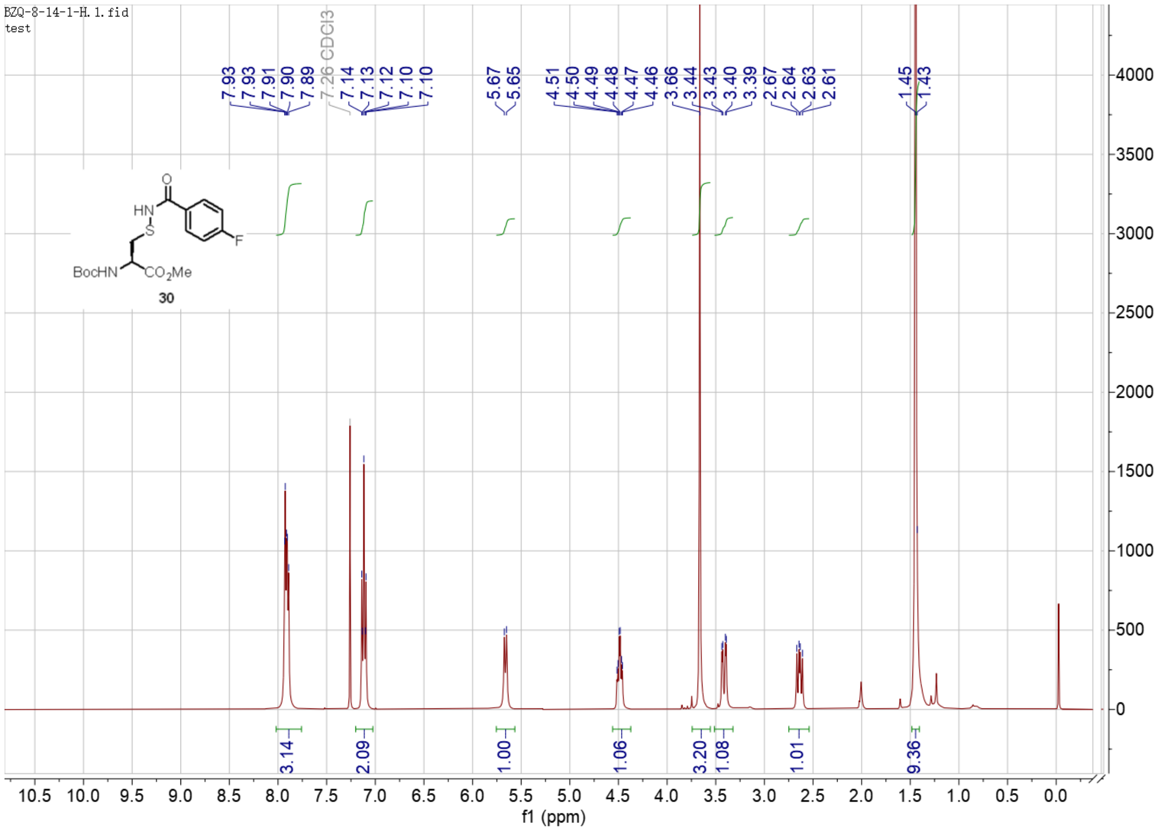

Supplementary Figure 164. ^{1}H NMR (400 MHz, $CDCl_3$, 293 K) spectrum of **30**.

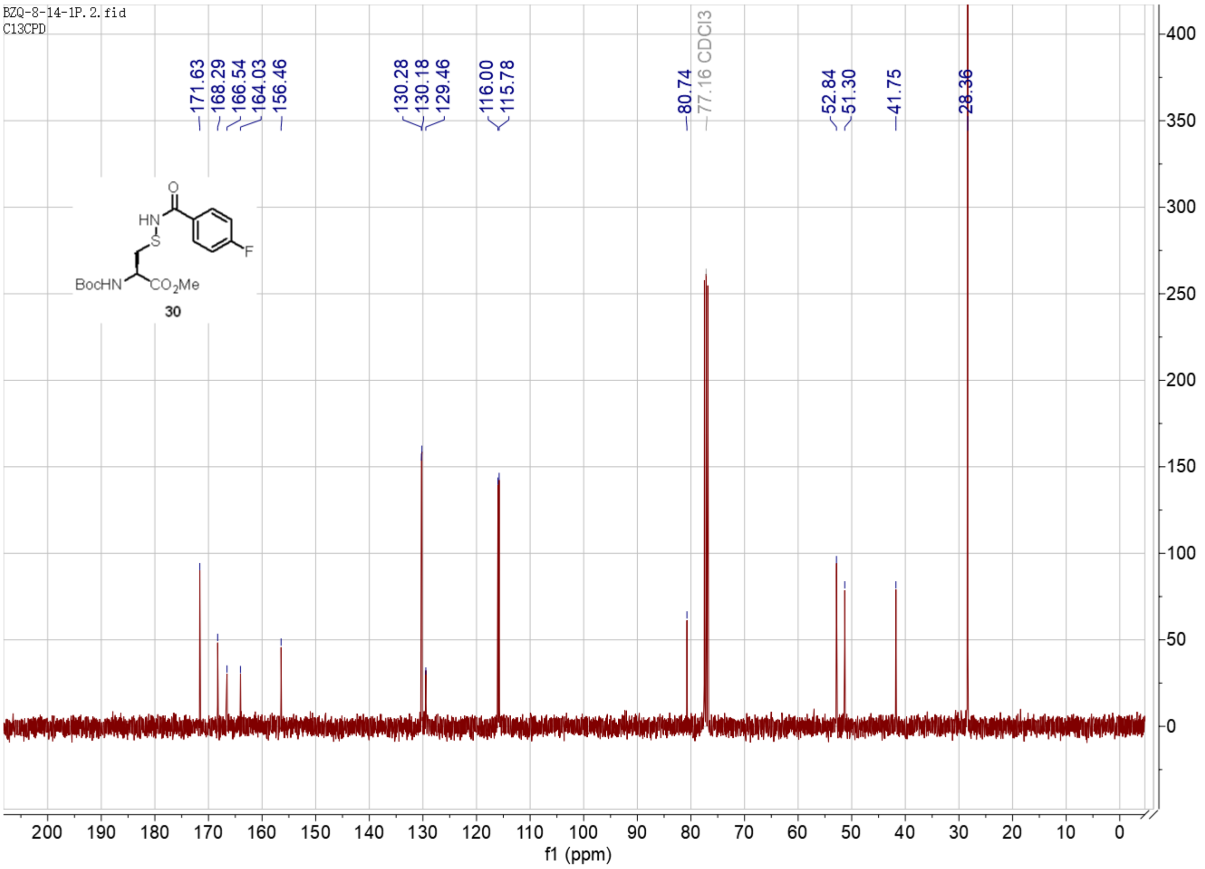

Supplementary Figure 165. ^{13}C NMR (101 MHz, $CDCl_3$, 293 K) spectrum of **30**.

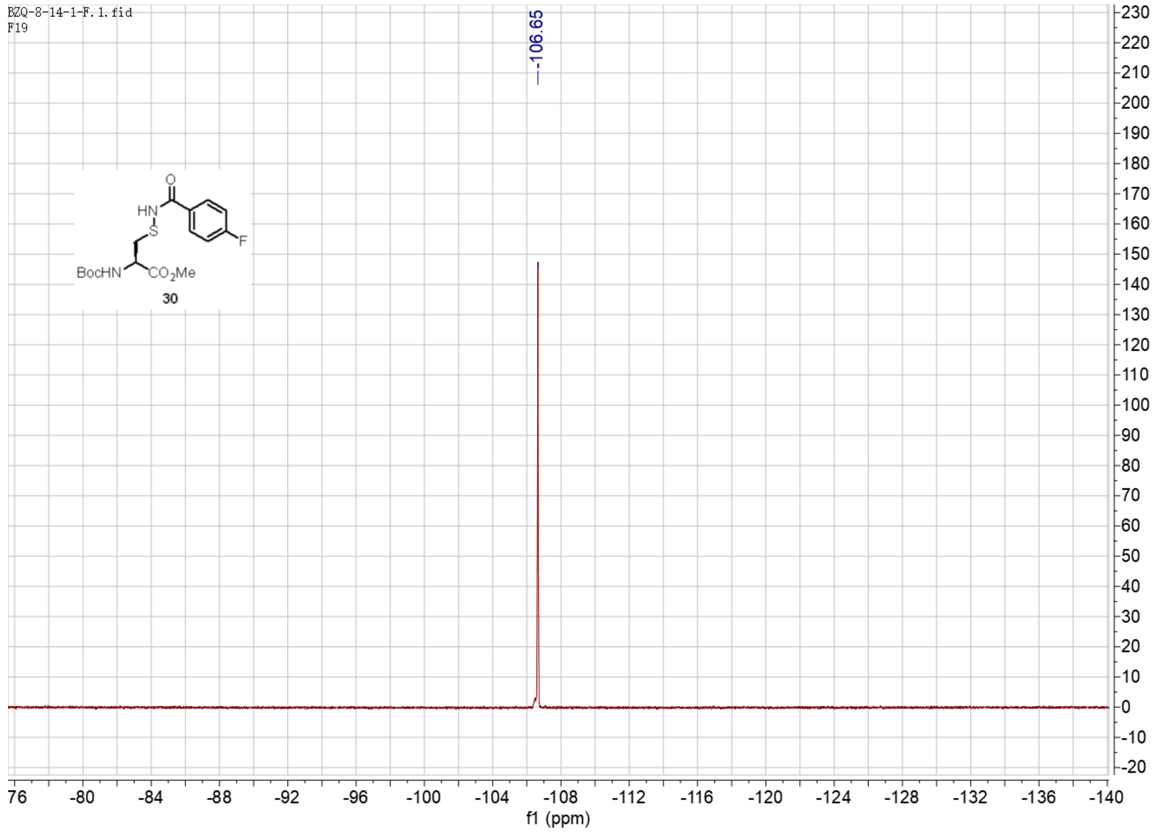

Supplementary Figure 166. ^{19}F NMR (376 MHz, $CDCl_3$, 293 K) spectrum of **30**.

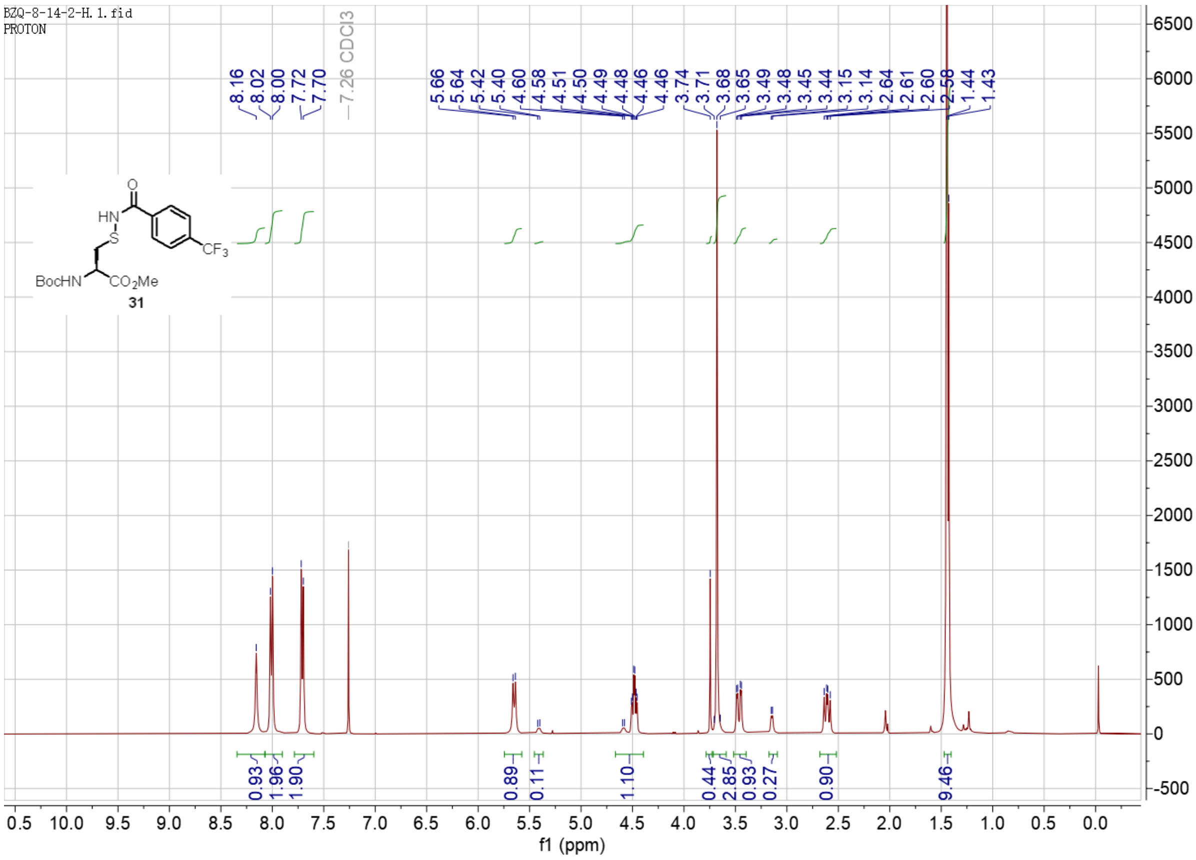

Supplementary Figure 167. ^{1}H NMR (400 MHz, $CDCl_3$, 293 K) spectrum of **31**.

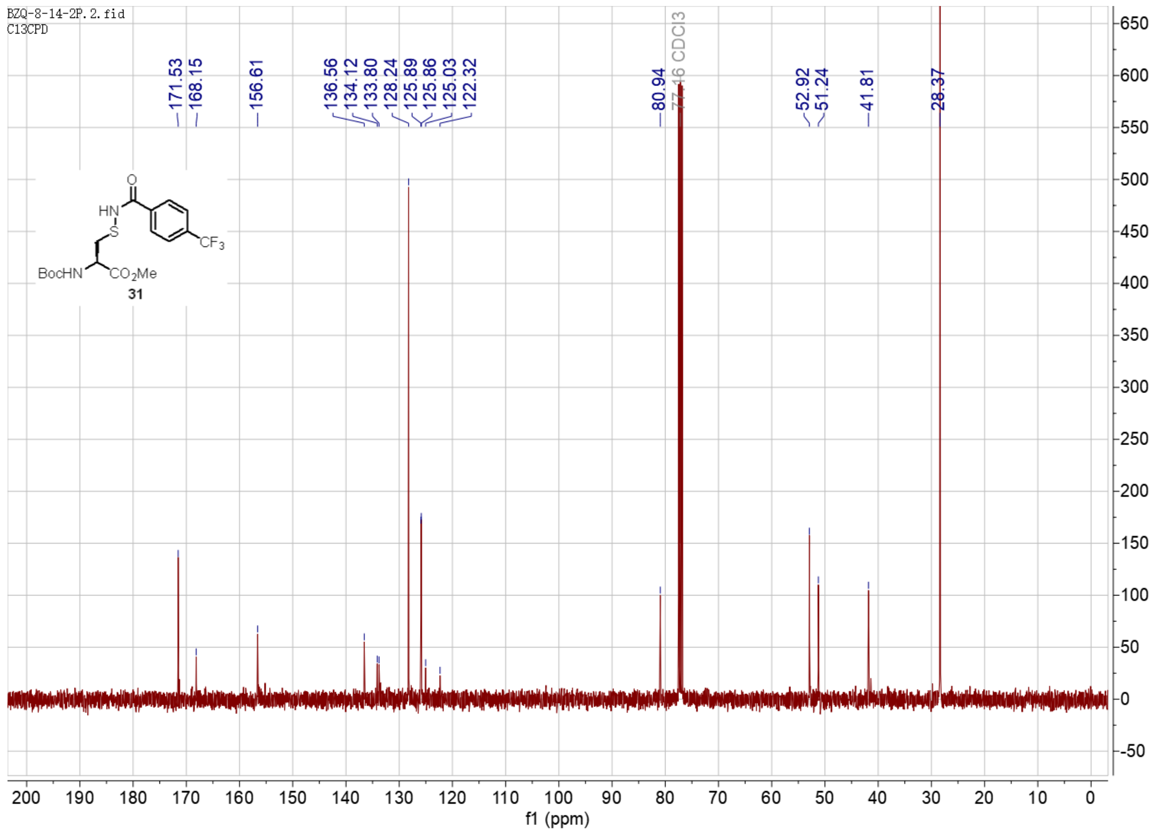

Supplementary Figure 168. ^{13}C NMR (101 MHz, $CDCl_3$, 293 K) spectrum of **31**.

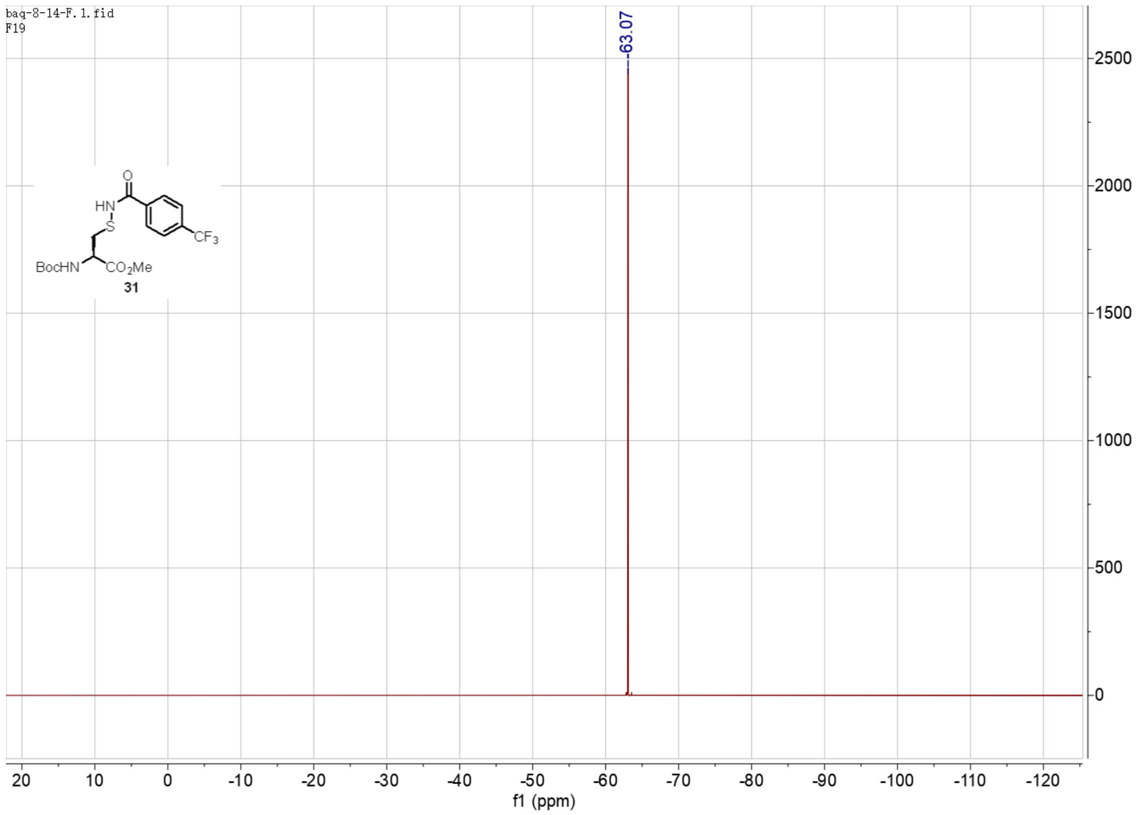

Supplementary Figure 169. ^{19}F NMR (376 MHz, $CDCl_3$, 293 K) spectrum of **31**.

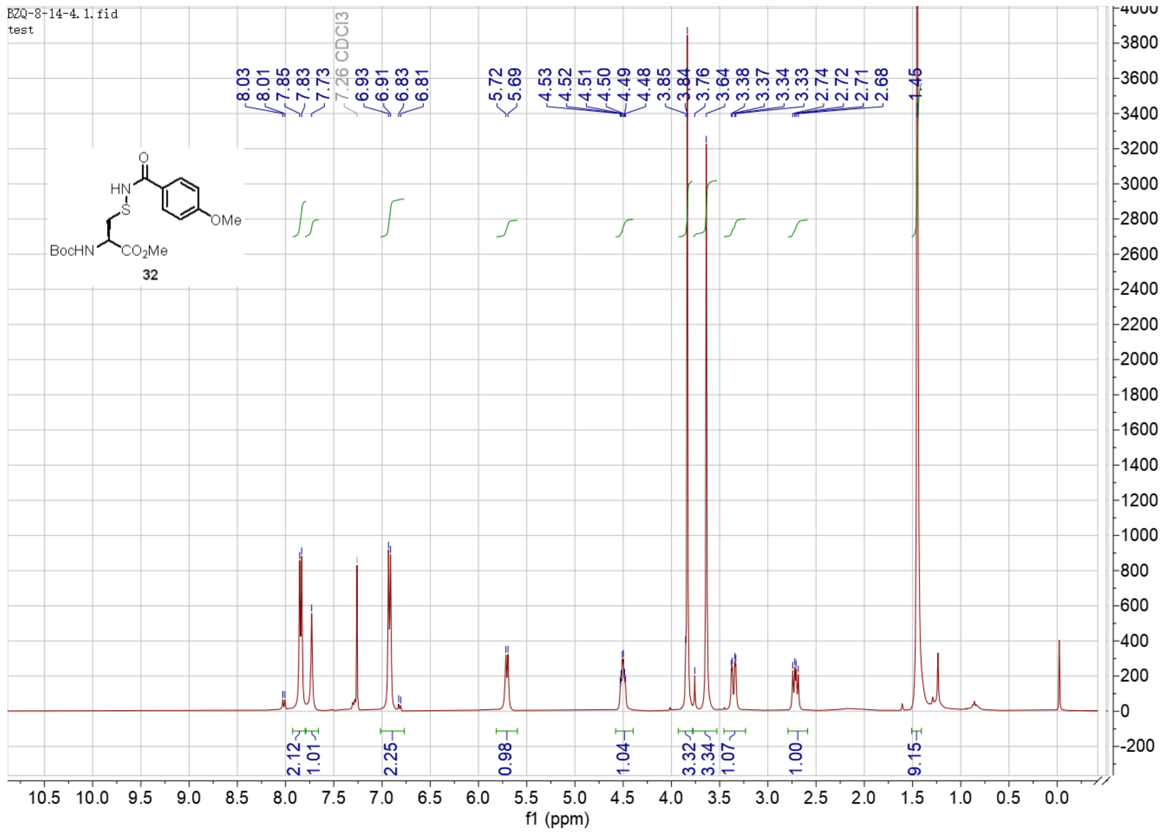

Supplementary Figure 170. ^{1}H NMR (400 MHz, $CDCl_3$, 293 K) spectrum of **32**.

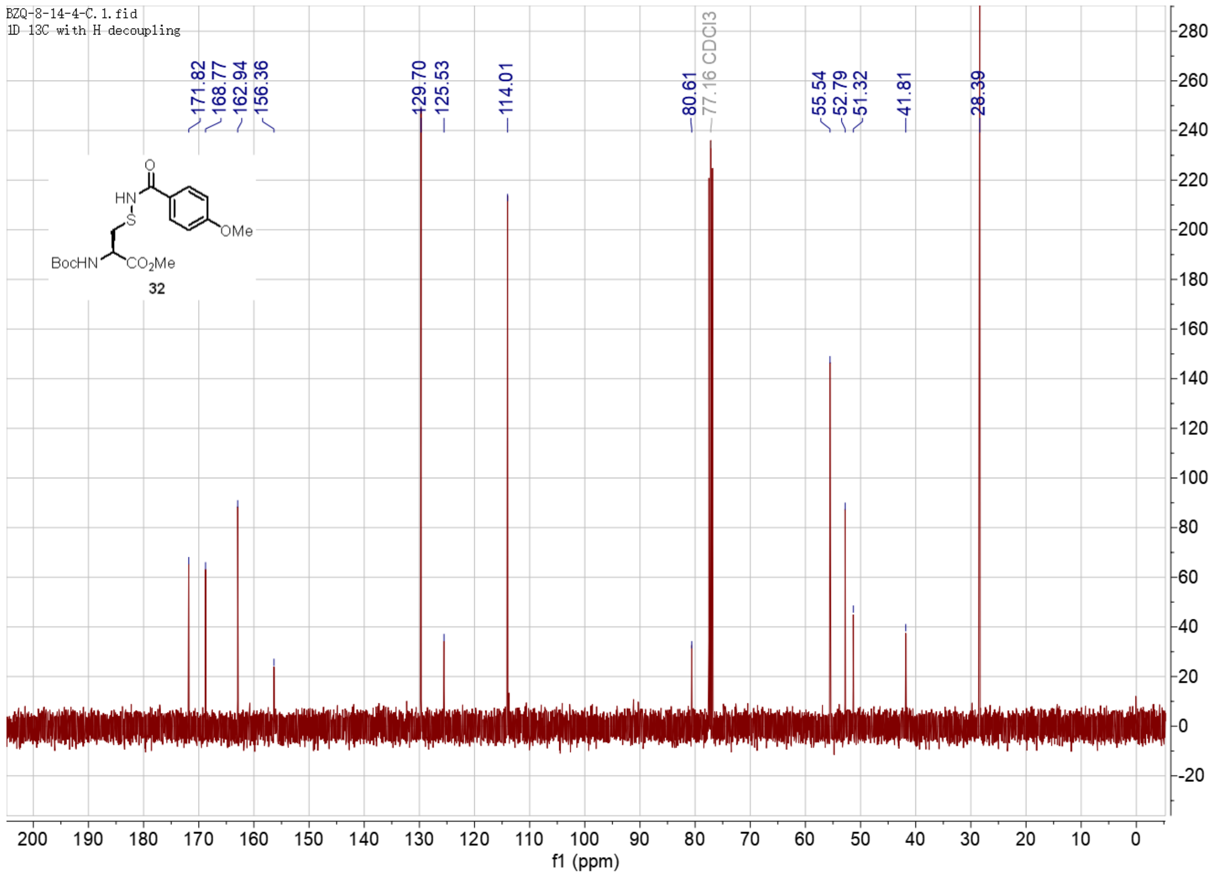

Supplementary Figure 171. ^{13}C NMR (101 MHz, $CDCl_3$, 293 K) spectrum of **32**.

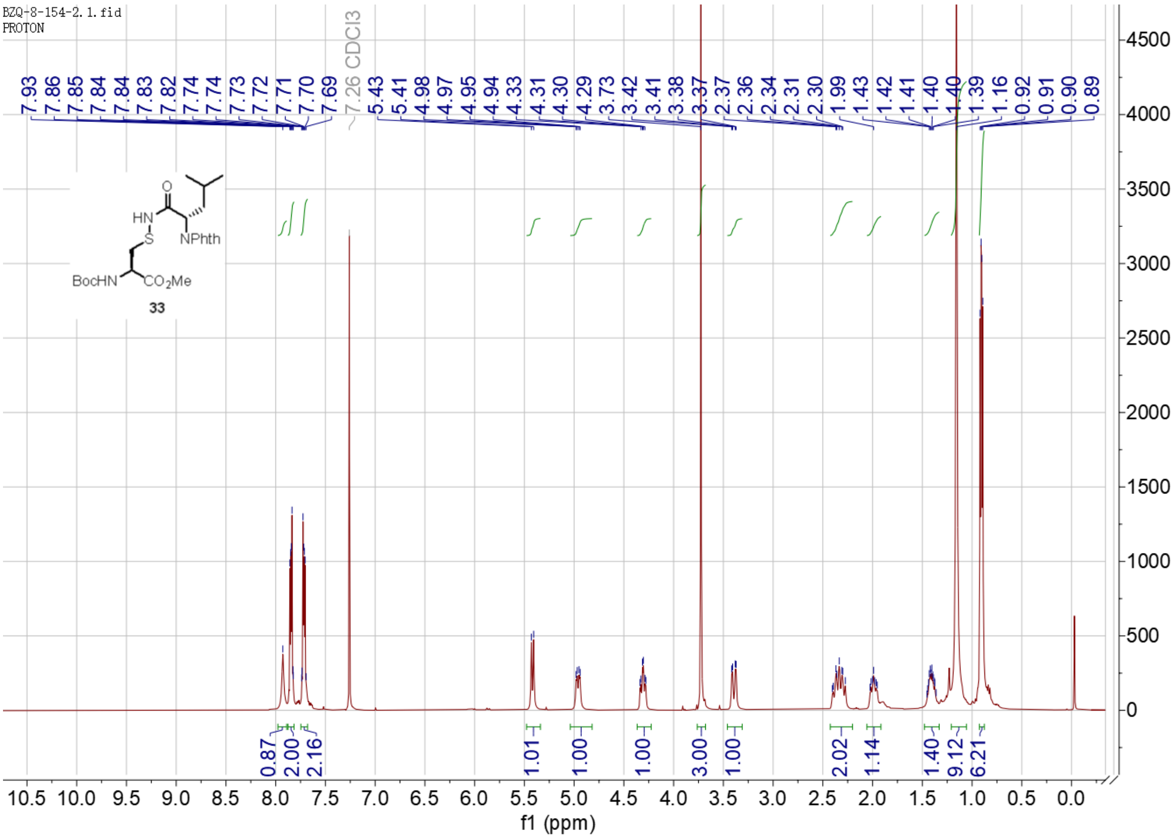

Supplementary Figure 172. ^{1}H NMR (400 MHz, $CDCl_3$, 293 K) spectrum of **33**.

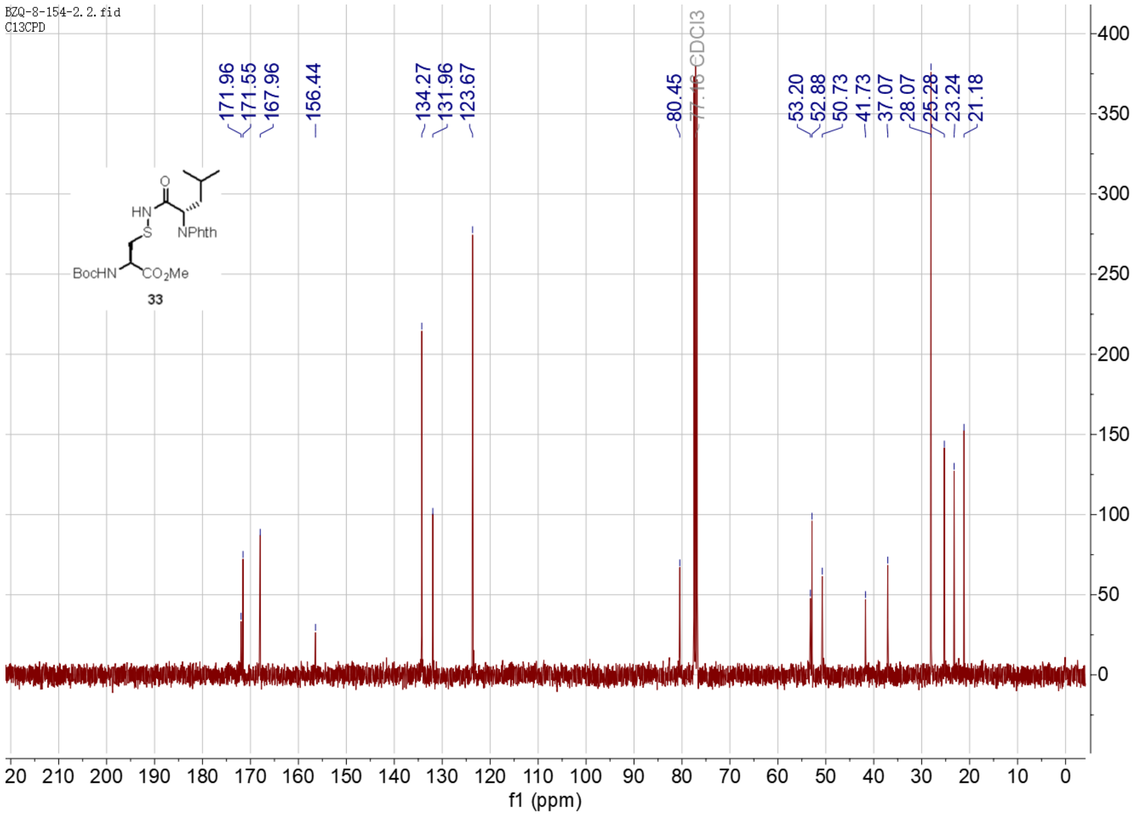

Supplementary Figure 173. ^{13}C NMR (101 MHz, $CDCl_3$, 293 K) spectrum of **33**.

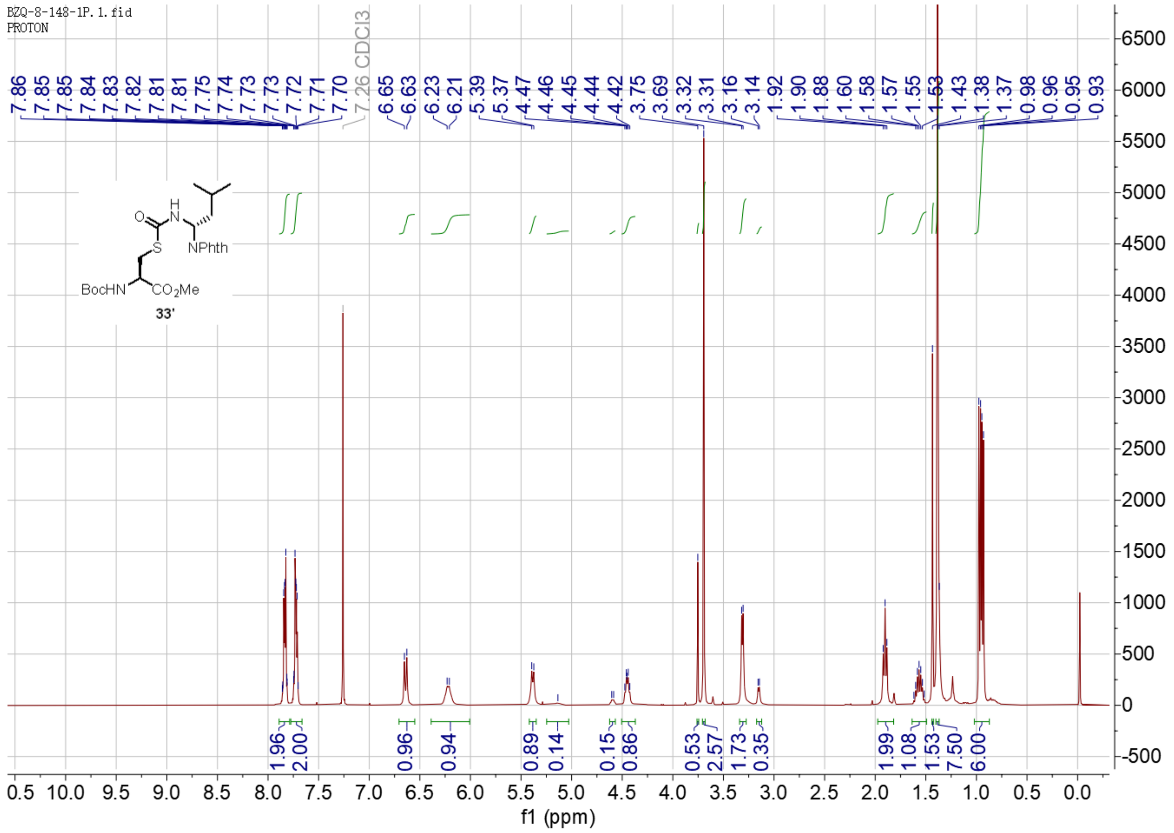

Supplementary Figure 174. ^{1}H NMR (400 MHz, $CDCl_3$, 293 K) spectrum of **33'**.

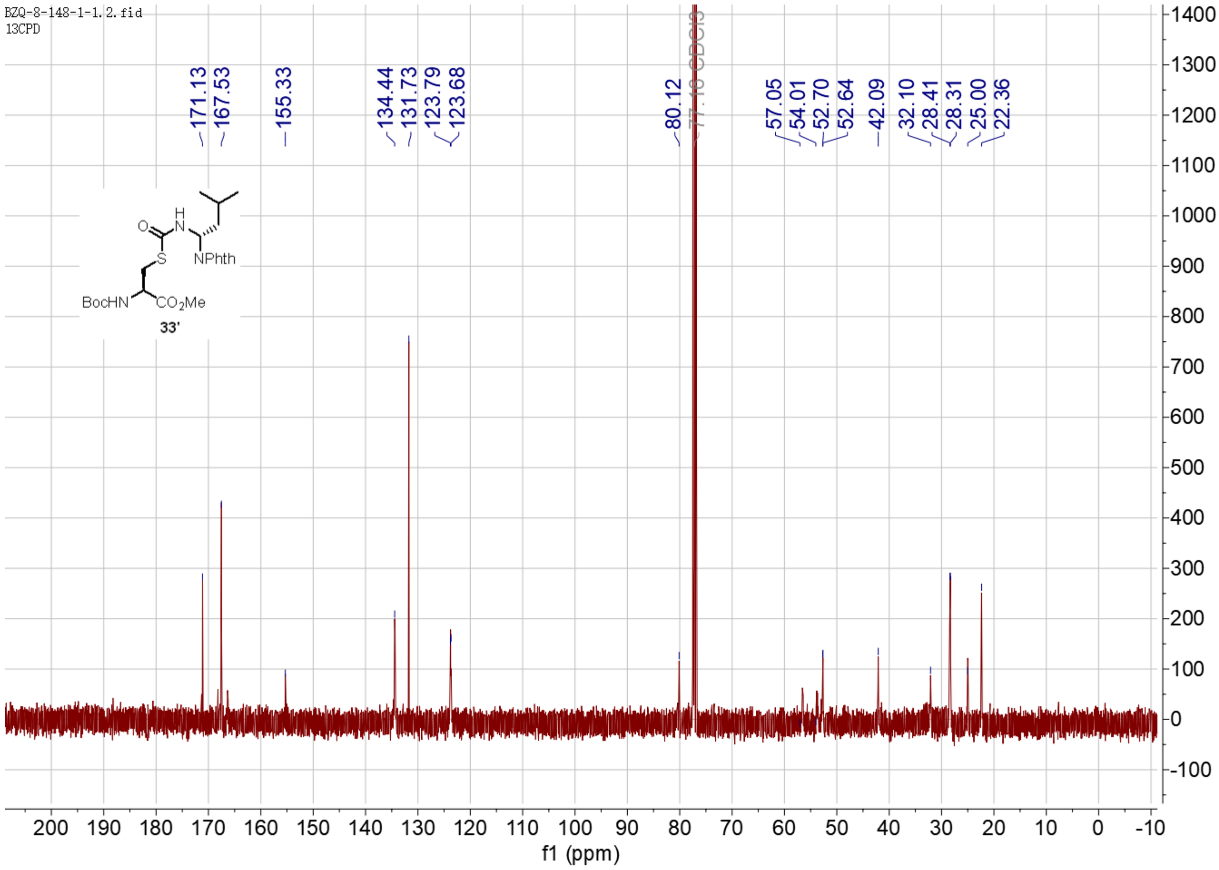

Supplementary Figure 175. ^{13}C NMR (101 MHz, $CDCl_3$, 293 K) spectrum of **33'**.

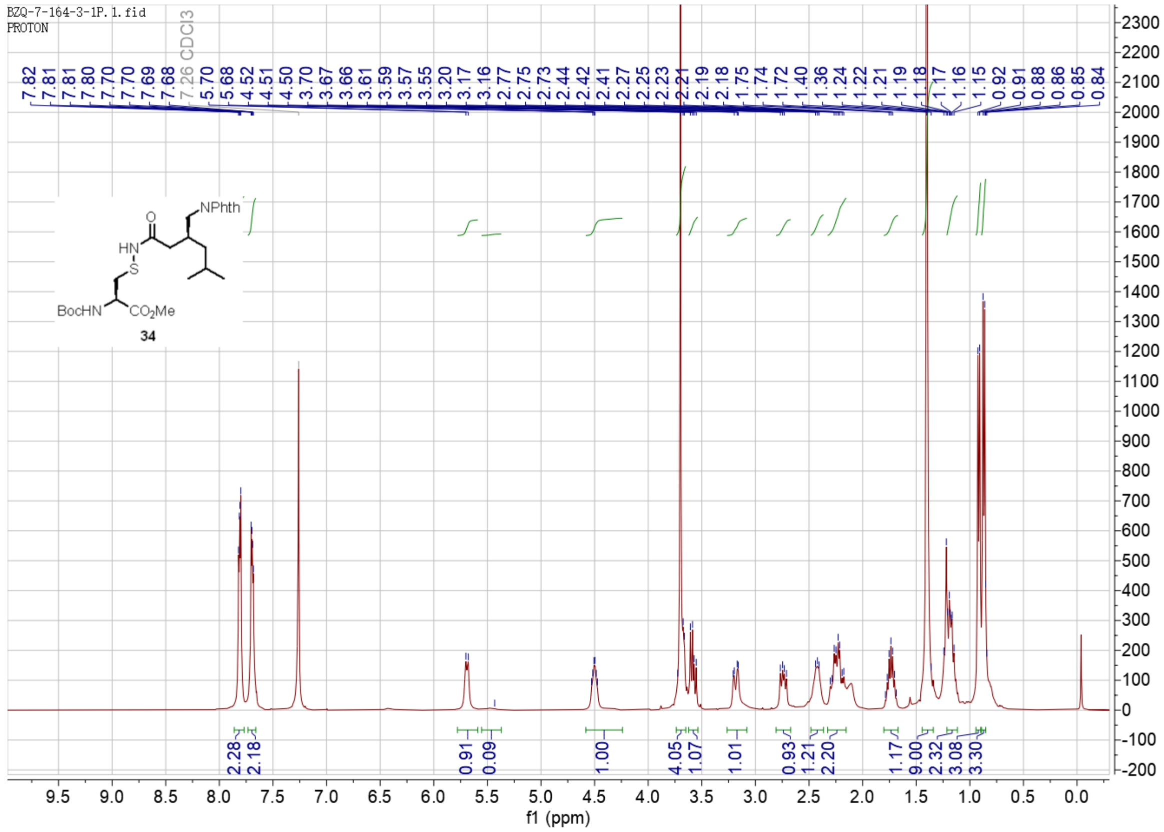

Supplementary Figure 176. ^{1}H NMR (400 MHz, $CDCl_3$, 293 K) spectrum of **34**.

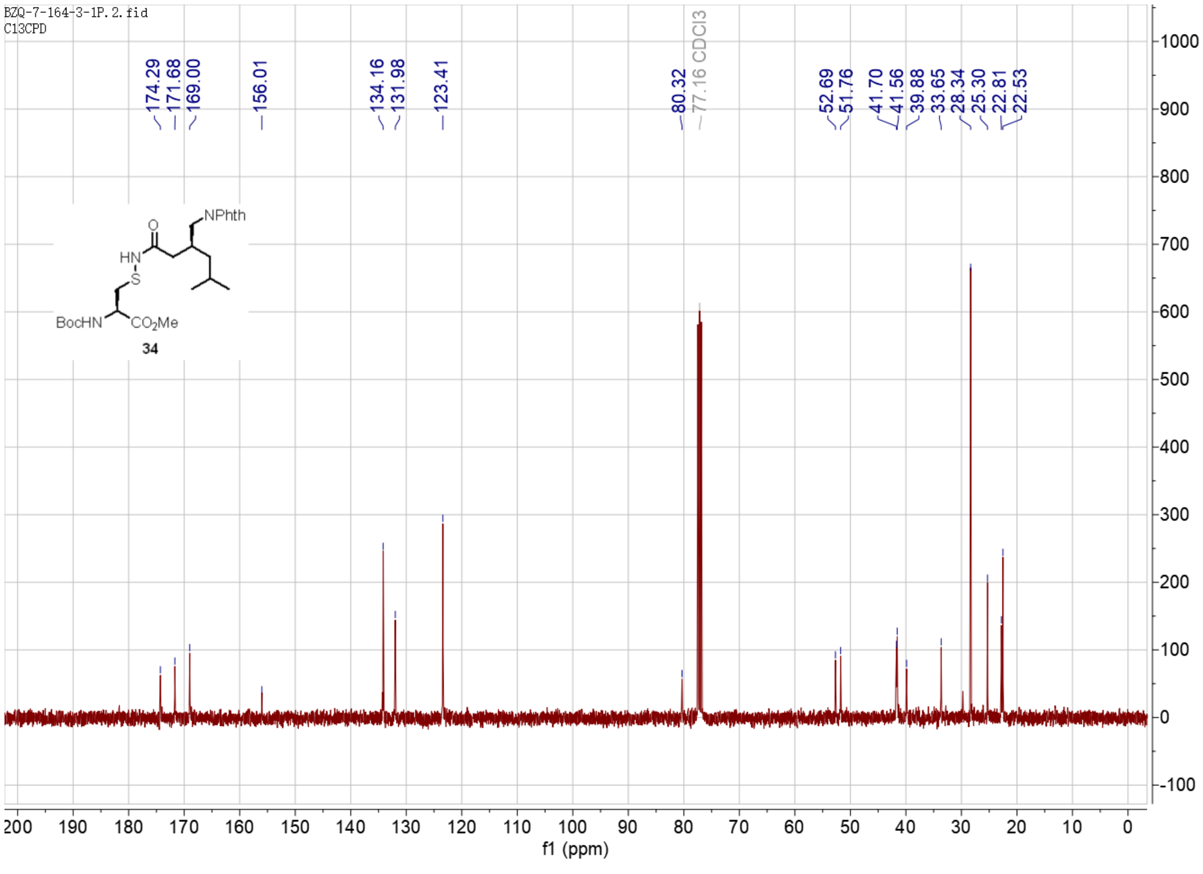

Supplementary Figure 177. ^{13}C NMR (101 MHz, $CDCl_3$, 293 K) spectrum of **34**.

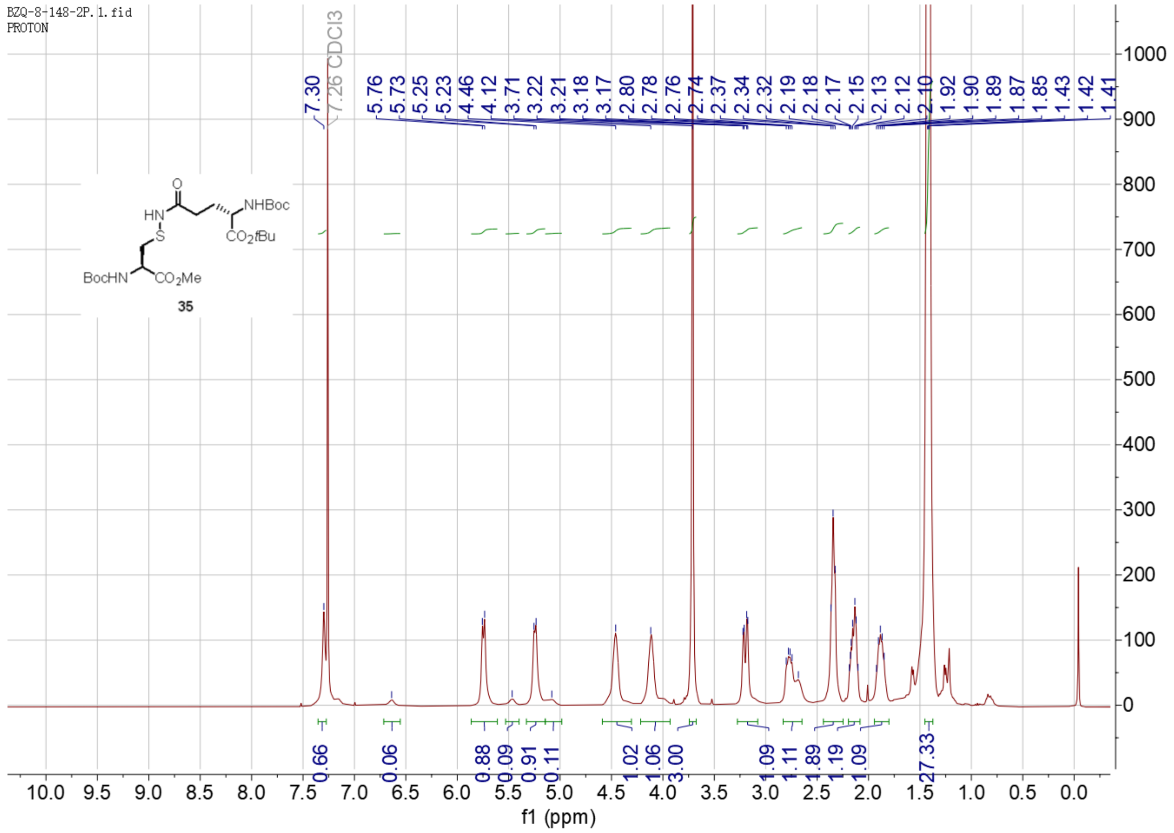

Supplementary Figure 178. ¹H NMR (400 MHz, $CDCl_3$, 293 K) spectrum of **35**.

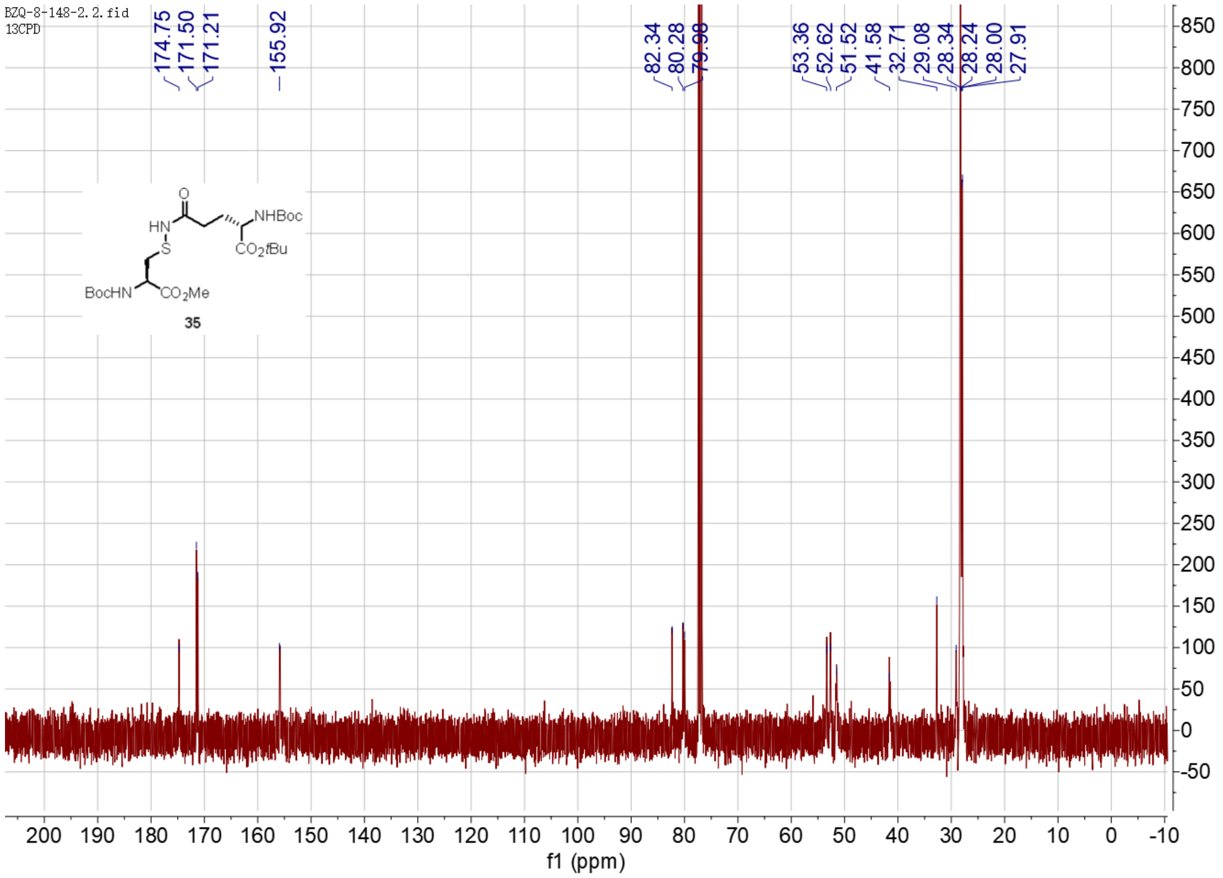

Supplementary Figure 179. ¹³C NMR (101 MHz, $CDCl_3$, 293 K) spectrum of **35**.

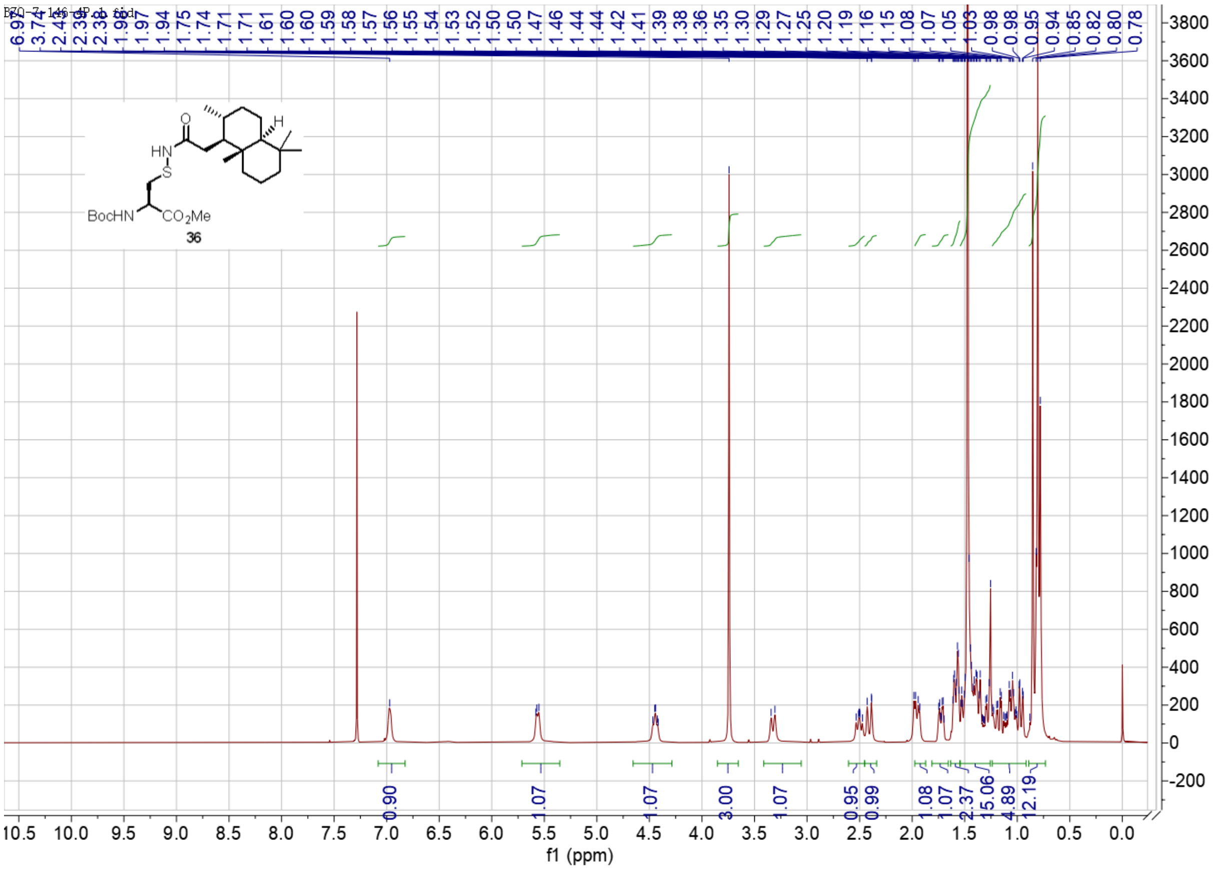

Supplementary Figure 180. ^{1}H NMR (400 MHz, $CDCl_3$, 293 K) spectrum of **36**.

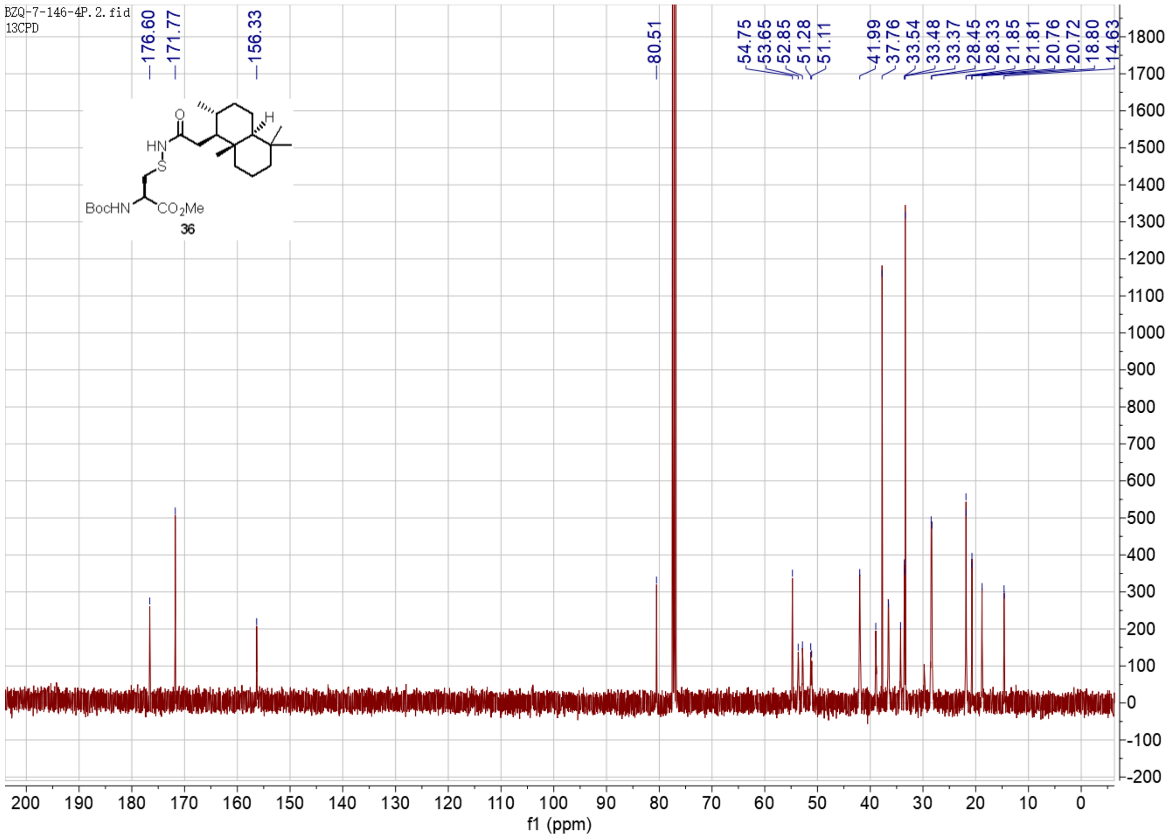

Supplementary Figure 181. ^{13}C NMR (101 MHz, $CDCl_3$, 293 K) spectrum of **36**.

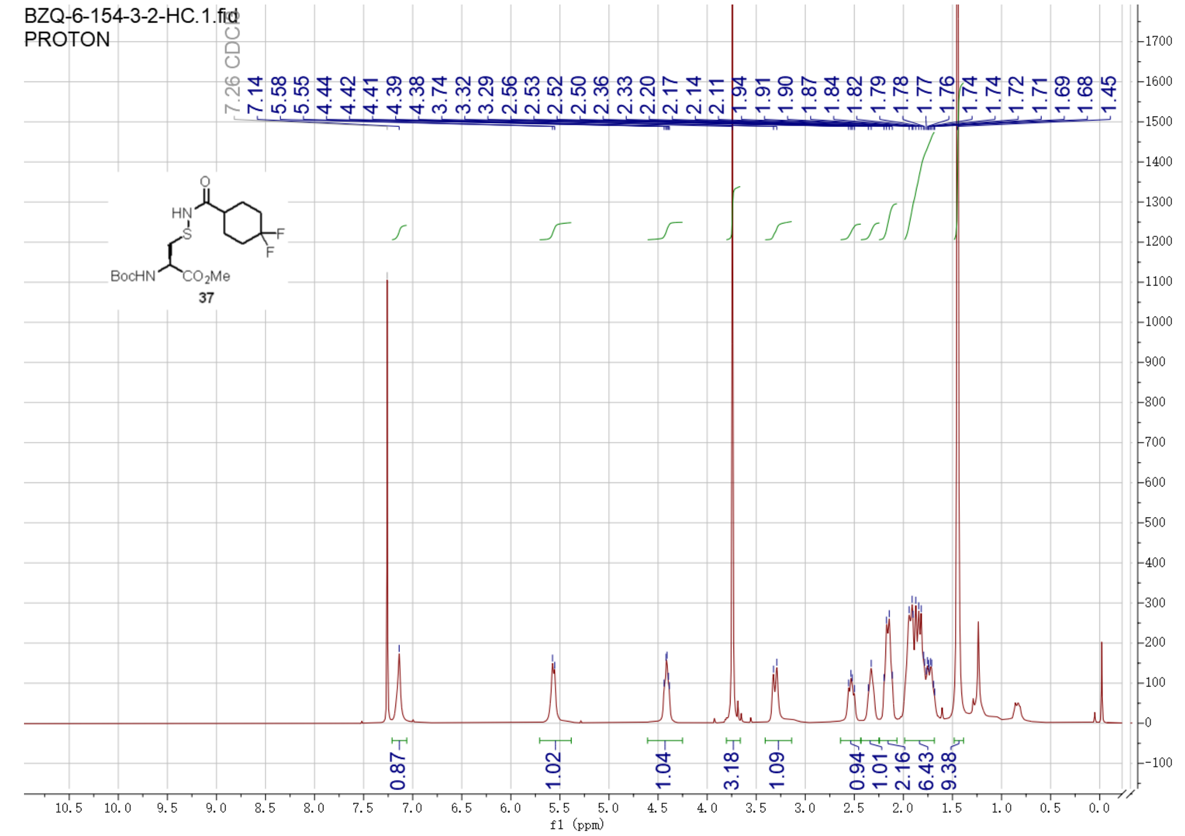

Supplementary Figure 182. ^{1}H NMR (400 MHz, $CDCl_3$, 293 K) spectrum of **37**.

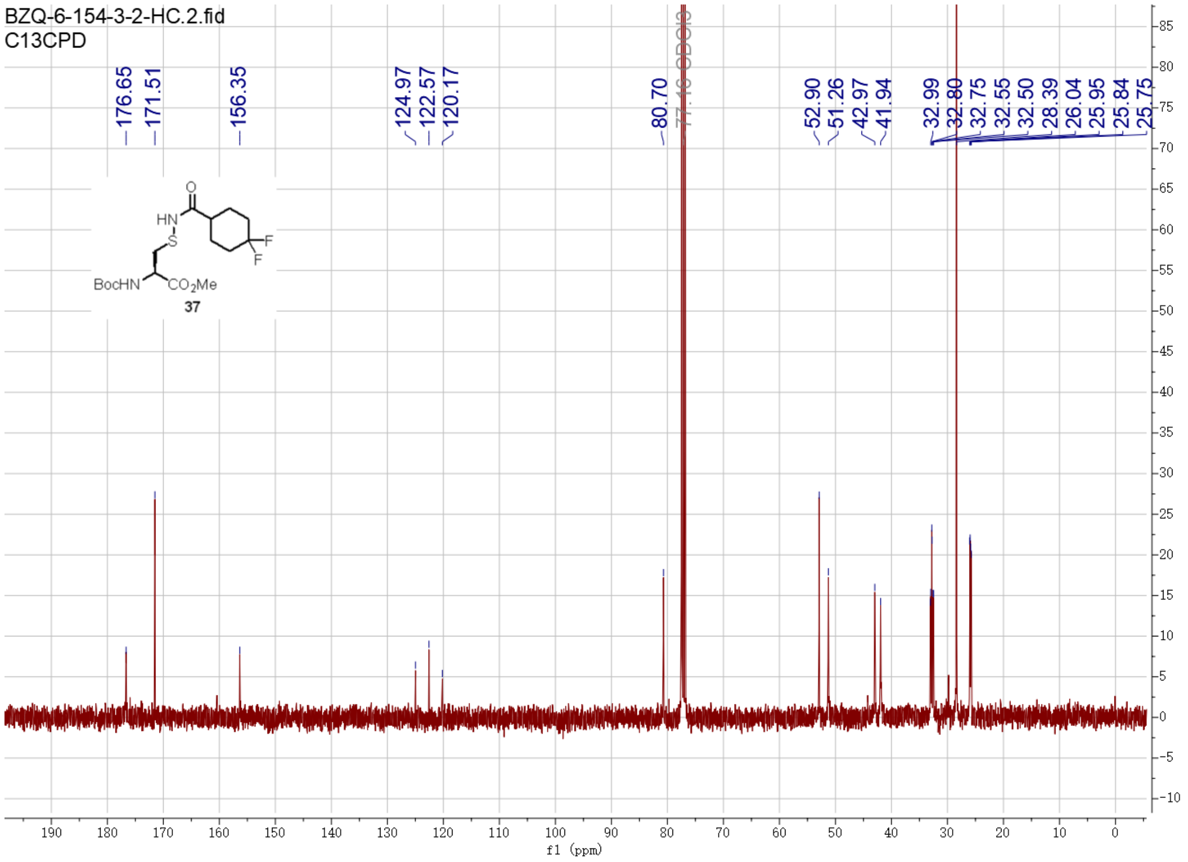

Supplementary Figure 183. ^{13}C NMR (101 MHz, $CDCl_3$, 293 K) spectrum of **37**.

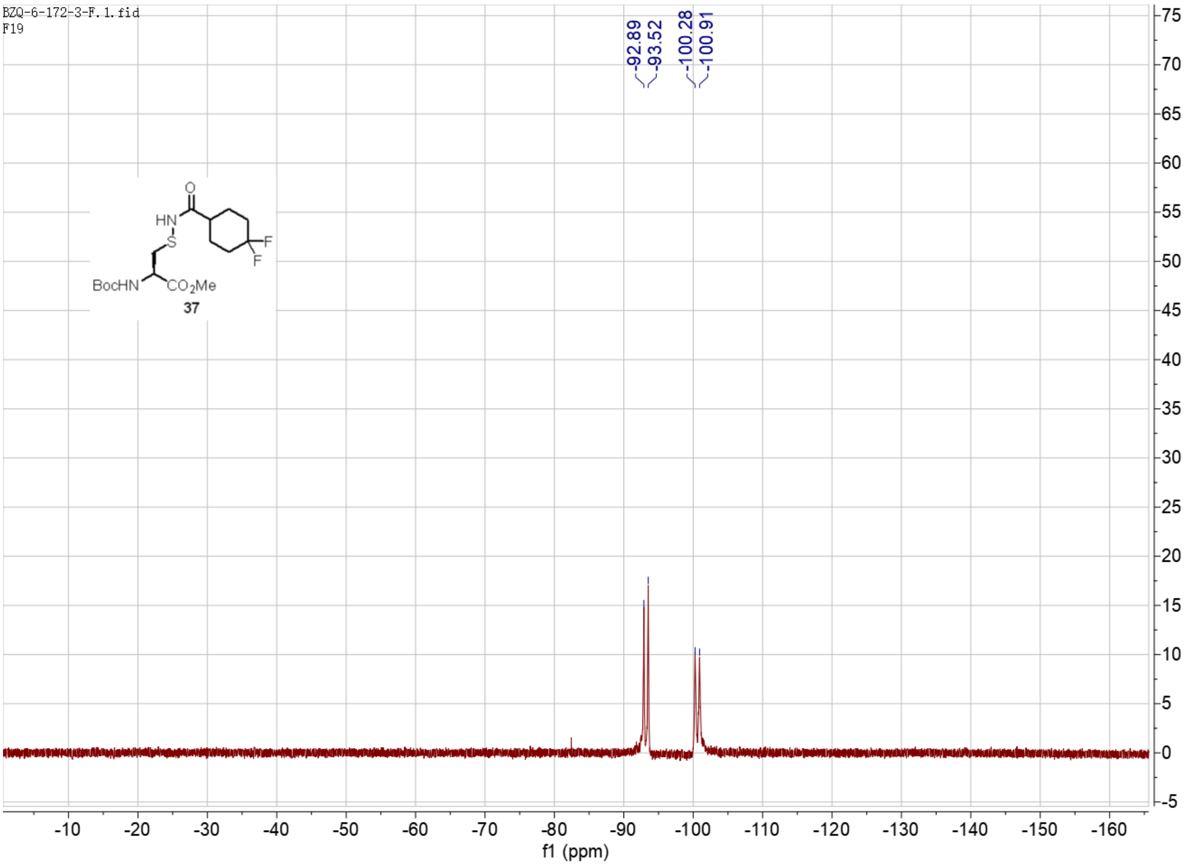

Supplementary Figure 184. ^{19}F NMR (376 MHz, $CDCl_3$, 293 K) spectrum of **37**.

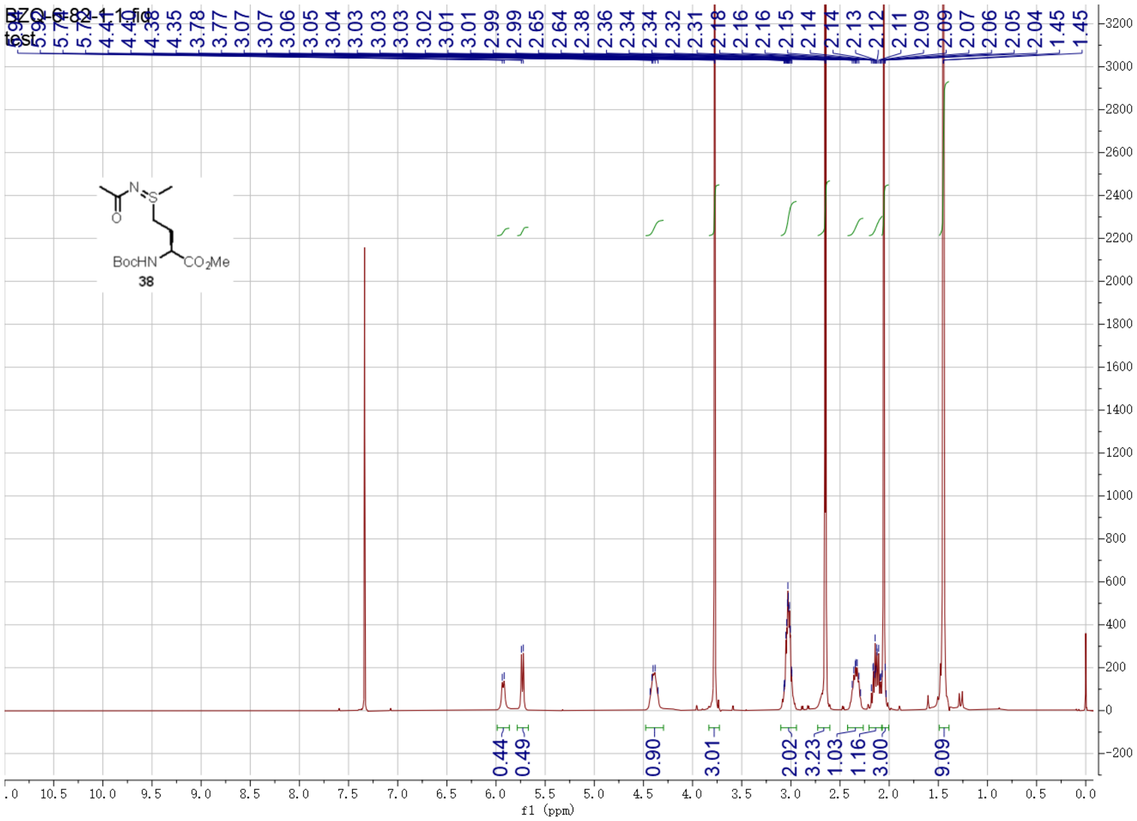

Supplementary Figure 185. ^{1}H NMR (400 MHz, $CDCl_3$, 293 K) spectrum of **38**.

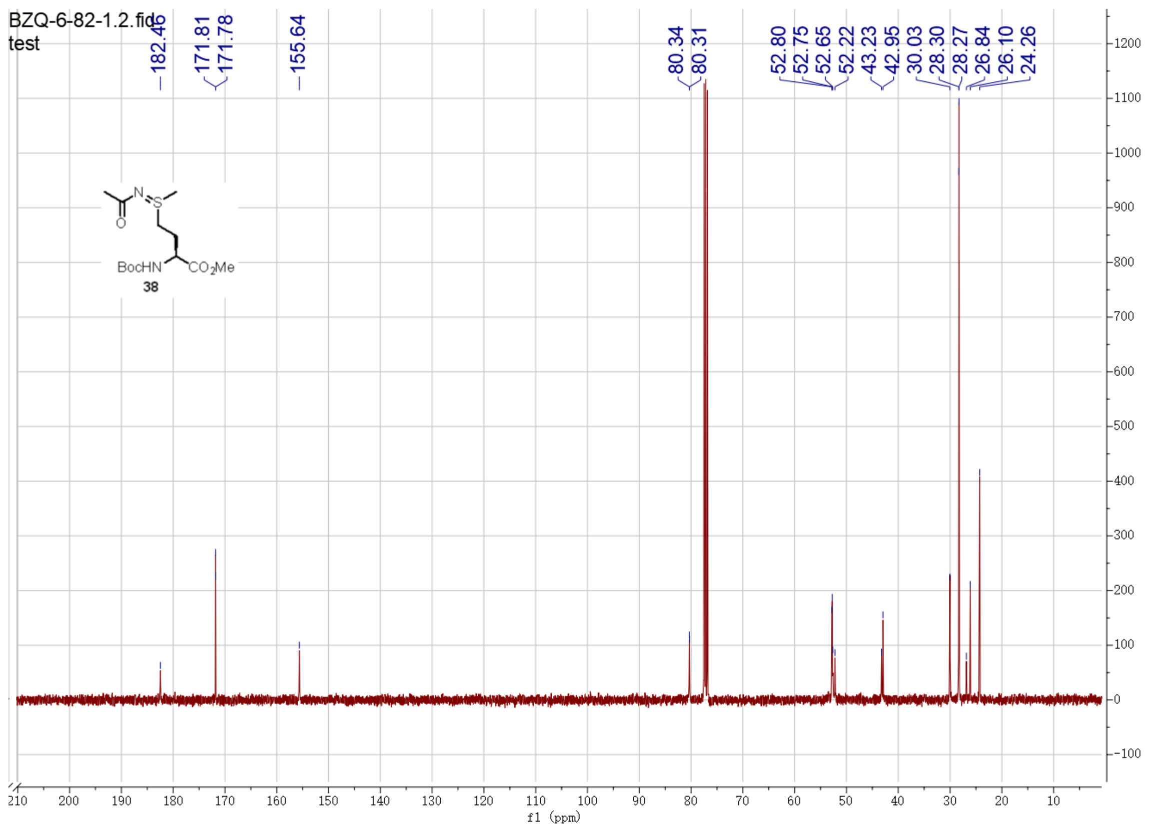

Supplementary Figure 186. ^{13}C NMR (101 MHz, $CDCl_3$, 293 K) spectrum of **38**.

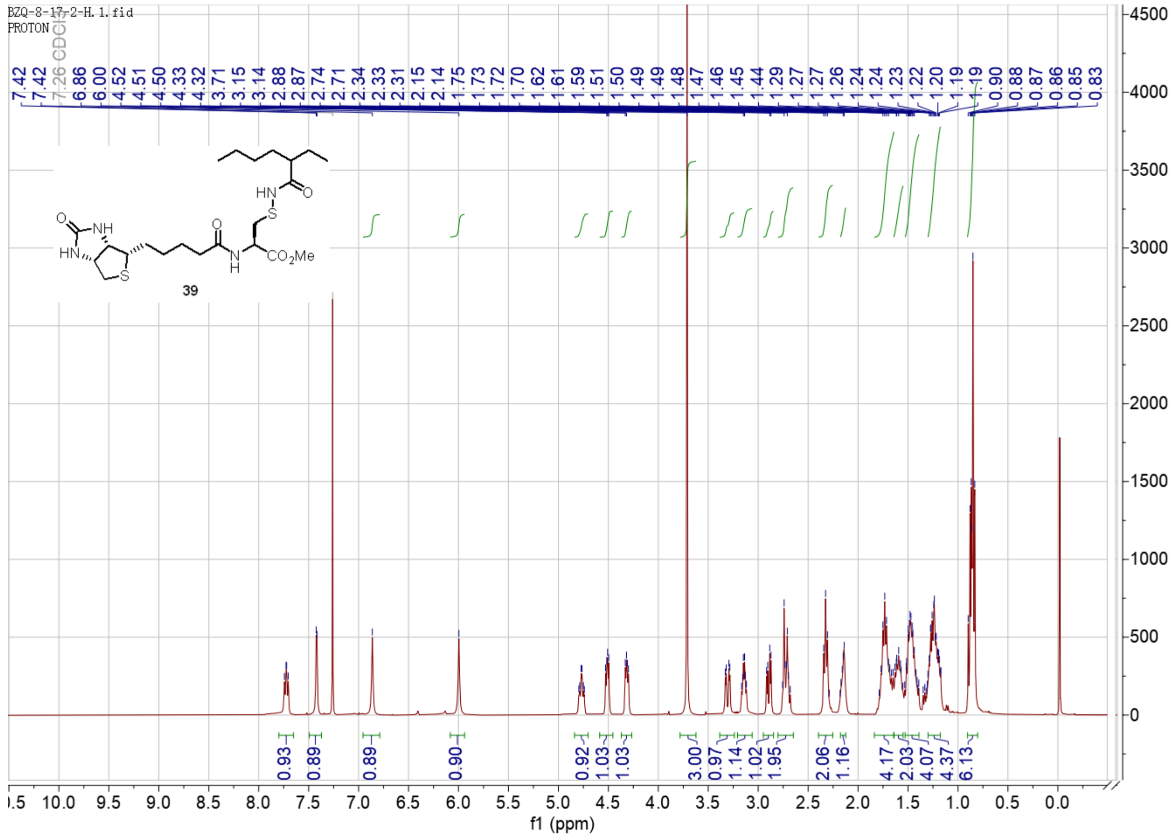

Supplementary Figure 187. ^{1}H NMR (400 MHz, $CDCl_3$, 293 K) spectrum of **39**.

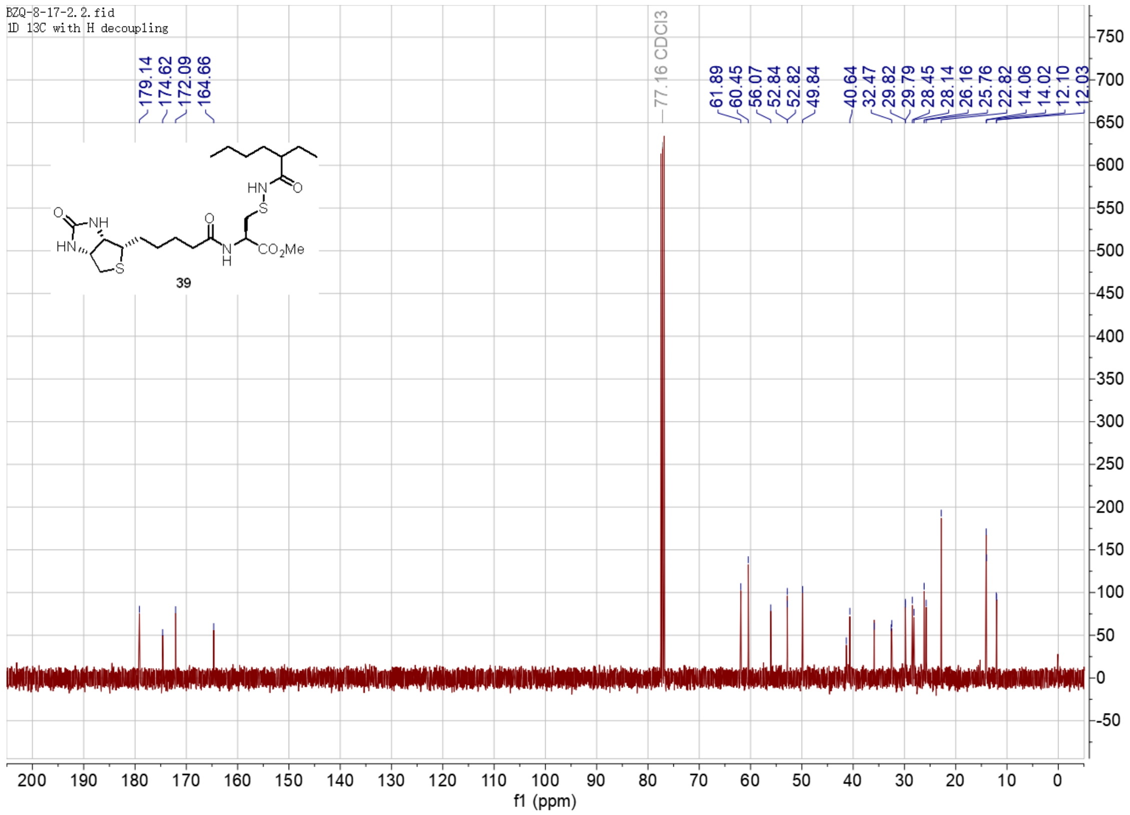

Supplementary Figure 188. ^{13}C NMR (101 MHz, $CDCl_3$, 293 K) spectrum of **39**.

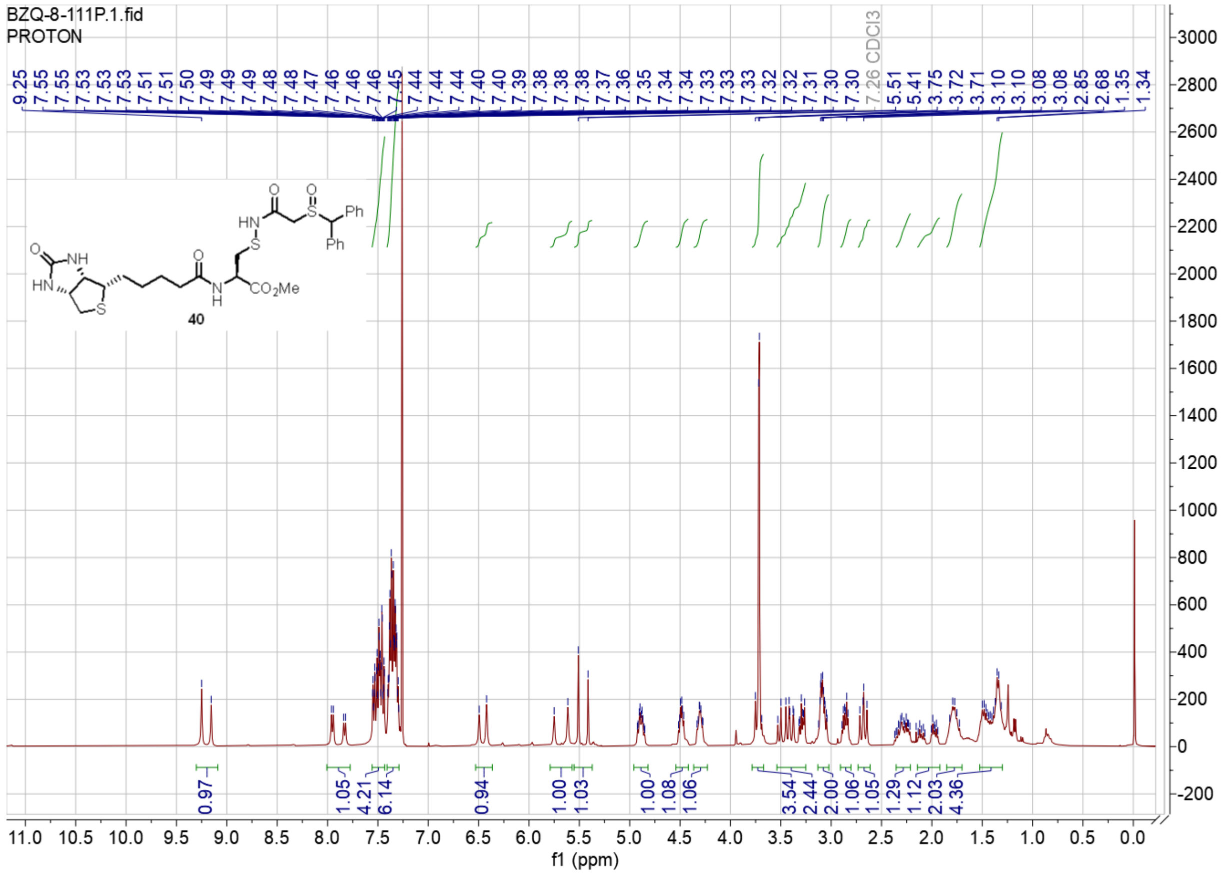

Supplementary Figure 189. ^{1}H NMR (400 MHz, $CDCl_3$, 293 K) spectrum of **40**.

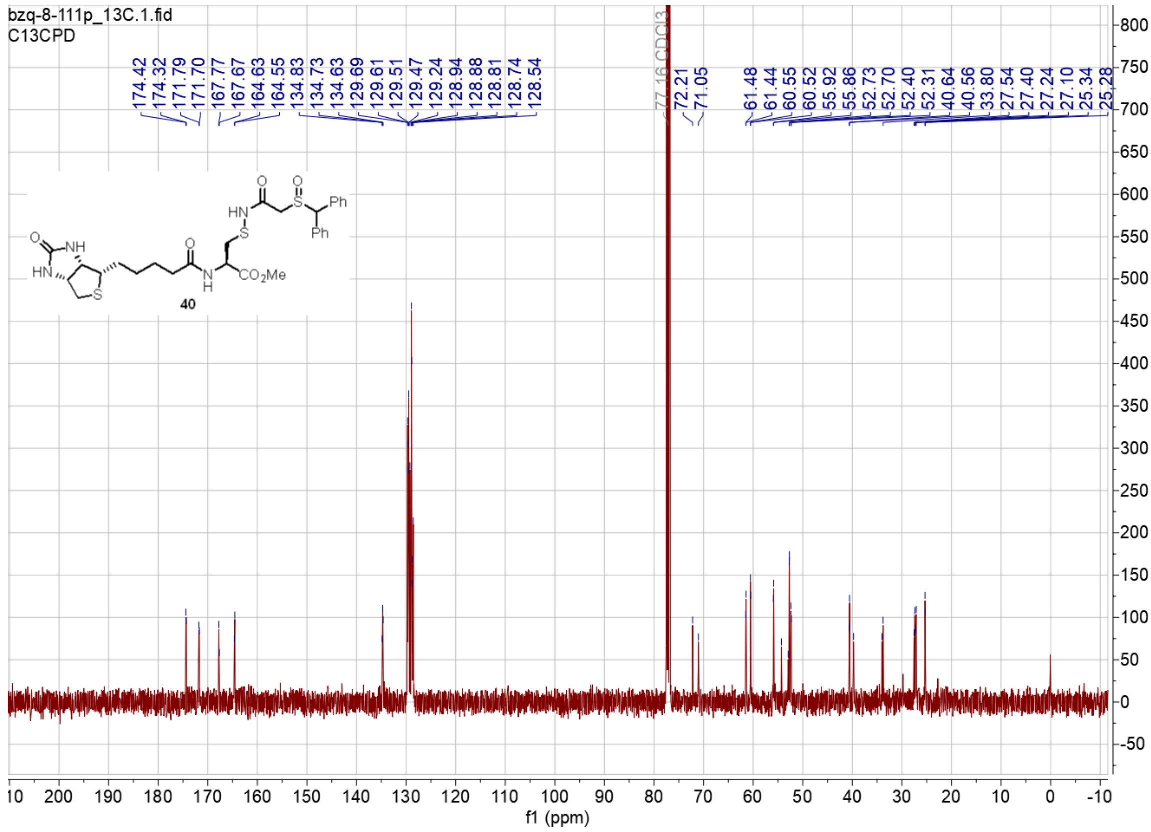

Supplementary Figure 190. ^{13}C NMR (101 MHz, $CDCl_3$, 293 K) spectrum of **40**.

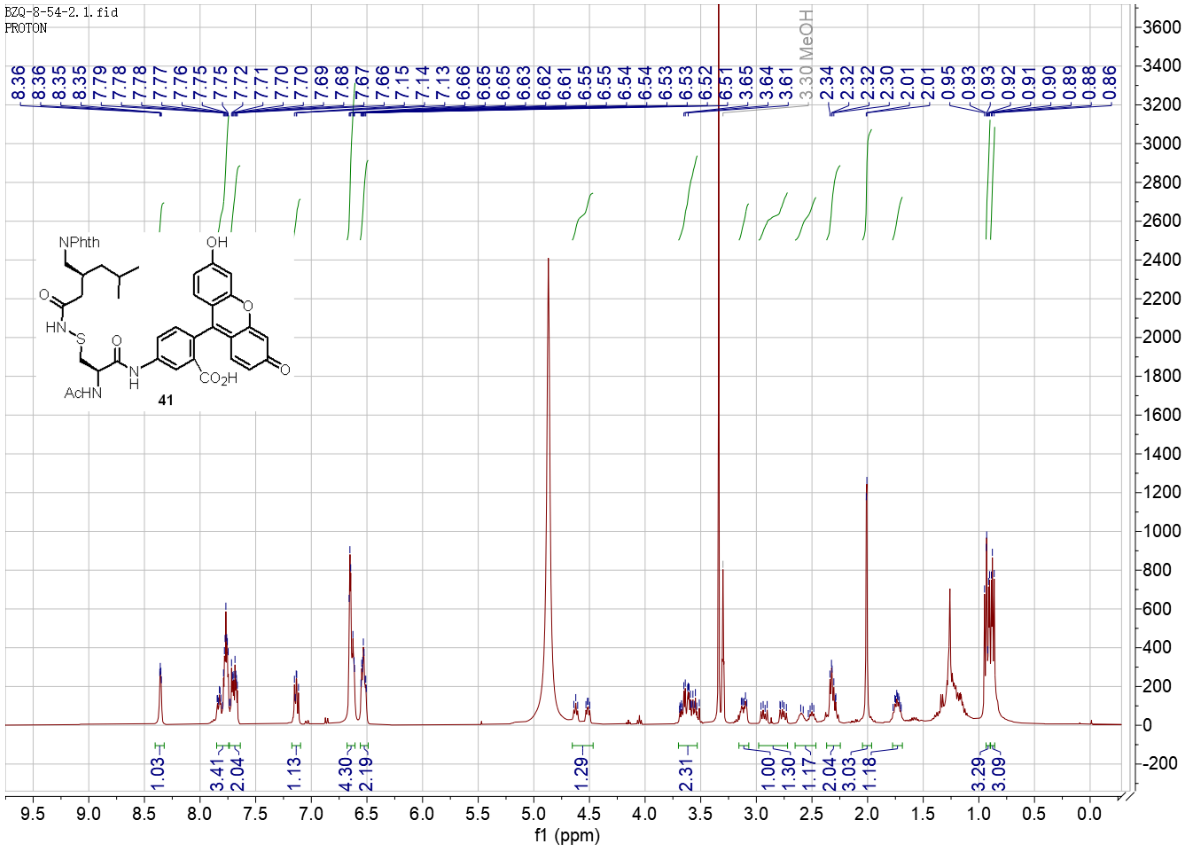

Supplementary Figure 191. ^{1}H NMR (400 MHz, Methanol-d_4, 293 K) spectrum of **41**.

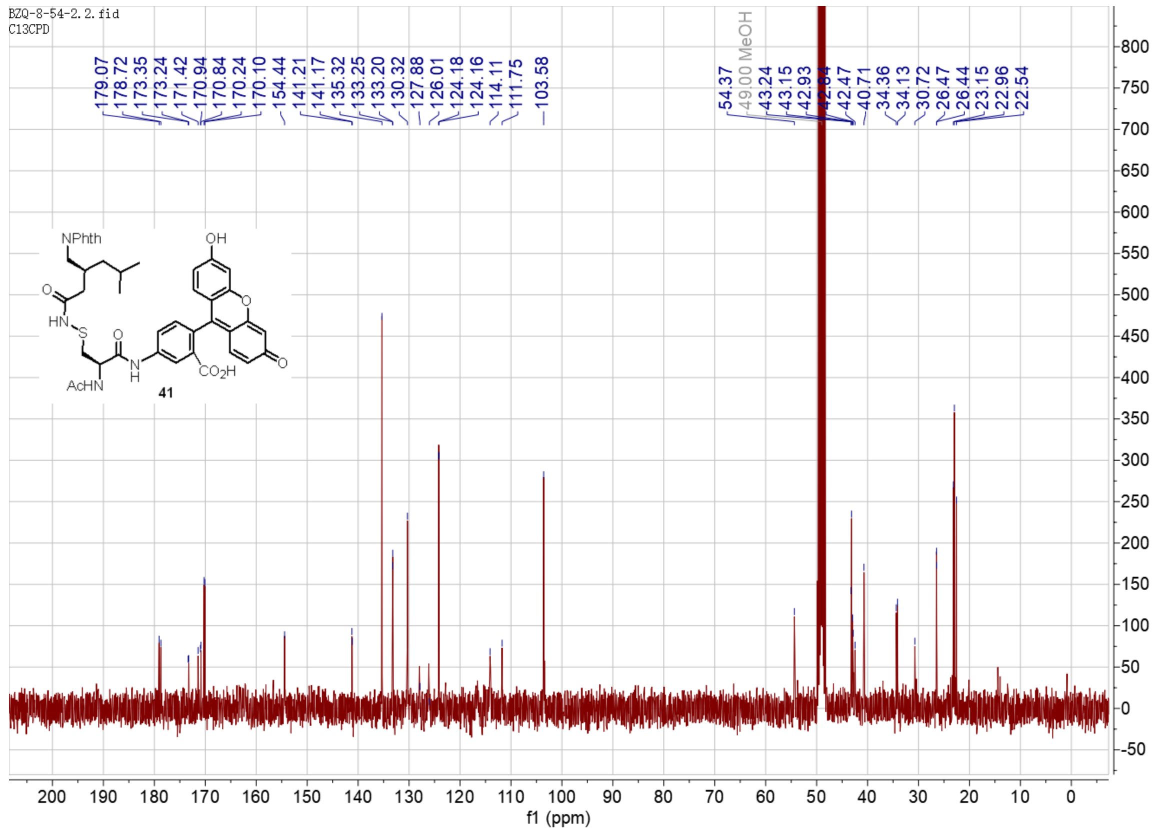

Supplementary Figure 192. ^{13}C NMR (101 MHz, Methanol-d_4, 293 K) spectrum of **41**.

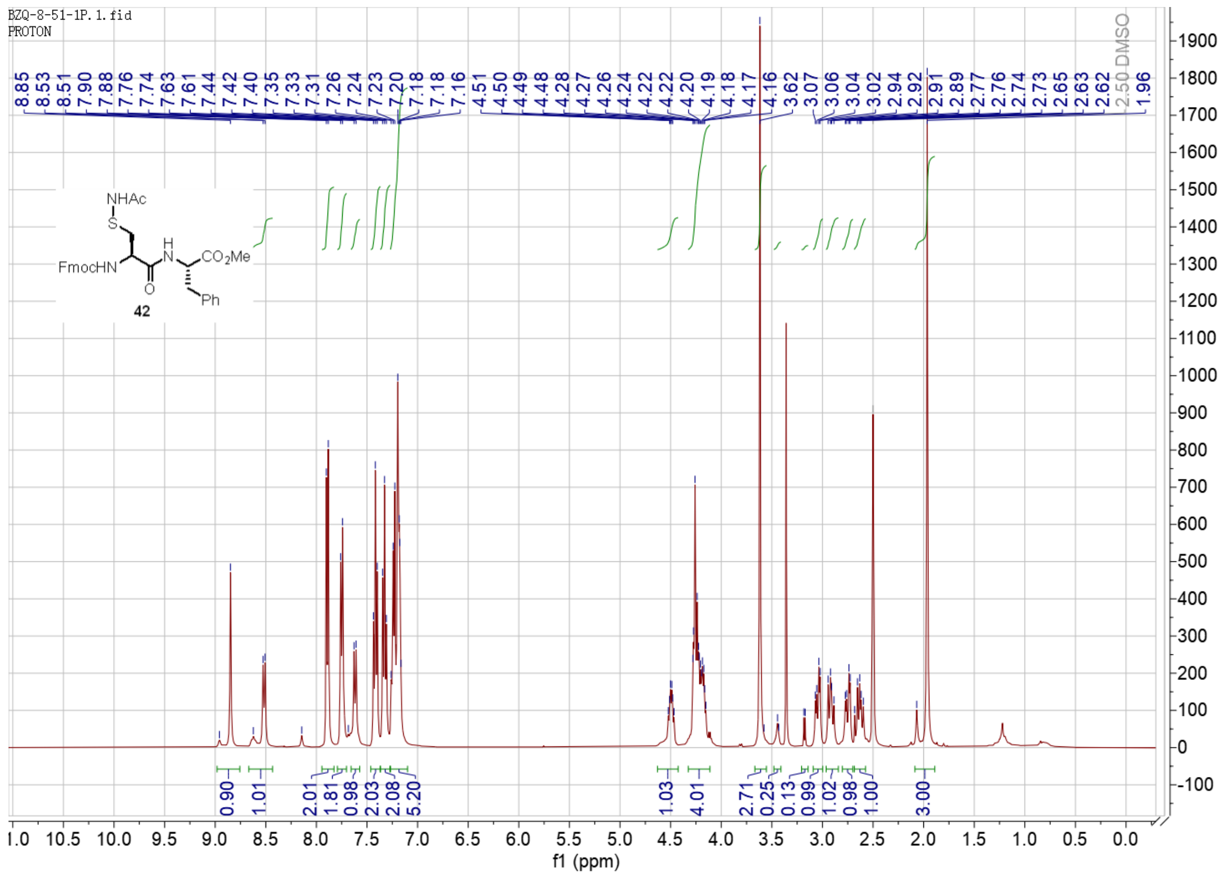

Supplementary Figure 193. ^{1}H NMR (400 MHz, DMSO-d_6, 293 K) spectrum of **42**.

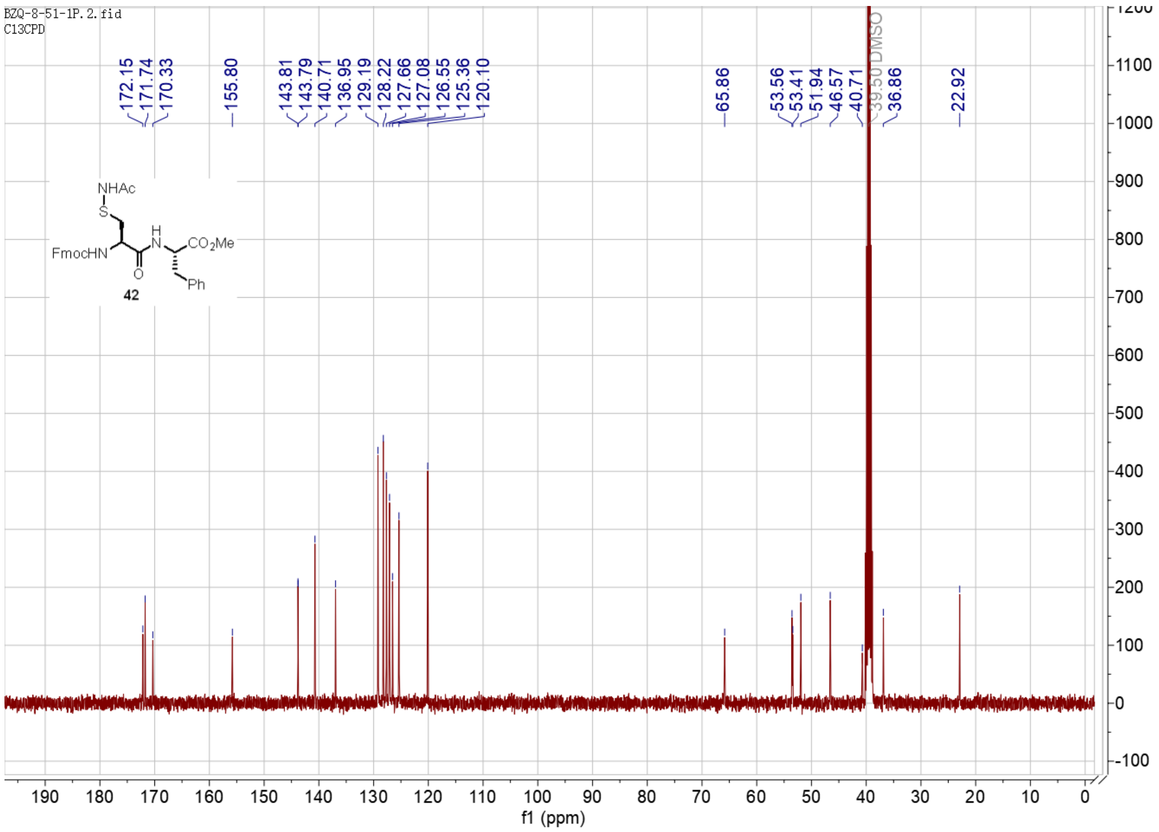

Supplementary Figure 194. ^{13}C NMR (101 MHz, DMSO-d_6, 293 K) spectrum of **42**.

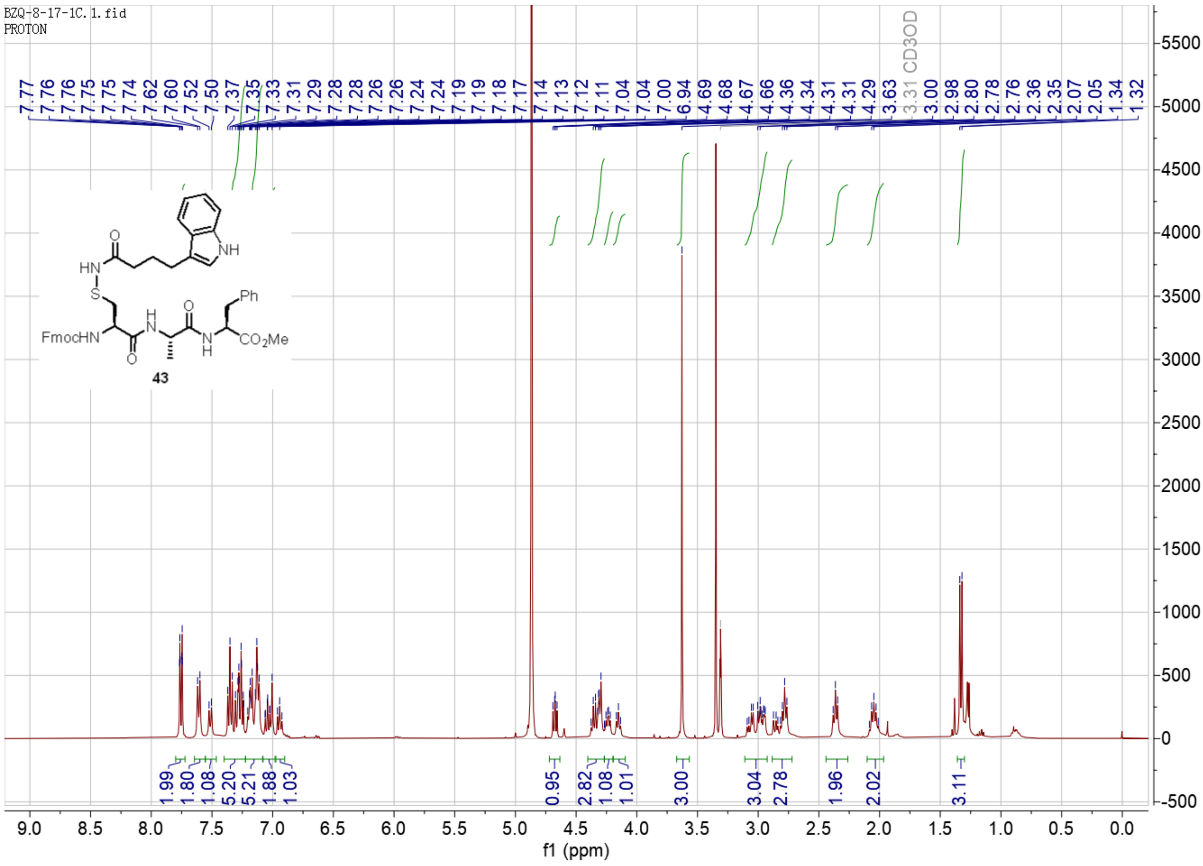

Supplementary Figure 195. ^{1}H NMR (400 MHz, Methanol-d_4, 293 K) spectrum of **43**.

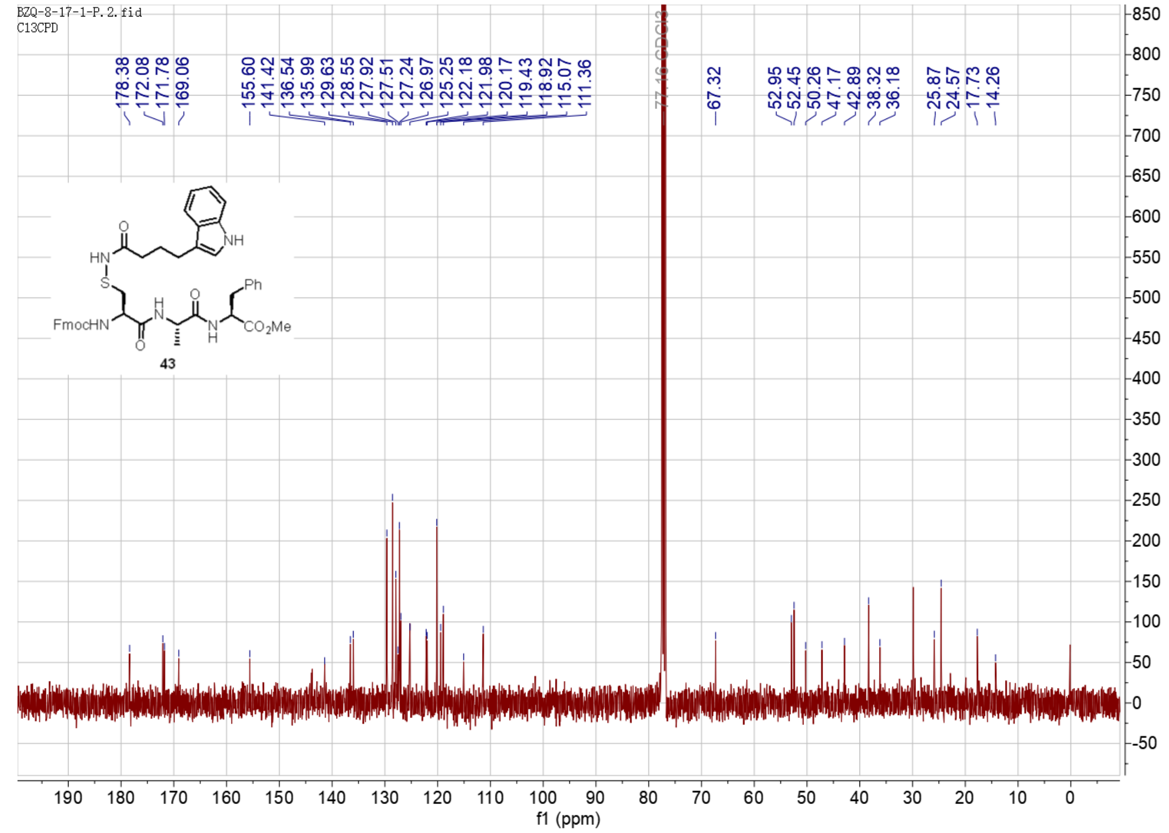

Supplementary Figure 196. ^{13}C NMR (101 MHz, $CDCl_3$, 293 K) spectrum of **43**.

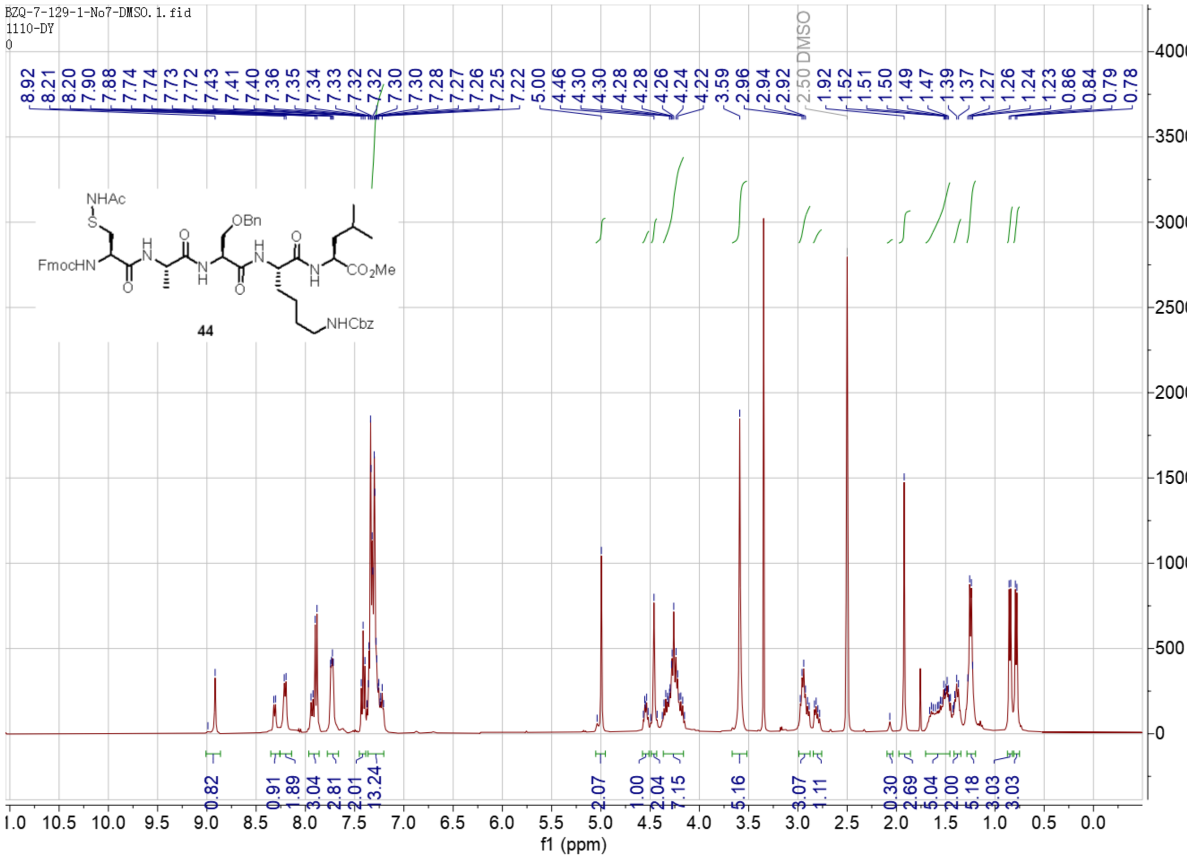

Supplementary Figure 197. ^{1}H NMR (400 MHz, DMSO-d_6, 293 K) spectrum of **44**.

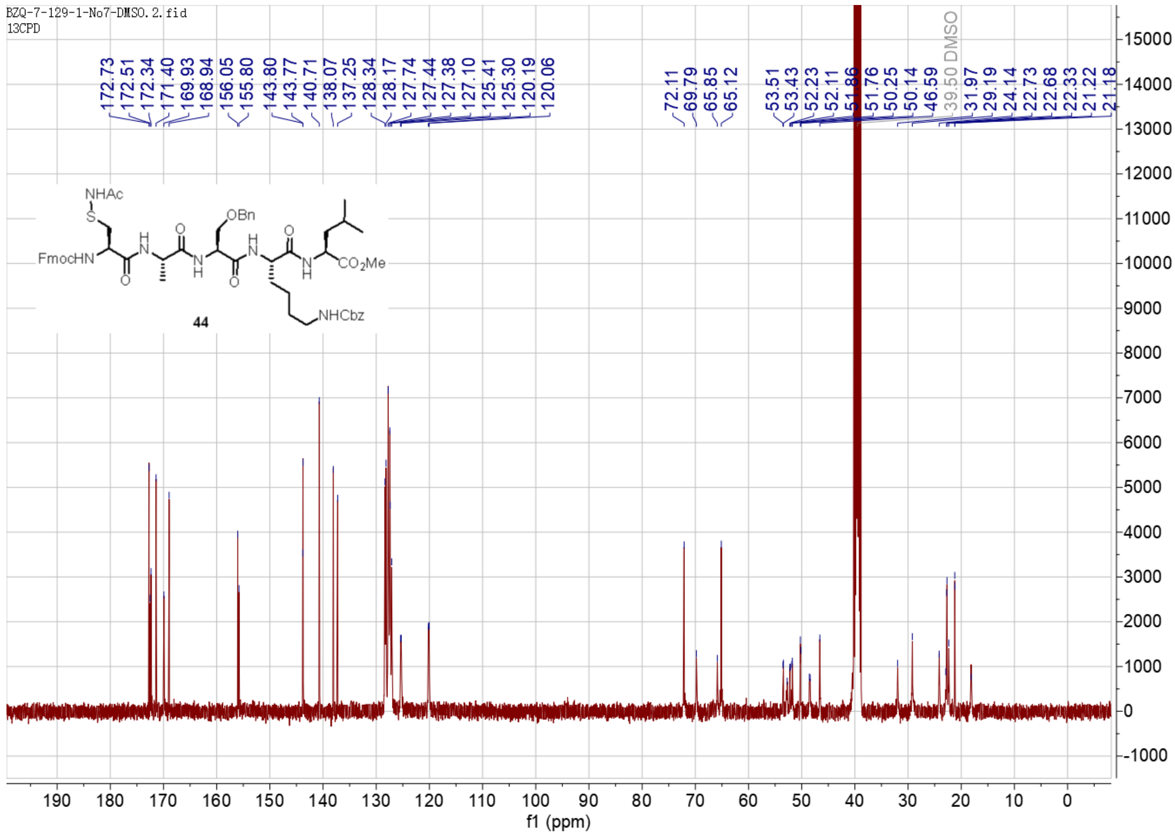

Supplementary Figure 198. ^{13}C NMR (101 MHz, DMSO-d_6, 293 K) spectrum of **44**.

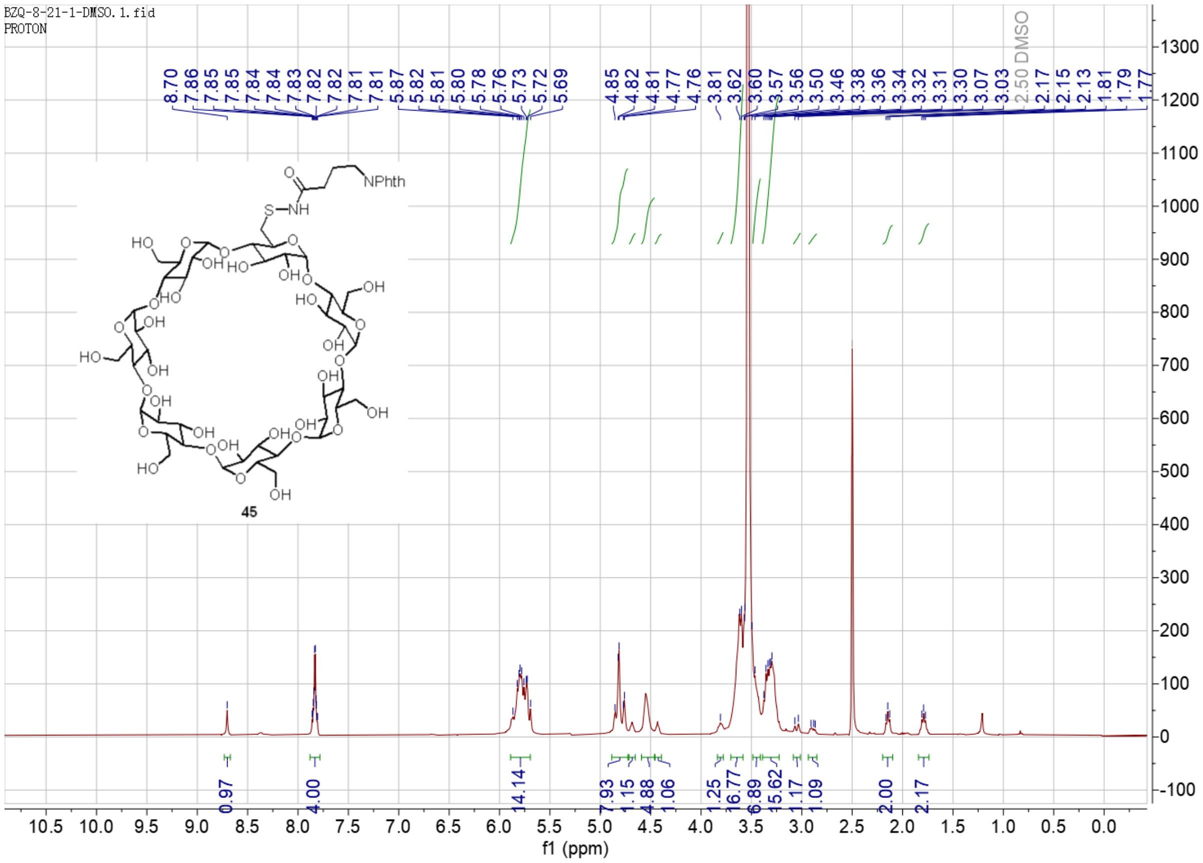

Supplementary Figure 199. ^{1}H NMR (400 MHz, DMSO-d_6, 293 K) spectrum of **45**.

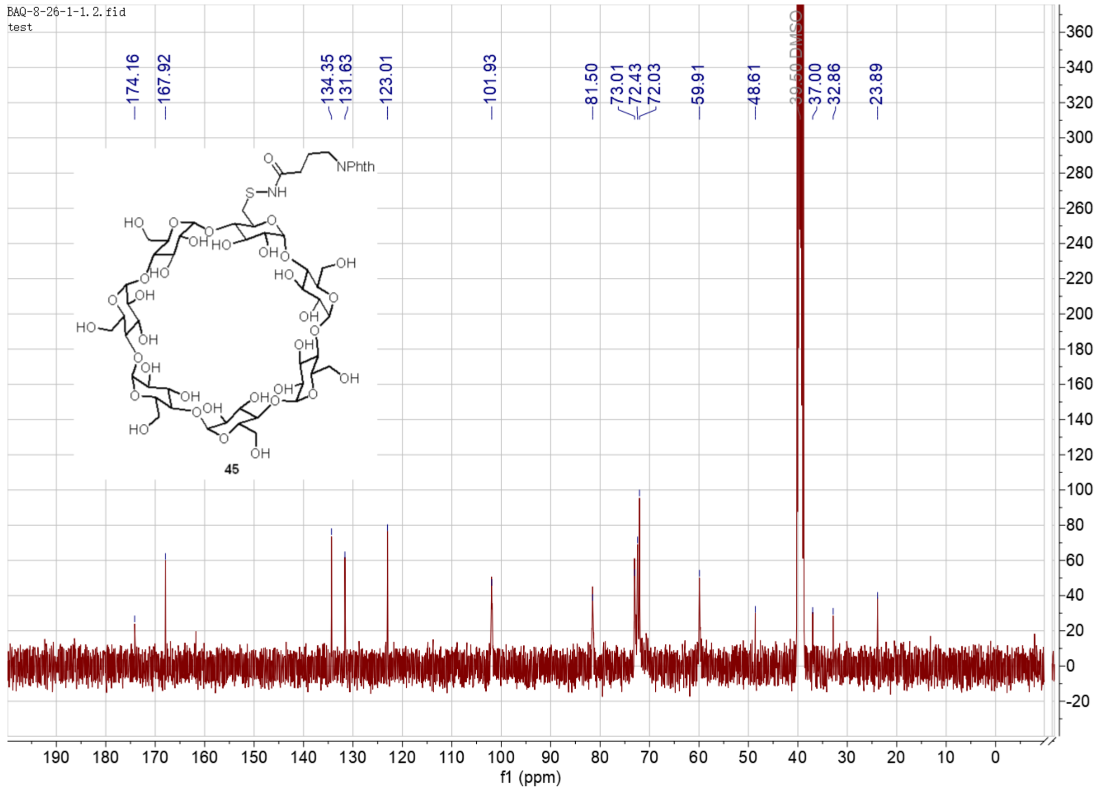

Supplementary Figure 200. ^{13}C NMR (101 MHz, DMSO-d_6, 293 K) spectrum of **45**.

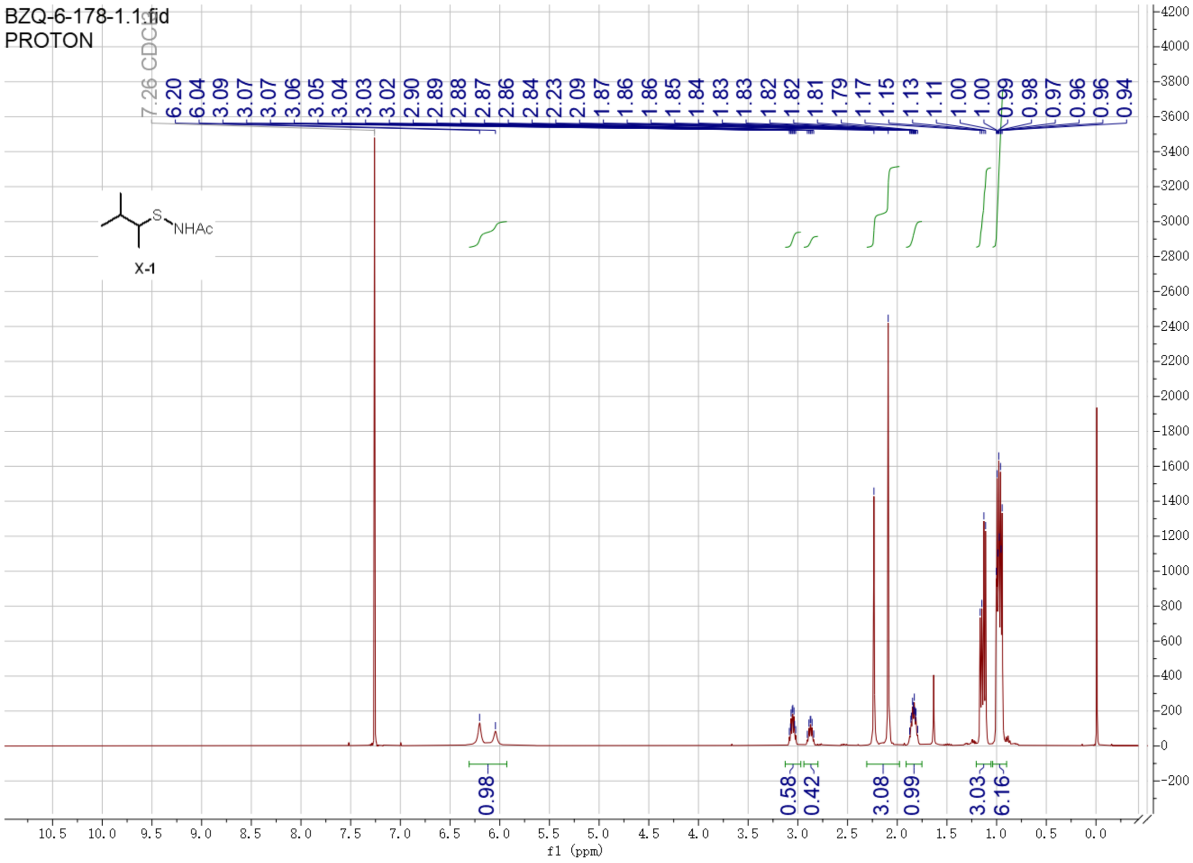

Supplementary Figure 201. ^{1}H NMR (400 MHz, $CDCl_3$, 293 K) spectrum of **X-1**.

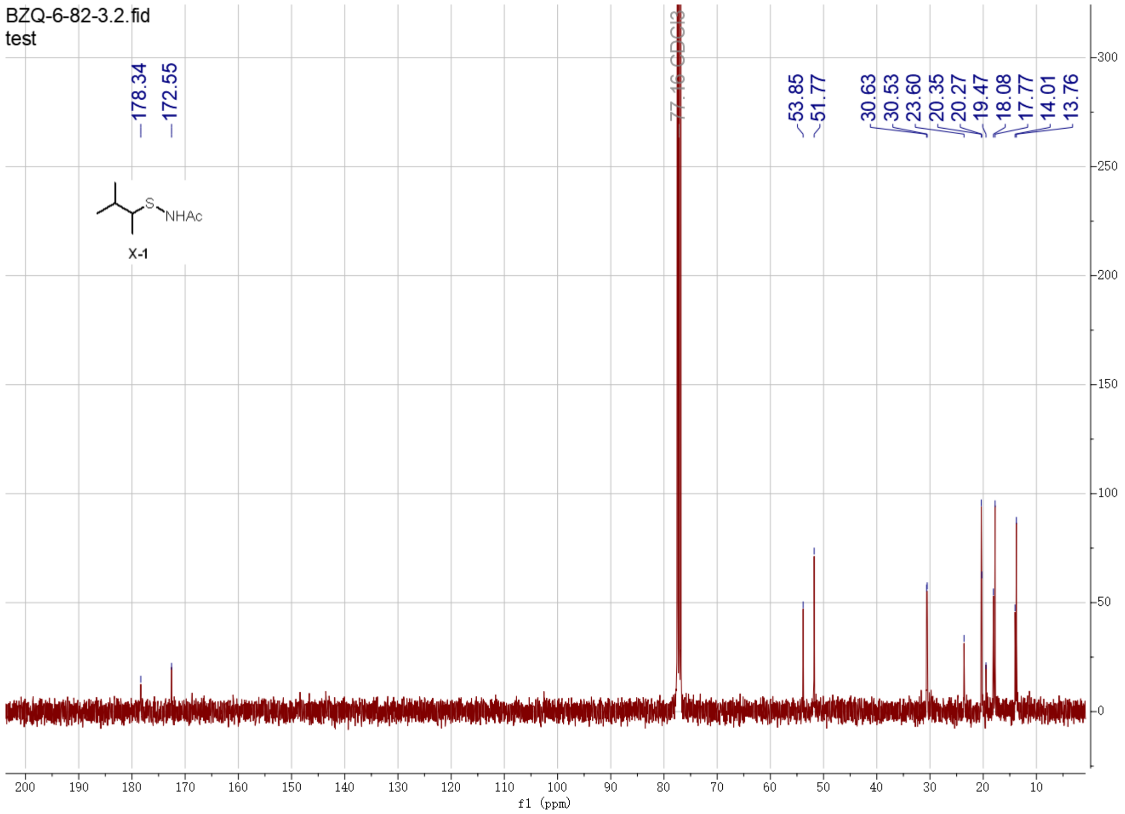

Supplementary Figure 202. ^{13}C NMR (101 MHz, $CDCl_3$, 293 K) spectrum of **X-1**.

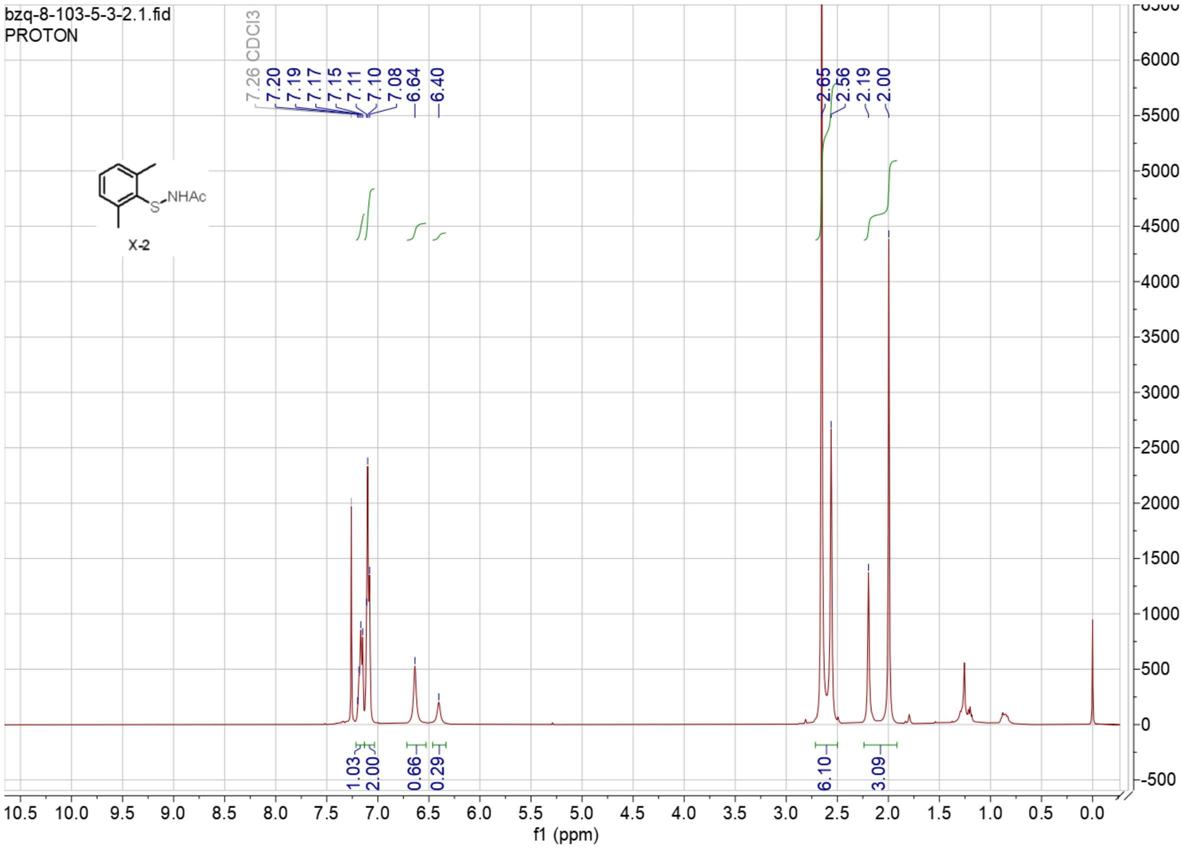

Supplementary Figure 203. ^{1}H NMR (400 MHz, $CDCl_3$, 293 K) spectrum of **X-2**.

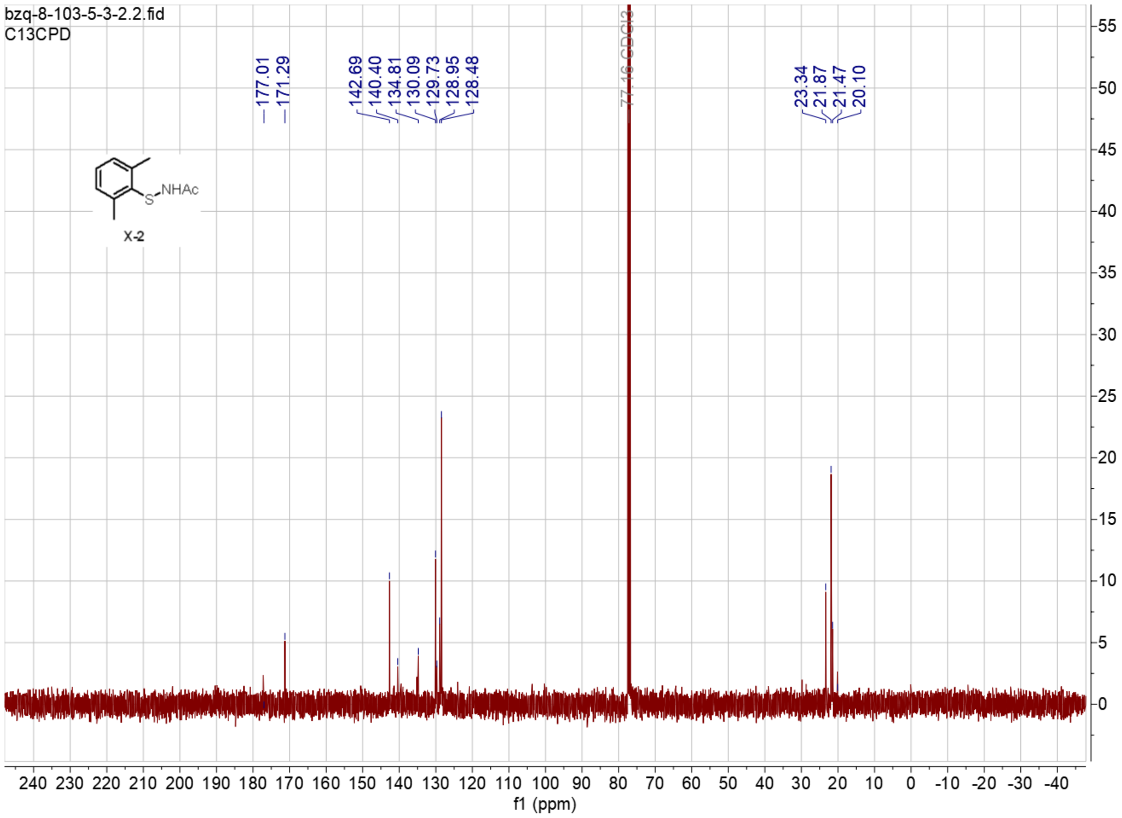

Supplementary Figure 204. ^{13}C NMR (101 MHz, $CDCl_3$, 293 K) spectrum of **X-2**.

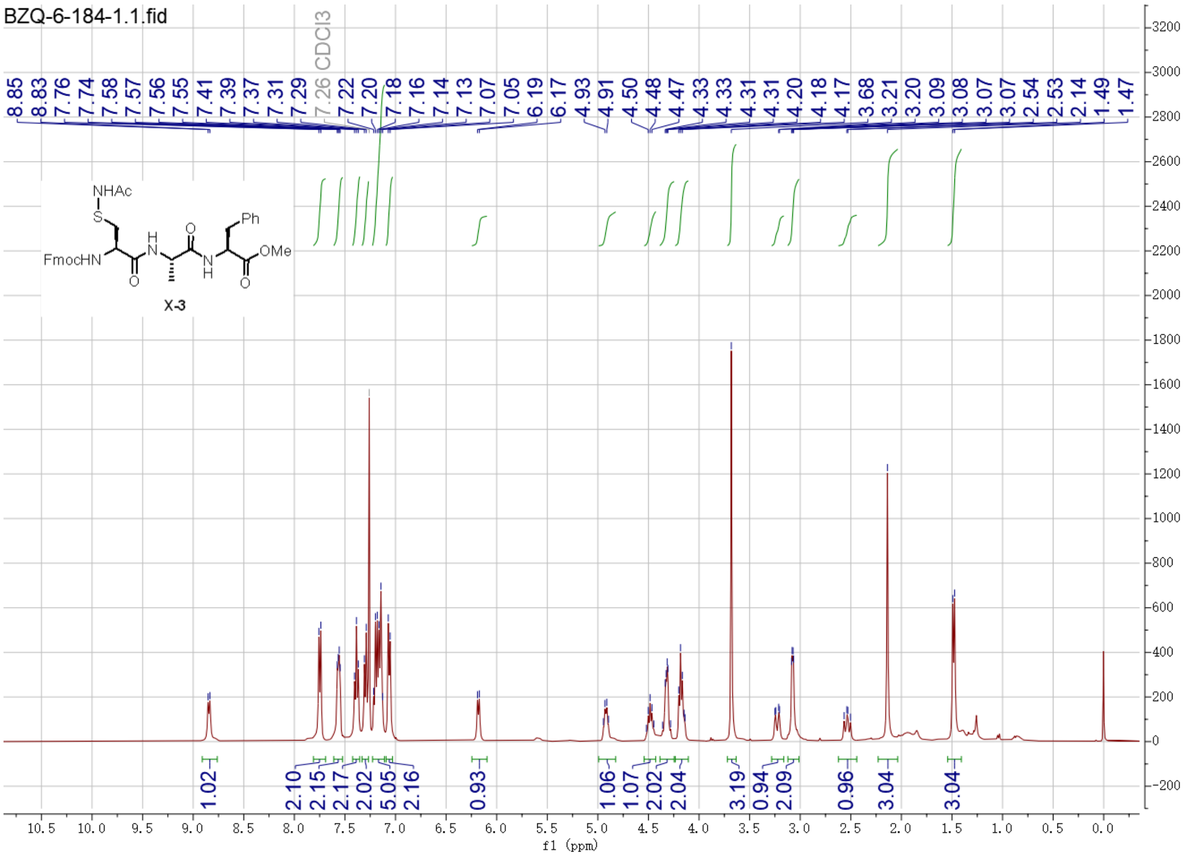

Supplementary Figure 205. ^{1}H NMR (400 MHz, $CDCl_3$, 293 K) spectrum of **X-3**.

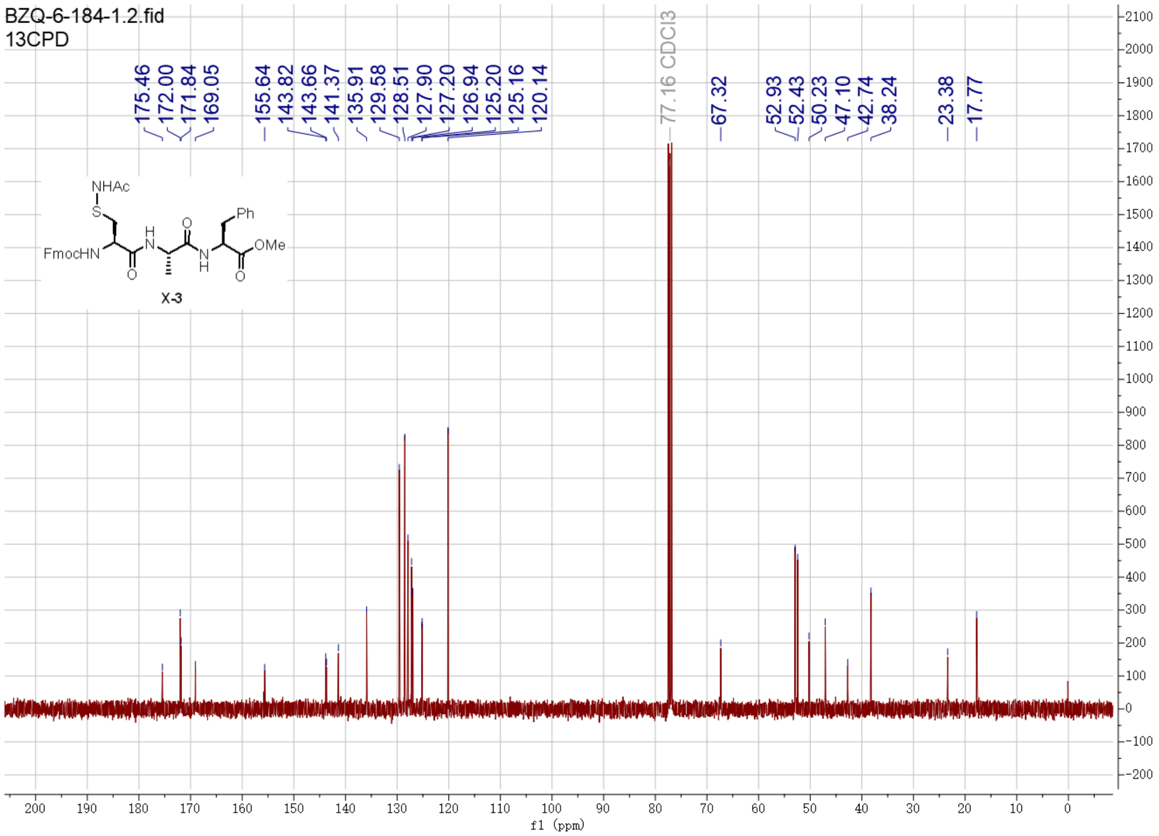

Supplementary Figure 206. ^{13}C NMR (101 MHz, $CDCl_3$, 293 K) spectrum of **X-3**.

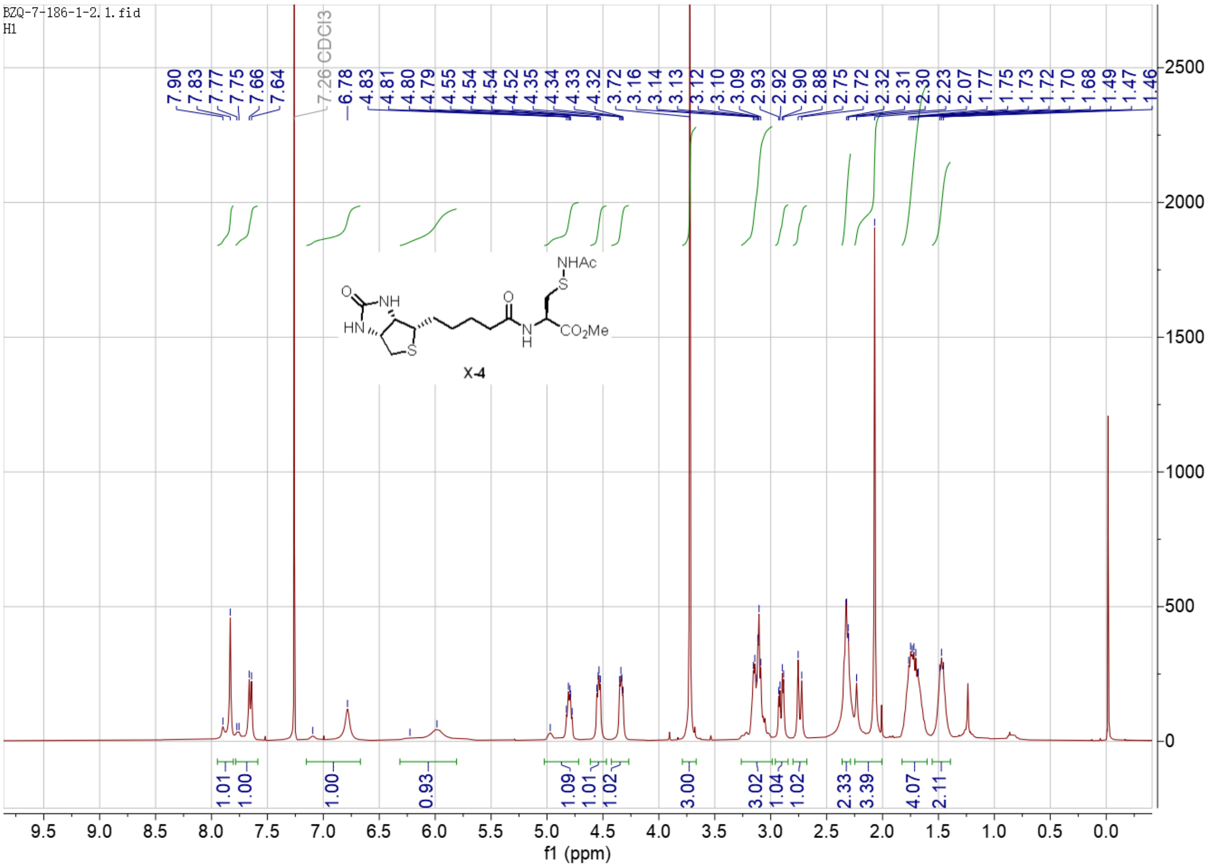

Supplementary Figure 207. ^{1}H NMR (400 MHz, $CDCl_3$, 293 K) spectrum of **X-4**.

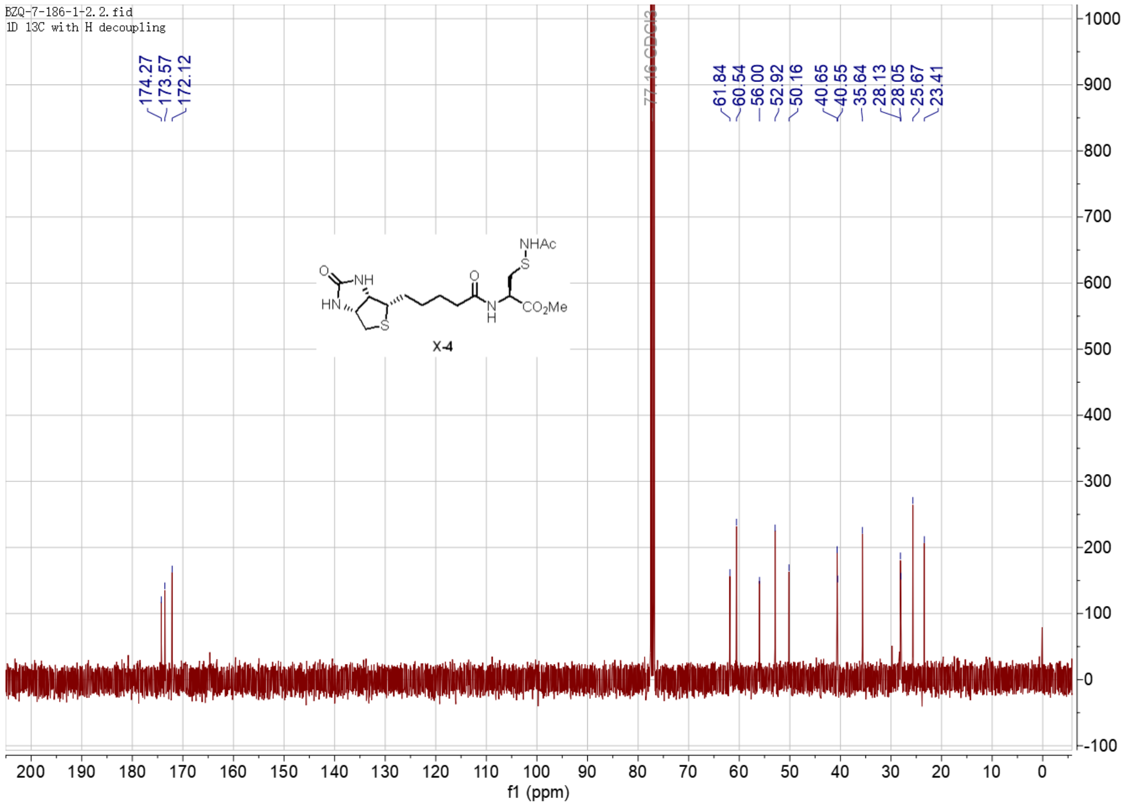

Supplementary Figure 208. ^{13}C NMR (101 MHz, $CDCl_3$, 293 K) spectrum of **X-4**.

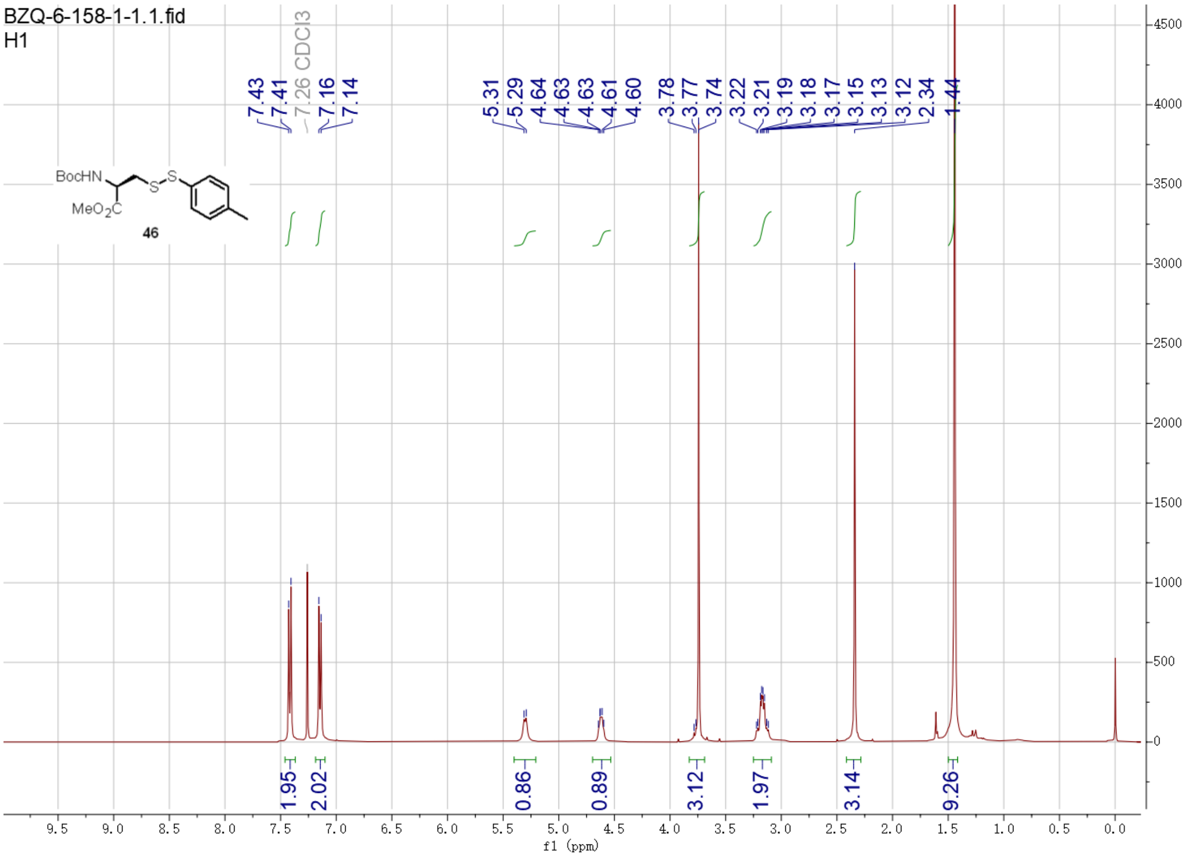

Supplementary Figure 209. ^{1}H NMR (400 MHz, $CDCl_3$, 293 K) spectrum of **46**.

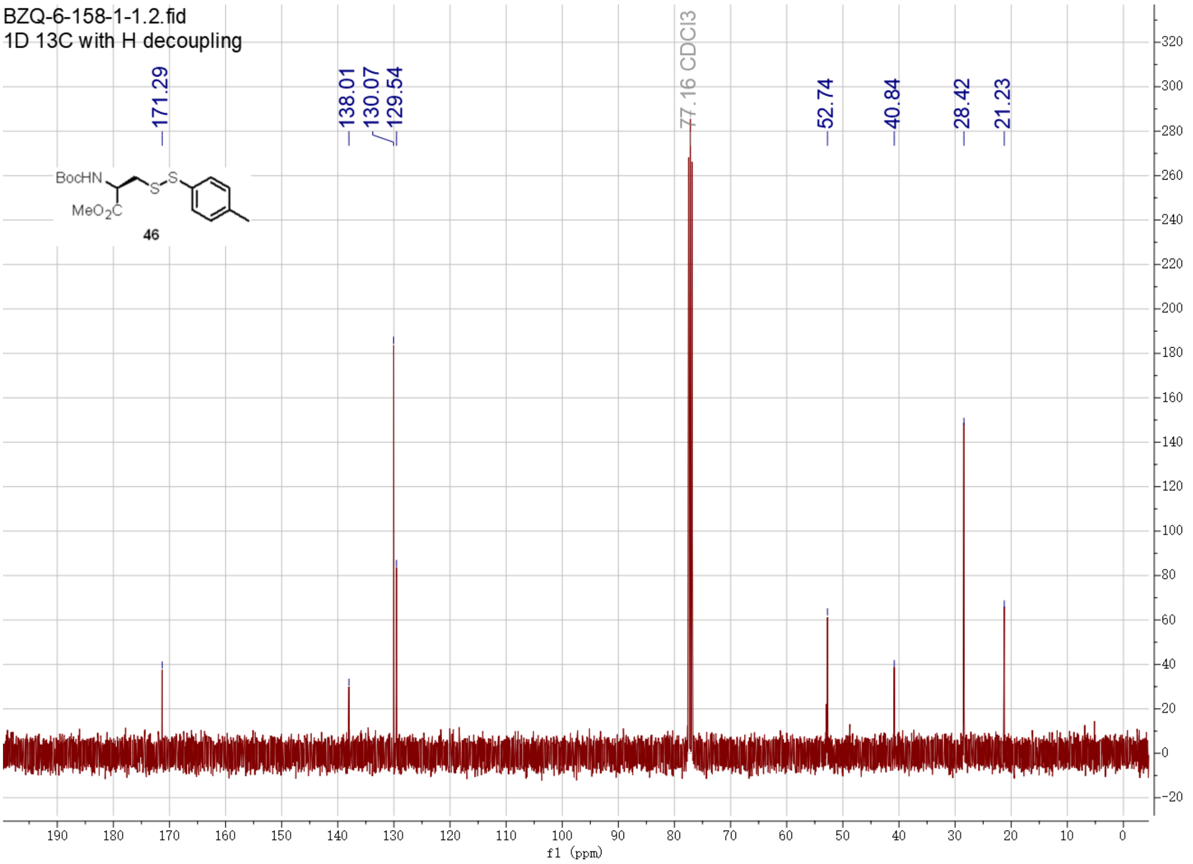

Supplementary Figure 210. ^{13}C NMR (101 MHz, $CDCl_3$, 293 K) spectrum of **46**.

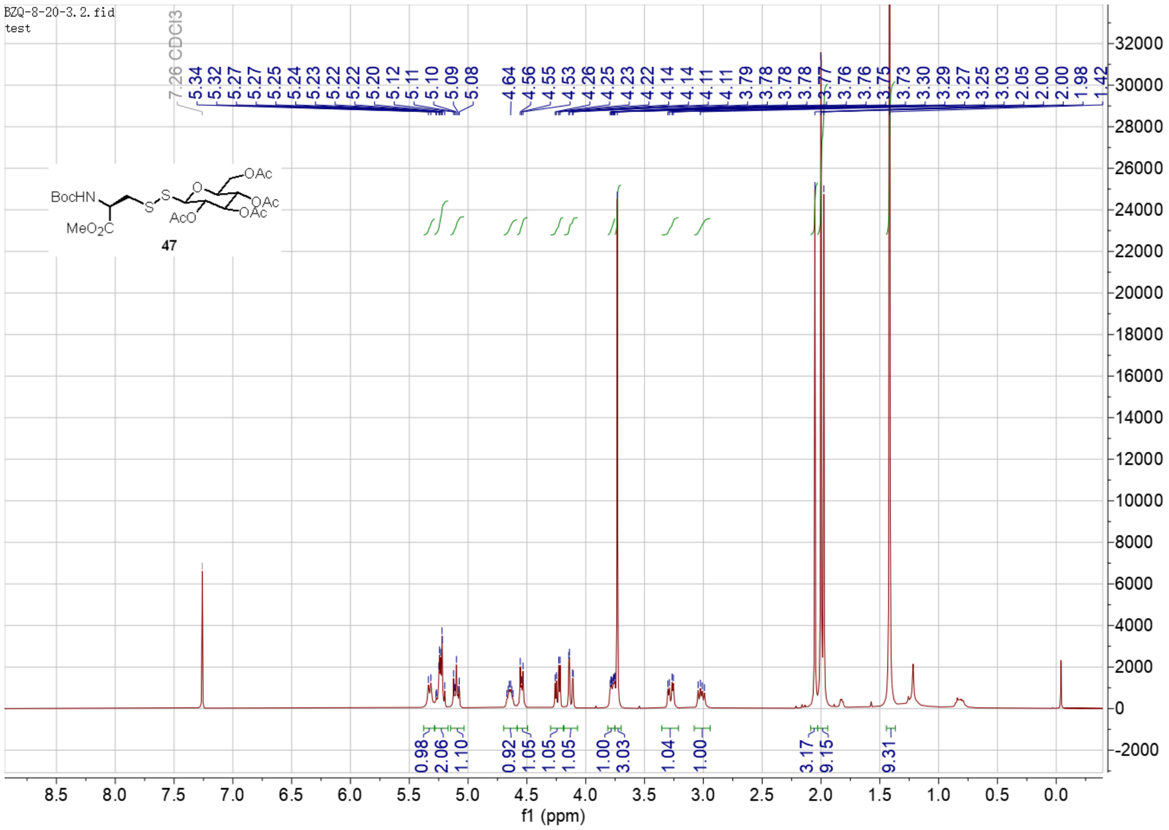

Supplementary Figure 211. ^{1}H NMR (400 MHz, $CDCl_3$, 293 K) spectrum of **47**.

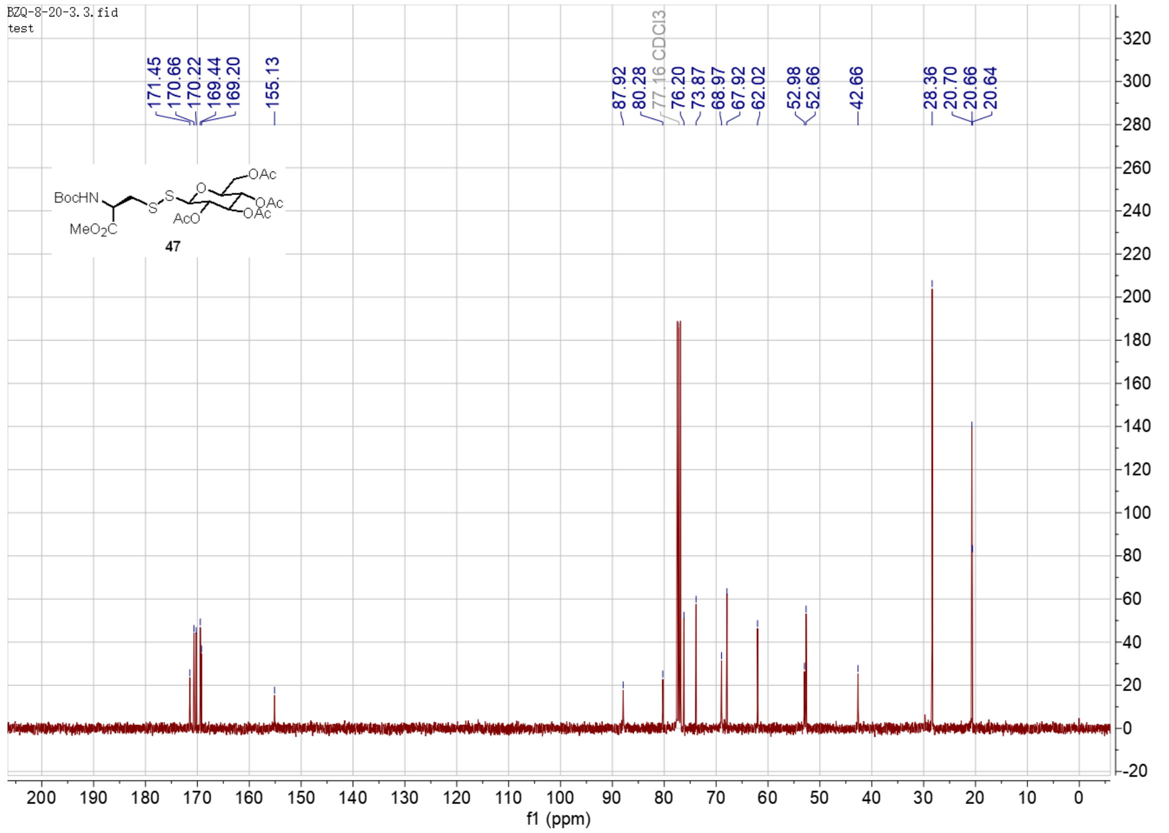

Supplementary Figure 212. ^{13}C NMR (101 MHz, CDCl$_3$, 293 K) spectrum of **47**.

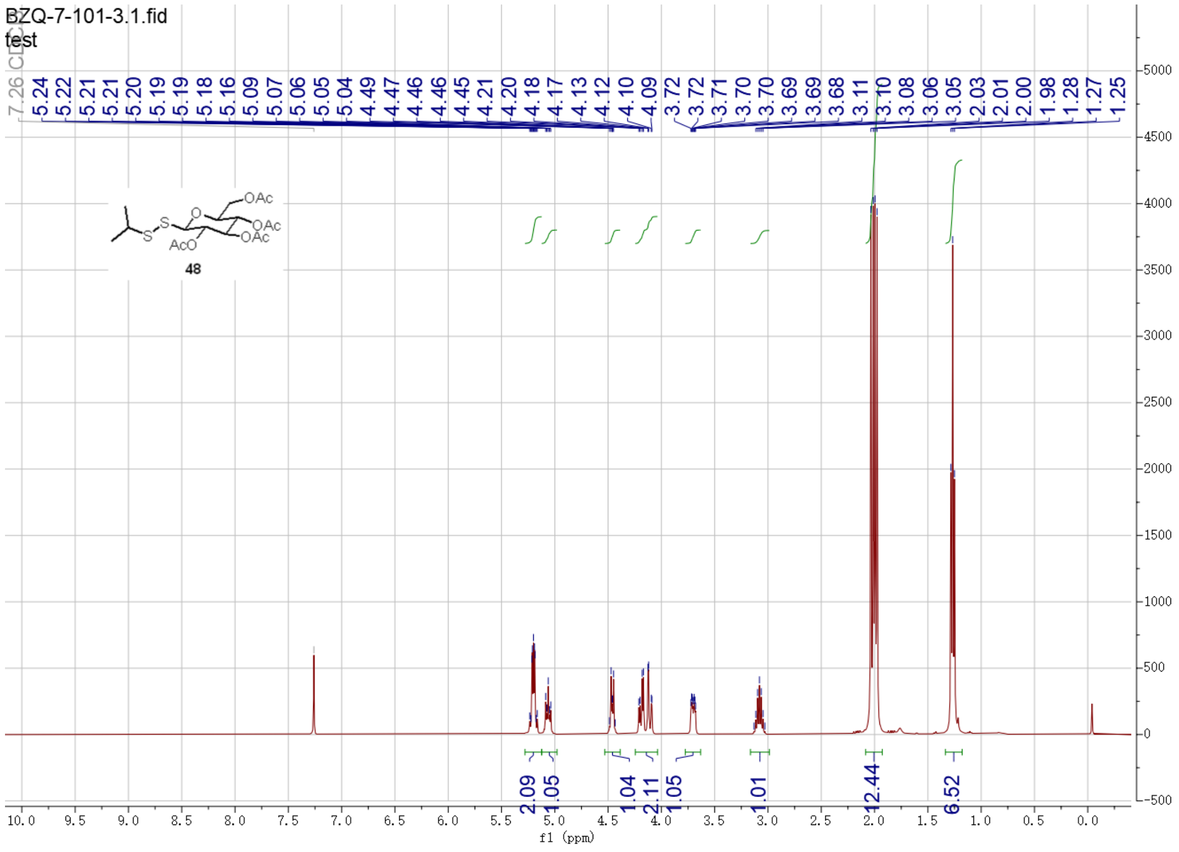

Supplementary Figure 213. ^{1}H NMR (400 MHz, CDCl$_3$, 293 K) spectrum of **48**.

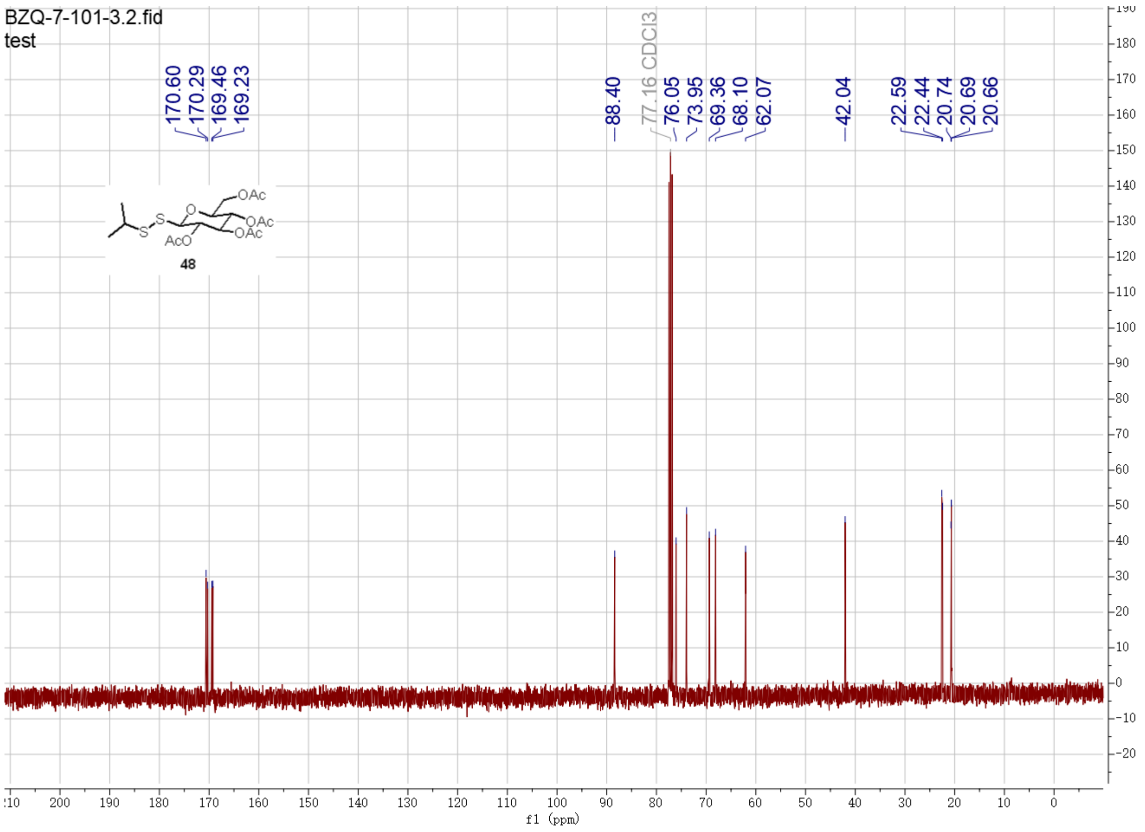

Supplementary Figure 214. ^{13}C NMR (101 MHz, $CDCl_3$, 293 K) spectrum of **48**.

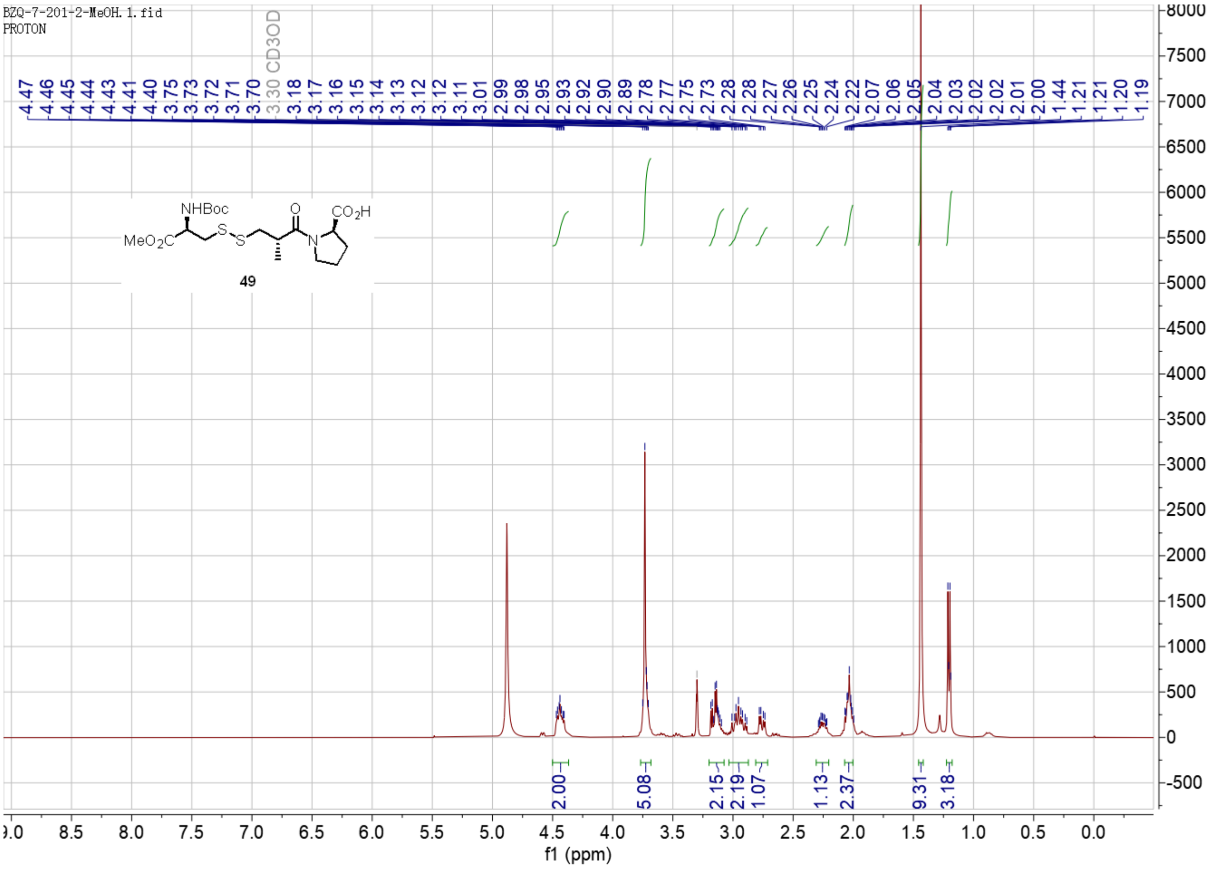

Supplementary Figure 215. ^{1}H NMR (400 MHz, Methanol-d_4, 293 K) spectrum of **49**.

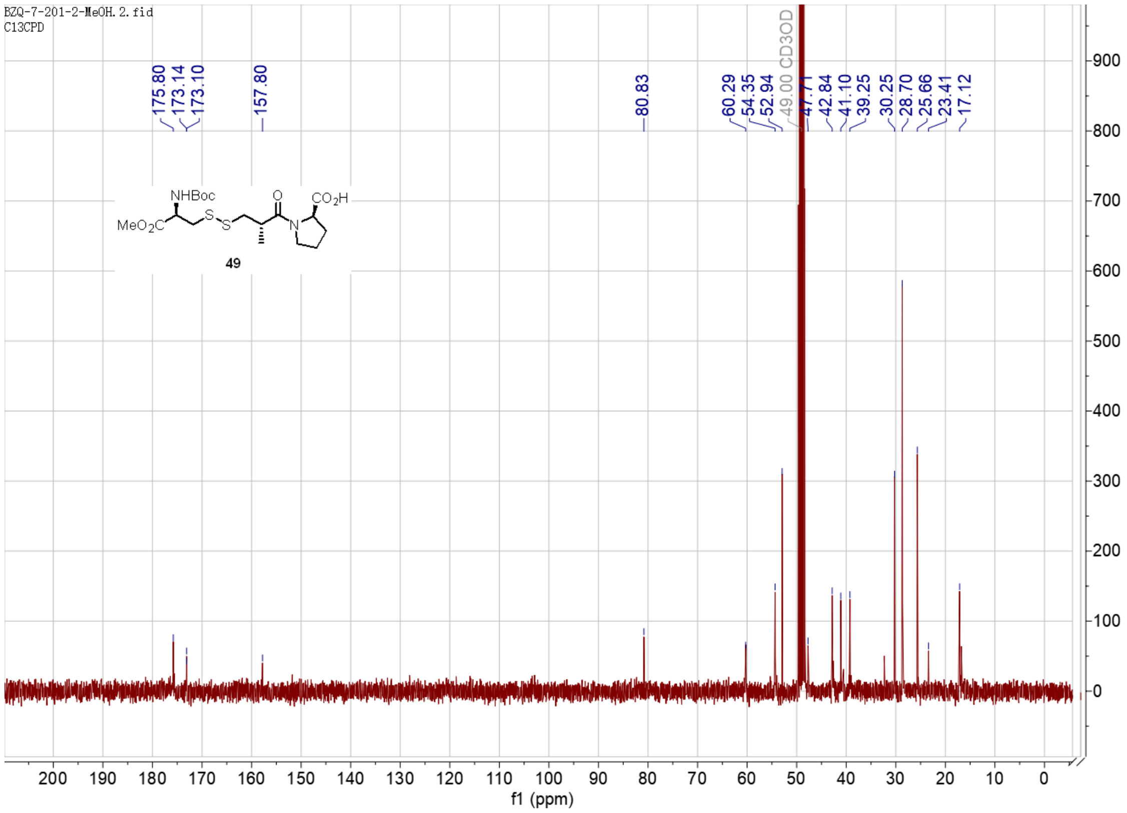

Supplementary Figure 216. ^{13}C NMR (101 MHz, Methanol-d_4, 293 K) spectrum of **49**.

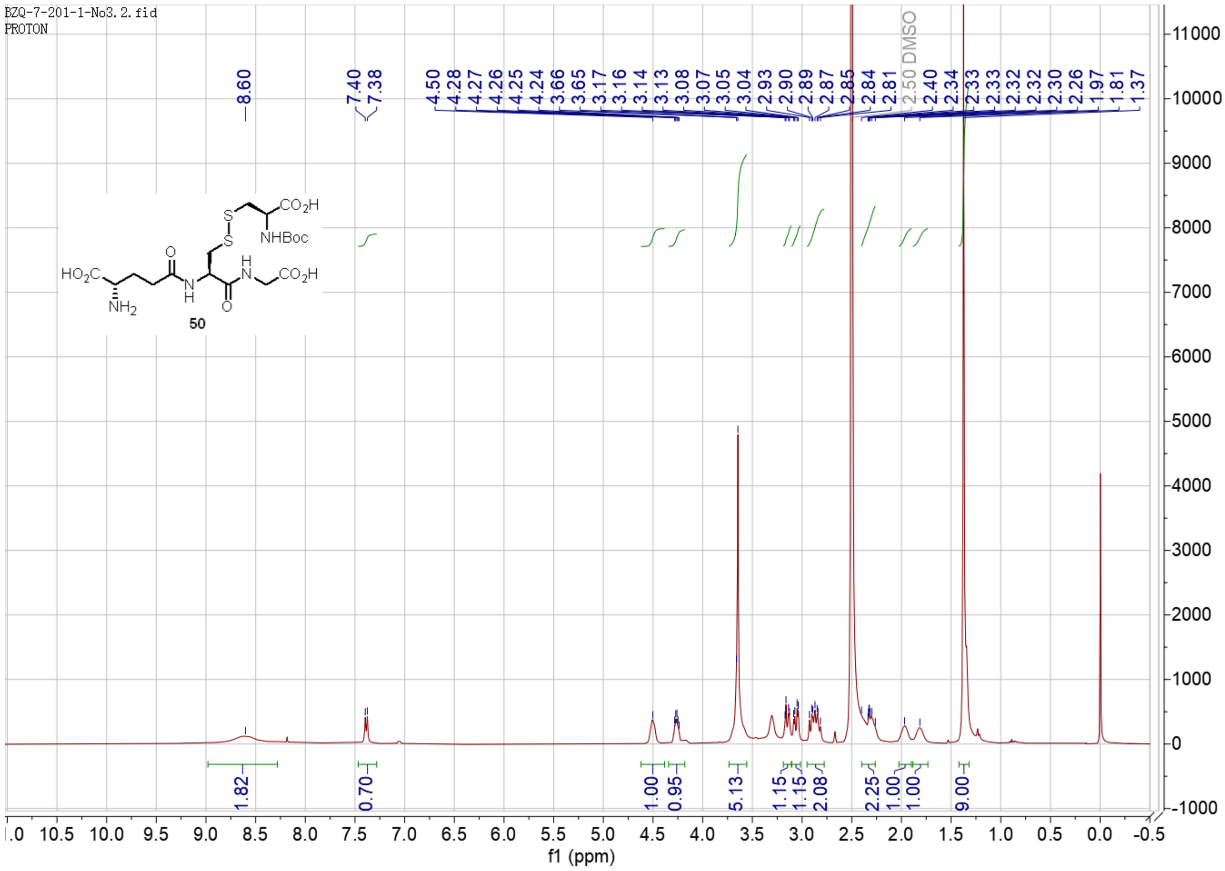

Supplementary Figure 217. ^{1}H NMR (400 MHz, DMSO-d_6, 293 K) spectrum of **50**.

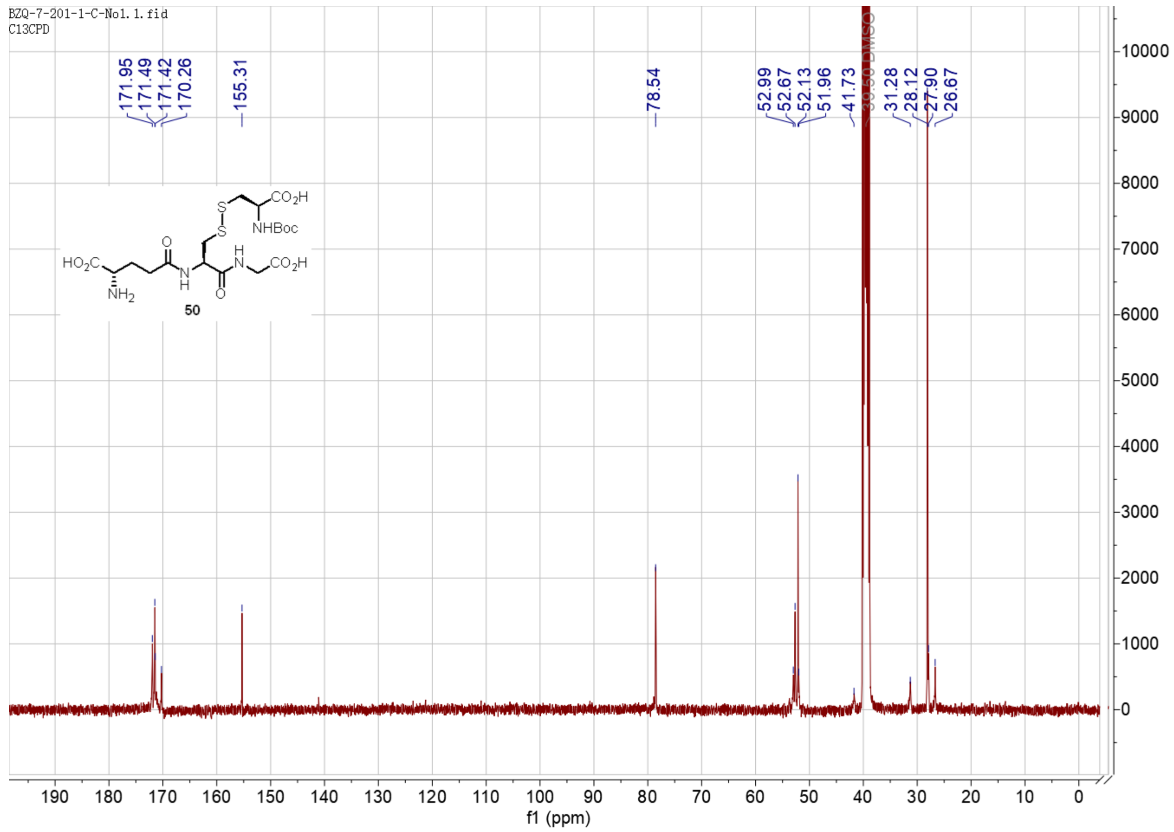

Supplementary Figure 218. ^{13}C NMR (101 MHz, DMSO-d_6, 293 K) spectrum of **50**.

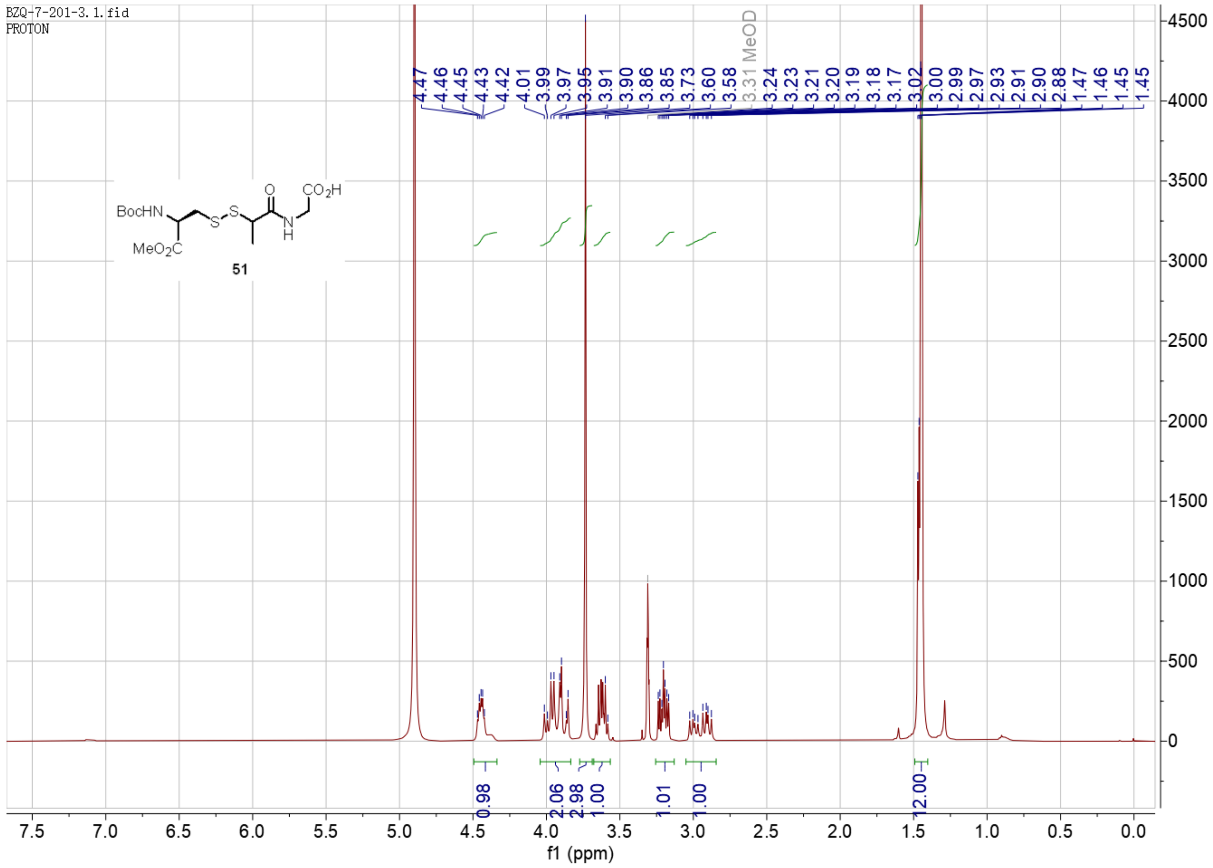

Supplementary Figure 219. ^{1}H NMR (400 MHz, Methanol-d_4, 293 K) spectrum of **51**.

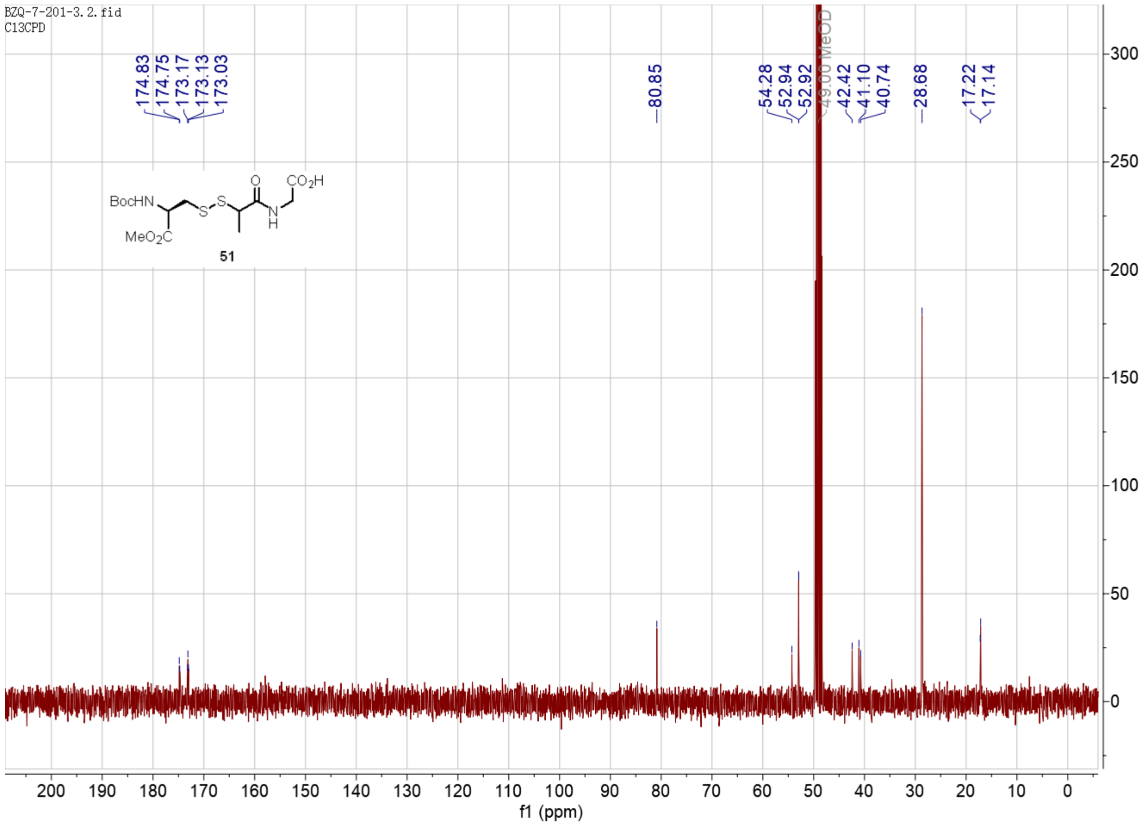

Supplementary Figure 220. ^{13}C NMR (101 MHz, Methanol-d_4, 293 K) spectrum of **51**.

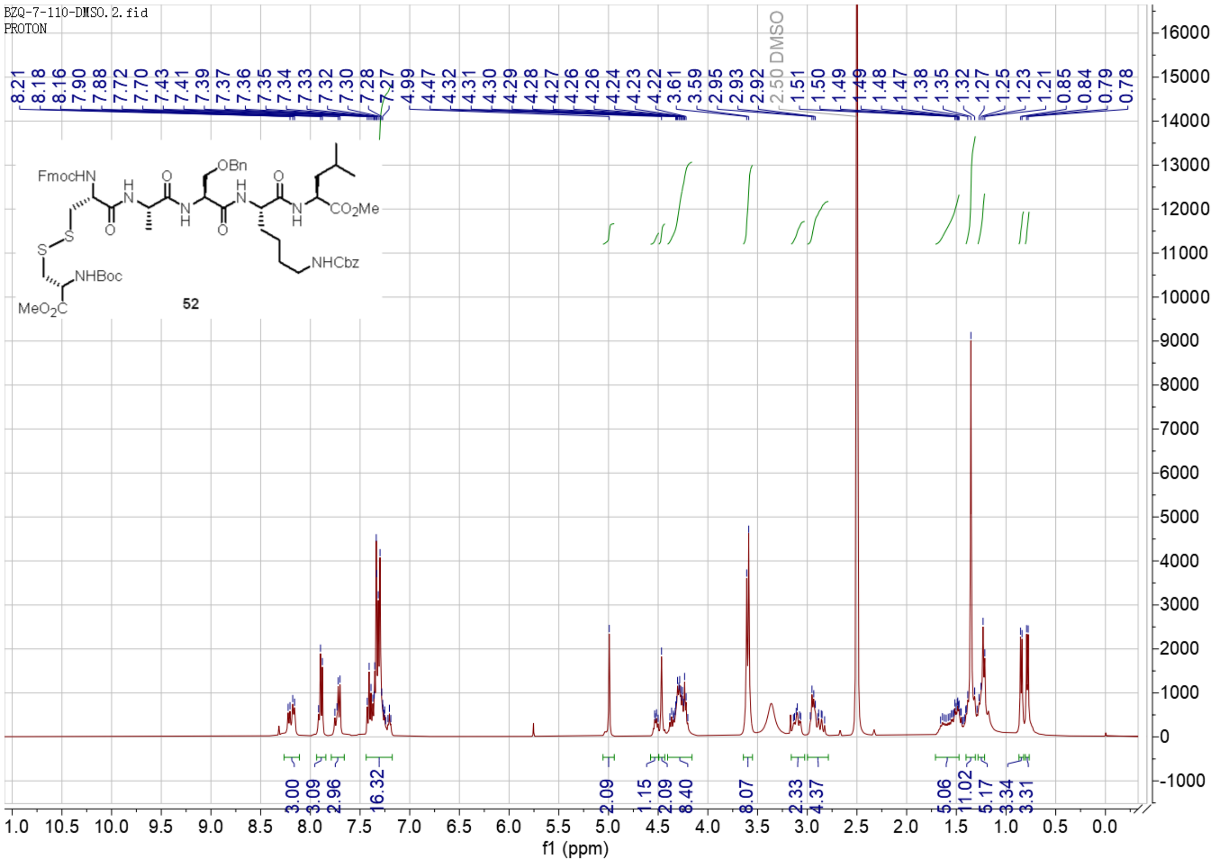

Supplementary Figure 221. ^{1}H NMR (400 MHz, DMSO-d_6, 293 K) spectrum of **52**.

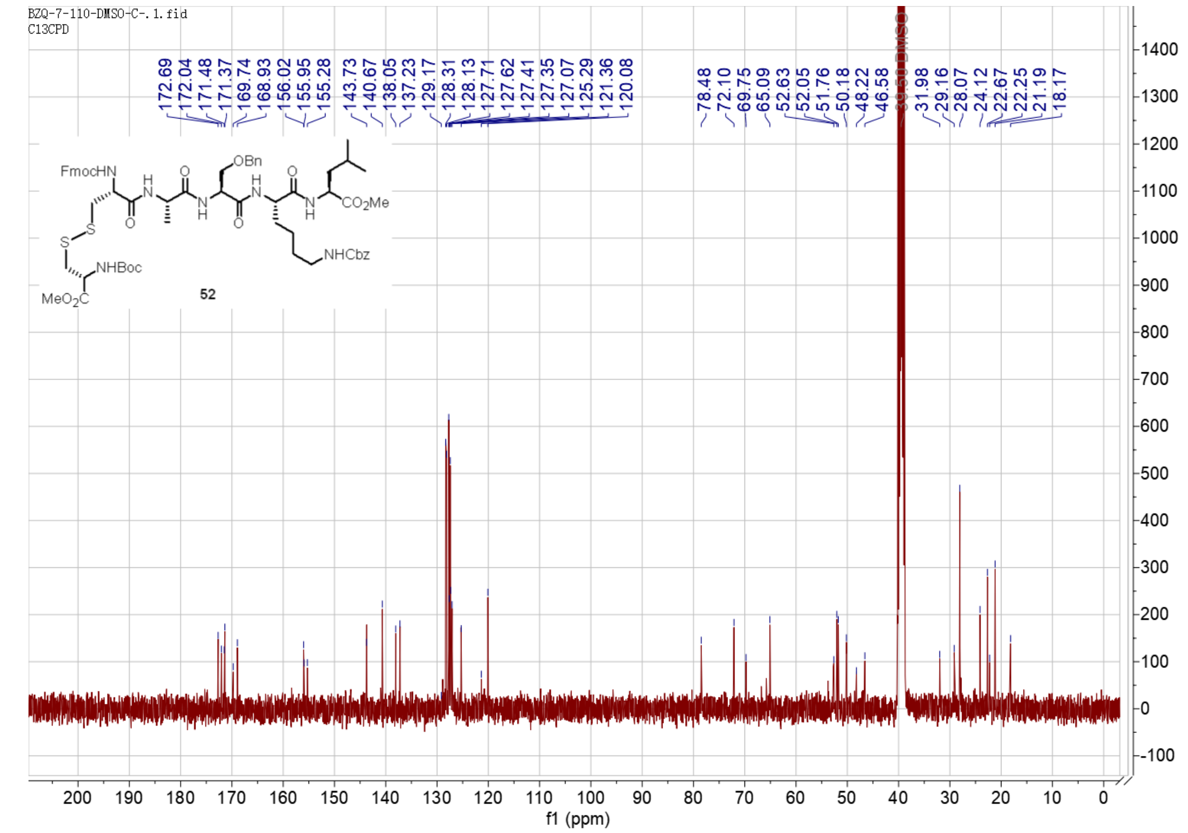

Supplementary Figure 222. ^{13}C NMR (101 MHz, DMSO-d_6, 293 K) spectrum of **52**.

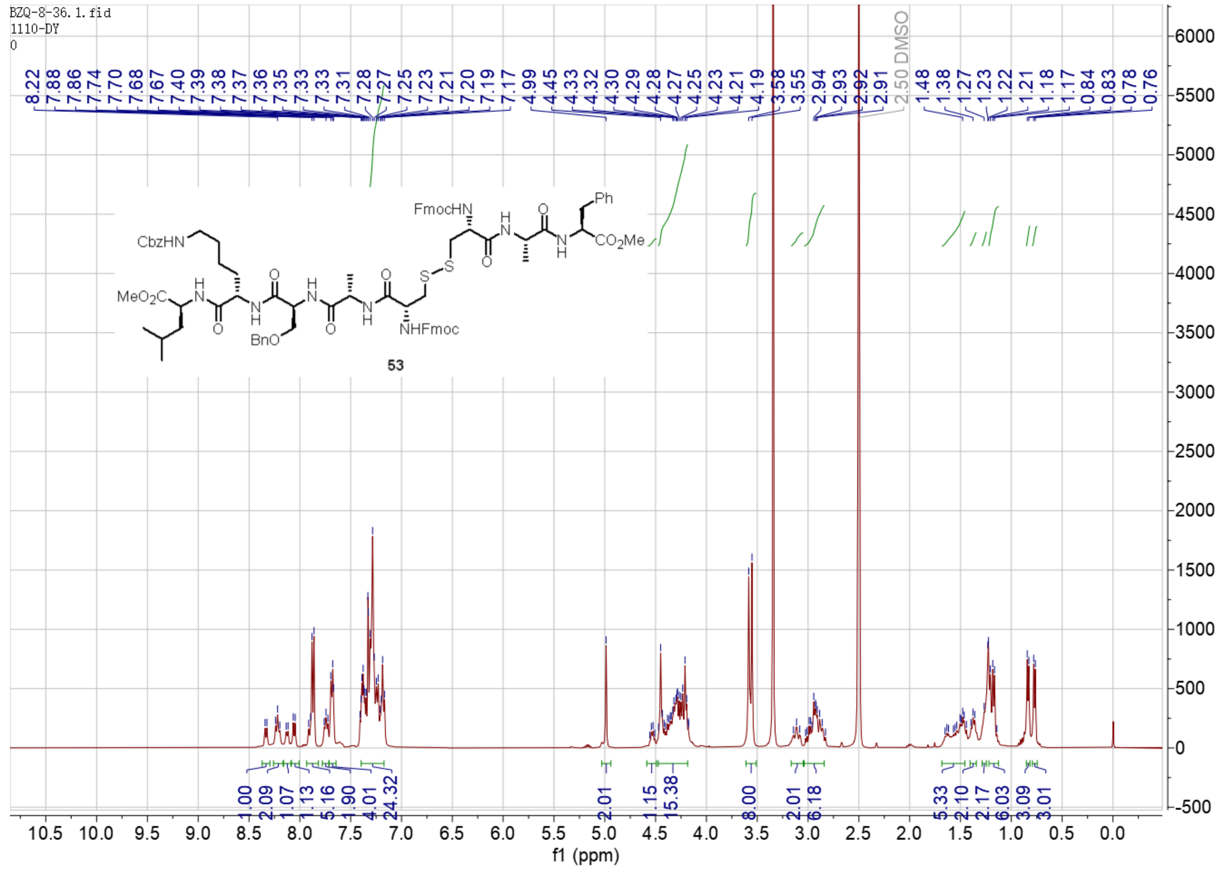

Supplementary Figure 223. ^{1}H NMR (400 MHz, DMSO-d_6, 293 K) spectrum of **53**.

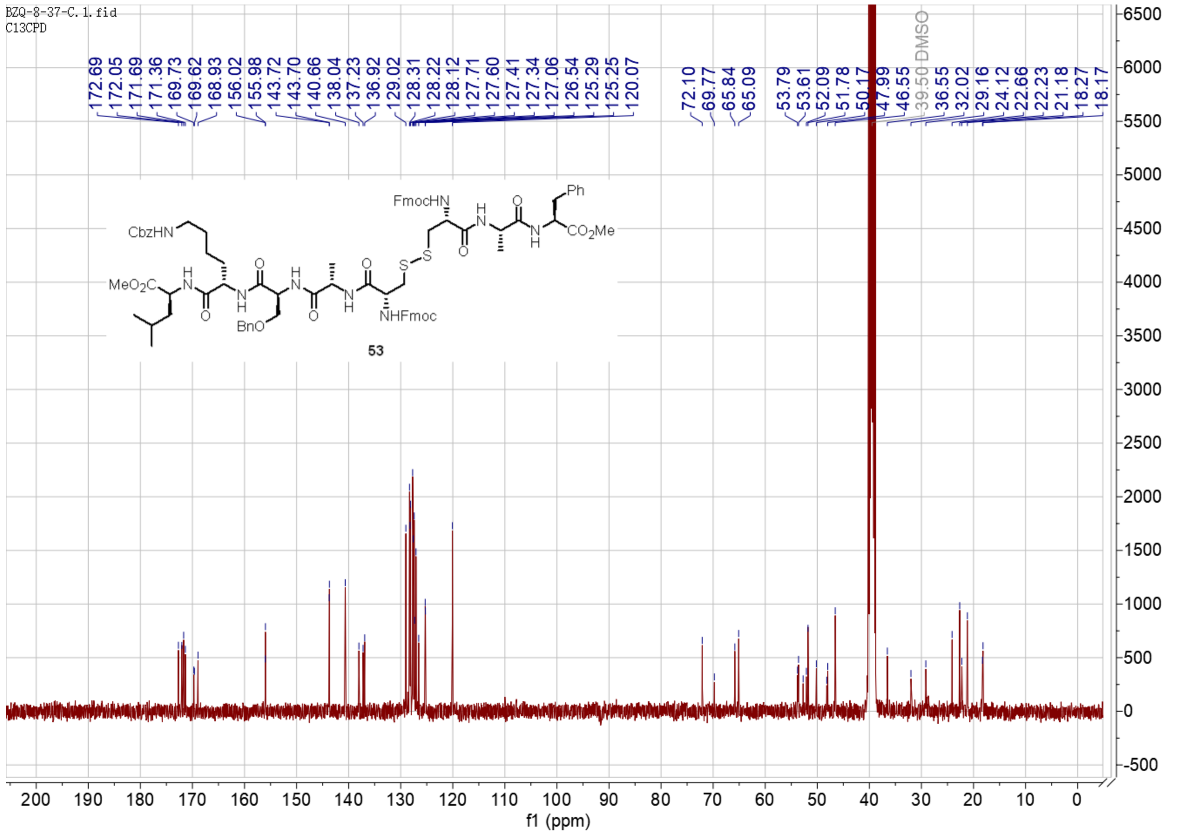

Supplementary Figure 224. ^{13}C NMR (101 MHz, DMSO-d_6, 293 K) spectrum of **53**.

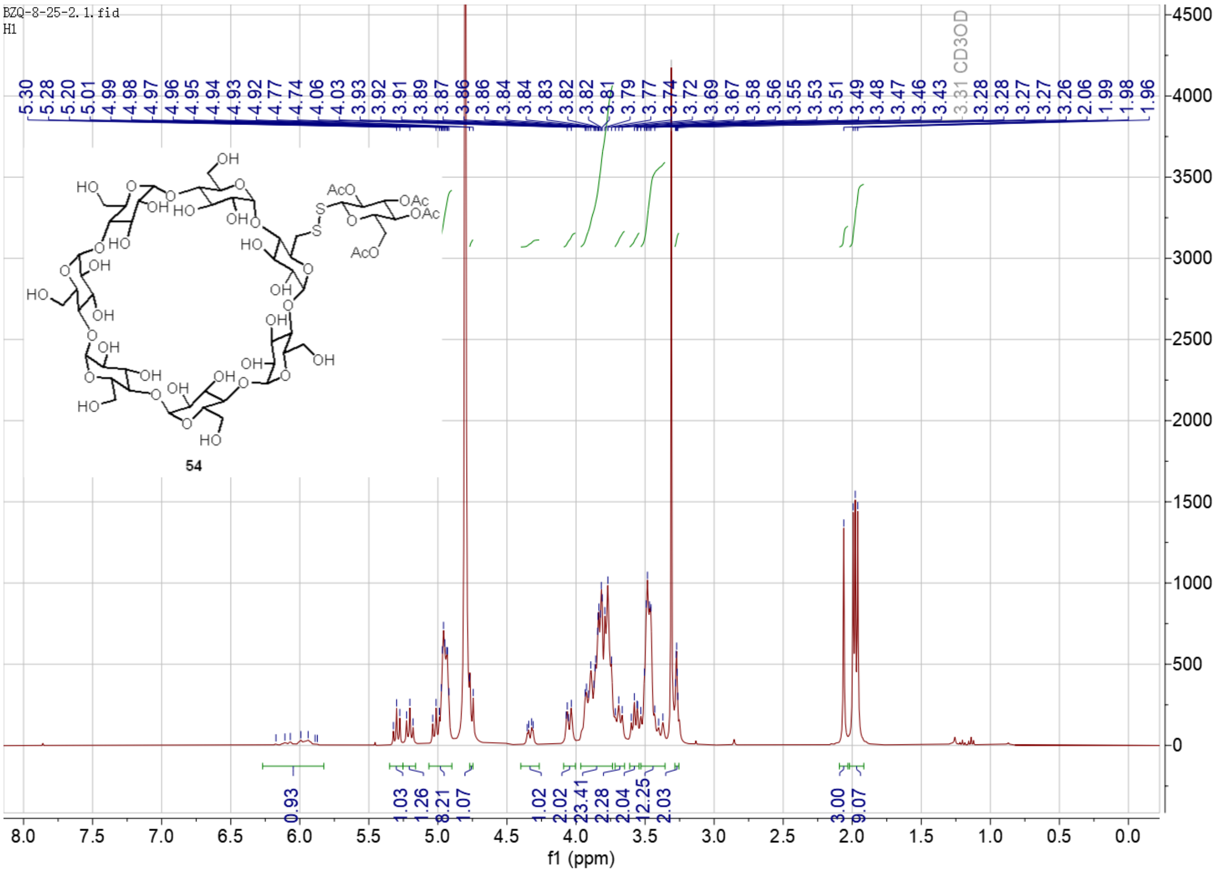

Supplementary Figure 225. ^{1}H NMR (400 MHz, Methanol-d_4, 293 K) spectrum of **54**.

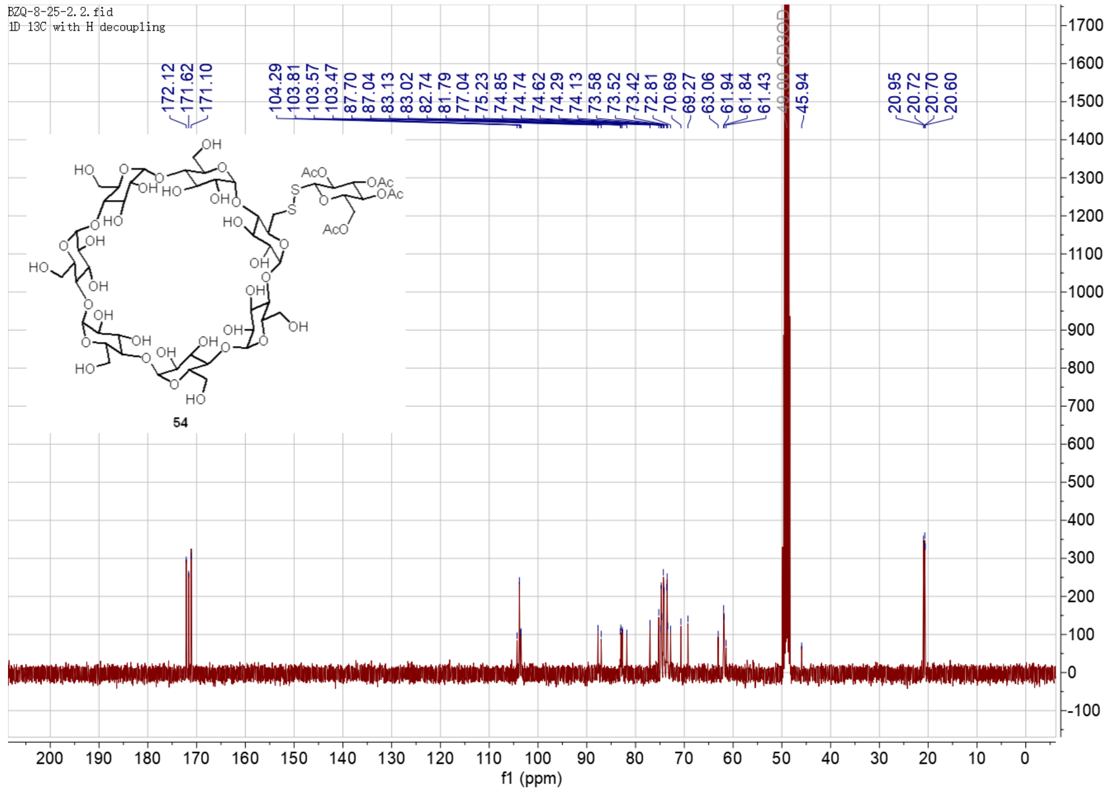

Supplementary Figure 226. ^{13}C NMR (101 MHz, Methanol-d_4, 293 K) spectrum of **54**.

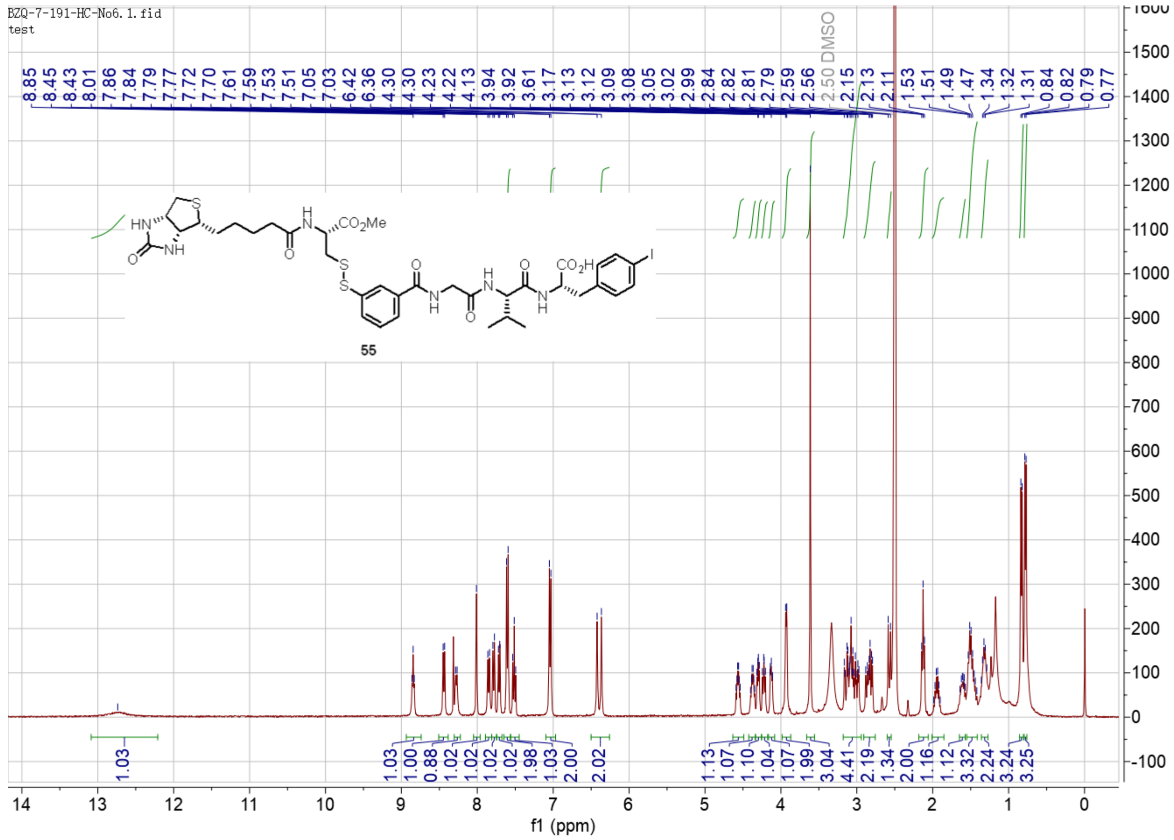

Supplementary Figure 227. ^{1}H NMR (400 MHz, DMSO-d_6, 293 K) spectrum of **55**.

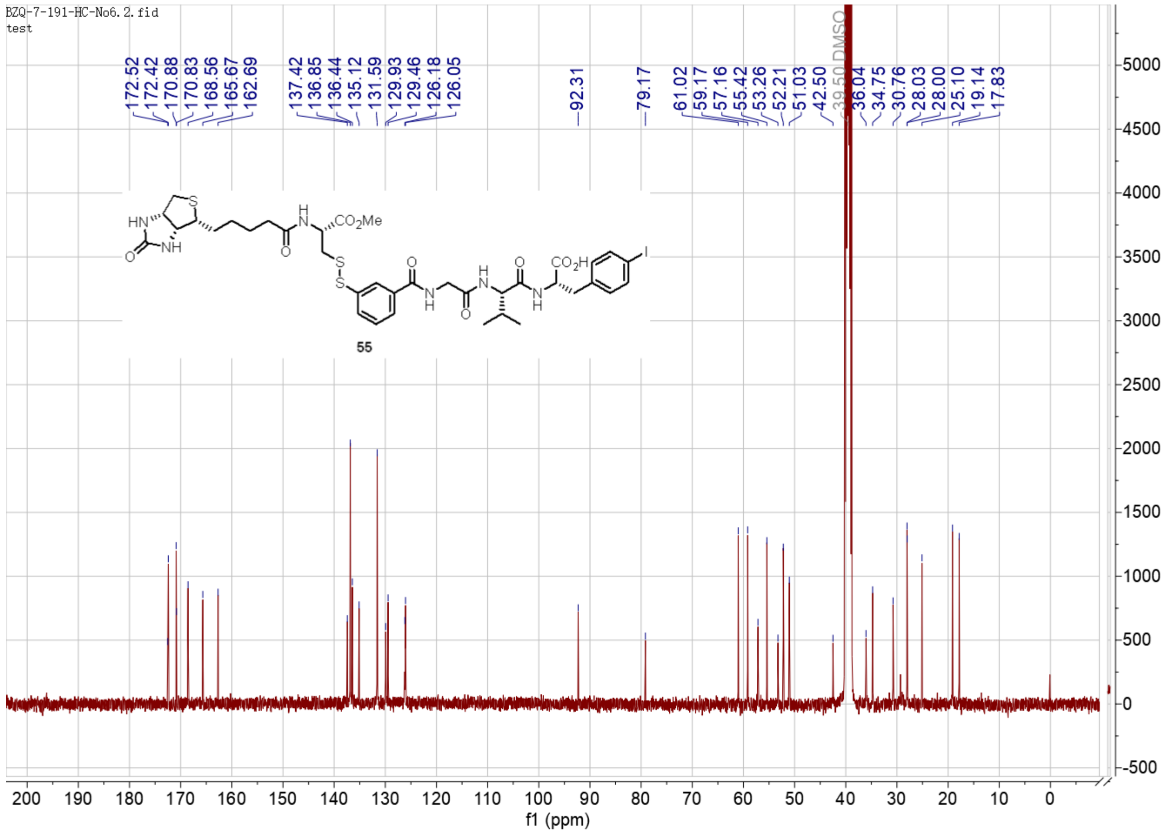

Supplementary Figure 228. ^{13}C NMR (101 MHz, DMSO-d_6, 293 K) spectrum of **55**.

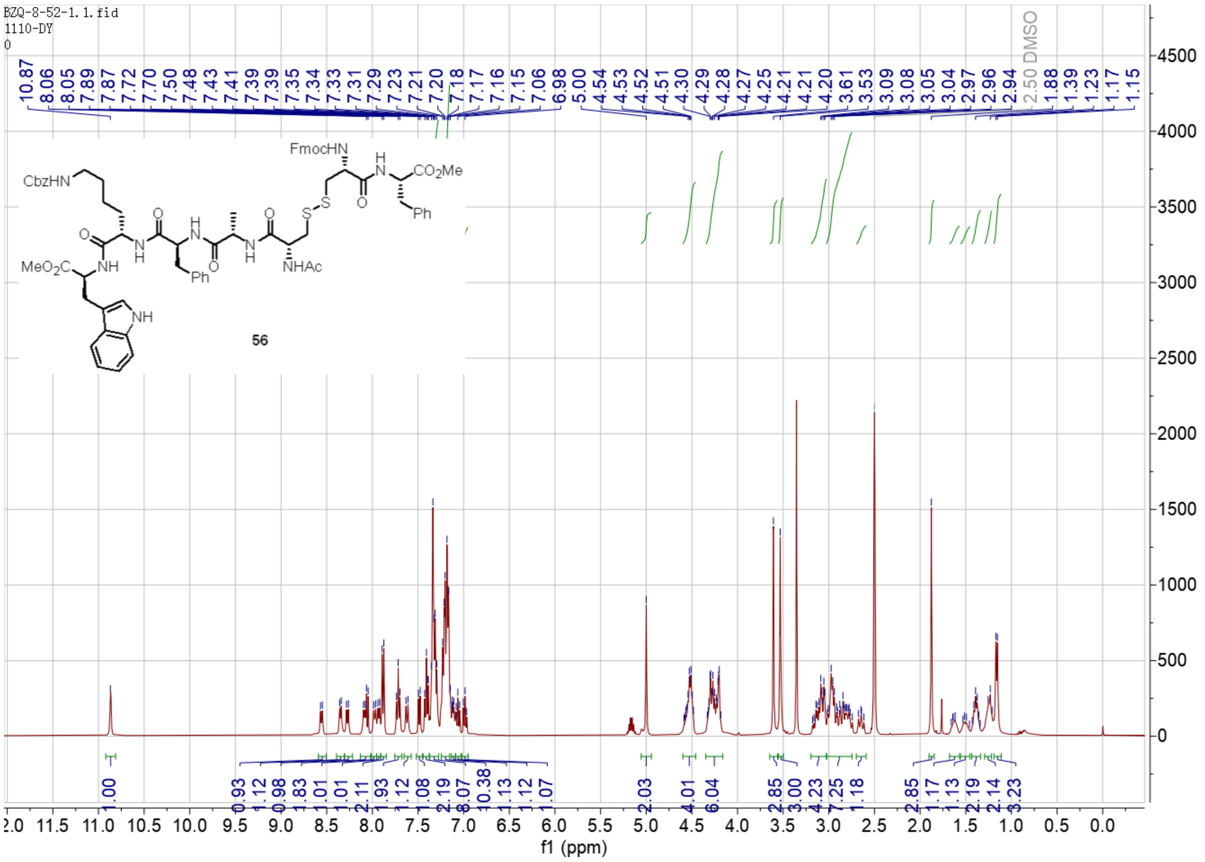

Supplementary Figure 229. ^{1}H NMR (400 MHz, DMSO-d_6, 293 K) spectrum of **56**.

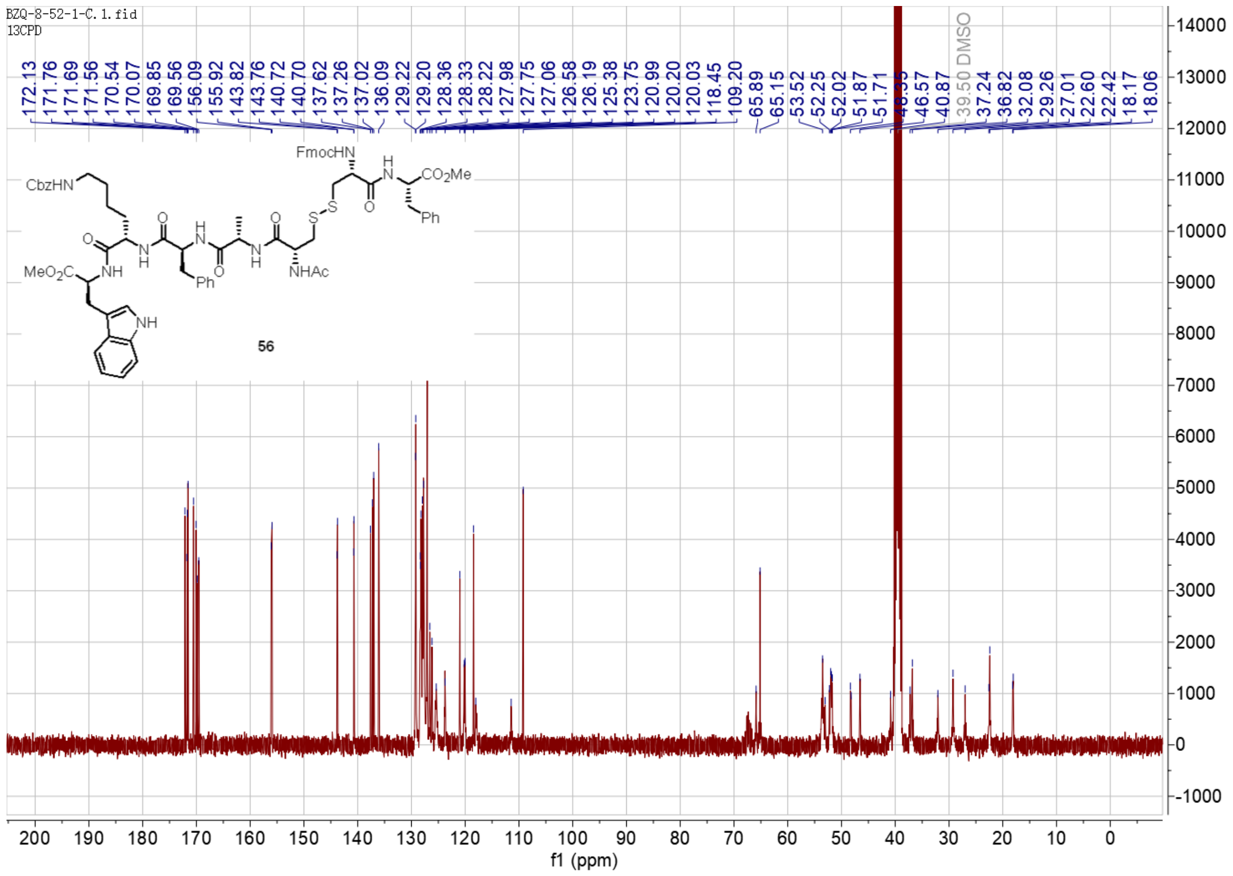

Supplementary Figure 230. ^{13}C NMR (101 MHz, DMSO-d_6, 293 K) spectrum of **56**.

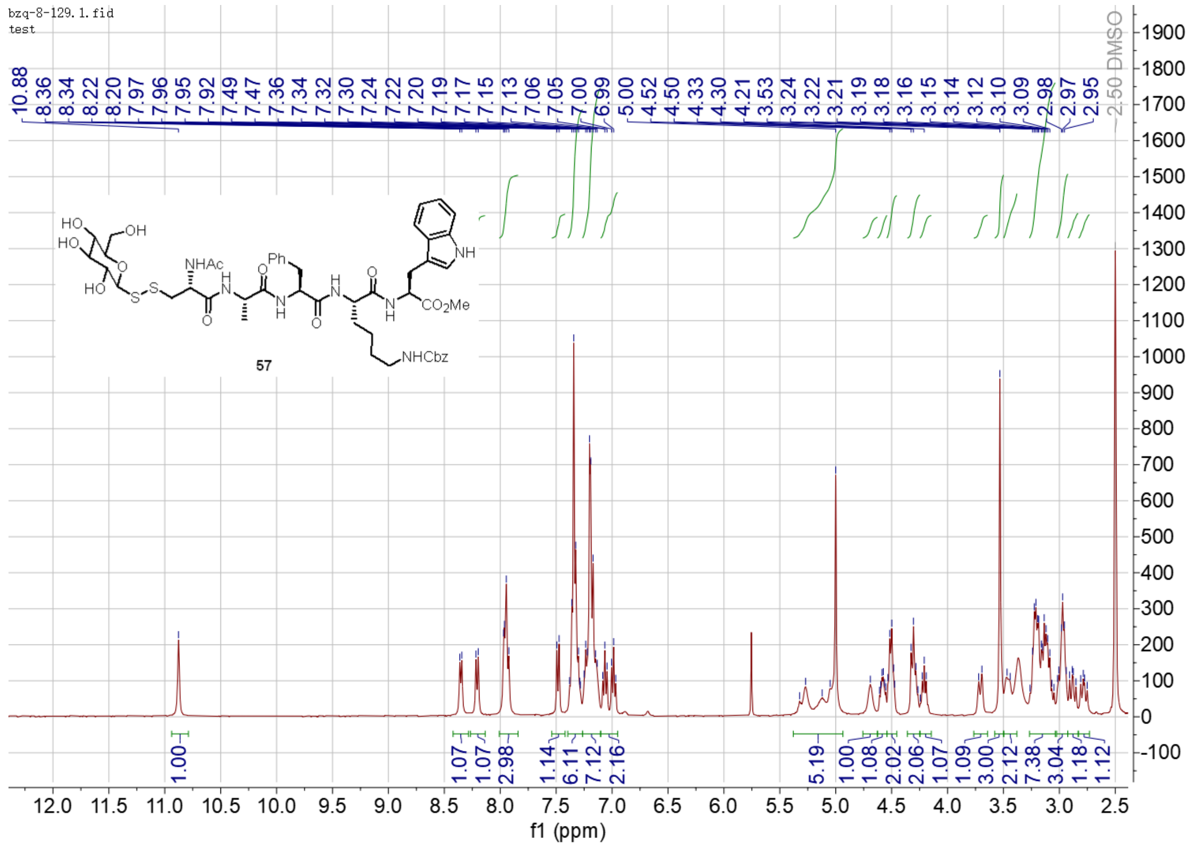

Supplementary Figure 231. ^{1}H NMR (400 MHz, DMSO-d_6, 293 K) spectrum of **57**.

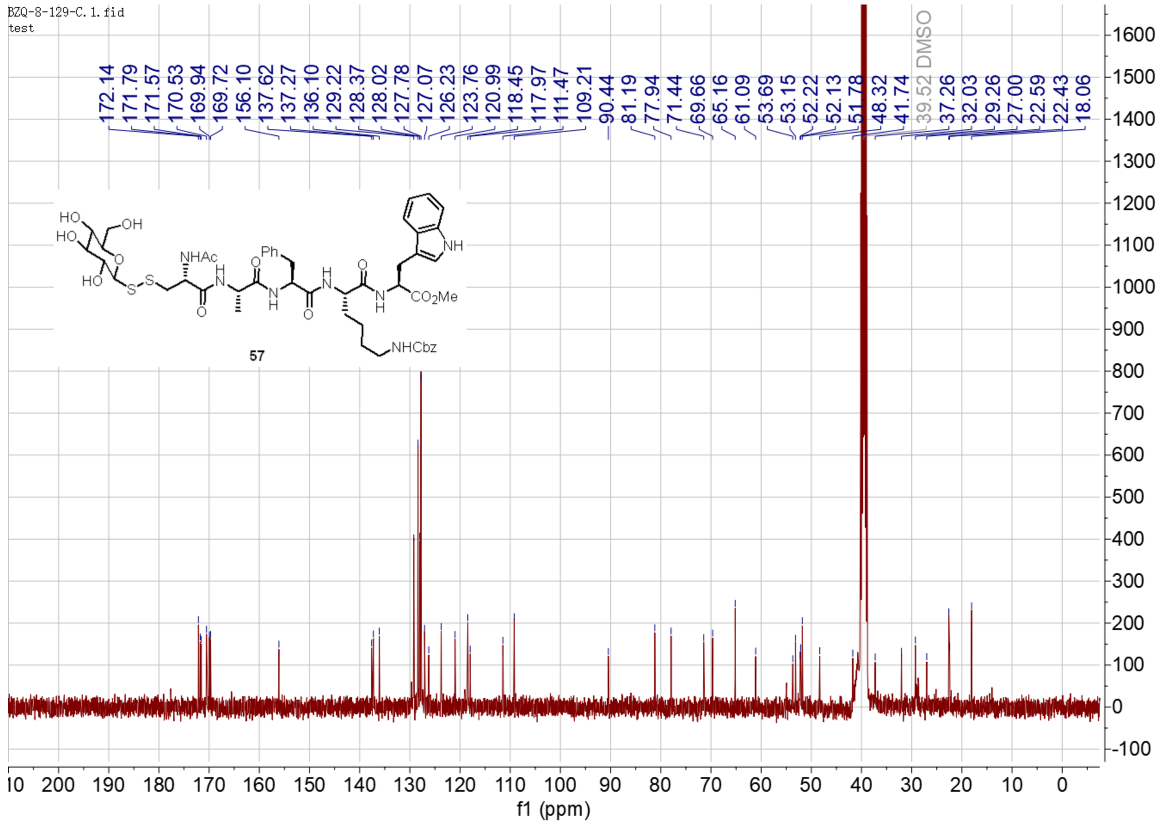

Supplementary Figure 232. ^{13}C NMR (101 MHz, DMSO-d_6, 293 K) spectrum of **57**.

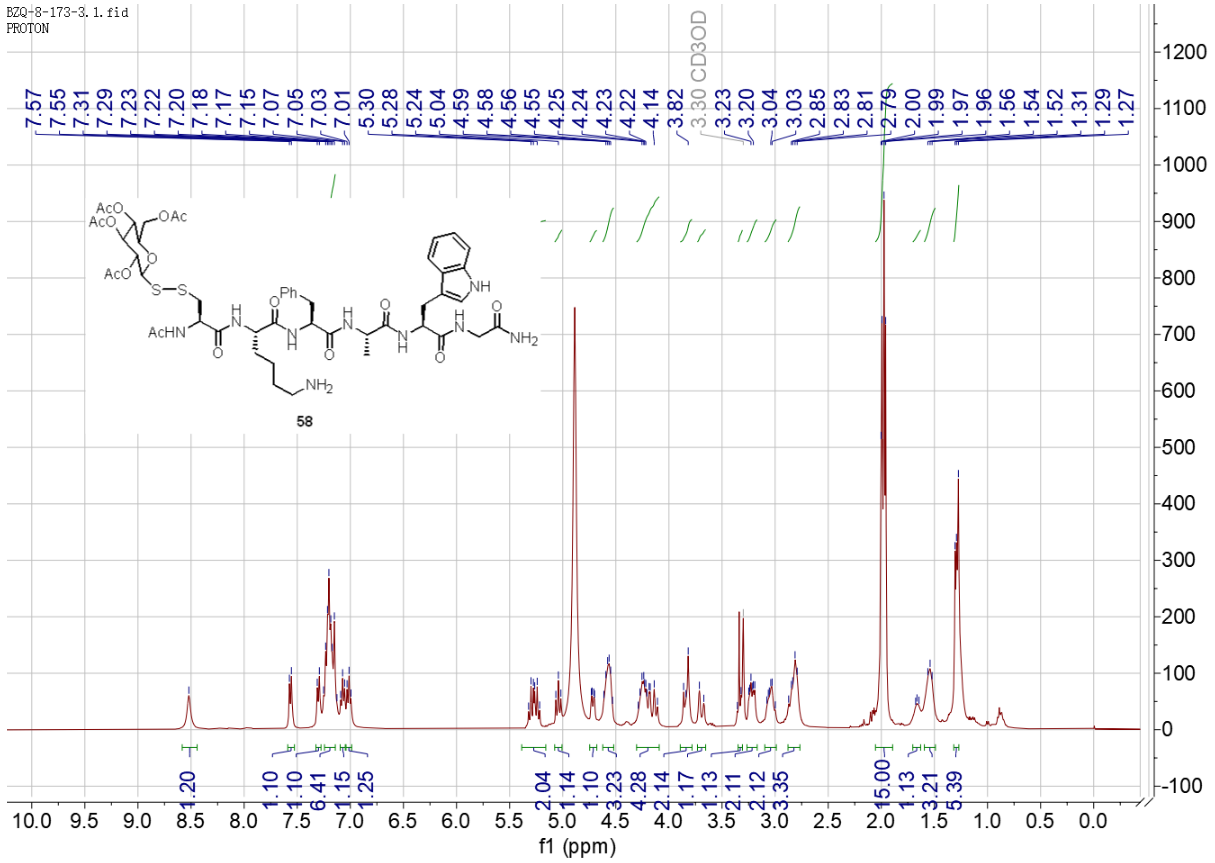

Supplementary Figure 233. ^{1}H NMR (400 MHz, Methanol-d_4, 293 K) spectrum of **58**.

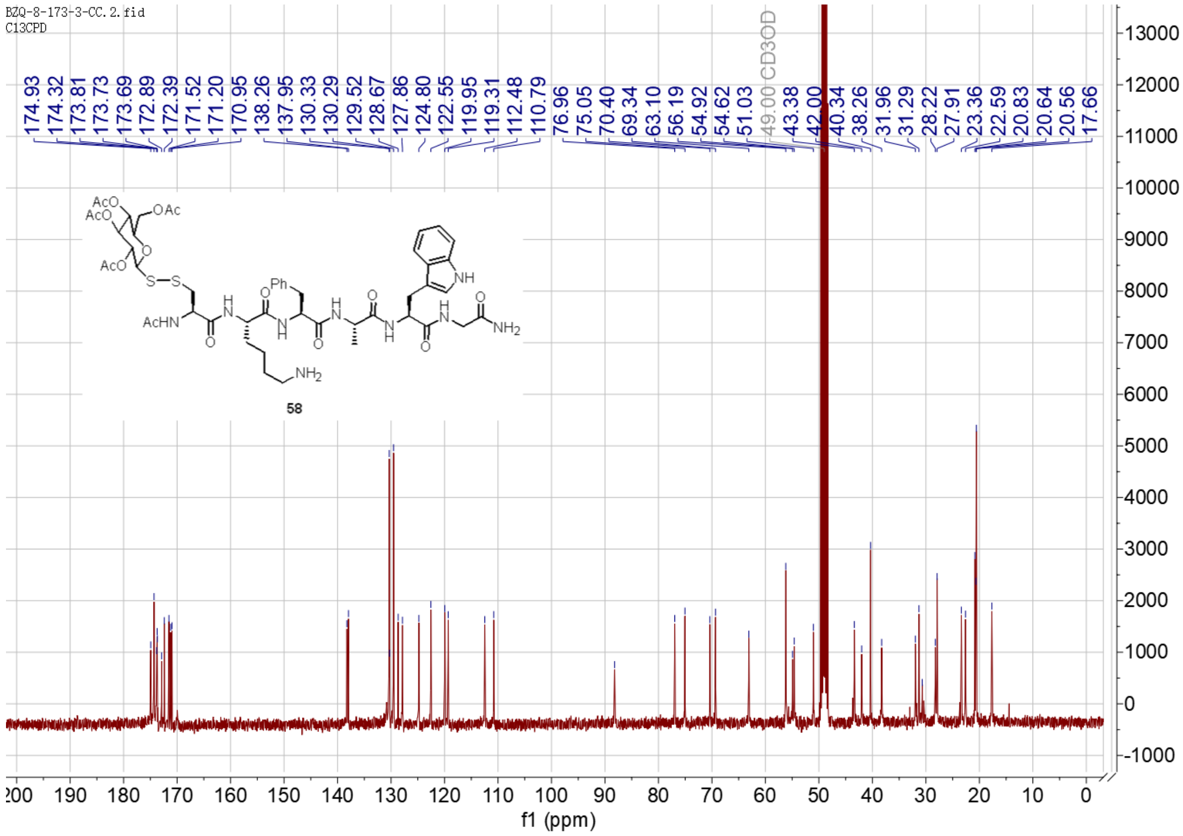

Supplementary Figure 234. ^{13}C NMR (101 MHz, Methanol-d_4, 293 K) spectrum of **58**.

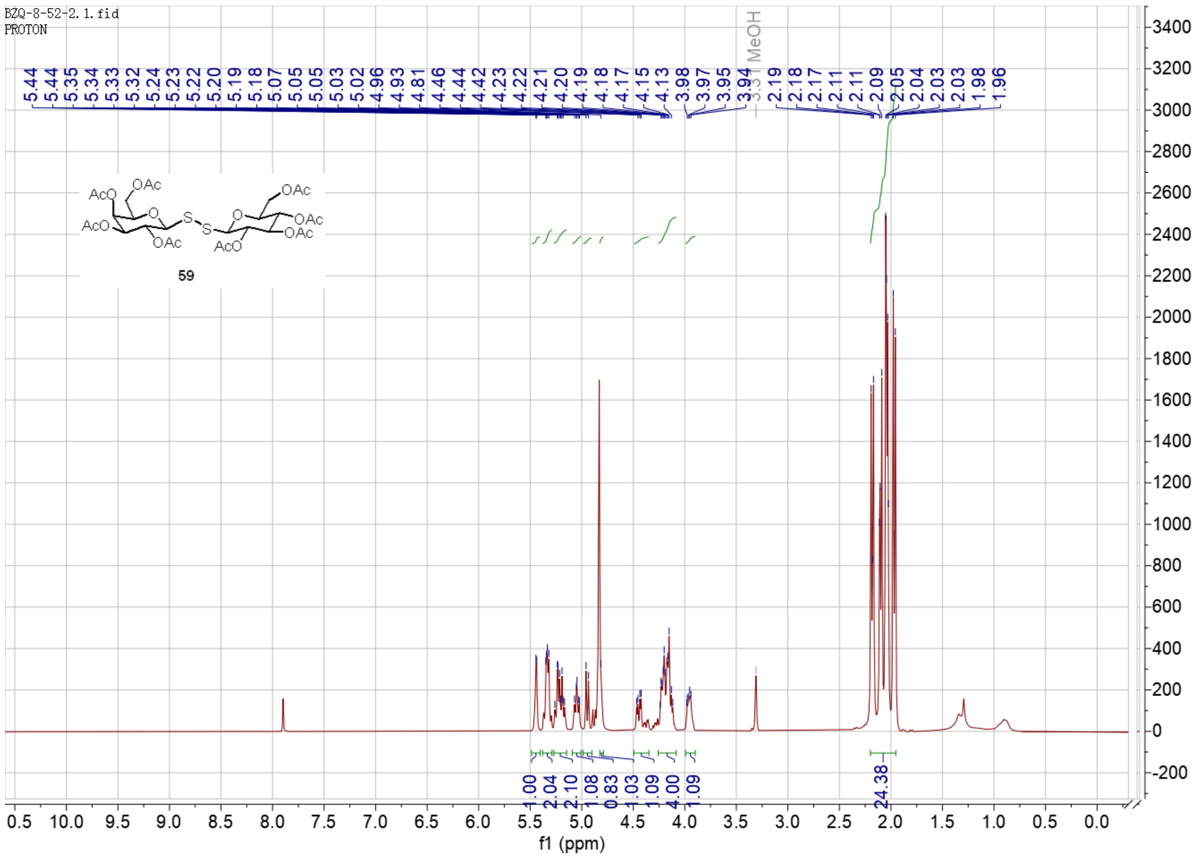

Supplementary Figure 235. ^{1}H NMR (400 MHz, Methanol-d_4, 293 K) spectrum of **59**.

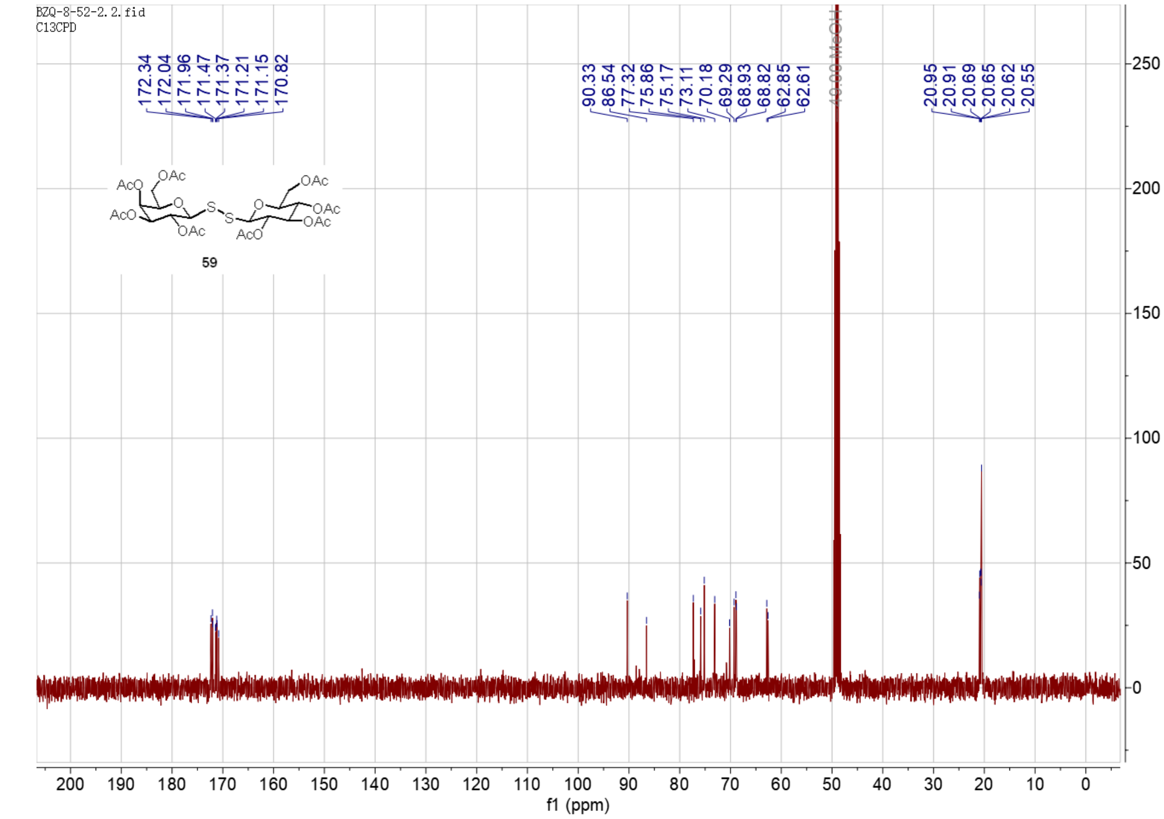

Supplementary Figure 236. ^{13}C NMR (101 MHz, Methanol-d_4, 293 K) spectrum of **59**.

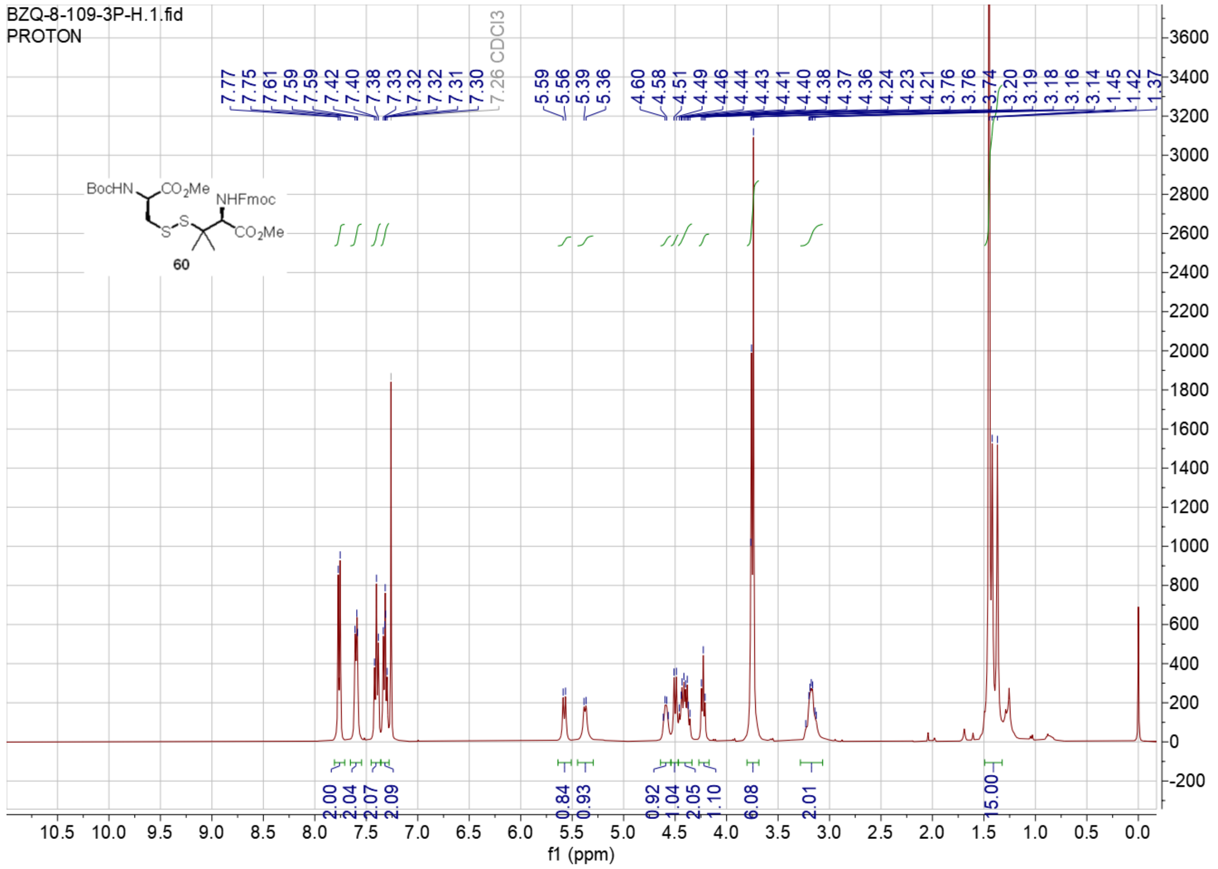

Supplementary Figure 237. ^{1}H NMR (400 MHz, $CDCl_3$, 293 K) spectrum of **60**.

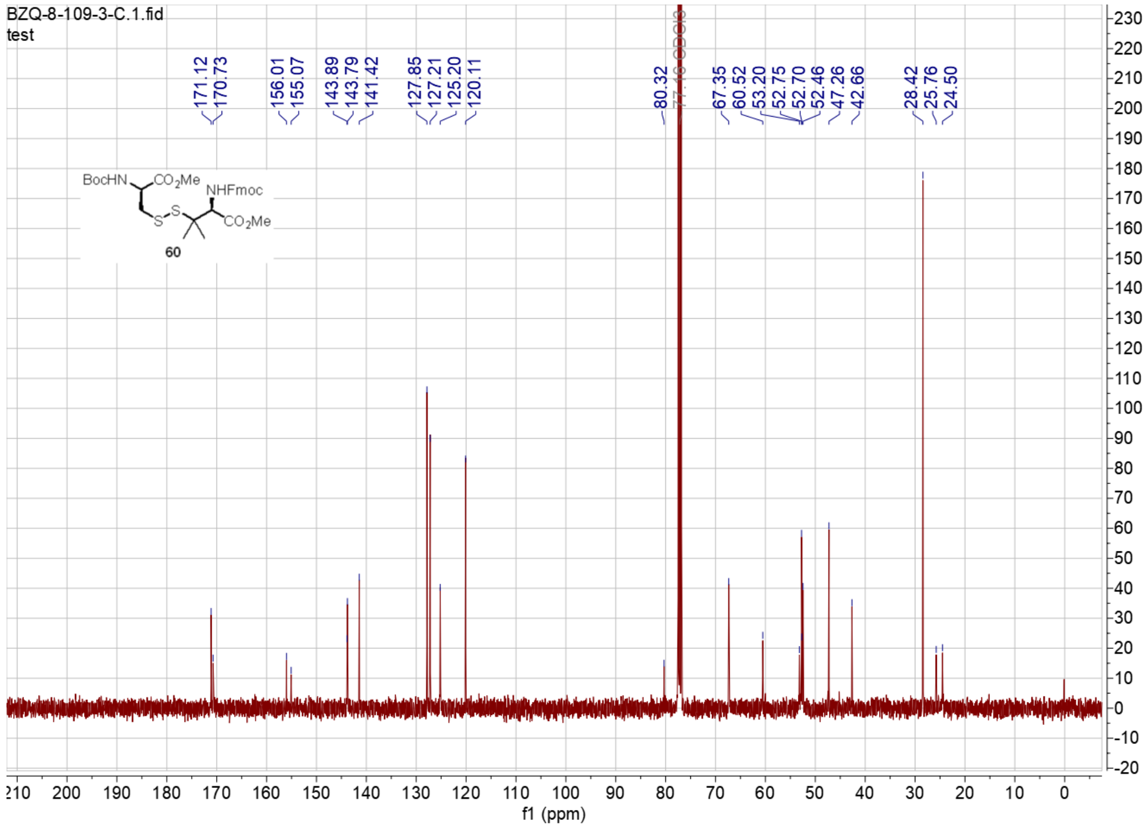

Supplementary Figure 238. ^{13}C NMR (101 MHz, $CDCl_3$, 293 K) spectrum of **60**.

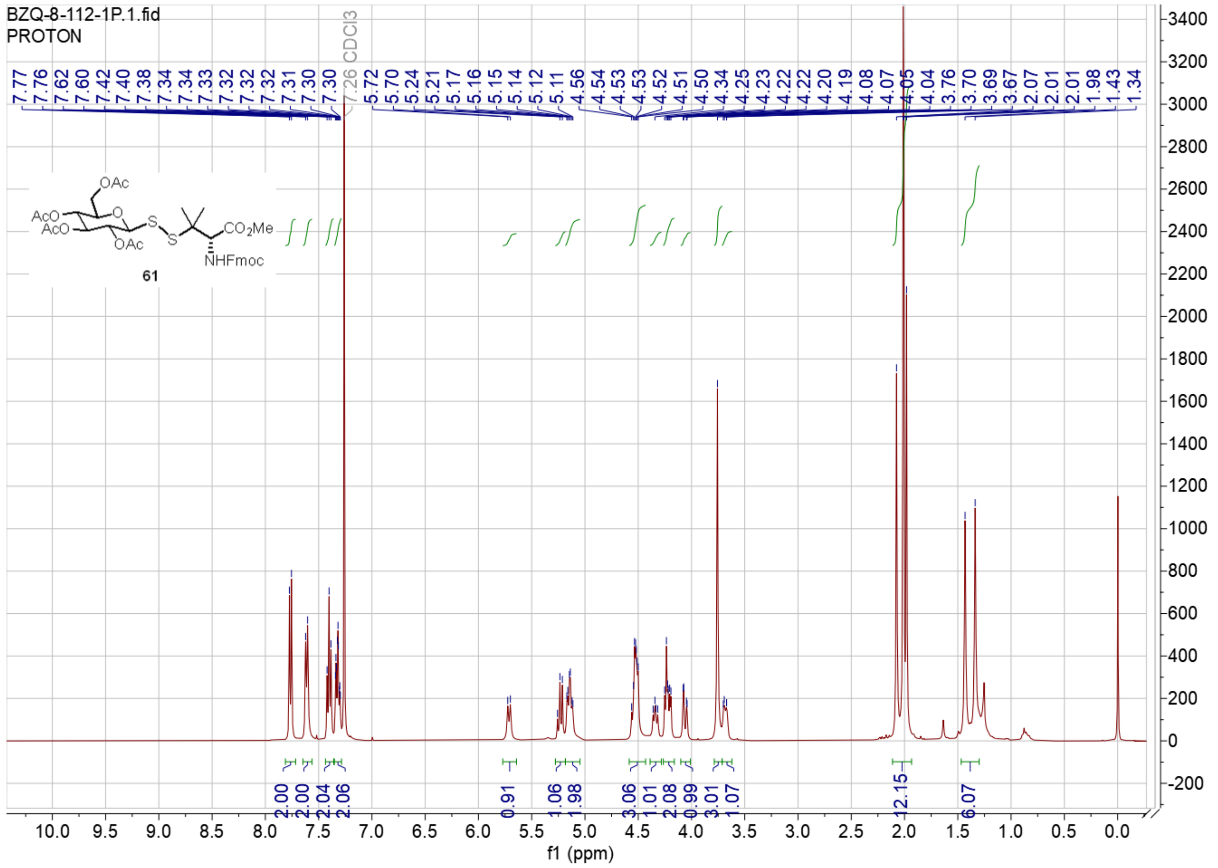

Supplementary Figure 239. ^{1}H NMR (400 MHz, $CDCl_3$, 293 K) spectrum of **61**.

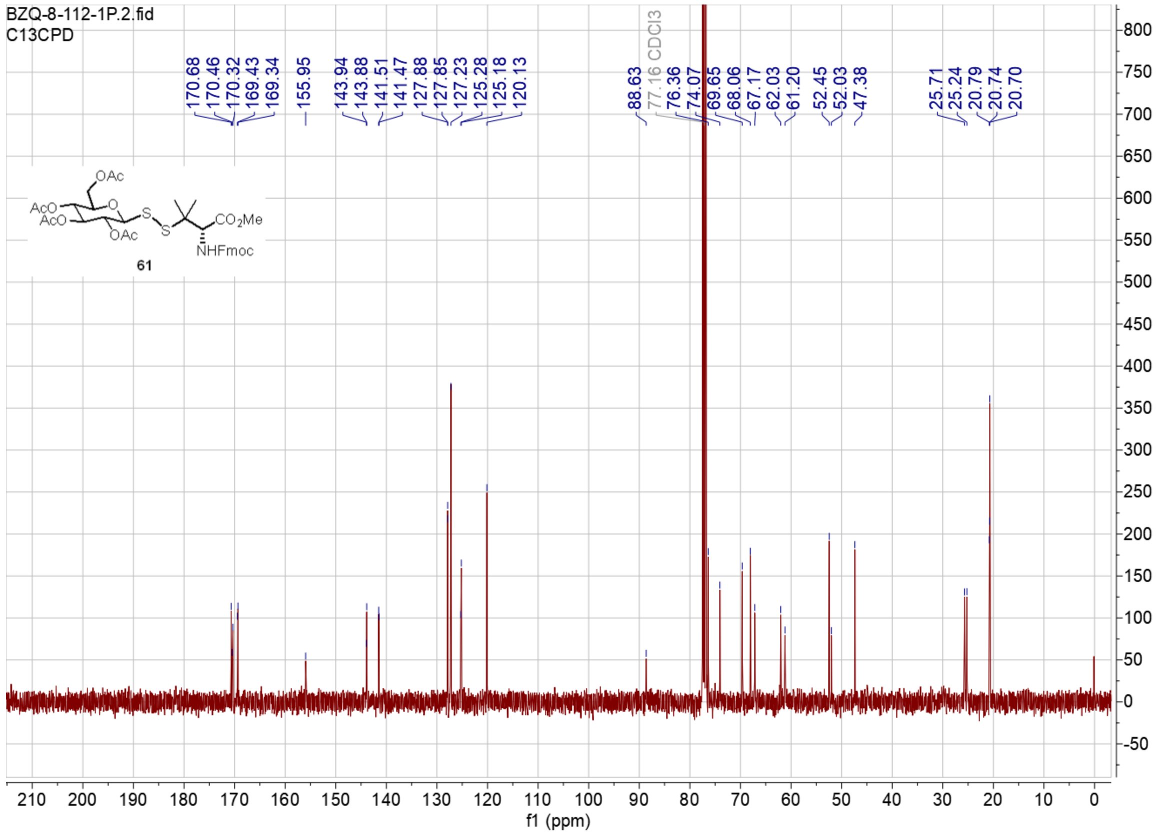

Supplementary Figure 240. ^{13}C NMR (101 MHz, $CDCl_3$, 293 K) spectrum of **61**.

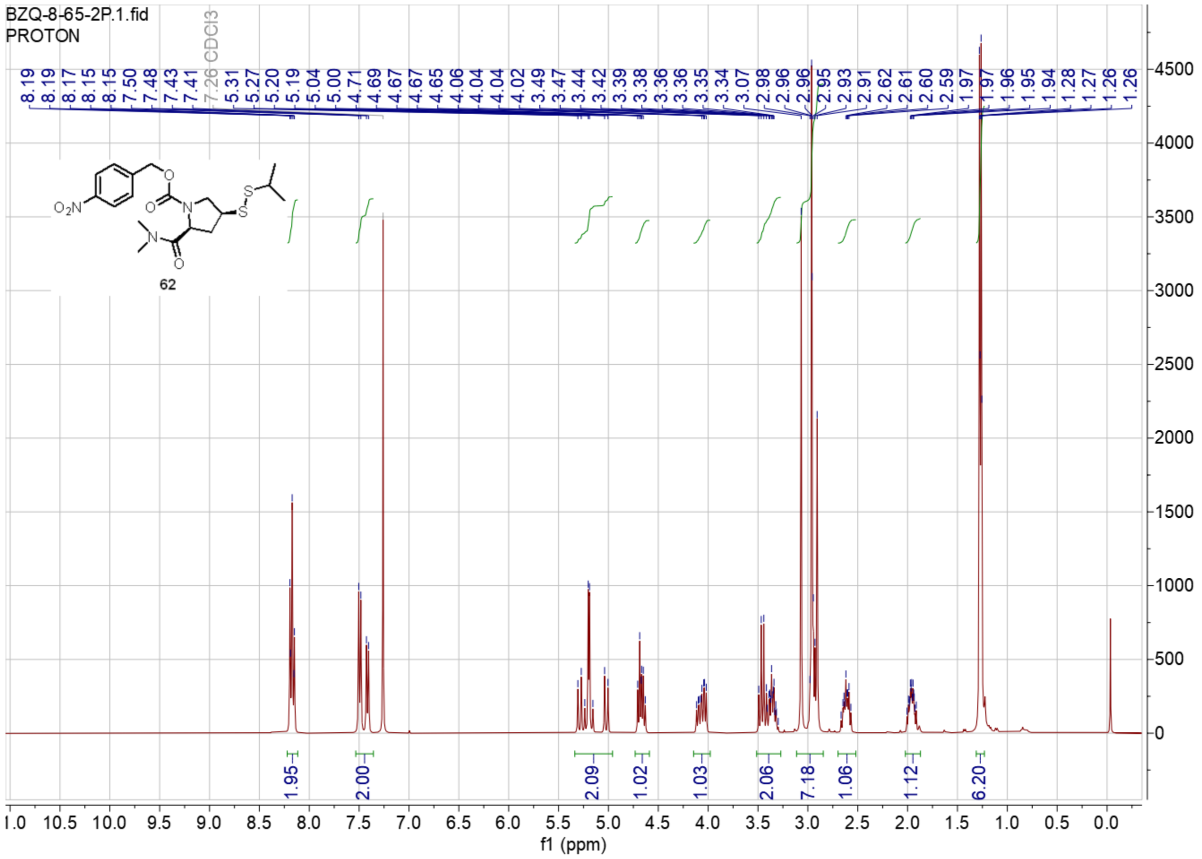

Supplementary Figure 241. ^{1}H NMR (400 MHz, $CDCl_3$, 293 K) spectrum of **62**.

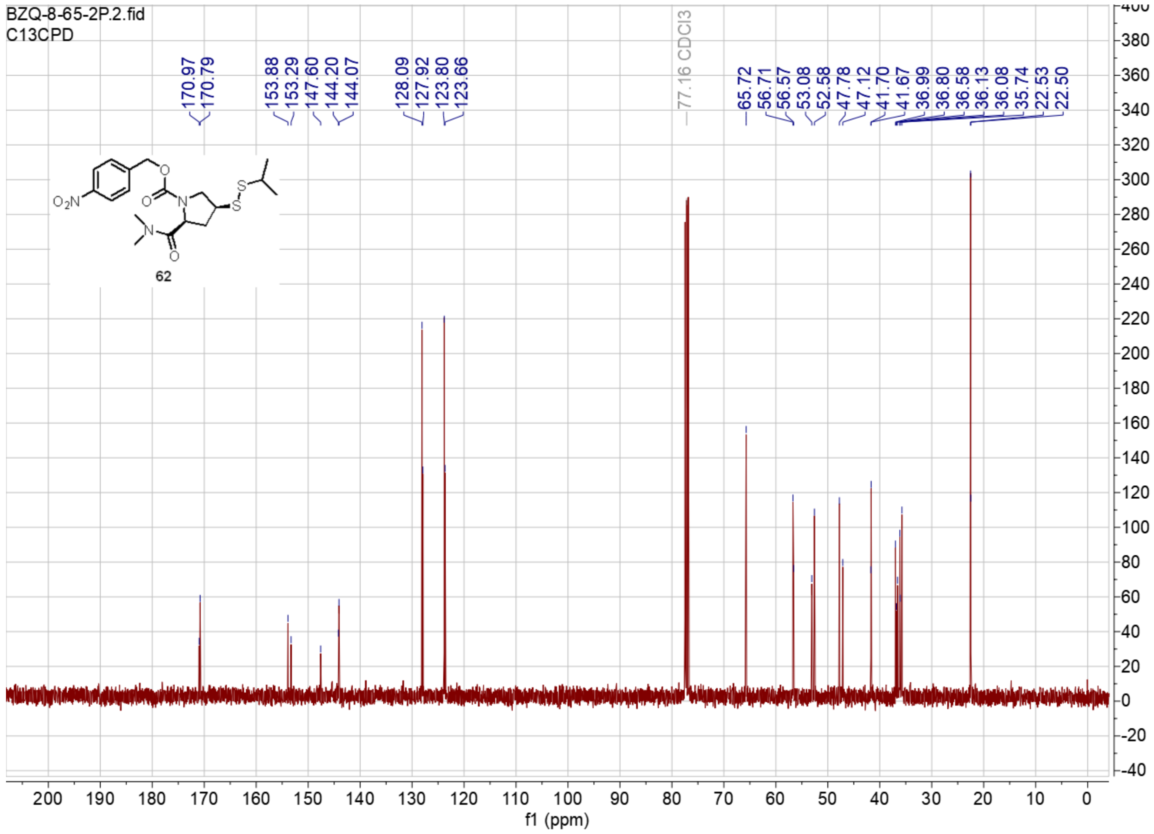

Supplementary Figure 242. ^{13}C NMR (101 MHz, $CDCl_3$, 293 K) spectrum of **62**.

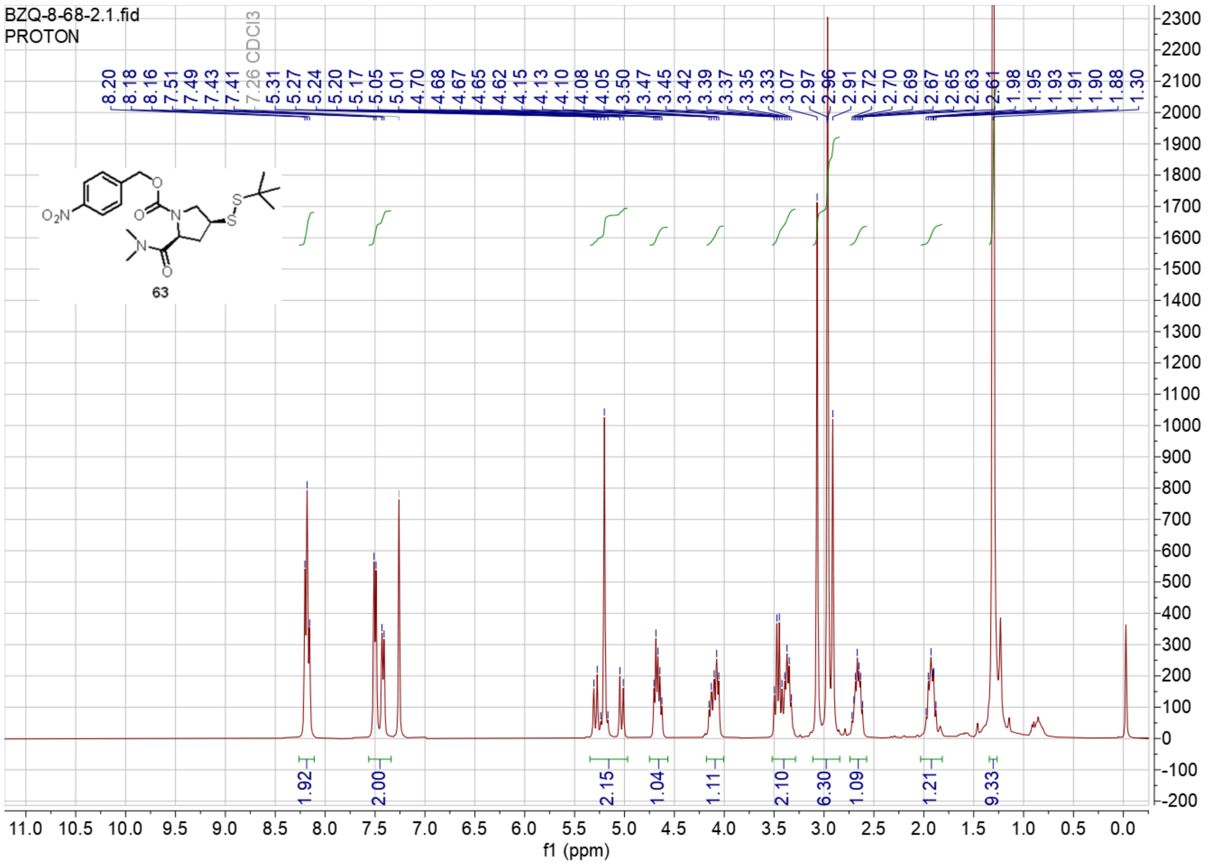

Supplementary Figure 243. ^{1}H NMR (400 MHz, $CDCl_3$, 293 K) spectrum of **63**.

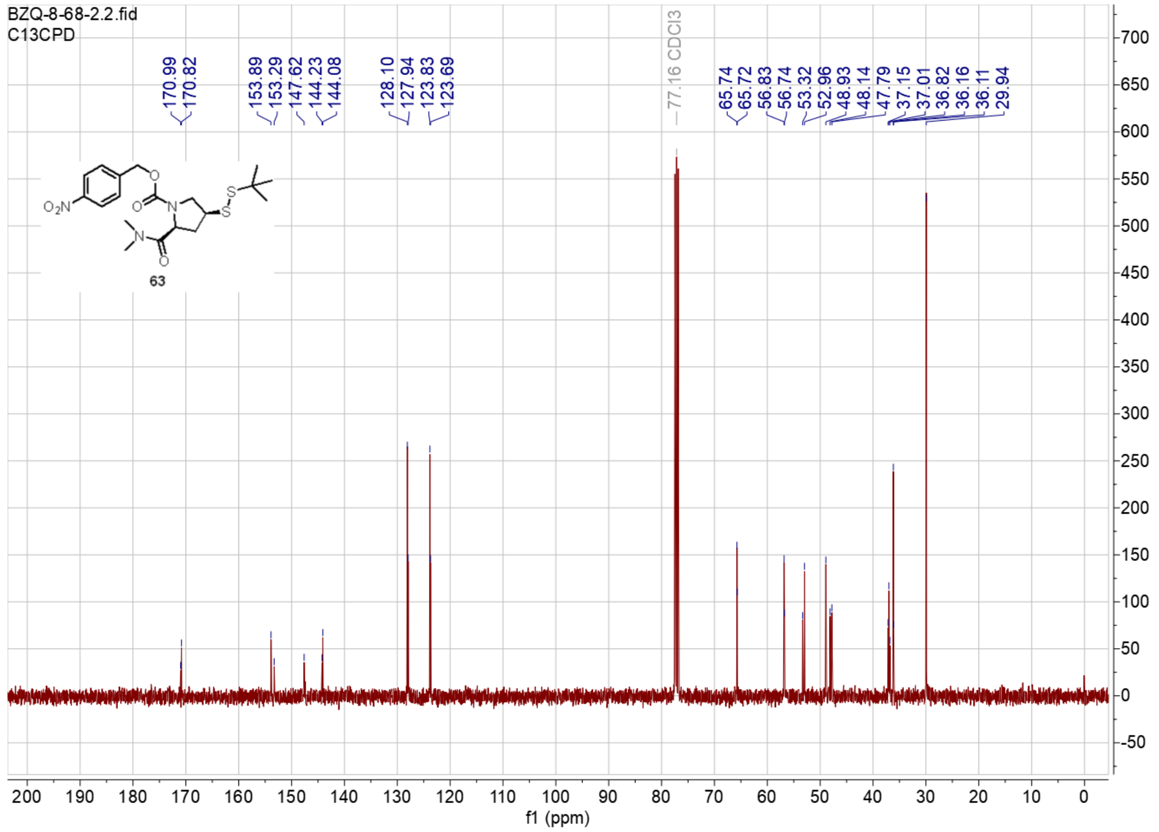

Supplementary Figure 244. ^{13}C NMR (101 MHz, $CDCl_3$, 293 K) spectrum of **63**.

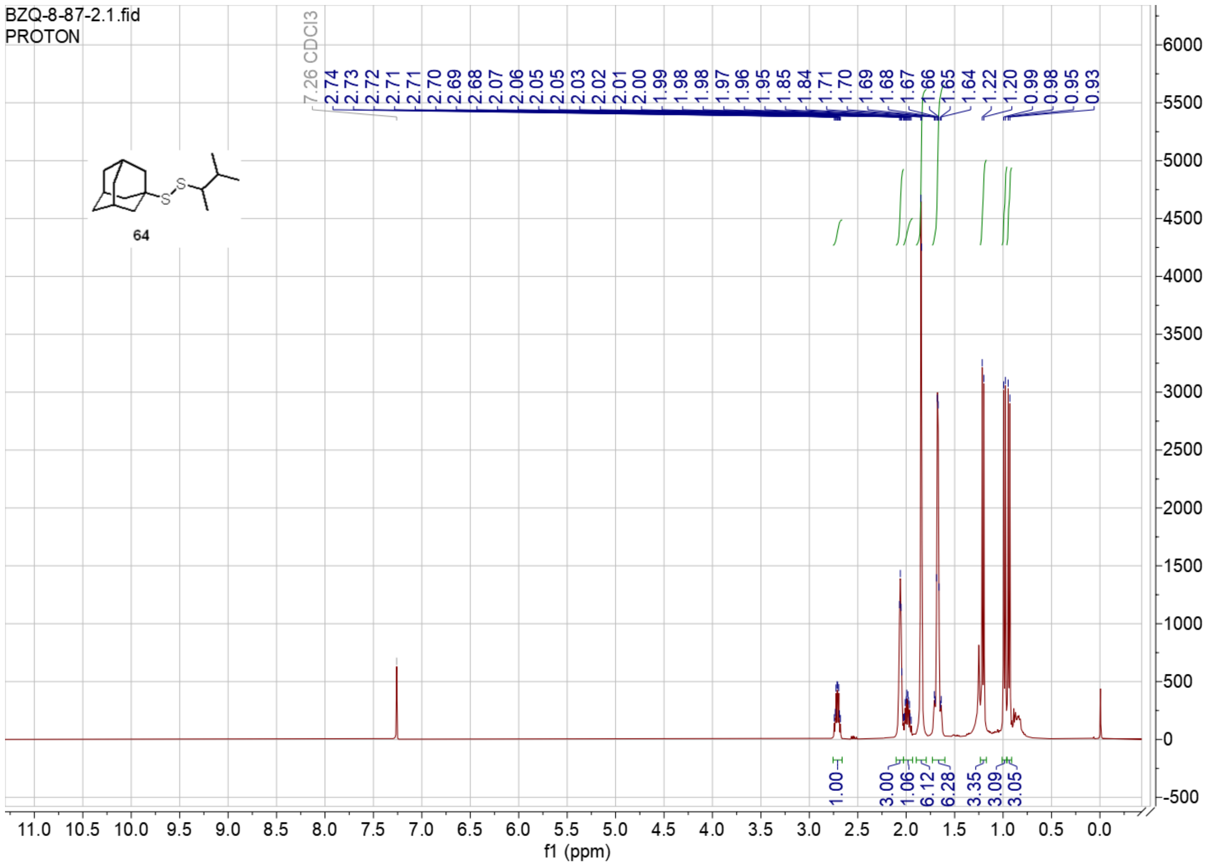

Supplementary Figure 245. ^{1}H NMR (400 MHz, $CDCl_3$, 293 K) spectrum of **64**.

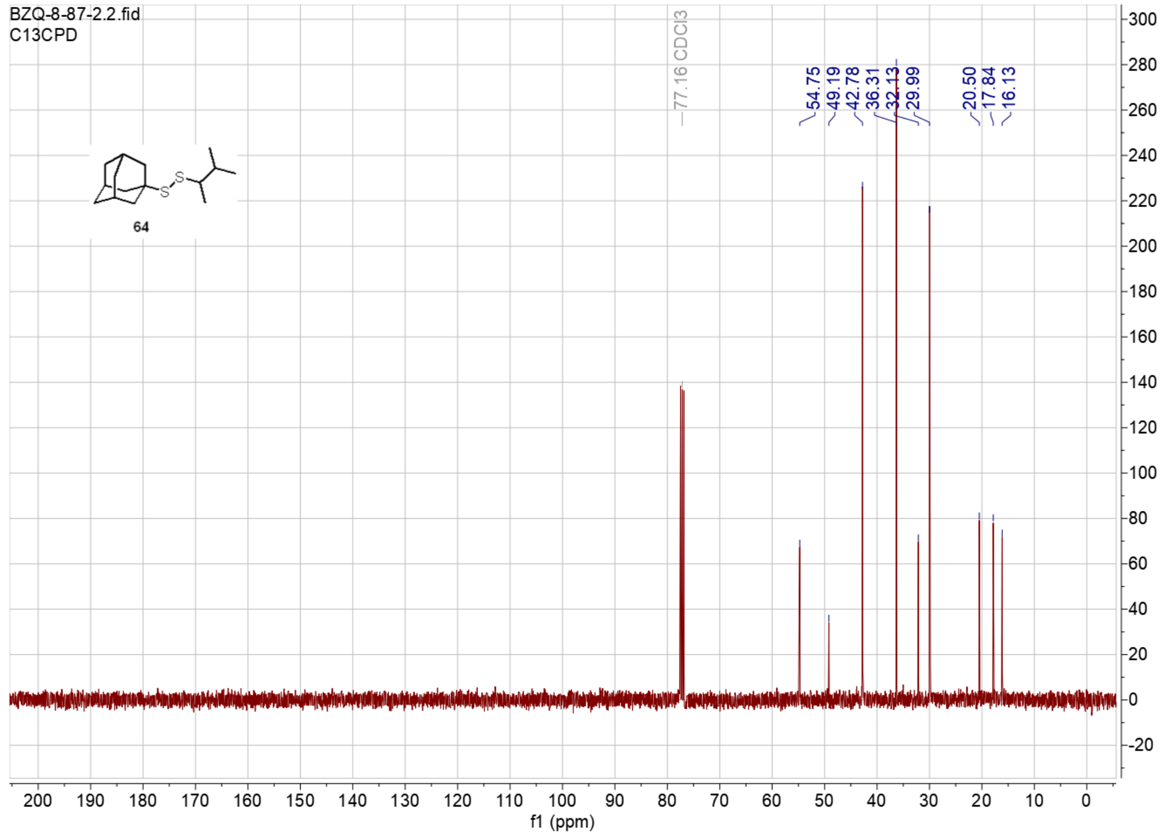

Supplementary Figure 246. ^{13}C NMR (101 MHz, $CDCl_3$, 293 K) spectrum of **64**.

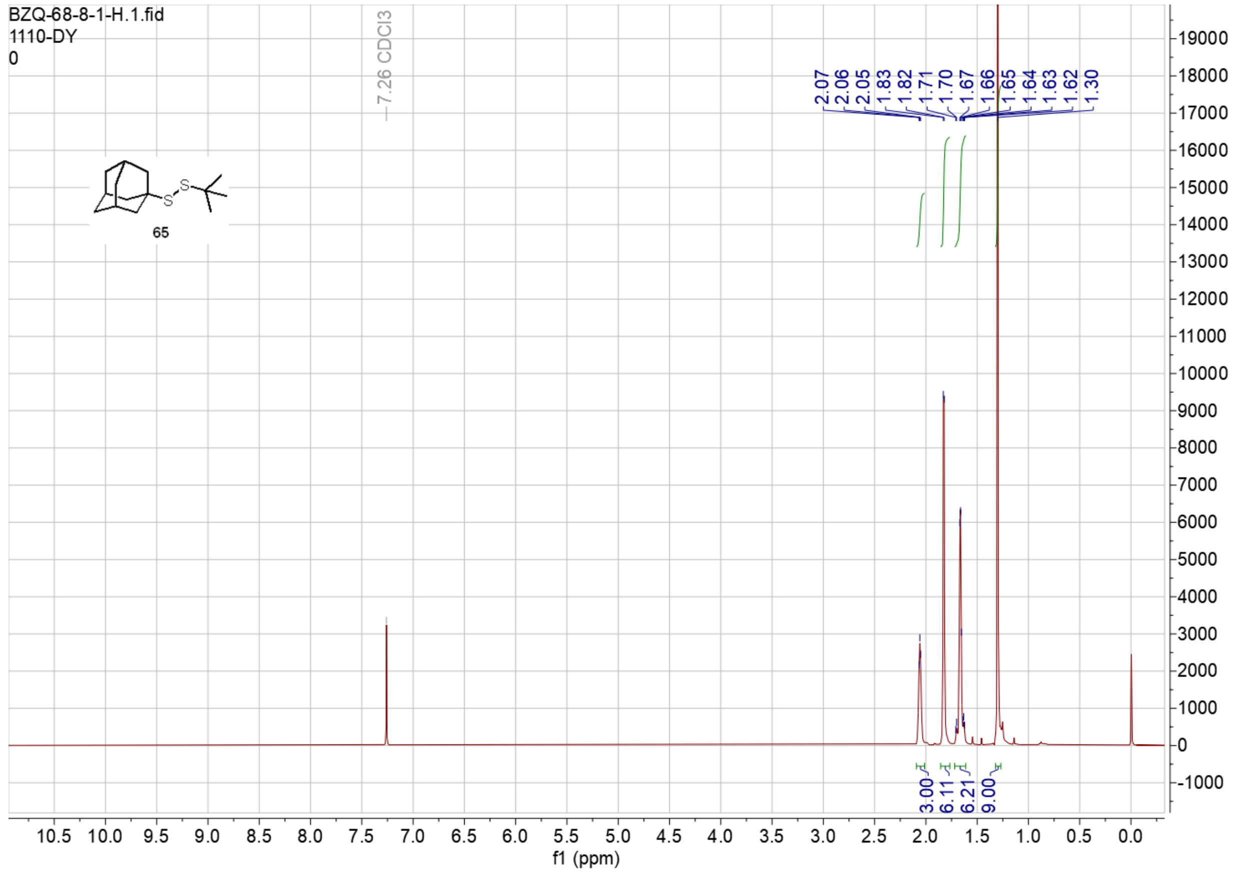

Supplementary Figure 247. ^{1}H NMR (400 MHz, $CDCl_3$, 293 K) spectrum of **65**.

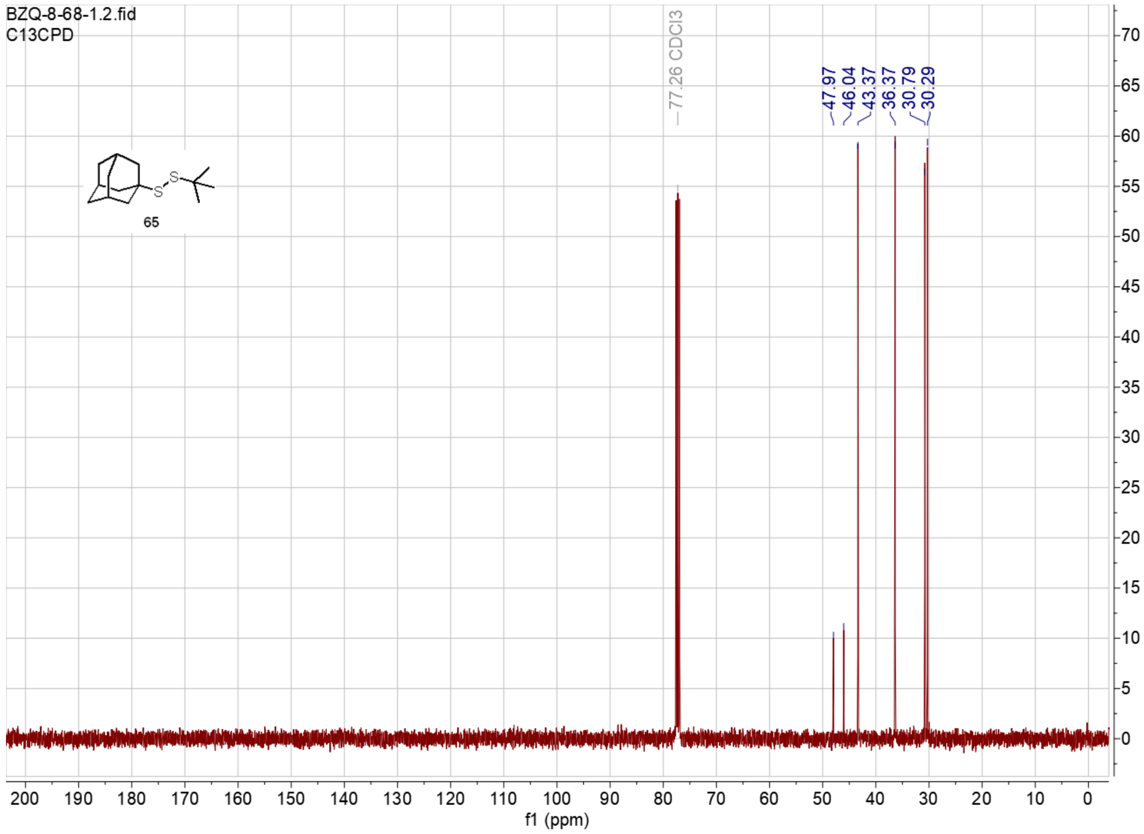

Supplementary Figure 248. ^{13}C NMR (101 MHz, $CDCl_3$, 293 K) spectrum of **65**.

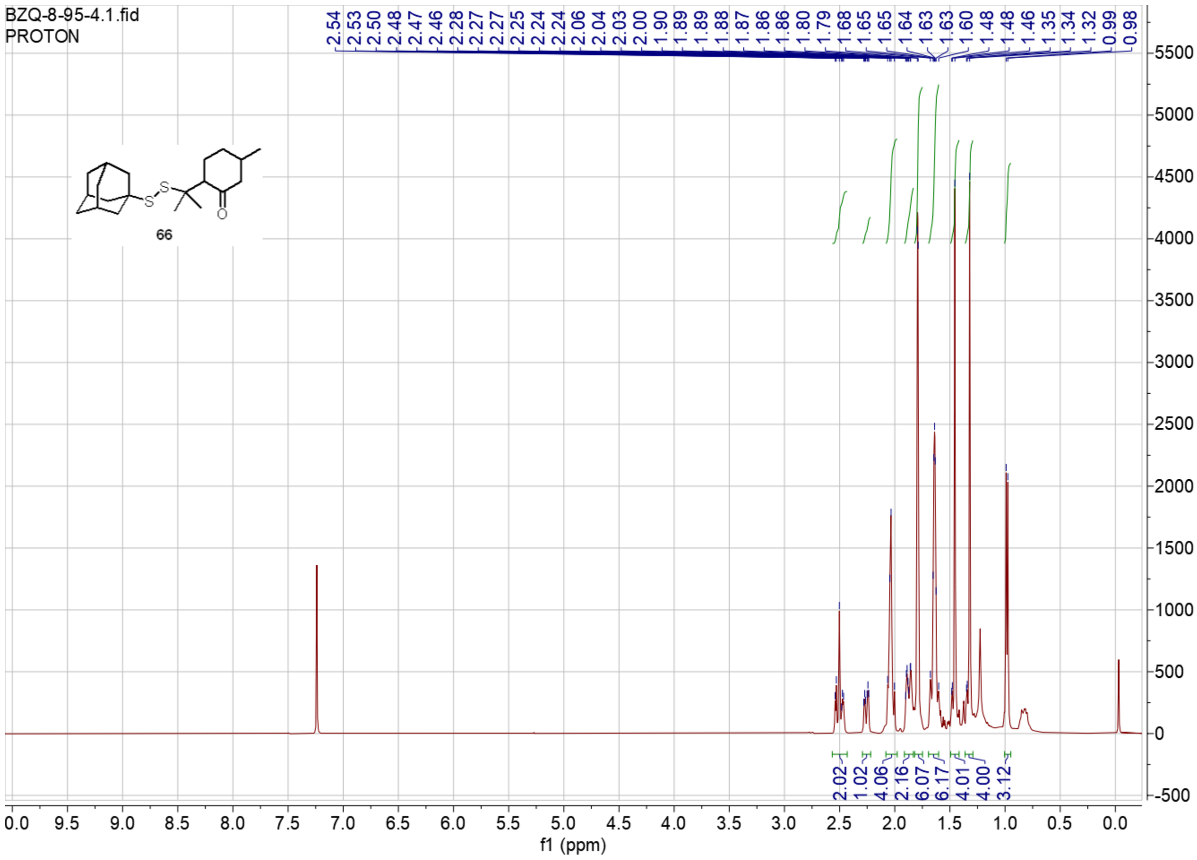

Supplementary Figure 249. ^{1}H NMR (400 MHz, $CDCl_3$, 293 K) spectrum of **66**.

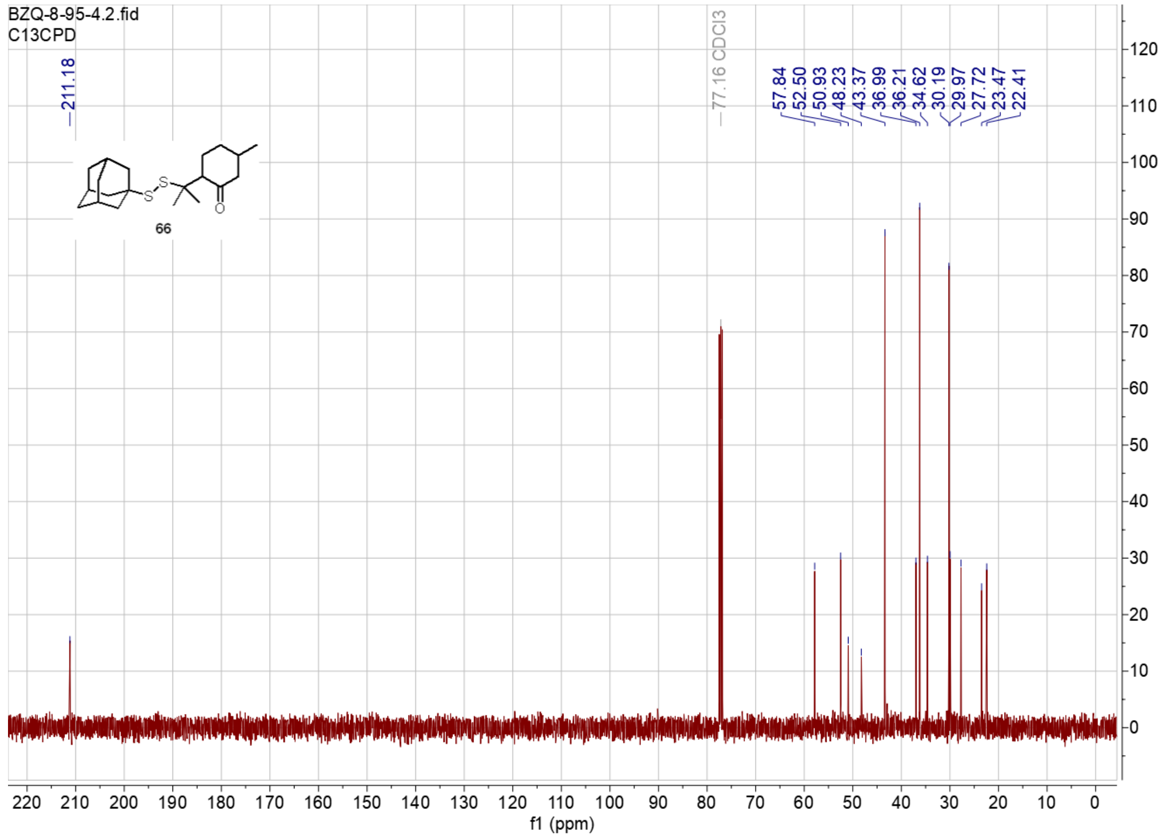

Supplementary Figure 250. ^{13}C NMR (101 MHz, $CDCl_3$, 293 K) spectrum of **66**.

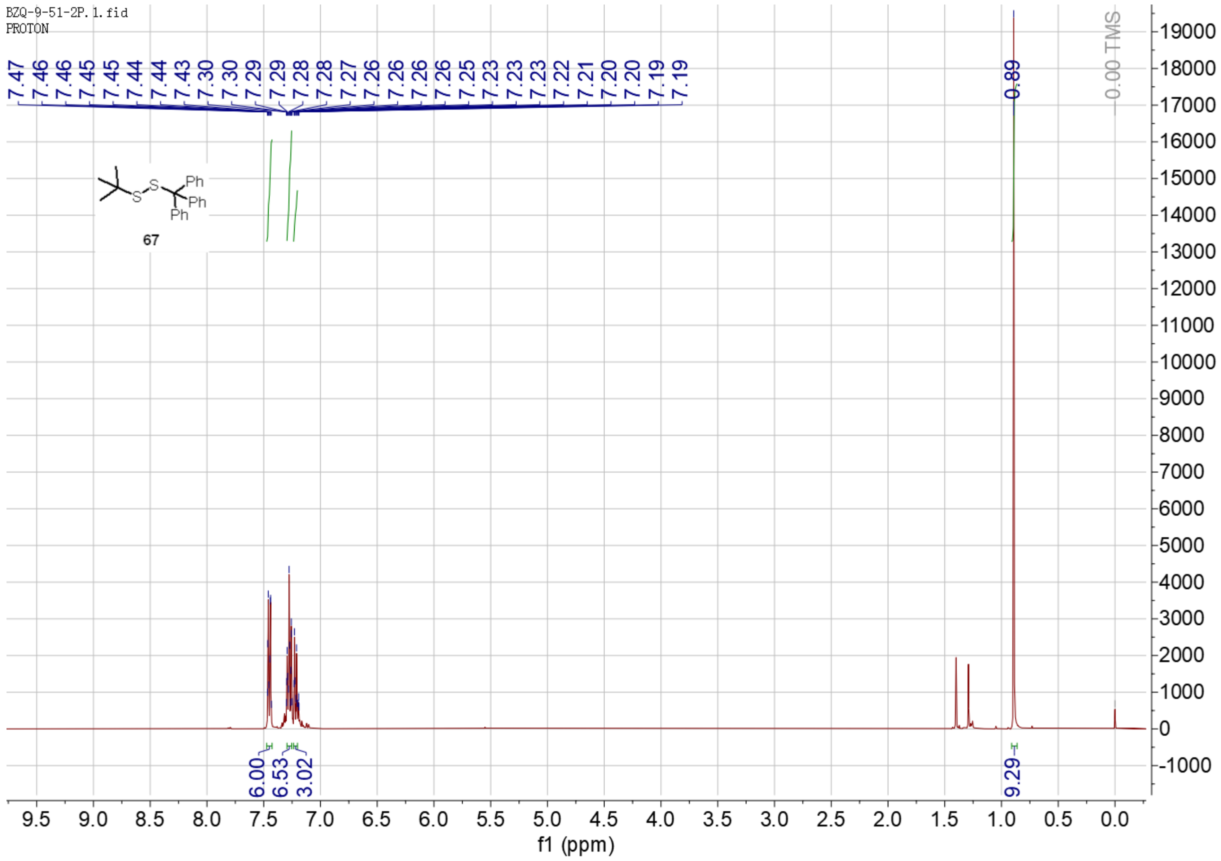

Supplementary Figure 251. ^{1}H NMR (400 MHz, $CDCl_3$, 293 K) spectrum of **67**.

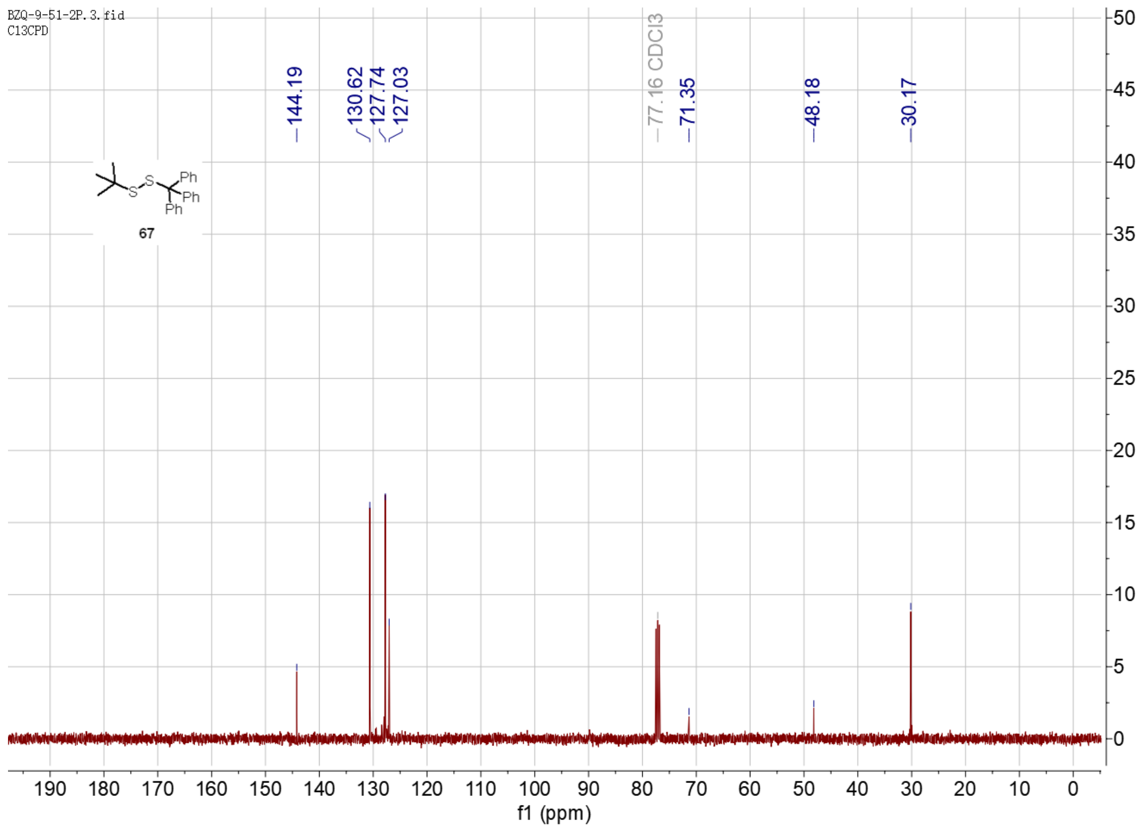

Supplementary Figure 252. ^{13}C NMR (101 MHz, $CDCl_3$, 293 K) spectrum of **67**.

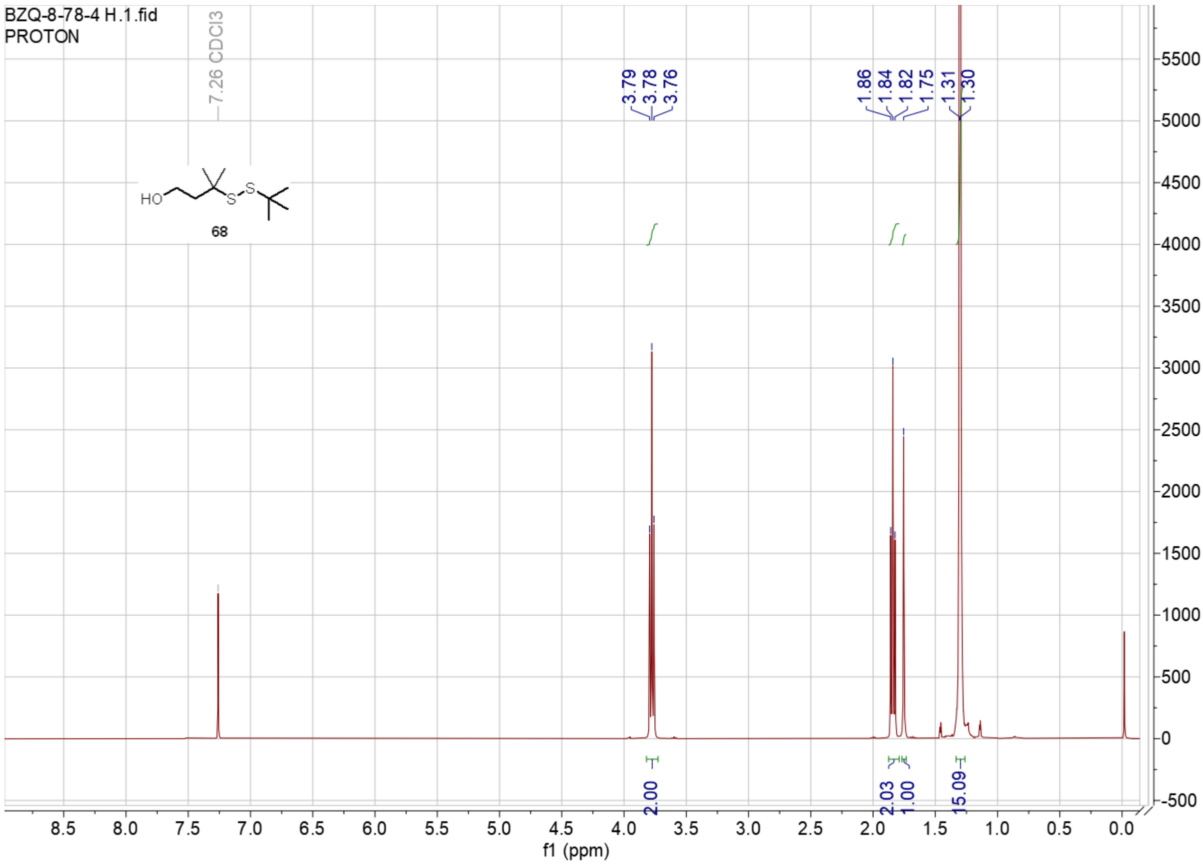

Supplementary Figure 253. ^{1}H NMR (400 MHz, $CDCl_3$, 293 K) spectrum of **68**.

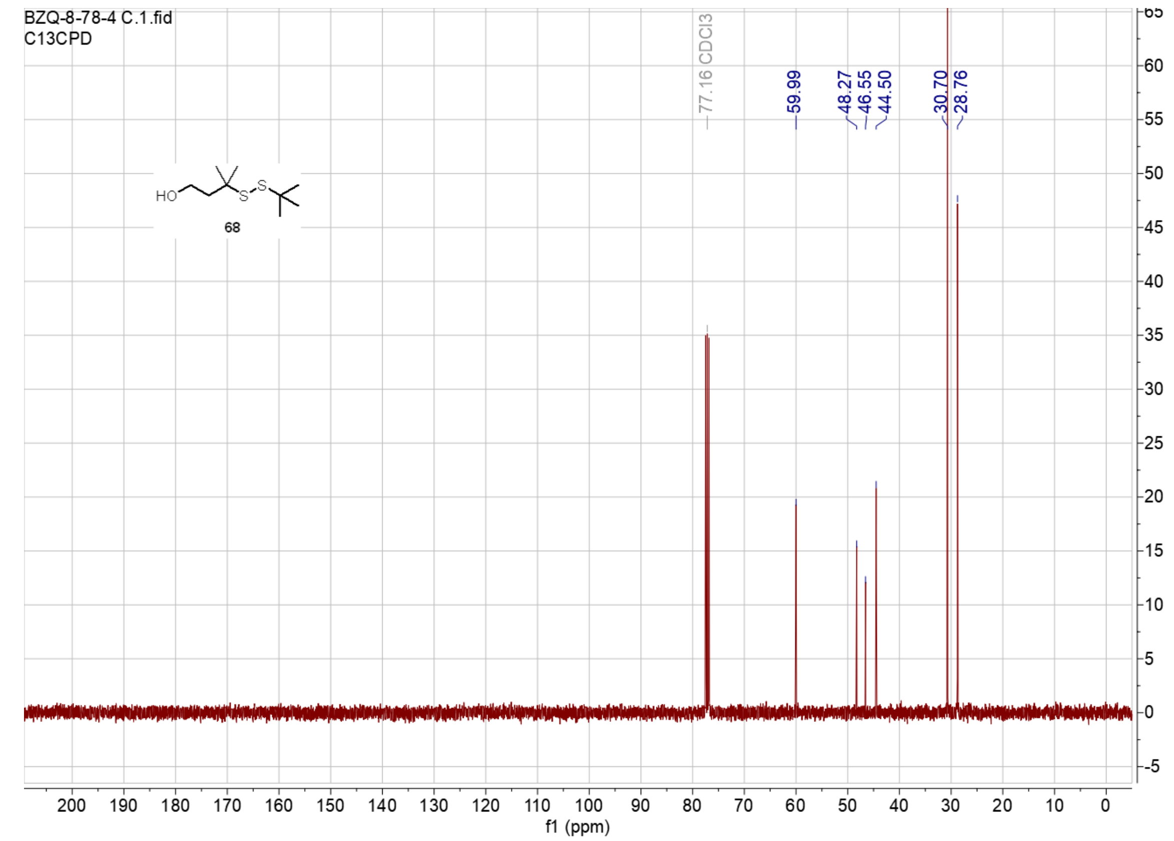

Supplementary Figure 254. ^{13}C NMR (101 MHz, $CDCl_3$, 293 K) spectrum of **68**.

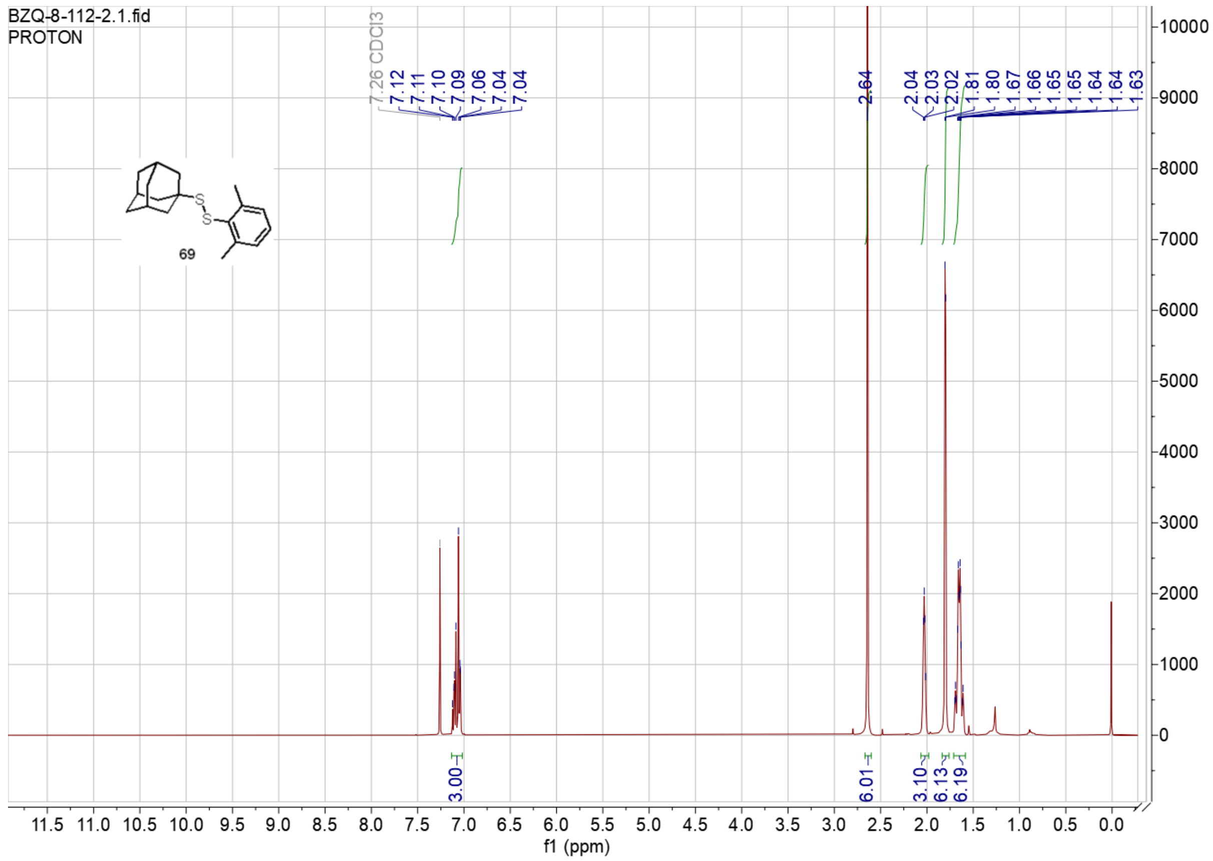

Supplementary Figure 255. ^{1}H NMR (400 MHz, $CDCl_3$, 293 K) spectrum of **69**.

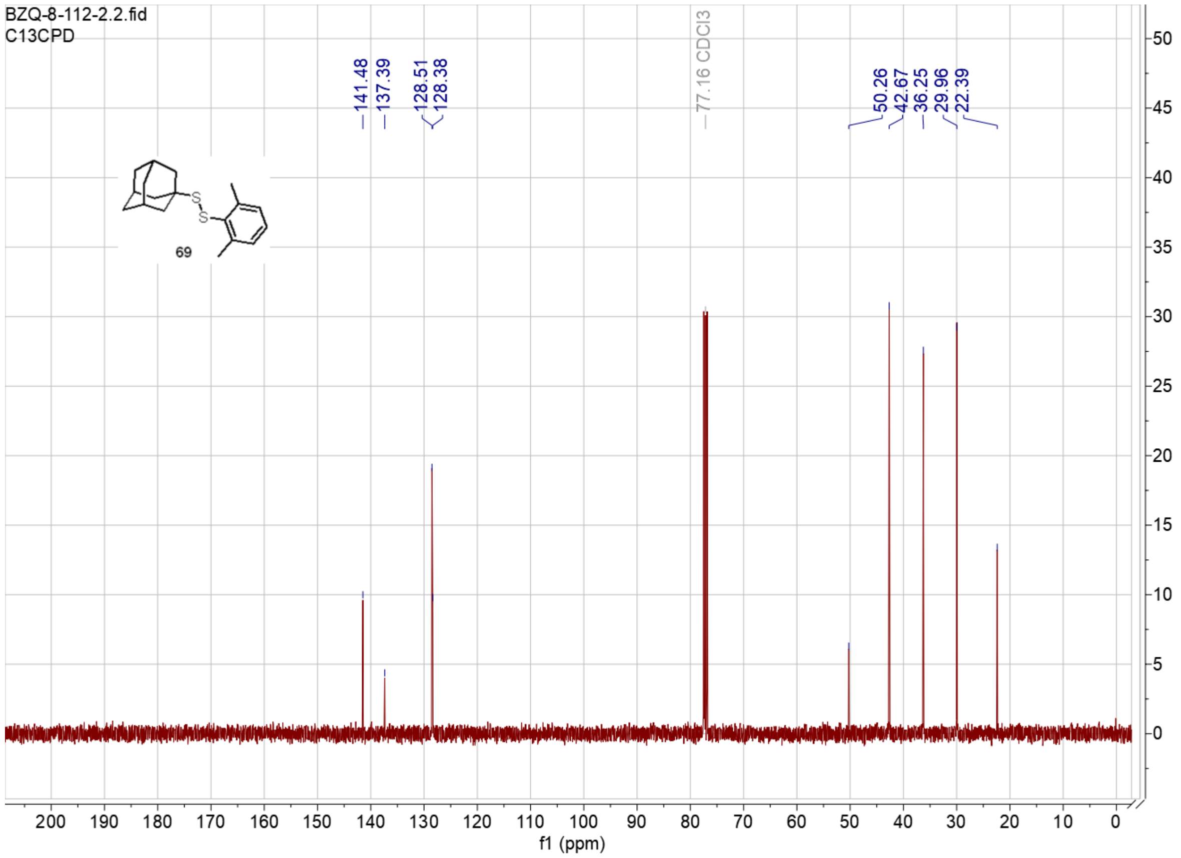

Supplementary Figure 256. ^{13}C NMR (101 MHz, $CDCl_3$, 293 K) spectrum of **69**.

Supplementary References

1. Hong, S. Y. & Chang, S. Stereodefined access to lactams via olefin difunctionalization: Iridium nitrenoids as a motif of LUMO-controlled dipoles. *J. Am. Chem. Soc.* **141**, 10399-10408 (2019).
2. Du, B., Ouyang, Y., Chen, Q. & Yu, W. Y. Thioether-directed nih-catalyzed remote gamma-C(sp^3)-H hydroamidation of alkenes by 1,4,2-dioxazol-5-ones. *J. Am. Chem. Soc.* **143**, 14962-14968 (2021).
3. Jung, H. Y., Chang, S. & Hong, S. Strategic approach to the metamorphosis of gamma-lactones to NH gamma-lactams via reductive cleavage and C-H amidation. *Org. Lett.* **21**, 7099-7103 (2019).
4. Tota, A. *et al.* Synthesis of NH-sulfoximines from sulfides by chemoselective one-pot N- and O-transfers. *Chem. Commun.* **53**, 348-351 (2016).
5. Yang, P. *et al.* Streamlined construction of peptide macrocycles via palladium-catalyzed intramolecular S-arylation in solution and on DNA. *Chem. Sci.* **12**, 5804-5810 (2021).
6. Kise, H., Whitfield, G. F. & Swer, D. Iminosulfuranes (sulfilimines). Thermolysis of N-acetyliminodialkylsulfuranes. *J. Org. Chem.* **37**, 1125-1128 (1972).

7. Banfield, S. C., Omori, A. T., Leisch, H. & Hudlicky, T. Unexpected reactivity of the burgess reagent with thiols: Synthesis of symmetrical disulfides. *J. Org. Chem.* **72**, 4989-4992 (2007).

8. Mampuys, P. *et al.* Iodide-catalyzed synthesis of secondary thiocarbamates from isocyanides and thiosulfonates. *Org. Lett.* **18**, 2808-2811 (2016).

9. Zhang, J., Wang, H. & Xian, M. Exploration of the "traceless" reductive ligation of S-nitrosothiols. *Org. Lett.* **11**, 477-480 (2009).

10. Song, L. *et al.* Natural gallic acid catalyzed aerobic oxidative coupling with the assistance of $MnCO_3$ for synthesis of disulfanes in water. *Green Chem.* **21**, 1432-1438 (2019).
